# Subtractive genomics and comparative metabolic pathways profiling revealed novel drug targets in *Ureaplasma urealyticum*

**DOI:** 10.3389/fmicb.2024.1484423

**Published:** 2024-10-30

**Authors:** Liesong Chen, Zhuojia Zhang, Qilin Zeng, Wei Wang, Hui Zhou, Yimou Wu

**Affiliations:** ^1^Institute of Pathogenic Biology, School of Basic Medical Sciences, Hengyang Medical School, University of South China, Hengyang, China; ^2^Hunan Provincial Key Laboratory for Special Pathogens Prevention and Control, University of South China, Hengyang, China; ^3^Special Inspection Department, Hengyang Traditional Chinese Medicine Hospital, Hengyang, China; ^4^Center for Medical Test and Pathology, The First Affiliated Hospital of Hunan University of Chinese Medicine, Changsha, China

**Keywords:** *Ureaplasma urealyticum*, subtractive genomics, metabolic pathways, virtual highthroughput screening, drug targets

## Abstract

**Introduction:**

*Ureaplasma urealyticum* is a commensal organism found in the human lower genitourinary tract, which can cause urogenital infections and complications in susceptible individuals. The emergence of antibiotic resistance, coupled with the absence of vaccines, underscores the necessity for new drug targets to effectively treat *U. urealyticum* infections.

**Methods:**

We employed a subtractive genomics approach combined with comparative metabolic pathway analysis to identify novel drug targets against *U. urealyticum* infection. The complete proteomes of 13 Ureaplasma strains were analyzed using various subtractive genomics methods to systematically identify unique proteins. Subsequently, the shortlisted proteins were selected for further structure-based studies.

**Results:**

Our subtractive genomics analysis successfully narrowed down the proteomes of the 13 Ureaplasma strains to two target proteins, B5ZC96 and B5ZAH8. After further in-depth analyses, the results suggested that these two proteins may serve as novel therapeutic targets against *U. urealyticum* infection.

**Discussion:**

The identification of B5ZC96 and B5ZAH8 as novel drug targets marked a significant advancement toward developing new therapeutic strategies against *U. urealyticum* infections. These proteins could serve as foundational elements for the development of lead drug candidates aimed at inhibiting their function, thereby mitigating the risk of drug-resistant infections. The potential to target these proteins without inducing side effects, owing to their specificity to *U. urealyticum*, positions them as promising candidates for further research and development. This study establishes a framework for targeted therapy against *U. urealyticum*, which could be particularly beneficial in the context of escalating antibiotic resistance.

## 1 Introduction

*Ureaplasma urealyticum*, which falls under the category of Mollicutes, is a prevalent mucosal commensal capable of self-replication and cell-free survival (Glass et al., 2000). This microorganism typically inhabits the lower urinary and reproductive systems in humans and can be transmitted through sexual contact (Kawai et al., 2015). Accumulating reports suggest that *U. urealyticum* is gaining recognition as a significant sexually transmitted pathogen. It is causally associated with various conditions, including non-gonococcal urethritis, infertility, chorioamnionitis (Yang et al., 2020), prostatitis, epididymitis (Pollack, 2001), cervicitis, bacterial vaginosis, pelvic inflammatory disease, reactive arthritis, spontaneous abortion, prematurity, intrauterine growth retardation, postpartum fever, and extragenital disease (Capoccia et al., 2013; Waites et al., 2005). Additionally, it poses a serious threat to newborns and individuals with weakened immune systems as it can lead to severe infections (Bharat et al., 2015; Deetjen et al., 2014; Pollack, 2001; Sprong et al., 2020). Noteworthy, *U. urealyticum* typically results in asymptomatic or chronic persistent infection with observable clinical symptoms. Currently, tetracycline, quinolone and macrolide antibiotics are generally considered the primary treatment options for *U. urealyticum* infection (Kawai et al., 2015). However, the rapid emergence of antimicrobial resistance among clinical strains has compromised the effectiveness of these available drugs (Zhang et al., 2023; Ma et al., 2021; Beeton et al., 2009; Kong et al., 2022; Yang et al., 2020). Consequently, the treatment of *U. urealyticum* infection remains a formidable challenge.

The urgent need to explore new therapeutic targets in this bacterium is highlighted by the global increase in antibiotic resistance and the absence of a licensed vaccine. The post-genomic era and advancements in high-throughput sequencing have paved the way for the development of well-established tools for analyzing big data. These tools have opened up new avenues for identifying novel drug targets. Currently, subtractive genomics analysis is universally utilized to discover potential drug targets against various pathogenic bacteria, including *Chlamydia trachomatis* (Aslam et al., 2021), *Vibrio parahaemolyticus* (Liu et al., 2023), *Mycobacterium tuberculosis* (Uddin et al., 2018), *Salmonella Typhi* (Jalal et al., 2021), *Staphylococcus aureus* (Naorem et al., 2022), *Streptococcus pneumoniae* (Khan K. et al., 2022), *Mycoplasma genitalium* (Nogueira et al., 2021), and *Haemophilus ducreyi* (de Sarom et al., 2018). This approach, which involves differentiating the pathogen proteome from the host proteome to identify non-host essential proteins, offers a more efficient and cost-effective alternative to traditional disease-based drug development methods. Through in-silico analysis, suitable drug targets for *U. urealyticum* can be explored.

To date, there have been no reported therapeutic targets concerning the metabolic pathways unique to *U. urealyticum*. In this study, we utilize extensive subtractive genomics and comparative analysis of metabolic pathways to uncover promising therapeutic targets against *U. urealyticum* by leveraging the existing sequenced genomes. Moreover, we performed virtual high-throughput screening of FDA-approved and FDA-experimental drugs, and identified potential candidates with inhibitory activity against shortlisted drug target proteins.

## 2 Materials and methods

**Figure 1:**
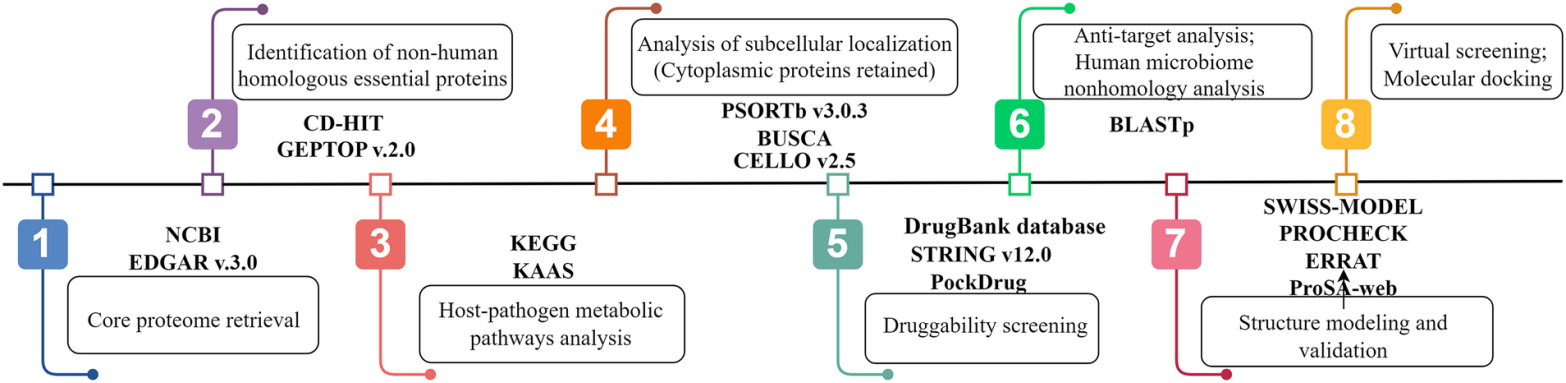
Systemic workflow of novel drug targets identification using subtractive genomics and comparative metabolic pathways analysis (By Figdraw).

The workflow used in this study for the prediction of putative drug targets against *U. urealyticum* infection is detailed in Figure 1.

### 2.1 Acquisition of genomics information and core proteomics investigation

The genetic information of selected strains within the *Ureaplasma* sp. (Supplementary Table S1) was gathered from the GenBank database at the National Center for Biotechnology Information (NCBI), which functions as a central repository for biomedical and genomics data. In order to maintain uniformity in genome annotations, the RAST server (Rapid Annotations using Subsystems Technology) (Overbeek et al., 2014) was utilized to annotate all of the genomes. The analysis of the core proteome among 13 genomes was conducted using reciprocal best BLAST hits of all coding sequences in the EDGAR version 3.0 software framework (Dieckmann et al., 2021). For this analysis, the genome of *U. urealyticum* serovar 10 str. ATCC 33699 was chosen as the reference genome, and the genomes of the remaining strains were compared to the reference.

### 2.2 Identification of human host non-homologous essential proteins

The duplicated copies or redundant sequences from the main protein set were eliminated by implementing the CD-HIT module within the CD-HIT suite (Li and Godzik, 2006). To be considered redundant, the sequence identity had to exceed a specific cutoff of 0.6 (or 60%). For the subsequent analysis, priority was given to the protein sequences that were not duplicates. To identify the essential proteins of *U. urealyticum*, the core set of proteins was further assessed using GEPTOP version 2.0 (Naorem et al., 2022). An essentiality score cutoff of 0.24 was utilized. The GEPTOP platform was employed to detect essential genes in prokaryotic organisms. This was achieved by comparing the orthology and phylogeny of the query proteins with the experimentally defined datasets in the database of essential genes (DEG). After identifying non-duplicated protein sequences deemed essential, a BLASTp search against the *Homo sapiens* genome was conducted using the threshold E-value cutoff of 10^−4^. Any resulting sequences showing significant similarity to the human host were disregarded, while sequences with no homology were chosen for downstream analysis.

### 2.3 Identification of metabolic pathways in the pathogen and human host

To identify metabolic pathways in the human host and *U. urealyticum*, we utilized the Kyoto Encyclopedia of Genes and Genomes (KEGG) and the Automatic Annotation Server (KAAS). Using the KEGG database, we retrieved unique identification numbers for the metabolic pathways in *H. sapiens* and *U. urealyticum*. We then conducted a manual comparison to distinguish exclusive from shared pathways. Pathways exclusive to the pathogen were labeled as unique metabolic pathways, while those found in both the human host and the pathogen were identified as common metabolic pathways. In this research, the protein sequences implicated in the pathogen’s unique metabolic pathways, as well as proteins annotated with KO numbers but not associated with neither specific nor common pathways, were screened for subsequent detailed analysis.

### 2.4 Analysis of subcellular localization

To prioritize proteins found in the cytoplasm for assessing potential therapeutic targets, we first utilized the PSORTb version 3.0.3 server (Yu et al., 2010) to predict the subcellular localization of these proteins. To enhance the credibility of the PSORTb version 3.0.3 findings, we subsequently validated them through the BUSCA (Savojardo et al., 2018) and CELLO version 2.5 (Yu et al., 2006) servers. The most accurate results from the localization analysis were determined by aggregating the data from these three servers. The final protein localization result was the same location predicted by two or more servers.

### 2.5 Druggability analysis of screened proteins

The ability of a target to bind with drugs and drug-like molecules at a high affinity determines its druggability. To evaluate the druggability of crucial cytoplasmic proteins, Blastp was used to search for them in the DrugBank database, accessible at https://go.drugbank.com/. DrugBank is a valuable resource for bioinformatics and cheminformatics, offering extensive information on drugs and their targets. Potential drug targets or drugs were identified by considering proteins with an E value below 10^−5^. In instances where no matches to known drugs or drug targets were found, these proteins were classified as novel drug targets of *U. urealyticum* (Khan M. T. et al., 2022). The DrugBank non-homologous proteins were then prioritized for druggability assessment based on druggability probability via the PockDrug server (Hussein et al., 2015) and their protein–protein interaction characteristics retrieved from STRING version 12.0 database (Szklarczyk et al., 2023).

### 2.6 Anti-target analysis of shortlisted novel drug targets

In the field of host cell biology, proteins that act as anti-targets are of great significance due to their ability to bind with potential therapeutic compounds designed to combat corresponding pathogens. Among the human population, a comprehensive search of the existing literature discovered 210 such proteins (Fatoba et al., 2021). Noteworthy examples of these proteins include P-glycoprotein (referred to as P-gly), adrenergic receptor, dopaminergic receptor, and ether-a-go-go-related protein. In order to minimize any negative consequences resulting from the interaction between these anti-target proteins and the proposed drug targets, a thorough analysis was performed. The novel drug targets were subjected to a search using NCBI BLASTp against these 210 anti-target proteins, applying the following criteria: an E-value of less than 0.005, a query coverage greater than 30%, and an identity below 30%.

### 2.7 Analysis of non-homologous proteins in the human microbiome

Accidental blockage or unintentional inhibition of proteins found within the gastrointestinal microflora may result in dysbiosis, significantly affecting the microenvironment and possibly causing toxicity with adverse effects on the human host (Sarker et al., 2023). Essential non-homologous proteins, identified as potential novel drug targets, underwent screening via BLASTp, applying an E-value threshold of less than 0.005, a query coverage exceeding 30%, and an identity percentage below 30%. This analysis utilized the dataset available on the Human Microbiome Project server^1^ under “43,021 (BioProject)” (Khan K. et al., 2022; Peterson et al., 2009). Proteins exhibiting less than 30% similarity were classified as novel therapeutic targets and proceeded to the subsequent phase.

### 2.8 Structure modeling and validation

The target proteins analyzed in this study underwent structure prediction using the SWISS-MODEL program (Waterhouse et al., 2018) accessed through the Expasy web server, known for its comprehensive homology modeling service. The accurate evaluation of the 3D model is of paramount importance in the field of computational structure prediction (Senior et al., 2020). Recent advancements in sequencing techniques have led to groundbreaking discoveries in computational structural biology (Mendez et al., 2023; Tripathi et al., 2019). The introduction of widely accepted and efficient techniques for structure evaluation has enabled the qualitative estimation of protein structures. In this research, three distinct tools, namely PROCHECK (Laskowski et al., 1996), ERRAT (Colovos and Yeates, 1993) and ProsA-web (Wiederstein and Sippl, 2007), were utilized to assess the stereochemical quality and accuracy of the predicted models (Hooda et al., 2012).

### 2.9 Ligand library preparation for virtual screening

To identify a suitable target for drug development, it is essential to investigate the interactions between the drug target and potential drug or drug-like compounds. Consequently, it is necessary to search for suitable ligand molecules that are capable of binding to the target proteins. In preparing the ligands library, we selected the FDA-approved drug library, which contains 2,470 drugs, along with an experimental drug library comprising 6,041 drugs (Gan et al., 2023). All drug molecules were obtained from DrugBank in the structured data file (SDF) format. The structures of these drug molecules were subjected to energy minimization and subsequently compiled into a ligands library designed for virtual screening.

### 2.10 Virtual high-throughput screening

The recently developed DrugRep server^2^ facilitated the execution of virtual high-throughput screening as an online tool for computer-aided drug discovery. In the process of virtual screening with DrugRep, the structures of well-prepared drug target proteins underwent receptor-based virtual screening against a library of ligands that included 8,511 distinct drug molecules. For the docking procedure, the DrugRep server employed Autodock Vina (Trott and Olson, 2010) to identify potential inhibitors against the drug target proteins and returned 100 best molecules based on estimated binding affinities (kcal/mol).

### 2.11 Receptor–ligand complex analysis

To elucidate the interaction dynamics between the receptors (shortlisted drug target proteins) and the ligands (the top-ranked drug molecules), the tools AutoDock Vina and PoseEdit were employed. The PoseEdit tool was sourced from the ProteinsPlus Server.^3^ An examination was performed on both receptors alongside their respective ligands to evaluate interactions, binding conformations, and affinities. Furthermore, the Top-10 screened drugs underwent filtering based on Lipinski’s rule of five (RO5). Ultimately, the ligands that demonstrated the most advantageous binding affinities were selected according to their optimal docking scores and adherence to RO5.

### 2.12 Molecular dynamics simulations

All-atom MD simulations were conducted using small molecule-protein complexes derived from docking as the initial structures, employing Gromacs 2023.3 software (Pronk et al., 2013; Rodríguez-Martínez et al., 2024). Both the small molecule and the protein were represented using the AMBER protein force field (Wang et al., 2004; Maier et al., 2015). The pdb2gmx tool was utilized to incorporate hydrogen atoms into the system, followed by the addition of a truncated cubic TIP3P water box at a distance of 10 Å from the system. Sodium (Na+) and chloride (Cl−) ions were included to neutralize the system’s charge (Bejagam et al., 2018). Subsequently, the topological and parameter files for the simulations were generated. MD simulations were executed with Gromacs 2023.3 software for a total simulation duration of 100 ns (Case et al., 2005). Prior to the simulations, energy minimization was performed using the mdrun command and the steepest descent method within the NVT ensemble, with an initial step size of 0.01 nm and a maximum force tolerance of 1,000 kJ/mol•nm. Following energy minimization, a 100 ps NVT (isothermal-isochoric) ensemble simulation was conducted at constant volume, with gradual heating from 0 to 310.15 K to facilitate the even distribution of solvent molecules within the solvent box. This was succeeded by a 100 ps NPT (isothermal-isobaric) ensemble simulation, employing the Berendsen barostat to equilibrate the system pressure at 1 bar. During the MD simulations, all hydrogen bonds were constrained using the LINCS algorithm, with an integration step size of 2 fs. Long-range electrostatic interactions were calculated using the Particle Mesh Ewald (PME) method, with a cutoff of 1.2 nm. The cutoff for nonbonded interactions was set to 10 Å, and these interactions were updated every 10 steps. The resulting trajectories underwent periodic boundary condition removal, after which root-mean-square deviation (RMSD), root-mean-square fluctuation (RMSF), and radius of gyration (Rg) analyses were performed.

## 3 Results

### 3.1 Prediction of the core proteome

In our study, a total of 396 coding DNA sequences (CDs) were discovered to be present in all 13 strains. These CDs were converted into protein sequences and defined as the core proteome (Supplementary Sequence file 1). In order to verify the uniqueness of the identified protein sequences, we employed the CD-HIT web server with a 60% identity cutoff. Our analysis conclusively showed that all 396 protein sequences were distinct from any paralogous counterparts.

### 3.2 Identification of human host non-homologous essential proteins

The 396 unique protein sequences underwent analysis using the GEPTOP server, identifying 170 sequences as essential proteins (Supplementary Sequence file 2). These vital proteins, crucial for the pathogen’s survival within the host, serve as potential targets for drug development. However, these proteins must not bear resemblance to human proteins to avoid potential drug-related complications. To address this requirement, the 170 essential proteins were compared against human (*H. sapiens*) proteins using BLASTp, resulting in 94 sequences identified as essential and non-homologous proteins (Supplementary Sequence file 3).

### 3.3 Metabolic pathways analysis

The KEGG database currently catalogs 44 metabolic pathways for *U. urealyticum* and 358 for human. By manually comparing these metabolic pathways between the pathogenic *U. urealyticum* and its human host, we identified 9 unique pathways specific to the pathogen and 35 pathways that are common to both the pathogen and the host, as detailed in Table 1. Furthermore, an analysis conducted using the KAAS server demonstrated that out of 94 proteins screened from the previous step, 92 had assigned KEGG orthology (KO) identifiers. Notably, among these 92 proteins, four were only linked with unique metabolic pathways exclusive to the pathogen, as illustrated in Table 2, and 21 were involved in neither unique nor common metabolic pathways (Supplementary Table S2). All of these 25 proteins were selected for further investigation. The remaining 67 proteins were excluded from further analysis because they were found to participate in the common metabolic pathways.

**Table 1:** Unique and common metabolic pathways of *U. urealyticum* with reference to human host.

Serial no.	Unique pathways (KEGG)	KEGG pathway ID	Total proteins
1	Methane metabolism	uue00680	6
2	Two-component system	uue02020	2
3	Quorum sensing	uue02024	13
4	Phosphotransferase system (PTS)	uue02060	1
5	Bacterial secretion system	uue03070	7
6	Biosynthesis of secondary metabolites	uue01110	29
7	Microbial metabolism in diverse environments	uue01120	20
8	Carbon fixation by Calvin cycle	uue00710	6
9	Other carbon fixation pathways	uue00720	4
**Serial no.**	**Common pathways (KEGG)**	**KEGG pathway ID**	**Total proteins**
1	Glycolysis/Gluconeogenesis	uue00010	8
2	Pentose phosphate pathway	uue00030	8
3	Fructose and mannose metabolism	uue00051	5
4	Oxidative phosphorylation	uue00190	12
5	Purine metabolism	uue00230	13
6	Pyrimidine metabolism	uue00240	11
7	Cysteine and methionine metabolism	uue00270	3
8	Taurine and hypotaurine metabolism	uue00430	2
9	Selenocompound metabolism	uue00450	3
10	Glutathione metabolism	uue00480	2
11	Glycerolipid metabolism	uue00561	4
12	Glycerophospholipid metabolism	uue00564	6
13	Pyruvate metabolism	uue00620	3
14	Propanoate metabolism	uue00640	2
15	One carbon pool by folate	uue00670	4
16	Thiamine metabolism	uue00730	3
17	Riboflavin metabolism	uue00740	2
18	Nicotinate and nicotinamide metabolism	uue00760	7
19	Folate biosynthesis	uue00790	2
20	Aminoacyl-tRNA biosynthesis	uue00970	51
21	ABC transporters	uue02010	22
22	Ribosome	uue03010	57
23	RNA degradation	uue03018	7
24	RNA polymerase	uue03020	4
25	DNA replication	uue03030	11
26	Protein export	uue03060	9
27	Base excision repair	uue03410	4
28	Nucleotide excision repair	uue03420	5
29	Mismatch repair	uue03430	9
30	Homologous recombination	uue03440	13
31	Sulfur relay system	uue04122	2
32	Carbon metabolism	uue01200	16
33	Biosynthesis of amino acids	uue01230	14
34	Nucleotide metabolism	uue01232	16
35	Biosynthesis of cofactors	uue01240	16

**Table 2:** Screened proteins involved in pathogen-specific metabolic pathways.

Serial no.	KO assignment	UniProt ID	Gene name	Protein name	Metabolic pathway
1	K02313	B5ZAH4	UUR10_RS00005	Chromosomal replication initiator protein DnaA	Two-component system
2	K06881	B5ZBR4	UUR10_RS02265	Bifunctional oligoribonuclease and PAP phosphatase NrnA	Microbial metabolism in diverse environments
3	K02078	B5ZC11	UUR10_RS02850	Acyl carrier protein	Biosynthesis of secondary metabolites
4	K00997	B5ZBN9	UUR10_RS02135	Holo-(Acyl-carrier-protein) synthase	Biosynthesis of secondary metabolites

### 3.4 Subcellular localization prediction

The analysis conducted with the PSORTb, BUSCA, and CELLO servers identified that among the 25 proteins screened, a significant majority, specifically 24 proteins, were categorized as cytoplasmic proteins, as detailed in Supplementary Table S3. This classification indicates that these cytoplasmic proteins might play pivotal roles within cellular processes, enhancing their relevance in drug development. Consequently, given their potential as effective drug target candidates, these proteins were selected for subsequent more in-depth investigation.

### 3.5 Novel drug target prediction

**Figure 2:**
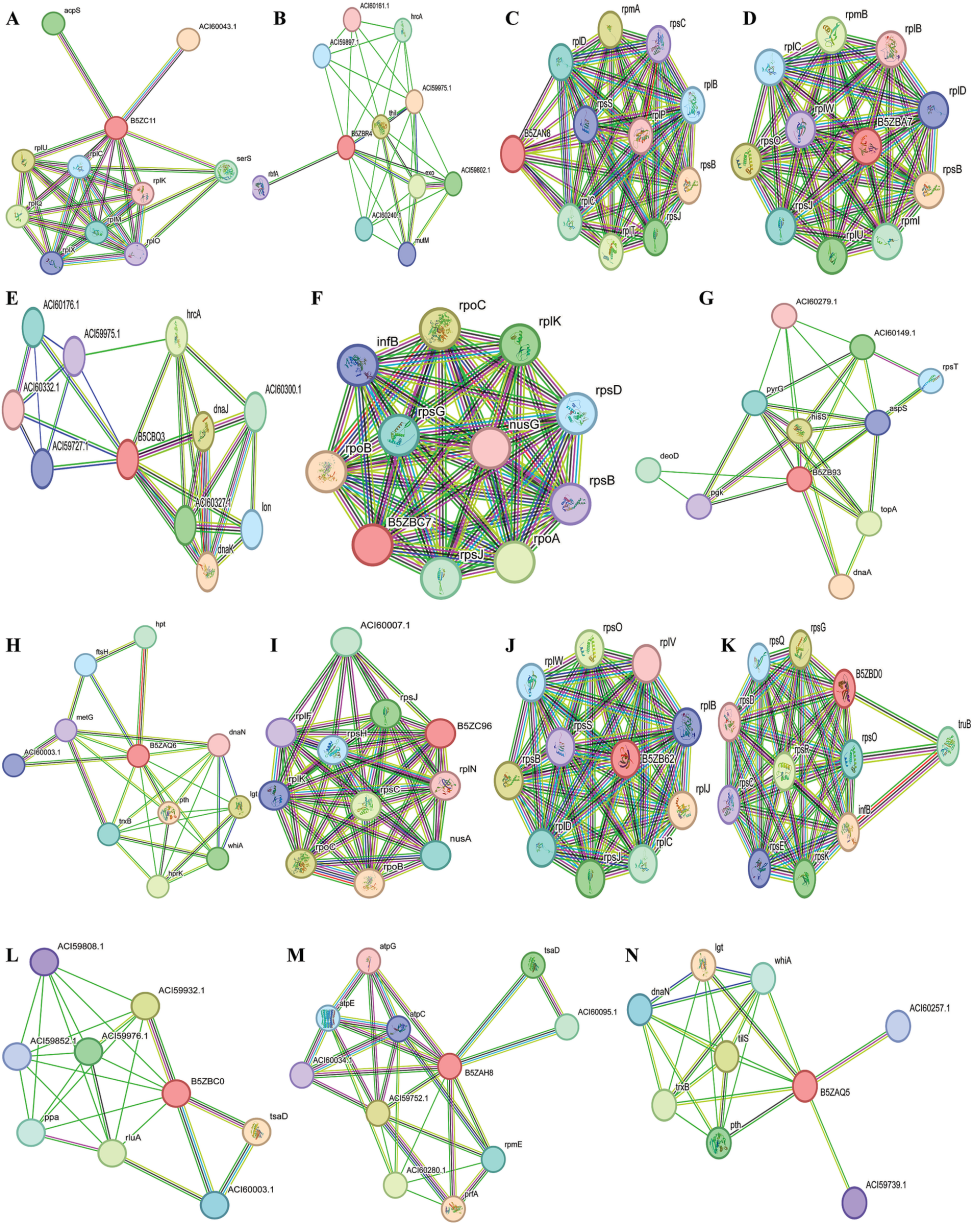
Schematic PPI network generated through the string v12.0 server for (A) B5ZC11, (B) B5ZBR4, (C) B5ZAN8, (D) B5ZBA7, (E) B5ZBQ3, (F)B5ZBC7, (G) B5ZB93, (H) B5ZAQ6, (I) B5ZC96, (J) B5ZB62, (K) B5ZBD0, (L) B5ZBC0, (M) B5ZAH8 and (N) B5ZAQ5.

A total of five proteins exhibited similarity to the available drug targets documented in the DrugBank database (Table 3). Conversely, the other 19 proteins displayed no matches with any entries currently identified in DrugBank. This observation indicates their potential as novel drug targets for *U. urealyticum*. Among those 19 proteins, 18 possess suitable binding pockets that have a druggability probability exceeding 0.5 (Table 4). Additionally, protein–protein interaction (PPI) analysis recognized 15 of these proteins as hub proteins, fulfilling the requisite threshold of a node degree of at least 5 (Aslam et al., 2021), as indicated in the STRING version 12.0 database (Table 4). Following the elimination of the five proteins that did not meet the specified parameters, each of the remaining 14 hub proteins was found to interact with several proteins (Figure 2). These hub proteins are integral to a variety of functions, and the inhibition of their activities may affect the functions of other interacting proteins. Moreover, these 14 unique therapeutic proteins lacked similarity to 210 identified human “anti-targets.” Furthermore, a BLASTp analysis conducted on all microbial strains from the Human Microbiome Project (HMP) utilizing the NCBI blast server demonstrated that only two out of the 14 proteins showed a similarity of less than 30%. Given their unique properties and the absence of overlaps with common host-pathogen pathways and human “anti-targets,” it is advisable to assess these two proteins (B5ZC96 and B5ZAH8) as promising candidates for drug targeting.

**Table 3:** Identified druggable targets with names of corresponding drugs.

Serial no.	UniProt ID	Gene name	DrugBank ID	Drug name
1	B5ZBN9	UUR10_RS 02135	DB01992	Coenzyme A
			DB04447	1,4-Dithiothreitol
			DB01812	Adenosine 3′,5′-diphosphate
2	B5ZAR1	UUR10_RS00430	DB00537	Ciprofloxacin
			DB01059	Norfloxacin
			DB01044	Gatifloxacin
			DB06771	Besifloxacin
			DB11943	Delafloxacin
			DB00218	Moxifloxacin
			DB00365	Grepafloxacin
			DB00467	Enoxacin
			DB00487	Pefloxacin
			DB00685	Trovafloxacin
			DB00978	Lomefloxacin
			DB01137	Levofloxacin
			DB01155	Gemifloxacin
			DB01165	Ofloxacin
			DB01208	Sparfloxacin
			DB01405	Temafloxacin
			DB00827	Cinoxacin
			DB04576	Fleroxacin
			DB09047	Finafloxacin
			DB12924	Ozenoxacin
			DB00817	Rosoxacin
			DB04395	Phosphoaminophosphonic
			DB05022	Amonafide
			DB05488	Technetium Tc-99m ciprofloxacin
			DB06042	ZEN-012
			DB06362	Becatecarin
			DB06421	Declopramide
			DB00694	Daunorubicin
			DB08651	3'-THIO-THYMIDINE-5'-PHOSPHATE
			DB00380	Dexrazoxane
			DB00773	Etoposide
			DB00997	Doxorubicin
			DB00970	Dactinomycin
			DB00276	Amsacrine
			DB00385	Valrubicin
			DB00444	Teniposide
			DB01177	Idarubicin
			DB01204	Mitoxantrone
			DB00445	Epirubicin
			DB01179	Podofilox
			DB01645	Genistein
			DB05129	Elsamitrucin
			DB04975	Banoxantrone
			DB04978	SP1049C
			DB04967	Lucanthone
			DB05920	RTA 744
			DB05706	13-deoxydoxorubicin
			DB06263	Amrubicin
			DB06420	Annamycin
			DB06013	Aldoxorubicin
3	B5ZC78	UUR10_RS03240	DB01752	S-adenosyl-L-homocysteine
4	B5ZC20	UUR10_RS02905	DB04082	Decyloxy-Methanol
5	B5ZBF9	UUR10_RS01745	DB08874	Fidaxomicin
			DB08226	Myxopyronin B
			DB08266	Methyl [(1E,5R)-5-{3-[(2E,4E)-2,5-dimethyl-2,4-octadienoyl]-2,4-dioxo-3,4-dihydro-2H-pyran-6yl}hexylidene] carbamate

**Table 4:** Druggability analysis of predicted potential novel drug target proteins.

Serial no.	UniProt ID	Gene name	Druggability probability	Number of pocket residues	Average node degree
1	B5ZAH4	UUR10_RS00005	0.87	25	4.36
2	B5ZC11	UUR10_RS02850	0.55	9	6.18
3	B5ZBR4	UUR10_RS02265	0.65	18	6.36
4	B5ZAN8	UUR10_RS00315	0.94	10	10
5	B5ZB36	UUR10_RS01080	0.39	8	10
6	B5ZBA7	UUR10_RS01445	0.95	11	10
7	B5ZBQ3	UUR10_RS02205	0.7	13.0	5.27
8	B5ZBC7	UUR10_RS01585	1.0	16.0	10
9	B5ZB93	UUR10_RS01370	1.0	14.0	5.45
10	B5ZAQ6	UUR10_RS00405	0.99	12.0	5.64
11	B5ZC96	UUR10_RS03335	0.86	12.0	9.45
12	B5ZB62	UUR10_RS01210	0.83	7.0	10
13	B5ZB23	UUR10_RS01015	0.99	10.0	4.55
14	B5ZBD0	UUR10_RS01600	1.0	11	8.73
15	B5ZBC0	UUR10_RS01550	1.0	10	5.56
16	B5ZAH8	UUR10_RS00025	0.79	19	5.27
17	B5ZAQ5	UUR10_RS00400	0.61	12	5.11
18	B5ZBI2	UUR10_RS01860	0.9	10	4
19	B5ZBB5	UUR10_RS01525	1.0	19	3

### 3.6 Comparative structure homology modeling and validation

**Figure 3:**
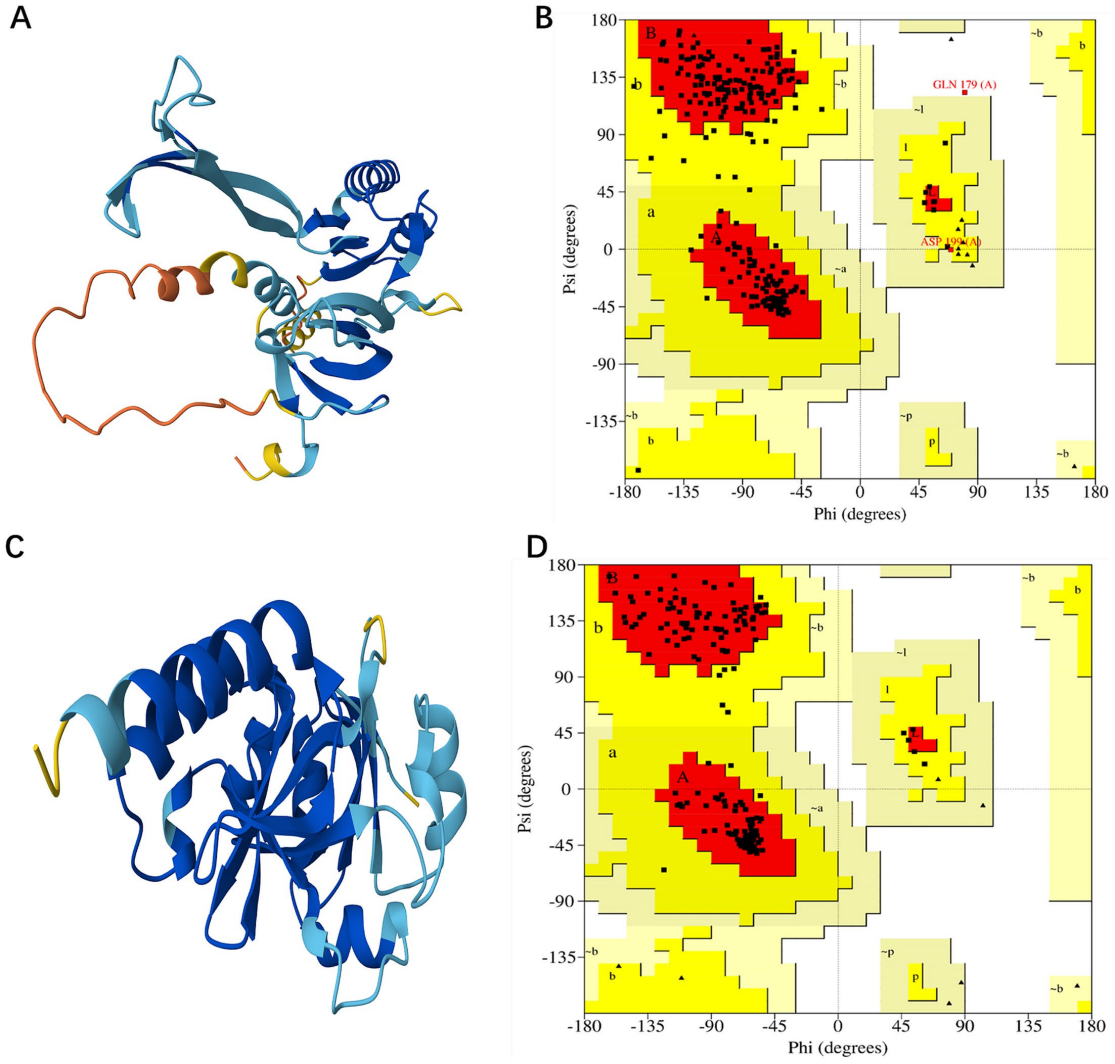
Modeled structure of shortlisted drug targets: (A) Three-dimensional structure of B5ZC96, (B) Ramachandran plot of B5ZC96, (C) Three-dimensional structure of B5ZAH8, and (D) Ramachandran plot of B5ZAH8.

Due to the unavailability of the desired protein 3D structures in the Protein Data Bank (PDB), the homology modeling for each protein was conducted by utilizing the FASTA sequences sourced from the UniProt database, specifically for the proteins with accession numbers B5ZC96 and B5ZAH8. The AlphaFold DB model of C0AGN1 was selected as the template for the homology modeling of B5ZC96 (Figure 3A) based on optimal GMQE values and sequence similarities, while the AlphaFold DB model of C0AGQ9 served as the template for modeling B5ZAH8 (Figure 3B). With these chosen template proteins, the 3D structures of both proteins were successfully modeled.

The validation of the developed models was performed using the PROCHECK, ERRAT, and ProSA-Web tools. For the protein B5ZC96, the Ramachandran plot revealed that approximately 88.1% of the residues are situated in the most favored regions, with 11.1% located in the additionally allowed regions, and both 0.4% of the residues found in the generously allowed and disallowed regions, as illustrated in Figure 3C. Conversely, concerning B5ZAH8, the Ramachandran plot indicated that nearly 92.9% of the residues resided in favorable areas, 7.1% in additionally allowed regions, and neither the generously allowed nor disallowed regions contained any residues (Figure 3D). Furthermore, the ERRAT plot provided an overall quality factor of 87.2038 for B5ZC96 and a perfect score of 100 for B5ZAH8, suggesting that the proposed models exhibit excellent quality. Additionally, the ProSA tool’s cross-validation of the modeled structures yielded a Z-score of −6.11 for B5ZC96 and −8.36 for B5ZAH8, indicating that these models are consistent with structures derived from NMR/X-ray crystallography.

### 3.7 Virtual high-throughput screening

The DrugRep server conducted a virtual high-throughput screening involving a set of 8,511 unique drug molecules. Our docking analysis indicated that the docking scores for the top 10 candidates ranged from −8.7 to −7.8 kcal/mol at the B5ZC96 active site, implying a significant affinity for this target protein (Table 5). Regarding RO5, the compounds 2-[3-({Methyl[1-(2-Naphthoyl)Piperidin-4-Yl]Amino}Carbonyl)-2-Naphthyl]-1-(1-Naphthyl)-2-Oxoethylphosphonic Acid, Irinotecan, and 3-(1 h-Indol-3-Yl)-2-[4-(4-Phenyl-Piperidin-1-Yl)-Benzenesulfonylamino]-Propionic Acid showed violations associated with molecular weight (MW), exceeding the ideal upper limit of 500 g/mol. Additionally, both 2-[3-({Methyl[1-(2-Naphthoyl)Piperidin-4-Yl]Amino}Carbonyl)-2-Naphthyl]-1-(1-Naphthyl)-2-Oxoethylphosphonic Acid and Pimozide went beyond the LogP constraint, exceeding the suggested threshold of 5. Furthermore, 2-[3-({Methyl[1-(2-Naphthoyl)Piperidin-4-Yl]Amino}Carbonyl)-2-Naphthyl]-1-(1-Naphthyl)-2-Oxoethylphosphonic Acid also violated the criteria for rotatable bonds, surpassing the preferred number of less than 10. Nevertheless, since Irinotecan and Pimozide have previously received FDA approval, it is unlikely that these two inhibitors will adversely impact pharmacokinetic properties. The remaining drugs complied closely with the RO5 criteria.

**Table 5:** Binding affinities as well as Lipinski’s rule of five of top-10 inhibitors targeting B5ZC96.

Drug ID	Drug name	Score	MW	HBD	HBA	RB	Rings	LogP
DB04016	2-[3-({Methyl[1-(2-Naphthoyl)Piperidin-4-Yl]Amino}Carbonyl)-2-Naphthyl]-1-(1-Naphthyl)-2-Oxoethylphosphonic Acid	−8.7	670.6895	2	6	11	7	6.4
DB07362	1-(5-{2-[(1-methyl-1H-pyrazolo[4,3-d]pyrimidin-7-yl)amino]ethyl}-1,3-thiazol-2-yl)-3-[3-(trifluoromethyl)phenyl]urea	−8.6	462.451	3	5	8	4	3.0
DB07360	1-{5-[2-(thieno[3,2-d]pyrimidin-4-ylamino)ethyl]-1,3-thiazol-2-yl}-3-[3-(trifluoromethyl)phenyl]urea	−8.5	464.487	3	4	8	4	4.4
DB00762	Irinotecan	−8.4	586.678	1	5	7	7	4.6
DB02449	3-(1 h-Indol-3-Yl)-2-[4-(4-Phenyl-Piperidin-1-Yl)-Benzenesulfonylamino]-Propionic Acid	−8.3	503.613	2	4	9	5	4.9
DB08896	Regorafenib	−8.1	482.815	3	3	8	3	4.1
DB08512	6-amino-2-[(1-naphthylmethyl)amino]-3,7-dihydro-8H-imidazo[4,5-g]quinazolin-8-one	−8.0	356.3806	2	3	3	5	4.5
DB01100	Pimozide	−7.9	461.5462	0	1	7	5	6.9
DB06210	Eltrombopag	−7.9	442.4666	3	4	7	4	4.7
DB15233	Avapritinib	−7.8	498.57	1	5	5	6	1.8

In addition, the binding energy for the leading 10 drugs concerning the B5ZAH8 receptor was significantly higher than that for the top 10 drugs targeting B5ZC96 (Table 6). The energy values for the top 10 drugs were established to be between −9.8 and − 9.0 kcal/mol. All top 10 drugs fulfilled the required RO5 parameters, with the exceptions of Zk-806450, 3-(2-aminoquinazolin-6-yl)-1-(3,3-dimethylindolin-6-yl)-4-methylpyridin-2(1H)-one and Arotinoid acid, each of which demonstrated a LogP violation exceeding 5, and Fluazuron, which violates both the LogP threshold greater than five and the MW limit of 500 g/mol (Table 6).

**Table 6:** Binding affinities as well as Lipinski’s rule of five of top-10 inhibitors targeting B5ZAH8.

Drug ID	Drug name	Score	MW	HBD	HBA	RB	Rings	LogP
DB03373	ZK-806711	−9.8	455.5746	1	1	6	5	3.7
DB07514	3-(2-aminoquinazolin-6-yl)-1-(3,3-dimethylindolin-6-yl)-4-methylpyridin-2(1H)-one	−9.7	397.4723	2	3	2	5	5.3
DB02112	Zk-806450	−9.4	489.6107	1	0	6	6	5.2
DB07586	5-(4-METHYL-BENZOYLAMINO)-BIPHENYL-3,4’-DICARBOXYLIC ACID 3-DIMETHYLAMIDE-4’-HYDROXYAMIDE	−9.3	417.4571	3	4	9	3	3.0
DB07397	(5S)-5-(2-amino-2-oxoethyl)-4-oxo-N-[(3-oxo-3,4-dihydro-2H-1,4-benzoxazin-6-yl)methyl]-3,4,5,6,7,8-hexahydro[1]benzothieno[2,3-d]pyrimidine-2-carboxamide	−9.1	467.498	3	5	6	5	2.1
DB02877	Arotinoid acid	−9.1	348.4779	1	2	4	3	7.4
DB06976	1-(5-OXO-2,3,5,9B-TETRAHYDRO-1H-PYRROLO[2,1-A]ISOINDOL-9-YL)-3-(5-PYRROLIDIN-2-YL-1H-PYRAZOL-3-YL)-UREA	−9.1	366.417	3	3	5	5	0.5
DB15583	Fluazuron	−9.1	506.21	2	3	7	3	6.0
DB07430	(10R)-10-methyl-3-(6-methylpyridin-3-yl)-9,10,11,12-tetrahydro-8H-[1,4]diazepino[5′,6′:4,5]thieno[3,2-f]quinolin-8-one	−9.0	374.459	2	3	1	5	4.0
DB07261	THIENO[3,2-B]PYRIDINE-2-SULFONIC ACID [1-(1-AMINO-ISOQUINOLIN-7-YLMETHYL)-2-OXO-PYRROLDIN-3-YL]-AMIDE	−9.0	453.537	2	5	5	5	2.2

### 3.8 Receptor–ligand complex analysis

**Figure 4:**
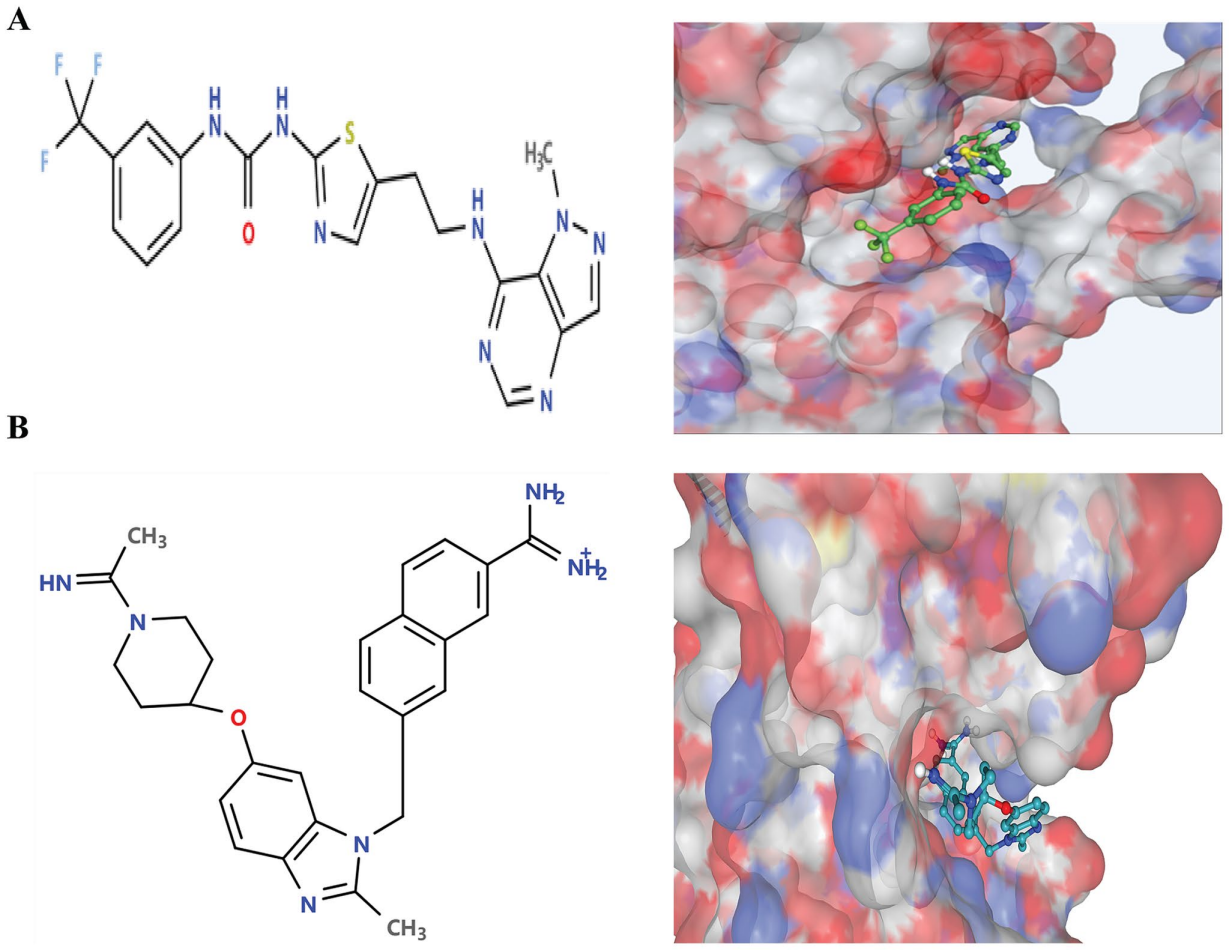
Two-Dimensional structures of optimal ligands and their binding configurations with respective target protein: (A) Two-Dimensional structure of 1-(5-{2-[(1-methyl-1H-pyrazolo[4,3-d]pyrimidin-7-yl)amino]ethyl}-1,3-thiazol-2-yl)-3-[3-(trifluoromethyl)phenyl]urea (left) and binding configurations with B5ZC96 (right), and (B) Two-Dimensional structure of ZK-806711 (left) and binding configurations with B5ZAH8 (right).

**Figure 5:**
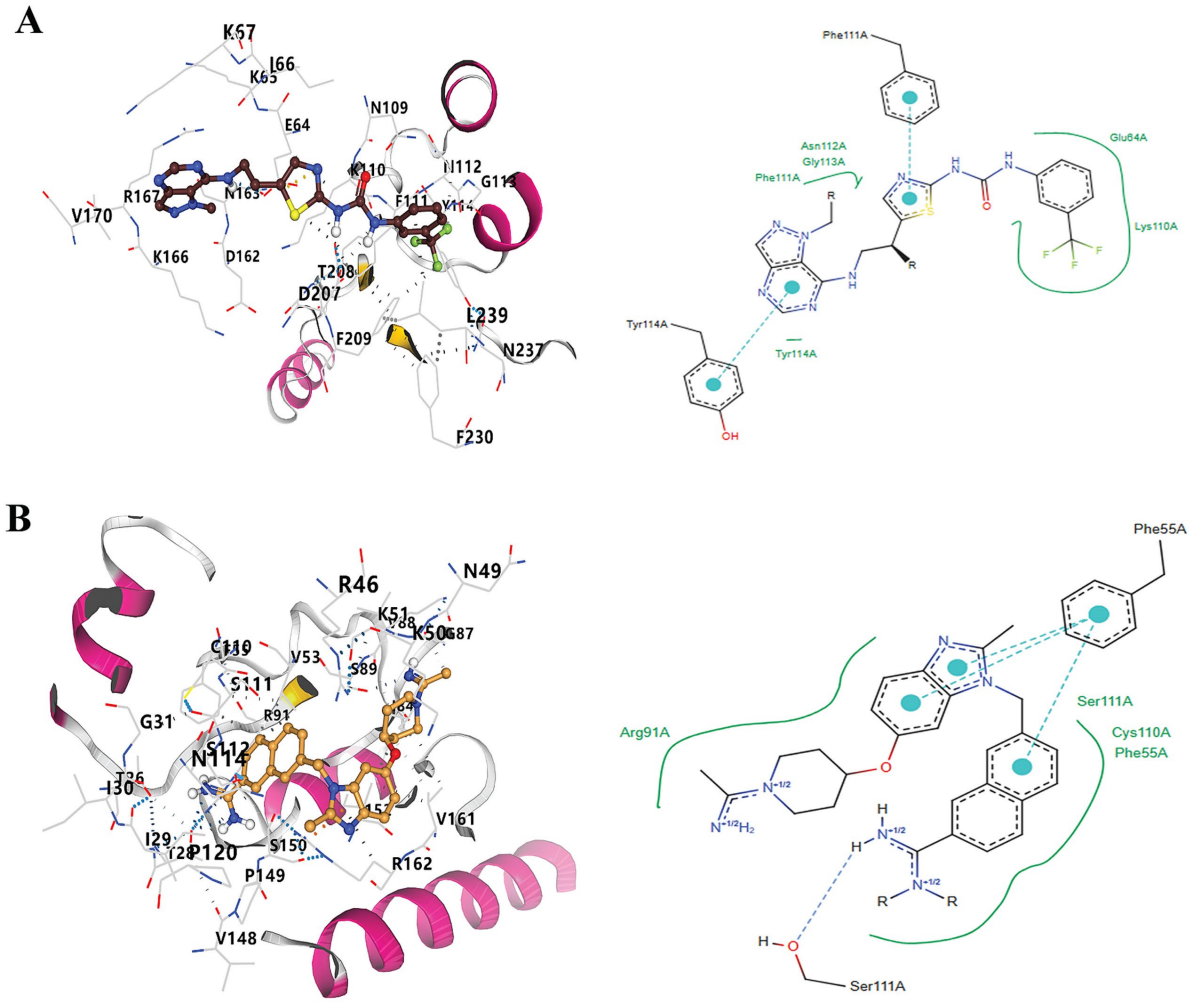
Docking of optimal ligands with their respective drug target proteins. (A). Three-Dimensional and two-Dimensional interaction diagram of 1-(5-{2-[(1-methyl-1H-pyrazolo[4,3-d]pyrimidin-7-yl)amino]ethyl}-1,3-thiazol-2-yl)-3-[3-(trifluoromethyl)phenyl]urea with B5ZC96, and (B) Three-Dimensional and two-Dimensional interaction diagram of ZK-806711with B5ZAH8.

Following the docking and sorting processes, the Top-10 molecules evaluated for each drug target demonstrated the lowest docking scores. According to RO5 filtering criteria, the compound 1-(5-{2-[(1-methyl-1H-pyrazolo[4,3-d]pyrimidin-7-yl)amino]ethyl}-1,3-thiazol-2-yl)-3-[3-(trifluoromethyl) phenyl]urea was identified as the best ligand for B5ZC96, while ZK-806711 was recognized as the optimal ligand for B5ZAH8. The two-dimensional structure and binding conformations of the best ligand for each target protein were illustrated in Figure 4. These binding configurations were generated utilizing the DrugRep server. Following the completion of the docking process, further analysis revealed that the compound 1-(5-{2-[(1-methyl-1H-pyrazolo[4,3-d]pyrimidin-7-yl)amino]ethyl}-1,3-thiazol-2-yl)-3-[3-(trifluoromethyl)phenyl]urea mediated two pi bonds with Phe111 and Tyr114 (Figure 5A), whereas ZK-806711 mediated three pi bonds with Phe55 and single hydrogen bond with Ser111(Figure 5B).

### 3.9 MD simulations analyses

**Figure 6:**
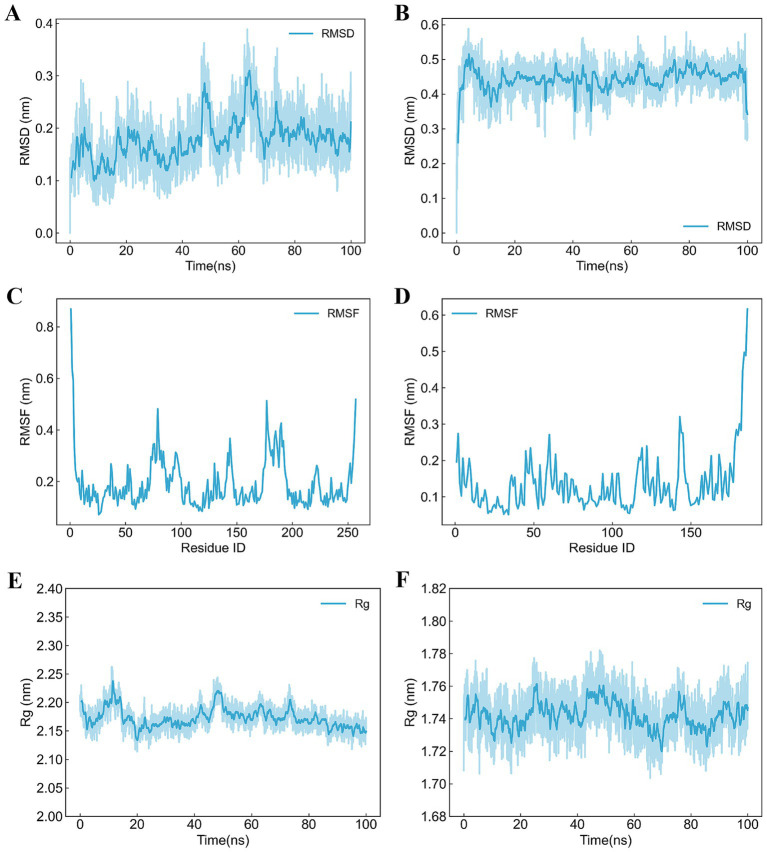
Trajectory analyses of receptor-ligand complexes over 100 ns molecular dynamics simulation. (A) RMSD analysis of B5ZC96–1-(5-{2-[(1-methyl-1H-pyrazolo[4,3-d]pyrimidin-7-yl)amino]ethyl}-1,3-thiazol-2-yl)-3-[3-(trifluoromethyl)phenyl]urea, (B) RMSD analysis of B5ZAH8-ZK-806711, (C) RMSF analysis of B5ZC96–1-(5-{2-[(1-methyl-1H-pyrazolo[4,3-d]pyrimidin-7-yl)amino]ethyl}-1,3-thiazol-2-yl)-3-[3-(trifluoromethyl)phenyl]urea, (D) RMSF analysis of B5ZAH8-ZK-806711, (E) Rg analysis of B5ZC96–1-(5-{2-[(1-methyl-1H-pyrazolo[4,3-d]pyrimidin-7-yl)amino]ethyl}-1,3-thiazol-2-yl)-3-[3-(trifluoromethyl)phenyl]urea, and (F) Rg analysis of B5ZAH8-ZK-806711.

The receptor-ligand complexes underwent 100 ns MD simulations using Gromacs. RMSD was employed to assess stability, with lower values indicating greater stability. For B5ZC96, the RMSD curve initially increased as the small molecule adapted within the binding cavity, stabilizing around 10 ns at 0.2 nm. Oscillations were observed around 50 ns but stabilized by 75 ns. Throughout the simulation, the small molecule maintained stable interactions, with minor adjustments reflected in the RMSD fluctuations (Figure 6A). In the case of B5ZAH8, the RMSD curve raised to 0.5 nm before stabilizing around 0.45 nm, subsequently dropping to 0.3 nm near the end of the simulation. The small molecule exhibited no significant displacement relative to the protein, maintaining stable interactions. Minor adjustments and the rotation of an alkyl chain contributed to the fluctuations observed in the RMSD curve (Figure 6B).

RMSF, assessed by the average deviation for each residue, was analyzed to evaluate receptor-ligand binding and protein dynamics. For B5ZC96, overall RMSF values were low, with increased values observed at the N- and C-termini due to their less constrained positions. Elevated RMSF in other regions may result from small molecule interactions or intrinsic flexibility (Figure 6C). Similarly, for B5ZAH8, RMSF values also remained low, with higher values at the N- and C-termini for analogous reasons. Increased RMSF in other regions might stem from small molecule interactions or inherent flexibility (Figure 6D). Low RMSF values suggest minimal fluctuations, indicating stable vibrations in the solvent environment and consistent sampling and analysis throughout the simulation.

Rg was a metric that reflected the compactness of protein structures and can also be utilized to assess changes in the looseness of the protein’s polypeptide chain during simulations. For B5ZC96, the analysis of the Rg curve indicated that during the initial 60 ns of the simulation, Rg exhibited fluctuations, gradually decreasing from approximately 2.20 nm to around 2.15 nm. In the subsequent 40 ns, Rg stabilized, exhibiting minimal fluctuations around 2.15 nm until the end of the simulation (Figure 6E). In contrast, for B5ZAH8, the examination of the Rg curve demonstrated that the protein’s Rg values oscillated within a narrow range of 1.72 nm to 1.76 nm throughout the entire duration of the simulation (Figure 6F).

## 4 Discussion

*U. urealyticum* has emerged as an important parasitic pathogen associated with various urogenital infections, underscoring the pressing need for effective treatment options. The management of *U. urealyticum* infection is further complicated by the increasing prevalence of antibiotic resistance. Commonly used antibiotics are losing their efficacy due to the development of resistance mechanisms, including genetic mutations (Kawai et al., 2015; Piccinelli et al., 2017) and biofilm formation (Feng et al., 2015; García-Castillo et al., 2008). This escalating resistance not only makes treatment more challenging but also raises concerns about the potential for treatment failures and the risk of recurrent infections. In light of the limitations of current therapeutic approaches and the troubling rise in resistance, it is imperative to explore new drug targets and therapeutic strategies. Therefore, addressing the challenges posed by antibiotic resistance and the need for innovative treatment options is vital for enhancing clinical outcomes for patients. Notably, the distinct biological characteristics of *U. urealyticum*, including its genomic adaptations and metabolic pathways (Pollack, 2001), offer a chance to discover new antimicrobial agents that can effectively combat the increasingly resistant landscape. Consequently, the primary objective of this study was to identify and evaluate novel drug targets through an integrated approach that combines subtractive genomics with comparative metabolic pathway profiling.

Through extensive proteome analysis, we identified 170 essential non-homologous proteins in *U. urealyticum*. To ensure the reliability of our findings, we employed a rigorous methodology. Utilizing EDGAR v3.0 software, we conducted a core proteome analysis on 13 *Ureaplasma* strains, encompassing 7,689 proteins and revealing 396 core proteins. Subsequent BLAST comparisons validated the consistent presence of these core proteins across all strains. We employed the CD-HIT server to confirm the absence of paralogous proteins and further analyzed the core proteome using the GEPTOP server, leading to the identification of 170 essential protein sequences. Our results were cross-verified with the DEG database, acknowledging that while this database may list proteins that are not essential during the *in vivo* infection phase, it has been well-documented in existing literature as identifying reliable novel drug targets. The 170 essential proteins are likely vital for the pathogen’s survival within the host. However, targeting these proteins may have detrimental consequences and disrupt metabolic processes. Therefore, we conducted Blastp analyses to select non-homologous proteins absent in the *H. sapiens* proteome, thereby minimizing potential adverse effects and cross-reactivity. Our analysis indicated that 94 of the 170 essential proteins exhibited no significant similarity to the human proteome. By focusing on these proteins, we could potentially eradicate the bacteria and address associated diseases. Consequently, these non-homologous essential proteins should be prioritized as targets in the future development of antimicrobial drugs and vaccines. It is meritorious to note that with a view to exhaustively validating our screening outputs, genome-level target knockout of 94 selected genes will be a necessary task in subsequent in-depth studies.

In a comparative analysis of metabolic pathways, we identified differences between the metabolic pathways of *U. urealyticum* and the human host. Previous studies have predominantly focused on proteins involved in the unique metabolic pathways of pathogens (Khan M. T. et al., 2022; Aslam et al., 2021; Alhamhoom et al., 2022). Our method also includes the proteins with KO numbers that do not participate in unique metabolic pathways or in shared metabolic pathways. This strategy not only enhances the specificity of drug targeting and minimizes potential off-target effects, but also broadens the effective scope of drug target exploration, thereby addressing the methodological limitations present in most prior studies. Our results indicated that out of 94 proteins screened, 67 were involved in common metabolic pathways, while 12 were involved in metabolic pathways unique to *U. urealyticum*. Notably, four proteins were found to be exclusively involved in specific pathways of the pathogen. Additionally, we identified 21 proteins with KO numbers that were not associated with any metabolic pathway. We conducted a Blastp analysis on these 21 proteins using the DrugBank server and discovered that seven of them were related to documented drug targets, suggesting that non-homologous proteins in pathogen-specific pathways may not be the only avenue for drug development. Consequently, our subsequent studies should include the 25 proteins derived from our refined screening criteria, which encompass proteins solely involved in pathogen-specific metabolic pathways and those with KO numbers not linked to any metabolic pathways. Furthermore, exploring cross-reactive proteins in shared pathways can yield valuable insights, as demonstrated by the successful development of pantothenate synthase as a therapeutic target across multiple pathogens (Umland et al., 2012). This underscores the necessity for a nuanced approach to identifying drug targets, considering both unique and shared metabolic pathways to enhance efficacy against *U. urealyticum*.

In this study, we approached the identification of druggable proteins in *U. urealyticum* through a comprehensive evaluation of their druggability probability, protein–protein interactions (PPI), anti-target analysis, and human microbiome non-homology analysis, adhering to specific thresholds. Following these assessments, molecular docking analyses were conducted to validate the findings. Statistical values were employed to select and prioritize suitable therapeutic targets, resulting in the identification of several prioritized drug targets against pathogenic *U. urealyticum*. Notably, novel targets absent from existing drug libraries exhibited significant interactions with multiple proteins, potentially serving as hubs during PPI network examinations. By leveraging the central lethality principle, developing knockdown models or inhibiting these proteins may effectively combat pathogen survival (He and Zhang, 2006). Under stringent criteria of comparative sequence analysis, a total of 19 essential and unique druggable proteins were prioritized as potential drug targets against *U. urealyticum*. Ultimately, two proteins, B5ZC96 and B5ZAH8, were selected for further virtual screening as potential drug targets. B5ZC96 is annotated as “Transcription termination/antitermination protein NusG,” a general transcription factor that has retained vital functions while rapidly evolving to meet the demands of bacterial pathogenicity (Wang and Artsimovitch, 2021; Tomar and Artsimovitch, 2013). Accumulating evidence demonstrated that compensatory evolution in NusG enhances the fitness of various pathogens, indicating that this protein could serve as a crucial molecule for pathogen survival (Eckartt et al., 2024; Cardinale et al., 2008). Conversely, B5ZAH8 is identified as “L-threonylcarbamoyladenylate synthase,” a critical enzyme in the synthesis of N(6)-threonylcarbamoyladenosine in tRNAs, which is essential for pathogen metabolism. Thus, these two proteins emerge as promising antibacterial targets for further exploration.

## 5 Conclusion

In the computational analysis conducted in this study, a comprehensive bioinformatics approach combining subtractive proteo-genomics with comparative metabolic pathway profiling led to the identification of two promising novel drug targets for treating *U. urealyticum* infections. While developing new drug candidates aimed at these protein functions has the potential to eliminate *U. urealyticum* from the host effectively, these proposed drug targets require further in-depth investigation and experimental validation. The findings of this study encompass all significant and viable drug targets associated with *U. urealyticum*, potentially aiding future researchers in the development of effective therapeutic compounds against this pathogen.

## Data Availability

The original contributions presented in the study are included in the article/Supplementary material, further inquiries can be directed to the corresponding author.

## Footnotes

^1^https://www.hmpdacc.org/hmp/

^2^http://cao.labshare.cn:10180/DrugRep/php/index.php

^3^https://proteins.plus/
