# Supplementary material for: Subtractive genomics and comparative metabolic pathways profiling revealed novel drug targets in Ureaplasma urealyticum
Source: Front Microbiol. 2024 Oct 30;15:1484423. doi: 10.3389/fmicb.2024.1484423 (PMC11557531; doi:10.3389/fmicb.2024.1484423)
Supplement: Supplementary file 1 [file Data_Sheet_1.docx]

Supplementary Material

# Supplementary Data

**Supplementary Sequence file 1 Core proteome of *U. urealyticum***

>UUR10_RS02285 Ureaplasma_urealyticum_serovar_10_str_ATCC_33699_NC_011374 heat-inducible transcriptional repressor HrcA

MKNNKILQEQIIEPKLSDRQKKVLKSIIDEYTITATPVSSKLLVQKEFQDQSSATIRNEMMLLEKFGFIEKQHISGGRVPSLKGYDFYNKNLINKNNDVSDNFKMRLHKILSKRYSNIDEILNAAVSIINETTQLPAVVTKSSSEELLKRIDLVKINDNSALVLIVTSSENILTHSIKLDKNTNFNDLQTCFSVLDERLVDTKLSLISSKLDLIVDIVRAKIEEAEYYFSKIVHRVFDFYAHKPTVNSKTYGVKYLTKHSEFEDQQKLNDLLNLLEDSTI*QQIALNKKTDGLAKIMLGNEIGHEHLAIATAQINLPNTNRQITVVGPTRMDYAKIKALLDFLKIEVEKILGSSNEH*

>UUR10_RS03090 Ureaplasma_urealyticum_serovar_10_str_ATCC_33699_NC_011374 50S ribosomal protein L1

MAKISKKLSAAYEGIDKQKAYPLFDAIKLAQEKSITKFDGSINIAVKLNLDTTKVEQQLRGSISLPNGNGKNVRVLVLSEDITKEQAAAVGADYFGGADYIQNIEKMLNQIDVIITNQKMMPLLAKLGKVLGPRGLMPNPKIGTVTNDVLKAVEEFKKGRIEYRTDTYGNIHMSIGRVSFETAKIEENANALLSLIRSKKPATVKGQYIQNIAISPTMGPGIKVIINNN*

>UUR10_RS00315 Ureaplasma_urealyticum_serovar_10_str_ATCC_33699_NC_011374 SsrA-binding protein SmpB

MIVSNKHARRNYELLEFFECGIVLKGTEVKSISRANCSINEAYVQIVKNEALILNMHVASFFEGNNFNQDPYRNRKLLLHKKEIIKLQHLVQTQRMTIVPTKIY*KNNKLKVEIALAKGKQLHDKREDLKKRDLARESRLF*

>UUR10_RS00735 Ureaplasma_urealyticum_serovar_10_str_ATCC_33699_NC_011374 ATP synthase subunit C

MSSFIDITNVISSHVEANLPAVSAENVQSLANGAGIAYLGKYIGTGITMLAAGAVGLMQGFSTANAVQAVARNPEAQPKILSTMIVGLALAEAVAIYALIVSILIIFVA*

>UUR10_RS00930 Ureaplasma_urealyticum_serovar_10_str_ATCC_33699_NC_011374 phosphate uptake regulator PhoU

MATNYFLMKESQCDLLNDFKNFFQMVIDNQKLICDLLKNDQSSYKETYEKACFLEKKVNTTYADMLEEIM*IIQRDQPRASYLRFFIAGINSIKDLERISDHSEIICEYFLETEFDEEQKNIFLTSFEISNQILSDLYDFFDKNEFSVNTIEHIKDLRSKSIMKINKNLIQSLHDQSKLLDENSNYIKLILMYRHIERNIEHGINIVQNFGNVHITSR*

>UUR10_RS01780 Ureaplasma_urealyticum_serovar_10_str_ATCC_33699_NC_011374 hypothetical protein

MKMKAKKFLITGLLGVVSLATITTVATACTNGNNQKANTVNNKSDLKTNPNINLTQQDVENLEKQRQQ*SEDLDVYFNGGLSKSGKNFDRINTLSSNATE*FKNHQKLAATRDSGSLVQDDAAYSFGIAPDYVHYK*VSKDGGDVSPSYLKPY*NKNLTEGRNFSKLDPKTLDDNGIGVLLISDHHYGK*NDNFLSKKTVGGLIMQSRVSGLPSHYPAVLTPKFLFDPNLNPNLKKYASANYALPNYYSDPLDGIILTGKTLDRIYDAKKFSNVYNAHVAGKTHFETFTDFAKAMVDETRRGISQ*SLKNDK*KDKTVLMVMPNFTPTAKMVDVNFKPEDLEFLSNVCINEPMYCPTIYSDPNDKYLPGLGAKFPIPVNHISQVQNYIDDYGQIGGKVLNGTGNPEASKQVGPTLGDAFKGTSDVVVFCYNEYGLLNYKPGTEEGEALVQQFEKNLTNYVNGLDINTKNNFNPTKMLKEKPVVGKNFFIIRKSSFYDAFLGFLGTIHNFNTFNK*MNGENARPIELNIPKFNKDNVQHIRAFKDDFKNK*

>UUR10_RS00345 Ureaplasma_urealyticum_serovar_10_str_ATCC_33699_NC_011374 oligoendopeptidase F

MENKKYE*DLDDLLNNKSLDDLYAEFEAKKAERIKAFATFLDSKENFAQ*QVLEEEFTIIANRFYNYVSNNLNTNVVDPK*NS*SQKLSASFYELETALSNYDSVILANEAKIKEYLKDPQLSVYTRKYDEIFRYQPHTLNDEQSKLYSKLARADEGFSTIYSTYTNNDMKFADAIDSKGKKHPINNEAEAFVHLKAKDRALRKSAYLSMYKAYYDSRESITKMLYYNYLSLNQQAKAKNFEDYIAKAAFDDVVDKSLITLIYDQVKLYKEANEDYKKARNAYLKKLIKVSKIEP*DNALPLISKKIDISIEEAKQMSIDSLSILGDEYVSNIKRAFDEK*VS*LPQKGKRGGAYSIGGTKGISKYYILMNYTNSLRDVQTIVHELGHSMHSLYSNRTQKIYSDYKIFYAEIASISNEVYLNYYLLEKYKDDLEMKLMILDEMISGFFATTTRQVIFSNFE*IANELINSGAPFTADVVMKEYQKLEFEYTNKPIVEDLTSIYSLSSVTPLRIPHFYVGNFYVYKYAVGQVAAIISGHRVFTKVEGAKQKVFDFLSSGGSKDPLDTIKLLGVDLTQPQS*QEALEIVKL*IKDYKQTIVLLNKKKSKK*

>UUR10_RS01165 Ureaplasma_urealyticum_serovar_10_str_ATCC_33699_NC_011374 type Z 30S ribosomal protein S14

MAKKSLIAKQKKHQKFAVREYTRCVRCGRPHAVNRKFGVCRLCFRDLAYAGAIPGIKKAS**

>UUR10_RS03080 Ureaplasma_urealyticum_serovar_10_str_ATCC_33699_NC_011374 ATP-binding cassette domain-containing protein

MQKLMDTFFKKKLKVPRPISDDISVRVKNLYAIYDEKQENELVALNNISYDFKKNKIYFIIGNSGSGKSTLVTHFNGLIISRYGFVQVGDIVSGDHYDLEHQLLGVIDSYDKKIINLL*KNQLDQ*TFLVLYSNEVNVQQARILFEANFKQKPVSLKFIKTKNNHELITNPYVRENTKIAVVRVDKNVFLEINDKMDYDELQRFEFIKKEIKTNYHLSKKLKRFKELRRRVGFVFQFPEYQLFKDTIEKDIMFGPINLGVKKSEAKKRAKFYLNKLGLGDDYLERSPFGLSGGQKRRVAIAGILAIENDILVFDEPTAGLDPAGEHEMMQIILDAKANNKTIFVITHTMEHVLEVADEVVVMDEGEIIKTGTPYEIFFDEHIINSTSIQVPRVIAVINELIKKDSKYEVLKQKQPRTIEELADAIVEFKKGGK*

>UUR10_RS01225 Ureaplasma_urealyticum_serovar_10_str_ATCC_33699_NC_011374 30S ribosomal protein S11

MAKKKKLSFTNGIAYIHATKNNTIITLADEQGSVLS*ASSGSIGYKGTKKKTPYSAGIAAEAAAKAVIDMGLKSVEVHVNGTGASRDTAIRSLQAAGLEVTKIKDVTPIPHNGCRPPKKPR*

>UUR10_RS01335 Ureaplasma_urealyticum_serovar_10_str_ATCC_33699_NC_011374 hypothetical protein

MGSNKTKKNRSTIQFLFGTIKKRKIANYLIIAFAFLTFALAIIGSFDAIASKYAIGIGSGLSLFITIGFSI*RVLMIHLGKE*NKSEIIWQSVMGAILVLSIIF*I*ATATTSVQYSALKEYFQLLKQKDQRASFIVLTQATTDQFGTALKGGL*A*VVTSCVYIFTNTFHNMFMKEKSPK*

>UUR10_RS01385 Ureaplasma_urealyticum_serovar_10_str_ATCC_33699_NC_011374 histidine--tRNA ligase

MSNYTKPRGTVDLYNEAMNEFKSLENFLLTTTKKYGFQQIKTPIFEFAELFMKSAGESSDLVSKEMYLFKDKSDR*LALRPEGTAGVIRAVVENKLLLNNPLPLKLMYFEPCFRYERPQAGRQRQFHQFGVEVLGTKNIYYDFELIALANNILKKLAISDYVLEINYISTAHNRSL*VKSLQEYFNLYRDELTPLSQERITTNPLRILDDKLESQKLVVQQAPKITNFLSNEEKEEFALIKKMLDEHDIKYRVNEGLVRGLDYYSGLVFEFISTSPRLLGQSTIIGGGRYGQLIKQTGGPDYEGIGFGIGIERLLIALLDSNKQILNNFEDKYLIAYFDKELENEAIKLTQSLRINNQLNVDIILDTIKADKIFRLAQRLNAKKLIILAKKE*LNKQVILKDLLSFEQKTLNLDEIKKIKE*

>UUR10_RS01565 Ureaplasma_urealyticum_serovar_10_str_ATCC_33699_NC_011374 inorganic diphosphatase

MKLNVTIEIPKNSNIKYEYDRATKEITVDRILYGSMVYPHNYGFLKEALDYDGDELDVLVFADQAFQPGIKVPARILGAMKMIDGGETDTKLLAVIDVDPRYKHINTFKDIPLH*LAEVQDFFENYKNLQNKKVEILGFEDEV*AQKEYEECVALMQEHGHLKKDEFVSKMMKQRPEKYSQ*

>UUR10_RS03010 Ureaplasma_urealyticum_serovar_10_str_ATCC_33699_NC_011374 5-nucleotidase, lipoprotein e(P4) family

MKLLKSKKF*AISLSSILVGASVVAAATACTNSSIESRVSTTFAKTQSGIYAIYEITN*SKLDANEKKSLESLKFTASVIDKDGKAEFTASTGILKKDKVYVKLPREPKADDRVVVKPDNANLKIGAVYVTTLNVSSVELNDGSKIDNNTNQKDESKPYNSVESIIYNQR*LANV*NTLSAEKDGMLLTAYNSAKHQFDAMVKQDAFDTNKVKVEKDASGNITKVTVSNPDSGKAIPVVFMDIDETILNNYANQNYQLLNNKAYSPRD*DLFVADKASKRLAGAFEFIKYV*EHGGVVMFNSNREQSSHIEPTVENLVSEGLDRALLPK*VF*MQGIDFASDKP*DNVKKDAKGKRVKSTKEDRMNTMNERTQGYDLSAFGSGNAVVLKTVMRVGDNFDDFNDNASKGKTNAERVALLKEYGKLFGNFDTKNTKGIKYKKDATSGKIVKSDET*SESYVMIGGNSSYGGFESGIAKGYFGLSKEDQVKALREYVKQLY*EPKNQNK*

>UUR10_RS00255 Ureaplasma_urealyticum_serovar_10_str_ATCC_33699_NC_011374 hypothetical protein

MMNTKTFTSVNRVIYDDNYSLKQQQKSSFINQFLKGLLSAITLLFFILLLIFAENTLFGLGFGDENKSMMISKSLNAFFDLHSPKYLQLNFLIVFRFFILSFTLFYALIKNFTNLYWHRVTIKKYLP*FVLYLVIATISFLLFFTFFSV*PKEVFNLVFLLLVLFLLNLSYEIFNYFISK

KTNPLLYGNYKNLIITMVFQALLLLFVIITPFV*INTGKSPNFLFVDNRFYTRIVDIFTVQSGKNFIILIAFFFFLITFIVLANTNFFALVINKRYDRNYVKNNL*FILLLFSAIFI*LLRVFAYKHENENLPIGNNHLL*VYILQSFFAIIILILYMVFTLKKRLSAKSSLNTLLNLVVTQTILSLSLFLVTLFNSKSVVSLINVFITITVQMSVFGFYIFQNKNISTKLLVLLKVIMILIILTAAIVGFDYLLTSDHHNNYLFSNIQPKMNLVQIMLLLNFSLSFTLISYLTIKFTMVIFKINKLNKELNNEKK*

>UUR10_RS00995 Ureaplasma_urealyticum_serovar_10_str_ATCC_33699_NC_011374 ribosomal-processing cysteine protease Prp

MIKINHYPNALLVKGHANFDEHGKDIVCAGVSAIIMGALN*FDQQKTTIKVEQGFILIIIDDNNQLYRQYLELIIIQLKAIYFKYQSYIELHEYKQQYKRGL*

>UUR10_RS01080 Ureaplasma_urealyticum_serovar_10_str_ATCC_33699_NC_011374 translation initiation factor IF-3

MNTPNNQRHMSSNNDARKNQPLINDQIRFRTMVVIDDHGNNLGEMNRIDALNLATSKNLDLVVIAKKGNIPVTKILDYGKYKYEQKRRQKESRKNQTIIKVKEIKIKPMIGEHDLKVRAENAKR*LEDKDNVKFVIEARGRMCTKDEFILQAYEKFIDLIKDYGTVVQANKKVSNYRYETIIEPIKK*

>UUR10_RS02990 Ureaplasma_urealyticum_serovar_10_str_ATCC_33699_NC_011374 elongation factor Tu

MAKAKFERTKPHVNIGTIGHVDHGKTTLTAAISTVLAKKGQAIAQSYADVDKTPEERERGITINASHVEYETKTRHYAHVDCPGHADYVKNMITGAAQMDGAILVIAASDGVMAQTKEHILLARQVGVPKIVVFLNKCDFMTDPDMQDLVEMEVRELLTKYGFDGDNTPVIRGSGLKALEGDPV*EAKIDELMDAVDS*IPLPERSTDKPFLLAIEDVFTISGRGTVVTGRVERGTLKVNDEVEIVGLKDTQKTVVTGIEMFRKSLDQAEAGDNAGILLRGIKKEDVERGQVLVKPGSIKPHRTFTAKVYILKKEEGGRHTPIVSGYRPQFYFRTTDVTGAISLPAGVDLVMPGDDVEMTVELIAPVAIEDGSKFSIREGGKTVGHGSVIKTSN*

>UUR10_RS00150 Ureaplasma_urealyticum_serovar_10_str_ATCC_33699_NC_011374 hypothetical protein

MNLKAKRFLKVITIISPIIIIPTIIASCAQTNSSTPNNPVKTPKSSVEVVNINPYKTKQMLASNINKEQINAYFIFVFNLIKEDQKIEEKILEKNDYEIIN*AGNDNDGTLSINVLIKATKQSYLISTQAVFLTNKQNSYLQELKYKTPAVYSIDDILKLSNDPYNLIYNNPYYQEQLLR

AKVYFNQNEANIADSKLYMNKIGYSEFGDDVQKRLENAFKVRYDEQNIYQINSPQILVKETKTVTSYTDEQTNNSYSINVELIKKLIQINPFGKLPTNFAQLVNLIKKEEYPKFLTITKNEPINNVVVKDIYYRIIDRYAKLEFILEIYNQKTKQTVYLSANFNQKNSQLLKNEDYFQYIFDRTISLNLLTTKDGKNVELNSGTG*IVDRIVDDSLPKNKIKLLVATNNHVMS*SNLAISKDNKTKSR*FSKQEYANYLENNAGLISSNIYEDKDRYQCLL*GTAPLKSPVSNKYNSLSGISFSNLAKVYNITSQNFVNRA*YLPQLSANGIKINEELKT*YQVNQESIKSIKNGTLDFALVPMVFDIEDIKEKLPNYYKVLNTKDEAN*YIGLGNSKKYLPQLQLFSGGYPGDRNFNSSAIVS*RGSKSYGSLIQAFDREIENESILDYYGPKRINNIDAYQKVDEGYLNKLFNVGTRVITSDEIGDLGSGSSGSMIIDSNFNLVGIHFASLNSRAYGAPNDSMIGNLFVAQSQDLSGDIDVRAAVIKKLKAENIYTYKLNPKVSS*

>UUR10_RS00375 Ureaplasma_urealyticum_serovar_10_str_ATCC_33699_NC_011374 hypothetical protein

MHKKTKIYLLGTFGLLSFGISVATIASSCTKTSSTNIQEQIKYDIMPTYTSQADNLLALGITPDYYPQQMY*KKKAPYDYLNPQKSDYQK*FYEKDNFANQFKEKLTSLEKDIKQYGTT**SFSGNTYGEGVSEEY*NKNKGKLVYYDRYLIDNSHFERAKKSLVAHDGPISNPNTIIPVDFKMSRDFYLTLNKDDILNFKNKPNSLLRKSLLLGLEYKDKEKGELNELYNYSYIANKLQEDYFNGQTFDGINFNNSAFKKRVLEGNDIPIFYTEKN*NNPKSLNSKIYEALKTVVLTKKVRESGLNHLNYNPFSPTIKQDDYITSILQHHPIYEQNMR*DGGTQVYLGTMRDSLLYLYDIAYATTKYAYSEQAQIDFKDNLAKLEPLKKALFNANQIAKNLIERLNKIRAYFQAVGVVDKNYNPDLKQFDNTNSKTLGLLTTSQNSGSSTLQTQSKYGFLYYDLGFKAPKPILKKDE*TELLEDVNGKQLLCHRHGNGQIHCFGGGDSQDGVQVDKNIVGSLFNMDDNG***NLGEGSLSASNFAKFNSQFDYLIKISYQNLEDFHSNNLTRPQKELIMSLIKPNYEDPANRVFDDNYEL*NDGIKSPIGYNMILDSIIRIINKTIDPQVIKQNEHLLREANN*GSY*DTFVNSK*

>UUR10_RS00910 Ureaplasma_urealyticum_serovar_10_str_ATCC_33699_NC_011374 methionine--tRNA ligase

MLKQKKFFISTPIYYSSGNPHIGHAYTTIIADVLARYKRLFGYDVFFLTGMDEHGQKIQQKAFEENISPKALVDRNSIIFLNL*KRLHISFSKFIRTTQMDHEESVQKVFSYLYKQGKIYLGQWTGYYCVSCEENYNPAEIIKSQDNIMLCRMGHKLETKSEESYFYKMSDQAPFLKTYYQNHPNFIIPNERANEMVNNFLNNLEDLSISRTTFD*GIPIAENPKHVIYV*LDALMNYLTATGYLSNNEELFQKY*CDNETEIVHLLSKEIARFHCIY*PIFLNDLQIRFPSTILSHG*IITKEGKMSKSLGNVIDPNVLIDTYGVDALRYYLMADLSLFRDAIFSEDNLIETYNTQLANSYGNMISRTLGMLKKYRNNIVPKYVGCVLKNDEKLENLINKNIELVQENINKYSIDKALNCIQEILVEANKYVEDNKP*ELAKNQQKQELDSLLVHLVKVIQVTTTLLSPILIEGSKKAVEQLNFDESFLTLASLASYDIFNYHKVNDSKPIFARIIVEKQ*

>UUR10_RS01340 Ureaplasma_urealyticum_serovar_10_str_ATCC_33699_NC_011374 nicotinate phosphoribosyltransferase

MGPVIPNTRLIDFKFDRDLLNKAYTSHYFIKTCKIIELHAPSHSVIMQFTHFSKTPIMVCGTSEVLALLEFCLSRKELKQLKIYYVPDGHVIKPKEALFAIEGPYEIFG*LENIIDSILARRSSVATNCYNVLNVINDEQKVIYMSDRSDDYSLQPYDGYAAAVGGMQYFVTQKQVEFLKDINYECKVMGSMPHALIQQNNGRVDLACEMFAQTFPNDPLIAVIDYNNNVLNDLEQLRYMFDRLYAVRIDTAKDLIDNSLLSTFDNVRNHDLHGCNPYLIDLVREYLDNNGGEHIKIIASSAIDLNSIKNFNKHNSAIDFYGIGTYLTHLSIHITADLVCLDNVYGAKVGRKIAKNFAEMTLY*

>UUR10_RS01390 Ureaplasma_urealyticum_serovar_10_str_ATCC_33699_NC_011374 aspartate--tRNA ligase

MRVYCGRIGKEHLEKNVILNG*VKKVRKMGNLVFVDLKDRFGIVQIFATKQDEVFDELTQLSREDVINVEGLVLLRKSPNHELKTGEFEVHAQKLLIYSKAKTPPLIIEDETDANEEIRFKYRYLDLRRDVNLRTFELRSKVYQTFRNYLHSEEFIETETPILAKPTPEGARDFYVPTRTKKFYALPQSPQTFKQLLMVAGFQKYFQIAKCFRDEDLRSDRQPEFTQVDIELSFADELEIQTLIENLFKHVFKQTINVDLTTPFVRMSYEQAINDYGSDKPDLRFDLKLKTLDTYFKTSKSQIFQKALSNNQSIRAILVPNVNLNKKQIQSLEKFAKDKGAKGLA*ISIENEKVIDGSLSSIKEDHVIYQTIFKDYHLSTGTILLIADEFDIASQSLGLVRVNLASMLNLKKPNDFKFV*IID*PLYEYDDENQRFVAAHHPFTMPTLETLDTFDIDQKNAKGRSYDIVLNGYEVGGGSVRIINQQIQRRMFKSINMSDEEANLKFGFLLNAFEYGVPPHCGIALGLDRLIMILINSEYIRDVVAFPKNNNGVDMMLDAPASMNDEDLKELGLIIKND*

>UUR10_RS02160 Ureaplasma_urealyticum_serovar_10_str_ATCC_33699_NC_011374 hypothetical protein

MKFKRFDSKKSILRFLHVDEAKREINLSFNDTTDFIFFLKEIAEDPNIAKKNAKKMTNLYVEISDKLGNDDNTDLSAYETKCNNLIEACVIDEAIFLIYMAKNMIIFLAHNKLDILQKDFFDKLLLIESHFSFDQNLSYSYIKNSSIALLIFHLKQFVAVYDEFLNDFEKVINDVLENAIPTKTKLNQQINHLHTLKRRPS*

>UUR10_RS00010 Ureaplasma_urealyticum_serovar_10_str_ATCC_33699_NC_011374 50S ribosomal protein L31

MKDIHPVSKPCVYNCVTCKKEFVINSAAKNTEVAIEVCSNCHTFFIGKQNATTTLRGRAEKLNNRFEAGLNNINKKPEKKKVQGKSEPRKSLNEL*

>UUR10_RS00950 Ureaplasma_urealyticum_serovar_10_str_ATCC_33699_NC_011374 30S ribosomal protein S15

MAVSKQQKHDLTVKFGGSASNTGKTEVQVAILSAEIDSLTTHMIENKKDKASKRGLYKKVAQRKKLLSYLQRVDIERYRALIKELNLRG*

>UUR10_RS01095 Ureaplasma_urealyticum_serovar_10_str_ATCC_33699_NC_011374 30S ribosomal protein S10

MNQELRIRLESYDHRLLDDTVKTIVDISNSTGSKLRGPIPLPTKKEIFTILRSPHVNKSSREQFERRTHKRLIILENPQPKTMEALKRLSVPFGVEVTFKI*

>UUR10_RS03130 Ureaplasma_urealyticum_serovar_10_str_ATCC_33699_NC_011374 hypothetical protein

MKKNKSKKLIFSLLGISFIGLVPIIAASCSAAAHPQVNVHYVSANVKTNNIGKVKIRAFEGKDQKHEMVFENENLLDDVYYEAGDLTYTSIKDNKRFDKNNKEIDQLGYLIPKKEV*TKAPSNSNKLTPINLSILQNELIRLIGNKLSFDPTSKYANNLEVVDEASKQTVFNQKVYTQGELTKINKTVELINSLVISDGLYTYITSMTDQLMK*LGEHAFNNHSNKDFELFKGLKNEVAKEFLLALVQGVGAGQHTYKLALYNFSFD*EYVDAITGLSITKPSFGTKVSDELLVNPSSKNVFVRLKNIKLQYA*YNSSNKTAGSFMEASSNGSQVNALSSINAALSGVDKQIYAKIHEDKKPEIRNLVYDISLKDIVVNFAPSTMRSANVNENATKLLQENFAKIKNQKTELTETEKKQTTVYNEYYTGTLSKIHAFSVLGKDLKEDLKNEAKANEKEYVEDKKIED*QYYEHDRGLNGVYPYAFLAENNALELKVDDFIKARKFVEAIKASNTSRTILPITDPKNNQVLSKQAYSQLAMIALENKDANIHDLSSFESAKNNLKKYLFDLNDIKSVNVNYSK*NEKNL*

>UUR10_RS03355 Ureaplasma_urealyticum_serovar_10_str_ATCC_33699_NC_011374 deoxyribose-phosphate aldolase

MNKYSIYVDHTLLKPDASLDEIHNLCEEAEENEFYSVCINPCFIKVAKHYLLETPVKICTVVGFPLGANTTETKVFETRNAIALGADEIDMVININQLKSANREYCLQEINEVKKACSDKVLKVIVETALLDQEQKEFAARIILESDADFIKTSTGFAKEGAKLEDIIL*KQILGDAKQIKAAGGIKNLDDFKAFIDAGATRIGTSSAIKILNNQ*

>UUR10_RS02345 Ureaplasma_urealyticum_serovar_10_str_ATCC_33699_NC_011374 urease subunit beta

MSGSSNQFTPGKLVPGAINFAEGEIVMNEGREAKVISIKNTGDRPIQVGSHFHLFETNSALVFFDEKGNEDKERKVAYGRRFDIPSGTAIRFEPGDKKEVSVIDLVGTREV*GVNGLVNGKLKK*

>UUR10_RS02480 Ureaplasma_urealyticum_serovar_10_str_ATCC_33699_NC_011374 hypothetical protein

MIVFFILYGEINLLGFNGIVGANGR*YGEGFKLDEHQQ*VVGVKDFYH*NPIVYDQLKPKPDAFMIEKMQALVTKITNERNHGVEFGTSIISSYPV*VAMFSTLGLAIIVCSILAFSTPKIR*DILTPVVSCWFGMFILIVSGFIPSQIWGYLVRFLILVVAFVLPIFIMAKITNAIMNRSKHFETYAADLYNEFKDSEQYYNEFNKKRLELKKRNKANEKTKYKVEDDYEGK*

>UUR10_RS01010 Ureaplasma_urealyticum_serovar_10_str_ATCC_33699_NC_011374 23S rRNA (adenine(2503)-C(2))-methyltransferase RlmN

MKLNDNSLKQIYSLSLNELKEELLKLNLKPFISKQIYS*IYQKRIFDFDKFSNISKSNQSILKENFDNNLLTINEYQSNSDGSIKFKLLTTINLIINCMIIKFENGFLIKINPFGINDKKEIINLSTNELVLQTLLVQQFLDQHKLGKITNVIVKGSQDSLLNMEAVSNFINIINDENGLNIGKRKIVV*TSGVDVDLIK*GQLQNQIELIISLNASNSQVYKKLMLNKTNQN*SFIKLIEQIKTYTEMTNNRVVLEYLLIDKINDNLDYANELVELLKNILCYVLLIPYNLNHKTSDNLEEFFNILSINKIRISKRVRKSNDLDISFTQLKIKE*

>UUR10_RS01760 Ureaplasma_urealyticum_serovar_10_str_ATCC_33699_NC_011374 hypothetical protein

MCISKVDMAKVKKFFKQYLFAKFQCKN*ELCRELKDYDPKDDQKYLKWEHFVEYVEQVLDALDKTSARIIKEIYIQNKRICELPYSYSTYYAYRKKAIIELLAYLDLKI*

>UUR10_RS02350 Ureaplasma_urealyticum_serovar_10_str_ATCC_33699_NC_011374 urease subunit gamma

MNLSLREIQKLLVTVAADVARRRLARGLKLNYSEAVALITDHVVEGARDGKLVADLMQSAREVLRVDQVMEGVDTMVGIIQVEVTFPDGTKLVSVHDPIYK*

>UUR10_RS03300 Ureaplasma_urealyticum_serovar_10_str_ATCC_33699_NC_011374 30S ribosomal protein S9

MQKSNIVEYKGLGRRKSSIARVKLVPGSGKVFINDRQPENYFPNKLVIQDMMQPLVLTKTAETYDVYVKVIGGGFNGQAGAIRLGITRALIQTREDLKTDLRKAGLVTRDSRVKERKKFGLYGARRAPQFTKR*

>UUR10_RS01740 Ureaplasma_urealyticum_serovar_10_str_ATCC_33699_NC_011374 endopeptidase La

MKKPILISRAIVVLPYETTTIEVGRPKSIQAIDLAKQSSSKEIIVISQKNIDTDEVVNFDELYKVGTLVKIKSIVDNFDDGYSIEVEGIKAVYINSDSEVIDAIEYEYEDVITNPILSTKDEVAINEINSEIFNTINKRTKHKDITFENMHALISLEKEKFAYLAAATYINDYDGEIKEKTIKDRINILLQPNLLLVHETILHFLFDQLVDKRVIEEEVEKMIADKINNNLQKQQREFFLREKLKVVKEQLGELSSREEDADKIRAKIEQLELPPNVRERALAELNRFESAMSSNESSVIKSYLD*LLDLP*TQQGVDNTDLMSVRTHLDDNHYGIEKVKERILEYLALRMRNPNLKGPIICLVGPPGVGKTSLVTSIAQALNKKFVKVSLGGVRDESEIRGHRKTYVGAMPGRIIKGMKKAGVVNPLFLLDEIDKMTSDQRGDPAAAMLEVLDPEQNKNFSDNYIEEEYDLSKVMFMATANYYQQIPYALIDRLEVIELSSYTAIEKREIAKSHLLKRIFTDAKLNENELIFNDDALDFIINHYTKEAGVRELDRQLGHIVRKYIVETYKNKNNKSKPSVEVDEAVIIKYLGKIKFDFNKKEETTIPGIVNGMAYTAAGGDLLPIEVNHSTNGKGGNITITGNLEKTMNESVSVALGFVKANAEKYGIDTKKVSFKEIDIHVHVPSGGIPKDGPSAGIAITTAIISSLSQRPVRTTLSMTGEIMLRGNVGIIGGVKEKVISAYRAGVREIILPIDDERYLEDVPKYILDDIKIHLVKHYDEVYNIVFGTK*

>UUR10_RS01755 Ureaplasma_urealyticum_serovar_10_str_ATCC_33699_NC_011374 Nif3-like dinuclear metal center hexameric protein

MKKTDIKAQDILDFLTKKYDLSRAES*DRNGLFFDEQQIINNIQIALDITDDVVNDAILNNANLIISHHPLFTNQDLNDELDYFVNNDLIEKIKKNKISVIHLHTAFDASPYGMSMQMAKRLGLLNIKQDDQNPYLVVGELKLGVSVDYISRIIKQKFLSPIVKYNNIFRLETNLKKIGIIGGSGYKFADDAFVRHELDMLITSDLKYHN*LDAQAKNQNIIDMNHLSESIFIDVIYDELTKFYGNDENLNKSLSIIKINYI*

>UUR10_RS02365 Ureaplasma_urealyticum_serovar_10_str_ATCC_33699_NC_011374 DNA polymerase IV

MQSSGQKIIMHLDIDAFYATVSELLHPEYKNFPIAVGSLNSRTGIISSPNYLARSYGVKSAMPIFLAKELCPNLIILPCEHDIYQTYSNHFFQIVNKYCNKVEITSIDECFIDATNSIKKYHNNVRLLAAKIQNEVKNKLNLSISVGISFNKTIAKMATELNKPFGISIIDENKITNLIH

ELDISKIPFIGEIKSQELYAINIFKIKELIASNNKQKASLVLGSMYQNLVNDLKGLNEIKTIEDDIYKSISHSKTFNEDLNDFYEISNELNELISNVVNRLKKHNLMTNNISINIKYPNFQTKVKQKRLDYYTDDYQTIFLAIKNLFKKVYKDELVRLIGVSLNKLVPKESVKKQLFLFD

*

>UUR10_RS03420 Ureaplasma_urealyticum_serovar_10_str_ATCC_33699_NC_011374 DNA-directed RNA polymerase subunit delta

MSRQLVDIAYNAIKNNKIYNKKTFSFDNIIDEIVKNSDVNLVEVNSQLGDLYTTLIQDTRFISIGDLE*NLRERLSLDEITKINNAMYEVGLYKDSDREEDEHEMMKNDKLTQSKEQEESDEESSLSDFVGYDDEEEEFKTNSTSDKDEIEDEEELEEEE*

>UUR10_RS01715 Ureaplasma_urealyticum_serovar_10_str_ATCC_33699_NC_011374 hypothetical protein

MNFDINLINENDYVFSGYIVLKKNNEEVIELLTYLGDDPVSDEAKIITLYNNDHIDLKNLNDGDLIIVKASLITNDNSKQLMLKDINKLELSIN*

>UUR10_RS02560 Ureaplasma_urealyticum_serovar_10_str_ATCC_33699_NC_011374 5-methylthioadenosine/S-adenosylhomocysteine nucleosidase

MIGLIVALNSEIKTFFKQIKKKVYQINNIDFYLCTHQNIEFVLVFTDVGKTNASFITALLINNFKPKVILNVGSCGALNDQLQVLDIAIIDQCQYLDVNVSAFGYLKNQIPRLDKFFILDKNYNQQIKNQLIKKKLKC*IANVGSSDTFINRDNILFFYDQQIDLVDMELAAIAHVCTRMLTPLVSIKLVSDHITLPNSNQEQFNKNLSLIDK*FNEHLTSIIEAILEIY*

>UUR10_RS00500 Ureaplasma_urealyticum_serovar_10_str_ATCC_33699_NC_011374 signal recognition particle protein

MFKAMIGNIVSKQMSKKLKNATIAEEDIKELLSEIRITLLDADVNLLVVKKFIKNIKEKTIGLYVEQNQKPADVVLKVIKDELVEILGKENKPVNTAKSQLKIMMVGLQGSGKTTTAGKLANYFRNKYNKKPLLVAADIYRPAAIDQLRTLAKQVRVDF*EEGTQRPDLTVKNALHKADENENNLVIVDTAGRLQTNEELMQELVNVKKTLNPDEVFLVVDAMAGQDIINVATEFNN*LKLTGIIVTKLDSDARAGAVLSLTSLLNVPIKFTGTGEKIGSIDSFYPERMADRILGLGDIMTLAEKAADVIDEKQVRGSMQRMMAGKMDLEDLMRQMSQISKLGSFSGIAKMIPGLNSISENQIDDAENKMKI*TILLSSMTLKERRDPRVFKKEPSRRMRVLKGSGRSPDELNKLLKQ*EVSRDKMAELGKMLQKGKNPFSKSGGIFG*

>UUR10_RS00540 Ureaplasma_urealyticum_serovar_10_str_ATCC_33699_NC_011374 hypothetical protein

MKHKTKKLTLLMAGITLTSLCTVFAITACSKQKSQSNSQTKPNKVIEKVDQAFFDEFKTKIEQKRTLPNIKITSTFKIPFEKNENTLKSPLFKIGDKSVYDLKQNEIPIT*VQDLKDYFLSQKTILTYYALANLKLTSTDAKGAYFLQFIQYVQKMQQKGFLSEDFYQQL*YLVSEISDYDMQNGILRSVYRIANPNLKIKDLYLDGSIYGFKKQKSKYMNFTNLFQ*PSNDEKVCFDIVFKNKNTNQTYTKNIELTYSKEKKYILDSLIDYSEFAPGEYELVSIKKHDDTNAKNLINPNNPNIARIFKVKVFKENEKFKPITLATINQQEADEYDRQNNQIIYKNNSEIGILNDPNKIKELGTFLYFKKSLSEITIDDLDRYFKTYFINPEQDFNNFSRQYKITKIDKEKQTVELSVLHINNKTNETYVSPINFKFHYQHVYDVNKNTDAKLELNFDESKKEQKQKLQALVNKLDFNKLTYNYLSELALVMFEDVDLENTKIHVEMIKNKDRSITLKAYVDKAYKNGKLKISNKADILIKEFKVNYFMTNYFK*

>UUR10_RS01150 Ureaplasma_urealyticum_serovar_10_str_ATCC_33699_NC_011374 50S ribosomal protein L14

MIQHMTRLKVADNTGAKEVGVIKVLGGSKKRYASVGDIVVVSVKKATPAGLIAKGQMAKAVIVRTKKSIRRESGLLIRFDENACVLIKEDKTPRGSRIFGPVAREIRDRGYTKIASLAPEVL*

>UUR10_RS00285 Ureaplasma_urealyticum_serovar_10_str_ATCC_33699_NC_011374 F0F1 ATP synthase subunit beta

MNNGRVIKI*SDIVEVEFKNELPALNHLLTTHDGNTFLLVKRLVDATHARAIVVYASKELAINDVIVNTNKSFMVPVGNDAKNNIYNF*GNPLLKTDKKPQYVEMNSTILNERYVDKSVEIVETGIKAIDFFMPILKGYKLGIFGGAGVGKTVLMKEIIFNLNRHKQANSNIFIGSGERSREAIELYDELNASNLMPNSVMFISKMNEAPGARSSIVPIGITAAEYLRDQNKENVLLFIDNIYRFIQAENEVSTALGKKPSVGGYQSTLESDVTHVQNRLFKNKNGSITSFQTIFLPMDDLSDPSAVAVFNHLDGKLVLSRAQAAKNIFPAFDPLASSTNAIDPKIIGQRHYDAIIETKKVLKAYKDLEDVILILGFDELDAESKIIVKKALQLEMFFTQNFFMTEHFTKAPGQFVPLKETVESVIRILEGKYLKQNPESFAYIGSNKDIPQDN*

>UUR10_RS01105 Ureaplasma_urealyticum_serovar_10_str_ATCC_33699_NC_011374 50S ribosomal protein L4

MAKIKLLSIDGNFAKELEVTSDLFVEVPHKQAMFDSVLAENAAERQGTHSTLTKGEVRGGGKKP*RQKHTGKARTGSTRNPH*TGGGVVFGPKPNRNYNLKVNAKVRLLAFKSALTIKLNEGKMLGLVANSDLETPSTKKMVNFINNANLENQKVLLVIADHFSNIKKSTNNLQKVTTKL*YQVSVRDLMHANVVVVAEEAFTNYARKVSK*

>UUR10_RS01595 Ureaplasma_urealyticum_serovar_10_str_ATCC_33699_NC_011374 translation initiation factor IF-2

MAKKNIKQKKDNRIAIDVKKHIKKVDVGVFGGTFVFTSPLSIAELAPKLNKSTNEIIMRYFKKGVVYNLNTILDEEQIGELCLEYDLDFKIEKNVNTENLLENIAFDDLEADLVARAPIVTIMGHVDHGKTTLLDTIRKSSVTASEAGGITQHIGAYQILKGDKPITFIDTPGHEAFTEMRARGANLTDIVILVVAADDGIKMQTEEAIDHAKAANVPIIVFVNKMDKYEANPDKVLNQLSAKEIVAEELGGDIVFVKGSALKNEGIFELLDSILLIAELNDYKANPNRLAYGTTIEANLDKGHGPLATLLVQNGTLRKGDYLVVGSTYGKIRNMFDEYDNEIEMALPSKPVKVSGFEEVPTAGDKFLALADEKQARAIANDVKQKKIRLERSMLQSSDIRAKIANGELKNINLIIKADVQGSLEALKGIFNSINIEGVTTTLVRSAIGTISESDVRLAQTSDAIIIGFNVRANRIIKDLADSVGVQIMNYDIIYKFKEDLEA*MKGTLDPIIVEEVIGEAKVLKLFKHSQVGTICGCRVINGKIKRNALVRVLRDGIVIYNSKIATLQHNKDSVNEVIADKECGLTIANFNDVKENDIIEVYVKVEKNHDEVK*

>UUR10_RS01025 Ureaplasma_urealyticum_serovar_10_str_ATCC_33699_NC_011374 ribosome small subunit-dependent GTPase A

MRAKITSVIVNNFYVYIYDLKIETKAIPKGIFKHDSHELKPMVGDDIEVELVDGVYLIVKIYDRYNQLIRPKVANVDIVLVVASIVQPDLNTLTLNKYLAFYEARNVKNVAIGLSKYDLASDSLKQKVDQLILDYQRNNYKVFVLTNEHDISLLKKFIKKHTLCLAGNSGVGKSTLINKLDPSIKQRTQEISQFLNRGKHTTTSTKLISFANGFLVDTPGFGNLEVNLTKNEMANAFSDFANYARFCKFSNCLHIDEPHCAIKKAVNDDQIVN*RYDDYLKIMKKLPNDVLEIKTRNQNKK*

>UUR10_RS01285 Ureaplasma_urealyticum_serovar_10_str_ATCC_33699_NC_011374 valine--tRNA ligase

VKKKLNKNYLFKEVESNKLLF*QENNLFKAQANSTKPPFAIVLPPPNVTGHLHIGHAYDFTLPDILMRYKKLQGYDAFIVPGTDHAGIATQTKFEKILKTNEQVDRFVLGRKAFLEKLKI*KDEQTYYIHKQ*NALGLGLDYNNYLFTLDEPVVQTVREVFVKMFNENIIYRAKKLVN*DIQLKTAISNIEVIHKEIEQKLYYIKYSSEDQKDFVIVATSRPETMFGDKHLIMNPNDQRYVHLHNKIFINPINNAKMSVILDDYIDIEFGTGVMKCTPAHDFNDYELAKKHNLELINIMNEDGTLNEKCAEFKGLDRLQARALIVDKLQKSNHLVKIENYQSNVGFSERTNEIVEPYLSYQ*FIKMDNLVKNTIKMQNDFNDKVDFYPNRFNKTLLT*LENTED*CISRQL**GHQIPV*YHKKTNEIYCNTTPPKDLEN*IQDEDVLDT*FSSGM*PLLTTK*NSNDQFFKRYFPTALMVTGMDILFF*VSRMMNFSQYLVQKRPFKDVLIHGLIRDAQGKKMSKSLGNGIDPFDIINEYGLDTMRLFFASSTTVGEDLNFSTERLGAN*NYLNKI*NIAKYIENLDEINESFSIQDVHEFCDVNKWIIAELSKLSVEMNKNMDKYNLVVATKDLYDFI*NTFASNYLEYTKVLLQDTTFKNETIKTIRYVFNQILIMLHPFAPNISEEI*LNLNQTNESILLQKYPMVNFEFESIIINKIAKIILEIRKLRLQENINNKTNLCFELVSANDEFYNSNIKLINLLLVLVNAKVSEIKKSSVNSCTYELVIDDFILKT*YEKSIDYDTQIKKVSEQLKYLENEIKRATNLLNNQGFVNKAPTELIAKEKDKLNNLEKEQANLLKIFADLKQKVN*

>UUR10_RS00515 Ureaplasma_urealyticum_serovar_10_str_ATCC_33699_NC_011374 hypothetical protein

MIKK*NSKRK*SLVCASLVIGSASVVTATACANTNNKVVNAGFIYNGSTTNDAITGFDINDYIIKVPSSDVNLNQQILRNNNYEQKPIL*EDYLKQYDSVKKQHDQQTKSFYEYV*INKASKYKNLNKAIDINDPYFADWFLKLTPNTLKNDLYDFINDIRKDNMKPSLVFSSSEPQVEV*KDLGNNQKQKIAPNQVIFEPLTQEDTKNNVTLFKKNKHLRVNISF*YALTNQNSANVLINDPFYKKPSGIASNTSEKYFINIQNSPISLSFSHTDRLEHDIYSAQTTSIKNTYKMRYYFDNIRVEKQVTKVKSNAKRGDQNAEENLAKEFYFANDNNEKFSFGTRSYTLNNEITQASYEQDIFSLDQKIKNELKKLNQQQILKDIEYAFASGYDVAIDSISSIFNILKGVANDLDLKELFLQSTQDFKNLTYNITQHNNLTNLIALITSNQSLGVVIDGFKPILKEIIYKNASIDQSVKNTI*SKIESMDFKNNLIAEIGQIKTLLDSISLAEIKTYKPIINKLLELISLIEARSKKDPKNYGFIDGLDELFSFFLNLKQDDLPSNINFKISDQNYGLYDLIVEVKKIVDNLVPHENQYDPKNQQIISTNYFLSKIKVLDLISLNKTNELDYRKGVESIFSLLKKFQVAIPEIVYKIIDELLIQNTN*NKENIKKLLDAILYPKITSNDASINDLKSYFKYGISKPEITSKELEYDQNNLLIKKLNIKYRYKVLANAEFDIKPLFDLLPQKAPSFINIGSL*DQIKKEFPYKVVLAKDDYVDHTISINEPQELTPLVFQDRSDHDKYKIGYSFYPTHTVQTHMPNSMKAIIEEISSRNDRLLPNKYIAQLLS*LFYKKWVFTNPLAIYETFEGDKKVANDKNVKYLIKDNLNKYLKHYDTNTYYEGFDFKHFSNNLENKVNNQTIRNLILAKIKTIKTDGQELYQDKLGRKVIATSAYDQINLTLQELIDYKLLEFGKKVDLKKDVYLSAVAFNFGLNTNDSDQTNALNNNDEIKYNLNILKKVVITLHFTKPTLDLSDPNHPKLVNSYTFVV*

>UUR10_RS01320 Ureaplasma_urealyticum_serovar_10_str_ATCC_33699_NC_011374 hypothetical protein

MEQIKNLINLQIKLKPIQKTFYDI*SKTFINGIDNNGEIHQQNKQIILEIYTTLKTFLNQNQNVISKLPLNEVKKIANDILKKEINLEQPLVDYYYQSSSYFILIPYLIQILYQSYDANKPAYKAMAKFIIKNNLGLFKE*DLIERQTLEIVKLKTNLIEDQRKVINLFSCEQREAQHNR

FVKLFNNFILVY*TKEEVKYIEMIRFLMYFA*IPIIFIILLVLILGLYFGLTNSSSSSTTQLLLNLINLY*

>UUR10_RS01515 Ureaplasma_urealyticum_serovar_10_str_ATCC_33699_NC_011374 tRNA1(Val) (adenine(37)-N6)-methyltransferase

MQKKYIKNTLGFDTNLFVYQDKQMFNYSVDTVLLANFISLSSKTKKVLEIGTNNAALSIFLASRKEDMNIDAIEIQSEAIDLALLNVKENHLEKQINIIHADFNEY*KTFDKIENNKYDAIICNPPFYKQDKIIPSTKKPLKTLALYEIALNFEQIMQGCAKIIKQKANLAMVIPTTRLVDLLEMMRKYQFEPKRIKMIYPRIYEQSNLVLVEARYKTG*GTHFEPNLYLHYEDKQNHEYTKEVLK*YKPIKFKKSNEGEAK*

>UUR10_RS01795 Ureaplasma_urealyticum_serovar_10_str_ATCC_33699_NC_011374 iron chelate uptake ABC transporter family permease subunit

MQTKQVNINKKDLSIYKKIIEAKKFVSTQIRRPYMITIIVLTFIILAVGMFFMNVFYERSTYFDANYNTHVTYFRT*AKVVADDKSILLYWYPPIVKLFVAISMPVAGYAIQITTQNRLSSPSTLGYIPVSILAYVAMLMIDQGKS*LVYVFGFIFSSFIILVNYILQRQKSSNRSFKPVLIGFAISATITAVGLVIAVSRPNILNRVTI*TGELPNVYE*LKLYISMPLILICLFAFLVLSPKLKIMQRDFALAKSLGIKVNLIF*VVTVLTAIVTIATVNITSPMILLGLIIPNIVRATFNKHEPLFVFFVSIVFSLALLEVSLFLSLNYRFGPNFLMAIVSAFVLVFIMRKHG*

>UUR10_RS02135 Ureaplasma_urealyticum_serovar_10_str_ATCC_33699_NC_011374 holo-ACP synthase

MKLVHGIDIIE*NREELNNPSFAKRILVDDELKYYLQLNSLKEKNRYLASIFASKEAVMKAFKLKYGYNDILILKTKNERQVYLNKILIKELVLSISYTENYVVASVVGLINTVESNS*

>UUR10_RS03365 Ureaplasma_urealyticum_serovar_10_str_ATCC_33699_NC_011374 HPr family phosphocarrier protein

MISREFTIVAELSTFIKAITYFVNTASDFSSSITIKANGRQADAKSIINIMALGIKQGTKIELSAVGNDANEAINKLEEILIEQKLI*

>UUR10_RS00440 Ureaplasma_urealyticum_serovar_10_str_ATCC_33699_NC_011374 recombinase RecA

MKENDKLEKLDPIATLEAKFAKSTYFIADEIKNEKVSAISTGSIHIDQITGINGIPVGKITEIYGNESSGKTTIALQTIAECQKKGGTAVLLDLEGSFDLNYAKSLKVDLTKLIITQPQTGEQAFDMIETLIKTNSIDLIVVDSVAAMIPESEYQANMSEALMGAHARLMSKGLRKIQPLMNKSQTAIIFINQLREKINTFFGNPEMTTGGKALKFYASLRIETRKADLIKEGINKIGIKTKVTTVKNKLAPPLQTCFIDVFFGHGFDYDNEIIDFAIQYGILKKNGSWFYFDDNKIGQGREQLKNTLLKNNELFTQVSEKTLAFVNNEKINQ*

>UUR10_RS00830 Ureaplasma_urealyticum_serovar_10_str_ATCC_33699_NC_011374 23S rRNA (pseudouridine(1915)-N(3))-methyltransferase RlmH

MIIKIISVGKLKQTGFVNLVNDYLKRINYYLKCQEIVVNDEPEPTQISTKLLEQIKDKEANRILKNINQNDFVIALIIEGKIISSEMLAENLQN*LNASYPNICFVIGGSNGLHEKIYERANYHLSLSKMTFAHGLAKVMVCEQIYRALSILNNGKYHK*

>UUR10_RS01195 Ureaplasma_urealyticum_serovar_10_str_ATCC_33699_NC_011374 preprotein translocase subunit SecY

MTNKQKKKNAFRQLLMIFKNKKVLVALIVTLSILILFRIGSVIPMPYIKLNGNFGNQGSFFSIINLLGGGGLSQFSLFAIGIGPYITAQIIMQLLSSELVPPLAKLSKSGERGRKKIEVITRIITLPLAVMQAVIIINLMTRANGFISIVPNAPFAIGSPLFYVTYIFLMVGGTYISLFLADLISKKGVGNGITLLILTGIVASLFNHFIAIFSNLGSLTSSKVSQIIGFILYILFYIMILIGVVFVNNSTRKIPIQQTGQALILDHEKLPFLPIKIMTAGVMPVIFASSVLAIPAQVAEFLDKQSMGYYVIHNYFIVDS*TGLAIYVVLILLFTFFFSYVQLNPPKMAEDIKKAGRFIPGVQVGMDTEKHITKVIYRVN*IGAPILAFLACLPHLVALVAKTINHGIPVIQPSTIFGGTSIIIMVTATLEL*NAIKSTSTSTSYAYQRKELETAITISVESDKSSKSQI**

>UUR10_RS00890 Ureaplasma_urealyticum_serovar_10_str_ATCC_33699_NC_011374 ribose-phosphate pyrophosphokinase

MPKNHDILLFSLSNSRQLANKIANLLKIELSPIRIDKFADGEFIVAPQVPVRGRRVIIIQSTSKPVNDSLMELLIAIDSIKRASAKAISVVIPYYGYARQDRKAKPREPITARLVAKMIESAGATSVLT*DIHSLQTQGFFDIPFDSLEAV*VLMKHYFDAYKDSSNITIVSPDYGGVKRAREISIATGATLAIVDKRRSGKNQVEINNVLGDVQGRDCVIVDDMIDTGGTILGAAKIVREKGAKSITIIATHGLFNNNARERFEQAIKDKIINKVCIADTIENEPFEGLEIVSIAPAIAKCIEIYSKGAGSMSFVHDENSKFLFTKKNNK*

>UUR10_RS01140 Ureaplasma_urealyticum_serovar_10_str_ATCC_33699_NC_011374 50S ribosomal protein L29

MSSIAQDLRKKDSLELEKIVIELKAKLLELRFAAANGEAEKLHTAKEIRKTIARALTILNERELAEKLNNKEANK*

>UUR10_RS00430 Ureaplasma_urealyticum_serovar_10_str_ATCC_33699_NC_011374 DNA topoisomerase (ATP-hydrolyzing) subunit A

MALKKPKKSRLTTEEIKQQLEGSTIKEQSITKEVETSFLDYSMSVIVARALPDVRDGFKPVHRRALFAAFENGMTHDKPYKKSAR*VGDVIGKYHPHGDQAVYQTIVRMAQEFSMRYLLVDGHGNFGSIDGDSAAAMRYTEARLSKISYELLKYIDKETVDFVPNYDASEQEPSVLPSGFPNLLTNGTTGIAVGMATNIPPHNLTEVCQAIKAYAKNHDISIPEIMEHLKGPDFPTGAEIYGDSGIINYFNTGRGSVTIRSKYEIEDIGQGRVAIVVTEIPYMVNKVNLIEKIVELVTNKQIEGISDLRDESSRDGIRIVIEVKRDVIPEVLLNKLFKTTALQTNFSVNNLALVNGVPMVLNIKEMIKYYFEHQIEVLVRRTKFDLRKAKERIHIVEGLVIAVNNIDEVIKIIKASGDDDIASKALIARFGLTELQTKAILEMRLRALTGLNIDKLKKEYEDLLLIIEDLEDILENYDRQVNIICENLDYLIEKFGDERRTEIMYGVSSHIDDEDLIPVEDIVVTMSKRGYFKRLPIDTYKNQRRGGVGVQGLKTYEDDDVEKILVANTHTDLLFFSDLGRVYRLRGHEVPLGSRQSKGIPAINFLPIEKSESILTILPIDNYEQGSLFFTTSKGIIKRANLSDFESIRANGKIAITLKEGDKLFSVMQTLGNDEVFIGASNGNVIRFNENDAREMGRIATGVKGINLEDDEYVVGTGLSSHGEYVLAVGSKGLGKLTDINDYRLTKRGAKGVNTLKVNDRTGNLVSIKVVNRDEEALIITTSGKVIRLSIQDISVIGRNTSGVKLISLENKEEVKSIAIFKKEEIDDNDDEQKTSHGNEHNLE*

>UUR10_RS01230 Ureaplasma_urealyticum_serovar_10_str_ATCC_33699_NC_011374 DNA-directed RNA polymerase subunit alpha

MRKFLKYQLDVPSINSEDKNRTVVKIAPLEIGFGDTLGNALRRICLSSIPGASMFAVKFGGYSHEFQPYEGVKEDITHIILNLKNLAIKIDELIYSEDYFNNLLIDK*PKMKINFKGPGVITAKDIVCPVGFEIVNQDLYIAEVTKPIDVEIEIFAKTGRGRVDFNTNKDFVSTLHIIATDSNYSPVLHYAYNVEMIKDSKSSMSEILTIDIATNGTISGSEAIAIAAKIMQAHLEPIMNIDKTINEMIIMREREEEEKRQNASISIDDLDLTVRAYNALKQSGINTTAELIELTKSQLEKIKNLGRKSVTEIIQKLTERSLELKKD*

>UUR10_RS03240 Ureaplasma_urealyticum_serovar_10_str_ATCC_33699_NC_011374 tRNA (guanosine(37)-N1)-methyltransferase TrmD

MKISILSLFPELYETWINHSIISNAIKNNQVTIEIINFRLYTNDKHKKVDDYQYGGGAGMVLMIEPIVSAIRAIRTPNSYVILTTPKGQVFNQELANEFVSKYDHIIIIAGHYEGFDERINYYVDAQYSIGDFVLTGGELPSMVISDAVIRLLDGVISSSSLESESFNNYLLDYPVYTRPVVFEGHQVPDVLLSGHHKNIADFRKQQQEMITKKNRPDLYQKYLNSKK*

>UUR10_RS01235 Ureaplasma_urealyticum_serovar_10_str_ATCC_33699_NC_011374 50S ribosomal protein L17

MSYINKPGKTRA*RKMVSRQQVSDVISHGSIVTTKTKAKESQRHVDHLITLAKKNTLASRRAAAAILLGTNQHSADDLLRKLFNELGPKYANRAGGYTRVIKLGNRPGDNTEEAVLQLV*

>UUR10_RS01445 Ureaplasma_urealyticum_serovar_10_str_ATCC_33699_NC_011374 elongation factor P

MATIIQAKDLRAGHTFLYKGSIYQVIENSFNKTAMREGIVKCKVKNLRTGAITVEVLTGEKVEQAIIEKSKMTFSYDDGSGYVFMDNETYEQISIPYNQLS*EKNFIEEGTEVSVMRYDGELMGVSLPDQLVVTIVEAEEAVQGNSVQNATKRA*LASK*EFQVPQFIKSGEKVIINPSNGQYVGRAK*

>UUR10_RS00250 Ureaplasma_urealyticum_serovar_10_str_ATCC_33699_NC_011374 ATP-binding protein

MKIKKLIASVSIITPIVFVSALAASCVNNKHEDKNIVHNSSDHGNNKKNNFTNDLNQNLTTHEFKSNLISTSQLDNIAKLINFTYENKANTYLKNILINQLKHSPIQNQDFKLEIIGLYPKQNSLQDLIIYYKLTNQKTKEIKGYYFELNGFKKPDNTIFSNQLTPELKKIIDTIQFKKTFDLAINNQQVLNYENVLPSQIKNQLLMGLKVIQKEYTDKIKLSVLDVLFINEGGQINANKLGAFSLLLEVLDLKSKKTFQILIPVDKFKTNPYGADEYDLLPTQVNNGFAPTSLAQINQYNNADQQTRYLYDNENYLKSLKAYQRNVN*YQIRQDLINNKQKINEFDQKAPQVFQDSYESAARKGFTLPVYDEDGKYQGLSFNETEIGKSVS*VDAIGKDQWKINGLARTLPNDMYKQIALQTFGIQIQTPNGKPRESDIVAGTM*IMDYQKRNDNKYPTKWYFGTNLHVAEALKSTTTVFGINKIMPTVKTKTTLGLANADDNIYRFSLVSKDNQPNMDKPISNGIKTIYDGRDFLKLNPSDLLTSKLKNKYHNLQEFVDFAVFEIDFEKIKLGSVTKNFYSGSD*SVDKYNNLDPSELAKLITNDYAQKPNEQIKFLSKSYLNDYARINVPLDSKYNQAFDTNKYDELYAVGWPSSASDYFLDPIKDQKQFESRRESYSL*INSNYQFYNKLNHNPPLFPLTQINRGDFLSYNIGYRSFTNKPGLLDAFIASPITGKTIHKSTDNQNYIGFGLNYLPMHYSPIGGSSGTSLRNQKNELVGIWHVGNGFAQTGLAVAFRSEGYDYHGLYGSYNLPQYDLIYGGGKNQKTSYRQAMMALYKNKNIATALFPNGFGEDQIPSAFKFKN*

>UUR10_RS00820 Ureaplasma_urealyticum_serovar_10_str_ATCC_33699_NC_011374 DUF4234 domain-containing protein

MKNLNTKQKSLIIFLSIITLGIFAIYFFSKAKKTSQIKNTHLTTSSKIPFSLTAFYDCVGSKDNLANVDATINTLKIELKEASLLNNEELKHLGAKGIMRNQTKISIIFGDFCLELKELIKKDLLS*

>UUR10_RS01155 Ureaplasma_urealyticum_serovar_10_str_ATCC_33699_NC_011374 50S ribosomal protein L24

MNRIKKGDTVVVISGKNKNKSGVVIQVNPKEQTALVEGVNKIKRHQKKDQTHEQSGIIEKEAPIRLCKLALVDPKGKDKGKATKVKYLLKDNKKVRVARKSGSELDANKK*

>UUR10_RS02205 Ureaplasma_urealyticum_serovar_10_str_ATCC_33699_NC_011374 nucleotide exchange factor GrpE

MSKNNENIKHQNEGKLHDQVDKKETKNHAKQEFKYKELYEHELKKNKELQNVNELLINKNQQLEIQINQLNQDFVKQLETKTKQAQEILEQKVNELEARHETKVNDAVFKIFKFKMEPLLDAINHFTKIVNQNYDDPKIQAFIEGFKMFSQNMIDGLENLKITKISPQINDMLNDDTMEVFEVVQNTNKPSMHVTEVISDGFKYNDKVIKFAVVKVAK*

>UUR10_RS01730 Ureaplasma_urealyticum_serovar_10_str_ATCC_33699_NC_011374 segregation/condensation protein B

MSNDKKNKKNSLNDELDLAKYTLHNVSVKNDEDENDLELD*ADIDDEIIDNNQQVYQKKRNNHKTRLFLEKDNEINNHGQVGLYKETINQPKALSGKEFLEFVNSKLIQKKVEKSNYDGIDNEFLENRLVKNNNKILREKLKPQNDFNKIFKPKGLDDYIDEQQAEAQKVEFKNDFNNSTKKPKSIKQNSVSFRNSFNLNDLDQKQIVNIKSIIDSTLFLAGEEGVSLQDLKRTTGLGESSQIKLILNELQKDYDADDSGLILVQFGDKFKLLTQSKNKDALSKFVTTSFKTPLSQRNLETLAIIAYNQPTTRAKIQAIRDRDPKPSIDALLKLNLIVEAGRQDTPGHPILYTVSQKFYDLFGIRNLTELPRLNKEIKEFNPIDETTN*

>UUR10_RS02965 Ureaplasma_urealyticum_serovar_10_str_ATCC_33699_NC_011374 DUF2188 domain-containing protein

MATDDKVVYYLSPNEDKG*KIFKKGGERATKLFSTKKEALEYIKTLGRNQNAVVYIQTKDGKFQDVRNYREK*

>UUR10_RS01065 Ureaplasma_urealyticum_serovar_10_str_ATCC_33699_NC_011374 hypothetical protein

MKK*QKILTIALPTSLLVAIPIVAASCSTKSEAQKEFEKTYKEYLS*LDKLASKMPVLKEAFSTLKEEINKQINKSEKLSDEAYKALTASLKVSIDGMKKTLGQN*

>UUR10_RS01170 Ureaplasma_urealyticum_serovar_10_str_ATCC_33699_NC_011374 30S ribosomal protein S8

MYLDPIAELITKINNGRKAHKAEVSFATSKLKTAILELLVKEGYIKSYDIRPTENNKSETVVKLKYKNQTTSSINGFKQISKPGLRIYSTHLNLPKVLNGLGIAIITTSKGVMSDKQARKENVGGEVIAYV**

>UUR10_RS01310 Ureaplasma_urealyticum_serovar_10_str_ATCC_33699_NC_011374 hypothetical protein

MFNQILKRRKRHLNFEIFIFFFILFLIAFLAL*IISGNIQTITDFIKTKIYDASPQLKYLTSVKIEPFNNLEEVLDFIIKNKTLIKELQNQPEILDAIKNNPQILKTISDNQEILKQISEYKAFIEVAGKQPEILKFAQNNKELIKQIQAKPELLDLIKNHSNLIDHLKANEKYMDIIKQNPQMLKDLYHIDDYLFNIIKTSLENEKENNILALAVKNATLINSFTKNLNLQEIKHAIEFYKTVDLNKLQVDDKLIDIIKNNYEIFEVFLKNPDYIKNLNDNQNIINAIQQNPDLIDNFKNFDFDVLVQKKDLVRALIKIIEYKPHPQIQKFNEKVFLKFFKDNRELANILNIVSLIGSALFIILYMIFMFASFIAHVSMLKQIKNFNKIFKEAKLKKAYLVFSLLLQFFFFVVLFVINFIVLIILVVDYNKLKAIVQDNDDGEREEAY*

>UUR10_RS02885 Ureaplasma_urealyticum_serovar_10_str_ATCC_33699_NC_011374 ribonuclease J

MAKINFLSLGGQDERGKSCFVLEVNDDIFIFNAGAKIPTSDVFGVNMIVCDYSYLEKNAKRVKGIFIGTPTFNNVMGIKLLLAQVGYKIPIYTSPIGAIVVKKIFEQKVNNKKIEPNIIELDPISDKKIGSIYVTAFKVSNSMPHSYGFVLKTSDGAIVYIDEFIISNDKNKTFDSQINALNNITKNNTLALIVGMGQAGNPCFTAPNHKNKGFYESILQNTKNRLIVGCYSNDAYSIFTLATIAKQQNRPFIVYSNNFINTFVGVLKLKLFNSKNLISLPVSEINNSKNAIIVVIENQDTLFPRLNKILHNEDKHITLTSEDQLVLGVVITPGFEMLAAQLSDEVGRLDIPYKALPKTVLPMTQSDEDLKHLINFLQPKYLIPINGLYKTEVKFTSTVTTS*IKSDQIISISNGELFSIEDKVLNPKPQVIELEDKYISSFDALDVGANILFERAQMGENGVINLIVIFDKQFQKLFNYVEFDYCGVVNDDPQVRAQVKEIEETFKKRMGECLVYDDRKRLVLKDTKASLKRLLTKLFEKKFNKRPLVLPTVVDCYKTK*

>UUR10_RS03455 Ureaplasma_urealyticum_serovar_10_str_ATCC_33699_NC_011374 50S ribosomal protein L34

MKRTFQPNNRKRAKVHGFRARMKTKNGRNVLARRRLKGRHSLTVSGEK*

>UUR10_RS02140 Ureaplasma_urealyticum_serovar_10_str_ATCC_33699_NC_011374 DivIVA domain-containing protein

MININKINEYKNKKFIANKVGYDPESVDVFLDELINELETFVDEHKNLLTKIQELSEYKIKFEKEQEYTTSLNNFIDLLEKVVDKKSTFDV*EKRPVRK*

>UUR10_RS02950 Ureaplasma_urealyticum_serovar_10_str_ATCC_33699_NC_011374 iron ABC transporter permease

MNKIKNNEKHLFLRFKKMHNLKTFSKKGHRLKTKQIVFIVFLILFCSSFFLIDLFFTGNHLNDVKRMFENSSLSNSTYIFVPVANIIAGFSLGVGSISIQITSKNILSGPSTLGFTPMTILASTISFIITSSGVFSTVLVYCLGLIFSFVVIGVNFILVRSNFLENNFKPILVAFGIGALVTGINIVLIATHDNLKIVG**RFIAINNTLINNYRMIVSSVLMVISTIILLFLSPYLNIIKKDYLLAKSLGIKVNLIY*LVAICVVIITISSSILLGIIALLGVIVGIITQTIFKKTHVLLLMVLAGLFGSGILSFSSYINEYIPSAREMIICIFAVPVFAYILSKRKGFVK*

>UUR10_RS03295 Ureaplasma_urealyticum_serovar_10_str_ATCC_33699_NC_011374 hypothetical protein

MYTKLT*NNYEIVEQIKENINPNQQLSALTLLL*SYYGYDIYFEYLKDENIVLFYARANEQLKLIENDNIANQFIDKFFIIFIYFNDEKYN*TTLIPIALKALKENFKNQSLLVASGIVYEDYLNDLLKFNHELVYT*YSNFIYETESLKYFRGKALQKKRNNLNFFIKNFKEDYEIIKYDSQIHLNKVCVFLKN*DIKNFINSKSMLVNSNCDLLSNTQNNPYFAGSILVKKSTNEIVGFTLVYIRPNIAEIIIEQTDRQIRGMYQYLLSQNLIINNVNNLLIDRQDGA*SDNISASKLSYQPKIITKRANILIKE*

>UUR10_RS00230 Ureaplasma_urealyticum_serovar_10_str_ATCC_33699_NC_011374 membrane protein

MKKLKLITLSLSPIPIVAIMATACSTTKKEKEVDHKQDELTTINSNLQLEYPQNKDIETSAAIKEEIKPLNLPSGVVFSIKEISPKADDESTLIVKYTLKKGNITKEFKKEINGFKKIRKDQNISNLKDMHDDFKTIFNNIKLKDTFDFKLKGLFNSNFGEYDKLLPSQIINNFAQGVETRINTNQDRLGIEVIDVSYPNRNFGSANREGNLKMSLLVTDKKTKQVFIKSIIAYGFKTNAMGLDENGSIPGGGADLVKPKVDENNYFSKTQLERYEIDNQAYLNGLKGQHMGKT*QQVRPELGDNEQKIKEFDEKAKGVSQDAYASAAYKGFTLPVYDKNGDFKGLSINESSYGQARS*VDTRGRDE*KTTGLPRTLPNEKYRDEALQTLGISSLTLKDGKKDT*DKSSGTT*ILDYQKTNDNKYPTK*YFATNLHVADTITDKTTSIDLMKLMDSVKTKTTLRLSNLDENIYRFGFASKNNEFLLNHGLKKIYDGRDFLKTKPTEYLTEQQKEKYKDAGSFVDFAVFELDFEQLKLLSV*NNQLNSNGGVITKYDHSSAQDLAKIITSDYANHSDKQIKFLPKSYLNDYSKIDVPLRSTSYKFEGKDELFALG*PNSTRDGFFERYVDDDQTKYRTTDNFSL*TNSDYRFFGKLTEQEGGQPAFPTERTERGNYLSYAIGYRSFIDKPGVLDAFISAPHTGNDLYKSLDGKKYINMGLEYMPRHYAPAGGASGSSVRNQNNEIVGIYHVSNEFASTGLATAFRSEGYDYKGLYGDKYKELPQYDLIYGGGKDQATGQSYREKMEEIYKNNNNIKTALFPNGFDQYDEKFKFNNNQSK*

>UUR10_RS01120 Ureaplasma_urealyticum_serovar_10_str_ATCC_33699_NC_011374 30S ribosomal protein S19

MSRSLKKGAYADPSLLKKVEAANASVSKKPIKT*SRRSQIFPNFVGLTFEVHNGKTFLKVYVTEDMIGHKLGEFAPTRNFKNHTEAKR*

>UUR10_RS02910 Ureaplasma_urealyticum_serovar_10_str_ATCC_33699_NC_011374 UMP kinase

MSKQRIVIKISGACLRQDDNSIIDVNKINDLAKQIKEISKKYIVSIVLGGGNI*RGHIAKELGMNRNLADNMGMMATIINGLALENALNNYNVDAIVLSAIKCDKLVYESSANNIKKAIEKEQVMIFVGGTGFPYFTTDSCAAIKAAETESSIILMGKNGVDGVYDSDPKTNPNAQFYQHITFNMALTKNLKVMDATALALCQENDINLLVFNIDKPNAIVDVLEKKIKHTIVSK*

>UUR10_RS00095 Ureaplasma_urealyticum_serovar_10_str_ATCC_33699_NC_011374 DNA polymerase III subunit delta

MKYSFANLLIQSPKTSLTLGVEQIMLAFINEKNHEQQAYYINKVKNNQYFDLKIYDSLSMKKSDVIDLQNAFLYDGIEDINLKFYLIKNIDLASKYVLNALLKFIEEPPKNTIAIFSTKNLNQVLKTIKSRCQLFYLPANYDLYHQLIKQINQPISATECDLIFDDLDELKTLLENNEINEVLAYHAKLNDIKSFETLNDLKETFKNLSILQIHYLLKLIFIKINNINSKQAILDLMRANLKININKNSLFTIIYTIIIENRGD*

>UUR10_RS01615 Ureaplasma_urealyticum_serovar_10_str_ATCC_33699_NC_011374 M48 family metallopeptidase

MLNFVKDLNFEINQKKIIVKVCLQPKIKYIKIKIINHEIVCLINNLTLVDIAQDFVINNKLKILKLYENDLKRIKYDEHYLN*ITLLGIKFSTIKLVNDTFHCEFNLQKQIIYIYDQSQNLANSKLIYQKILKYLAEIIFPQIIKNAENITNLAVKEYEFGFYKSR*GVYDKLKHIIKLSYFLVHYDQEIIQYVVIHELTHIKYQHHQNSF*DFVLKYCKNAKIYNKQLKS*

>UUR10_RS02715 Ureaplasma_urealyticum_serovar_10_str_ATCC_33699_NC_011374 membrane protein

MSFKTKTKFKQK*KSVLAFLGIGALTVGTIVGTGVGAANYAYKNKKTGDFGDKISARTQVLLNDYQTEEEQIDLLKTTANLNQKHLQALGVNNVSAKYGIYQRINKNTKK*EKFGEIVYEFYPTNSKLDIVNFLTKTKPYEQISSKIQLISLFTTANRLELQNISSLLSPKPELSQNKEFDDTN*LTLNSDAKKIEIKTTNEGQQVEVSLPKTSADDQKFDLNFFAKEFDANFNYSASSGKTRAQTVAAFEKTKQNRKAPVNS*LL*VNRDGLIARFNMLLTLAHAQKQKYVDAKS*DYVDATYKNLNVNGEEKAFID*LATQDFDKYFIHNSSIIKPINVQVDQLLNIIRAFYQSSSHKVKTKDANNKDVAQTFKDNEMFYS*NINKLSLINGFVYAIDYNNFFNYFEAPKKDDKLTLAQQSIVKYTSNKLVLNQKNTSRAFINTVYNLKQYYLPTYFAQAIYSENDIKKDYAKTFYSLLNNFSTKLPNLKQGAIKMLDPIDAILIGIAVLILIVAIIISVLYKVPGLIHSLLMAFNFVISLLLIKASNLGFSTETYGALIISVC*PLLNLVNFNNHIKRLVEQKYSYKNALRISLNKTIINQGMFYLLMIFISLVFMYFGKNNISIFGFNLILITFSCLLISYILFIAMMYLL*LIAHHVPKIHL*RQYLAATNAISQNRFNEEFD*NDQQKYQQFIFKAMNQKVYKLSFLIFVFVIGLAGLFVLGFVVPNMSFSFGSVYELTMKNDLKNFNEHEFSSFLDYEINNGIVSMYFDAKNPSALNEIASISLNNRFSDAIVYTSSLYNLINNITNSIAAYFIIFAIIVI*SSI*LKPQSTIPLIINLICTTLVSFGIAGLFRLFNNQASIIAINTSFVLIVSFSLHICLNLKQTLNLAKVLTKKQLQLQIQDSLIKYFNTYNIIYIVMLFTLLWLMIFAPLALISFNAIILFSLMFTHYLAIIIINYL*MLTMILYERLLAKTLAKSDNANNVYDKFDEQEIVNINKF*

>UUR10_RS00765 Ureaplasma_urealyticum_serovar_10_str_ATCC_33699_NC_011374 putative DNA-binding protein

MLNKNKR*YLIALYDIYQGLLTTKQCEYFNLHYFKDLSFSEIAELKEVSKSAISDCLNKVCDQLLKYEQALLIYEKNKKRNDLYTLINDSELVKKLKDI*

>UUR10_RS03225 Ureaplasma_urealyticum_serovar_10_str_ATCC_33699_NC_011374 hypothetical protein

MINLNNNQNNFFNHTIGMFHDKRIFCSRFIIICISIIYKMYINTIKINKR*SIINKMYF*

>UUR10_RS03255 Ureaplasma_urealyticum_serovar_10_str_ATCC_33699_NC_011374 ribonuclease J

MENAKKSPTYVYALGGLEEIGKNTYVVEHEDEIILIDAGIKFANASLPGFDGTVANFEYLIKNNHKIHSLVVTHGHEDHIGGIPHILRHVNIKTIYAPTLAAKLIERRLSEYKDIKPPRIIIFEDESMYKTKHFEVDFYRVCHSIPDSFGICVKTPNGYIVTTGDFRFDFATAGDETNLAKISQIANRGISVLMCESTSAEIPGFSESERYVIDNIRDYMVNIKGRTFISTFASNLGRVEEIIAIAVGLNKKICIIGKSMEANIKTSRKLGYLNVPESSFITHKELPFYKDHEIVVILTGSQGEKMAALNVMANNNHSKITLKPSDTIILSSNPIPGNYAQVEAMVNKLYKLGLTVYENSPNKKIHASGHATRSEHQLMIKAINPSYLFPIHGEYKMFRALKQNAVDQGFDKDHVIIATNGQKLQLLDGVLSHSNIHVDAEPKFINGYEISSKISKLLSERVVLSSDGILNLVLNADFKKAKLNSAVSISTRGCFFAKESTNLINKISNVAKSSLEDALAKKEFDEKKLKEIVSGSVKSIV*K*RKKNPIINITIINNDLVEQFRKDNNYVEFIKQTEVEEIEQEVDIDDLISNGL*

>UUR10_RS02315 Ureaplasma_urealyticum_serovar_10_str_ATCC_33699_NC_011374 hypothetical protein

MVKSQKVIDVLNAHYNLNLELGSVYAQYAHIADDQFSMPFLAKFINDLSNDKLGVHKDLISEYARKIEIPLHTKFSVDVSFKPTDPKELVKHILETEQKVRKHVANMAKVCLEEGDFETFSFVK*FVDDGIKDFDDVRTIHDFFENGNNNLQVEYAIRKYLKQMKLEEEK*

>UUR10_RS02915 Ureaplasma_urealyticum_serovar_10_str_ATCC_33699_NC_011374 elongation factor Ts

MTKAELVKELRTRTQASMSECIKALDASENDIEKAII*LRENGAIKAANKLKNAATDGVTLAKKVGNKAILIEVNCQTDFVAKNENFLAYANQILEEALAKVESKEDFDKLIINGKPIAESGLDLTAYIGEKIVFRRGEILKANDQQTLGVYTHNNNRVAAIILVDGKVEDEVVRNVAMHAAAMRPRYLNEQVVDQV*LAKEREIIVNQLEHEGKPAAFAAKIIEGRLNKILKENCLVDQSYFKQPELTIEKYLKNNNAVAVGYYSYEVGEGIEKAPQMSFADEVAAQMKK*

>UUR10_RS00740 Ureaplasma_urealyticum_serovar_10_str_ATCC_33699_NC_011374 F0F1 ATP synthase subunit A

MENYNPLDIMIALPHIAAIIIVTLIIATISLIYFSMIRKLTVHDVPNRFVIIIGMIVDYFRGLVVDTMGAKHVKLAPYVLFTFCYIFTANLVSLFGFKEATTASSVPLAMALATVVGGQIVALKYQKASFFLKFTFKIKGFPIMVNPLEIVSKLTPIISLTFRLWGNISAAAILLNITY*AFAGFTNVVP*VGVSLIAAVIILPILIGYFTCFAGTIQAFVFTLLTSIN*GLEIKEGEEHYAHLAHKKAEKLAAKKLAELDAQNQAQNNEVQVVL*

>UUR10_RS00885 Ureaplasma_urealyticum_serovar_10_str_ATCC_33699_NC_011374 tRNA-binding protein

MYIYYNRTSLNDTLIFLKNNEQYDHQVFLNDDLLLFYRNKELIGFNL*NASKYLENLSEGYLYPSLELMQKLSNLTKTKLVPNDDFKGFIVGVILEANLIPNTHLHVCLVDIGNQQVQIVCGAQNARVGLKTVVATPNLLMPNGNEIKKSKLMNYDSFGMLCSQKELNIEGFNSQGIVELNEQYQVGQLFTKVYSNLK*

>UUR10_RS01130 Ureaplasma_urealyticum_serovar_10_str_ATCC_33699_NC_011374 30S ribosomal protein S3

MGQKVNPNGLRFGINKQ*LSR*VPTDQLQMAK*LVEDDKIRKYLSTKYKNAGIDHVEIERDQQRVNVYVYAVQSGLLIGTEASEKKLIELAINKIVGRKQLVSLKVVEVQIPELQASLMAREIADAIENRVSFRIAQKMVIKKVLKAGARGIKTHVSGRLGGVEMAREEGYTQGVMTLHTLRADIDYSMQEAHTTYGIIGVKV*INRGELFGNKLVNSVAHAANKEFSRSSKPKKGSFNRSSRSKNTKPAPKQAVSE*

>UUR10_RS02400 Ureaplasma_urealyticum_serovar_10_str_ATCC_33699_NC_011374 cysteine desulfurase

MDNYKQFFP*FKNNKDVVYLDSSATSLKPQVVVDAIVDYYTKYSTNPHNSDSNFAFHPHKIMYETRANVAKFINADFEEIVFTSGATESLNLIANGLRPYLKKDDEIVLTYVEHASNLLP*YKLRDDLGIKIVFANQKNQFPQLSDFLNAISPKTKIVSFASGGNLIGNILDENVIIKHIKQLNPNILVCVDATQSVQHRMFDVEKCQSDFMVFSAHKLLGPTGIGVAYIKNEWIKKLQPLKYGGGMNFSIDLDSYQLYDDYMKFEGGTPHVAGFYGFNAALKFLMDIGYEKIHDHELKITQYAREQLALIPQIKTYVQDPTSSTITFSYEGVFCQDFASYLGTKNIIVRSGLSCAKIINNIIQTECAIRASFYIYNDFSDVDKLVQAIKEYQKGDELNGIL*

>UUR10_RS00530 Ureaplasma_urealyticum_serovar_10_str_ATCC_33699_NC_011374 hypothetical protein

MAKYIKTGVSYINLDNARTINVLPEDIDSYLELGGDEAYQTSDLGSELYINYADFESTNILFDLKKEELQAKIDAFLVSNDTILDLSEVFLDVHFSEDDDYEEDCCCEDECCGDEENEVCCNSETKSLEVEEECCGGTKDDCCGGHEHEHHHHQH*

>UUR10_RS00850 Ureaplasma_urealyticum_serovar_10_str_ATCC_33699_NC_011374 phosphopyruvate hydratase

MKIVDLLAYQVLDSRGQPTVAVKLFLENDQSVVAMVPSGASTGTKEALELRDGDANYFFSKSVKLAIQNVNNIIRPHLLNKNVLNFFELDNLLINLDGTENKTKLGANALLGVSIVIVKGGAVAASKPLYQYIKEDLMHNYDEHYYAPIPLMNFINGGAHADNNLDIQEFMIVPLNAISFSQAIQTGSEIFHELAKILKANHLNTAKGDEGGFAPMLNDNYAALELLVRAIKKAHYFPSKKQGVCLALDVASSELYENEKYVFKKALSHNTNLEQTSFSSDE*AKY*SDLASQFPIISIEDCFDEND*NGFSLFLKNNPHIQSVGDDLYCTNLKYLQKGINFKATNAILIKPNQIGTISETLDVIKYAQENNINTIISHRSGETEDTFIADFAIGVGAGQIKTGSLSRSERIAKYNRILEIEQELKDKLVYEPNKFFKFN*

>UUR10_RS00895 Ureaplasma_urealyticum_serovar_10_str_ATCC_33699_NC_011374 ribonuclease M5

MNKPIIQEVIVVEGKTDAQKIDQLVNAQIITTNGSEISKKTLALIKQAQLSKGVILFLDPDYQGEKIRKTITNYLKYGITKQCFISKDSMLDNAKKIGIAEANNDALLKALKSQATFIINPIESIS*LEYLTLNLNNKKMRLMLCDYLNISYCNHKQLFKRLNMMQKTLEQIKAIIKEF*

>UUR10_RS00730 Ureaplasma_urealyticum_serovar_10_str_ATCC_33699_NC_011374 F0F1 ATP synthase subunit B

MLDKRREYIAKEITDAENAKQEALQYLENAKSEHLAAQAETAEIIAKAKSESLTLRELLEKEAREAADKIISSAKISIANERRENLERLQTEAREAAYIAAEALMKKELSREDNDKLVDQFIKELETNEK*

>UUR10_RS00855 Ureaplasma_urealyticum_serovar_10_str_ATCC_33699_NC_011374 6-phosphofructokinase

MSQTSFLNSTKNILIITSGGDAPGMNASLVSLIHELMDSNFNVFVGIEGLLGLYNNLIEPIKNKHIFDVYFKEQGTIIKTSRFIKLNVNDEKTQVIKKNLLEHNIHKIIILGGQGSMQAGLVLTDLGFEVYGILHTIDNDFNQTQMCIGASSAAHFNQQLLTCLNYTAKAHNAFSLVEIMGHQCP*LVNNSIGQLKPILTLTNQDPKYSVDQVIDLVKTKITLAKEYDPLIIVQELIYDQQ*YEALKKAFAQKLHQTLRVTILNYLQRGAPVIDFDLQLAKDSASVLVDFIINKNEIENTSNMYVVVNKNDIKPQVIKFND*

>UUR10_RS02250 Ureaplasma_urealyticum_serovar_10_str_ATCC_33699_NC_011374 5-3 exonuclease

MKKAIVIDGNSLIYRAFHATYKQAE*AVENQLMPTNAIKLVASMIFKILNEDQFSYALIALDASKKTFRAQEYAAYKATRKPMDEKLVVQLPYIKKLFTAMGFHIISQPGIEADDFVGSFSNLMSKSNIDTIIYSTDRDMLQLINPNTKLKLLKTGTSIVQEINLANFALLNNGLLPKQIIDYKGLVGDSSDNLVGVKGIGPKTAINLILKYTNLENIYANLEEITPSVKNKLIEHEKMAFLSKKIATIQTDLLLDETLENFILKPYNIQELDTLFESLKINNMHNYYK*

>UUR10_RS02735 Ureaplasma_urealyticum_serovar_10_str_ATCC_33699_NC_011374 DNA repair protein RecO

MAEIITKGYLVARRDYDVFDEILTFINEHGNRFVMFAPGTKRITSKNARALFFGNYLEIQFFHATNETKLSKLKKVIPLDQIDYKYENTYSMLILSELMSKVIDFNVEFYQFYQLILQYIILEYNDYYISCFLLVKFLIMNGINFNFRSCAYCNSSKNIKTFSLVDRGLVCVNCESKIINKIDYDPKALQL*QKLYFASQVQKEQIEDNELTFKGLLKILNSIMYDQLGIYLTIIKNI*

>UUR10_RS00110 Ureaplasma_urealyticum_serovar_10_str_ATCC_33699_NC_011374 iron ABC transporter permease

MRIKTRFQQFLKYQFEQKTFFRFKKNKVFLPFVLLILTISLMLIAVAGASRFVNVFSYLID*KIISQLLISGIALGISGYIIQRLTKNRLADSSLLGMGNINLVILTVLFLIFDFGQLQVQRRIEYILPFIYLIGSLIICFFIHFLCNSSGGYIFKRIIISGIVINLITIVIAQSLRILMSKESSMYLKIILLGNIESRTDFCFYFCLAMLIISII*LMANSTKLKIMVTNQQLSEQLGINSKSLTLQTFVCISLLVSTSYSLSGNVIFVGIVAANVAFNYAKNKICNGIINAGLMGGIVLLVSYIFVVLILKLSADQVILLVPIISGPYFLYQVILLKN*

>UUR10_RS00210 Ureaplasma_urealyticum_serovar_10_str_ATCC_33699_NC_011374 tRNA uridine-5-carboxymethylaminomethyl(34) synthesis enzyme MnmG

VKKYDVIVIGAGHAGLEAAFATSNLNLQTALITLDEKGIGMMPCNPSIGGPAKGIVTREIDALGGIQGKAADATTMQMKILNSSKGPGV*AIRAQIDKIAYQR*FKQQIKQQKNLDLIIAEVSDLLVENNIVKGVILSDQKIIQADYVIITTGTYLKSITHRGSVCVDEGADGTKNAKFLSDVLVKLGFELIRLKTGTPARIKKDSIDFTNMVLEPGTNQKIAFSHYHPVYKPYDKQLPCHIIYTNEQTHQIIRENLNKSAMYGGMISGIGPRYCPSIEDKIVKFSEKPRHQIFVEPESYELDSMYLGGFSTSMPIDVQEKMIRSLPGLEDCEILKYAYAIEYDAIDPTQLYPSLESKLVNNLFFAGQINGTSGYEEAAAQGLMAAINVSQKHKNKEPIVLGRDQAYIGVMIDDIVTKGVVEPYRLLTSRAEHRLALRNDNADDRLMKIGFEIGLLKPEVYDQYLNNLKQINEVLN*LKTTTVGQIDDLKFTTLKTNSYLIDYLKRPEVKLNDLLIYCPIKIEDEQIINKVQIQVKFEGYIKNQEENLKQLKRLNNIKLHGIVDYKEVPNISLETIDKLNKIKPLDLEQASRISGVNLTDIAMIKYYLERIKND*

>UUR10_RS00410 Ureaplasma_urealyticum_serovar_10_str_ATCC_33699_NC_011374 aminoacyl-tRNA hydrolase

VEKYLIVGLGNPGSNYAKTRHNAGFMVINEICNKLNLFLDNSKFNGMFAKTIYNNCVVFFCQPTTYMNLSGEFVSKMLKFYDIPIKNLIVIYDDVDTKLGVIKLRKKGSSGGQNGIKNIINLLKTEEIKRIRVGIGKDPHAKLDQYVLSNFKIDELVIIKPAIIKGALAALEAIGEDFDKVMNKFN*

>UUR10_RS00535 Ureaplasma_urealyticum_serovar_10_str_ATCC_33699_NC_011374 excinuclease ABC subunit UvrA

MDKIIIKGAKENNLKNIDLEIPKNKLVVITGVSGSGKSSLAFDTIFAEGKRRYFESLSSYARQFLGGNDKADVESIEGLSPTIAVDQKSTNQNPRSIVGTITEIYDYLRVLFARVGTPFCPNGHGQIKSQTPKQIADFLFSLPPRSKVQILAPIEIKKGYKINEVLNNLRSQGYLRVLVNNEVYQLDENLPQFLDNKKTDIAIIVDRLVLNIDHQTKTRALDAIEFALNYSGGEIAFKVDDNIHYFTQNDVCRVCGFKIKEIEPTLFSFNSPIGACEQCKGLGYNYVPDERKMIPNPNLSINEGGLDYFKNTVNTTNLD*QRFNSIIKHYKIDKTKPLKELDRKEIDLLLYGSDEAIEIDITSANNKNYSSIDYVEGVLELVNRRYQETSSEMAREHYNKYMSEKVCKSCKGKKLSPQALSILINEINIIDFIEKNINEGIDFLLHLQLSEAQAKIANPILKEVLDRLGFLKNVGLEYLTLARPASSLSGGEAQRIRLATQIGSKLTGILYVLDEPSIGLHQRDNDKLIKTLKEMRDLGNTVIVVEHDEETMLAADYLIDIGPQAGVNGGYVIAAGTPQEVMQNPNSLTGQYLSKQKDILVPKTRRSGNGHKIILKGAKHNNLKNVDLTIPLGKFICVTGVSGSGKSSLILETLVKAIEYTNFNPFVIPGEYKDLIGASNVDKIVVVNQDAIGRTTRSNPATYVGVFDDIRTVFENTIEAKARGYTKSRFSFNIKGGRCERC*GDGTIRIEMHFLPDVYISCEECHGKRYNDETLQVKFKGKSIYDVLKMPIDEALVFFENYPSIHRKLQLLVDVGLGYLELGASSTSLSGGEAQRIKLAKFLQRKPTGKTLFVLDEPTTGLHIDDVAKLIKILNKIVDGGDTVLVIEHNLDLIKVADYIIDVGPEGGNNGGKIIATGTPEQLLIKKDISYTAQYLEKYLKKN*

>UUR10_RS01270 Ureaplasma_urealyticum_serovar_10_str_ATCC_33699_NC_011374 cation-translocating P-type ATPase

VKKNNEDQTDSFVSFDPQNTDPLTGLNDEQVLKSRQIYGFNEIKKKKKSNILTKFFKQFLDFMVILLVIAGIITLILAIVKPPHDITELIVQYVEVGVIGFILFLNAIFGTIQEVKAEKNTEALSKLTSPQAKVLRNNQILIIDSREVVIGDILILEAGD*IPADALLINSSSLEVDEAVLTGESLPVQKDAKAIVKQGAGIGDRLNQIFSGTSITNGTAKAIVTNIGMNTEIGKIAKLINDQKVQLTPLQQKINKLSKIIGAFASVLCIAVFIIYIYLVGGGN*EIN*HPALVMAISLSIAAIPEGIVAIVTIILSFGVKQMAKKNALIKRLPAVETLGSANVICSDKTGTLTQNKMTVTKVFTNILKTTDLINEKDVYELIK*ASIANNGSRNFNDKKQEYEFIGDPTETSIIEAALKLNIDKSELDKEFVRIHEFPFDSTRKLMSVIVRNNDNYYLVTKGAIDAIEKIVVEPITNDVYKANDFLGKQALRVLGVGIKKLAFLPTNFNQDELERELEFIGLVGMIDPPRPEAQEAVEIAIKAGIRPVMITGDHINTASAIAKQIGILNEGQEVLSGHELSSMSDEELINNVERYSVYARVSPTDKIRIVKA*QSHDKVVSMTGDGVNDAPALKAADIGCAMGITGTDVSKASSDMILTDDNFATIINAVSLGRSIMDNIKRIIVLLLITNLAGLISLIFGIIILGINPMSSLQIL*INVIAETLPGIALGVHLADANLMRHKPLKKSAPIVNKKM*MTIFINGFFIGLISILLFYLGASSHFDFDFIAMRNEFKELANLEAIYQNV*N*LGENHEITNIVHEKIIAIKTPIMAGSSLTFIFMGMSLAFNALSLRSNHSIFINF*KNSKYIVYSIIISVIMIIVITYTPHLNEVFNMNPYNMNGYE*FNVFPFVLFTIPLGIFEVIKYVKYLKLRRSFDYKNKTYASLNQEIKSLNLKINNTKINYEKEYYKALLNNLIVKRKILINKCHKEI*

>UUR10_RS01670 Ureaplasma_urealyticum_serovar_10_str_ATCC_33699_NC_011374 hypothetical protein

MNNSDKQTNNLDENQQKPTTNDFFNDKNNQLTKVLNIPSVVKYTESKIKKSGKNFSINLIITTTTMFIVIGIIITIAIAIGIKLG*

>UUR10_RS00940 Ureaplasma_urealyticum_serovar_10_str_ATCC_33699_NC_011374 cation-translocating P-type ATPase

MLKNNLIKKFQLLPINKRRYLISLTKTSIALLISIPLMVFEMLFMFKSNLILGLDGYFIYG*IVFVLSIFIVFGLGFSFFKGAFFEVFK*KKPGMSLLVVISTCVAFIYSTYSLISNTIIYKPKLHGFFETACMIIATMSVGQLVSDRIKLKANQDLQSLNDLQVKKYNSYDLNTKQVSEKVVFQAEINEYALVKKGEIVPLDGVLYSQIAEVDESSLTGEARPILKTINNDIIAGSINVGDNFIFKITKLYNDSTIKKIINGVNQIASSKPKIQVVADKISL*FTPFILLMAILAFLLQAFVPSIQELPIAFLNLHGSNNDSNLYEKAAYVAVSVLVISCPCAFGIAVPLAVLIGAGHGAKSGITFNNSNIFEKIKKVNAIAFDKTGTLTYGKLQLKQVIGNDQFLDLIYQMESISLHPLAKSFVTYAIINNISMSTKLIDIKEVAGVGIIAKDIDGNVYELTSEHYANENQFDFSLINQKSSTSTNLLASNIIFSINKKVQSILVFEDEIRADAYETIKVLHENNIETYMITGDNTKVAQKIANELGIKHFYAQVKPEEKANIIKEIQNQQKTVMYVGDGINDLLALKQANVSISIGETNKATNAVADISLIKPDILNIYKVIKLTKTTKMFIVSSLL*AFGYNLIFIPLALIGIIPPFISVLIMTTSDIAVVLNSLIFRLLKMRLVNRKQAHKLDLKIIDKAMLHK*

>UUR10_RS01005 Ureaplasma_urealyticum_serovar_10_str_ATCC_33699_NC_011374 guanylate kinase

MKRGKLIVFSGPSGVGKHTILSKIIDRKELNLAYSVSMTTRKKREGEINGVDYYFVNDEEFKKAISNNELIE*AEFVGNKYGTPRFVVEKLRNEGKNVILEIEVVGALQVLELFKNDDLISIFLLPPSLDELKNRLLKRNTETLETIEKRIQKASHELSIKDHYKYNIINDNPDHAANQLAEIILDEIKR*

>UUR10_RS01585 Ureaplasma_urealyticum_serovar_10_str_ATCC_33699_NC_011374 transcription termination/antitermination protein NusA

MSNSFKSKEFIEYFKDTAKQNEIELEVLSSIIKEAFEKTYLRTHPGENFETNINLKEGTINCFRNLVVVENEKAHNEDLETCLDDAVEILLDDARKINANAQIGDTIKQYISIDDFKSIEVGQIGSLLRQKITEIHNKRVADF*KPSLMKMIRAKVAEINYNKQRNEITGVKVELDDQ*KTLGYLSRKDRIGDEKFKVGETYDFIIKEVKEQSRL*PVLLSRTEPELVEEILKREVVDIKNGNIEIKKIARIAGFKTKVAVSTNLLNIEPVAVVVGNKGLTITSISKQLNNERIDVIRYADDKRIFIANAIGLDKLKGLLVQENESDQRSAIAIVSKEDLPSVIGRGGANIRLIAKITE*NIDVKTIEQAFEENVVYEKFDEKIYRS*NIESINKKNVTNDEMLALIDNMQDEKVEKTEQVKDQLKQQEKQTIVSNNDDSENDDEQLEYLEGFEDFKF*

>UUR10_RS02125 Ureaplasma_urealyticum_serovar_10_str_ATCC_33699_NC_011374 AAA domain-containing protein

MEFKQKNTSNPLEEFGRNLNQEILDNKIDPIIGRDEEIRRTIEILSRKTKNNPVLIGEPGVGKTAIVEGLAYRIVHKDVPSNLLDKTIIELSLSSLIAGASYQGQFEERINAILKEVKKSNGNIILFIDEIHQIVGMGRNQGSNMDVANILKPMMARGEIKLIGATTLDEYRLYIEKDQALERRFTKVLVNKPTIQETLTIMRGLKPR*EAFHGIKIHDNALIAAVNLSERYINDRNLPDKAIDLIDEAAAKIKTQINSQPIYLDEIKRDLQHLQTEKAALESEKDEKSIKRLNDILEKIKLKQDEFNNLNDIYLKEKKQIDDLKNLRQKIERIQHDIEFYQSEGKYEKASRLLYSDLPALEKQRGELENKINENSSQKMIVDSLTENEIADVIARATGIPLNHLLADEKTKLLSLNKRIAAHVIGQDEAVKLISDAVIRGRAGINNPNQPIGSFLFLGPTGVGKTELAKTLAKELFDSQKALIRFDMSEYMEKHSVSKLVGAPPGYVGYENAGLLTESVKRKPYSILLFDEIEKAHPDVLNILLQILDEGSVKDAKNNEINFKNTIIIMTSNVGAEALLENNKTNALMELQKTFKPEILNRISEIIFFNKLSKEVIFKISENLLKELSDLLAKQDYSIKFNDNIAKIMVDEAYSSNYGARPLKRWITKHLENEIAKLIIENRITKNRNYEINYDREADQVLIK*

>UUR10_RS03215 Ureaplasma_urealyticum_serovar_10_str_ATCC_33699_NC_011374 hypothetical protein

MSKKNQAFPNRLLNYKNIIVLVALLALMIISHLIVELIVDYHQYRVNVFIAYTCIFMVVFLYVIFSRQAYRRR*AAKLRKYNEYKLSPWEIKLVDRFYTNAKYELRSVDYATIRLNILITIITLPSFIYALACMIAYIVKTVQSTT*

>UUR10_RS00835 Ureaplasma_urealyticum_serovar_10_str_ATCC_33699_NC_011374 triose-phosphate isomerase

MVKMKYIIANFKMNATEELINHFLNNLISFDEQKLTIGLAPGDLYLKTFVDLSQTKKVKLYAQNPSAYSKGPYTGQISCLQLLDSNIKNTLVGHSEIRIDCSQSIIDQKTKICMDLLDQVIICIGEPLDVYEQKKSLSFVLSQLANVINYKGLKKIIIAYEPI*AIGTNLTLDLKHINHMIEGIKTYLYNCTGLNIPILYGGSVNANNIKELCTQKLIDGFLIGNASLDVNNFNQIINACK*

>UUR10_RS01050 Ureaplasma_urealyticum_serovar_10_str_ATCC_33699_NC_011374 site-specific integrase

MKDFIRYTKKRNLSLNTIRTYESVLKHYEPVLDS*IKIRNKIINSNFKPRTIHLHKNVLLSFFEFKKLKRYLQNLKLLKLPQIEMKYFDVISKNNLYKKTDILDDDSLEIKKYKTIIRFLFETGIRAHELFFLEPVNNRLYVLGKGNKKRQIFFVKQTFEQLQKFYENLKGFETTKTLRLYIKKIIGKNFTPHSLRRSFATFMLIKGANPKTVMLQMGHANIQTTFSYLNLNEQTNRRIYNKIMYQNDAE*

>UUR10_RS01305 Ureaplasma_urealyticum_serovar_10_str_ATCC_33699_NC_011374 hypothetical protein

MINQNKRFCFQYFKKQSNQPISIKESLKKYFDFKNQSVKSIIYNLVLAAFFLALFLIGRFLTKNLDFLNGFS*QLQMGIFVLAIVCIPNFYYKLMYYIIAPLVMLAIGFSAEPFFGYLMPHYGFGLILFIDVILIITKK*NQTIKKQIF*TYLLVFSFSIIGYFIVWMGYSIQGTLFYNTPFAPSMIYNSLVTFGSMAINFGLYIIAIPVIAALKNKYSTKLI*

>UUR10_RS01665 Ureaplasma_urealyticum_serovar_10_str_ATCC_33699_NC_011374 50S ribosomal protein L32

MAVQQRRVSKSRKGMRRSHDHLTVSNTVACNECGKALLPHRACRDCKTYRSIKLSIK*

>UUR10_RS02280 Ureaplasma_urealyticum_serovar_10_str_ATCC_33699_NC_011374 hypothetical protein

MKTINLKKEFIQYCSSLKTQDFVQQYFNDDQLKEKLLKNRLVGLISVIVGFVIGVAIIICGAFVNKGNGANLNSGAIVCIIIGIFIFLCLLIPYSIVANQNKKIMHDYIEHHFNEANVAQLIKKISTFFQEFRLLSGSFLNFKQSSQVINVNYCNDQYSLIYSFQPQPQVQLMKNNNSNKISSKE*DLKGNYDLKIYELQK*NAGFSKFYFENNVESIAFQIETKLGTIINDFEELLIKGGF*

>UUR10_RS02360 Ureaplasma_urealyticum_serovar_10_str_ATCC_33699_NC_011374 TlyA family RNA methyltransferase

MSQRLDHYLKNTNQCETRSKAIDLIKRGFVFVNNLQILKPSFLVQENDFVDIKNNENNFVSKGGYKLFKIIQELNLKIKDFIIADLGSSTGGFTDCCLQLGAKKVYAIDVGVDLLHPNLKNHPKIVNYEKTNVKNLDKSYFLEELDLIVGDLSFISLEQIFPTIEKISSCKTLLLLLIKPQFELGKDIANKYKGIIKNKNLQQLAINKIINLANNYGFKYKHLTPTDIFDQKKQNQEYMIFLQKYAK*

>UUR10_RS03000 Ureaplasma_urealyticum_serovar_10_str_ATCC_33699_NC_011374 30S ribosomal protein S7

MRKLKPQKRQVLADPVYNSRLVTKLINAIMYDGKKGLAQSIIYSAFEIVEQKTGKPALEVFNKAIDNVMPIIELKVRRVGGSNFQVPTEVTPERRQTLGLR*ITLYARLRHEHTMIEKLAHEIIDASNNVGAAIKKKEDTHKMAEANKAFAHLR**

>UUR10_RS00215 Ureaplasma_urealyticum_serovar_10_str_ATCC_33699_NC_011374 16S rRNA (guanine(527)-N(7))-methyltransferase RsmG

MTRKDFFNVLTRYFP*VDEQTFISFEKYKTIIQKYNQIFNLTRLDSDDKIYQNFFLDSLAPYKELDFFTQNTNLKLIDIGSGSGIPGVVLKIIFKNLNLTLLEANQKRCEFLKILTQELGLNDVLI*NMRAEDLTQSMRESFDVATSRAVASLDKILEISAAFVKVNGYLIQPKSIKFYEEELKAKNIIKTLYLERIALKDF*ENDYHHLVGVYLKKQITPLQFPRP*NLILKKPL*

>UUR10_RS03395 Ureaplasma_urealyticum_serovar_10_str_ATCC_33699_NC_011374 ribosome biogenesis GTPase YlqF

MDIINKKIN*FPGHMKKATDEILKNLKNVDFFIQLVDARCPITSSNNELIKQIASKPIINLANKADLSD*NTNFNNDFLLISTKKVNDKNLVIKHLYQLFEQKIKTYQKKGLVNPKFIGMIIGLPNIGKSSLINFLAPKKTLKVENRPGVTKTQSIRQINQHFYLIDTPGIFLKDIQKERDGFVLTLINCIKKEVLELEAIIRFAYEFYLKNYQKDLFIRYKINQVMNFEDFIDYICQLYNYKLVNNEFDYSRAYENLFNDFCNGLICKVNYDQ*

>UUR10_RS02195 Ureaplasma_urealyticum_serovar_10_str_ATCC_33699_NC_011374 glycerol-3-phosphate acyltransferase

MDQVYSVAMAYILTLIISPLYSYLIGSLNASIILSLLLKKQDIRHFASKNAGMTNMTRVYGKKLGILTLFLDIVKPIITISLTYIIYKYALNAPFVLSNGFNQAILVYFGGIFTIIGHCYPIFFKFQGGKGVASYGGFLITIDPIVAVIGIITLLIILLITKYMSLSAMITATITCFLVLIPGINYIPYYNEHFVEYLFDLNHVIKGTWYV*LFLLISASILIYRHKTNILSIATKQERKTFLFQPKPKNNI*

>UUR10_RS02975 Ureaplasma_urealyticum_serovar_10_str_ATCC_33699_NC_011374 hypothetical protein

MATLIEKSPFIVRRYIFKKRYCVFTTL*EVIKDSSSFITSSLFSSLALLLITVIIALHKFNGAFAATSISYITIFQFSFIQLGGNLGVVLAL*AKRLYDGSKHKFVPAHQTVNLASFYAFTFGLVMSGVYLASAYTYNLYANIHQNTLFAQLFGEQYL*SSLALVVLAPVHNYFLISI*SNDRRKILFTIILDFATWTTCLVTSFLLGKYSVLAYNGYGLGLSIGYIA*TIIISFIKFHKE*KINNFGLSLNLLKITIKQI*SQTVLSVFASVAKMFVLLALYHLINQKMVGSVPLNLQSSRIL*YQSMLFIQGFGFGFADYLFYVFQKQMIRDRRYHSRQLFICIFSLLFIYSMIAAIIFGFSIKPLSAVYAKEQNQAYIHLESKIPEHFYASIRLKMLENPKLVYLLGQKAHINPKLILAHLASNDPKV*KIVIEQLITPI*RAGPNFQYLYGIPNPITKSFVSISAEGIEKLLTGDNTYIHLAIFGIFYSLSSSLMRYYNLITRRLDLPFVSLIFQILVIAFVVGFGVDYQSGTKFAGLLA*SMPMSISSAVILIFSLFIFTTTYSKFLSTYSYKDNNFENPYPFKWIRHIL*

>UUR10_RS03135 Ureaplasma_urealyticum_serovar_10_str_ATCC_33699_NC_011374 AAA family ATPase

MSTKDLNDAILDLFCLVINNNDF*KDVILRLEAKDFPEKVQQNIFNTIANLNEQKYKISESNILNGLGNYVIVDEQDQNYLLHKNYLVQILERTDYLVDLKDCIEIIKNASIKNKLDLFANEILSTQISLTNAKDQFKEMHEKFLEILASRTEDTIENMELIANRYFEKLNKIGNSGIIPGVIKTKYDNIDKFTNGYKPGELVVIAARPGIGKTTFCLNVMVNNVNEIIEYNQNIQPNQKEKIIVMFSLEITKEQILQKFISIKTGISNREVIENKYRIAKGYDTRSFAMQAINEIKS*PIFVDDRPNISIVDIEAKLYDLKKRYDIALVVLDYLQLVSAGNANKNMTRTQEVGRVSSALKVIAKEINAPVIAIAQLSRKAEERDVSSNANMKNNPLVKTIDNSPKLSDLRESGSIEQDADVVAFLH*DRKQRNAMQNDNQETRMRDDLIEAKFIVEKNRNGSTGETDIIFSKLNSKFIRATTSKE*

>UUR10_RS00645 Ureaplasma_urealyticum_serovar_10_str_ATCC_33699_NC_011374 ABC transporter ATP-binding protein

MKIKKEKDNDINSVSNENENKKINIKDLSKDERKELKKRIKEVEKLNKNHKGDIDMPSRDPNNIIELRDVKKIFTNGYLINETLKGVNLDIRKGEFVIILGPSGSGKTTLMNIMSGLDRATDGDVRVCGKQLINMSQNKLTDFRKEYIGFVFQQYGLLPTLSVEENVEIGADLQVDKKRRIKPQDALKAVGMLEYAKKFPHELSGGQQQRVSIARALAKNPIILFGDEPTGAVDETMSKIILKEFVKVNQELKTTVIIVTHNPIFAELGTLVIKVKDGNINELIRNDHPKSVDELK*DEQ*

>UUR10_RS01055 Ureaplasma_urealyticum_serovar_10_str_ATCC_33699_NC_011374 amino acid permease

LINNKIKEVKIKKKLEKNKKVSLMGFVLLTASAIIAFYTFPSLSTAG*IAIIFAFITAMC*FIPIGIAAAEMATIKGWTEGGIFTWVRNLLGPRNGFIVT*LQFQVTFGFVAMILFVLSSFSFAFAGADGYNYFNTLKLGTTLNQATSVNFSKNFNNGALYGIGIVVVAGLIFMSLLGQKRTHQFGQVALIIGILLPFFIILGFQIYTVATMPDPLYFIGKHFQKEPHLDPSVTGTSYELNKTFMSSVVMASFMAFSFSLHGVEVSAIAANRMDNPSKKYPQAMMFVVISALVCIIIGSVMISMTVPTSTLSFNGGLVQTIMFNLSVGGIDVKLPDGTVAHYKTLAHAYSVVETQYGTAAANQVMYQFVYGHPINTGSDLARSDLLTLTSEVNDPLKNPSLFISVMNVETNKMAMQGLQAITFFIGIGVFVEVAV*MSNLSTGLNYAMRKAHFPVWVTYRLKNGSALTVAIL*IALLMCIFAIYMFGYNSLMQYKGQTISDVLANEIRIDQNQKEPMALLSNQNLDEIKLMARSIYEHSHAPGAQANVLHFDFNKHANSEVNAIYEKIKDYIGINAVASGTPASITNISFISNVVSQISMYYIGYNIFLIGYLRYVFKAN*LRREFRVK*P*LQISLALLAMGVNTFAVVATYLPAAPELYPGQEAYYVFISIALPMFVCTLLLGLFIYLINYA*NKKRGINLSEHSNLTNVDQNVLDVINN*HLYKNQKEFAKFERHVQLLQQKQVIVRTTKDKVQRELVANEMQYLAAEIATRFAPEEEH*

>UUR10_RS01620 Ureaplasma_urealyticum_serovar_10_str_ATCC_33699_NC_011374 elongation factor 4

MDKKFIRNFSIIAHIDHGKSTLSDRIIEFTNTLSKREMTNQILDSMDIERERGITIKLNAVQIKYHARDNNEYLIHLIDTPGHVDFTYEVSRSLAACEGAILVVDAAQGIEAQTLSNVYLALENNLEIVPTINKIDLPSADPERVKKEIEDVIGLDTSDIPLISAKTGLNIQDVLEAIIKHVPPPLDANDDAKLQALIFDSFYDSYKGVVCLVRIKQGTIKVGDKIRMMANNKDYIVSELGIRTPKIVNKTELVAGEVG*VAAAIKTVKDINVGDTITHANNPADKPLPGYKKILPMVYCGLYPIDTSQYDDLKEAMAKISLSDAALTYEYETSQALGFGIRCGFLGLLHMDVIRERIAREFNIELILTAPSVIYKIELTNNQEISIDSPAKMPEPTNIKAIKEPFVKLAIITPDNYVGAIMELCQSRRGSYQDLEVIDGTRRRLIYKMPLAEIMYSFFDSLKSITKGYATMDYELIGYQAEKLVKIDIMLNGNKVDALSIIAHRDFAYGKSKIICERLKEVIPKHQFEIPIQASIGSKIIARETIKAVRKDVIAKCYGGDVSRKKKLLEQQKEGKKRLKAIGNVDVPQDAFVKVLSEN*

>UUR10_RS00955 Ureaplasma_urealyticum_serovar_10_str_ATCC_33699_NC_011374 ribonuclease III

MDNKKFLDFLKQNRIEPKNLSIYLEALTHKSYANEHKLTKNYQRLEFLGDACVEWVISNFIFNYKIKDNEKMRSLDEGEMTRARSNMVRSEILSYAAKDLGLTDFLMIGVGLEQDQSARMEKIYEDIFEAFIGAVAQDQGIKKVSLILEKTLIKYFREGQINYQKDYKTIFQEQAQRINKKPIMYKLVRNEGDKKEVHLV*NDLIYGIGIASTRKEAEILAAKNAILKLDDYTKKA*

>UUR10_RS01060 Ureaplasma_urealyticum_serovar_10_str_ATCC_33699_NC_011374 hypothetical protein

MKKNHKK*LLMTTPVLLIGGLSTLALTSCKDEKKSNPKPDKQSTIEEYKLSLSSVETGFNEAKLIFDNNDSFKSLDNFKVILKNIQNTNEVVEANNLSATIENQKLVLKLNNLQDGQTYKIDKILYQTNKEVILSNNQVFTTKTKPKIEYEVQEKILKLDDKTYYVVLKVKDPQNLNEPTKEILKSVTFTSKISNVANLKNAILENQRPYVKEDYSEIQIKLPRVPVISEKIEITANNSLFKSFTITVGEKHIENVEKIIDITKKVFGNQELKSISITKQNSSSANITLSLKLNGNSIIAGANKLINKKDKAIKFSFFVKNKATNTIKEFNFNEKSKPIGSYQFTLNGLDANSDFVIEDIRYDDKSIKITEELLASLSFSTK*

>UUR10_RS00635 Ureaplasma_urealyticum_serovar_10_str_ATCC_33699_NC_011374 uracil phosphoribosyltransferase

MHKIINHPLIKDKLTRMRKVSTVSTVFRTNLEELTQLMVYEATKDLELNEIEIETPVVKNAKGYKLKNKICLIPILRAGIGMVDGVKSLIPTATIGHIGLYRNEETLKPVEYFKKFPKNISESDVIILDPMLATGGSVVEAVNIIKKYNPKSIKFVCIVAAPEGLEYVQKIHPDVDVYIAALDDKLNENGYITPGLGDAGDRIFGTK*

>UUR10_RS02540 Ureaplasma_urealyticum_serovar_10_str_ATCC_33699_NC_011374 DNA topoisomerase IV subunit B

MANKYDGSAIKILEGLEAVRKRPGMYIGSTSSAGLHHLV*EIVDNSIDEVMNANAKNISVVLHEDNSISILDDGRGIPVDINPQTKISTVETVLTVLHAGGKFDESAYKTAGGLHGVGSSVVNALSA*LICEVYRDQKIYQAKFSNGGHIDQPLKVIGTTKKTGTLIHFLPDPLIFKNLFFNPNTIKERLHESTFLIKDLKISFEDKINNKKYEFINDQGLIDFIKFINETKKTFSDVIFFKNTINKIDVEVAFQYSDQNNEIMVSFANSVKTSEGGVHENAFKNALTSVVNNYARKHDLLKEKDKNLEGDDIREGLSSVISLRIPESLISYEGQTKNKLFTPEANEAVKKTIEDNFSF*LEENKTQALDLVNRAIVARDAKLAAKRAREETKKVKKIKEERGMGGKLTPAQSKDPTLNELFLVEGDSAGGSAKLGRNKKYQAILPLRGKVLNVLKARLVDVLKNEEIASIFTCLGTGIGAEFDLKKLKYHKIIIMTDADTDGSHIQVLLLTLFYRFMRPLIENGNIYIALPPLYKLTNKNTKKFFYA*DDVELDQLKKEQKNYEIQRYKGLGEMNADQLFETTMDPSKRLLLRVNINDILQAERQINTLMGNDVSIRRQ*IDNNIDFSVIDELQINNEESK*

>UUR10_RS03125 Ureaplasma_urealyticum_serovar_10_str_ATCC_33699_NC_011374 uracil-DNA glycosylase

MK*KEFIINQTKQDYLRNIIQKVNTIENHQVVYPLKKQRFRCFNFFDIEQTKVVILGQDPYHTPKMANGLCFSVDLGNNLPGSLVNIFKALEYDLQIKRTNPDLSD*AKQGVLLLNTVLTVNAHQANSHKDFGYDQLIKNAFIELKKQKHVVYLL*GKQAMSYIDLIDKDHNLILCAPHPSPLSAHRGFLTCKHFSACNDYLIKHFRTPIKW*

>UUR10_RS03220 Ureaplasma_urealyticum_serovar_10_str_ATCC_33699_NC_011374 hypothetical protein

MKTKSNLRTRLIAFISTILATIVISLVLITSCSTTNTKKPTISQDTQIKFKSITNNSINLANEKQILNQAQDSETKVFFNLYESLKIYFEQTIKRSLYLYYSYNFALLNLYNAKYHVQLNDFSLIYNRNQNPNDTSFDVNYQVSLVVDDLGDINIKTKPQFYEPIPPDQKVKNEDYRFTKKIKNFWFGSKKAILDYFNQAISMRINYSNVKIYRSIVKYPDSKDHYEGYTMTSGSFYR*FKQAMSIDDDQNKVRYMINEISDLNVDDDVKLFNLNEAILKQTFVIDRPPKIPIKPERDLVYANI*NNSLNDFNFSPIAFLAPTFIKKDAQKLTPLDLKTNKEYLNDLYTKVLVEGQKNNNALIKGKYVIYNMYDYFDLTLEPEKPGGVHHCHADGYCH*

>UUR10_RS02875 Ureaplasma_urealyticum_serovar_10_str_ATCC_33699_NC_011374 leucyl aminopeptidase

MTLNEKKEFVYELKAVKKDHNLKGFEKNTAEHVVFGEVYQKQHYLILTPDFDSNELSRSLVSFLEKSPKPVSVDLNSFLDLVPESRHASLLNVVVSALEYVEVTPFSLKSKVEPKNIHNLVVDAKYHDLIAKLQVIAQSQTITRTLQDTPANLMTPGDFEERIKELFKDLPEVKVSVLYRKDLEAKGMNAHVGVGKAAVSDKAQPRLVVVEYNNNPDTDEKYAFVGKGVCFDSGGYNVKTGSHMR*MKFDMSGSAIVSMTVRALALNKEKVNVVAVCPLVLNLLAPEGQKPDDIIKSYNGKTIELDNTDAEGRLILADALTYAVRDLKASKLFDIATLTGAMIFALGDTYSGV*ATNNDI*NEVVVAADYAGELV*RLPFHNDFLKMLNSNVADIANSVTDPRGGSSRAACFLKEFTEGVPYAHFDIAITADVGHKGTGVMLRTFYRIAQNQKFN*

>UUR10_RS02905 Ureaplasma_urealyticum_serovar_10_str_ATCC_33699_NC_011374 ribosome recycling factor

MNFKIYETKIREEFELVLK*MHNEFIKLRTGRATPAILDGILVDYYGSMTPINQLANISVPEPRVLAIKPYDRSSIKDVASAINASNLGVNPQVDVDIIRLTFAAPTEEVRKNLAKKAKQVGEEAKIRVRHIRQEAQDLFKKNSSTVEDDKKFFQTELDNLTKELNKEIEAVVSHKEKDIMTV*

>UUR10_RS02235 Ureaplasma_urealyticum_serovar_10_str_ATCC_33699_NC_011374 methionine adenosyltransferase

MQYKKIITSESVGAGHPDKICDQISDAILDECLSQDQNSRVACEVLACNRLIVIAGEITTHAYVDVVKTA*EIIKPLGYDENDFTIISNVNKQSVDIAQSVDKTNKNLIGAGDQGIVFGYACDETPQYMPLTSVLAHELLKEIERQRRSKEFIKIQADMKSQVSIDYSNSTPLIETMLVSIQHDEDYDVEYFNKKVSAIMEQIAKKYNLNTNFKKIINSSGRFVIGGPIGDTGLTGRKIIVDTYGGVGHHGGGAFSGKDPTKVDRSASYFAR*IAKNVVAAKLAKQCEIQLAFAIGQPQPVAMYVNTFNTNLIDETKIFEAIKKSFNFDIKTFINDLNL*TTKYLPVATYGHFGRDDLDLS*EKLNKVEDLIKNSK*

>UUR10_RS00485 Ureaplasma_urealyticum_serovar_10_str_ATCC_33699_NC_011374 YbaB/EbfC family nucleoid-associated protein

MDFQKLAQELKKMQNTLSKKQKEFEEKVFDFDYKGYVLIKIKGNLTIESIEVKTEIVDPEDKETLQDILRAAVNEAISKTCKERDAIMNSTIPKGTGFF*

>UUR10_RS01205 Ureaplasma_urealyticum_serovar_10_str_ATCC_33699_NC_011374 type I methionyl aminopeptidase

MVIVKTEKDITAIKEAVRI*KIAREAIYEQVKAGVSLKELDLLAKEVIEANGGIAAFHNYLGFKGHICISVNECVIHGVPTDYILKDGDKVTFDVGVKYDNHYCDAAFTIIINNSNVEALKMSDICKKSIDEAVAIIKPKVTTHAISNVIQKFIEKNGYFILRDFAGHGCGNEIHEDPLIPNYRSLLYRNVTLEENMVICIEPMILSGSNAYYIDPNDQ*SVKSKNHQMTCH*EHMILITKDGCEVLTA*

>UUR10_RS00690 Ureaplasma_urealyticum_serovar_10_str_ATCC_33699_NC_011374 hypothetical protein

MKIIGSAFLGIVFCILLAFAIIFGIEIDYYHQGDYLKYLNFLDKLHQYNKIDNLFEYSNHYESALIGVIVLTIICFLIFITPIIIIAIAKVKKKKVINKNI*

>UUR10_RS02565 Ureaplasma_urealyticum_serovar_10_str_ATCC_33699_NC_011374 hypothetical protein

MIYKEIVRNIIKHPLILPEGKHYHLHKILTSKDELRKGEVSLVAENGKEYTVDLSAFVDLLVDGDCIIDDNHSLVALYFDESK*

>UUR10_RS01370 Ureaplasma_urealyticum_serovar_10_str_ATCC_33699_NC_011374 HU family DNA-binding protein

MSEKIKAKTRVQMIDELSKMLNIDKKQTKTFMDTYEAFLILELSRAKEVRFGNIGKFKVTVRAERKGINPKTGETVIIPEKTIPKFTFTKGIKEIINAGISVEDETVFLDDNDYEDDGDEFVEEYIAPESN*

>UUR10_RS03415 Ureaplasma_urealyticum_serovar_10_str_ATCC_33699_NC_011374 class II fructose-1,6-bisphosphate aldolase

MFSLVNAKKMVQNAYKNHYAIAAININNLE*IKAALLAAQETNSPLLLATSEGAVKYMGGYDNCYAMVVNLMKQMNIKTPVCLHLDHGTYEGCIKAIDAGYSSIMYDGSKISIQENIENTKKLLAIAKSKNVSVEVEVGSIGGTEDGITSEGELANVNDCYQMCLLDIDMLACGIGNIHGLYPEN*KGLNFDLLKEINIKVNKPIVLHGGSGISEEQILKAISLGVAKININTECQIAFSNALQDHLIKAGDLVAAKQYDPRKVLAYGVDAIKNTIIEKFTKFNSLNKA*

>UUR10_RS03055 Ureaplasma_urealyticum_serovar_10_str_ATCC_33699_NC_011374 50S ribosomal protein L33

MAIKRGVRLQCNESKSINYITTKNAKNNPDKLSLNKFCPKCRKVTTHVEIKKK*

>UUR10_RS00425 Ureaplasma_urealyticum_serovar_10_str_ATCC_33699_NC_011374 DNA topoisomerase (ATP-hydrolyzing) subunit B

MNDSNKENKYTAESIKVLEGLEAVRKRPGMYIGSTQSEGLHHMI*EIVDNSIDEAMGGFATVVKVIIKKDGVIRVEDDGRGIPVGIHEKTGLSGVETVLTVLHAGGKFDNDSYKVSGGLHGVGASVVNALSKNFKV*VNKNYVQHYVEFINGGHAIEPLKIINDKDIKEKGTTIEFIPDFEIMEENE*DELKIMARLKQLAYLNKGVNIEFESEMTNRKEK*HYEGGLKEYIADLNAEKEPLFDAIVYGEEEKEVKVPGHNDQTYNIKCEVAFQYNNSYNNSTHSFCNNINTTEGGTHEEGFKLAITRLLNKYAIDKKYLKDTDDKITKEDVSEGLTAIISIKHPNPQYEGQTKKKLGNSEVRPYVNEITSIIFEKFLNENPEESKKIVAKVMQAAEARRRSHEAREATRRKSPFESNSLPGKLADCSNRDSSVTEIYIVEGDSAGGSAKTGREREFQAILPLRGKIINVEKAKIDKIFANEEIQNMITAFGAGIGPEFNIEKLRYSKIIIMTDADVDGSHIRILLLTFFYRYMLPLIQNGNVYIAQPPLYKVSYGKTIKYAYSDQELEKIKSTLLNTKYNIQRYKGLGEMNPDQL*ETTMDPKNRLLLKVNIEDAAIADKTFSLLMGDDVTPRKEFIEKNAKYVKNIDA*

>UUR10_RS01400 Ureaplasma_urealyticum_serovar_10_str_ATCC_33699_NC_011374 peptide-methionine (S)-S-oxide reductase MsrA

MVKSI*VAGGCF*GIQKYFDSIKGVRHTIVGYSQGNVINPSYEQVCTQTTNHTETVQVDYDDRFVSLTSILEHLYQIIDPFSLNKQGEDIGNQYRSGIYYVDHEDALIIKNFLLQKQNQTSKKIMIEVHKLHNFNIAEEYHQKYLDKNPNGYCHVNLSLSKKRFN*

>UUR10_RS00260 Ureaplasma_urealyticum_serovar_10_str_ATCC_33699_NC_011374 DUF2714 domain-containing protein

MKKNRSNLTPTTNYFDVFNTYKEKKASVDLITYEELMASVLFDNKLGFESEVYLDFVKKFTLAFEKKLDI*FENFIINFNLNLKFSTTIMIPILVTKANSTTDAINFRNDQNPVYNNFLISYNQKIKKLLLQNHPVQILPHLILFKSNLNGSLVLVFSEKIIASIEQKSGN*

>UUR10_RS00640 Ureaplasma_urealyticum_serovar_10_str_ATCC_33699_NC_011374 tyrosine--tRNA ligase

MHNLIKDLKARNLINNITNEEKLKKALAENKGIYVGFDPSADSLHLGNYIMIMLLKRFRLHNIKTFALVGGATGMIGDPSGKSAERNLLDKTILEHNITKIKYQLEKFTNSQVINNYDFYKNMTFLDFLRDVGKLININYLLEKEIISSRLDVGISYTEFSYNLLQGYDFLQLYKNDNIAIQAGGSDQ*GNITTGIEIIRKSLGDDNIACGLTINLLTNSEGKKFGKSEKGAIYLDENKSSVYEMYQFLINQTDADVEKLLNFLTLIDVDEINKIMQAHKENPALRIAQKALAQAVVVDVHGQQKYEQALHISQVLFNGNINELNQEEFNIAIKSLPTTKLDKDEIKIIDLLNLANISSSNRVARDFLSTGSILVNDIKVNDENFLVKKQDAINQEFSIIKKGKRNYFLIV*NKD*

>UUR10_RS01695 Ureaplasma_urealyticum_serovar_10_str_ATCC_33699_NC_011374 molecular chaperone DnaK

MTKEIILGIDLGTTNSCVAVIENKKPIVLENPEGKRTVPSVVSFNGDEVLVGDAAKRKQITNPNTVSSIKRLMGTKEKVTILNKEYTPEEISAKILSYIKDYAEKKLGTKINKAVITVPAYFDDAQRQATKNAGIIAGLTVERIINEPTAAALAYGIDKLDKEQKILVFDLGGGTFDVSVLDMADGTFEVLSTSGDNHLGGDD*DQVIIN*LLKSIADEFNIDLSKNKMAMQRLKDAAEKAKIELSGVNTTTISLPFIAMDSSGQPINFEKELNRATFDNLTKNLIERLKKPVLDAMKESKLSLADIDQVLMVGGSTRMPAVQNLVKELTGKEPNHSLNPDEVVAIGAAIQGGVLAGEIDDILLLDVTPLTLSIETMGGVATPLIPRNTKIPVSKSQVFSTAADNQPSVDIRIVQGERSLAADNKLLGNFELSGIEPAPRGVPQIEIKFNIDANGIMSVNAKDLKTQKETSITIKDSQGLSQEEIDKMIKEAEENKEKDAKVKHERELVNRADSLINQLEQVVKTENVPQEQKDAFNKQIEELTNARDAQDYTKLEAEVKKVEDLLANAAKFAQQTQQQDPNNQKDDVTEATVTDDSTKK*

>UUR10_RS02220 Ureaplasma_urealyticum_serovar_10_str_ATCC_33699_NC_011374 DUF5378 family protein

LSTNYFLIAITILTIICIGLGNFFIKPVQTQKIN**T*NHFINTK*IINILISISFLIYFCCLR*APGAKDFFSNEMEIIKTLNDKYANYINSYEASITFSRTFLLD*CPCFSVLISIVAIFDKKQIIVNYLGFLCFCFGLLTIVGGLVGDEAIG*ENLINYVFIGKSPNTIYFALHFYLCVFGFYLFVNTRKTIKIYWIYIIIHTILIGYIIYVNVMIVIFNVKNNASGFSFGD*YSPVYAQYSTVAQVLQLDYRLNALVMCLFMYAIFVVGYLIRYYLQKVYINKYNIKYSQEGSYFAYVKNHR*

>UUR10_RS02325 Ureaplasma_urealyticum_serovar_10_str_ATCC_33699_NC_011374 urease accessory protein UreG

MKRPLIIGVGGPVGAGKTMLIERLTRYLSTKGYSMAAITNDIYTKEDARILLNTSVLPADRIAGVETGGCPHTAIREDASMNFAAIDEMCDKHPDLQLLFLESGGDNLSATFSPDLVDFSIYIIDVAQGEKIPRKGGQGMIKSDLFIINKVDLAPYVGANVEVMKADTLKSRGNKDFFVTNLKTDEGLKSVAD*VEKRLQLALLEE*

>UUR10_RS02395 Ureaplasma_urealyticum_serovar_10_str_ATCC_33699_NC_011374 SUF system NifU family Fe-S cluster assembly protein

MASYNINDNLTLRSIIMEHYERPKNKVCFVQNETDYLSCHNTTEGCSDDITVYVKLVNQKIVDVVFLGTGCAISTSSTDIICELVKNQDLQQALELINNYLNMIQGLEYNQDIMQELIAFHNVKNQMNRIRCARIGINALKTCLEQYKQ*

>UUR10_RS00080 Ureaplasma_urealyticum_serovar_10_str_ATCC_33699_NC_011374 BMP family protein

MKKSKINKKILFASFAGVATLASVAAIAASCNDSNKNNGGTSQDGNYVSKLVLENFYSKPAGDIGDNPGYHRTYNSLYDDGARMLGLISFSHSGPIAEYFKSSNAKKDLSAVLIDDKFSGNEGKDRIASVSYRVDQAAFLTGIAAAYYLNANQETFGKDGKLT*GGYVGLHFTSTSTFIQGFKFGVQ*ANEKLKDKNINQEDANGTNKK*MNVEQVFANKYVAGSFKPDEEGATNIINDLITKKADVILPVAGPQTNIATSIVSNATDPSVIIGVDTAQELDDVTNRKRITNKTVNDGKTILFSIVKRVDLAMKGAIENASKGAQLTDDITKDAYKLGTHTEASLDKSTYVDDTPLVELSNAGRVYLEQAAKLAGLKAITYAQIVDAIQNEDLFKLLSQKGTTTLENVATKTNDG*TLKNEYSNKPFTELQKLLGGEVYINKLDKKLYPYSLTGSSYLEEDKTKRSASQEFKKA*DAATTPEAKEKLAKVVLGQNNAVIKDKSFSESAYNGLAAFYKSKKIIIPKI*

>UUR10_RS00970 Ureaplasma_urealyticum_serovar_10_str_ATCC_33699_NC_011374 hypothetical protein

MLKKTKFDSQLVKSLITSSILKVPGIFSVDISDKLSDFNQNRFVIKISLHEDVVNVLSVANEARNLVYYELSKQLNDDSVVINIIINC*

>UUR10_RS01030 Ureaplasma_urealyticum_serovar_10_str_ATCC_33699_NC_011374 ammonium transporter

VSTITTTS*GIDPNIYFSKHELT*VPATADANTLALAHFNPTSTLLVALAIAFVLLMTPGLALFYGGLTRRKSTLTIINQCVASLGVTTLI*IFGGFSLAFGPSVGKGIIGDISTYFAFRNLLFADG*SGDAGIVFANFTNGVPLILFFAYQLAFAIITPPLMVGAFADRMKFKNYLVFLVL*QYLIYIPFAH*I*GQGFLAAAGVIDFAGGIVIHTSAGFGALAASLVLGKRVLLKNDKSRPNNIPMTILGATLLFFG*FGFNVGGSGFVSGGNAAV*SLATSA*ISTIIALAIGMIG*AILETIFNKNHKPTGVGLVTGAIAGLATITPAAGYVPI*ASVPIGVAAVLVCYASAKTLHHFHKIDDTLEV*GVHGMGGVTGSLLIGAFASKSVNPSIMYEAIGPETTGILFGVQLGAMLLAAVYAFVFTILLVYITRPRLSARQQLGHIDYINHGEDAYAFDIEIPSEKEMEQYELADSS*HSSTGVKHPLVKKANLQAGESTSSH*

>UUR10_RS03150 Ureaplasma_urealyticum_serovar_10_str_ATCC_33699_NC_011374 single-stranded DNA-binding protein

MNKVILIGNLVRDPEARQIPSGRLVTNFTVAVNDNIPNANANFIRCVA*NNQANFLTTYLKKGDAIAIEGRIVSRSYVDNNGKTNYVTEVYADQVQSLSRRNQNANDHNNDKVNVDTMMGAYASINTDAAFSSNQPQTNFQSTTSNSNKNDDEEDEITS*INLDDDLE*

>UUR10_RS03305 Ureaplasma_urealyticum_serovar_10_str_ATCC_33699_NC_011374 50S ribosomal protein L13

MQKSSMLKKEAAIARRQ*YLVDATDLVLGRLSVKVADILRGKNKVDYTPNVDAGDYVIIVNSDKVVLTGQKALREN*YNHSHYIGGLRTRSGEEMISKYSDELIRRSVKGMLPKNKLSKQILNKLFIYKNDKHSHEAQQPTILELKLK*

>UUR10_RS02085 Ureaplasma_urealyticum_serovar_10_str_ATCC_33699_NC_011374 ribosome biogenesis GTPase Der

MRTIAIVGKPNVGKSSLFNRILMRRKSIVDDQPGVTRDRIYDVGN*LTRDFMLIDTGGIISSEDTYQDNINEQVLFAINEANTIIFLVSAKDGINNDDKKIAKMLKEKAKDKKVILVVNKVESEKYYFNEGELYSFGFGKFFKISAEHGIGMGDLLDELVKDMPIQNALDQQERFKFCIIGRPNVGKSSLTNTILGEQRMIVNAEAGSTRDSIDNDFSYHNKKYTIIDTAGVRRKGKIVEAVEKYAVLRTQKAIERSQLILLVLDGSEPFKEQDEVVGGLAYDANIPTIIVVNK*DNIVNKNSHTMEMVKKQIRSQFKYLS*APIVFISALDNKRIHTIFETIELVREQAMRKVATSLLNDVVIKANAFQEPPPFKGGRISISYVVQVQSQIPTFVLKCNNPKFLHFSYARYIENEIRKAFGFDSVPITLY*QDKNKKLRGE*

>UUR10_RS02545 Ureaplasma_urealyticum_serovar_10_str_ATCC_33699_NC_011374 DNA topoisomerase IV subunit A

MSVNQQKIINTPLDNIVGESYAKYAKYIIQDRALPDIRDGLKPVQRRILYAMSELGIFHDKPYKKSARTVGEVIGKYHPHGDSSIYEAMVRMSQD*KNNLCLLDMHGNKGSIDGDNAAAMRYTETRLSKIASVMLTNLKKDVVKFSPNFDDSEKEPSILPSLFPNLLINGATGIASGYATSIPPHNPNEVFDALIYRIDHPDCSIEKLIKICPAPDFPTGGEIHDLNGCANAHKTGEGKFVIRASIEFKTSEAKINQIIINSIPYETNKALIIKEIEDIIYNKEVAGLIEVRDESDAKGVSIIIDTKKDVNLENVKNYLYKKTSLEISYNTKFIAIVHRTPTLVSLSTYLDAQINHSLDVINKVDLYDLNKVLLRIEIVEGLIKCVDLIDEIIKIIRASDSRQDAKNALIQTFAFTNNQAEAIIMMRLHNLTRTDIFDLRNE*ESLQQQAKTLKERIKSLQVRKNYLKQKMIEFKKEFGYQRKTKLFDEFIKAEVNEDQMIEKQSLNLVISRDGYIKTVSKKSFESSKYDELGLKTNDLLFYHNVINSHDRILIITSKAKLINLIAHKISCMR*KDVGEHLNNYAKFDANEKVVAVYVCNEQFKVDEHQLVLGSKLNLIKRIELNELDLNKNSKQISIMKLNENDGLISANLIKKDHNQFVVAISKLGLVLMFLVHEINCLNRLAKGIKIMKLKPNDEISSILIVPNNGYSIQLFLDQGNKCFSISELKLSKRAMTPSPLYLPTKKAQSVLAAFLVGNENVFYLLDEQQKINPYYLPNLKPIKLDSKINKYENDLIITDVVKDSFLSDSVISDFKKISMYANEFDSELLKTNENQEQDDLQLELINEKEEND*

>UUR10_RS03005 Ureaplasma_urealyticum_serovar_10_str_ATCC_33699_NC_011374 30S ribosomal protein S12

MPTIAQLIRNKRAPKVKKTKSPALLFTYNSLHKKTTKNPSPLKSGVCTRVGTMTPKKPNSALRKYAKVRLSNGFEVLAYIPGEGHNLQEHSVVVIRGGRVKDLPGVRYHIVRGAGDASGVEKRRQQRSLYGAKRPKKEASK*

>UUR10_RS01280 Ureaplasma_urealyticum_serovar_10_str_ATCC_33699_NC_011374 YihA family ribosome biogenesis GTP-binding protein

MAKFIKSAQYFDQYPVDKQFEICVIGRSNVGKSSLINALANEKIARTSNTPGRTQLVNFFDFNSFRLVDLPGYGFARVSKDKQLDLATIIDQYLGYRQNLCAVFQICDINVLTNDDVEMSRYFENQNYAHFVVLNKVDKVNKSHFDNNKQKIAKFLNISVDRLLCVSAQKNTNVATLFALMKKVVIETRQKQLLLKKEEKKSSEEEIK*

>UUR10_RS01815 Ureaplasma_urealyticum_serovar_10_str_ATCC_33699_NC_011374 hypothetical protein

MKNKYFRKSKFWLLIPISFISLGSITLVATACVKKNFYPNTFADDQIYITKNNEKTVKFRISRNDEENKKYFKDDLK*NTFINKLKKTYINVETVIPKENAKQNIKQQDLCMRALPVQVLSNNKYVDIYVKIPVLLANTKMLVVGDDLLKSFYFDTDNLIMNAKHFQPIPIKML*

>UUR10_RS01830 Ureaplasma_urealyticum_serovar_10_str_ATCC_33699_NC_011374 CDP-diacylglycerol--glycerol-3-phosphate 3-phosphatidyltransferase

MAKLNFKKNILTSPFFRNIPNIITIFRIFLALICIILLLVDFYTKNLNIVYEVLDANISALRLSATTIFIIAAFSDFLDGYIARKYNLVSNLGKILDPISDKILVNGVLICLTLDHTALAYLTIINILRDIFIDGLRMFASSKKIIIPANIFGKIKSILLFISICFILFLLSLTSS*KNVYLFNIPLFFATSLSIVSAIIYYIDFYKGVKKRGSITKS*

>UUR10_RS01430 Ureaplasma_urealyticum_serovar_10_str_ATCC_33699_NC_011374 YebC/PmpR family DNA-binding transcriptional regulator

MPRKHLIASGINKKQQQQAKI*MKCAKEIKAAAKMGGPNPEANPRLKVAIERALNNNLSRDSIERNINGASKDADNLKELTYEGYGPNGLAIIVRALTDNEQRTISAVRGYFSKLQGQIAKPNSVSMLFNEYGQLLIDKKTKTLDEWFELLVDQDIVDINEDDEIIEILVQPKDFSATKLILENNNANIQSAEIKLIPTDFISLDDHARERLVRFVNACENDDDIS*VITNYEEEL*

>UUR10_RS00220 Ureaplasma_urealyticum_serovar_10_str_ATCC_33699_NC_011374 hypothetical protein

MSSLPVIILVIGIFGSIFLVIGYIPQVIKVIKTKRTDGISLTFLISLNIACFLFVIYSILVMIFNKHNGIPTALPLCLANTIVGILGLVILIYKVKNIKKAKLYLMDEKTYYEKYVLNNL*

>UUR10_RS03490 Ureaplasma_urealyticum_serovar_10_str_ATCC_33699_NC_011374 hypothetical protein

MYSRLLSNILFVGFHTNHNTNEKTTHIAIT*AINTHTFGKSVLMISAGFNSGISPVHEANIGMNITDKERTDSVATKCFLFNFITLLP*

>UUR10_RS00810 Ureaplasma_urealyticum_serovar_10_str_ATCC_33699_NC_011374 Spx/MgsR family RNA polymerase-binding regulatory protein

MIYVLYSPNCAVCKKVVRFFRNNQIEITKIIIGEDKIERSMLIDILSLCEDGFGTIISFKTESSKRLNITSKTFLDLSTKELLNLIQEDLNLIRRPLIYQTKNNKPYRLQIGYDSEEIEIFKRVVHEGR*

>UUR10_RS01190 Ureaplasma_urealyticum_serovar_10_str_ATCC_33699_NC_011374 50S ribosomal protein L15

MQLHNLEYKKGSRNHKEKRVGRGHGSGLGKTSGRGQDGQKARKSGMVRLAFEGGQTPLYRRVPKVGFNNDRFANKYNVVTLISLVKYETKELTAEFMYVNKIAKNEDLPIKVIGNAVLPSGTVVSAHKFSKGALESISNSKAKAQILE*

>UUR10_RS00405 Ureaplasma_urealyticum_serovar_10_str_ATCC_33699_NC_011374 tRNA lysidine(34) synthetase TilS

LTKL*TNLINKITNKKYLAAVSGGPDSMAMLNMYKRNISVVCHVNYHKRESADRDQEIVVDFCKKNNLPIEILDVDEKVYEKYAHIDNFQAKARLIRYDFFKEIGKKYNIQHLYIAHNFDDFLETAYMQRARQSKALFYGIKESNVVNGMIVKRPVLFIRKQTLQRYCDENKIKYGIDETNELDIYERNRVRKTISN*SLNEVYDFKKAVLKYNKEHSSFANFVELSYIEFKKNKYRYDYFVRQDDGVQYYLIYYFLIDQKISNPNENKIISLIKFFGKQINKEKAYRVQENLYMHVNEDDLISLISYDKNDVIDDPNIIEKQAGN*

>UUR10_RS02095 Ureaplasma_urealyticum_serovar_10_str_ATCC_33699_NC_011374 division/cell wall cluster transcriptional repressor MraZ

MFIGTYNHSIDSKNRMLIPSKVKATLNEVTFVYLSLGFDENIDMRLESEFNQFVDNINNLPIGSREARNLTRLLLSQTYKVEIDSASRILIPQNLIDKAKIKKDIYIIGTNDRYEI*AKEVYDDFSLNQEDTLSDLAEKLLINGI*

>UUR10_RS03190 Ureaplasma_urealyticum_serovar_10_str_ATCC_33699_NC_011374 PDxFFG protein

MKKRLNLSAKIAIAFASVGAFAGAVVGFMKLYAVSTGGLGRQILQVNSSFDTRIPNRQVNRALLMNQFGKPVAEYDFKAKKGNNTPVTLLVDFGKYKEGQKISYIEFLDTFISLNNNNLPNLKLEVGPIVFSNNYINSVSPDEFIEFTN*FFTNVS*GPDLLTLKEFKLSRGIQQNGNSITLGLHSGTREKTTIEFFPDAFFGSLPIYNINAGAGNAYDSLARQLNQDGMILEELEKYHQKIPLMIASHNSHSFGGIKLVNALKEAKNTY*FNADKYFDLTKDPLNFAGLKGVKAKDRTLNYLFYAENEQQAKQKVQAYLNELQFVKGLIPKDKKFNPSIDVKADEIKKIEIKEFLELGGALPSFPGGSVDLESLGGNSVGQKTLLIDALVNNQKQKIKYHVFEDEFISRANKSNDSEFIQNDRSILASSLREIITKNFNNYISQKQNFRNVYDTDETFVGKSFYIYDANGNGKNPRFYKTKESLLQSELVNYDEKKVSLVTIDKTKVEGPNKLVLTTKDKKEIVLESPANNPKGYKYDTSFDDAFNTLKVAANYFDTFTPRLIKQGSKFINGKIVKTYTIFVDAYSGLLDKVLNKNRHLLQKINGIHTEVVQNKDGKNTYKVVNGEYEGIYATDRIPYLSLVAESDPAFKTTGINYLKYVSTHEYGHHQTLQDMKDISDSDESVVGGGIDSRSGVSDESYVNGQALQDYLNARSSGITFRKTDVNYQPTKTGSFLNFSLNNDPKNPI*ETEKDIFGSATADDPKAFFNNKKRRFLQKFDELQEAAKLRNVKPYDLFIMNSFDHESATVNPSFSSEMKDPSRLKAEYFFYNNQENQNQKNDKFNFGSVVEQPGLLRYKGILKDGMGTPIEFDKKTGNPIIYKLKDSKKPITKDKVEILVKTKNNTPVIDLSTCLNKNGTVNLTKLKRQANQIINSINSLIVKNYYNGG*DESGNFETSMFNFQTYINHPLFTSTEKRK*AERITNAMFKHPDFNISKSLKLPEKTNSTSTSVPYYKKVLSQIIGQDVTNKDFNGFKRVLLDASYKTSKLLDNKTDEELKALDPELLEIKKYYNKQLDR*TVKDANVVAQTTMFNYFDSGIEGNNGYKYYVKPKDRELYRTILNTKTKESFESIIGIRPSYLTQNKITTYEQLFNNFMINLNQIGTIALVVNKSNGEGKPQKTLDQLTEKEIYPLMILTEAEAKAINFNYLKSNNGAFTTVILDGNNGQKFYTIKFKDIPSLIEFMSIDPSKYTIVENALSKNHENVRK*DYDYLKERYDVDKFFNEVVKKQEAYKDITLEQFKENLTGLLFDGFTESHDIFKFYKSKDFKATELDKYKQVFDGKLGLYGFRSFGNKFDKPASPYGEPIDRYNFAGNPQNV*KAKPNQKNVRTVDSIVKGIQVEIERKHRQNNTLNFGQLLQYAFGFTIYTDKPGGSIKDIYGQLGSFFGISQSSEIPNEDGTVVS*DYVAINKKRVQEKLNEIFGDYVFNIAEVLTRDYVQTVFVPSQQELDNLPNYLSGLSDFNTGNEYVFSGDNTKQ*NERLIPVNNFLSADSNTIASNVVFATNDYEKIQSVLANQASNVYKKNQQLFDNYATKPEDLEENFAKVKNSLLSDNNFLTLSKTHDNDILNDLKSYSSLTRLSADANSYIGKTRLTNNGFFKDR*LRKIID*QIYDDNRESVKDDHLNILELDNKTKVKDRARAM*LYMLRSKGIGDRTLAQIYRNKEKDSILMYGFIKKEYKDKVKKIAIKNKNNGHISYIDVHTNNTNNLFYLKRQSDISSK*TLEDEGYVS*TTDYAILSNFTNQLIGYDSVNAKGSEFELYFVDENLKEVMDLSDPSKPKSLMNLGSRKYVAENGKSYSISPVYARNENTPTQNRTIIRISNQFSV*

>UUR10_RS03330 Ureaplasma_urealyticum_serovar_10_str_ATCC_33699_NC_011374 preprotein translocase subunit SecE

MANEKKQKTNKYLTYQDQEYERNLLLEKERKQQKKLLAQQTYRELNLHKSLIKKAKDDQKLILKLLKENKLISKTQFNAHRLELKNAINEMIDEHYQLLDKYSVDFEKLSFKLKR*FYGIGKEIRRTS*ASKRSVLVSLIIVIIIVLILAAIFFGIDSGFYKLSAK*

>UUR10_RS00385 Ureaplasma_urealyticum_serovar_10_str_ATCC_33699_NC_011374 DNA-binding protein WhiA

MQKSTLQEIIDKDLNKFTFSEFVKEDIYTNTDYSSTDYKVILYSFFRNNLTIKIGQKLT*ILKSQNLQIIEFILNGLEHFNNLNLEYEIIVEPDHLNKTRTNYSLAL*GDLDKLDEILKLFDNENDENFHKDRYCSNFLIGAMLSGGSIAHPLENYHLEIRCDSNNYITLLTKALSRYGLEYKIVYRNKKTIIYFKKSETISDFLKAIRTQNSLFEFENIRIQRDFNNQQQRLNNLDISNLSKSSKAGVLAKEMILEIKKNHEDFSKQSDKFLKYCELRIQNPDCSLNELAYLLKQTFNIEISKSGLNHFNSRIKQMYEELILKNERKN*

>UUR10_RS00935 Ureaplasma_urealyticum_serovar_10_str_ATCC_33699_NC_011374 heavy-metal-associated domain-containing protein

MTYMLLTSSDLHCGSCSSNLYNVLEKIGAQNISVNILNSEFAFEFEENKIADQDVIKEINKNGFKTNILEKYTY*

>UUR10_RS02835 Ureaplasma_urealyticum_serovar_10_str_ATCC_33699_NC_011374 hypothetical protein

MNTKEITQAFLYKSKLFRIQYLFLDV*DEKQYLKIDVIEDLRFINK*NINKIKALNHKTKEGDENAC*

>UUR10_RS02995 Ureaplasma_urealyticum_serovar_10_str_ATCC_33699_NC_011374 elongation factor G

MSKELKLFRNFGIMAHIDAGKTTTSERILYHTGKNHKIGETHDGAATMD*MAQEKERGITITSAATYAK*KGHSLNLIDTPGHVDFTVEVERSLRVLDGAVAVLDGQNGVEPQTETV*RQATKYNVPRIVFVNKMDKTGADFYYSIETMKNRLGVKATAIQIPIGAEADFVGSIDLIEMKAYIYDGQADEEYKIEDIPADYVTKAQVMRSQMIDDVAIFDDEVMEKYLSGEELSHEDIKKCIRKGVISTELYPVLCGTAFKNKGVKKLLDAVVDFLPSPIDVPPIKGVDDHGNPIEYHNDPSEPFAALAFKVATDPFVGRLTYIRVYSGKLDKGTYVYNATKDKKERISRLVKMHSNNRDEIDSISAGDICAVIGLKDTTTGDTICDEKKPVILEQMVFAEPVISLSVEPKTKADQEKMSLALSKLAEEDPTFRTYTNEETGQTIIAGMGELHLDVLVDRMRREFNVQVNVGAPQVSYRETFTEIADAEGKYIKQSGGRGQYGHV*IKFEPNHDKGFEFVDNIVGGKVPKEYIKEVENGLIEALTSGPIAGYQTIDVKATIFDGSYHDVDSSGMAYKIAASLAFKEAAKVCKPVLLEPIMSVDVTTPDDYFGTVMGDISKRRGVIEGQEQRGNAQAIKAKVPLSEMFGYATDLRSNTQGRGQYIMQFSHYAQAPKSVTEEVMAARAKK*

>UUR10_RS02355 Ureaplasma_urealyticum_serovar_10_str_ATCC_33699_NC_011374 ABC transporter ATP-binding protein

MSFFKKKNDTEKEEHKHVSVVDMDEILGYEDGEEFKDDEIDSNEAIFQLNNVSLGYGKKMIIKNLTATIHRNDFIVVLGPNGSGKSTLIKGLCRINNPSSGYIKYRDKLISRP*LPLL*LEKA*ASIVNVFTKDRKEVIKTID*HIKNYKKIHAYKSKELALNLAYVPQLAVFPEATSIYDFVKMGRFPSSNALGINTNTEREKQIIDEALKNVGIYEFRHKNLEDLSGGQKQKALIALALAQDTETIVLDEPTNHLDIRSQLEIIELLHKLHHEMKKTIVLVIHDINNGLKYAHKVMIMKNGEMVRYGKLKETIDHEILLDVFGVESIIVKNESKYPQVSVTDFSLPKDYKINEVQENERTNLIYNDGEENELEKEVSKKKN*

>UUR10_RS01775 Ureaplasma_urealyticum_serovar_10_str_ATCC_33699_NC_011374 riboflavin biosynthesis protein RibF

MIIEITSTNIQQIRDQYFINELVIGFFDGIHLGHMNLLSDPNNQTILTFKNIPRKIKKLYDFNERIQQLEDLGFKRIFIYDIDQNNLSGEEFIDQILKPLTPKKIIVGANFTYGNNFCNASSLKQYFNVEIKIITNDVSTTKIKELIINKQVEIANKLLIKPYYRVGNVVRGDQIARNIGFNTANILCDNNLIDIAEGVYKAQVIFNNKKYDSVVYLGIPKTINTRSFSMIEAHILDFNQNIYDERIKIVFLKYLAPNLKFNNIDELITAIKNYIKLVLDKTN*

>UUR10_RS03205 Ureaplasma_urealyticum_serovar_10_str_ATCC_33699_NC_011374 ABC transporter permease

MAKFGQYLKEKSKSLASSFVDELASDAPNKFLQPFQHQQ*KIIGHLIEFRDNTYMGGRPRPFREFANRYGRSFSGLIGLAIIIILFILAIIIPFTTGSPTELRPNQRYLDYFTDGFIFGTDNNGRDL*AML**GLRYSLGIAIIVTAIELVVGVTLGILMGYFEMFDKIMTFIIKILTNVPTIIILMILTIILKPSFGVMIFAISFSGWITMANQMRSQVKRARSFL*VAASKTLGTKA*RII*NFLPILVPMMITQLVFSIPGAILAESSLAFIGLSLPNTPTLGNLIADGSSIITLYPRFTLIPSFLLVCLVASIQLVGGATQDALRRQR*

>UUR10_RS03335 Ureaplasma_urealyticum_serovar_10_str_ATCC_33699_NC_011374 transcription termination/antitermination protein NusG

MAYKIKDLDSKLLSDLKIDLNHTHQ*YIVTVVSGNEQKVIENIKDKLNGYGYGDKLSDLKIIKEKIKEVKIYEPSEAPRSMKNRANTK*ETIVVDGVTKYRCTKIKEGNKFNGYIFLKAEMTDQI*FLIRNTQMVTGLVGSSGKNVKPIPVPEDKILKLIADNDAKRALVSLDEQTNSQQNVVVVESHETEDLPNFEVDQQVKIVADTFFGEIARIAKIDQNKKVATVEFEFFGRINTLDLNFNDIQPYDEEAELEN*

>UUR10_RS00650 Ureaplasma_urealyticum_serovar_10_str_ATCC_33699_NC_011374 preprotein translocase subunit SecA

MNLISKISPQNRILNHARLIAEEVLKKEDEYTHFSDQELINKSDDIIEYLANNNPLDDRLVESLCIIREVIYRVHNKRAFKVQLIGAIIVYFGDFAEMMTGEGKTLTLVLVAYLNALYKKGVHMVTVNEYLVKVGAEFATPALNFLNMSVGQITANMNEYEKRNNYDCDITYTTNSELGFDYLRDNMVTNYNSKVQRGL*FAIVDEGDSVLIDEARTPLIISGEPQEEIGNYVKADRFVKTLYPQDFTLDPESQSVALTESGVEKAQKFFNTKNYYNFENSDIIHKVTNALRANFTFFNGREYIVKKDDEGEDVIALVDQSTGRIMEGRSYSAGLQQAIQAKEQIKIEPENLTVATITYQSLFRLYKKLAAVSGTAITEVEEFLNIYNMVVVTIPTNKPIRRIDHPDYVFDNKRTK*KYVIADVIRRHENGQPILIGTASVEDSEILHQLLERVNIPHEVLNAKNHAREAEIVARAGEYKAVTIATNMAGRGTDIKLSPESLEAGGLCVIGTERSDSRRIDNQLRGRAGRQGDIGESRFFISMEDTLFSRFATDNLAKADDKLSEDVISTKFFTRLLNNTQKKVESLNYDTRKNLIDYDHVLSNQRELIYKQRDKILVSSDNKDILYRMLDSVIDDIIYQSHNEPNEDIIDVKKLIDLATQNIFYDNYLNHDEYYGLDLDEIKTKLKNDCISFFEQKEQLMTPGIFNQILSEIMISNIDEE*TKHLDVTSKIREGVNLRAYEQKAPLNIYVEDSDKLFEKLKHDVA*KTVCSIGKINYVHQEYDKVNNEFIINDNEIIDNDNVIDFENTDHSLISEQEIEDSLVNIDELNDQNTKNENND*

>UUR10_RS00990 Ureaplasma_urealyticum_serovar_10_str_ATCC_33699_NC_011374 50S ribosomal protein L27

MNKLY*LTDLQLFASKKGVDSSKNGRDSNPKYLGAKLGDGQSTKAGQIIYRQRGNKIYPGLNVGQGKDHTLFAKTAGVVKYTKFMGDKTKVSVLPKEDNK*

>UUR10_RS01460 Ureaplasma_urealyticum_serovar_10_str_ATCC_33699_NC_011374 16S rRNA (uracil(1498)-N(3))-methyltransferase

MQRYFVSKIKDKKVYFYDSDVHHIKNVMRMRLHDEIIVINETQAFIAEIINLEPLHATIIKPYFNDCELSTKIDLFQASIKPNNFE*IVQKACELGINSIYQTIFKRTYNSTLIKNNRISTIIKEACEQARRNYLVNYYEQFSFSELLKVLLNYDLILVAYENEKQIFLNDIFKENSTYQKIALVIGPEGGFEQEEIALLKKINNVVCISLTKTILRSETASLYLLANLINKLI*

>UUR10_RS02225 Ureaplasma_urealyticum_serovar_10_str_ATCC_33699_NC_011374 isoleucine--tRNA ligase

MKDYKSTLNMPSTAFEMRANLNIKEPKIQQF*IEHGIYEKLLAKNKDKKPFVLHDGPPYANGNIHIGHALNKILKDFVVSYHNMNNYYSPYIPG*DTHGLPIEVALSKKVKLSNLSVNERREQCKKYALEQVDNQIQQFLRLGMVSDFKQRYLTLDHSYEIDQLKLFANMLKKGFIYQDFKPVF*S*SSQTALAESEIEYGDRQSPAIYVKMQVVDSSELFNDKPTSFVI*TTTP*TLPANLAIAIHPELTYSLIEYKNENYVIAKSLVESFTKKVGFEDYKLIKDFKASALEKIKYISPITKKHAFIIMDEYVSANDGTGLVHNAPAFGLEDYYACKKYGIETEVIIDQFGKYNALVNDSELENMFYEDANQVILDRLICNQLLIHHELITHSVAHD*RTKKPVMYRATKQ*FVSIEKILPNILQTLKNDVKSTSFRGIERMHEMIVNRKE*CISRQRV*GVPIPMIFDENHEAIMDPDLVENIINVLNEKGVNA*FDLDVNAFLTPKYLSMKNKTFYKEKDIMDV*FDSGSSYNVLQHYNLPYPADVYLEGYDQYRG*FNSSLITGTILNNKAPYKYLVAHGMVLDGEGYKMSKSKGNVVDPLDVCKVYGADVLRL*IANSDYQNDTRISEEILKQNAEIYRRIRNTLFKYSLSILNDFEPSVDFSFDVRQEDQFVLNEFNELHLKVIKAYESFDYQTIVKLFNKFILDLSSWYFENIKDDMYCLAVDDPIRKQIQSTVY*ILKNSLIDLTPIIPHTTEEAYSFLNDANKKESIRLEDFYDQSQFQFKKGIAHVKAFFSIKDEIFNELENARKNNVLKKNNEALVTIAKNLILDDYLLNNPKLLAKWFGVAKIEFTNTTSVVNANFKKCLRC*NHFADDEMYDDELSMNCYKVINKIK*

>UUR10_RS03245 Ureaplasma_urealyticum_serovar_10_str_ATCC_33699_NC_011374 30S ribosomal protein S16

ILVKIRLTRVGTHKKPFFRIVVMDAKAKANGAYIENLGHYDPVLGKVVLKKEAILAQLQNGAQPSETVKNILSQEGI*KEFIALKDANKKRKAALAKAK*

>UUR10_RS00390 Ureaplasma_urealyticum_serovar_10_str_ATCC_33699_NC_011374 FAD-dependent oxidoreductase

MNQQIYDLVIIGAGPAGLAAAVYAKRSGLNVIIVEKQFPGGKVALTANVENYLGINSISGPELAYKMYEQVLNLDILVIYELADEITLKEKYKEVKLATQTLIAKTVIIATGTENRRLNIPGELTFENKGISYCAICDGPLYKNKVVSVIGSGNSAVEEAIYLATIAKEVHLIANKPEFKAERQMVEIVKNTSNIKIHYNKQTFEFFGEEFLQGLRFKDLVTNEITTLNVEANFTFIGLLPSRINASNLNIFNETNGFITTNKNMETNVHGIFAAGDIVDKSVRQIATAINDGVIAALYAKEYITRNN**

>UUR10_RS03380 Ureaplasma_urealyticum_serovar_10_str_ATCC_33699_NC_011374 thioredoxin

MLIKLENNQNLNQILKDNHSKPVLIDFYAD*CPPCRMLSPVLDSIEKKYGDEFTIIKVNVDHFPELSAQYQVKSIPSLFYVKNEEIKTNSLGFIDENSLVNKLRSI*

>UUR10_RS02820 Ureaplasma_urealyticum_serovar_10_str_ATCC_33699_NC_011374 glycosyltransferase

MLLTIAFYLSKTTHYLKKNFNYFLDLLNQNKKHIELIIIDDASDYNLFKTLKPLIENTNSKIKYFYLNETQGNAYAYNLATKYAHGKYI*YLGGHTELNLDASSLLFSVLEKDYDVISFNLNDNVNQNPSLVFDSLNKEVLVGL*ESISNKIIALDFIKKHQLAFYNDK*YPALFIYDLFTKFSS*RNVNVNFISNNSGEVGYNVYDLLQQINELYAKFSNDGLLEIYKDELCY*ITGICIHSFLKKIYELYTININSKKQIKERTMIISHALSNAKKYLETYFANFENNPYVRKYKTNILKYYLKSKQGLN*

>UUR10_RS02955 Ureaplasma_urealyticum_serovar_10_str_ATCC_33699_NC_011374 iron ABC transporter permease

MINLST*NKTYSFKNPLKYQNKLAYLKTIITVAISIIVFIITLA*TYDFNFKQNNIALVYIVMQIFLSGFVLGFCAYFIQKLTKNRFGDTSIMGISSVNILGVIIIAFQVDFNNFSIDKLTTLQRTEPLIFFIAPIILCSIYYFTCKEESNFNYKKMLISGVIVNFLSVALGSSIAKNLPKLANAYISRYTYGSIEPQQESFFIALVLIIIGFLIIMFNFKKIQIVSSNQDLANQLGINVKLISGLLLVAICLMVGASYSLNGNLIFIGLMAGNMGSVISNNRFKSAAISSGSCGALIYMLSFLLFIKILNFDSQ*LNLAIPLLISPYFIYIIVKKNKSDL*

>UUR10_RS01210 Ureaplasma_urealyticum_serovar_10_str_ATCC_33699_NC_011374 translation initiation factor IF-1

MADTEKLKMLGKIVEILQGGNFRVQLENGITIMSHVSGKMRVNKINILPGDTVDVELSPYDLTRGRITYRHRDS*

>UUR10_RS03430 Ureaplasma_urealyticum_serovar_10_str_ATCC_33699_NC_011374 glutamate--tRNA ligase

MKIRTRYAPSPTGYLHIGGARTALFNYLLAKAYDGDFIIRIEDTDVERNVEGGIDSQFNFLE*MGIVADESIRNPKTFGPYIQSQKLKHYEALALDLVAQKKAYFCFCSKERLDADRELAEKLHETPKYKRHCLNLNEQTIQANLLANKEYTIRLKIDENNEYS*DDLIRGKISIPGSALTDPVILKSNKIAMYNFAVVIDDYEMQISHVIRGEEHISNTPYQLAIAQALNYDLSKIKYGHLSIIVDETGKKLSKRNLALKQFVSDYEKDGY*PHAITNFVALLG*SPKNNQEIMSLVEMVENFDVNNLSKSPAFFDINKMN*FSTQYFNNISQDEFIDFVKTHPLTKELVLKDTTFIDKALLFKSHIVNLKQLINLVDEQFNSNKQLLEEDVNHIKNNQLTNVVQVFYEQLIVSEKFDEQSIKEVIKQVQKTTNNKGANLYMPIRIATTFSSHGPELAKTIYYLGRENVLKNLQSILKVLG*

>UUR10_RS03445 Ureaplasma_urealyticum_serovar_10_str_ATCC_33699_NC_011374 membrane protein insertase YidC

MSSVDKQNLMNRMRISVSHFAGASNANSTKKERRKKILNILLKVFKVIVYTFFLGIGLYGCFQNMANH*TINSTVVGNGFELGFHVDPILGANDIRFDLIYSGTGP*YPMSDFSFDYGPFYALFVWPIAQILLHFMYATRD*PAGLNAILGLIIILLIIRVITMLISARATIQTERISEIQGKIAEINAKYKDAKDMQSRQKKQMETKELYQKHNVKPLAPFESMIITLPIFLIIYRVVTILRPLKFISIFYI*DLSATPISEIFSNFTTSG*PYIFFLLIIIPVQILSQKIPQLLAKKRNRSATTVGAKNKQQLKRVRMTQNIIAIVLAVVVAISASGIGLY*FFNAIFTILQSYIIHVIIMKRRSNSATRIESKLAKLGIS*

>UUR10_RS02260 Ureaplasma_urealyticum_serovar_10_str_ATCC_33699_NC_011374 hypothetical protein

MSDKKSDFEKLFERSKVQETSIFDINDIDEQKPYEQHSIEELSYILEYEKIDKKTREKIKKIIKEKQRNL*

>UUR10_RS02680 Ureaplasma_urealyticum_serovar_10_str_ATCC_33699_NC_011374 DUF1410 domain-containing protein

MKKNTRKKFLQLFSLIALIPLSSFIVMCSKAKTQEIKEQSKIKARSIRFMDKDLTSINIQFYFDKIDIDDILVKKFNIELEDDKKNKINIDVNPIYNKKLQLLSFKLENLKPNTTYKITKFSITNQIADLTKVDNLSFNTSPENNLPNLPNLPNDPNIKIEDIKTNEANNNSIKITLNINIENNNNLENKYVRLVYKDNENKLKLSNILKINDLKKQNFILEDLTSNRKYSFEELIIGESNDLNLNNAQTKISTNQKQKFSFVTLPNPVKITAIEIDSKFDNNPSSLITLKFKDNENNLKENDILKIKYKKAGPNQVVFEKSVRLTNNLEVLFEIENIKKNEQYEIISIESNSKHGYNVNPSVFNFSSNSLRIFSIKD*

>UUR10_RS03405 Ureaplasma_urealyticum_serovar_10_str_ATCC_33699_NC_011374 thymidine kinase

MAKAHAFSKKVG*IELITGPMFAGKTAELIRRLHRLEYADVKYLVFKPRIDTRSTQNIKSRTGTSLPSIEVENAPEILSYIMSDNFDNEIKVIGIDEVQFFDDRICEVANILAENGFVVIISGLDKNFKGEPFGPIAKLFAYADKITKLTAICNECGAEATHSLRKIDGKYANYDDEIVKIGCQEFYSAVCRHHHKVPNRPYLNANSEEFIRFFKNKKRNKNV*

>UUR10_RS01345 Ureaplasma_urealyticum_serovar_10_str_ATCC_33699_NC_011374 30S ribosomal protein S21

MSRGVNVEGDLEKALKKFKRISNETKKDSKRHEYYLSPRIRRKEKIKEANKYRSF*

>UUR10_RS02120 Ureaplasma_urealyticum_serovar_10_str_ATCC_33699_NC_011374 hypothetical protein

LNLRSFEFLKGNETV*DKEKKAINVSLIKASISCTIMCILIVIFGFLMRKYLFEYSSAVSLTINSKFVFQIITGIVIVLSIIYFIVHLILYFRRRYNFFAPKIG*SITLYILDGITMCFMISYVSAIVGLDLTLIAIGISLSIIVAMGILGFIMNHKIAFKLSIANIVLFTIATIGSIILLIVFFTTRYNHGFNQGIIIADIVLSII*LISLSIGFASTIYSMRIMAEHHNLENHKIMRDFTT*NSYLLFASFNRILLYVIRLIALFKRV*

>UUR10_RS02740 Ureaplasma_urealyticum_serovar_10_str_ATCC_33699_NC_011374 glycine--tRNA ligase

MKNKFKTQEELVNHLKTVGFVFANSEIYNGLANA*DYGPLGVLLKNNLKNL**KEFVTKQKDVVGLDSAIILNPLV*KASGHLDNFSDPLIDCKNCKARYRADKLIESFDENIHIAENSSNEEFAKVLNDYEISCPTCKQFN*TEIRHFNLMFKTYQGVIEDAKNVVYLRPETAQGIFVNFKNVQRSMRLHLPFGIAQIGKSFRNEITPGNFIFRTREFEQMEIEFFLKEESAYDIFDKYLNQIEN*LVSACGLSLNNLRKHEHPKEELSHYSKKTIDFEYNFLHGFSELYGIAYRTNYDLSVHMNLSKKDLTYFDEQTKEKYVPHVIEPSVGVERLLYAILTEATFIEKLENDDERILMDLKYDLAPYKIAVMPLVNKLKDKAEEIYGKILDLNISATFDNSGSIGKRYRRQDAIGTIYCLTIDFDSLDDQQDPSFTIRERNSMAQKRIKLSELPLYLNQKAHEDFQRQCQK*

>UUR10_RS03230 Ureaplasma_urealyticum_serovar_10_str_ATCC_33699_NC_011374 YitT family protein

LNNNEEKTHPKNRIKKIFN*RNKTKEALRTENNLLKQISNEATIQQSVQIEKIRYKAGLLKFAALYSTKKIFLRYLIIVIFSLLASLLVLLLVHNTGIYSAGIMGTSQGIARILQSIMISNHSSPKEAKLAYDIMF*LFAIFVNIPLLVFSYKKIGKHFTLMTLTYIVTVQVFGFALSQIPNVEKIMIFGNNRLSYPPINSFFTNSILDEKIAQNIEANVLSYANISNLEIRNEFINYVQANPNATIGDLFHLNQTTTYRQALYEFAKPLYVFHDLIVNKVQILS*VDPNQASKIPSILIYAVIYPIFDGIFLSIIYIAGGSSGGTDIISF*YSKKYGKPTGSILTYFNVATLIIGIVLGSFAPAGMINPRY*DTQYFFSPNMVASILASIVLGIVFNIYFPKHKSLKIQVYSKNTSAIIENLRANDFNNSITLNSLSDSLSLRTNYSLEIISPYIELPSLIHLVRDIDKDCLIVIYPIIDIDGEMVVRKSTIS*

>UUR10_RS01590 Ureaplasma_urealyticum_serovar_10_str_ATCC_33699_NC_011374 YlxR family protein

MAMKVNQRTCIFSKKKYPKNELVRFVIINDELIFDLNHYRGYYLKIQKNMNLEKLYQFLKKRFMIKNEEQVYQILKQLIQSLI*

>UUR10_RS02850 Ureaplasma_urealyticum_serovar_10_str_ATCC_33699_NC_011374 acyl carrier protein

MVVNVKDIIIKVAKENKINLNMNNLDVELKSLGIDSLSAMSLIMKIEDKIGVQLVDEKLLKIKNLGDLIMAFEDALK*

>UUR10_RS01250 Ureaplasma_urealyticum_serovar_10_str_ATCC_33699_NC_011374 membrane protein

MSKFFKKQHL*K*SLFCLPLVIIPFIISACNNSSLQKQQKLVRNQISFISDGFKNYYVNLAFDQSLKNNKKMIFNVSLIDEKNQKNDLLVKDYIIKDKQILIKLPRLIK*NDRLVITSNNPLFKPINIRIFLDDVIKLDNSIITTKTSVDSQNN*TTFLNNKVIHNLLLSIYPDQKEREEYIKSQQNIKPDYTQEIAN*LNYYNTVQNDANKGPVYDKNPKKNKPKKNPFGGGSVSNPFAYQQARKAHNKLFNEN*L*FLFNLTKQIFMLYPDDNLFQESSEETVQNLKDSKVNNHSSFYRASSNEFIDGLYVIESSNLDKKPEELDEYTDFSKTAKFALLNKEGFIFTIFIEENYDKTKKLVSRQVSLVP*IKTLPKLMFNDHTIKHDFNLANYTENSYDYNAINFHSPLEIKVYGDQFGGKAIRFSFVDIDSN*

>UUR10_RS01075 Ureaplasma_urealyticum_serovar_10_str_ATCC_33699_NC_011374 BMP family protein

MRKIKLNKKIILASVIGTLGVALVPTALVACSQNKTKSDSKNLTLNNFYGRPSSDGDDAYQAIYESKIQDGARMLGLISFRHKNPISKYFNSPKDNQQVSAILIDEIYDLQTGKDRIASITYRADQAAFLAGIAAAYYLNSNQNVFGKDNKLT*GGFVGIHLPSTTRFIQGFKFGIQ*ANEKLKNKKVKQTENNEEKE*INVEQVFATDYQSGDFSPTSDKAKTIVNQLVSNNVDLILPVAGLQIDYATTTAAESSKPIVVVGVDTEQELDDNTNKARISENNKTLANGKTIIFSIVKRLDLAFKGALLKASEGAQLTNDINKDAYKLGTHTEASFNKNTYVDNTALVELSKAGHQYLIDAIKLSGLKEVNDYKTIVEKIQEDPLFKLLSQTGTKKLDEVATKSQQGD*VLKSEYQNLSFIQLQKMLGGLVYVDQKNELYPYELSNSFYLEKDPNKRQASKAFYNY*NAKDANQINLVKIFLGQSVDVLKDKSFSESIYKGLEEFYKSKNIIIPKLY*

>UUR10_RS00325 Ureaplasma_urealyticum_serovar_10_str_ATCC_33699_NC_011374 ABC transporter ATP-binding protein

MNQQSQTIKPVSKLHLIYYYFKDFKKSFIGLSIAIVL*LAATIAATFLLQETVDKYAVANGVVDTVVKMCGGLIGIYFLSFIFLLIMNEFAIRISFKIESHLSMMVINRIRYLPMKYFDVNKSGEIFTKTLSDPTSVQDGIVNFYIELNKTFFGALGFGIALLVTSPYIALIGIAIYTLV

MLFNILIFKKSRQLMRKKRQDFGEMNGYIEEMIHGQNVVANFDEQAYFVKKLDNMIKNLYKN*VKAQYSSQIIFP*SIFSMRLMNAVLIVSYLLISIKGIHLPGIVSNIDPVTNMISFGGLVSISLFGNFFCDNFSQLSNAIPIFIIARTSLAKIDEIVKTPNEID*DEKLVIDDSQGIEVRFENVNFNYSKNKPTLKNINFVAKKNQKIAIIGPTGAGKTTITNLINKFYDINSGHIYFNDVDITNKSRASVREHISIVLQDPFLFSESIYENIKKGKMNATKEEIVEAAKKAQAHELILSFEKDYGTVISEKQSLSQGQKQLITIARAIVSDAKIIILDEATSSVDTQTEHKLQLAINNLLKGRTSFVVAHRLSTIINSDLILVVKDGEIIAQGNHDYLIKNSSFYQDLYYTNFAE*

>UUR10_RS00360 Ureaplasma_urealyticum_serovar_10_str_ATCC_33699_NC_011374 hypothetical protein

MNFKSTIINKKPID*KHTIVLEELSVDPKALEMHKERINTVFAKQTEEQRAQQLHNIIVRENLFNKAMTYLADFYEIDVNEEDVKDLAPRIKQAFGVEDEKLAYEISQKIIAKALIFQDLQKEFNIEIKDDELTKILESYYEETNLSIRDFKENKAQ*EAAKSTLLEEKTTAFIVDKFDRDLSILEANIRKKIAEQMELDKKIKEVQDNSKAKQNADK*

>UUR10_RS00715 Ureaplasma_urealyticum_serovar_10_str_ATCC_33699_NC_011374 F0F1 ATP synthase subunit alpha

MTDNKNHSLISDIKSQIKKFSEKALTLEVGNVISLGDGIVLVDGLDNVMLNEIVRFENGVEGMALNLEEDAVGVVLLGDYSNIKEGDRVYRTKKIVEVPVGDIMLGRVVDALGKAVDNKGNIVANKFSVIEKIAPGVMDRKSVHQPLETGILSIDAMFPIGKGQRELIIGDRQTGKTTIAIDAIINQKGRNVNCVYVAIGQKNSTIANVVRDLEAHGAMEYTTVVTANASELPALQYIAPFTGVTIAEE*MHQGKDVLIVYDDLSKHAIAYRTLSLLLRRPPGREAYPGDVFYLHSRLLERACKLKDELGAGSITALPIIETQAGDISAYIPTNVISITDGQIFMMTSLFNAGQRPAIDAGQSVSRVGSAAQIKSVKQTGASLKLELANYRELEAFSQFGSDLDDETKRILKLGKAVMAVIKQEPNKPYNQTDEAIILFTVKEKLIPQVPVERIQDFKEYLLNYFKGTKLRADLEEKKAFDKENTPAFKCAIQKAINNFLNNSQDFKPCDELEQTAYDKFFNENEPIVVVNENEFFDEQINSLPTFESIHPVQIEEKVQELAEPREVFEVNKIEKEHLFEEVEPEKIICEHHQFEITEDQEEVDGQEVLEDENHEYAIYEVVEQNDTIENCKEANDEAEIQVPVAEVVQDEEILNERENRN*VFSDSAVSEVEKQTIMISISPNESEQLFDNGRSVVFFKVAPKYPVEKVLVYVTSPIQKVIGEFDLLKIDVNSVNSS*NKYRSSSVISSRKEYLEYFSSHKEAHALLASKVYKYRRPKDLASFNMKKGPSGFTYLK*

>UUR10_RS02645 Ureaplasma_urealyticum_serovar_10_str_ATCC_33699_NC_011374 adenine phosphoribosyltransferase

MINIDYIKSKIRDVPDFPKKGIVFKDITPLFLEPKIIEKIVDDFADFAKSLNIDAIIGAESRGFLFAAPLSIKLNKPFILVRKPNKLPNDVYSAEYTLEYGSSRVEMHKDALKPNQRVLIVDDLLATGGTVAAIENLVHQAKGIVAGSVYLIRLGFLKGEEKLSGKVHALINY*

>UUR10_RS00570 Ureaplasma_urealyticum_serovar_10_str_ATCC_33699_NC_011374 ATP-binding cassette domain-containing protein

MEKTLLHLRDITKIYDDGFAAVNKFDLKIKKGEFVTLLGPSGCGKTTMLKIIAGFEQPTNGKILYNGIDIKDMPIRLRPTSTVFQDYALFPNMTVKQNIKYGLKLMRKPKDNVDQSIYLQADKVYNSALKKANEKIKELKKQRRGLLAEIKKMDLKYQKNKNIFEIKEMRKNQYLGTLDELYQKQGINKNGKSDFLNYFKN*FSREKLNLNDPIDKEIYNLKKAYKEKSGLDKRYDKITYKYNDLDY*ESY*ATYPQLKKEQFENKNITRLLTKEEVEKEANRVIDLVGLSARKDSYPSDLSGGMQQRVALARSLVIQPEIILLDEPLSALDAKVRKQLQDELKKLHKNLGITFILVTHDQEEALSLSDKVVVMSNGQIEQVGKPSDIYDSPSSL*VANFIGKTNIFEGHYIAKGEVEFDGIASKTDVIDGFDENEACYIMIRPEDFDVVKKDEGSINARVESVLYKGLM*DIKCKYNDMIINVEGVNKVNEGDEIGLD*DDIDVHVIKKDCLSNEQAI*

>UUR10_RS00725 Ureaplasma_urealyticum_serovar_10_str_ATCC_33699_NC_011374 ATP synthase F1 subunit delta

MKSSLKPVEKYAYSIFEIAKEEKKLDLYKHNLETINSIIEEVPAFFEAVGDPARDRNERKQIVIKNLEGEIDIYLISLIDLLIDVKSIKLLKKIVLKALDFVNEALSVKKVLITTAYELTKNQIDRLVQSLKKKYACEKIEPIVVVDKSIIGGLSINFESQVLDNSLKTKLFNVVKKTN*

>UUR10_RS02320 Ureaplasma_urealyticum_serovar_10_str_ATCC_33699_NC_011374 urease accessory protein UreD

MILSKEKINNYAAYLYIKVAYDEAHNKMAHTVYFTNFYRSSKPLFLDEEDPINPCFQTISMGGGYVSGEVYRSDFEVEANARCIITTQSSAKAYKAVDGKTSEQHTNITLGKNSILEYISDNVIVYEDGKFAQFNNFKMDSTATLIYTECFGPG*SPHGSAYQYEKMYLNTKIYYDNKLVLFDNLKFQPRKNDESAFGIMDGYHYCGTMIVINQEVVEEDVIKIRDLVKEKYPDMDMIFGVSRMDIPGLGLRVLANTYYHVEKINAVAHDYFRRKLFNKKPLILRKP*

>UUR10_RS00040 Ureaplasma_urealyticum_serovar_10_str_ATCC_33699_NC_011374 hypothetical protein

MSFTSLLQNSV*EVGNGAIVTDQAPYLGIAPDYQDAYGFPTHP*GIFFQVIGAILVFGAYLPAVIKVLVSKRTENLAIGM*IISIAGLALLAVFA*LGVSTNPGGFILVALSETLSCIASIIVFALKIVNKSKAKAAGMTELEYCNLHYPIVKKLPKR*

>UUR10_RS01215 Ureaplasma_urealyticum_serovar_10_str_ATCC_33699_NC_011374 50S ribosomal protein L36

MKVRASVKAICKDCKIVKRSGVVRVICANPKHKQRQG*

>UUR10_RS03290 Ureaplasma_urealyticum_serovar_10_str_ATCC_33699_NC_011374 hypothetical protein

MMNDKLVKELDGITRLYYENDREQFEKSLINI*TKTMGYTPNLQQPKTYNEKLLYMYLKADQSKILEIVDKISFKK*LIKHRLYQYQVPTLAVFNRQNQITLLDLNKFAKPYIIKLNNSTENEDLYIVRETTSQAELMQIAIHFNDILLNKKVNQSRNFYET*CYSQIKPQVLVEPLLGKQTLVADYKIFCFEEEQFCLYMNKDEIEKNN*N*DFLKSDIFDLKNNKSILNDETIDFDFSEIKSVCQKIYQILKDDFKHFRVDFYYVDNRLYIGEITFNTSNAFFTF*DDPQ*RQKDLE*GKY*K*

>UUR10_RS00070 Ureaplasma_urealyticum_serovar_10_str_ATCC_33699_NC_011374 sugar ABC transporter

MLKFKTVLTCDNLFKGQERKTITKKFLSTVFAIFVAIVVSAILVGILGYDIRDFFIRLFTK*VGSPDIYLTKVAILGIAALSFIFAFKAGLFNIGISGQMLGSGLIMLLVVKNMQIANINMPNVLGQFFLLIIAMLAGAMFAVFIGILKVFLKINEVVSSILLN*IMFYITRYVVFTAQGKMYNPTPDQVGSRSIPFADNYSLFHLASGYGYLFALLIFISLAIII*IILKYTVFGHKVLSVGKSFDAAKYAGYRTKIISLATFAISGALAGALAMVNYTSTTTNAIVISQASDALPTQGYDGIAMGLISLTHPLATIPVSFLMGLLQQSADNLGGSFPQDLSGIIISFVMLGAAMFILFERISPVY*IYHLIYTYKGKDYYRDYENKNNNTISEYKAIYIEINKKRNQYLQELKELRKELDKFKNQKQYNGVNEQYQHTVNYIKTTYNN*LKVEYSKYLEQMKNNVMQLKKHLIIERATLVYYPEINTTRKVKNKLKHFENISAKKLSRIKDKIHNEDFLIKNLVAKEIGKYAQSKSFDYNKLKYLLQQYENNDSILTIDLIDELSLILKDEQVQVMFNEFKQASNENNLEHISSLKNEIMSYINSLLDSRLEQLSSILKSKNLVHDEQMDAIISLLNPNDKIFNKIIVSRNKHLENYAKEITKLETKTTSQSDEIEKNTKQHSTSYLTKYYQKTKQHINKIKLDQKQKLLLED*LTTAYQKAITNQQNMKEVIS*

>UUR10_RS02815 Ureaplasma_urealyticum_serovar_10_str_ATCC_33699_NC_011374 RDD family protein

MSDKTTLKQENTIEYPVVSALRRAGAGVLDYIFVSILAFLLSLLVFINSNFKISDVFVNNDELAPEP*RLFLMAIIAFFVYCTYYIIIPVFCKSYTLFR*AFSIRMISLVKQNDKKILFVALIKHSLLT*LIFIMINLLAMGFCFAFADASFKSIEAMNEIELKEHYRQIKDLKAYVQSILSLKINDNFTSTTTMAILSILVKSLYSIMGLISCIIVIHMFMNSKKRTLHDQVAKVVLL*MHYEKPKQAPKHKTKREVEIQKIDNLIQELDKI*

>UUR10_RS02290 Ureaplasma_urealyticum_serovar_10_str_ATCC_33699_NC_011374 hypothetical protein

MNIKKIKQEFSFSYELIEKEIKNIIKN*AMFKNE*EFLMATIYFSSKAIFREISLNN*KKFSSYANEIFLEINAKNNPLQTFDLDY*YLEIKKLIQELSISNHLLNEQLEQIYLKFLIFY*HNITIPKTYEIGDFSIVCLRNKALVDCMQILQEYYHINVDILIDLVIKQIK*

>UUR10_RS02200 Ureaplasma_urealyticum_serovar_10_str_ATCC_33699_NC_011374 DUF1600 domain-containing protein

LINNKKFVNKTLDNANQIRIQNNLKTYAPKFLNLIILNNLFLLIMIVSLCIFFSDPRFLYRNDVISNQYRQFIFTYQQTSIAITLYTLA*YIMVNFFFFGYILEYVKLNKLETFNFAVASILFNPYAYVIIAKN*TS*TYY*QRSLYHILSENNKIEFNIKDSRSIFALIFLIIAIPFIVFSSIDYVPKNPGRLIIFDKNSMGTEIIYTNNI*FHNMHYFTSQGN*MCIGMAFLYFINPKARFVKNNRVLLIVLSYILIVSSI*LFVLFPVFSQRST*V*FNNMVGFYNHLVTPVTFTIFAYYCIVKNKYVIHLKYFYA*KGFMFYVVIYAIYAMFLPLLANVTVYGAITNI*PSANGNPIFTTMLFALIGYEILIFTIN*MILKFINKRKKAKILIN*

>UUR10_RS03160 Ureaplasma_urealyticum_serovar_10_str_ATCC_33699_NC_011374 GNAT family N-acetyltransferase

MVSIEKINEQDINAIVAYVVAFRKRLYPEIDHNTLPKELANFKQTYIINPLGA*FSVFLEGTHELVGTISCHEYNYRYNDLFCLDKNIKTSEVGKLYIDPSIRRQGIATSLFNALREQAKKQGIQCFYLHTHHHLPGAKQF*LKMGFTIQKEMTLEDDGKDIIHMTLEL*

>UUR10_RS02080 Ureaplasma_urealyticum_serovar_10_str_ATCC_33699_NC_011374 NAD(P)-binding domain-containing protein

MSKILIIGSGAFGSALTQVLVSNHHAVDVYGINQNELNDLQQNQKNTTYFQDQKLSQPINNTYLDIHLALKNHYDFIVIVIPSFAIKNFVDSIKTLDLSQAIVVNAAKGLNLETKSS*CDYIQQNLKIKALIGLVGPSFAIDVFLKKPTVVNLVGTDLDALIKTKQAFEND*FKCVLSKQFEVANYISCFKNALAIGCGIIYGLEKSHNSLVAFLTKGINEMQLILETIYQKKVNPLEYFFIGDTILTCTDQKSRNFSFGLLVAQQGVQTALENKQKTVEGLNNIKVIYEIIKTKQIDAPLFESLYEVINENLTPKSLFNKSFC*

>UUR10_RS00005 Ureaplasma_urealyticum_serovar_10_str_ATCC_33699_NC_011374 ATP-binding protein

MSNNYQNLYDSAIKKIPYDLISDQAYEILEKAKVHKVYDGVLYIIVASAFEKTIINGNFINIISKYLSEEFKKENIVNFQFIVENEKVLINSNFLVKETVIKNRFNFSDEIMRYNFNNLVISDFNRKAVKAIESLLSTNYENSSMCNPLFLFGKVGIGKTHIVAAAGNQFANSNPNLKIYYYEGQDFFRKFCSASAKGTSHVEEFKKEIASANLLIFEDIQNIQSRDSAAELFFNIFNDIKLNGGKIILTSDRTPNELNGFHDRIISRLASGLQCKISQPDKNEAIKIINN*FEFKKKYQITDEAKEYIAEGFHTDIRQMIGNLKQICF*ADNDLNENLVITKDFIIECSVENDIPSNIIVKQQLKPEQVIEIIAKELNLKVDLIKSTTRKNSIVWARDIVCYVLKNKLNLTLTEIGKLLSGREHTTISHSVNKVEKILADKNSQEALQINLIIDKF*

>UUR10_RS01635 Ureaplasma_urealyticum_serovar_10_str_ATCC_33699_NC_011374 RNA methyltransferase

MLKVITSNKNEDIIHFYNLIHDKKYREANQLFIIEGFKLLDEALQNQQTIVAIIINERVYDKEFETIFKYTLYDNVQIYKVSENVIKKLSVNIDPSPILAIVKMKEETCDVNDNVLLLDNIQDPGNLGTILRTCFAFNVKQVIFNNCVDLYNAKTIKASMGAIFKINFLRFNSSKLTLEFLLKNNFYLIATILDKNAISLNELKIKQKYCLMVGNEGHGLSQNFIDYASIKTMIKMNQTESLNVAVATAIFLYELNK*

>UUR10_RS02730 Ureaplasma_urealyticum_serovar_10_str_ATCC_33699_NC_011374 GTPase Era

MVKKYGIVAIVGKPNVGKSTLINAIMRKKVSIISNKPQTTRNAIKEIYEDDDSAIIFTDTPGFHEPSNKLDLFLNHEIEVSYKEANVILFVSSMDKELSEDDFEIINLIKESNKENVILVISKAEVAKNQDKIDERVHQLNKYIQFKDVIQISALHVINIDKLINTIKQYLHKDVVTDYFRQKVEKEDKFIIAETIREQCLLNLNHEVPHGVGVEIDESKYNQEANH*IIKASIIIEKNSHKPIVIGQNGAMIKKISMAARKQLHEIYDCHISLTIFVKVENN*RENNNVVKSLGYKIKK*

>UUR10_RS03120 Ureaplasma_urealyticum_serovar_10_str_ATCC_33699_NC_011374 membrane protein

VTNITSVLHATNTSQGNGINS*QSILIFLAFLIVFIIFTVIKKIYYSIRFQKRFYIIPKTSVKGISNIAMVISISVAVIILLTVLSANTASVIFRA*PGTRVTLEGILVKIGGLLFGPILGIFIGAMTDLLSVAMTAGVFHYGYLIAAMAYGLIGGLIRIILTTSKKKDLPFAIYSSIATILIGVAIALFLYFAPGIGDKGFNIQLFGFDIKIARTLMIGIILGFFLLSIIVV*FSLLIKYLLNHKNKNKAKSN*FIIFAPVLVTILLTEAVVNVLMMPSFDAELSSLKYTQ*LSIRAVLFVPMVVLNLLIIYPIFKIVVPLIRYDYEDDIIEDKHIPIHVD*

>UUR10_RS01705 Ureaplasma_urealyticum_serovar_10_str_ATCC_33699_NC_011374 hypothetical protein

MNRQQIHAKTRLTINELNKFYALIKVQKQSLEDLENKIKKLD*INFKEIKDQKLFNDSKFDYKNITPYVISQIQDLVSAYNTKQISEIILELNQVNIQVQAKDSEFYIKNNNKLNLISSEFEKYSLKNYDAYEHFKITMDDPKIQTLNHQMPFESKFNTKIIIFTILGFCLLCLFIIVIVCALLIAGVI*

>UUR10_RS02100 Ureaplasma_urealyticum_serovar_10_str_ATCC_33699_NC_011374 16S rRNA (cytosine(1402)-N(4))-methyltransferase RsmH

MEFNQHTTVLLNETIELLHVKPDGIYVDCTFGRGGHSQLILKKLSKKGKLICIDQDQQAIDFANNLFKDNPNVIVIKTNFKNLKSVLYDHQIFHVDGFVFDLGLSSPQLDDPERGFSYHKDALLDMRMDQEQKLNAHYIVNHYSFAKLVNIFTKYGEIKYAKTIANGIVKERSTKAINTTLELVEIIKNYSPKKILFEKKHPARLFFQAIRIEVNDELNILKKAFNDAISMLNPLGVVAIISFHSLEDKIVKKVFNNYAKNKLPKEIPLNNYVNQYSLLNQKIMPSTQELNDNNRSRSSILRGLVKNY*

>UUR10_RS02550 Ureaplasma_urealyticum_serovar_10_str_ATCC_33699_NC_011374 ribosome biogenesis GTPase YqeH

MITNKKCKGCGAFLNDEINTIGYTPKLNDTKTNLCQRCFKLIHYNKLEKVHTNLNKNIKNTIDNLDFSNLQIFMVLDILDLEHTIIDELKKYQEQIIFLVNKIDLLPHRYNSELVNENVIKTLVDHGFNNPQIVYVSTHSNTSLKKVMDCIKNATNKKQKSIFLGKSNVGKSSLINALLALNKIKTKLTISSYTNTTINLNKINLLEHQIIDAPGVCFNENILNYVRDEDNKSIMISYGAKAINYQINPQQAIMISGLVGVQYLQGQKTTFTLYVSSQLDIHRCKLENFITNFNNRYLLAKFNYVDQDIEFIDHTIALNKNQKTNICIAGLGLLVINANAEQICIRLPKCVGIKVAKYAII*

>UUR10_RS02185 Ureaplasma_urealyticum_serovar_10_str_ATCC_33699_NC_011374 hypothetical protein

MKKAILISIASLVGVSAIVATSTFLSLSAKTDVYAKSIQASSVNQSLNYTNLSNANVGIKEMLLGTKKINNGNYVLYIGTQSNLDNLDFVYSNQANHINSVNDLQYNDNLNFNGSLSKTINNVKNYADKDIYEHVPQFYSFIDLINSDVFKQKQEYENLIKQNKSSTIKEDQN*ANNAPAEYSFDVNKKYKDKNGKEVYFRNDAQAIKFREILNFLKTYLSKEKLVELSATKTPGIVLFYSQDNLSKGPRVYASSKTYDQSKNERKESYPSGNNVYNSAQPEFGGDLNAAIYSIYGKK*

>UUR10_RS02335 Ureaplasma_urealyticum_serovar_10_str_ATCC_33699_NC_011374 urease accessory protein UreE

LTVFKEILGNITDIENVESYQIENIHLTSDDVLKRVIIISSDQNVEYGIRLEEDKKLRDGDILYKDDYKLVVIRLELSDVLIITARTIGEMAQIAHNLGNRHMPAQFTETQMIVPYDYLVEQYLQDNKALYEREKIKLKEAFRHCSDAK*

>UUR10_RS01015 Ureaplasma_urealyticum_serovar_10_str_ATCC_33699_NC_011374 serine/threonine-protein phosphatase

MNFGFISDIGSQRKHNDDCALVIQNEHQQTLLIVCDGLGGYKGGAAASHITLETIKDNFLATNFNEYDEQQIRK*YIKVIKLAQIEIDRAVLLDKDVYNMGTTVVASIIINDFVYTLNIGDSRAYLLSNNQSSQISRDHNLLQVLHERKVGPEVYEKHEKNLFSLTQFVGRTSNVVLSYDLFVTKLHHNEIIVLTSDGFHNYFELNDLYDKLIVTNQQTNNQILQQLINQAIDNGSNDNLSLAFLIF*

>UUR10_RS02725 Ureaplasma_urealyticum_serovar_10_str_ATCC_33699_NC_011374 rRNA maturation RNase YbeY

MRFLITNEVKSVFNDEIYLQRFEQIANLISIKLNIDKERFFECHFVDEKTIQEINRDYRNKDYITDVISFAFDDGEIITPLLGEMYICYQKVVNQAKEFGHSFERELCFLFTHGLLHLLGYDHIEVEEEKIMFGLQDEILNELNITRNVNGNKNG*

>UUR10_RS00590 Ureaplasma_urealyticum_serovar_10_str_ATCC_33699_NC_011374 suppressor of fused domain protein

MNYKYTAKEKQIIYNYILREYGQVDHIIFLSDEHIRVPIEYDILVIKKDDLQILMTFGLGAFKSHNHIEKTQERAEIFLELPID*DFSKYENM*PVHFLINIVKYSYSNHLTLKWLQTFVNPSYFNKSNKIAGFLDLS*YSENSLECKINDDFFVSFYQILIIDDEELFYAKTNGIRALSKFFDDGKSRIVDLNRKSFVK*

>UUR10_RS02385 Ureaplasma_urealyticum_serovar_10_str_ATCC_33699_NC_011374 hypothetical protein

MGLFSKKNKLNQELSTNQNTVNDEVSHDDKSIYQLYDETIVTKKVKVRCKVCGVFM*VDYTKTIKSLRNLALTNVQQEVITIEEPQVLEDNSDVLLTPVNDQEELVSEEYESAPIESIQEEQIVEDNEPIQDENEEITPPVYEVEEPTLKRTSFTPVAFEFEEPIFENIYSNQKVYAENLFLESMFSEKENLINDDQNDFS*LSKYVSV*NKRDEAELAQMVYDERLLAPEISEFTHNEYFIEEEYEHADPDNHHNKHKAQRHLHGHDLVAKKHEKQLEKKTNEIIDQAIVAIDSMDEEQQTSLKTIISEEEFKANPVLKKT**ETESANKSSK*

>UUR10_RS01085 Ureaplasma_urealyticum_serovar_10_str_ATCC_33699_NC_011374 50S ribosomal protein L35

MAKIRQKTKRAAAKRFSITKNGKLKRKHAYRSHLALGRSTKAKRHLRKDAIMSTSDTKRYTQCL*

>UUR10_RS01330 Ureaplasma_urealyticum_serovar_10_str_ATCC_33699_NC_011374 hypothetical protein

MSTKAFNPKERVTKTVVLSDLDQHQNYSKKHKKMDFKKRVVLSIFLILLTCGAIILLVFVITELFRLSA*

>UUR10_RS03145 Ureaplasma_urealyticum_serovar_10_str_ATCC_33699_NC_011374 30S ribosomal protein S18

MAKVINNRNRKPRKKVCILSAKGIEHVDYKDVELLQRFINNNNKIASRRVTGASARMQRRIANAIKRARFVGLLPYVKE*

>UUR10_RS00665 Ureaplasma_urealyticum_serovar_10_str_ATCC_33699_NC_011374 hypothetical protein

LKINITKGKTIFKRNYKKKHSIYYSTSKIIKDYTLSAVMLGLGILIAYLSHFIRFNFLAFDFSLFTLVYLIYKVKYRFVYLVTILLSLANLLHGSPSWIGTMVLSLNNLFFVSMVILFKKLFIPKRPKFYLNL*VLVLSSFLTTVFNVIMNGILYTPLY*YTFKITPTLNFLEVQKIYEKHPTLFLLNIKSY*LGIIALYTSFNLIKNIIISFISLTILKIFADKKTIITY*

>UUR10_RS00705 Ureaplasma_urealyticum_serovar_10_str_ATCC_33699_NC_011374 F0F1 ATP synthase subunit gamma

MSLDAIKRKISSVQTTAKITNAMKLVATAKLKRQRDRLAAIKEYCHDYYDVIGLLLSVVNDIEFLKIPNAKNRTLYITINSTMGLAGSYNYNVNKLVSKIINEDDITFTIGKKGHDFMRLSNRLHQVNTYLNLNDNDLTFDMSLQIAREALELYSNGEVNKICIIYTKFINAITFEVNNIDVLPFDKTVLTKDNLAETIELAKDNIIFQPNKVELVKKILPTYIATVLYGSLIESKISENASRRNAMDAATKNAKALAEDYKLIYNTLRQGKITREITEIVAGSDD*

>UUR10_RS00805 Ureaplasma_urealyticum_serovar_10_str_ATCC_33699_NC_011374 tryptophan--tRNA ligase

MKRLISGIQPTNNLTLGNYLGAIKNFVDLQNDYEVFLFVADLHSLTPNIFDNTNFFATKRQIIATYLAAGIDPKKTCLFYQSDILAIPLLSHILLCSTSIGELTRMTQFKDKSAKATKMANNTEMIPSGLLTYPALMAADILAFNADVVPVGQDQKQHLELTRTLADRFNKRYGQTFKLPQVYIPKIGAKIMDLLDPSVKMSKSSKNPKGVIFLNDSREQIIKKIKGALTDNLNQVKYDVEQQPSVSNLITIYACLTNLTFAEIETKYNQQNYGVFKNDLANIVADFLENLQQKISY*LNSPELDIMIDNSCERANDVANQNVQLVLKQMQLK*

>UUR10_RS03280 Ureaplasma_urealyticum_serovar_10_str_ATCC_33699_NC_011374 hypothetical protein

MRNNLSDLEIEKLTKTLVSKNKRVVALYGVPENASYKLMNDLISLMDVEIPN*SSISSATEDQCINDIKNFLLSNASLENFKNLPSNSPLIN**KKRRLNTVVKKIEEINSAFADREITSRSKIFLMPVLTAFSTLGTTLAVPLISVTFLRGDAIINL*GKSGYIAFLSLLFSLVIAVIISAFVALFSSLKNNKRNYIVSNMTEGFKRIYDKYFIDGNSEERINAKVTFYSRFLERSKLVVQNNYSFFYDVVDINSEQYPKMLKYFKTLNQLNNTVIFDASGFKYLDERKIFRNIIDPEKTNVVRLDRYKTKTSGRRLMNFIFYQLSIIANVNTRKLLQKFPFFVNSLYRFLDYSEKNTELLTLLLDLKKHASKTQVPLDDESQLFFVDFFTFVVFKALDESGFETLINDLTVYGRPSEITKKNITYNSLKLDYIINRNARNFGQQALLFNLLDYFDETGNKNIFNELGNSNKNMIFSKNHQLSLANEALSKKGFTKREIDLNAH*CDALYSNLHDDEDMFVKVIEIKENIDVLSALDEVFVRAQNENVKNLLIYVFNVKMLYCLIDNEYELVNESII*

>UUR10_RS01600 Ureaplasma_urealyticum_serovar_10_str_ATCC_33699_NC_011374 30S ribosome-binding factor RbfA

MANEVRVARLESLIKDVINNALANEINDKIAKLARVTAVRLSNDLSVAKIFLDAHKRESMPKVLENVNKVSGLLRSKLAAE*TSYKVPELRFVIDETIDYANHIDELFKKIKQQEN*

>UUR10_RS03155 Ureaplasma_urealyticum_serovar_10_str_ATCC_33699_NC_011374 30S ribosomal protein S6

MAKYEIMLVVRGDLDQEQANKVANELKATLKNTEVKENNYEGVQQLAYEINKLKTAYRYVYNFETTDVSLINEFRRLAIINKNVLRHIIINLEKDYGYKATVNAKKVQRNEKRAEVYVRQKEEAERRAAERQAAYEAMKAEREAAGLPVKEFVKGANSKR*

>UUR10_RS00115 Ureaplasma_urealyticum_serovar_10_str_ATCC_33699_NC_011374 iron ABC transporter permease

LLTSFIFLFNIIYTNKTFI*QSFYFQKASSEEVFLLFFYSPIILLVCGVGLVCSGFSLQSTTRNGLAGPSTLGIFPLAALGSILSQLITKSFSNIFIGYAFGIVFSLIALGINFLFIKMTPHKKTFKPILFGFSLGAIITAINVLIANFHSSIVASPIQLLGSVGVYSSIERFYIGVPIVFISTIVLLCYAKKIQILYTSHYLAKSLGININRMY*ITSICSIFIAISTTFLIGGLSLIAIVMPHLSRLLFKRTNYLFQMIAAIFLTCILLQLLGLVVEFFSKFNIVFLVATVLAIPMIVLLK*ELGWKVNKN*

>UUR10_RS01405 Ureaplasma_urealyticum_serovar_10_str_ATCC_33699_NC_011374 TetR/AcrR family transcriptional regulator

MSTRTLKISKVKLKIVDALFNLLKTNAFDKIKISEIIEVANISRPSYYRNFDNKISIITYFIKNIYN*NVKNKLINAKVNKKFDEHGFIKFYEASLQTSLEFKDYFLILDKNNFSGLIVDLTTENGFYAIGNMSNNSIDKYIINHLTGFFCNIRMQ*LKNGAKESVHDMAMFLVSILKDNVIEKLKKYNCHHDNDDEE**

>UUR10_RS01550 Ureaplasma_urealyticum_serovar_10_str_ATCC_33699_NC_011374 tRNA (adenosine(37)-N6)-threonylcarbamoyltransferase complex dimerization subunit type 1 TsaB

MNSLYQLFIDVTSKKCVLAIYKNFKILANIIVETNNNLTDIIVEHIIALLKAVHLKYQDLDAIYLDIGPGSFTGVRVGAIVAKTICTTHNQIKLFINDSLNIIANNKNNVFVHLDAKGNKSYTISIINNIQSDYRIITNEQLQIELKNTSLTIIDANQVDYHNLIYNLKFDNFKLTNILDFDLNYVKKPLS*

>UUR10_RS00055 Ureaplasma_urealyticum_serovar_10_str_ATCC_33699_NC_011374 50S ribosomal protein L7/L12

MSKLTIEQFIAAIKEMSMLELNDLVKAIETEFGVSAAAPVAVAAAPAAAEAPTEVTIKLVEAGANKVGVIKLIREITGLGLMEAKTAAETAGSVIKEDVKTEEANEIKKKFDELGAKVQLV*

>UUR10_RS00920 Ureaplasma_urealyticum_serovar_10_str_ATCC_33699_NC_011374 phosphate ABC transporter permease PstA

MSILNLNSQQTKKIIFNKKIKDKLAKTLVISFA*LFSICFIGLVVFIIIRSIPGFEAYGLKNIF*SKNFNLADYAAGSSV*FPLAITLLVSFGAIIIAAPIGIKTATFIKFRIKNKRLQKFFRVTILALAGIPSVIFGLFAIQSLGPAISFVFHIDTVQNITTSMFMLAFIIIPTIISLTLSTYDGIDMRLIENGIGMGSSYTRSIYKIFKKEARGGIIIAIIIALGRAIGETMAISMILSDQGYQNIFGTGFLQIMHSALRPLGAVISANMFAENGGEGLRGLLYVYGIVLFVAIMILNGLVTYLTRKRSKKTYA*FIKLEKSLAYIVCFIPDQLKILYEKITHRSQYKLHVNNLDNLNNYITDRIQNRKLKRLYTIHKLFFESLAFMVAFAFLA*ISLDILVNGIKAINLPTSTVVAYTKNTTGQATINTLIIIIVAILIGLPFSLFVAIYINEYAKNK*PKKVLLFFIDSFGSTPSIIFGMFGLVIFIEIFGFTSMGNIGKSLLAGALTITLVVLPTFTRSIQQSLKAVPMSIRENAYGLGCSK*ETIVKLVLPQAKKGIISAIVLTIGRIVAETAPLYLTAGLSSAQSITILNPGQTLTTRIYAQLYENNTNIAHNVMYESAFITL*LVIALIIIAHVIVPYFEFIKHDIKKWFKILKNYLRAPYMRDIKVFKPQIYQKHLYLTHNQAKIMGYNESVNKVAKIGLKLYEIRYVDDEQIQKELLKVRG*

>UUR10_RS01765 Ureaplasma_urealyticum_serovar_10_str_ATCC_33699_NC_011374 hypothetical protein

LKTILIVNKSFQKKRIFDTRFLHDSGPIYTNDRFLKIVRESFDAHINKKSKFLFYFME**FQTTNYKLNPEISHTKRSLRS*LIEIFFLFCIIALIVCILAGVARPIIDCVIKSITLSRGQFKGDLIIDGQVIAKGLIPSSASGLVGGSSILNVEQ*ELIPKV*KTYFSSPSVVVLILLVLFFLFFSCLYIYMLKKIRPRTNKMSLEDYLVSRMNLINSFKFILKRKVLFVEGIKEYNHRFIFNMSADYNLMRLMNYIYSGLVELNIIMVVDFESDEKIFELQKIIKQDFDNLDLIILEKEVADKLKSIHQNGCLNSKNPSLINISNKEIDQNQEIQNIEIY*

>UUR10_RS00625 Ureaplasma_urealyticum_serovar_10_str_ATCC_33699_NC_011374 HAD family hydrolase

MTKKLIVFDFDGTIMHTHTTMSLSIIGVLEFYNHHVPSLKEMNDLLGNLSMANIFRKYAKHDLLDIEIETMIAKYYEIYESSLFMIHSYFFDGILELIKKIRSIDNVKLAILSNKKSTLLTTMVDYYNLHHYFDYIYGAEDVDQMKPHPSGLLRLMNNCNVDHKNTLLIGDSLADLKAALNVKCHFLLVN*EPEYQKHKETIANLKPLVVQTIDELETEISQFLR*

>UUR10_RS00695 Ureaplasma_urealyticum_serovar_10_str_ATCC_33699_NC_011374 ATP synthase F1 subunit epsilon

MANLTKLKIVTPYAQNLEKDVYSVELKTSEGRIAVLPDHNPLMSIIENHVAYIRELPNAPRKPLLLLDGIVYVEEHQVRVFSDYFKFLDEIKIDEINSLLNKLKNDLANEEDDKKKLQLKSKIKLNESILIAYKDR*

>UUR10_RS02245 Ureaplasma_urealyticum_serovar_10_str_ATCC_33699_NC_011374 DNA-formamidopyrimidine glycosylase

MPELPEVQTIVDYLNLNVLNLLIKKVIVHLPKILKNKTPAEFENLLVNHKITNIKRLGKYLLFFLDNNLVLSVHLRMEGKFYYQPKDE*FNLAHTHIIIEFENGMQLRYNDTRQFGTFHIYEQESFLDSKELKKIALDPLDANFTPQYLYEKLKKSNKAIKTALLDQSNVSGIGNIYADEILFATKIFPTTLAKDLTIKDYENIAKEAKRILLLSIQNKGTTIHTYKFGNDETGMFQKMLLVHTHAKKPCQTCGTIIQKTKVNGRGTYYCSNCQNQK*

>UUR10_RS01180 Ureaplasma_urealyticum_serovar_10_str_ATCC_33699_NC_011374 50S ribosomal protein L18

MKRINFSRAKQRALRAKRLHVKIRNLQLAANKPVLVITKTNAHI*AQLICYNKNITLASSSSVQLDLQNGNKDNARLVGADIAKKALAQGFKQVIFNKNGAKYHGRIKALADAAREAGLEF*

>UUR10_RS02145 Ureaplasma_urealyticum_serovar_10_str_ATCC_33699_NC_011374 hypothetical protein

MPLITGALGIWNKVLFGTIIANTAISVIGGITNIAMAASTLPKQESETIETIDVDMTTMVGAKTPYANIYYLGS*

>UUR10_RS01220 Ureaplasma_urealyticum_serovar_10_str_ATCC_33699_NC_011374 30S ribosomal protein S13

MARILGVDIPNDKRVVISLTYIFGIGKSTSQKILKLANIDENIRVNDLADEQIAEIRRVALNFVKANGEKLQLEGDLRRTVAMDIKRLMEIGSYRGIRHRRGLPVRGQRTKTNARTRKGPRKTVANKKIETR*

>UUR10_RS01315 Ureaplasma_urealyticum_serovar_10_str_ATCC_33699_NC_011374 HIT family protein

MQDCIFCKILKGEIISKIIDENEFAIAILDIQPASDGHILIIPKKHYRNFSLTDPIYLDGMMRLAKNMTFVLEEVFPNVLGFNYLMNSNSGAGQVVMHTHMHIIPKQNNDRGFVFKAIKEEGDISDIDEIYKKIATKTQKLKKSRLSKHYMV*

>UUR10_RS01000 Ureaplasma_urealyticum_serovar_10_str_ATCC_33699_NC_011374 50S ribosomal protein L21

MFAIFQTGGKQYKVQQGEKIYVEKLDLEVGSKISFDQVIMVEGSVGTPFVKNAVVNATVLKQGKQKKINIIKFKSKKHHLKRQGHRQPYTQLVIDSISVK*

>UUR10_RS01160 Ureaplasma_urealyticum_serovar_10_str_ATCC_33699_NC_011374 50S ribosomal protein L5

MAFLKDLYKNKVAKDLQKEFAYSSVMQIPKIEKVVINAGIGNAVADKKHLEAAISELTLITGQRPVETKAKKSIATFKLRAGQSIGAKVTLRGDRM*AFIETLFNIALPRVRDFKGISNNSFDDQGNYTLGIKEQIIFPQVVYDDVKSVRGFDVTFVTTAKTAQEAKALLVGLGAPFQKVRGDK*

>UUR10_RS01710 Ureaplasma_urealyticum_serovar_10_str_ATCC_33699_NC_011374 (d)CMP kinase

MKKYINVAIDGPSGSGKSTAAKGLANKLGFLYINTGLMYRAYAYFLNENNLDINTNETACIEAIKNARFIFNGDDVKIDDQDVSDILRSNDVAMLASVVAANAKIRNLATNEQRKIASENNVVMDGRDIGSIVLVDADLKFYLNTSIQTRAKRRLAQNKDIEKLDYESIYNDIKERDYRDMTRDIAPLKKAIDAIEIFNDNMNLDQCVAHLYEIYLNKIKKS*

>UUR10_RS02270 Ureaplasma_urealyticum_serovar_10_str_ATCC_33699_NC_011374 tRNA 4-thiouridine(8) synthase ThiI

MKPIIYIKYGELTLKGKNRAQFIKVLVHNIKQMLLEYHELVYQVGYDNLKIINLEKYNLQQVINDLQQVYGIAFICVAYQVNKEINEIQLACNKLVNNTDQTFKIEARRNDKSFIYDSMQIKQICATYLLQNQPTLKVDVHHPQLLINIEIKHDCAIVYGHKIPGAKGLPVGINGKALVLLSGGIDSPVASRLIMKRGISVDFITFITPPHTSQKALDKTIALAKQITLNNHLTKANLYVCNFTKLQEEIAHISKESYRITLMRRYFMRIAKRLAIDIKAGALVTGEALGQVASQTLNSMQTISSVLNDFLVLRPLITYDKQEIISLAKQFNTYELSILPYDDSCSLFAPKNPTTNPNVQTAMKLEQESLVLDAIYELVFTKEITKINLSSK*

>UUR10_RS00505 Ureaplasma_urealyticum_serovar_10_str_ATCC_33699_NC_011374 ABC transporter permease

MNKKMNFNSSFAYVGFLTRHIIVKKTTYILPIITFLITLIVGIIVGCVVNQKSQFLIISYVMIIFNLLMTTIFASLKALNVFKDFSEDGIDILVISKPISRKNIV*SKIFFFILTGVVWSLISFLGLLIFYLISFNFAKDVNYY*LLSYISPFICYLVFGLLTSLLALKLNAKISVLIPLVSFVPLLAVGIVSNIVSQLQSKAFLSSLKQKPTNNIEPFYLNNNLDQYYLINTGFANHEFKQSQNLEVLDAYLKTRNLATF*QVSS*IFPIYQLVDAFNKSDYEPFSDFIKNQENHKVLYANNLASKQFNYRLEKNSDSLPSFNINNDKGFLVPSLLKNDSQNLINNNLNQEVIYAIED*KNQTIEYQKNSYTQLSADDIVGSIK*TIIKEVLNTKLFNEYANNLFKTLDKKASKKQILDLISNSVQKFDFNKQIDENTELFKKDVNKLLIKSKTERQIYLAISLIYYLYFSSNYTDLLNTLLFDQKNHLQYQTQFKINLNNKNYLIGGFKNYLVNQILPQETNDAIDQSKKDKKIRYKYSLEQGGNYLFDYVTQNYSIKREQQIVYKEVYCVI*LVLIFSLLIGTYYGYKRKDYR*

>UUR10_RS00815 Ureaplasma_urealyticum_serovar_10_str_ATCC_33699_NC_011374 NAD(+)/NADH kinase

MKDVKPVYFYDIYCFNPNKCTEDKGVLLLETKLKEYQKVTFLRSEQKPQIVFLLGGDGSFINFVNQQ*KQN*KIVGINYGQLGFYSSYDGINTINIDEIVDESMYANAFLIEVNINNENKFYCLNELSIFSNELASCDISINNTFYEKFRGSGLLFATPSGSTGKNKVAHGPIIFNNQPCFSMLEIFPVNHLKYSSLNAPVVFGKDYQISLTNIKFKRTLNLVVDGNNINFNNKIDFIEVKLIQASLQIHGLNNYKKYIERLRRSFIKEE*

>UUR10_RS01100 Ureaplasma_urealyticum_serovar_10_str_ATCC_33699_NC_011374 50S ribosomal protein L3

MKSLLGTKVGMTQVFTETGKAVAATVIYVEPNKVLAVKTNEKDGYNAIQIGYETVKEKALNKPLLGQFKKANSDPKRHIKEFRDVVAEVGAELTVSEFEPGQLVNAQAYTKGHGFTGSIKRHNFSMGPMGHGAGYPHRYVGSIAKGRGGSQAQRVFKGTKLPGHYGHELVTTKNLLVLDVKANENLILIKGAIPGPKGSIVLLKSAKKVGHIVSDPQVVNYLANKASSSEANK*

>UUR10_RS03075 Ureaplasma_urealyticum_serovar_10_str_ATCC_33699_NC_011374 energy-coupling factor transporter transmembrane protein EcfT

MSANAYVFRRSPIHRLNPAIKFISFILLIAMIFLPLGFFAQMIIGVFILIIFFVAKLPKKTL*NVFKSVIMLFVILLLIN*MTYKDPIAIYNITDQAKVILGDKD*INGPINKNLSFSLIYNDISSTHVQNLVSNIWGGEIKNYISPEIIKKLIDKPDYNVAKFLSENNITVKKLASTFNALNQDVRLNNYYPIYGDAVLRSGKVEVPLSHLSYYMSTNL*KIEGVKYQGLILSGVGDQLGKAETALFYTRSPFALSPVAIQLAIYISIKIFLMITLSSILTATTSSIELTNGLEDLLSPFKILRLPVAEASMMISIALRFIPSLLDESKRILNAQASRGVDFNNGGMLQKLKSLISLVVPLFSIAFKKAEDLANAMEARSYNPRYARTRYRAFPLNLTDYVLFGILCILVGFLISLAVIKFYFTPFGAFEASALFAK*

>UUR10_RS03275 Ureaplasma_urealyticum_serovar_10_str_ATCC_33699_NC_011374 DUF4011 domain-containing protein

MKKAIKQKLMNLVNLNPHDSIILTRLSSSRNIDLFKYIKKEDLISFVNGEISYIELNVGKNLNLFEESLKNSLNIGEFLEILVNYNVSLPDNKINLLQRDFHANKKKVIPLAIEQLLAFRKKFISINRLAKNYFEDTTV*PLYFAFNFLKGKLLPNDAFKSPLTLFKVEIREEADKIFIAKMQDEPIVNEKIQIFLKKGYQDFKVETTELLTNYELSSTIKMIEDMTGQKISVNENEFVPFMNENEAQILDRYTTFEVEPSAMLGLFEPDGGALKADLHKIIEMDVDPFESENEGLNKPVEYYEEKVIKEQAVVEIGRPLNIFQKYAVASSLSQNTLIYGPPGTGKSEVIANIIFNVLLKGKSSLLVSEKRAALDVLTERIGSLSQFALYVYDLTNKETFFEKISNLNDLLGTQ*YREQSRSTKIKEIEPIKFTPEESMFFKNYED*NAELLHLVKKH*SIEDYNDGIYKMDYADYIATKNELGEQIVKE*LTPQQFNDESEKRSTLFEEISAIFNEYNLLKIEDLFSAYLRFTSFIKKYKLTDTYSSTEILKHLKMITNKIQTNDELVTKFLMHSNRITKDIDNYYNFLAEHNLESNSVFMNKSIRDKRIFIDKIGDYLKFRKDVIDKDFSLQSKTTQQLNEIIDICDNFFTKHKKLLVKNE*YDFLVKNKDRISAFLSVYNNASEENKQIIFAEFITNGTIINNSDPSDESTLSLKEIKTRNRDSQEVIELFVDFLNNVEYLAKPKMDQIASYREFINQDVDFLSKLHTLAEIYTPIMQDIIRE*S*LSLPYIKTLYLEPLMLFDLEKVGKIMKHVSTLITHEQFKKLKVVVL*DEITHIIPMFSETKGRLLQDIIVQLRRESSRSARIVGEIVFKKYINNLRNYLTKLPQQEKDEITNALRIASSRS*PSISRYLSKYYNALKRLFPI*VARPDNVASLIPLVENEFDYGIFDEASQMTIERSYPIVYRCKIKVVSGDDKQLKPTSFFINKLVDSDFEIDDFDKVDSLLERAKTS**NEYHLKNHYRSDSKELIEFSNKYIYNNNLEVATRQGAFEKGIDVINVNGV*EKGNPLEAEQTIAILIDN*KKYEKILIVTFNAVQASLVENLLFERMSMFEKGLCDKIENNEIVITNLENVQGNEGDLVILSIAYGPNPEGNLRNNFGPLNAKGGMNRLNVAITRARKKMIVIKSLYGHQIQVSNLNNQNALTFKRFIEYIDRINGELSISDTLESLEQQTYLEFDNDLVKEIYSELTKKLSNKYQIFPN*NIGTKKIDLVIIKKETKEIVKTILLET*KENRSVQIMFEDIDRQYFLEDRGYSTYRIKEYE*YIDKHKIVSRINDSLSSNNNSNKIDYVL*QQNNF*

>UUR10_RS01185 Ureaplasma_urealyticum_serovar_10_str_ATCC_33699_NC_011374 30S ribosomal protein S5

MENNVKKETIVDSEKVEKQQPVTAPVVNKKENTQPKAKTFKRETTTSNFEERVVKIKRISKTTKGGRMMRFSALVVIGDKNGTVGFGMGKSIEVPDAIKKAIKNANNNLIKVKQTKKGSIYHDVNGRHGAAKVMLLPAPEGTGIIAGGPVRAVVELAGFTDIYTKSRGANAPMNVIRATINGLLQQLTPQEIARLRDKSLKEL*

>UUR10_RS01570 Ureaplasma_urealyticum_serovar_10_str_ATCC_33699_NC_011374 hypothetical protein

VKEFSKNKTYNNFKNHKSPLRVVEGIVREIKNNELQIDFVDSYKKGICKFINLTDYN*NTISSRFLINSKHLFLISKFDPTRRVY*LNYKIIHPIEIKNKRRSYPTLSHDRNLRIFLTNLLENEDKEHNNHEIN*

>UUR10_RS02275 Ureaplasma_urealyticum_serovar_10_str_ATCC_33699_NC_011374 hypothetical protein

MPFIKTPNKDFELFDNQIVLSNDYKKRYQRYFAKITGSRLGAILNVGDFANPVKT*AMMVKIYNEPIDPIYAQAGVIIEPKIKDYVESVLKIKYKQYEPAQIGYDVFKDNLIFGGIPDGEPVDENGELLYPNQPMLEIKTTSIDSFAFKTIDYVLVLQKDEFNRPIVKKKGDKRAK*FNSDESQVIISEDYKLQLGLYCYLRKITKGIFAIAFLTSEDYINPQSFDINKNEIILVNYEINLNEFEKVISKAKTWYEEYIIKGISPKLSKDDLE*YKV*VNKYETNNLY*

>UUR10_RS01115 Ureaplasma_urealyticum_serovar_10_str_ATCC_33699_NC_011374 50S ribosomal protein L2

MAVKRIKNHSSGKRQTVVVDYKSILTTSKPEKSLLVTLPKKAGRNNQGKITIRHHGGGHKRKYRIIDFKRNKDNIYGTIKSIEYDPNRTSFISLVVYADGEKRYIIAPKGIKVGDKIISGNENIDILLGNSLPLEFIPEDTLVHNIELSPNAGGQITRSAGASAQILGFDETKKYILVKLNSGEVRKFRKECRATIGTVSNDEHILENLGKAGKSRHLGVRPTVRGSAMNPNDHPHGGGEGRSPVGMDAPRTP*GKRHMGVKTRNNKKSSTSMIVRRRK*

>UUR10_RS02180 Ureaplasma_urealyticum_serovar_10_str_ATCC_33699_NC_011374 tRNA 2-thiouridine(34) synthase MnmA

MEVNTKKRVVIGLSGGVDSSVSALLLKQQGYEVIGLFMAN*DTVANFENNRESDKKHQGCESELDYQDAQAVAQKIGIPLYRVEFIKEY*NNVFEYFLSEYQKNRTPNPDILCNQFIKFDSFLNYAKNELKADYIAMGHYAKVKHTNNLSYLLKATDVNKDQTYFLCNLKQTQLQNALFPIGDLTKQQVRTIAKEYGLVTANKKDSTGICFIGERNFKYFLENYIPNQPGEIVNIVNNQIVGHHMGTMYYTIGQRKGLNLGGMNERMFVCEKDINKKIIYVSPLSLEDQYLISNQALVENMNFIEPYNPQIPISVRFRHRQNLVVVNSFLCIENTNNVLINYEPAKAITPGQYAVFYQNDHCIGGGVIAQTNANHKKINF*

>UUR10_RS03200 Ureaplasma_urealyticum_serovar_10_str_ATCC_33699_NC_011374 ABC transporter ATP-binding protein

MKPKNNRYTYIKYLRSQRAFVQALKPQVLVEPTLNNNVHLNEQQVDLLPQLTNLENTNESVVNELVETKSNEEINIQDVINLEQKINYENNNSQNQDLDNEIFATTLINEQASGINEVSEQKQESPKVSKKHKKQTMAFKVANSRKNFDFEKFEKNVKYKTFKDGTVKKIAAEIDNVRLTFTNPSKPEDRALVLRNTSVQFYEGEVHAIIGESGSGKSVITSLLYGLAGKNANVEEGRILLYNNEVQDFSFKD*EKSRYLGKVISAVFQNPMSTLNPTMKIGKQIMEGMLINGIVKTRKEAYKKSIEYLKLTKINNPEEIMELYPHELSGGMIQRVVIASIVSLHPKILVLDEPTTALDPTVQALVLDVIRELQEKFKMCIIFITHDLGVVASIANYISIMYAGQIIEEGQRDEIL*NPQHPYT*GLIMSMPDVNKGDRLATIKGSVPSRLNEIVGDAFAIRNDYALTRDFEHEPEMYYVSETHRVKSALLDERAEEYTPPQIILDK*NSFKQRQENKNNQ*

>UUR10_RS00435 Ureaplasma_urealyticum_serovar_10_str_ATCC_33699_NC_011374 CinA family protein

MNVALELINLLKEKRLKLSVCESASCGALSSSIGEVPGASSVFVGGFISYSNEVKIQIVGVSEKTILKYGAVSEQTAKEMCLQTNQKFNTDIAISITGNAGPQGSENKEVGLFYIGIAIKDFAIVKKVILNSSERTFNRFSIA*EAISYLIELIKK*

>UUR10_RS01450 Ureaplasma_urealyticum_serovar_10_str_ATCC_33699_NC_011374 hypothetical protein

MQYITINNAKTGKILISKPNIKKFIEQKFNLIVNKKFVIKSIDITQYDESLVDISIIIALFDEHRKVDLDEVRDVQNHLASFIYSNLGVDTKSVNIGIDL*

>UUR10_RS02190 Ureaplasma_urealyticum_serovar_10_str_ATCC_33699_NC_011374 transposase

MDKKNIFTILEKLIKKYEYLYKPILDDYISNFTRTFLEYILTIEMKYHLNYERFERYLSNSSRNYRNGFSKKQLHMNDLKILVNIPRDRNGAFESKIISKYQVDISRFEYRLLSLGINNLQLNEYEKILEDFYDIYNDKTIDKLRNEIALKVYDYLENKKQELNNIDVDQIFIDGFSLSTNRYLTIVITKSLDKQLTILGF*VINNLQNK*WNDIIAKIAMHHPQVISYKILFKNDDELLMSLKNHSSNDFERARFIFINKRILK*

>UUR10_RS00025 Ureaplasma_urealyticum_serovar_10_str_ATCC_33699_NC_011374 L-threonylcarbamoyladenylate synthase

MKIYRITNLNAIYDALVANKCVLIPTDTIIGLLAKNQDVIYEIKRRDRNKKIVRFVADYKLLGDLTVEQEQFLDLF*PGSVTVIKNGVSYRMPNSPYILKLIQKLGPLYCSSANISGEEPVKNHNEAIFKFGANSKLIYVEAQQQIGVPSTIVDIDK*EYVRRGANIEMVDMFIKELKYNNTKEKE*

>UUR10_RS00350 Ureaplasma_urealyticum_serovar_10_str_ATCC_33699_NC_011374 phosphotransacetylase

MLNVVNKLKIKLENLTKKPLKVLFIEQSLEIDKAKQILSQNSLINIVDLASFKTPEIINECENIWYELRKSKGESQKDAQLAISNDLNFAVCLSYLKKIDILIVGANLSSKTCFSSILKILKNPNGSIACSTMAMASEQELIFMSDCALNLTLNKDQMIDVAKHSYTIAKKYFEVLNNHAAYISYLSQNDVLYQNNDFDFIGPMQFDAVVDLRVRNKKIINSHQNHINHYVFDNISIANTIFKIYQTKLHYLTIGSYISNILTKVSVISRSASVDEIVATTYLLCVAFLVDLF*

>UUR10_RS02300 Ureaplasma_urealyticum_serovar_10_str_ATCC_33699_NC_011374 DUF3196 family protein

MNAIIIPKNILIKKVVHIILKTKREVVKIMNNDYKGHIKKYFDDILKQAKQVLQKRDYGYAYDLISNEFNNPLIDLKTLQEFEDFALEIKKSAELDFIDENEAKLAKTEFYHKIHDPKTTYVSLAYLETFLMRFINEIDQLDIAFLNNLLSNKTINGSTKLDILDLLAVNNIDQNFDFYNKYLKQSKSINPTNPSEHHLMVVQIQNYLNNDLAKNPSLLNLANKLLMMYITYHFPFAFTHDPNIIAKTIIDYTKSAMSDFECEYNQQQKDIVACINKILEEEQE*

>UUR10_RS00790 Ureaplasma_urealyticum_serovar_10_str_ATCC_33699_NC_011374 hypothetical protein

LNYQTVAFEQVPSQKSEPKEEKQTKAVDYEKAIKDEDYVKQITLSNLEKYISNEYQYLNPNNKTLDYKNHNFVQKFLDEIDVLAQKLESNDLNLEDKQIYEKIILGLR*LLAEDNNETKNKALALFKDYFTTIMKATKVNDKVHYDPSFINEFYGSMSNLFEEYNLEPSVEIESNKIRIIKEDKDVIKDKTVTATFNLYNGFD*PTLLKDVVFKEYKKDSEGKVELKDGKPVETDKIIEDPVVMRLQIAEYPKD*KTSEKDPQNYSYVDFKKSEVLKSPKLVFTKVFNLDKEQKYHVKNVMFSHKLIPNLLKAFNIENKGNNTQNELSAKIKSNLTLVDEKTQTNLNTNYIPLSLTETNNKK*

>UUR10_RS01125 Ureaplasma_urealyticum_serovar_10_str_ATCC_33699_NC_011374 50S ribosomal protein L22

MTNKVIQRNIHISHRKASLVIDLVRNKPVHEAIRILSNTPKKFAPIVLKLLNSAISNVQHNSKDMDPSKLYIYKIVANQGPTMKRTLPRAKGSADQLFKRTTHLEIVLSDDVNEREKELAAIKAKKSKKPLVVEPVAKVETKKVAKPSKVETKPVEKDENVDPELLKREQQVLKVVEKTASQKEEETTETIMISTSPKNAQVLFDDLEKNVIFYKTTPVNKVLRVLVYVTSPTKKVVGEFDLESVEIGAISSI*RKYNKQSVISKKEYDAYYEGKDKAHALVSKKAYKYRNPKDLSEYNMTKGPSGFQYLK*

>UUR10_RS03095 Ureaplasma_urealyticum_serovar_10_str_ATCC_33699_NC_011374 50S ribosomal protein L11

VAPKKKEVTRIAKLNLIGGQAKPGPALASVGINMAEFTKSFNDKTKDQNGKVIPVIITAYKDKSFDYVVKTTPVTYLLKDAAKIKSGAKDPKKQVVATISKEQALEIARYKLVDMTAYDEEAALRMIAGSAKQMGIAIEGVSAYKEKKGN*

>UUR10_RS00090 Ureaplasma_urealyticum_serovar_10_str_ATCC_33699_NC_011374 tRNA uridine-5-carboxymethylaminomethyl(34) synthesis GTPase MnmE

MSTIVALATAPMNCAIHIIRISGPQAFEMINKISTTKIKKETFKIWYTTLKDNDQVLDEVLVNTFVGPKTFTGEDLVEINCHGGVIVANLIIKILIKYGCQPAQRGEFSRRALLNKKMDLSKIEAINNLVNAKNELSVKGVIGALLGRVSQSISDFKHELFMIIGQIEVNIDYPEYDDVEQVDAINLKQRLLVLNEKIKKIIDQSKKFLPINKGIKVLIIGKPNVGKSTLLNALCNEQKAIVTDIPGTTRDVIESSINIDNITLNILDTAGIHSTNDFVENLGINKAKELINKVDLVLYLVPANNQQDLELYDLIKDQKHLLVYTKKDLIDQYSDDQIYINAKDNDIQALIDKIKELFYVQEFDNANIDVLQSQRQIGILENVNYLIDNAITNLEKGDTVDLVVADLEFCNLRLNELLGIGSEYDFLDDLFKNFCVGK*

>UUR10_RS02150 Ureaplasma_urealyticum_serovar_10_str_ATCC_33699_NC_011374 hypothetical protein

MQKMSNNFKQEYYGGFAASNSFNYM*NNALISSMLGQTVVGFFGMISQLVYAFNPQPRFHAHQMVDNSAARTYARIAQKPMNSTISLGSPFF*

>UUR10_RS01135 Ureaplasma_urealyticum_serovar_10_str_ATCC_33699_NC_011374 50S ribosomal protein L16

MLQPKRTKFRKPHKVSYEGKAKGNKQVDFGEFGLMALEGA*IDARQIESARIAISKRLLKTGKM*IRIFPHMSLTKKPLEVRMGSGKGSPEK*VAVVKAGTVMFEIANVSEELMREALRAAGNKLPIKVKIVKKGEAN*

>UUR10_RS03065 Ureaplasma_urealyticum_serovar_10_str_ATCC_33699_NC_011374 DegV family protein

MKKSFLIMTDSSTTLDRE*AKNNDVMILPLSILRSDHTLIVDDGIESKPERIYQDIDNGYSFQTSCTPYGVLIEAIEQKLQEYEKIIFIGISSGFSSQFNNAKNLEKEYENRLFAVDTEDFGYSLEDLVYKIKSMLANNISFDSVIKMIDEHHNYTSSFLACENITGLVRSGRIPKIIGTMLKLSKVTPIIKAE*KNHRAGMALNIRSAPHKILEGISHVFDDQLNDQTIEKVCILQAGLSNERIEELKKAVIDHFHINEEKIVVRSGPPIFLVYV*KGALGIQVMANIPKKHVEKKH*

>UUR10_RS00175 Ureaplasma_urealyticum_serovar_10_str_ATCC_33699_NC_011374 SocA family protein

MKEITPLIVAN*FLTKESMTLKKVQKLVYYAYS*YLILMNEKVDDLKNKLFDEKIKA*VHGPAIPMLYNEFKEHKYNSIPKINDFNEQEYFNLDTIDILNQV*DEYGHYSANQLESITHQEDP*IKARKGFGPLDSCNAVISDKEIFSYYIKQMKIN*

>UUR10_RS00300 Ureaplasma_urealyticum_serovar_10_str_ATCC_33699_NC_011374 preprotein translocase subunit SecG

MALTIVLILFSVLALVIGLLLSRTSPSGGLSSLNGQDLEIFKKTKDRG*IKGLQVLMFLLTIVMILIIIFYRVS*

>UUR10_RS01035 Ureaplasma_urealyticum_serovar_10_str_ATCC_33699_NC_011374 ammonium transporter

VST*GLNKDIYFSTHELT*VPADASQSTLDAIGFNPTNTLLVALAIAFVLLMTPGLALFYGGLVRRKSTLTIINQCVASLGITTLI*IFGGFSLAFGPSVGKGIIGDISTYFAFRNLLFADG*SGGAVLVFANFTNGVPLILFFAYQLAFAIITPPLMVGAFADRMKFKNYLVFLVL*QYLIYIPFAH*I*GQGFLAAAGVIDYAGGIVIHTTAGFGALAASFMLGKRTMLVTDKNRANNLPMVVLGATLLFFG*FGFNVGGAGFVKDSSNHISQAISS*LNTIIALAIGMVG*ITLETIVNKNHKPTTVGLVTGAIAGLATITPTAGFVPI*ASVPIAIMGVLVCYSIAKLLHHFHFDDSLEVLPVHGMGGVVGSLLIGAFATDTVANGIKYNAVGLDSSGNPVYGLLFGLQLGAVALAIV*AFAFTCLIIYISKPRLSAREQLGHIDYINHGEDAYKFDFLIPSEDEMASYEGFDQV*EQKVSGCKKADLASKLVPVFMEHESDCT*QSKSRECTKSK*

>UUR10_RS02210 Ureaplasma_urealyticum_serovar_10_str_ATCC_33699_NC_011374 molecular chaperone DnaJ

MAKRDYYEVLGVSKSASPEEIKTAFRKLAKEHHPDRNKSADDTVFKEINEAYEVLSDPKKRAQYDQFGHDGPQGFAGAGGFSGFSDGFGGVDFDINDIFGSFFKNGASSRSSSSQYETYDIHLRLHLEFIEAIKGVSKNISYDRKITCNKCQGTGAKDPKDVKTCTKCHGRGTTIENVHSLFGTIQQEVECHECEGTGKVANSKCEQCYGKKVINERVNLTVEIPAGTQDNEKLVVSKKGNIINNQEFDLYLHISVKPSKYFAFDGLDIYSETYVDPIKAIVGGVIEVVTTSGIKTIEIPPNTPEGKKFRISGAGIVNKKPNIFSKKNGDFYTTIRYAKPLELTKEEIAYLKNISARTNQSVEYYKNKLLKEVNK*

>UUR10_RS03105 Ureaplasma_urealyticum_serovar_10_str_ATCC_33699_NC_011374 Asp-tRNA(Asn)/Glu-tRNA(Gln) amidotransferase subunit GatB

MQNFEVIIGIEVHTALNTKTKMFSNTPTSHKSMANTLINEIDLALPGTLPSVNQEVVHKGLFLANALHMHTNHQFIAFDRKHYYYLDLPKGYQITQNYFPIGQNGYIQITDENNNPKKIRIKQIHLEEDTAKQTSVNNQVYLDYNRAG*PLIEIVSEADLRSAQETVLFLEELRKILLFNDISDAKMEDGSLRVDVNISIRPRGAKSFGTKVEIKNINSISNVAKAINYEYNRQLNLILLNQSVEQQTRRFDDSTNTTVFMRSKNDAINYRYIRELNIAPIYLSDEYVSQLLSTKPYSINDLRQELLQKGLVSSAIEQLLGDGPLFKAFKYVNKIVNNPSSVYK*LCLEFIGLINKNTQIIEDISMELLQKIGAMIVLFDQTLINGKQTKTILEKIYLTNKDPQTLIKELGFEQITDENEITNL*NQILANNQEMLLQYEERPDRVEKFFMGEMMKLTKAQANPTISFNILKKILQK*

>UUR10_RS00555 Ureaplasma_urealyticum_serovar_10_str_ATCC_33699_NC_011374 serine--tRNA ligase

MFDINLIRKDIVVTKEKMLNKKVSSDLFDQIFGLDVLVRNLMQQEQNLNAKKNQLSKEIGILAKNKDPKLQQTLDLVNSIKSELQDISLTLSNKQDELNKLLLVIPNMPDDSVPIGNDENDNVEIKKVFEPRKFDFSPLAH*DLAAKNKLIDFDKSTKITGSRFIIYTNFGARLYRALQQFCLDMNVKAGFNEI*APVIVNQESLIGSGNLPKFVDDLFKLENSNYYLSPTAEVQLTNLHRNEILKASDLPLYYTALTPCFRSEAGSAGRDVRGVIRQHQFHKVELVKLCKPEDSFKELESMTRQAESILEALELPYRRIALCTGDLGFSSAKTYDLEVWLPSYNAYKEISSCSNCTNFQARRAKIRYKETVDAPTELVHTLNGSSLAIDRL*AAVVENYQQEDGSITIPKALEKYIY*

>UUR10_RS03040 Ureaplasma_urealyticum_serovar_10_str_ATCC_33699_NC_011374 cytidine deaminase

MKYNDIYKGLIELLKKSYSPYSNYPVAAYVDTDIGLIPGVNVENGSLGLTSCAERNAIFNAITNGAKVFKTVYVITKNNGDIGSPCGACRQVVSEFLKKDAKVVVFNNDGTYKEYSVEQLLPFG*DPNVSL*

>UUR10_RS01485 Ureaplasma_urealyticum_serovar_10_str_ATCC_33699_NC_011374 transcription elongation factor GreA

MAKYTISKHRLEELQLELREILDVK*PAITKQLQDAREQGDLSENADYDAAKNEQAALKKRKDEIEEILENYELIEDVMRSTDEVSIGSTIEIYNYQKDHKEVITLVGSMDSDPFANKISMDTPLGKAVVKQKEGSEVTVHTLALPYKVKIIKIID*

>UUR10_RS01745 Ureaplasma_urealyticum_serovar_10_str_ATCC_33699_NC_011374 RNA polymerase sigma factor

MSTKQNEPLFENLEDLKQKVKLSFTEEFSFSYALSEREAGIFESRNLKGADASNPEEILLNVVLDVSKRKRSRNEIKFNKLQNYFIHMNLRDEHFSEIVDVLENIGIRVPDYELVMQSKSKSTAKKKDEYGIDDTLEISTSKIGFSSTTTEKVDDGIKAYLGVLGESKMLRSDEETEYAKMVISNDPALIKIGKNQLYTSNMRLVTSIAKKYLNRGLDLEDLIQEGSSGLLKAIDKFDHEKGHKFSTYAT**IRQSITRAIADQARQIRIPVHMVETINKLTKAERSLIQELGRDPTAEEIAQAMNKASQAKNQKEQLITAQKVVEIKKLNVDPVSLDKQIGHDEESQFSDFISDDEIISPEKYTEKKALNDQINEMFEKVLNDNEQRVIKMRYGLLPFERPYTLEEVGEHLGVTRERARQIESKAIRKLKHPSKTAKLRSFIGESEN*

>UUR10_RS00400 Ureaplasma_urealyticum_serovar_10_str_ATCC_33699_NC_011374 HPr kinase/phosphorylase

MEIRGKLFVSQVVRKFNLNVVANSDYIDREISTTGITRVGFELAGEILFKEI*NIVYFGSKESNYFSKFSETIISKKLGKILDLNPPLIIFGKNFKHAGILLKLAERYKIPIVEVKYSFYELNFTINTYISQKLSHQSLVHGTLLSIYGIGVILMGESGVGKSELAIELVKKGHIFVGDDAILVNRIGGNLYGRAEDSTKDFIEIRGLGIMNFSRSFGIERMIESTKIEIVIELIKAAKHEKIKFERFGREIQHKEFLETKIAYYYIPVIEGRSISDIIETAITDYKLKTSGYNSAEEFILQIDKKGN*

>UUR10_RS00980 Ureaplasma_urealyticum_serovar_10_str_ATCC_33699_NC_011374 50S ribosomal protein L28

MARRDQLTGKGPLSGNTRSHAMNHSKRR*NVNLQKATIKTENGSQRVLVSAKTLKTLKKHNLLA*

>UUR10_RS01700 Ureaplasma_urealyticum_serovar_10_str_ATCC_33699_NC_011374 YbhB/YbcL family Raf kinase inhibitor-like protein

MVEDKFLKQIKEGEQIKIEIKGLDEFNNFDEQSYGLNNISPQMR*SPVLEAASYAVAIIDYEAVGGAPFVH*YALNIFEPQLELNVANLGYANLYQGENSTSKHFTHSTVDANYQTDVANNYFPPCPPNKAHKYEIKIYAVHHKMSTNRNEILYLDDFEAKLNEAKIIAMGSTYVYAPQISFENNELVVGSLEHKHPYLFTDAKNEYHVIEDVIVDDIEKNVYDETQLNNFQDLEQAKIKIIDSSDALSYAVIISSNHTFKKYGRPIINYAHIVEKKSNKRIKFNNSYKLNHAFSYISPSKYEHLGNELLILKDNDANIESGLFTIHVFGLNKKINQLNKVENPTDSIIDLFEMISGNVISYKAKFFKL*

>UUR10_RS00670 Ureaplasma_urealyticum_serovar_10_str_ATCC_33699_NC_011374 cysteine--tRNA ligase

MKLYDSYSNQLVEINDELISIYNCGPTVYNHIHIGNARPLITMDVLYRFLKKHNIKTKYVLNITDIDDKIINYALANNLKELEVSEYYFNEYLKIKKALNTLEMINPKVSTHMDKIIDYIQKLIDKQAGYFIGDDVYFDTKKALNYGQLSKRDLENDIVGMRIESAANKHNPNDFIL*KKTNKGIM*NTP*GIGRPGWHSECSCLINTYIGEQVSIHGGGIDLKFPHHENENAQNQVLYNKNLAKV*MHFGLVNINNEKMSKSLNNFILVKDLLAEYDYQVVRWFFYQADYKQPIKFSHEIMKQNEKEILKIKNAIYNAKNYLYFNHQLKSLTQIDHFGFFDERINDDLDFVGIVDLIHISVKKINILIKKNKDMNELKLNLTQLLYMLDILGINFVDLHNDENLALLNT*KNYVDKKDYVKADELRKQLINIGIL*

>UUR10_RS00825 Ureaplasma_urealyticum_serovar_10_str_ATCC_33699_NC_011374 hypothetical protein

MLIKPAKRSEKIYTIISIKDSVVTLKNDYQDEKEIEIYEFDLQNIIPEIGKFINLIKYDQQGCSVFEEYDQNA*

>UUR10_RS01530 Ureaplasma_urealyticum_serovar_10_str_ATCC_33699_NC_011374 hypothetical protein

MDIVIKKNCNFIKNENYLYSFYTPLIGIQATAMYS*FVNRENIFNKKGIINLTREYLLNELNISINTYNNLITKLVNVDLIKIYCIENNQQQIIEVFRPLNFNEFSNIEKFNEELKNNVSNDHYKILNLLNKENSINNNRLIDLSTQFKTNYSNSFENKLDKIKKIILDEYQLSLQINESVKLSILNFFKKYKFSVNEMANLIKNNLINNIDFLTIDLNKLELSIISNQQVENNKTKLQYQNIERDSSIFNENADLNLFKNVINHYNYFEPSDFLKHIRKEKLSVEDKEAIILLTYDKTLNNAIVNVILDFVLFKNLGRLNIKYLKCIKETVLGINIKNEYEMIKFFQNKKMNQKQLVFNNDDNTNTFKNTDFVEVKFGDL*

>UUR10_RS02075 Ureaplasma_urealyticum_serovar_10_str_ATCC_33699_NC_011374 excinuclease ABC subunit UvrC

MNDLLKTKLKLIPHKPGCYL*KDEFDQIIYIGKAKDLYNRTHSYFNGPKDNKTSKLVSNIKDLEYIVVNNVNEALILENNLIKTHRPKYNILLKDGSNYPYIMITNEQYPRLKYVRTYDKNKGIYFGPLADSTNKYQLFNLLNSIFPFNKCNHQPHQKCIYYDLHRCINQVQPQTYEKAIAEVKEIFKGNLDHILAILENKEQHAVAKLDFENAQKYAEQQKALTYIINSGLVQLDNNESFDVVGFYEKNNYLVIIIFNYVKGKLLNKSADTFAIYDHEINELITSFLMQYYSQNRISTKIIVSLDDDNLLALSQRFKTKFINAQTKFHKQILKLAFDNAILYFDSNIKSVINKQNELDEALNQLKQILKLPDLSMIECFDNSNINLSLPIAGMIVYQNGKLNNKLNRKYNLMTTKNASDYHFMIEVITRRYQRLVSQHQKLPNLIVVDGGKLQVNAALYALEQLQISIPLIGLKKDQKHKTNAIVLANGDEIILDRKSVLYKFLANMQNDVHNYAISFLRNKHTKSIFNSLLNDVQGLGKKRLNELLNYYDSINDLKSASDQELLQFLPKNVLVNLREKLNKI*

>UUR10_RS00015 Ureaplasma_urealyticum_serovar_10_str_ATCC_33699_NC_011374 peptide chain release factor 1

MEYNKKLYEAIERVAVKNKTLKQELETITSDFKKIKEINIQLKKTTKIAEAFAKYKQKIDAGIVAEELLNTEKDLELVELAQMDLDDAKASIPVIENELKIMLLPTDPNDDKNVIVEMRPAAGGDESSIFVGNLFDTYRAYTESNN*KMKIIEMTPNAVGFSFISFMISGEEVYSRMKFESGVHRVQRVPATESKGRVHTSTITVAVLPEQDEVDVVINPSDLRIDTYRASGAGGQHVNRTESAVRITHIPTGVVAACQEGKSQIENRETAMKMLRAKL*EAAQEQQNAEFANLRKNQVGTGDRSEKIRTYNYPQNRVTDHRISLTLNKLDQIMMGELDEIIDALITDEQTNLMANLGI*

>UUR10_RS00475 Ureaplasma_urealyticum_serovar_10_str_ATCC_33699_NC_011374 deoxynucleoside kinase

MSDKKLDFLTPSFKKNRISNSIAIGGMIAFGKSTLAQALHEKYQPSNVVYEMVEGDKLMDLLLAKMYERENNVLFGSLFQLYFVLNRFANYKNNCNRENLTIFDRSIFED*LFAHANIDKPSVFSYYDGL*QGVCKELIYEHGVPKLYVILDGD*ELFKERIFKRNRKVEIDNFSINETYFKRLLEMYKNYMVNVCKDFGIDYIVLDARNSVEHNVNEVTKRLQELKNEQNK*

>UUR10_RS03180 Ureaplasma_urealyticum_serovar_10_str_ATCC_33699_NC_011374 hypothetical protein

MDLIFKRLKFVSIQN*ALLLFSVALFICVGA*IGHELVRHNLFDAHTLSHEKELLNMLSAKDLKNYLTLKEEIQLSIGFFGLFLVL*TLTYLISLTVNIRILVKYLNDFNENKTKVKNALIISLIPFIHYASAIFLTIVYDKVFN*NRKKSHTLQEEYISAYA*

>UUR10_RS03535 Ureaplasma_urealyticum_serovar_10_str_ATCC_33699_NC_011374 50S ribosomal protein L33

MVKKIILVCETCMSRNYQTTRNKFATSRLELNKYCKKCNMKTLHKETR*

>UUR10_RS02265 Ureaplasma_urealyticum_serovar_10_str_ATCC_33699_NC_011374 bifunctional oligoribonuclease/PAP phosphatase NrnA

MQKDLLNKLIEQTYGFSKISIFVHTNPDCDALGSAFALARILKLNTFGTRVKIVGINTLNPNDFKNFFTFDKNEVEDEFIEGSLAFIVDTANQERVLSQKHTLAKKTILVDHHVKTVSYTDLTYINDQSIATCEMLAYSLMHTNLNFDVKTLNYLLLGLTTDSNRLMYDKVSDITYEIMA*FFKNNVKHYQIYQQLYERNLDDILFDNELIKTIKTHKQIAYLNIDKS*NQKYNFTR*GDKVYLLSNIKNYPI*FVVYFDETTNTYKVSLRSNKYKVRLVANQFNGGGHDLAAGCSLANIDQLNDLLKALELLIKNQEVVD*

>UUR10_RS02390 Ureaplasma_urealyticum_serovar_10_str_ATCC_33699_NC_011374 proline--tRNA ligase

MAKKLEKIITRNENFAD*YTSIVNNAKLIQYTDIKGMMVFQPNA*AI*EAIKNQIDLEFKKHGVRNLAMPTLIPLSEFQKEKDHIEGFAPELFMVNQIGDKKLDNPYAIRPTSEILFCNYFKNIVNSYNDLPIKNNQ*CSVMRAEKTTRPFLRNAEFH*QELHAIFASEHEADEFAKTILDVYTDFVQNYLCIPVIKGLKTP*ERFAGAQKTYTIEAMMQDGQALQSATSHYLGQFFAKAYDIKFQGQDNQMHYVHQMSAGLSTRIIGALIMVHADDQGLILPPDIAFNQIAILSIFANKNPQLLTISEQIRNELSDYRLFEDHSDKGVGYKLAQQEIEGTPICILVGVKELANQQVVLVRRDTHEKINVNLIDLKSTIKKLLLDIKTNIYQKAKKQLDESIVFVNSIEELKQVIAQNKMAKAFFDGSKEDDEQIKLLTNASTRCIFDETQSGQCFYTNKKTNKLTLFARAY*

>UUR10_RS00265 Ureaplasma_urealyticum_serovar_10_str_ATCC_33699_NC_011374 hypothetical protein

MQLKDLINKKKNLNNINLKVSNERNIFLINIMKLNQRLTFFSKNAFEIKESILSLKRIYNIKHDMLRHEERKIFKFLNKINDRVLWIYLTEEQKYSTDSYSRYEQKILETIKSNRDDFILIGQGAIEFGKNHNLNILQTFNDSNIKNLTTQLTKMIMILYTFDNYKKVNFVINSNKNYDGHFTILPMNEFSFDKFINLEKCDSNIIDFQKVKIYPNLNEFINVQINVFLVNIINTLITESSFYKTKNGLVATNNILKELDDNLSKIQRKITRVKTELQIEEINLLARQNMNEDDNDNDGGVYES*

>UUR10_RS02340 Ureaplasma_urealyticum_serovar_10_str_ATCC_33699_NC_011374 urease subunit alpha

MFKISRKNYSDLYGITTGDSVRLGDTNL*VKVEKDLTTYGEESVFGGGKTLREGMGMNSTMKLDDKLGNAEVMDLVITNALILDYTGIYKADIGIKNGKIASIGKSGNPHLTDGVDMVVGISTEVSAGEGKIYTAGGLDTHVH*LEPEIVPVALDGGITTVIAGGTGMNDGTKATTVSPGKF*VKSALQAADGLPINAGFLAKGQGMEDPIFEQIVAGACGLKIHED*GATGNAIDLALTVAEKTDVAVAIHTDTLNEAGFVEHTIAAMKGRTIHAYHTEGAGGGHAPDILESVKYAHILPASTNPTIPYTVNTIAEHLDMLMVCHHLNPKVPEDVAFADSRIRSQTIAAEDLLHDMGAISIMSSDTLAMGRIGEVVTRS*QMAHKMKAQFGALKGDSEFNDNNRVKRYVAKYTINPAIAHGIDSYVGSIEVGKLADIVA*EPKFFGAKPYYVVKMGVIARCVAGDPNASIPTCEPVIMRDQFGTYGRSLTSTSVSFVSKIGLENGIKEEYKLEKELLPVKNCRSINKKSMK*NSATPNLEVDPQTFDAAVDYNDLEN*LEQPAAELAKKLKKTANGKYVLDAEPLTEAPLAQRYFLF*

>UUR10_RS01350 Ureaplasma_urealyticum_serovar_10_str_ATCC_33699_NC_011374 arginine--tRNA ligase

MMITQKISEQLSKALEKMGIFETKVLVDKTKNIKFGDFYTNVAMTLSKRVNQSPLVVAKEIINNLDQDLFFKVNLQPPGFLNFTLKAKDHEDLLTQIYDQKDLFGQFAKKNITYNVEYVSANPTGYLHIAHAANAIYGDILANLLKIYGYDVKTEY*INDAGNQIDKLAMSVLVRYLQLQNINIELPTDAYHGQEIYLVAQALYEIYKDQFINVRLNEKEEIDDVIVNEQIKKFAVSYLLDEIKKDLASINTYIDTYTSEN*IRSSGRILEVLSKIKQHTYTLDGAL*LRTTAFGDDKDRVLIKSDGSYTYFTPDIAYHDYKFSKDNTTKLIDV*GTDHLGYIARLKAAMSALGYDPNNLEIVCAQVMKLVKNNEEFKLSKRSGQSLTIKDLVEIIGKDALR*FLGSSSMNSHVVIDVDIALSKNNNNPLYYVQYAHARANQVLNKQVYEFDFKTDLLIETRERELLNQLHFYKQTIANAANNREPHRISNYLYDLAQIFHNYYANIKINDENNKALSAQRYTLV*CVKQVLANGLAIMKITPYDQMY*

>UUR10_RS01540 Ureaplasma_urealyticum_serovar_10_str_ATCC_33699_NC_011374 30S ribosomal protein S20

MANIVSNEKTYRHTQKVRKENHAKMSKLRTIVKKTRSSNEQAQLNEAYKVIDTTASKGVIHKNKANRLKSRTAKAFKANLQVVA*

>UUR10_RS01630 Ureaplasma_urealyticum_serovar_10_str_ATCC_33699_NC_011374 hypothetical protein

MEKTIIQLEFNEEELASIKKIKEKMDPNNNLELNVFLKDLIKDLARDYLNFSNQSFENIAKQMNDLKDLIGNMANDPSSFDFSSMMNEFQKYNKSQKDDEQEQTTKSDKENKSTTKPTKKS*

>UUR10_RS03050 Ureaplasma_urealyticum_serovar_10_str_ATCC_33699_NC_011374 aminopeptidase P family protein

MSDFKLQQVLKTIKEHEAQGVDGMILFCPYNRY*FLEFASSDGFVFINKDGKAIYLVDARYYTAASEAVKNAKVILLARTPQKSTFDLLKDAMVELNITNALVEADYVTLNVHEMLQKLVRKTTLFTSAALRAIKTEKELEYLQKAADIAALTCN*IREQDIIGRTELEVAMLVSKHMLELGGELNSFDPIIASGPNGGSPHHHPGNRVIEDGDMVTVDIGCTYKGYCSDITRSFIVGNKANPQMQEIYDKVLESQTAGIDLVSTKVTGQEVDKLCRDIIDNSKFNGYFTHGTGHGVGLEVHELPNTNAGNPNKLPLNAVVTVEPGIYIPNVGGVRIEDTVVVKDGQALVLTRLAYK*

>UUR10_RS00065 Ureaplasma_urealyticum_serovar_10_str_ATCC_33699_NC_011374 ABC transporter permease

MPSLSLINDASIYAAILILGALSGFFCERVGIANISINGQMIIGALVFTLFSTIIYKYMGTKTETEYTFIVCLIIAGILTIPTSLLFGFLTIKLKTNQVIAGTAINLLASGVATFVTYPLGDKIANKTSLSSDYLGLMKIGDGINTIYIGSILILIAVVLITIGLIIMMKKTPFGLRLYAIGENPNAADAQGINVYKYQ*IAVSISGFIAGIGGGLYMYASRSPFGGEVGGIGFLALAILIAGA*RIPLIVVVSIAFAVITKTFSQIDKIPQEIGKLIPYVITLIALISFSKYSVAPKNVGIPFDKSKR*

>UUR10_RS00125 Ureaplasma_urealyticum_serovar_10_str_ATCC_33699_NC_011374 iron ABC transporter permease

MHNYTFRFKLFKQIQNQTTKKLKPLILLVAAIILIATIFSI*I*NISYANFYDQTLNKYVYFSLNESLKLIFSGQFLDSSNIKYLNLQATF*ASIASILSGISLAIAGCVTQALTRNPLADSSTLGIVQSSVFMIVIAFSHNIISFGGLFGFAIVGGIVASIILLILVFATRNKLSYVKITLAGLAIGVFFNTIAYFVRIQSAKGSSINFKYVLGGSENIYPTHIDPFLTL*VSAVLILIGVIIACVLSHKLTLLEIGDEKAKNLGSSIIIVKILAIVSTIFLISPTILLVGNIAFVGLFAPHIVRKVFGIRDYRYVMPLSGLFGAMITSLGLILYRETIYINSSI*MSFIGAPVLAYLG*KH*NRA*

>UUR10_RS00365 Ureaplasma_urealyticum_serovar_10_str_ATCC_33699_NC_011374 ABC transporter ATP-binding protein

MFKIFSKYFCNHQLDRDRIIKESNDYALVVDNLSYKYHKKLPDVLKNLTLDIKKNSFTTILGPNGCGKSTTAKAIVKLLKLKKGNIKVFDKNISELSFKQLAQLISYIPQSIEIPQGTRVIDFITFGRNPYLGISGILGKDDKAVIDLVINEMQLHDLKEKFMQELSGGQRQKVVLALCLVQDTPIILLDEPTTYLDIKNQYELLESLKKLQILRQKTIIAILHDINQAIQYSDEVFVLKDGQIYANGNPNEIITKTLLKDVYNIDAQIDIVDDQKIVHNIKVKDYLSNNK*

>UUR10_RS03115 Ureaplasma_urealyticum_serovar_10_str_ATCC_33699_NC_011374 hypothetical protein

MSYNLQKLMSSCMFELSDDEIKQVENRLNTLFNEIKVFELFDLQQIAPFEVINNSFDNFLRQDEIDSCATNNSEEVLNNCLEVIDHYGVLKNEK*

>UUR10_RS03235 Ureaplasma_urealyticum_serovar_10_str_ATCC_33699_NC_011374 50S ribosomal protein L19

MALFKINKGEIMNFVNSTQLKTDIPSFDSGDTIIVHNRIVEGKKTRIQKFEGVVLRRRGSGSSETVIVRKESNGVGVEQSFNIHSPLVEKIEVIKYGKVRRAYISYMRNRSGKSARIKELNKQ*

>UUR10_RS00510 Ureaplasma_urealyticum_serovar_10_str_ATCC_33699_NC_011374 ABC transporter ATP-binding protein

MNNQFILEVKNLTKIYKKSNQGVFDLSFNVKRGEIHAFIGENGSGKTTTIKSIINAYTNYKGSILIAGFDNKTPKSHEKIGYVPEIALFPSELTTFQYLYSFARLSNLNKKQAIERIDYFLEKFHISDLKNKKPINFSSGQKKKVILIQALLHDPELIILDEPTANLDPSARHEFNTILKELHEQNKTIFICSHILKEMDSYVDSLTLINKGKLVYSGHKYDELEKIYYENVLKNK*

>UUR10_RS00975 Ureaplasma_urealyticum_serovar_10_str_ATCC_33699_NC_011374 hypothetical protein

MYKEILIKNSSANYIVSDPFENMIDAFPIILFENNNYVYYLAGQNAYLPSQMRR*KN*DEILVNKNDLSKEDFLYKDIYINLFQIYITKKTNLTKYTNNQLNFVLTKNDFKKEL*DKLIYIPIKKLINTQKPQILLFEIFKKQDQKYHSTLLFANKNVLKKFYLNSATRVNHEDVINLAFEKLETLQKLSKTTKVKKHLTNLKKY*

>UUR10_RS01860 Ureaplasma_urealyticum_serovar_10_str_ATCC_33699_NC_011374 Holliday junction resolvase RuvX

MRKLALDLGTKSCGFAISDLLGIIASGLDNFIYEENDFIAVLAKIDEIMINYHHEIDTIVLGYPTNVYDGSKNKRTYLIESFYTLLKQHFLNHEKIKIVYEDERFSTKIATQRLKNSCVKAAKIKKVKDKMSAVVILESYLSKNHFN*

>UUR10_RS02490 Ureaplasma_urealyticum_serovar_10_str_ATCC_33699_NC_011374 DUF5385 domain-containing protein

MLDKQEIIESILQADATKGGNSNFLMILIIIIPIILVVVFIMRKKRKQNGENQANVNNKDKSDTNEV*ATIKKYLRSIDDKGKEVIDSYVVKRAEPHNLAQMTKQQKIDYKNEQKAIKALKTTNPEQYKIEMERIKKEKRAKPKELYVVLFTTRNAKTLVVDEPRAIECEVRLVKVNKKENRREIDVVRALDYDEEML*IEPIKAKDDEIYNKRLEADKKKQQKAAERRQKQLEKQKSKTK*

>UUR10_RS02555 Ureaplasma_urealyticum_serovar_10_str_ATCC_33699_NC_011374 nicotinate-nucleotide adenylyltransferase

MKIILFCGAFDMVHNAHIAMAKYAIDLIKADKLIFLPSNFKFFKPINKDDNLEYEKTKLTHGHHRLAMLKIATKNLVNTEVSDYELNQVNKSYTINTIDHFKKLYGAEHEYYFIIGSDNLERFKQ*KD*ERILKEVKIICFKRSGVCLKKTCFQNQCNCENFNFFEHQIILVNDFNYNISSTEIKKQHNLASGIDPAVLDYINEHGLYAL*LLEKHLISYDNFNNLEKKIARINHCRRVAQMCVDLMNVYDKKLIDQAYCAGIYHDILKCLDEQESIAYFNEHKSELNIGDDFIS*RILHSYLGAHLLQTQYGFKNQLILNAIRRHTRPFDFIKDYSELTTLDKILYCADKLEPNRREEIDQINIDYYRKLVFEDLDKAFIEVYKYQQRQRK*

>UUR10_RS03400 Ureaplasma_urealyticum_serovar_10_str_ATCC_33699_NC_011374 hypothetical protein

LKIVLEELKEYLTNKLYFKYKFINIFISILLAT*LISFIVSLILVLNYGYNNKLLNHQKCLTFAILACISFLMLTITIVSFL*IIFHNSTASYLVLKINKYAPKKPIKKLPFLFFKLAYYSFSKKQKSQYSQKQIYEYLTSFNDYV*

>UUR10_RS00135 Ureaplasma_urealyticum_serovar_10_str_ATCC_33699_NC_011374 iron ABC transporter substrate-binding protein

VNKKIKTLIVSSSLSIGLLSLVATNIAACSSSIKSKQEYEIYPTIKGYEQRDFSKILASEFIKIIKTNIQFSSTNKDVKYELYDARLNELDEDVIDLIIRYELKGQTHTYKKVLKGFKNAKQYRLFLENKKYLEDEKNRDALYLTPTVNSSIDISKTTIAEALK*QLSYLSINNGNRDLKYEILKITKSEVANSINVEIRISKGNGENLVSITYSRTLNGFISDEYKKVYLANLEEIKQAKNQIEPQLLDEKTKQKTVKEVIENYKTLFKLPTQTGFIYEIQSVETDVNNKTAVVFNIIVKKGENSQQATLRYTKVITGFVENK*

>UUR10_RS00395 Ureaplasma_urealyticum_serovar_10_str_ATCC_33699_NC_011374 prolipoprotein diacylglyceryl transferase

MQLEIINPESTLINDVVAHRIAFSIGSNFNIY*YGIIFVCGFLLAILTYSLRLKFHYKVPYDPGFYYIFLAIPMTIIGARL*SLAIGDAKDFFDFRNGGLAIQGGVIAGVLSAAIYFPLILRMPKYHVRDLDADGNVIIRQPSM*IYADAIIPTILIGQALGRWGNFINGEIFGAESTVNDLQ*LKKAMPAVFEGMKHYFIEGDKTLFTIYQPLFLYESFFNVIVFVFIYFGLSYIKQLKIGFVSMSYFFFYGVIRFSTESARAPQFSFAGTYVINSLLLIFGVLGALYVQFIAPILRKRFLLDAIIELFYKKKQQAHKFGQLRNPEEFLYYCHK*

>UUR10_RS00840 Ureaplasma_urealyticum_serovar_10_str_ATCC_33699_NC_011374 2,3-bisphosphoglycerate-independent phosphoglycerate mutase

MSLNKKLALIIIDGLGIGKKDDTNAVYLANPKTLNYLIKNYPTLEISAAQQPIGLLENQAGNSEIGHLTIGAGRIILNDNANINSYTKRLDYESLVLNDINNEIVHVVGMYSNGLVHSNYEHIH*IIKELVKNNNQVVLHLISDGRDDYPYGFAQFIEQINALKTQYNVIIKSLSGRYFAMDRDQR*ERTQKAFNTMFIKQDKICEQSLLEVAQSIANHYESDEFVEPIVFNNDEKYNLKPYQKVILTNYRSDRMRQLAHLLKPNRKFNYHNPFLIKDIHLITLVPFPDVDAITLFEKQNLNNTLGDVLNDHHIKQARVAETEKYGHISFFFDGGINKHYASKTQYLIPSQKVATYDLCPQMSASLITKTIIDHYFDHDVFIVNYANPDMVGHSGNMKQTIQAILSVDSEIQKLYDFFKKNNGVLMITGDHGNAETMIDANGQIITSHSINDV*FIITDNNIVFDQTQKFSLANIAPTILEYLNIKKPIEMAASSMIKKIHK*

>UUR10_RS01020 Ureaplasma_urealyticum_serovar_10_str_ATCC_33699_NC_011374 serine/threonine protein kinase

MREQIQTNSILNHKYKVVKHLADGGFSKVYLCCFLSDETKFIVVKVLDISDEKQQMVVYDELRISNLIKNSNSDKRSYIMEYYEYFESGSLETDDKRIYIVFEYIDGLTLREYLDEFKTVTYVKAVEIIRQVALGVSFFHSCNPQIIHRDLKPENCMINKTLSKIKIIDYGAASVFYNREDLTKDQEIKCTIIYASPKLLSLGQKVKEQASKGLNKNALSLINDALGVNYDIHSLGVMLYELITGTNPFSEHTIKDDRDYLEK*TTYDVEPLSSINKTIPKGIDNILIRCFA*KKEDNKLLYKDIYTLIDDLNNVMDPESSLNQDYIKPLNKLRIYRKNVAGLYEIKGKDRK*YLQK*FIILVGCLSIILIILLIIILSFKKSGAI*

>UUR10_RS01855 Ureaplasma_urealyticum_serovar_10_str_ATCC_33699_NC_011374 alanine--tRNA ligase

MLKLSTNEIRKK*IEFFESKDHLFIEPKSLIPKNDPTLL*INSGVSTLKDYFSGKVKPPHKRLVNSQKAIRTNDIFNVGLTSRHHTFFEMLGNFSIGDYFKKEAID*AYEFLIDVLKIDVKKLWVTVFEDDQFTYDE*IKLGIIKEQIIKCNRDRNF*DVGNGPCGPCTEIHYDRGEHFDPNKVGSKLILEDIENDRYVEI*NIVFSQFNNDGHNNYTELLQKNIDTGAGLERIACISQDVPTNFDSDVFMQITKSVEQFSEYKYDMNEYFHPNVAQNKINFAYKVIADHMRATVFAIADGAIPSNKERGYILRRLIRRTMVLVRRLNINNLL*VDAVVNAIASTMGDFYTYLKDEKTLAKIKMILNKEVQLFEKTLQLGLNIFESSIHNQELDKDITFKLVDTYGFPIELIKEICEQRNVKVDLEAFDAMFKHHQLVSKANKANLKVMESQNESLMQLDVDSTFHYEIFK*ENAKIITLFNEDFELVDGLDHEDGYVVFDNTCFYATSGGQQHDTGYIIKNDQQFFVDDVFKAPNRQHVHHVKNASLSMNEYVILQINEQDRKSITANHTAEHLLHYCLKQVLSPDIKQEGAAKYPHKVTFDFTYHAQPTKAQLDKLENVLNEMVQSNFDVQELHMDLDEAKAVGAAAYFEDVYKKLKGKLRVIKMGPSIELCGGTHAHHTSEIERIKIVECASKGAGS*RITMVTGHDNLAKYIHDLYVEYLNEINHLKANLDINDHKLNDLYNAFAN*KNLSIDDYDLLNEKFTELKQALINFKIEFDKQNAKQAIIDIKNTFNAQQTNKRIHVFKNTDNKNIFNALNELINENQNTLFISFNLDENKIQYLLAINEKFATTNQINLNKYIKELNTISNGKGGGKPYFVQGGTSEQEKLDELLTAIDK*VINA*

>UUR10_RS02230 Ureaplasma_urealyticum_serovar_10_str_ATCC_33699_NC_011374 tRNA (adenosine(37)-N6)-threonylcarbamoyltransferase complex transferase subunit TsaD

MEEKYLILSIESSCDETSLALFENNKLIAHKISSSASAQAFHGGVVPELASRYHEHNINRLFVDILNETKIDPLTITHVAYTAMPGLPGCLHVGKVFAKQLASLINAELVPINHLHAHVFSASIDQELVFPFLGLVVSGGESCLYLVSDYDQIKILNQTQDDAIGECYDKVARILG*NYPGGPIIDKNYQEDLATLEFIKSQPAAKNFSFSGLKTAVINYVHNSKQKKLDFDPIVIASSFQKFAINEVIKKVKYYLDLYQLKRLAIGGGVSANSLLRKKIRDLNVISYIPQMIYTGDNAAMIGAYAYALIKNHKKSILIK*

>UUR10_RS03250 Ureaplasma_urealyticum_serovar_10_str_ATCC_33699_NC_011374 Cof-type HAD-IIB family hydrolase

MQYKMLVIDLDGTLLSKTKNISKANLEALKKYISLGGKVVLSTGRSLENTLKIVHLIHYEIKELIEYISCFNGSYIYDVINDQVLFESIINKDVVNEIYDFSLKNNLGF*PYNEKFMQTHFLDVYNINYKLLLQLHHTKRKVCLNPVFNRNDKVYKINLLPSSFTKKLKHSIIDQLIEKFHDQVNISFTSKYIVEVTNKNINKASSLQFIANLYQINLNEIATIGDSPNDIPMFEISGLAAAARTKSKAILEHVDVIIKHKNNSKSVALFINNYLLK*

>UUR10_RS00355 Ureaplasma_urealyticum_serovar_10_str_ATCC_33699_NC_011374 GntR family transcriptional regulator

MDQYKINELIPSEYKLANYFNCGRITIHNAYEVLKVLGIVTTIKGSGYYVYSSIDHFTNKAFANLYEIIDNIKYEQFIIDFEYHGNKFNSITKFSLIKNKKTVAVSYYLTPNIMGLEQFDTNNINISKQLILSGFDDFFNVQTQINYENINLLKSILENENIKFDTPMILNKLTSSSLKVELLVLTHISKEYFSYEETNKLIVN*

>UUR10_RS00580 Ureaplasma_urealyticum_serovar_10_str_ATCC_33699_NC_011374 ABC transporter permease

MNNIN*LS*LRNSYI*IVLAIIYIPLIIIVLLSFTTPSIKGNITSAFD*NDGLNYLTLTTNQFTDALVNTIIVSIIAVPISTIIATMTCFGV*HAKAFYKKLMTASAQTNMMIPDVISGISLALLFAATFIPIGFSFGFSTIILAHISYCTPYAIMIIYPRMLKMKKNLILASYDLGYTKIATFFKIILPYLLPSIISATIIVFAMSFDDFIITKLVGGKVNTIGTEMYSMAKGIKA*AVCFGALMVLLTILIAALISANKIIKLKLINKQTNTRKLKI*NKQV*

>UUR10_RS01110 Ureaplasma_urealyticum_serovar_10_str_ATCC_33699_NC_011374 50S ribosomal protein L23

MELTRVILHPYTTEKTYSIRNKSEHETLTFIVDKNANKYQIREAFIAIFGLKPLKIRTTNRGPAKIRTSTARPGYTKAKKIAYIVMPIGVKVAVSKEEVEAANAK*

>UUR10_RS01605 Ureaplasma_urealyticum_serovar_10_str_ATCC_33699_NC_011374 hypothetical protein

MLKKQNKNKEQY*LEKHLRQKKGLIVS*SIIFSILVLLSISFGLILHFFDSTNLSIQLSFIVNVNKYLVDVTKILVYIGFGLIYLPIVFLLGCWITGINGVHESLYYHVFI*AFYFISVILLIITICLSIATHIYY*

>UUR10_RS02060 Ureaplasma_urealyticum_serovar_10_str_ATCC_33699_NC_011374 hypothetical protein

MKKINKKILFSSLLFGTVAIGTVAVATACSDDKKTKKTINTGTIQPGSSTGTTSKSLTQEKVLRNEIISEILNAKSKKPDRDKM*VN**NSSFEQARKLAMEMDKAVDLTKSSNFKKLIAEGKYKSTFSAQIEDIIAKVKHDIKMYAESPF*KK*RDEKKTVITLVNNNAPREDLAAMNVTSIDLPADFPIIYSKPDYDGIPGLGARFPTPKSKVAPEKLLDDGFSFGDIIVEDGSETQKANLPGQLTESFENTADKVIYLYYDSGLPEAFKNNQRQPEKIKQFED*MKSQNANDFIAKRMLKNPNNKDDLIVMPMSSL*YASYGILGVNYSLHALSEAFGMPKSELDALKAKEEFKVPTQLFTLVNQDTDLKEDKKTIKDECDPFKSHRDPNHKID*KVWATNSQVLDIAITLGLKPDLLVNGELSTSGHEERQLALYLSEYINGPLKDCRTITPSDGIR*ETTSLTKIKDLNVNLILAGIHGEAATKMFGALMNEHKEIANFAITNRRFSDETRKVVAPQDRDQYSQNAALVD*EDYLKTKDLH*

>UUR10_RS02090 Ureaplasma_urealyticum_serovar_10_str_ATCC_33699_NC_011374 ComEC family DNA internalization-related competence protein

VINNTFKTKNYYISFLILATSIACCFYYNNF*ILIVNVCQFY*LYQIKFNYKLIILIIVFSILCFALCLLIHKNFNIHYLITNLEKECD*SLRKQIIVYLSKKYLNKNTLMLIKLMIFNEKVSGNFKQILYDLNIAHLFIVSGLHLNIFLLIINKIFFKK*PKISFVIGICFLIFYGYLLEFSIGFIRVFIMHILSIKFFQKISKMDKLAISGLIIASLSIYNLSMLSFIFAYLALFCIYLLNLFIKDNSILKNIYINLYILSITFIISINLNEKINFLSIIFGYVMNLPILFVYQVLF*FMFIPHFEVVLDGVTNILVKAIYFINEIDANIYIKNYSEIITIFYTSIWLTISL*IYYKKILKKV*

>UUR10_RS02255 Ureaplasma_urealyticum_serovar_10_str_ATCC_33699_NC_011374 DNA polymerase III subunit alpha

MFINLNVHSYYSLLNSALSIDDLIQHALDNNQPYVCLTDLNNMYGCIEFYDKAKAHNLIPIIGLEFEYQNTTLVAYAKNYNGYLKLIK*SS*IMTNTTFIIQEDFDDLIIVCKKGGLVFENPNFYQAQNQNASNAIALQSVFYAQENDKTVFLAMLAIKNDLKLDDFIDCHEFDKNYFLNDHEAQSLFSTIALDNLNKVLNELQVEIHDLPINIPVYDKNNLTVSSEILKQLCISGLKQRLNAHDGQVKKVYAKRLKYELDVISEKQFDDYFLIVYDFINYAKSNGIIVGPGRGSAAGSLVAYCLYITDIDPIKHNLIFERFLNPTRKSMPDIDTDIMDEKRDQVIEYLFEKYGNDHVAYIVTFQRLKAKMALRDVGRILGIDLKVIDKICKNIKTDYDEDIDLAIKKSATLKEMYVLHKELFEISKKLIHAPRQIGTHAAGIILSNSSITNIIPIQLGINDRPLSQYSMEYLERFGLIKMDLLGLKNLTIIDNVLKMIYKTQNKKIDLFNIDYNDKFVFQDLAKAKTNGIFQLESPGMKKVLLKVKPQNIEDISIVSALFRPGPQQNIKTFVERRFKREEFSY*NEQTKKILEPTYGIIIYQEQVIELVKTIANFDIATSDNFRRAISKKDEKILMQLKDDFINGALANNYKQPLVNQIFEYIFSFAHYGFNHSHSLAYSYISY*LAYLKHYYPLEFLSVLLSHTSASKEKLLSYLDETKDFNISIKGPDIQHFSNDFVIDNHKQIIRFGFKTIKGFGDELLKKIKLALENAELSDYISYIDALKKGNISLKNIEILIRIGAFDSFEINRLFLLNNLEEIFEKTGLNGHFFDLNLVGLDYANDMSINERFQEDEIQYLGINLSSLNYTNYTNEIDYSNLKYEIESFNEINTNYEVNIVAQVLNIVQSKTKKGNDIFYLDVLVENKKEKLTIFQNSKHLVDEIDINGIYVFGVKLLNHFNFIVSVKQRV*

>UUR10_RS00415 Ureaplasma_urealyticum_serovar_10_str_ATCC_33699_NC_011374 DNA polymerase III subunit beta

MEVFVSIKKLIEAMKFSTTIANTNNANALLLGVLIEVNENKITFKTTNNQVSGYKEISDGFEYFSSGKILVTAKILLGLISKLKDKSVLLKQVDTNILLIKTENFETQINTMNIESFPSLNFSLEDYVKISLPHQIMQEINAKVLPNVLNSQGIEKIQPISGVLIDTETLDNQLIAIGTDKIKASCLTKPYLGEKFKFIISYSTMKLIMEVLRNVEYSNNQIVDFYVRNKSLVFKVNDAILQTRMIDGVYPNVYSIFNETNEEKNYVFDRRLLIEIIERGMNIVMQEQNPKISIKIENNEAEISLTTFEIGNMKEKMPIINLSNANVEFIVNPSLLAHVLKNFENNDVNFKVKDEILRPIIFIDAKDLGFKQILSRIKN*

>UUR10_RS00760 Ureaplasma_urealyticum_serovar_10_str_ATCC_33699_NC_011374 signal recognition particle-docking protein FtsY

MGFFKKIFNKILGKKDTGQVSEEISQKNEENRILKLEDNSINKFNEGLRKSSSALTNAINELATKYIDINEE*YEHLEEVLIGYDVGYVATNKIIESIRNEMIYQKVNDPELIKSIIIDKIFIYYIQDTEINTEINLKQNQTNVVLVVGVNGVGKTTSIAKITKKFINENKKVLLVAGDTFRAGAVEQLKV*AQRLNVDIELPIKEGQDPASVIYAGVKKGYEQKYDLVICDTSGRLQNKINLMNELKKIHDVIHKFDEHAPHETLLVLDATQGQSGINQAKAFNEVTKISGIILTKMDSTSRGGIVLAIKDAFNIPVKLIGLGEKLDDLSVFDLEMYVDSIVLGMKLDVK*

>UUR10_RS00445 Ureaplasma_urealyticum_serovar_10_str_ATCC_33699_NC_011374 ribonuclease Y

MGYLIAFIILLILFVLLITIVPVVMVVYLKKKQLKLALVPKSQTSFKKAIQKSKDLEEECEDLNNKNNELKKTISDQNLQIDLLKKSNENFLLNATSLTAEQAKKELFNLLKIKFKKELAQDYAKIKHEFNEAQEIYAQNILVETMEQIAEPLIVERSLFNIDIVDENLKGKIIGRDGRNKAVFENEGGVDLIVDRQQPIVGISTPNPIRREIARIVMQKLIDSKNIDINRIELLFKEEREKFEKKAFEIGKNVAEQTLGFFDLPEGIYPYIGRMKFRNSYGQNILSHSLEAAEYAERIAKLINIDPIKAKKAAFFHDIGKTIDFESNLDHVEAGLLIAKKFNLDDYIYNAIESHHNKVIPTTIYGALVKIVDTLSAARPGARVNSYDEYYNRVKELEAICMRFDGVKSAYVIKSGRQLRVIVDSNLVSDEQLDLLSHEIKTAIEESDLLANYKIKIVLIKEKRISIETNIIG*

>UUR10_RS00550 Ureaplasma_urealyticum_serovar_10_str_ATCC_33699_NC_011374 ATP-dependent zinc metalloprotease FtsH

MHFFKKILNLFTSKIESEDNSVKKDDLTQPRKQSPEARKKRNRRIIF*LIVLLIIGTIIGVIIYFSVRKEYDSVIVKSAQTEIVNDKRVLYLNTVRPNSNQITRYTIDEDQLLAARVNILNNTNFQIISNASSLGLSRISFNIVREGVEKFKIGETSIYTPISGATNNQ*N*LTSIIAQNAGIPSSGFNAQVIISPLISIIFFAIFLYIILRVSKAQSDSLLGTNKGNAKLTKSSVRFSDVAGIAEVKEELIEIVDFLKEPKKYVAAGARIPKGVMLYGPPGTGKTLIAKAVAGEANVPFFQTTGSSFEDTFVGVGARRVRELFEKARKSAPAIIFIDEIDSVAKKRGNSLTAVQDQTINQLLSELDGFDTSSGVIVMAATNRLDTLDDAILRPGRFDRQISVNLPDILEREQILRIHSRNKNLSAKVSLEDIARRTAGFSGAQLENVLNEAALLSVRDKATSIHMNHLDEAIDRVIAGPSRPNKVISEREREQVSYHEAGHALIGLYSPGADVVQKITIVARGRAAGYTLQTPERNENILQNKTELISRVRTALGGRAAEELIYGPNEITTGAANDFYKITNIVRAMVASFGMTDVGLTQYIATEGVDNPYRNNYSEQTALAIDIEIEKIIQREYKIVKEMINEYREELELIVQTLLELETILKPQIDYIHQYKQLPPEVIANKNKREASQKQANSSVEEAKVVDDKEKDQKSN*

>UUR10_RS00075 Ureaplasma_urealyticum_serovar_10_str_ATCC_33699_NC_011374 ABC transporter ATP-binding protein

MEIKTNQCPYAIEMHEITKTFLNGTVIANKDVNLFVKKNEIHAIIGENGAGKSTLMSILFGIYKQDSGSIKINGRIVNFNSAKDASKTGIGMVHQHFKLIDTLSVLDNVILGSEGTVQLGMIRRKKIAKELKKIIQEYGLNINLKSKISKITVGQQQKTEILKLLYRDIDILIFDEPTAVLSEDEIQAFLQMLKDFKAAGKTIIVITHKLNEIKEVADSATVIRRGHYIDSFDVKEKTVAEMAELMVGRKLVEIKNVDPITSDEKVFEVKNLDIQSIVKKQKDITKVKSSPTGDDVSLLAANNNNLINFSIRKGEIFAIAGVEGNGQSELAQIISGLLKGNKEAKIILDNQEIEHASIKNRYRLGLSYVPEDRHKHGLVLDDTIAMNTILQQVDDKPYSSLGFFNNSEISKHAINIIKKYDVRGTTRGTSDARGLSGGNQQKLIIGREFERSHKLILLVQPTRGLDLGAIEFIHEQTLEEKRKGNAILLISYELDEILSLADTIAVIHNGYFISVGDRFVMTRQKIGELMAGEKI*

>UUR10_RS01145 Ureaplasma_urealyticum_serovar_10_str_ATCC_33699_NC_011374 30S ribosomal protein S17

MERSRRKVLEGLVVSDKMQKTVVVSVETKSKHPIYRKLVISHKKYHAHNDNDDAKVGDLVEITETRPLSATKN*RVSKILERAR*

>UUR10_RS02895 Ureaplasma_urealyticum_serovar_10_str_ATCC_33699_NC_011374 hypothetical protein

MKK*ISIAFLGFLLSLFIAIGVAFGFKNKKYFKTSLNFKTKLTMINKVDYEKIKKRKQLELKIDNIVYYAKFKFIKQVNDYCVIKIFLNLKTNEKMIGISIYTNTQPLIKSIINNFKL*

>UUR10_RS03440 Ureaplasma_urealyticum_serovar_10_str_ATCC_33699_NC_011374 16S rRNA (adenine(1518)-N(6)/adenine(1519)-N(6))- dimethyltransferase RsmA

LNKTVIKNKLKQESFVPSKKMGQNFLLSNEIKNKIVNVANISKDDLILEIGPG*GAITELLVQKTDTLVAIELDKRLYAHLKTYIKAPNFHIINNDVLCVDLDKLILDYTNTKKNQKIKVVANLPYAISSKIVLKIIQSKLINDAYIMVQKEMAERIGAKVNTRGYNAFTVLVQLFCKTKILFQVNAKEFHPQPKVQSAVIHLENLHNKVDFDIEQVSKFLRICFLNKRKKLKNNLSNIYDIKLVNEMFIDYNLDMNLRAENIEPKMFLELFNYLNKSNNE*

>UUR10_RS01680 Ureaplasma_urealyticum_serovar_10_str_ATCC_33699_NC_011374 hypothetical protein

MNKTNQIVDLLTNKKNDSIQRRDQVVFIKNEQEAKEVLASDISLNDIFNPLLSNDYEQKKIKQLVILNNSIKVNKIEEYTKALENKVDDLYTNVSNQLIEDYQKIQNVNTNEIVTKNEELLNTHLKEVEKICEQIIDE*KIELLGEINFKFEQDLVQIDEMQENKLKAKSLTNEDSFLTRRQVKIKNKIIEEFEKQRTQASQNHLSKRNDILQKERDALLKNNELFELDSNGN*DNFKKIALFISSEIGIINDLEDLLNHQELIRTENYTKRATLNRYRINGHFKDEKNYFKALYDLRVKFMYGLGSEIVILKFLIEHYKTLSELRLKLQELKHPLSSYAVYFDVLNRIEKQTYLLAKVEALKYDNYDFNADVILDGLEENTTNENQVVNKTKVIKTIIEPKYELELNSKDNKFSGVKVFVNDQEITPK*

>UUR10_RS00120 Ureaplasma_urealyticum_serovar_10_str_ATCC_33699_NC_011374 hypothetical protein

MKISFKCKLISVISIIGLSIIFATLTSCANLKQPRLYNSN*LVENAN*KRNNFFRNPYKIHLEFARQLDDVLNFNELKKRLPKPMQNVKSFTE*SQKITQLIKDDVNNVAMLEGIQKMRKRKATVLVLENRDELVNNKENYNKFSILTPPDLPLIYSNPFTEVPGLGLNFPEPINEDVKDVIDRYQSIGHSALRTNPLESVLKQISSFYKTADYVFYMYDSNLFTSNKYKNNYRNRHQLFKKITSHKDFYPHKLLRNRYSKIIFIDRSLF**GSFAMVGQSLIIKELLRIFASKEFSKIK*NYKPFDKSELINLFDHKPSIQERNSIIKSHYINENTTLDYGYKIVGT*ADSIDHLISFGLKPDAIVDYASFNNISLKTQGFSNYLKKFVDYDKLEKTYKTPNDSEKSEFINKDFAIYAGAAYLLNSINKLIVIKNAGS*AIGTTQGINDPKTQFKTRINIAKSNL*

>UUR10_RS01200 Ureaplasma_urealyticum_serovar_10_str_ATCC_33699_NC_011374 nucleoside monophosphate kinase

MKILLIGPPGSGKGSVSELLTKNNALKHVSTGNLFRAILKEDSELARKIKEINVSGGKLVPDEITNQVAKSAIDELIKNQQSFILDGYPRTINQALALEQYCDLDYIFYLDINHQELMKRLTGR*MCPKCAGIYNIHFKKPQVDGVCDNDQATLYQRADDHEDAVSIRLDEYDKLTLPLIKHYKTNPRFIKINANQPIKDVYEDINNYLKQNK*

>UUR10_RS01365 Ureaplasma_urealyticum_serovar_10_str_ATCC_33699_NC_011374 purine-nucleoside phosphorylase

MTAHNQAKLGEIAKVVLMPGDPMRAKWIAETFLENAILVNEVRGMLCYTGTYKGKKISIMGHGMGIPSIGIYSYELYKFYEVQTIIRIGSTGSYKKELNVNDVVLVQKSYSDSTFASLIGAKVCDDKVLLPTKEVNELIEQTAKDLNLKLSLATCHASDVFYNNDFESLDEIIDRTKSDCVDMESFGLFANAQILNKNAATLLTVSDSLITGDALSPTERANTFKKMVTLALESAIKLL*

>UUR10_RS00595 Ureaplasma_urealyticum_serovar_10_str_ATCC_33699_NC_011374 3-5 exonuclease

MRTYDSISEISEDKDLVFVDIEATDNKGDQRIIQFSGYRLNIKKNKIRRFNKKFNPDQEINSRIKTLLNFNKNFNNIEQMPKLDQK*AKEIYNFVKNAIVVTFTDFDIKKMHQLFSHYEFDLEKIIYFDIYKFFEKKLHTNAVPSLFSLGILSGIKIDFFKLHNALYDAFILKEIFLHIRYKTNEELYEMYSYYQFLPKIINSSYFITNEQEKNKGIIKKEVKYVMYIKEFDFTDKFDLNFVVYKKNHHFYSKPIYDSSLELQTISSAIDEHSSPKVQLANTFFEYLSKSAVFSMKKLGIKQSEKFLKFYKTHTNKRKIIKVLSLNLKKEIKPENFVIKAQVICETLSKNEAIHPFVQEYLKAFSDISSQENENNN*

>UUR10_RS02130 Ureaplasma_urealyticum_serovar_10_str_ATCC_33699_NC_011374 ABC transporter permease

MLKNHFHSS*LYTTFLSRLVLFKKSTYFLVGISLFINLIFIIINALNIANDKQNYLILFYVFISINLVLTIIFSTIKAINLFKDLKDDGIEILVFSKSISRKNIIFTKIGFLVLNCVL*SLITYIFNIIFYVVNTKNNNDINLFYLYAFFNYYFCALIFGSIAALIVSR*SNKIAMIVPISCFLPFLVAGGVANLYSTSKINQVAKYMNINYDKYDSNTILDVEKFYLNNKSDEVYLITKNLNNPRFSQRQNEFLKTAFNQAKNASKPFQILS*LSIPYQLNNSFYKNDLDPFNINNHQQNHLNQYFNYHGLESKLYDYELNKNPNLPQFNINENQKQYFVPGALKNVSQFSTLENRNLIYARENVDRFDVNFLEDDNLFSSTNNFIGELK*EVIKNTLESKVFNNFAKKFYDNFDQDIEKPQIITAYNDVINEQSLINFSTIIDESSILFAKKIDNHYVKNLVEKKIYFIVALMYYLYFNKNY*HLLEKLLKNNKVINFYRPSPIRINVNNYAYNIGGIASYELMKKVVNNKTLYRYQLQKSNNYVFQTAQEVYSIKRSKQIVNKNYY*LI*IIFSMILIICLALVYLKRDYK*

>UUR10_RS01360 Ureaplasma_urealyticum_serovar_10_str_ATCC_33699_NC_011374 M42 family metallopeptidase

MSKFSEKEIRQKAIEYMEIYGMSRHEERVATKLKTSLKDVGVSYERDNLGSIIFKKTNSNKGPKILIATHMDEVGFVVQQILDNGQLLLSMVGGV*PNIVIGSVAKVYVDEQRQYTGVFGHTSIHILEPEARSKAVPVKELFVDCGFSSKQQALDLGVEIGTEVYMEGPSLNFHDENYIVGKAVDNRVSVAVLDLLVHSLKDKVIPNQTYFAATVQEEVGLRGAKTVVSKVKPDIGIVIDTTTSHDTYKCPEGDTKLNDGVCIRMKDGGTLVNPALVKYFEALAKKHNIPLYKYVARGGGTDAEELQYGPDGGVLTIGLSIPQRYLHAPIGVATVKDMKAAFDLVREFLEVFDENEFEKVKFK*

>UUR10_RS02165 Ureaplasma_urealyticum_serovar_10_str_ATCC_33699_NC_011374 iron ABC transporter permease

MKTKFNFFKNKQNVIGQKQLLELNKKSFVPYRFKPLLTTMILLITLLIIIILIFSVTLANDLNFSELAKII*QEVVQMIITGAALGVSSYVLQRITRNRFADVSIMGIGTINLILLCALSIPIDFTSANELNILQKKEP*IFMSMSCCLMIVYFIVSRQKENFNYKKLILIGVILTFFLVAIAQSIRG*LNYHANDYVIGHIVGSVQKAPLNTLIIASSFVILGLL*LLFNSYKLNIISTNQQVAKQLGIKINFQIFVALVFVGIMVGASYSVSGDFVYVGLLAGNAAMRKRNNSFSYGILNSGLYGILSTLITY*IGISLIGMDVHHIGAILPLLIGPYFIYKVLRS*

>UUR10_RS01840 Ureaplasma_urealyticum_serovar_10_str_ATCC_33699_NC_011374 hypothetical protein

MSVTNKKYDPQISELLKLRDLANKVDNLKRKLKTIEYNHYCEHENYDSDVNETNNVYEQEVSLNHETQIVKLRVDLNDLTNLSNPKIVEVEKPVYIEKVIEVEKPIEKIVEKIVYVNQENQVNKQESIKEERELKTPIKSGKYVKYRRLKNNGMSDAG*KIFELVKQTQQDKVAAEKRFDEQQEEKKRQREQKRLEKQQKLEAKNEQ*

>UUR10_RS03085 Ureaplasma_urealyticum_serovar_10_str_ATCC_33699_NC_011374 energy-coupling factor transporter ATPase

MSQSDKNTHNYQISSLDKVINDSSVAVEFENVYFAYTEERMILKNVSFTINDNEYVCIIGHNGSGKSTISKVLTGLLKPKSGAIKLFGIEISAANLKYLRNNIGIVFQNPDNQFVGITAEDDIAFGLENRKVPPNKM*DIINDAAIATGIEDLLKKESLELSGGQKQRVAIASVLAINPKVIIFDESTSMLDPKGKNELKDLMVSLRDVAKKTIISITHDMEEVVKADKVIVMSNGEVQYIGTPQEVFANEERLLKMQLDIPFTLRLAKTLKDKGLKIDLTLNNEELIEKICKN*

>UUR10_RS00140 Ureaplasma_urealyticum_serovar_10_str_ATCC_33699_NC_011374 hypothetical protein

MKTKFKKIIILFLGLPVLAIPFIVSACSQLKINQDVILTYRPTVSLAKEFTNDNNTIDDVLKKVKINKDDTNNLLVYDLSKSPKNVQYKIVDIKKDSEQILKVTINAKIQKYKQSINYDFYFQPFLTPKEKEDKTIFQQNLNIIKSA*DYDQKQKTGFFDLKIKTEYQKKSLVEIKTLGEKAFDFGKDLNPQLKVDPKFKDINIKIIDINYFEPLNEKQRELVIEIVITKGENEQQAQLFKKIKVNLN*

>UUR10_RS01090 Ureaplasma_urealyticum_serovar_10_str_ATCC_33699_NC_011374 50S ribosomal protein L20

MRVKGGSVTRQRRKR*LEKAEGS*GTRNTSYRIARQTVIRAAEYAYRDRRNKKRDFRKL*ISRINAAVRELGYTYSQFMNALVKANVVTKDGQGLNRKMLSELAINNPEAFNQLVNKVMK

*

>UUR10_RS01825 Ureaplasma_urealyticum_serovar_10_str_ATCC_33699_NC_011374 aspartate--ammonia ligase

VGQLQKAKINQTQKAIVEIKNRFQKYFAKNLNLSRVTAPLFVEGQSGLNDHLDHKQKAVSFYAKKLNKTLEIVQSLAK*KRLALLDYGFSLYEGLYTDMNAIRADDDIDEIHSIYVDQ*D*EILINKQDCTLDFLKSIVNKIYSTIKTVQLEIDQLYNPKQIILPDSITFIGSQELEDLYPHLTPSQREYEFAKMHQAIFIYQIGYPLKSGYIQSIRSPEYDN*NLNGDLIVYHKLNDQAIELSSMGIRVSKQDFIKQTNFANLKNDQENNFYHQMILNDQLPQTIGGGIGQSRLCMFLLNKKHIGEVQVSV*PNEYKDELLKKGIKLL*

>UUR10_RS02750 Ureaplasma_urealyticum_serovar_10_str_ATCC_33699_NC_011374 30S ribosomal protein S4

MSRYTGSIYKKSRRLGFSLLENNKEFNSGKKRTYGPGQHGNKKVKLSNYGQQLVEKQKLMFLYGLNDRQFRRLYRVALGRPGVLTLNLLQVLESRLDSLVYRAGFAPTRRAARQLVNHSHVLVNGKKVNIPSALVEVGSTIALKEKSLEMPLIKNTLNKPADFIELVDKDKKVAKLSRLPERSELPADVNEAYVVE*YNRLM*

>UUR10_RS00280 Ureaplasma_urealyticum_serovar_10_str_ATCC_33699_NC_011374 ATP F0F1 synthase subunit alpha

MQSTKANPRIKSIFNYIVTVSGIYDYQHHQIFVLKNDPTVKLFVISAKEDVAYLLISNEHANINIGDEIIETNREENVVTDLNYFGKIIDINNNFI*PKPLNNLPSAKIAQTNPAFGLAHNLMTVKTLNEQLNTGIIAIDLLIPIGKGQRELIIGDRQTGKTHIALNTIINQSRAGTKCIYVAIGQKRESLTTVYETLKAHDALKNTIIIDAPLTAYEQYLAPYIGMAHAENLSYQHDVLIVFDDLTKHANIIREMALLTNKPVGKEAMPSDVFFSHSSLLERAGSFKNRKTITALPILQTVDGDITSLISSNIISITDGQIVTSTDLFAAGKVPAINIDLSVSRTGSSVQSRMITKVAGEINKIFRQYKRHLKLAMLDYEFNKETSLLLHKGKLIDKMFLQKGFSLFSYRFIVLSTKLIA*GILKGVKDEQKAFMLLDYAINNYEDAQKAFNTISTTQNYDDKIMKNYFAFILKQYSDYLNLN*EVELEHSFIPLQNEFLMNVAKFLGDK*

>UUR10_RS00545 Ureaplasma_urealyticum_serovar_10_str_ATCC_33699_NC_011374 hypoxanthine phosphoribosyltransferase

MKDIDPRIKEVLITEEQIDQKITEAAN*INKEYEGKEPIMIGILKGCIPFIGKLLPKIKVDMKLDFLAISSFKGGTSAQTEPEIITDLKFEVKDQDLILVEDIVDTGRTIKKVYDLLKIRGARSIKLVTLVDKKDGRLVDLQADFACCDIPLVFIVGFGLDYKEIMRNLPYIGVLKEEVYQEDLNNKNEGDGE*

>UUR10_RS02980 Ureaplasma_urealyticum_serovar_10_str_ATCC_33699_NC_011374 hypothetical protein

LVEHKQKKTLKRCSSHFRKNLTIFRTLIELIKESSPFIAASVFTGLSLILIVVIVAHHKDIGAYAATSVSYLTIFQLSFLQLGTTFGIVFSV*SKRSFKDGKYKFEPTNDTANAASFYSFIFGLVVLLIYFTTSYIYNHFANDHQNTLINQNIGSAYIISGLAVVGLAPLRNYLLMLIRSNKSINILLAIVLDFCT*TTALIAAFLLGSYSSLGYWGYGLGMSIGFIF*TIIIGMTAIEKAQIVLRPLYISKKLFVLSIKLL*IQTILSSLKSVGKMFILLITFVVINERVVGSSLLDFQSSRILMYQSMIFIQMLNLGLSDFLFYLYQKQEVRDRRYHSRQLFA*IFIFGILFALVGSIIFGFSIKQLVELYTREQSPSYIFLEKQVPEHFYQSLRKILINDDELLNIIVNHTNINKDEIINALSSNDKNI*LPMAKKVIDPI*KLGDKFETIYHMKNPFTHRYVDISENNVYKYLIKNNAYILLTFFCTFFSFSSILGRYGGLIARRQRVPYVMIVIQLVMVAFTSGFGLTHQTDPHFIGLMA*SFPLFISSIFILGYSAYKILRGYLNYLKKHRYNDQKTSIMSEKPQEQQIKVRQN*

>UUR10_RS01175 Ureaplasma_urealyticum_serovar_10_str_ATCC_33699_NC_011374 50S ribosomal protein L6

MSRIGNRKLTIPANVNVSVESGKVHIVSQTAKLSVDFPVNLISVDVVDNTIKVSRANDEKQTKMFHGTVNANIANALVGVTTG*KKELEVKGVGFRAKVEGSKLNLGLGFSHPLLIQIPTGLKIETPSATEISISGSDKATVGAFAAVVRAYRKPEPYKGKGVMYKGERIVRKAGKTADKKK*

>UUR10_RS01865 Ureaplasma_urealyticum_serovar_10_str_ATCC_33699_NC_011374 leucine--tRNA ligase

MYNHNKIEKK*QKY*LDNKTFKFVDNPNNPKKFYVLDMFPYPSGKGLHVGHPKGYTATDVISRFKRLNGYDVLHPIG*DAFGLPAEQYALETNNHPHTFTQQNIKIFRKQLQMIGFDFDYDKEVDTTDPQFYQ*TQWIFVQLYKHNLAEIQDIDVN*CENLGTVLSNEEVVLNDKNERVSERGGHPVVRKPMKQ*VLKIVDYADKLLDGLNEVEFSESLKSLQRN*IGKSIGTNVQFKIKDSHLALDVFTTRIDTIYGAQYLVVAPEHPILKSIVSEQQASVVQAYVDQTKKISDLDRIADTNKTGVFSGTYAINPINQEIIPI*VSDYVLMNFATGAVMGVPAHDERDYAFAKKYDLPIKSVIDTKQSLPYTGDGLHINSPMINGLNIEQSQNILNDYLVKNHLAKRVVNYKLRN*IFSRQRY*GEPFPVLFDENNQIKIIEDLPVLLPNLDEFKPSKTGESPLANAQE*LYVEIDGKKYRRETNTMPQ*AGSS*YFLAYILKNEDGSYTPLNSEEAKKRFAK*LPVDVYIGGQEHAVLHLLYARF*HRFLYDIGVVPTKEPFYKVINQGMILGENNEKMSKSKGNVINPDDIIASHGADTLRIYEMFMGPLTASLP*SPDGLDAMRK*LDRVYRLYHNLSELEVVEDVNKLNEEIIITYHTLIKNYTKAINEQAFNIAISEMMVFVNVLYKNKVINYKLLDNFLILLSCFAPHLAEELYSLNHSESVCLQKMPIYDEQKIIAQNVTIPIQINGKLKHTINVLRDTNAEQLINLALACEQVKQAIGDQPIKKQIVVVNKIINFVI*

>UUR10_RS00420 Ureaplasma_urealyticum_serovar_10_str_ATCC_33699_NC_011374 S4 domain-containing protein YaaA

MKKIFISTEYITLNQFLKMAGLINNGGQAKF*LLENEVIVDKKKEDRRGRKLYDQMIVKIGNQLYQIVKTDESR*

>UUR10_RS03210 Ureaplasma_urealyticum_serovar_10_str_ATCC_33699_NC_011374 ABC transporter permease

MNQKNSLESNSSQSNYGELPQQSTTLSQKKRVNYSFSNNFVIFGGGRSLKTAADRDHVVDPLIDFFTFDSKFMTFITKSLKLIAEFFLLA*IVITITFFLINSVPGESALTIGITSPEAKKAILAQYGLDRPLGVRYADYLANIFRGEFGVSTSIRPGVEINSFI*SRYLVSFAVGIFSVLLTLVIGIPLGIFVGRKPGGVLDSVSTVIISIISAVPSLVFGLILLLIGKEVGLPFTFDINNFVTYILPGLALSLGSIIVYVQYIKVEMNRELNSMHAKFAYLKGATVNRFV*LHALKPSLFPIATFFPFVILGSFVGALFIEQIFQIPGSGSLFFEAIISKDYNVILLLVIIYSLITIIGYTIRDALYQIIDPRIRRGGK*

>UUR10_RS03360 Ureaplasma_urealyticum_serovar_10_str_ATCC_33699_NC_011374 transketolase

MNRYVNAMRSLALQAINKANQGHSGMSISAAPIIYTLYKGLMTISKSHPK*FNRDRLVLSAGHGSMALYPVFYFSSLLTLDDIKNFRNDNHLTPGHPEVLSNNYIDASTGPLGQGVANAVGMAITESYLRAEFASLKGVVDHYTYCIVGDGDLQEGISYEAMSIAGKLKLSKLIILHDSNDYQLDSAVSDVNIEDLKMRVESMG*NYLKTDNNPENIFKAIAEAK*KKNVKPTFIEVKTIIGEGTSFENSNEAHAAAISKEELEKFGKRFHTKTNNFEFHQEIFDHFFFNVVARGESAYNQ*QQLVDQYMQTNPEQMQRLLNYINGNYEDLNKMLDENKIVNLSDSTRSYLKQYFAQLKDLKSALVLSADLAKSTFTKIGENAFNDDYKNPYIKFGIREFAMAGAMNGISLHQGAKAIGGTFLAFSDYMKPAIRLTAISNLANLFIFSHDSYAVGGDGPTHQPVDQLPMLRAIPNVEVIRPADHYEVKHALSYSFKQKQKPICLVTSRQAIKQINEQKPQDFTKGAYIINSPFSFSENPDYTIIASGSEVSLANDAAKEIFEKHQLKVKVISAFNLNLFLQQKPEVIKNLVSSKNGLLAIEASSEML**KLSVYTNKFMQIAANQFGRSADGNKLMHEFGFSVENIINQLLNKK*

>UUR10_RS00100 Ureaplasma_urealyticum_serovar_10_str_ATCC_33699_NC_011374 dTMP kinase

MMLTKNSNELKSPKKGLFIVFEGIDGAGKTSILKQLLDVLKEPKLVNKIFLTREPGGKNNTAAELIRDFFLKNLEAFDPLTLAYLYASSRAEHVKKTINPNLEKGHIVISDRFVHSSYIYQGIVQNQSLEVIHHVNQQAIGNLEIDYIFYFDVSVNNALNRMKNRFDNTNAFDSQNKQFYEKLLKQYPSVFNAYNQPKKIIFIDANKSENEVLCEVKERLLEIFKEHKYI*

>UUR10_RS00105 Ureaplasma_urealyticum_serovar_10_str_ATCC_33699_NC_011374 alpha/beta hydrolase

MELIKTNTLNFYFEPAKNELKKGSIVFIHGLDASPHYFFLINQSLLEYDCYFVGLPAHGLTPVNSKKELNIPAFAELFIN*INEIDLKEFYLLGHSLGAGVASLVGFIVPQRVQKLILVCPYHYQYLNPFLNKKLFNA*VLFPNPFLKFKTDVILKKLYIDYRNNYKTLPETR*DSISREYPRVARDISLLCLSLLNIKFNHELKMAQRNLIMPTLVMVSKQDELIDYRLALKVFRNNSQISTYIFNNSGHIPFIEEPKLFTNILLSFLEDRFIEQEESESNDVNEK*

>UUR10_RS01655 Ureaplasma_urealyticum_serovar_10_str_ATCC_33699_NC_011374 hypothetical protein

MSYYYKSKFRITDHALARFRERAASTDVNIKNLSDTFIIPIINERILGIRPLNSLNDNFHIYMDPKNKGYYFLVDKYTNTIISYTKRTNKNDFYHIKKVK*

>UUR10_RS01525 Ureaplasma_urealyticum_serovar_10_str_ATCC_33699_NC_011374 ATP-binding protein

MNNNEIEDELDLENFNYQKALEVPNLKAINLTEDEFNLHF*DIVGVYRSYLNNLKEPNDSGYIYELNRNEYNHLCLVVIKKESKVDKVKKNYILNTIKNMDYDISLTDDSQIFSKKSEILDNDLLVERNKLINFFLEEARKNKKQSANKEDNITTNDQQLKSAFIYGDFGVGKSIITQAYTNTISLKYNLKIAYITLNELFKNVIQFFNYKDISDSVVNELINELSNIDVLVIDDFSSGNLNY*SISTILMPIIENRLKSMKQTIFISNFSIEQLNNSTKNIANIEEQKAKLRLFNRIECLTYGNVFKIKGPSIFKVTNNL*

>UUR10_RS02170 Ureaplasma_urealyticum_serovar_10_str_ATCC_33699_NC_011374 iron ABC transporter permease

MFKNKSLSFKTKLD*KTCLRKVAIALLTILSICVLICYCLFDGRELMDFKTIGEEISEGSSSYTRKFIGTPIAIFLSALALSFSGYSMQVVSRNPLASPTTLGYLPAAILGLAISKLAINHVLYLPFIIGIVFASCLIVINFFLVKGNALEASFKPILVGFAIGGIITGINVLLEDFAKDIHIKITGFVEPPINFQ*QHLYVGGPLIIISGLANLFMAPYYTIISKDYLLAKSLGIKVDLVF*LTAFFAIVSTVSSIILVGVLTLLGMIAPHVARILNPKGNSFQQLLLSFMISLLLLTSSR*LISVYNQFDINFFSAIAALPVFAYIFVSKQYRKNVE*

>UUR10_RS01660 Ureaplasma_urealyticum_serovar_10_str_ATCC_33699_NC_011374 uridine kinase

MNAKSPILVLIAGASGSGKTTFANEIVARIPQNTTSVIICQDSYYISNSQLNKNERRLINYDHPSSFE*DLMREQLSDIKKRKKIKVPIYDYKTEIRLDKTIDISDVDVIVFEGIYAIYDDVINQIADLKVFIETPKDECLIRRILRDVNERNRSFESVITQ*RSTVSPMYDQFVEPSKKNANVSVL*NEHNRVALHLINK*INNIH*

>UUR10_RS00925 Ureaplasma_urealyticum_serovar_10_str_ATCC_33699_NC_011374 phosphate ABC transporter ATP-binding protein

MNDQKDNKEIQTIVPLNQKTNLNELDAKAIYDEFQKLNKYQRAFA*LKLPKQKKAELKQFLNKKKTQVDLLKEDFNDANVFEIRNFNF*YMNRTKHVLHDLNLDIKRNKVTAFIGPSGCGKSTFLRNLNQLNDLIEGTSHEGEIYFLGTNTRSKKISSLELRTRIGMVFQKPTPFEMSIFDNIAYGPRNNGINDRKILEKIVEKSLKSAAL*DEVKDDLDKAGNALSGGQQQRLCIARAIALEPEVLLMDEPTSALDPIATAKIEELILELKKKYSIIIVTHSMAQAQRISDETVFFYQG*IEEAGETKTIFIHPKNKRTKDYISGKIG*

>UUR10_RS00030 Ureaplasma_urealyticum_serovar_10_str_ATCC_33699_NC_011374 RpiB/LacA/LacB family sugar-phosphate isomerase

MIKKIYFGNDHAAYEIKDQIIAHLKQKGYEIIDEGAQVELGSVNYSPYALKVANDVVNDAKNDSLGILLCGTGIGMNMAASKVKGARVALIYNESSAKLAKEHNNANVITIGARENSLEQIIKMIDDFLESKFVGERHQKRLDIITEYEKNQK*

>UUR10_RS00035 Ureaplasma_urealyticum_serovar_10_str_ATCC_33699_NC_011374 hypothetical protein

MSNILSNVSSSSLFSTAPSAAAENTSNLVSQLVPEAYTASGISIAISVFSVIGTIVIALSVLPQTIKTLRERDTASLSLLLFLFNGIATAFLTLYGIGLVTVHPNSLTFLRDIQNGNFIYNREE*VAGYLVCGIFLIMGEALCSVTSFIVLFYKVNNMIKAKNMGMNEEEYYEKQIKPFLKVKGAN*

>UUR10_RS00145 Ureaplasma_urealyticum_serovar_10_str_ATCC_33699_NC_011374 RNA-binding transcriptional accessory protein

MIDLIIKTLVQKLKIEAKYITNVLDLLADNNTIAFIARYRKHLTNNMDEFQIQAIAHEYEYLTKLNKRKEAIIKNLEQKGLLTDDLLESINNCQRLVDLENLYEPYASNKKTKASMAIEKGLEPLALLILKNNPNCNIKEVAKQYLNEQVLSVDEAIAGACDIIAQKVANDATLRQMLYETISKHAKLITKINKIANDPTENFALYYEFSCPIKYLKAYQLMAIDRANELKIITFKLDFKKEFLIDFAINKYTRKNKSDSYDYIKLAVNDGFDRLLIPSVSNAVYKEKLDEAHQQSAQIFSDNLQQLLLQKPLKDHIVLGFDPGYAHGCKLAVVDKNNQLLHTDIIYPHKPQELIEQAKNTLISLINKYQINTIAIGNGTASNESVIFISDLIKEFKLNINYCVISEDGASIYSASLIASEEFPNLSVEKRSAISIARRIIDPLGELIKIDPKSIGVGQYQHDINKNILEQKVDFCIDYCVNQVGVDVNTASIPLLSKVSGLNKRSAKKIFEYVKEHQSIKSREQLKTIPYVTDKVFEQAAGFLRINNSLNFLDKTSIHPESYAIVNQLCEYIKLPINDLINNHQVLNQLNPNDLTNVLKTDVYTIENIINNLKNPLQDVRDDYDIPILRSQMVDINDLKVGMLVQGTIRNQTQFGSFVDIGLKNDALLHISHYSEQDNLHVNKNINAYIDKIDTIAQKISLKLRP*

>UUR10_RS01260 Ureaplasma_urealyticum_serovar_10_str_ATCC_33699_NC_011374 YigZ family protein

MSFKTIKNDQNSLVIIKKSKFYISINKVNAKKEVSMLLKKYQDEYSDATHICYAYIIGPQSNYMKTQDDGEPNGTAGLPILNMIKTKALTNVIIFVIRYFGGIKLGAGGLIRAYTQCAKEIIELCEIINLQKYFYYKVTYKLKDTKFASQILASIDYEKLSENYDSNSVVLEIRSLIKIDIYTSHIQFVFVKNDY*

>UUR10_RS00060 Ureaplasma_urealyticum_serovar_10_str_ATCC_33699_NC_011374 BMP family protein

MKKLNKKKLFLSLGSVFALSSVVAITASCSQKSTLNYSQFY*TSPTSDDDNGFQTKYKSMANDGKRALLMPGFQHPEKLQNALANDKFDANSIALILDAVYNNDNKAEFYKGADRVADVYFKVDEAAFLGGIAAAYMLNSNQSVFGKDNKLT*GGYVALNAKNTTNYLAGFDLGVK*ANEKLKGKSVKQEGTQETKT*IEVEQVYASESSAGGFQPENDNAKKIIRELITKGVDLILPVAITQVGVAVTEAIATTSHNVGVIGVDVEVENDEAINKKTDKFINTNLSGNKNGVVRFSITKRLDVATVKLLENAISGQSLSKGKDEIIIGSEIDPRDKYKLGVNTVGNLSDGVVGISPSAYHYVIDAFNLAQANESDKISTYDQLVNKITNDELFKTLDKKPLVEGYLDVKKETDNDAALLENKTIDPRVNGGTLYKSAKGTYYFYPVAKSTYISNSSSNKFKEL*DGAKTNEEKQSLIGLILSYAGAKVKDGGYSEATYNGLKEFYAKHGIKIPTL*

>UUR10_RS02465 Ureaplasma_urealyticum_serovar_10_str_ATCC_33699_NC_011374 hypothetical protein

MNNKKSKKFYDFLKTIKGFKVITIISYIFVAILLAILI*SAVRLSYYVEDYYARIDDYNELKQNIIKIFKTKDFRLDSPYNQIKMFKYGSIIGVSSFLIIIININIFAY*SYSNVYIPELKDKKLVKNWFRNFQKNNKVQKTDKQEQEDLKKESE*

>UUR10_RS00720 Ureaplasma_urealyticum_serovar_10_str_ATCC_33699_NC_011374 hypothetical protein

MQSNTKLVKYSKIIANRLKAYSNQTRFIEIKTAFELNNEQKQRIKKTIINRFGDERPIKFIVDPSLIGGVSLKINLEIIDSSLKTKLNQIINIKEKEGA*

>UUR10_RS01835 Ureaplasma_urealyticum_serovar_10_str_ATCC_33699_NC_011374 asparagine--tRNA ligase

MQLKIKEIFDQDYTKLEGQKVQIKA*VRSNRDSKKIGFLVLNDGSSLTNLQAVYRVDKISNYEEITAARM*AAVAIEGVIKLTPTAKQPLELEVLNAQILKQSDEDFLLSNNDLSLETLRLNAHLRPRTNLFHAIMKVRATLAFAVHEFMNQNEYS*LAAPLFTGNDAEGAGETFSIQKFDNEEFFGKQTHLSVTGQLQAEAYAQAFGNVYTFGPTFRAEKSHTNRHLAEF*MIEPEMAFVDLKGMQDIVENLIKHVIKAVLEKNQQELEFLAQRNDENLIKKLQKVVDSKFERIEYKDAVKILANAVKSGHQFEDNEIFFGMDLGSEHERYMCETYHQGPVFLQNYPKDIKAFYMKLNDDQQTVASTDLLIPGVGELVGGSQREDNYEKLLKRCQELKMPIESLQ*YLDLRRFGYYMSSGFGIGFERLVMYVTGVNNIKDTIPFPRSHGQIEF*

>UUR10_RS00225 Ureaplasma_urealyticum_serovar_10_str_ATCC_33699_NC_011374 hypothetical protein

VSPISIELIIQISIGLSASLILLFAFLPQTLLTIKTKNTAALTISMFIICFIARLCFSLSAILTIIVYIHNQDYGLSLYALTLPVLICHGINMLLNLIIAFIKINNVYKAKIHKMNESEYIIFAYAQKLKEKVSIKK*

>UUR10_RS00620 Ureaplasma_urealyticum_serovar_10_str_ATCC_33699_NC_011374 dihydrofolate reductase

MLKLI*CQTLNGGISKNNKLP*YVKEELEHFYKTTKNHKIVMGKSTFDSLEQKPLSNRTNIIFSSIMQTPEDQSYFVTNDFQQLLNDAKKEDIFIIGGKELFDIFLNHADALIVSVLNDYYDCNLYMKVDYNNFNLDKKDVYDNFVVNYYSSKKDK*

>UUR10_RS01290 Ureaplasma_urealyticum_serovar_10_str_ATCC_33699_NC_011374 tRNA (cytidine(34)-2-O)-methyltransferase

MLHIVLFEPEIILNTGNIARMCVGFNANLHLIRPYGFILDKARFDKDFVRASANHLDELKLFEYDDFYEFIAKNNIIENQIYFFTRYGTKAPCDYKYANLNKEDVYLVFGKESTGIDHDILRKYPQN*IRIPTSINLRSLNIANSVAMGIYEVLRQNDFSDLLKYEPHKKFE*

>UUR10_RS01520 Ureaplasma_urealyticum_serovar_10_str_ATCC_33699_NC_011374 deoxyribonuclease IV

MDKYNLIIGSHVSLKANDFFYGSVKEALSYGANTLMVYTGAPQNTRRQPIELFKINEAHNLLKEHNIDLNNLIVHAPYIINPCSSKKYVRELAKEFLIQEIERTQFMGITKIVLHPGSCLDQDEDIALKQVYTMLNEIFATINTNVVVCLETMSGKGSEIGINLKQLKTIIDNVDSKKNIGVCLDTCHMSDSGIALDHDSFNQYLKEFDAQIGIDYIKVLHINDSKNPRGANKDRHENLGYGTIGFDNLINIIYHPLLNNIPKILETP*FDFHDQSISLYEYEIKMIRDRKWFDIKYKLLVGNK*

>UUR10_RS03390 Ureaplasma_urealyticum_serovar_10_str_ATCC_33699_NC_011374 DNA-processing protein DprA

LNYLTHYFSLKYKGD*LKIYLALINHEEQNEHSLLENEEYERLKAIDLTDEKYPNKYKKINQPPFILYYSKTIE*LDKKNI*LIDYENAISNYDILTLIKAGYGFVIYYDNAILDVLINRLITLKARICIISNEGIYSSKNIKYLNYCNVSVISEIPKDYNGSQTKQDLKRLACASSDHILCVNELYQIDDYLLNQYKDCQLDLVLLDKKLTKPNTIYPKYINRIYQFIKNTKLIN*

>UUR10_RS00045 Ureaplasma_urealyticum_serovar_10_str_ATCC_33699_NC_011374 magnesium transporter

VKNKQNTSNLNNNTNINQNFLSQNILKAYEHKDIAGLKKLLKSKRIDKIVESLEAIDDTQIILFVLVATKDGICGEIFKYLNTELKSKIIDDASLQQLRIILFELYNDDVTILKNDFPMHTKKILLSLDSQQRAAIKQLSEFDEDEAGSIVNSDFFTINQSISVKEALIEIKRMYNDFEQSNIIYVVDDYNRLKGYVTIHSLLFADSFDLKISSVVKEDVFYVRSDEDIDAVLDIFRKYQIEQLAVVDKNDQLIGYISDNDILPVINTETTTDIYKMYGISELDFPYIKSSVFVLFKSRLL*LAVLMISATCTGFLIDKFQNVGQLVTAGLSTLVIVPIIPAMTGTSGNAGSQAAASVIRALSIGEITTKEYNKVLAKEFLVGVLIGLVLAVINFARLIIYFAIIKPDLQQYNVLYNNITNNPHSQMIVGAIVSAGSSVALFFAIVISKLLGGILPLLATKLKIDPTIMSTPILSTLLDMVTTIILFGFGILFLLIIVDKVKVEELNNMRESIHAVKNINSLLPKQNFLLNNSNYLNHLA*

>UUR10_RS00710 Ureaplasma_urealyticum_serovar_10_str_ATCC_33699_NC_011374 hypothetical protein

MNKSFEEFSKLPKVKQIFTPWFIVALVVFIVGLVIAFTMVGLLNEGFENARALVNDQGQIVGKVADVNGNDHSLMS*LGYDQHANYQELYKIINNRLIDGQTMESLMNNTDKKVDPKLYEALHYHAHSKLINHNGT*GFTTKVLSD*ENS*FYKYNQLFSQLADSKDSILAAQGAKSLKKLSTIIQYQNPNYIVYN*VFIVFMMPQMITFVIIVVKLATVLSPKKSAEEKQAYKLAKLEAKKQKKAKNLNNSLQQTNLNLEAN*

>UUR10_RS01850 Ureaplasma_urealyticum_serovar_10_str_ATCC_33699_NC_011374 thymidine phosphorylase

MLNVVEIISKKKNNIELSEQEIKFVYDGFVNKTIPDYQMAAFLMAVNFCGYSENEQYYATKAMVESGKVLDLRVENKIVVDKHSSGGVGDKVSIILTPLLSALGLYVGKMSGRGLGHTGGTVDKLESLNLNLDFDLKTYMDQLKDNGLLLTGQSDDMVLADKYIYALRDVTATSDVFDLMVGSIMAKKLALITDYIFLDVKVGEGAFCKNVEQAESLATKMLKLSKKFNRNTIIHLTNMDKPLGKAIGNAIEIKESMDYLLGKPVPQDLYDLINQFALDILIDTKFAKNKEDAQAKIDNVIKNGLAYQKFVN*VK*YKGDYISLENNTYFNPKYKLEILAKQDGYLDFKSTKELGMIAVDLKAGRKVKTDQLDFQAGIYLNKKNNDFVKTNEVIATLYANQPISDEVVNKYHNNVVYLKTPKQIAPSIIKVMR*

>UUR10_RS02215 Ureaplasma_urealyticum_serovar_10_str_ATCC_33699_NC_011374 IS3 family transposase

MSKIIDNIDTKALRIDHYEE*EQNHIIKVINAYRHHESKKEKFKMIHELIKNHQMHLTKLIPLFDVSISGYYK*LEDLKIDQVCPIKKRNMDLIEKICKSHKHYVGCRKIQKILETQYNVKLNYKTINLYMNKMDLCKPCHHDYDAHRHEHCENEE*

>UUR10_RS00050 Ureaplasma_urealyticum_serovar_10_str_ATCC_33699_NC_011374 50S ribosomal protein L10

MANVRPSVVFKQQEVDHMADILKNSKSFIVFEYHGLTAANILALRNVLHSSNSKLFVLKNNITARAFEKAGVTGFEDRLTGPNAIAVAMDDEIAAIKAVNDVAKEFDFVKIKGAYLENKFADTHKIDQLAAIPGREGLYSMLLSCFTAPLRNVLYGLKAVAEQKGE*

>UUR10_RS00700 Ureaplasma_urealyticum_serovar_10_str_ATCC_33699_NC_011374 F0F1 ATP synthase subunit beta

MTEVKKGKINQILGPVVDVRFPSE*LPEINTALELNNHGSKLVLEVSQLVGDNIARCIAMDTTDGLVRGQEVINTEKPIMMPVGKQVLGRMFNVTGDPIDEQPAPTGKRMPIHRPAPSFAEQAEAIEILETGIKVVDLLVPFAKGGKIGLFGGAGVGKTVLMQELIHNIAKNHGGLSVFAGVGERTREGNDLYYEMAESDVLDKTALVFGQMNEPPGARMRVALSGLTMAEEFRDAFGQDVLLFIDNIFRFTQAGSEVSALLGRMPSAVGYQPTLAFEMGQLQERITSTKKGSITSVQAVYVPADDLTDPAPATTFSHLDAKVVLDRAIASLGLYPAISPLQSTSRLLDPLVVGVKHYSVARRVIEILQRFMELQDIIAILGMDELSEEDRQLVMRARKVRNYLSQPSHVAEKFSGQPGLSVKLEDTIEGFRKILDGECDDIHEQHFLYVGKIDDVFEKVAKSK*

>UUR10_RS00870 Ureaplasma_urealyticum_serovar_10_str_ATCC_33699_NC_011374 DNA-directed RNA polymerase subunit beta

MSQKGIKSLTISIASPEQILS*SKGEITKPETINYKSLKPEPNGLFDESIFGPSKDYECYCGKYRKVKHKGKVCERCHVEITESIVRRERMGHIELAAPVAHI*FTKELPSPSKISLLLDITYKEVDQVVYFVNYIVLDEGNNEYDGKSIFNKKEVLDLTSPKNSIRSRNKLRRTLRNIQERIEEELNHEREALIQDFDYRLAVTYDQMLKDSNIPFSVKDVMAFIEKHTGVRFGIGAEAIHELLEKLNLEEEHEKIKQAIQNSPNAYDQKTKRLLRRLECVR*IKDSGSKPE*MVMTRIPVTPSETRPIISLDGGRFTTSDTNNFYRKIIIRNERLKQMQATDAPEILLDNEKRLLQEAVDSLFDNNSRKKPVVGKDKRPLKSLSNHLKGKQGLFRQNLLGKRVDYSGRSVIVVGPELKMYEVGIPALMILKLFRPYIISELIRKRDEFGNEIQPICANIKLAEQKILAQDDEI*PVVEKVIKQRPVILNRAPTLHRLGIQAFEPKMVDGKAIRLHPLVTTAFNADFDGDQMAVHIPLSKEAVAEARSILLAS*HILGPKDGKPIITPTQDMILGIYYLTKEKFPQPIEEMILKDPVQARIEFINHFHIFATQDEAIRAYKLKTIRINDVIGITTKAFDNKSFSKEGILVTTVGKIIFNQAFPTNFPYINDVKNLYGDNQFEIIGMHESILDYLKAYNLKEPLTKKTLSTVIDYLYKVSEIEVVPQTMDKIKALGFKYSMISATSISAFDIPSYDQKYEYFKETDELVAKLREFYLDGKLTDDERYTKVVQA*SQTKDKVTHDIEKLINSDEYKDNPIVIMAKSGARGNTSNFTQLAGMRGLMSKSYNYDQKNNSGVIKDTIEIPIKHSFIEGLSVSEYFNSSFGARKGMTDTAMKTAKSGYMTRKLVDSTQAVVIKGNDCGTKEGIIVREIRNTKDNTSIESLKDRIVGRFSINPIYDTKNKLIIEGDKLITNEIANMIQNSGIREVEVRSPLHCSSLYGVCQKCFGLDLSTNKLIETGTAIGVIAAQSIGEPGTQLTMRTFHTGGVAGDTNITQGFERIKQLFDCIQPQENEKAIISQVKGTVDRIEKDSNTNGYNVVIKYNKDNFVSYPTRPNAVLRIKTGDNVVAGQKITEGSIDVNDLLKYAGIENVRHYIIKEVQKVYRMQGIEISDKYIEVIISQLTNKVTITNPGDSGLFVGETISINEFTEVAQSMLVNKKKPPSAINQVFGLDHAPSKSGSFLSAASFQDTKKILTDAAARSQKDMLIGLKENVILGNLIPAGTGLKDVEEVIAYGEEMYKKQY*

>UUR10_RS02455 Ureaplasma_urealyticum_serovar_10_str_ATCC_33699_NC_011374 phenylalanine--tRNA ligase subunit beta

MILSLNLLHKISPKLKKISLNELCTALMDLGCEVETINTIKPSTNLVFAKVLEKTKHPNANHLNLVKVKANQEVYEIVCGADNFNVNN*VVLAKINAELANGLKITPRELRGYVSNGMLCAYSEINPEVAHFLGQTDLDGILVLHDSYDHYKTPNQIFNLDDVILDLSIPSNRNDLNGYF*MAKELCAYFDLEYVIDATINHRSHKEIVNVRILSDDVNSYGMIEVKNIQNYTLK*NTKSVLVNNQIKIVNNFADNMNFLTLLTANPLHAFDAHKISGQIIVKNAEEDSILLGLDQKEYAIKKGDLIIVDDQKILALAGIIGSNDSKIDNNTTTAYIECANFNPLLIANTARRLKINTTAAMRFSKPLTNYVTKATLKKLLAHFKLDAKLICYFKHLVHNVIKNKIDQVSDFVGTKINLDTAHTFLKRLGYKINKTNLITPSHRYDVLNEFDVYEDIMKKFSIQQIKPQPINFDILSFKNNIAYDFEKKVSDFLVDQGLFECKTYNLKSQTQAYEIDFFNFQQAYEINNPISNIRSHLKLNNLNSLLEVLEYNQNQKNELENIFEISKINPINSNQQTILSIVLCKPLINAKLNDSIVVNNFVTTKALLHVLLTKLNISYVYDTNHIVNELYENNQLALVNENKQVFGFIGQLKNQIKKTYGLNNDIFVINLNLTSYLNQEQAITKVIKPSVYHDIVRDVSVKLASNVDLNDVMDNIEKIKNIRKVEISDLYVKDDEIIYTFKYYINDYSSNLSSEQIAVIEQEVNNYLKQF*

>UUR10_RS00900 Ureaplasma_urealyticum_serovar_10_str_ATCC_33699_NC_011374 16S rRNA (cytidine(1402)-2-O)-methyltransferase

MKLCDKEYILSIIATPIGNLEEASERVIKSLNEAYVILCEDTRMTSKLLHLLNIYDYKKLVSFHNFNEVEKLNEAINYIKKYPTALVSDAGYPTISDPGYKLINECHRQNIGVQVINGPSSLMHALVASGMCSQDFMFLGFLGKTQKQRVEKLKQYKNLQTTFVIYEAVHRLQTTLMDVYET*GDVLVFIGRELTKKNESHYYGYLSQLPPITEKGEFVIVIDNKTNFDVIKNNDINECLNEIRTLIKQNFKLKDACKEVSKTSGFDSKELYKLMVNQK*

>UUR10_RS03470 Ureaplasma_urealyticum_serovar_10_str_ATCC_33699_NC_011374 YneF family protein

MSFSNFLFKVSEVFSSIIHEATDIVTQADLDNANAHAHSLAVGLGIGIVLFLIAGLVIGYFVSMKIMKRQLKKNPPISKDTIRMIYQQVGRKPSESQINEIYNRAVKQK*

>UUR10_RS01625 Ureaplasma_urealyticum_serovar_10_str_ATCC_33699_NC_011374 LemA family protein

MDL*KEQDPSGISPNVSNERKIATASSGEKALYIFIVILSIITIIG*IFLLI**FKTKNHLIQTKNSVNEASSSIQVAQTKRFDLLNKMIEQTKSYYKFEQKVLDEITKNRSVQMSSDINKNEEVLSKLQNLVNVQFERYPDLKSSNILMELMSTSSYLENEIASSRRAYNSRATD*NIMIFQFMTVIVAAKLKLDTFPIYAASKQERADVSMKSLSDF*

>UUR10_RS02070 Ureaplasma_urealyticum_serovar_10_str_ATCC_33699_NC_011374 hypothetical protein

MKKTLVLYARVCPTKSFHTQQIFEAFQKQTNFVCVNLDANQPFDVLKEQEFILGFDRIILLFTLN*YNIP*SLSRYFIEV*RTFPFSLENKEVYKIITTGANQDFYEKKDPNINNISVEEYLNNVNGMLKKLRASIQKSFYYYGAINQDQARLNQFVNELIKYFKNQN*

**Supplementary Sequence file 2 Essencial core protein sequences of *U. urealyticum***

>UUR10_RS03090 Ureaplasma_urealyticum_serovar_10_str_ATCC_33699_NC_011374 50S ribosomal protein L1

MAKISKKLSAAYEGIDKQKAYPLFDAIKLAQEKSITKFDGSINIAVKLNLDTTKVEQQLRGSISLPNGNGKNVRVLVLSEDITKEQAAAVGADYFGGADYIQNIEKMLNQIDVIITNQKMMPLLAKLGKVLGPRGLMPNPKIGTVTNDVLKAVEEFKKGRIEYRTDTYGNIHMSIGRVSFETAKIEENANALLSLIRSKKPATVKGQYIQNIAISPTMGPGIKVIINNN*

>UUR10_RS00315 Ureaplasma_urealyticum_serovar_10_str_ATCC_33699_NC_011374 SsrA-binding protein SmpB

MIVSNKHARRNYELLEFFECGIVLKGTEVKSISRANCSINEAYVQIVKNEALILNMHVASFFEGNNFNQDPYRNRKLLLHKKEIIKLQHLVQTQRMTIVPTKIY*KNNKLKVEIALAKGKQLHDKREDLKKRDLARESRLF*

>UUR10_RS00735 Ureaplasma_urealyticum_serovar_10_str_ATCC_33699_NC_011374 ATP synthase subunit C

MSSFIDITNVISSHVEANLPAVSAENVQSLANGAGIAYLGKYIGTGITMLAAGAVGLMQGFSTANAVQAVARNPEAQPKILSTMIVGLALAEAVAIYALIVSILIIFVA*

>UUR10_RS01165 Ureaplasma_urealyticum_serovar_10_str_ATCC_33699_NC_011374 type Z 30S ribosomal protein S14

MAKKSLIAKQKKHQKFAVREYTRCVRCGRPHAVNRKFGVCRLCFRDLAYAGAIPGIKKAS**

>UUR10_RS01225 Ureaplasma_urealyticum_serovar_10_str_ATCC_33699_NC_011374 30S ribosomal protein S11

MAKKKKLSFTNGIAYIHATKNNTIITLADEQGSVLS*ASSGSIGYKGTKKKTPYSAGIAAEAAAKAVIDMGLKSVEVHVNGTGASRDTAIRSLQAAGLEVTKIKDVTPIPHNGCRPPKKPR*

>UUR10_RS01385 Ureaplasma_urealyticum_serovar_10_str_ATCC_33699_NC_011374 histidine--tRNA ligase

MSNYTKPRGTVDLYNEAMNEFKSLENFLLTTTKKYGFQQIKTPIFEFAELFMKSAGESSDLVSKEMYLFKDKSDR*LALRPEGTAGVIRAVVENKLLLNNPLPLKLMYFEPCFRYERPQAGRQRQFHQFGVEVLGTKNIYYDFELIALANNILKKLAISDYVLEINYISTAHNRSL*VKSLQEYFNLYRDELTPLSQERITTNPLRILDDKLESQKLVVQQAPKITNFLSNEEKEEFALIKKMLDEHDIKYRVNEGLVRGLDYYSGLVFEFISTSPRLLGQSTIIGGGRYGQLIKQTGGPDYEGIGFGIGIERLLIALLDSNKQILNNFEDKYLIAYFDKELENEAIKLTQSLRINNQLNVDIILDTIKADKIFRLAQRLNAKKLIILAKKE*LNKQVILKDLLSFEQKTLNLDEIKKIKE*

>UUR10_RS01565 Ureaplasma_urealyticum_serovar_10_str_ATCC_33699_NC_011374 inorganic diphosphatase

MKLNVTIEIPKNSNIKYEYDRATKEITVDRILYGSMVYPHNYGFLKEALDYDGDELDVLVFADQAFQPGIKVPARILGAMKMIDGGETDTKLLAVIDVDPRYKHINTFKDIPLH*LAEVQDFFENYKNLQNKKVEILGFEDEV*AQKEYEECVALMQEHGHLKKDEFVSKMMKQRPEKYSQ*

>UUR10_RS01080 Ureaplasma_urealyticum_serovar_10_str_ATCC_33699_NC_011374 translation initiation factor IF-3

MNTPNNQRHMSSNNDARKNQPLINDQIRFRTMVVIDDHGNNLGEMNRIDALNLATSKNLDLVVIAKKGNIPVTKILDYGKYKYEQKRRQKESRKNQTIIKVKEIKIKPMIGEHDLKVRAENAKR*LEDKDNVKFVIEARGRMCTKDEFILQAYEKFIDLIKDYGTVVQANKKVSNYRYETIIEPIKK*

>UUR10_RS02990 Ureaplasma_urealyticum_serovar_10_str_ATCC_33699_NC_011374 elongation factor Tu

MAKAKFERTKPHVNIGTIGHVDHGKTTLTAAISTVLAKKGQAIAQSYADVDKTPEERERGITINASHVEYETKTRHYAHVDCPGHADYVKNMITGAAQMDGAILVIAASDGVMAQTKEHILLARQVGVPKIVVFLNKCDFMTDPDMQDLVEMEVRELLTKYGFDGDNTPVIRGSGLKALEGDPV*EAKIDELMDAVDS*IPLPERSTDKPFLLAIEDVFTISGRGTVVTGRVERGTLKVNDEVEIVGLKDTQKTVVTGIEMFRKSLDQAEAGDNAGILLRGIKKEDVERGQVLVKPGSIKPHRTFTAKVYILKKEEGGRHTPIVSGYRPQFYFRTTDVTGAISLPAGVDLVMPGDDVEMTVELIAPVAIEDGSKFSIREGGKTVGHGSVIKTSN*

>UUR10_RS00910 Ureaplasma_urealyticum_serovar_10_str_ATCC_33699_NC_011374 methionine--tRNA ligase

MLKQKKFFISTPIYYSSGNPHIGHAYTTIIADVLARYKRLFGYDVFFLTGMDEHGQKIQQKAFEENISPKALVDRNSIIFLNL*KRLHISFSKFIRTTQMDHEESVQKVFSYLYKQGKIYLGQWTGYYCVSCEENYNPAEIIKSQDNIMLCRMGHKLETKSEESYFYKMSDQAPFLKTYYQNHPNFIIPNERANEMVNNFLNNLEDLSISRTTFD*GIPIAENPKHVIYV*LDALMNYLTATGYLSNNEELFQKY*CDNETEIVHLLSKEIARFHCIY*PIFLNDLQIRFPSTILSHG*IITKEGKMSKSLGNVIDPNVLIDTYGVDALRYYLMADLSLFRDAIFSEDNLIETYNTQLANSYGNMISRTLGMLKKYRNNIVPKYVGCVLKNDEKLENLINKNIELVQENINKYSIDKALNCIQEILVEANKYVEDNKP*ELAKNQQKQELDSLLVHLVKVIQVTTTLLSPILIEGSKKAVEQLNFDESFLTLASLASYDIFNYHKVNDSKPIFARIIVEKQ*

>UUR10_RS01340 Ureaplasma_urealyticum_serovar_10_str_ATCC_33699_NC_011374 nicotinate phosphoribosyltransferase

MGPVIPNTRLIDFKFDRDLLNKAYTSHYFIKTCKIIELHAPSHSVIMQFTHFSKTPIMVCGTSEVLALLEFCLSRKELKQLKIYYVPDGHVIKPKEALFAIEGPYEIFG*LENIIDSILARRSSVATNCYNVLNVINDEQKVIYMSDRSDDYSLQPYDGYAAAVGGMQYFVTQKQVEFLKDINYECKVMGSMPHALIQQNNGRVDLACEMFAQTFPNDPLIAVIDYNNNVLNDLEQLRYMFDRLYAVRIDTAKDLIDNSLLSTFDNVRNHDLHGCNPYLIDLVREYLDNNGGEHIKIIASSAIDLNSIKNFNKHNSAIDFYGIGTYLTHLSIHITADLVCLDNVYGAKVGRKIAKNFAEMTLY*

>UUR10_RS01390 Ureaplasma_urealyticum_serovar_10_str_ATCC_33699_NC_011374 aspartate--tRNA ligase

MRVYCGRIGKEHLEKNVILNG*VKKVRKMGNLVFVDLKDRFGIVQIFATKQDEVFDELTQLSREDVINVEGLVLLRKSPNHELKTGEFEVHAQKLLIYSKAKTPPLIIEDETDANEEIRFKYRYLDLRRDVNLRTFELRSKVYQTFRNYLHSEEFIETETPILAKPTPEGARDFYVPTRTKKFYALPQSPQTFKQLLMVAGFQKYFQIAKCFRDEDLRSDRQPEFTQVDIELSFADELEIQTLIENLFKHVFKQTINVDLTTPFVRMSYEQAINDYGSDKPDLRFDLKLKTLDTYFKTSKSQIFQKALSNNQSIRAILVPNVNLNKKQIQSLEKFAKDKGAKGLA*ISIENEKVIDGSLSSIKEDHVIYQTIFKDYHLSTGTILLIADEFDIASQSLGLVRVNLASMLNLKKPNDFKFV*IID*PLYEYDDENQRFVAAHHPFTMPTLETLDTFDIDQKNAKGRSYDIVLNGYEVGGGSVRIINQQIQRRMFKSINMSDEEANLKFGFLLNAFEYGVPPHCGIALGLDRLIMILINSEYIRDVVAFPKNNNGVDMMLDAPASMNDEDLKELGLIIKND*

>UUR10_RS00010 Ureaplasma_urealyticum_serovar_10_str_ATCC_33699_NC_011374 50S ribosomal protein L31

MKDIHPVSKPCVYNCVTCKKEFVINSAAKNTEVAIEVCSNCHTFFIGKQNATTTLRGRAEKLNNRFEAGLNNINKKPEKKKVQGKSEPRKSLNEL*

>UUR10_RS00950 Ureaplasma_urealyticum_serovar_10_str_ATCC_33699_NC_011374 30S ribosomal protein S15

MAVSKQQKHDLTVKFGGSASNTGKTEVQVAILSAEIDSLTTHMIENKKDKASKRGLYKKVAQRKKLLSYLQRVDIERYRALIKELNLRG*

>UUR10_RS01095 Ureaplasma_urealyticum_serovar_10_str_ATCC_33699_NC_011374 30S ribosomal protein S10

MNQELRIRLESYDHRLLDDTVKTIVDISNSTGSKLRGPIPLPTKKEIFTILRSPHVNKSSREQFERRTHKRLIILENPQPKTMEALKRLSVPFGVEVTFKI*

>UUR10_RS03300 Ureaplasma_urealyticum_serovar_10_str_ATCC_33699_NC_011374 30S ribosomal protein S9

MQKSNIVEYKGLGRRKSSIARVKLVPGSGKVFINDRQPENYFPNKLVIQDMMQPLVLTKTAETYDVYVKVIGGGFNGQAGAIRLGITRALIQTREDLKTDLRKAGLVTRDSRVKERKKFGLYGARRAPQFTKR*

>UUR10_RS01740 Ureaplasma_urealyticum_serovar_10_str_ATCC_33699_NC_011374 endopeptidase La

MKKPILISRAIVVLPYETTTIEVGRPKSIQAIDLAKQSSSKEIIVISQKNIDTDEVVNFDELYKVGTLVKIKSIVDNFDDGYSIEVEGIKAVYINSDSEVIDAIEYEYEDVITNPILSTKDEVAINEINSEIFNTINKRTKHKDITFENMHALISLEKEKFAYLAAATYINDYDGEIKEKTIKDRINILLQPNLLLVHETILHFLFDQLVDKRVIEEEVEKMIADKINNNLQKQQREFFLREKLKVVKEQLGELSSREEDADKIRAKIEQLELPPNVRERALAELNRFESAMSSNESSVIKSYLD*LLDLP*TQQGVDNTDLMSVRTHLDDNHYGIEKVKERILEYLALRMRNPNLKGPIICLVGPPGVGKTSLVTSIAQALNKKFVKVSLGGVRDESEIRGHRKTYVGAMPGRIIKGMKKAGVVNPLFLLDEIDKMTSDQRGDPAAAMLEVLDPEQNKNFSDNYIEEEYDLSKVMFMATANYYQQIPYALIDRLEVIELSSYTAIEKREIAKSHLLKRIFTDAKLNENELIFNDDALDFIINHYTKEAGVRELDRQLGHIVRKYIVETYKNKNNKSKPSVEVDEAVIIKYLGKIKFDFNKKEETTIPGIVNGMAYTAAGGDLLPIEVNHSTNGKGGNITITGNLEKTMNESVSVALGFVKANAEKYGIDTKKVSFKEIDIHVHVPSGGIPKDGPSAGIAITTAIISSLSQRPVRTTLSMTGEIMLRGNVGIIGGVKEKVISAYRAGVREIILPIDDERYLEDVPKYILDDIKIHLVKHYDEVYNIVFGTK*

>UUR10_RS00500 Ureaplasma_urealyticum_serovar_10_str_ATCC_33699_NC_011374 signal recognition particle protein

MFKAMIGNIVSKQMSKKLKNATIAEEDIKELLSEIRITLLDADVNLLVVKKFIKNIKEKTIGLYVEQNQKPADVVLKVIKDELVEILGKENKPVNTAKSQLKIMMVGLQGSGKTTTAGKLANYFRNKYNKKPLLVAADIYRPAAIDQLRTLAKQVRVDF*EEGTQRPDLTVKNALHKADENENNLVIVDTAGRLQTNEELMQELVNVKKTLNPDEVFLVVDAMAGQDIINVATEFNN*LKLTGIIVTKLDSDARAGAVLSLTSLLNVPIKFTGTGEKIGSIDSFYPERMADRILGLGDIMTLAEKAADVIDEKQVRGSMQRMMAGKMDLEDLMRQMSQISKLGSFSGIAKMIPGLNSISENQIDDAENKMKI*TILLSSMTLKERRDPRVFKKEPSRRMRVLKGSGRSPDELNKLLKQ*EVSRDKMAELGKMLQKGKNPFSKSGGIFG*

>UUR10_RS01150 Ureaplasma_urealyticum_serovar_10_str_ATCC_33699_NC_011374 50S ribosomal protein L14

MIQHMTRLKVADNTGAKEVGVIKVLGGSKKRYASVGDIVVVSVKKATPAGLIAKGQMAKAVIVRTKKSIRRESGLLIRFDENACVLIKEDKTPRGSRIFGPVAREIRDRGYTKIASLAPEVL*

>UUR10_RS01105 Ureaplasma_urealyticum_serovar_10_str_ATCC_33699_NC_011374 50S ribosomal protein L4

MAKIKLLSIDGNFAKELEVTSDLFVEVPHKQAMFDSVLAENAAERQGTHSTLTKGEVRGGGKKP*RQKHTGKARTGSTRNPH*TGGGVVFGPKPNRNYNLKVNAKVRLLAFKSALTIKLNEGKMLGLVANSDLETPSTKKMVNFINNANLENQKVLLVIADHFSNIKKSTNNLQKVTTKL*YQVSVRDLMHANVVVVAEEAFTNYARKVSK*

>UUR10_RS01595 Ureaplasma_urealyticum_serovar_10_str_ATCC_33699_NC_011374 translation initiation factor IF-2

MAKKNIKQKKDNRIAIDVKKHIKKVDVGVFGGTFVFTSPLSIAELAPKLNKSTNEIIMRYFKKGVVYNLNTILDEEQIGELCLEYDLDFKIEKNVNTENLLENIAFDDLEADLVARAPIVTIMGHVDHGKTTLLDTIRKSSVTASEAGGITQHIGAYQILKGDKPITFIDTPGHEAFTEMRARGANLTDIVILVVAADDGIKMQTEEAIDHAKAANVPIIVFVNKMDKYEANPDKVLNQLSAKEIVAEELGGDIVFVKGSALKNEGIFELLDSILLIAELNDYKANPNRLAYGTTIEANLDKGHGPLATLLVQNGTLRKGDYLVVGSTYGKIRNMFDEYDNEIEMALPSKPVKVSGFEEVPTAGDKFLALADEKQARAIANDVKQKKIRLERSMLQSSDIRAKIANGELKNINLIIKADVQGSLEALKGIFNSINIEGVTTTLVRSAIGTISESDVRLAQTSDAIIIGFNVRANRIIKDLADSVGVQIMNYDIIYKFKEDLEA*MKGTLDPIIVEEVIGEAKVLKLFKHSQVGTICGCRVINGKIKRNALVRVLRDGIVIYNSKIATLQHNKDSVNEVIADKECGLTIANFNDVKENDIIEVYVKVEKNHDEVK*

>UUR10_RS01025 Ureaplasma_urealyticum_serovar_10_str_ATCC_33699_NC_011374 ribosome small subunit-dependent GTPase A

MRAKITSVIVNNFYVYIYDLKIETKAIPKGIFKHDSHELKPMVGDDIEVELVDGVYLIVKIYDRYNQLIRPKVANVDIVLVVASIVQPDLNTLTLNKYLAFYEARNVKNVAIGLSKYDLASDSLKQKVDQLILDYQRNNYKVFVLTNEHDISLLKKFIKKHTLCLAGNSGVGKSTLINKLDPSIKQRTQEISQFLNRGKHTTTSTKLISFANGFLVDTPGFGNLEVNLTKNEMANAFSDFANYARFCKFSNCLHIDEPHCAIKKAVNDDQIVN*RYDDYLKIMKKLPNDVLEIKTRNQNKK*

>UUR10_RS01285 Ureaplasma_urealyticum_serovar_10_str_ATCC_33699_NC_011374 valine--tRNA ligase

VKKKLNKNYLFKEVESNKLLF*QENNLFKAQANSTKPPFAIVLPPPNVTGHLHIGHAYDFTLPDILMRYKKLQGYDAFIVPGTDHAGIATQTKFEKILKTNEQVDRFVLGRKAFLEKLKI*KDEQTYYIHKQ*NALGLGLDYNNYLFTLDEPVVQTVREVFVKMFNENIIYRAKKLVN*DIQLKTAISNIEVIHKEIEQKLYYIKYSSEDQKDFVIVATSRPETMFGDKHLIMNPNDQRYVHLHNKIFINPINNAKMSVILDDYIDIEFGTGVMKCTPAHDFNDYELAKKHNLELINIMNEDGTLNEKCAEFKGLDRLQARALIVDKLQKSNHLVKIENYQSNVGFSERTNEIVEPYLSYQ*FIKMDNLVKNTIKMQNDFNDKVDFYPNRFNKTLLT*LENTED*CISRQL**GHQIPV*YHKKTNEIYCNTTPPKDLEN*IQDEDVLDT*FSSGM*PLLTTK*NSNDQFFKRYFPTALMVTGMDILFF*VSRMMNFSQYLVQKRPFKDVLIHGLIRDAQGKKMSKSLGNGIDPFDIINEYGLDTMRLFFASSTTVGEDLNFSTERLGAN*NYLNKI*NIAKYIENLDEINESFSIQDVHEFCDVNKWIIAELSKLSVEMNKNMDKYNLVVATKDLYDFI*NTFASNYLEYTKVLLQDTTFKNETIKTIRYVFNQILIMLHPFAPNISEEI*LNLNQTNESILLQKYPMVNFEFESIIINKIAKIILEIRKLRLQENINNKTNLCFELVSANDEFYNSNIKLINLLLVLVNAKVSEIKKSSVNSCTYELVIDDFILKT*YEKSIDYDTQIKKVSEQLKYLENEIKRATNLLNNQGFVNKAPTELIAKEKDKLNNLEKEQANLLKIFADLKQKVN*

>UUR10_RS02135 Ureaplasma_urealyticum_serovar_10_str_ATCC_33699_NC_011374 holo-ACP synthase

MKLVHGIDIIE*NREELNNPSFAKRILVDDELKYYLQLNSLKEKNRYLASIFASKEAVMKAFKLKYGYNDILILKTKNERQVYLNKILIKELVLSISYTENYVVASVVGLINTVESNS*

>UUR10_RS01195 Ureaplasma_urealyticum_serovar_10_str_ATCC_33699_NC_011374 preprotein translocase subunit SecY

MTNKQKKKNAFRQLLMIFKNKKVLVALIVTLSILILFRIGSVIPMPYIKLNGNFGNQGSFFSIINLLGGGGLSQFSLFAIGIGPYITAQIIMQLLSSELVPPLAKLSKSGERGRKKIEVITRIITLPLAVMQAVIIINLMTRANGFISIVPNAPFAIGSPLFYVTYIFLMVGGTYISLFLADLISKKGVGNGITLLILTGIVASLFNHFIAIFSNLGSLTSSKVSQIIGFILYILFYIMILIGVVFVNNSTRKIPIQQTGQALILDHEKLPFLPIKIMTAGVMPVIFASSVLAIPAQVAEFLDKQSMGYYVIHNYFIVDS*TGLAIYVVLILLFTFFFSYVQLNPPKMAEDIKKAGRFIPGVQVGMDTEKHITKVIYRVN*IGAPILAFLACLPHLVALVAKTINHGIPVIQPSTIFGGTSIIIMVTATLEL*NAIKSTSTSTSYAYQRKELETAITISVESDKSSKSQI**

>UUR10_RS00890 Ureaplasma_urealyticum_serovar_10_str_ATCC_33699_NC_011374 ribose-phosphate pyrophosphokinase

MPKNHDILLFSLSNSRQLANKIANLLKIELSPIRIDKFADGEFIVAPQVPVRGRRVIIIQSTSKPVNDSLMELLIAIDSIKRASAKAISVVIPYYGYARQDRKAKPREPITARLVAKMIESAGATSVLT*DIHSLQTQGFFDIPFDSLEAV*VLMKHYFDAYKDSSNITIVSPDYGGVKRAREISIATGATLAIVDKRRSGKNQVEINNVLGDVQGRDCVIVDDMIDTGGTILGAAKIVREKGAKSITIIATHGLFNNNARERFEQAIKDKIINKVCIADTIENEPFEGLEIVSIAPAIAKCIEIYSKGAGSMSFVHDENSKFLFTKKNNK*

>UUR10_RS01140 Ureaplasma_urealyticum_serovar_10_str_ATCC_33699_NC_011374 50S ribosomal protein L29

MSSIAQDLRKKDSLELEKIVIELKAKLLELRFAAANGEAEKLHTAKEIRKTIARALTILNERELAEKLNNKEANK*

>UUR10_RS00430 Ureaplasma_urealyticum_serovar_10_str_ATCC_33699_NC_011374 DNA topoisomerase (ATP-hydrolyzing) subunit A

MALKKPKKSRLTTEEIKQQLEGSTIKEQSITKEVETSFLDYSMSVIVARALPDVRDGFKPVHRRALFAAFENGMTHDKPYKKSAR*VGDVIGKYHPHGDQAVYQTIVRMAQEFSMRYLLVDGHGNFGSIDGDSAAAMRYTEARLSKISYELLKYIDKETVDFVPNYDASEQEPSVLPSGFPNLLTNGTTGIAVGMATNIPPHNLTEVCQAIKAYAKNHDISIPEIMEHLKGPDFPTGAEIYGDSGIINYFNTGRGSVTIRSKYEIEDIGQGRVAIVVTEIPYMVNKVNLIEKIVELVTNKQIEGISDLRDESSRDGIRIVIEVKRDVIPEVLLNKLFKTTALQTNFSVNNLALVNGVPMVLNIKEMIKYYFEHQIEVLVRRTKFDLRKAKERIHIVEGLVIAVNNIDEVIKIIKASGDDDIASKALIARFGLTELQTKAILEMRLRALTGLNIDKLKKEYEDLLLIIEDLEDILENYDRQVNIICENLDYLIEKFGDERRTEIMYGVSSHIDDEDLIPVEDIVVTMSKRGYFKRLPIDTYKNQRRGGVGVQGLKTYEDDDVEKILVANTHTDLLFFSDLGRVYRLRGHEVPLGSRQSKGIPAINFLPIEKSESILTILPIDNYEQGSLFFTTSKGIIKRANLSDFESIRANGKIAITLKEGDKLFSVMQTLGNDEVFIGASNGNVIRFNENDAREMGRIATGVKGINLEDDEYVVGTGLSSHGEYVLAVGSKGLGKLTDINDYRLTKRGAKGVNTLKVNDRTGNLVSIKVVNRDEEALIITTSGKVIRLSIQDISVIGRNTSGVKLISLENKEEVKSIAIFKKEEIDDNDDEQKTSHGNEHNLE*

>UUR10_RS01230 Ureaplasma_urealyticum_serovar_10_str_ATCC_33699_NC_011374 DNA-directed RNA polymerase subunit alpha

MRKFLKYQLDVPSINSEDKNRTVVKIAPLEIGFGDTLGNALRRICLSSIPGASMFAVKFGGYSHEFQPYEGVKEDITHIILNLKNLAIKIDELIYSEDYFNNLLIDK*PKMKINFKGPGVITAKDIVCPVGFEIVNQDLYIAEVTKPIDVEIEIFAKTGRGRVDFNTNKDFVSTLHIIATDSNYSPVLHYAYNVEMIKDSKSSMSEILTIDIATNGTISGSEAIAIAAKIMQAHLEPIMNIDKTINEMIIMREREEEEKRQNASISIDDLDLTVRAYNALKQSGINTTAELIELTKSQLEKIKNLGRKSVTEIIQKLTERSLELKKD*

>UUR10_RS03240 Ureaplasma_urealyticum_serovar_10_str_ATCC_33699_NC_011374 tRNA (guanosine(37)-N1)-methyltransferase TrmD

MKISILSLFPELYETWINHSIISNAIKNNQVTIEIINFRLYTNDKHKKVDDYQYGGGAGMVLMIEPIVSAIRAIRTPNSYVILTTPKGQVFNQELANEFVSKYDHIIIIAGHYEGFDERINYYVDAQYSIGDFVLTGGELPSMVISDAVIRLLDGVISSSSLESESFNNYLLDYPVYTRPVVFEGHQVPDVLLSGHHKNIADFRKQQQEMITKKNRPDLYQKYLNSKK*

>UUR10_RS01235 Ureaplasma_urealyticum_serovar_10_str_ATCC_33699_NC_011374 50S ribosomal protein L17

MSYINKPGKTRA*RKMVSRQQVSDVISHGSIVTTKTKAKESQRHVDHLITLAKKNTLASRRAAAAILLGTNQHSADDLLRKLFNELGPKYANRAGGYTRVIKLGNRPGDNTEEAVLQLV*

>UUR10_RS01445 Ureaplasma_urealyticum_serovar_10_str_ATCC_33699_NC_011374 elongation factor P

MATIIQAKDLRAGHTFLYKGSIYQVIENSFNKTAMREGIVKCKVKNLRTGAITVEVLTGEKVEQAIIEKSKMTFSYDDGSGYVFMDNETYEQISIPYNQLS*EKNFIEEGTEVSVMRYDGELMGVSLPDQLVVTIVEAEEAVQGNSVQNATKRA*LASK*EFQVPQFIKSGEKVIINPSNGQYVGRAK*

>UUR10_RS01155 Ureaplasma_urealyticum_serovar_10_str_ATCC_33699_NC_011374 50S ribosomal protein L24

MNRIKKGDTVVVISGKNKNKSGVVIQVNPKEQTALVEGVNKIKRHQKKDQTHEQSGIIEKEAPIRLCKLALVDPKGKDKGKATKVKYLLKDNKKVRVARKSGSELDANKK*

>UUR10_RS02205 Ureaplasma_urealyticum_serovar_10_str_ATCC_33699_NC_011374 nucleotide exchange factor GrpE

MSKNNENIKHQNEGKLHDQVDKKETKNHAKQEFKYKELYEHELKKNKELQNVNELLINKNQQLEIQINQLNQDFVKQLETKTKQAQEILEQKVNELEARHETKVNDAVFKIFKFKMEPLLDAINHFTKIVNQNYDDPKIQAFIEGFKMFSQNMIDGLENLKITKISPQINDMLNDDTMEVFEVVQNTNKPSMHVTEVISDGFKYNDKVIKFAVVKVAK*

>UUR10_RS01170 Ureaplasma_urealyticum_serovar_10_str_ATCC_33699_NC_011374 30S ribosomal protein S8

MYLDPIAELITKINNGRKAHKAEVSFATSKLKTAILELLVKEGYIKSYDIRPTENNKSETVVKLKYKNQTTSSINGFKQISKPGLRIYSTHLNLPKVLNGLGIAIITTSKGVMSDKQARKENVGGEVIAYV**

>UUR10_RS03455 Ureaplasma_urealyticum_serovar_10_str_ATCC_33699_NC_011374 50S ribosomal protein L34

MKRTFQPNNRKRAKVHGFRARMKTKNGRNVLARRRLKGRHSLTVSGEK*

>UUR10_RS01120 Ureaplasma_urealyticum_serovar_10_str_ATCC_33699_NC_011374 30S ribosomal protein S19

MSRSLKKGAYADPSLLKKVEAANASVSKKPIKT*SRRSQIFPNFVGLTFEVHNGKTFLKVYVTEDMIGHKLGEFAPTRNFKNHTEAKR*

>UUR10_RS02910 Ureaplasma_urealyticum_serovar_10_str_ATCC_33699_NC_011374 UMP kinase

MSKQRIVIKISGACLRQDDNSIIDVNKINDLAKQIKEISKKYIVSIVLGGGNI*RGHIAKELGMNRNLADNMGMMATIINGLALENALNNYNVDAIVLSAIKCDKLVYESSANNIKKAIEKEQVMIFVGGTGFPYFTTDSCAAIKAAETESSIILMGKNGVDGVYDSDPKTNPNAQFYQHITFNMALTKNLKVMDATALALCQENDINLLVFNIDKPNAIVDVLEKKIKHTIVSK*

>UUR10_RS00095 Ureaplasma_urealyticum_serovar_10_str_ATCC_33699_NC_011374 DNA polymerase III subunit delta

MKYSFANLLIQSPKTSLTLGVEQIMLAFINEKNHEQQAYYINKVKNNQYFDLKIYDSLSMKKSDVIDLQNAFLYDGIEDINLKFYLIKNIDLASKYVLNALLKFIEEPPKNTIAIFSTKNLNQVLKTIKSRCQLFYLPANYDLYHQLIKQINQPISATECDLIFDDLDELKTLLENNEINEVLAYHAKLNDIKSFETLNDLKETFKNLSILQIHYLLKLIFIKINNINSKQAILDLMRANLKININKNSLFTIIYTIIIENRGD*

>UUR10_RS03255 Ureaplasma_urealyticum_serovar_10_str_ATCC_33699_NC_011374 ribonuclease J

MENAKKSPTYVYALGGLEEIGKNTYVVEHEDEIILIDAGIKFANASLPGFDGTVANFEYLIKNNHKIHSLVVTHGHEDHIGGIPHILRHVNIKTIYAPTLAAKLIERRLSEYKDIKPPRIIIFEDESMYKTKHFEVDFYRVCHSIPDSFGICVKTPNGYIVTTGDFRFDFATAGDETNLAKISQIANRGISVLMCESTSAEIPGFSESERYVIDNIRDYMVNIKGRTFISTFASNLGRVEEIIAIAVGLNKKICIIGKSMEANIKTSRKLGYLNVPESSFITHKELPFYKDHEIVVILTGSQGEKMAALNVMANNNHSKITLKPSDTIILSSNPIPGNYAQVEAMVNKLYKLGLTVYENSPNKKIHASGHATRSEHQLMIKAINPSYLFPIHGEYKMFRALKQNAVDQGFDKDHVIIATNGQKLQLLDGVLSHSNIHVDAEPKFINGYEISSKISKLLSERVVLSSDGILNLVLNADFKKAKLNSAVSISTRGCFFAKESTNLINKISNVAKSSLEDALAKKEFDEKKLKEIVSGSVKSIV*K*RKKNPIINITIINNDLVEQFRKDNNYVEFIKQTEVEEIEQEVDIDDLISNGL*

>UUR10_RS02915 Ureaplasma_urealyticum_serovar_10_str_ATCC_33699_NC_011374 elongation factor Ts

MTKAELVKELRTRTQASMSECIKALDASENDIEKAII*LRENGAIKAANKLKNAATDGVTLAKKVGNKAILIEVNCQTDFVAKNENFLAYANQILEEALAKVESKEDFDKLIINGKPIAESGLDLTAYIGEKIVFRRGEILKANDQQTLGVYTHNNNRVAAIILVDGKVEDEVVRNVAMHAAAMRPRYLNEQVVDQV*LAKEREIIVNQLEHEGKPAAFAAKIIEGRLNKILKENCLVDQSYFKQPELTIEKYLKNNNAVAVGYYSYEVGEGIEKAPQMSFADEVAAQMKK*

>UUR10_RS00740 Ureaplasma_urealyticum_serovar_10_str_ATCC_33699_NC_011374 F0F1 ATP synthase subunit A

MENYNPLDIMIALPHIAAIIIVTLIIATISLIYFSMIRKLTVHDVPNRFVIIIGMIVDYFRGLVVDTMGAKHVKLAPYVLFTFCYIFTANLVSLFGFKEATTASSVPLAMALATVVGGQIVALKYQKASFFLKFTFKIKGFPIMVNPLEIVSKLTPIISLTFRLWGNISAAAILLNITY*AFAGFTNVVP*VGVSLIAAVIILPILIGYFTCFAGTIQAFVFTLLTSIN*GLEIKEGEEHYAHLAHKKAEKLAAKKLAELDAQNQAQNNEVQVVL*

>UUR10_RS01130 Ureaplasma_urealyticum_serovar_10_str_ATCC_33699_NC_011374 30S ribosomal protein S3

MGQKVNPNGLRFGINKQ*LSR*VPTDQLQMAK*LVEDDKIRKYLSTKYKNAGIDHVEIERDQQRVNVYVYAVQSGLLIGTEASEKKLIELAINKIVGRKQLVSLKVVEVQIPELQASLMAREIADAIENRVSFRIAQKMVIKKVLKAGARGIKTHVSGRLGGVEMAREEGYTQGVMTLHTLRADIDYSMQEAHTTYGIIGVKV*INRGELFGNKLVNSVAHAANKEFSRSSKPKKGSFNRSSRSKNTKPAPKQAVSE*

>UUR10_RS02400 Ureaplasma_urealyticum_serovar_10_str_ATCC_33699_NC_011374 cysteine desulfurase

MDNYKQFFP*FKNNKDVVYLDSSATSLKPQVVVDAIVDYYTKYSTNPHNSDSNFAFHPHKIMYETRANVAKFINADFEEIVFTSGATESLNLIANGLRPYLKKDDEIVLTYVEHASNLLP*YKLRDDLGIKIVFANQKNQFPQLSDFLNAISPKTKIVSFASGGNLIGNILDENVIIKHIKQLNPNILVCVDATQSVQHRMFDVEKCQSDFMVFSAHKLLGPTGIGVAYIKNEWIKKLQPLKYGGGMNFSIDLDSYQLYDDYMKFEGGTPHVAGFYGFNAALKFLMDIGYEKIHDHELKITQYAREQLALIPQIKTYVQDPTSSTITFSYEGVFCQDFASYLGTKNIIVRSGLSCAKIINNIIQTECAIRASFYIYNDFSDVDKLVQAIKEYQKGDELNGIL*

>UUR10_RS00850 Ureaplasma_urealyticum_serovar_10_str_ATCC_33699_NC_011374 phosphopyruvate hydratase

MKIVDLLAYQVLDSRGQPTVAVKLFLENDQSVVAMVPSGASTGTKEALELRDGDANYFFSKSVKLAIQNVNNIIRPHLLNKNVLNFFELDNLLINLDGTENKTKLGANALLGVSIVIVKGGAVAASKPLYQYIKEDLMHNYDEHYYAPIPLMNFINGGAHADNNLDIQEFMIVPLNAISFSQAIQTGSEIFHELAKILKANHLNTAKGDEGGFAPMLNDNYAALELLVRAIKKAHYFPSKKQGVCLALDVASSELYENEKYVFKKALSHNTNLEQTSFSSDE*AKY*SDLASQFPIISIEDCFDEND*NGFSLFLKNNPHIQSVGDDLYCTNLKYLQKGINFKATNAILIKPNQIGTISETLDVIKYAQENNINTIISHRSGETEDTFIADFAIGVGAGQIKTGSLSRSERIAKYNRILEIEQELKDKLVYEPNKFFKFN*

>UUR10_RS00730 Ureaplasma_urealyticum_serovar_10_str_ATCC_33699_NC_011374 F0F1 ATP synthase subunit B

MLDKRREYIAKEITDAENAKQEALQYLENAKSEHLAAQAETAEIIAKAKSESLTLRELLEKEAREAADKIISSAKISIANERRENLERLQTEAREAAYIAAEALMKKELSREDNDKLVDQFIKELETNEK*

>UUR10_RS00855 Ureaplasma_urealyticum_serovar_10_str_ATCC_33699_NC_011374 6-phosphofructokinase

MSQTSFLNSTKNILIITSGGDAPGMNASLVSLIHELMDSNFNVFVGIEGLLGLYNNLIEPIKNKHIFDVYFKEQGTIIKTSRFIKLNVNDEKTQVIKKNLLEHNIHKIIILGGQGSMQAGLVLTDLGFEVYGILHTIDNDFNQTQMCIGASSAAHFNQQLLTCLNYTAKAHNAFSLVEIMGHQCP*LVNNSIGQLKPILTLTNQDPKYSVDQVIDLVKTKITLAKEYDPLIIVQELIYDQQ*YEALKKAFAQKLHQTLRVTILNYLQRGAPVIDFDLQLAKDSASVLVDFIINKNEIENTSNMYVVVNKNDIKPQVIKFND*

>UUR10_RS02250 Ureaplasma_urealyticum_serovar_10_str_ATCC_33699_NC_011374 5-3 exonuclease

MKKAIVIDGNSLIYRAFHATYKQAE*AVENQLMPTNAIKLVASMIFKILNEDQFSYALIALDASKKTFRAQEYAAYKATRKPMDEKLVVQLPYIKKLFTAMGFHIISQPGIEADDFVGSFSNLMSKSNIDTIIYSTDRDMLQLINPNTKLKLLKTGTSIVQEINLANFALLNNGLLPKQIIDYKGLVGDSSDNLVGVKGIGPKTAINLILKYTNLENIYANLEEITPSVKNKLIEHEKMAFLSKKIATIQTDLLLDETLENFILKPYNIQELDTLFESLKINNMHNYYK*

>UUR10_RS00210 Ureaplasma_urealyticum_serovar_10_str_ATCC_33699_NC_011374 tRNA uridine-5-carboxymethylaminomethyl(34) synthesis enzyme MnmG

VKKYDVIVIGAGHAGLEAAFATSNLNLQTALITLDEKGIGMMPCNPSIGGPAKGIVTREIDALGGIQGKAADATTMQMKILNSSKGPGV*AIRAQIDKIAYQR*FKQQIKQQKNLDLIIAEVSDLLVENNIVKGVILSDQKIIQADYVIITTGTYLKSITHRGSVCVDEGADGTKNAKFLSDVLVKLGFELIRLKTGTPARIKKDSIDFTNMVLEPGTNQKIAFSHYHPVYKPYDKQLPCHIIYTNEQTHQIIRENLNKSAMYGGMISGIGPRYCPSIEDKIVKFSEKPRHQIFVEPESYELDSMYLGGFSTSMPIDVQEKMIRSLPGLEDCEILKYAYAIEYDAIDPTQLYPSLESKLVNNLFFAGQINGTSGYEEAAAQGLMAAINVSQKHKNKEPIVLGRDQAYIGVMIDDIVTKGVVEPYRLLTSRAEHRLALRNDNADDRLMKIGFEIGLLKPEVYDQYLNNLKQINEVLN*LKTTTVGQIDDLKFTTLKTNSYLIDYLKRPEVKLNDLLIYCPIKIEDEQIINKVQIQVKFEGYIKNQEENLKQLKRLNNIKLHGIVDYKEVPNISLETIDKLNKIKPLDLEQASRISGVNLTDIAMIKYYLERIKND*

>UUR10_RS00410 Ureaplasma_urealyticum_serovar_10_str_ATCC_33699_NC_011374 aminoacyl-tRNA hydrolase

VEKYLIVGLGNPGSNYAKTRHNAGFMVINEICNKLNLFLDNSKFNGMFAKTIYNNCVVFFCQPTTYMNLSGEFVSKMLKFYDIPIKNLIVIYDDVDTKLGVIKLRKKGSSGGQNGIKNIINLLKTEEIKRIRVGIGKDPHAKLDQYVLSNFKIDELVIIKPAIIKGALAALEAIGEDFDKVMNKFN*

>UUR10_RS01270 Ureaplasma_urealyticum_serovar_10_str_ATCC_33699_NC_011374 cation-translocating P-type ATPase

VKKNNEDQTDSFVSFDPQNTDPLTGLNDEQVLKSRQIYGFNEIKKKKKSNILTKFFKQFLDFMVILLVIAGIITLILAIVKPPHDITELIVQYVEVGVIGFILFLNAIFGTIQEVKAEKNTEALSKLTSPQAKVLRNNQILIIDSREVVIGDILILEAGD*IPADALLINSSSLEVDEAVLTGESLPVQKDAKAIVKQGAGIGDRLNQIFSGTSITNGTAKAIVTNIGMNTEIGKIAKLINDQKVQLTPLQQKINKLSKIIGAFASVLCIAVFIIYIYLVGGGN*EIN*HPALVMAISLSIAAIPEGIVAIVTIILSFGVKQMAKKNALIKRLPAVETLGSANVICSDKTGTLTQNKMTVTKVFTNILKTTDLINEKDVYELIK*ASIANNGSRNFNDKKQEYEFIGDPTETSIIEAALKLNIDKSELDKEFVRIHEFPFDSTRKLMSVIVRNNDNYYLVTKGAIDAIEKIVVEPITNDVYKANDFLGKQALRVLGVGIKKLAFLPTNFNQDELERELEFIGLVGMIDPPRPEAQEAVEIAIKAGIRPVMITGDHINTASAIAKQIGILNEGQEVLSGHELSSMSDEELINNVERYSVYARVSPTDKIRIVKA*QSHDKVVSMTGDGVNDAPALKAADIGCAMGITGTDVSKASSDMILTDDNFATIINAVSLGRSIMDNIKRIIVLLLITNLAGLISLIFGIIILGINPMSSLQIL*INVIAETLPGIALGVHLADANLMRHKPLKKSAPIVNKKM*MTIFINGFFIGLISILLFYLGASSHFDFDFIAMRNEFKELANLEAIYQNV*N*LGENHEITNIVHEKIIAIKTPIMAGSSLTFIFMGMSLAFNALSLRSNHSIFINF*KNSKYIVYSIIISVIMIIVITYTPHLNEVFNMNPYNMNGYE*FNVFPFVLFTIPLGIFEVIKYVKYLKLRRSFDYKNKTYASLNQEIKSLNLKINNTKINYEKEYYKALLNNLIVKRKILINKCHKEI*

>UUR10_RS01005 Ureaplasma_urealyticum_serovar_10_str_ATCC_33699_NC_011374 guanylate kinase

MKRGKLIVFSGPSGVGKHTILSKIIDRKELNLAYSVSMTTRKKREGEINGVDYYFVNDEEFKKAISNNELIE*AEFVGNKYGTPRFVVEKLRNEGKNVILEIEVVGALQVLELFKNDDLISIFLLPPSLDELKNRLLKRNTETLETIEKRIQKASHELSIKDHYKYNIINDNPDHAANQLAEIILDEIKR*

>UUR10_RS01585 Ureaplasma_urealyticum_serovar_10_str_ATCC_33699_NC_011374 transcription termination/antitermination protein NusA

MSNSFKSKEFIEYFKDTAKQNEIELEVLSSIIKEAFEKTYLRTHPGENFETNINLKEGTINCFRNLVVVENEKAHNEDLETCLDDAVEILLDDARKINANAQIGDTIKQYISIDDFKSIEVGQIGSLLRQKITEIHNKRVADF*KPSLMKMIRAKVAEINYNKQRNEITGVKVELDDQ*KTLGYLSRKDRIGDEKFKVGETYDFIIKEVKEQSRL*PVLLSRTEPELVEEILKREVVDIKNGNIEIKKIARIAGFKTKVAVSTNLLNIEPVAVVVGNKGLTITSISKQLNNERIDVIRYADDKRIFIANAIGLDKLKGLLVQENESDQRSAIAIVSKEDLPSVIGRGGANIRLIAKITE*NIDVKTIEQAFEENVVYEKFDEKIYRS*NIESINKKNVTNDEMLALIDNMQDEKVEKTEQVKDQLKQQEKQTIVSNNDDSENDDEQLEYLEGFEDFKF*

>UUR10_RS00835 Ureaplasma_urealyticum_serovar_10_str_ATCC_33699_NC_011374 triose-phosphate isomerase

MVKMKYIIANFKMNATEELINHFLNNLISFDEQKLTIGLAPGDLYLKTFVDLSQTKKVKLYAQNPSAYSKGPYTGQISCLQLLDSNIKNTLVGHSEIRIDCSQSIIDQKTKICMDLLDQVIICIGEPLDVYEQKKSLSFVLSQLANVINYKGLKKIIIAYEPI*AIGTNLTLDLKHINHMIEGIKTYLYNCTGLNIPILYGGSVNANNIKELCTQKLIDGFLIGNASLDVNNFNQIINACK*

>UUR10_RS01665 Ureaplasma_urealyticum_serovar_10_str_ATCC_33699_NC_011374 50S ribosomal protein L32

MAVQQRRVSKSRKGMRRSHDHLTVSNTVACNECGKALLPHRACRDCKTYRSIKLSIK*

>UUR10_RS03000 Ureaplasma_urealyticum_serovar_10_str_ATCC_33699_NC_011374 30S ribosomal protein S7

MRKLKPQKRQVLADPVYNSRLVTKLINAIMYDGKKGLAQSIIYSAFEIVEQKTGKPALEVFNKAIDNVMPIIELKVRRVGGSNFQVPTEVTPERRQTLGLR*ITLYARLRHEHTMIEKLAHEIIDASNNVGAAIKKKEDTHKMAEANKAFAHLR**

>UUR10_RS03395 Ureaplasma_urealyticum_serovar_10_str_ATCC_33699_NC_011374 ribosome biogenesis GTPase YlqF

MDIINKKIN*FPGHMKKATDEILKNLKNVDFFIQLVDARCPITSSNNELIKQIASKPIINLANKADLSD*NTNFNNDFLLISTKKVNDKNLVIKHLYQLFEQKIKTYQKKGLVNPKFIGMIIGLPNIGKSSLINFLAPKKTLKVENRPGVTKTQSIRQINQHFYLIDTPGIFLKDIQKERDGFVLTLINCIKKEVLELEAIIRFAYEFYLKNYQKDLFIRYKINQVMNFEDFIDYICQLYNYKLVNNEFDYSRAYENLFNDFCNGLICKVNYDQ*

>UUR10_RS02195 Ureaplasma_urealyticum_serovar_10_str_ATCC_33699_NC_011374 glycerol-3-phosphate acyltransferase

MDQVYSVAMAYILTLIISPLYSYLIGSLNASIILSLLLKKQDIRHFASKNAGMTNMTRVYGKKLGILTLFLDIVKPIITISLTYIIYKYALNAPFVLSNGFNQAILVYFGGIFTIIGHCYPIFFKFQGGKGVASYGGFLITIDPIVAVIGIITLLIILLITKYMSLSAMITATITCFLVLIPGINYIPYYNEHFVEYLFDLNHVIKGTWYV*LFLLISASILIYRHKTNILSIATKQERKTFLFQPKPKNNI*

>UUR10_RS03135 Ureaplasma_urealyticum_serovar_10_str_ATCC_33699_NC_011374 AAA family ATPase

MSTKDLNDAILDLFCLVINNNDF*KDVILRLEAKDFPEKVQQNIFNTIANLNEQKYKISESNILNGLGNYVIVDEQDQNYLLHKNYLVQILERTDYLVDLKDCIEIIKNASIKNKLDLFANEILSTQISLTNAKDQFKEMHEKFLEILASRTEDTIENMELIANRYFEKLNKIGNSGIIPGVIKTKYDNIDKFTNGYKPGELVVIAARPGIGKTTFCLNVMVNNVNEIIEYNQNIQPNQKEKIIVMFSLEITKEQILQKFISIKTGISNREVIENKYRIAKGYDTRSFAMQAINEIKS*PIFVDDRPNISIVDIEAKLYDLKKRYDIALVVLDYLQLVSAGNANKNMTRTQEVGRVSSALKVIAKEINAPVIAIAQLSRKAEERDVSSNANMKNNPLVKTIDNSPKLSDLRESGSIEQDADVVAFLH*DRKQRNAMQNDNQETRMRDDLIEAKFIVEKNRNGSTGETDIIFSKLNSKFIRATTSKE*

>UUR10_RS00955 Ureaplasma_urealyticum_serovar_10_str_ATCC_33699_NC_011374 ribonuclease III

MDNKKFLDFLKQNRIEPKNLSIYLEALTHKSYANEHKLTKNYQRLEFLGDACVEWVISNFIFNYKIKDNEKMRSLDEGEMTRARSNMVRSEILSYAAKDLGLTDFLMIGVGLEQDQSARMEKIYEDIFEAFIGAVAQDQGIKKVSLILEKTLIKYFREGQINYQKDYKTIFQEQAQRINKKPIMYKLVRNEGDKKEVHLV*NDLIYGIGIASTRKEAEILAAKNAILKLDDYTKKA*

>UUR10_RS02540 Ureaplasma_urealyticum_serovar_10_str_ATCC_33699_NC_011374 DNA topoisomerase IV subunit B

MANKYDGSAIKILEGLEAVRKRPGMYIGSTSSAGLHHLV*EIVDNSIDEVMNANAKNISVVLHEDNSISILDDGRGIPVDINPQTKISTVETVLTVLHAGGKFDESAYKTAGGLHGVGSSVVNALSA*LICEVYRDQKIYQAKFSNGGHIDQPLKVIGTTKKTGTLIHFLPDPLIFKNLFFNPNTIKERLHESTFLIKDLKISFEDKINNKKYEFINDQGLIDFIKFINETKKTFSDVIFFKNTINKIDVEVAFQYSDQNNEIMVSFANSVKTSEGGVHENAFKNALTSVVNNYARKHDLLKEKDKNLEGDDIREGLSSVISLRIPESLISYEGQTKNKLFTPEANEAVKKTIEDNFSF*LEENKTQALDLVNRAIVARDAKLAAKRAREETKKVKKIKEERGMGGKLTPAQSKDPTLNELFLVEGDSAGGSAKLGRNKKYQAILPLRGKVLNVLKARLVDVLKNEEIASIFTCLGTGIGAEFDLKKLKYHKIIIMTDADTDGSHIQVLLLTLFYRFMRPLIENGNIYIALPPLYKLTNKNTKKFFYA*DDVELDQLKKEQKNYEIQRYKGLGEMNADQLFETTMDPSKRLLLRVNINDILQAERQINTLMGNDVSIRRQ*IDNNIDFSVIDELQINNEESK*

>UUR10_RS02905 Ureaplasma_urealyticum_serovar_10_str_ATCC_33699_NC_011374 ribosome recycling factor

MNFKIYETKIREEFELVLK*MHNEFIKLRTGRATPAILDGILVDYYGSMTPINQLANISVPEPRVLAIKPYDRSSIKDVASAINASNLGVNPQVDVDIIRLTFAAPTEEVRKNLAKKAKQVGEEAKIRVRHIRQEAQDLFKKNSSTVEDDKKFFQTELDNLTKELNKEIEAVVSHKEKDIMTV*

>UUR10_RS02235 Ureaplasma_urealyticum_serovar_10_str_ATCC_33699_NC_011374 methionine adenosyltransferase

MQYKKIITSESVGAGHPDKICDQISDAILDECLSQDQNSRVACEVLACNRLIVIAGEITTHAYVDVVKTA*EIIKPLGYDENDFTIISNVNKQSVDIAQSVDKTNKNLIGAGDQGIVFGYACDETPQYMPLTSVLAHELLKEIERQRRSKEFIKIQADMKSQVSIDYSNSTPLIETMLVSIQHDEDYDVEYFNKKVSAIMEQIAKKYNLNTNFKKIINSSGRFVIGGPIGDTGLTGRKIIVDTYGGVGHHGGGAFSGKDPTKVDRSASYFAR*IAKNVVAAKLAKQCEIQLAFAIGQPQPVAMYVNTFNTNLIDETKIFEAIKKSFNFDIKTFINDLNL*TTKYLPVATYGHFGRDDLDLS*EKLNKVEDLIKNSK*

>UUR10_RS01205 Ureaplasma_urealyticum_serovar_10_str_ATCC_33699_NC_011374 type I methionyl aminopeptidase

MVIVKTEKDITAIKEAVRI*KIAREAIYEQVKAGVSLKELDLLAKEVIEANGGIAAFHNYLGFKGHICISVNECVIHGVPTDYILKDGDKVTFDVGVKYDNHYCDAAFTIIINNSNVEALKMSDICKKSIDEAVAIIKPKVTTHAISNVIQKFIEKNGYFILRDFAGHGCGNEIHEDPLIPNYRSLLYRNVTLEENMVICIEPMILSGSNAYYIDPNDQ*SVKSKNHQMTCH*EHMILITKDGCEVLTA*

>UUR10_RS01370 Ureaplasma_urealyticum_serovar_10_str_ATCC_33699_NC_011374 HU family DNA-binding protein

MSEKIKAKTRVQMIDELSKMLNIDKKQTKTFMDTYEAFLILELSRAKEVRFGNIGKFKVTVRAERKGINPKTGETVIIPEKTIPKFTFTKGIKEIINAGISVEDETVFLDDNDYEDDGDEFVEEYIAPESN*

>UUR10_RS03415 Ureaplasma_urealyticum_serovar_10_str_ATCC_33699_NC_011374 class II fructose-1,6-bisphosphate aldolase

MFSLVNAKKMVQNAYKNHYAIAAININNLE*IKAALLAAQETNSPLLLATSEGAVKYMGGYDNCYAMVVNLMKQMNIKTPVCLHLDHGTYEGCIKAIDAGYSSIMYDGSKISIQENIENTKKLLAIAKSKNVSVEVEVGSIGGTEDGITSEGELANVNDCYQMCLLDIDMLACGIGNIHGLYPEN*KGLNFDLLKEINIKVNKPIVLHGGSGISEEQILKAISLGVAKININTECQIAFSNALQDHLIKAGDLVAAKQYDPRKVLAYGVDAIKNTIIEKFTKFNSLNKA*

>UUR10_RS03055 Ureaplasma_urealyticum_serovar_10_str_ATCC_33699_NC_011374 50S ribosomal protein L33

MAIKRGVRLQCNESKSINYITTKNAKNNPDKLSLNKFCPKCRKVTTHVEIKKK*

>UUR10_RS00425 Ureaplasma_urealyticum_serovar_10_str_ATCC_33699_NC_011374 DNA topoisomerase (ATP-hydrolyzing) subunit B

MNDSNKENKYTAESIKVLEGLEAVRKRPGMYIGSTQSEGLHHMI*EIVDNSIDEAMGGFATVVKVIIKKDGVIRVEDDGRGIPVGIHEKTGLSGVETVLTVLHAGGKFDNDSYKVSGGLHGVGASVVNALSKNFKV*VNKNYVQHYVEFINGGHAIEPLKIINDKDIKEKGTTIEFIPDFEIMEENE*DELKIMARLKQLAYLNKGVNIEFESEMTNRKEK*HYEGGLKEYIADLNAEKEPLFDAIVYGEEEKEVKVPGHNDQTYNIKCEVAFQYNNSYNNSTHSFCNNINTTEGGTHEEGFKLAITRLLNKYAIDKKYLKDTDDKITKEDVSEGLTAIISIKHPNPQYEGQTKKKLGNSEVRPYVNEITSIIFEKFLNENPEESKKIVAKVMQAAEARRRSHEAREATRRKSPFESNSLPGKLADCSNRDSSVTEIYIVEGDSAGGSAKTGREREFQAILPLRGKIINVEKAKIDKIFANEEIQNMITAFGAGIGPEFNIEKLRYSKIIIMTDADVDGSHIRILLLTFFYRYMLPLIQNGNVYIAQPPLYKVSYGKTIKYAYSDQELEKIKSTLLNTKYNIQRYKGLGEMNPDQL*ETTMDPKNRLLLKVNIEDAAIADKTFSLLMGDDVTPRKEFIEKNAKYVKNIDA*

>UUR10_RS00640 Ureaplasma_urealyticum_serovar_10_str_ATCC_33699_NC_011374 tyrosine--tRNA ligase

MHNLIKDLKARNLINNITNEEKLKKALAENKGIYVGFDPSADSLHLGNYIMIMLLKRFRLHNIKTFALVGGATGMIGDPSGKSAERNLLDKTILEHNITKIKYQLEKFTNSQVINNYDFYKNMTFLDFLRDVGKLININYLLEKEIISSRLDVGISYTEFSYNLLQGYDFLQLYKNDNIAIQAGGSDQ*GNITTGIEIIRKSLGDDNIACGLTINLLTNSEGKKFGKSEKGAIYLDENKSSVYEMYQFLINQTDADVEKLLNFLTLIDVDEINKIMQAHKENPALRIAQKALAQAVVVDVHGQQKYEQALHISQVLFNGNINELNQEEFNIAIKSLPTTKLDKDEIKIIDLLNLANISSSNRVARDFLSTGSILVNDIKVNDENFLVKKQDAINQEFSIIKKGKRNYFLIV*NKD*

>UUR10_RS01695 Ureaplasma_urealyticum_serovar_10_str_ATCC_33699_NC_011374 molecular chaperone DnaK

MTKEIILGIDLGTTNSCVAVIENKKPIVLENPEGKRTVPSVVSFNGDEVLVGDAAKRKQITNPNTVSSIKRLMGTKEKVTILNKEYTPEEISAKILSYIKDYAEKKLGTKINKAVITVPAYFDDAQRQATKNAGIIAGLTVERIINEPTAAALAYGIDKLDKEQKILVFDLGGGTFDVSVLDMADGTFEVLSTSGDNHLGGDD*DQVIIN*LLKSIADEFNIDLSKNKMAMQRLKDAAEKAKIELSGVNTTTISLPFIAMDSSGQPINFEKELNRATFDNLTKNLIERLKKPVLDAMKESKLSLADIDQVLMVGGSTRMPAVQNLVKELTGKEPNHSLNPDEVVAIGAAIQGGVLAGEIDDILLLDVTPLTLSIETMGGVATPLIPRNTKIPVSKSQVFSTAADNQPSVDIRIVQGERSLAADNKLLGNFELSGIEPAPRGVPQIEIKFNIDANGIMSVNAKDLKTQKETSITIKDSQGLSQEEIDKMIKEAEENKEKDAKVKHERELVNRADSLINQLEQVVKTENVPQEQKDAFNKQIEELTNARDAQDYTKLEAEVKKVEDLLANAAKFAQQTQQQDPNNQKDDVTEATVTDDSTKK*

>UUR10_RS02395 Ureaplasma_urealyticum_serovar_10_str_ATCC_33699_NC_011374 SUF system NifU family Fe-S cluster assembly protein

MASYNINDNLTLRSIIMEHYERPKNKVCFVQNETDYLSCHNTTEGCSDDITVYVKLVNQKIVDVVFLGTGCAISTSSTDIICELVKNQDLQQALELINNYLNMIQGLEYNQDIMQELIAFHNVKNQMNRIRCARIGINALKTCLEQYKQ*

>UUR10_RS03150 Ureaplasma_urealyticum_serovar_10_str_ATCC_33699_NC_011374 single-stranded DNA-binding protein

MNKVILIGNLVRDPEARQIPSGRLVTNFTVAVNDNIPNANANFIRCVA*NNQANFLTTYLKKGDAIAIEGRIVSRSYVDNNGKTNYVTEVYADQVQSLSRRNQNANDHNNDKVNVDTMMGAYASINTDAAFSSNQPQTNFQSTTSNSNKNDDEEDEITS*INLDDDLE*

>UUR10_RS03305 Ureaplasma_urealyticum_serovar_10_str_ATCC_33699_NC_011374 50S ribosomal protein L13

MQKSSMLKKEAAIARRQ*YLVDATDLVLGRLSVKVADILRGKNKVDYTPNVDAGDYVIIVNSDKVVLTGQKALREN*YNHSHYIGGLRTRSGEEMISKYSDELIRRSVKGMLPKNKLSKQILNKLFIYKNDKHSHEAQQPTILELKLK*

>UUR10_RS02085 Ureaplasma_urealyticum_serovar_10_str_ATCC_33699_NC_011374 ribosome biogenesis GTPase Der

MRTIAIVGKPNVGKSSLFNRILMRRKSIVDDQPGVTRDRIYDVGN*LTRDFMLIDTGGIISSEDTYQDNINEQVLFAINEANTIIFLVSAKDGINNDDKKIAKMLKEKAKDKKVILVVNKVESEKYYFNEGELYSFGFGKFFKISAEHGIGMGDLLDELVKDMPIQNALDQQERFKFCIIGRPNVGKSSLTNTILGEQRMIVNAEAGSTRDSIDNDFSYHNKKYTIIDTAGVRRKGKIVEAVEKYAVLRTQKAIERSQLILLVLDGSEPFKEQDEVVGGLAYDANIPTIIVVNK*DNIVNKNSHTMEMVKKQIRSQFKYLS*APIVFISALDNKRIHTIFETIELVREQAMRKVATSLLNDVVIKANAFQEPPPFKGGRISISYVVQVQSQIPTFVLKCNNPKFLHFSYARYIENEIRKAFGFDSVPITLY*QDKNKKLRGE*

>UUR10_RS02545 Ureaplasma_urealyticum_serovar_10_str_ATCC_33699_NC_011374 DNA topoisomerase IV subunit A

MSVNQQKIINTPLDNIVGESYAKYAKYIIQDRALPDIRDGLKPVQRRILYAMSELGIFHDKPYKKSARTVGEVIGKYHPHGDSSIYEAMVRMSQD*KNNLCLLDMHGNKGSIDGDNAAAMRYTETRLSKIASVMLTNLKKDVVKFSPNFDDSEKEPSILPSLFPNLLINGATGIASGYATSIPPHNPNEVFDALIYRIDHPDCSIEKLIKICPAPDFPTGGEIHDLNGCANAHKTGEGKFVIRASIEFKTSEAKINQIIINSIPYETNKALIIKEIEDIIYNKEVAGLIEVRDESDAKGVSIIIDTKKDVNLENVKNYLYKKTSLEISYNTKFIAIVHRTPTLVSLSTYLDAQINHSLDVINKVDLYDLNKVLLRIEIVEGLIKCVDLIDEIIKIIRASDSRQDAKNALIQTFAFTNNQAEAIIMMRLHNLTRTDIFDLRNE*ESLQQQAKTLKERIKSLQVRKNYLKQKMIEFKKEFGYQRKTKLFDEFIKAEVNEDQMIEKQSLNLVISRDGYIKTVSKKSFESSKYDELGLKTNDLLFYHNVINSHDRILIITSKAKLINLIAHKISCMR*KDVGEHLNNYAKFDANEKVVAVYVCNEQFKVDEHQLVLGSKLNLIKRIELNELDLNKNSKQISIMKLNENDGLISANLIKKDHNQFVVAISKLGLVLMFLVHEINCLNRLAKGIKIMKLKPNDEISSILIVPNNGYSIQLFLDQGNKCFSISELKLSKRAMTPSPLYLPTKKAQSVLAAFLVGNENVFYLLDEQQKINPYYLPNLKPIKLDSKINKYENDLIITDVVKDSFLSDSVISDFKKISMYANEFDSELLKTNENQEQDDLQLELINEKEEND*

>UUR10_RS03005 Ureaplasma_urealyticum_serovar_10_str_ATCC_33699_NC_011374 30S ribosomal protein S12

MPTIAQLIRNKRAPKVKKTKSPALLFTYNSLHKKTTKNPSPLKSGVCTRVGTMTPKKPNSALRKYAKVRLSNGFEVLAYIPGEGHNLQEHSVVVIRGGRVKDLPGVRYHIVRGAGDASGVEKRRQQRSLYGAKRPKKEASK*

>UUR10_RS01280 Ureaplasma_urealyticum_serovar_10_str_ATCC_33699_NC_011374 YihA family ribosome biogenesis GTP-binding protein

MAKFIKSAQYFDQYPVDKQFEICVIGRSNVGKSSLINALANEKIARTSNTPGRTQLVNFFDFNSFRLVDLPGYGFARVSKDKQLDLATIIDQYLGYRQNLCAVFQICDINVLTNDDVEMSRYFENQNYAHFVVLNKVDKVNKSHFDNNKQKIAKFLNISVDRLLCVSAQKNTNVATLFALMKKVVIETRQKQLLLKKEEKKSSEEEIK*

>UUR10_RS01830 Ureaplasma_urealyticum_serovar_10_str_ATCC_33699_NC_011374 CDP-diacylglycerol--glycerol-3-phosphate 3-phosphatidyltransferase

MAKLNFKKNILTSPFFRNIPNIITIFRIFLALICIILLLVDFYTKNLNIVYEVLDANISALRLSATTIFIIAAFSDFLDGYIARKYNLVSNLGKILDPISDKILVNGVLICLTLDHTALAYLTIINILRDIFIDGLRMFASSKKIIIPANIFGKIKSILLFISICFILFLLSLTSS*KNVYLFNIPLFFATSLSIVSAIIYYIDFYKGVKKRGSITKS*

>UUR10_RS01190 Ureaplasma_urealyticum_serovar_10_str_ATCC_33699_NC_011374 50S ribosomal protein L15

MQLHNLEYKKGSRNHKEKRVGRGHGSGLGKTSGRGQDGQKARKSGMVRLAFEGGQTPLYRRVPKVGFNNDRFANKYNVVTLISLVKYETKELTAEFMYVNKIAKNEDLPIKVIGNAVLPSGTVVSAHKFSKGALESISNSKAKAQILE*

>UUR10_RS00405 Ureaplasma_urealyticum_serovar_10_str_ATCC_33699_NC_011374 tRNA lysidine(34) synthetase TilS

LTKL*TNLINKITNKKYLAAVSGGPDSMAMLNMYKRNISVVCHVNYHKRESADRDQEIVVDFCKKNNLPIEILDVDEKVYEKYAHIDNFQAKARLIRYDFFKEIGKKYNIQHLYIAHNFDDFLETAYMQRARQSKALFYGIKESNVVNGMIVKRPVLFIRKQTLQRYCDENKIKYGIDETNELDIYERNRVRKTISN*SLNEVYDFKKAVLKYNKEHSSFANFVELSYIEFKKNKYRYDYFVRQDDGVQYYLIYYFLIDQKISNPNENKIISLIKFFGKQINKEKAYRVQENLYMHVNEDDLISLISYDKNDVIDDPNIIEKQAGN*

>UUR10_RS02995 Ureaplasma_urealyticum_serovar_10_str_ATCC_33699_NC_011374 elongation factor G

MSKELKLFRNFGIMAHIDAGKTTTSERILYHTGKNHKIGETHDGAATMD*MAQEKERGITITSAATYAK*KGHSLNLIDTPGHVDFTVEVERSLRVLDGAVAVLDGQNGVEPQTETV*RQATKYNVPRIVFVNKMDKTGADFYYSIETMKNRLGVKATAIQIPIGAEADFVGSIDLIEMKAYIYDGQADEEYKIEDIPADYVTKAQVMRSQMIDDVAIFDDEVMEKYLSGEELSHEDIKKCIRKGVISTELYPVLCGTAFKNKGVKKLLDAVVDFLPSPIDVPPIKGVDDHGNPIEYHNDPSEPFAALAFKVATDPFVGRLTYIRVYSGKLDKGTYVYNATKDKKERISRLVKMHSNNRDEIDSISAGDICAVIGLKDTTTGDTICDEKKPVILEQMVFAEPVISLSVEPKTKADQEKMSLALSKLAEEDPTFRTYTNEETGQTIIAGMGELHLDVLVDRMRREFNVQVNVGAPQVSYRETFTEIADAEGKYIKQSGGRGQYGHV*IKFEPNHDKGFEFVDNIVGGKVPKEYIKEVENGLIEALTSGPIAGYQTIDVKATIFDGSYHDVDSSGMAYKIAASLAFKEAAKVCKPVLLEPIMSVDVTTPDDYFGTVMGDISKRRGVIEGQEQRGNAQAIKAKVPLSEMFGYATDLRSNTQGRGQYIMQFSHYAQAPKSVTEEVMAARAKK*

>UUR10_RS01775 Ureaplasma_urealyticum_serovar_10_str_ATCC_33699_NC_011374 riboflavin biosynthesis protein RibF

MIIEITSTNIQQIRDQYFINELVIGFFDGIHLGHMNLLSDPNNQTILTFKNIPRKIKKLYDFNERIQQLEDLGFKRIFIYDIDQNNLSGEEFIDQILKPLTPKKIIVGANFTYGNNFCNASSLKQYFNVEIKIITNDVSTTKIKELIINKQVEIANKLLIKPYYRVGNVVRGDQIARNIGFNTANILCDNNLIDIAEGVYKAQVIFNNKKYDSVVYLGIPKTINTRSFSMIEAHILDFNQNIYDERIKIVFLKYLAPNLKFNNIDELITAIKNYIKLVLDKTN*

>UUR10_RS03335 Ureaplasma_urealyticum_serovar_10_str_ATCC_33699_NC_011374 transcription termination/antitermination protein NusG

MAYKIKDLDSKLLSDLKIDLNHTHQ*YIVTVVSGNEQKVIENIKDKLNGYGYGDKLSDLKIIKEKIKEVKIYEPSEAPRSMKNRANTK*ETIVVDGVTKYRCTKIKEGNKFNGYIFLKAEMTDQI*FLIRNTQMVTGLVGSSGKNVKPIPVPEDKILKLIADNDAKRALVSLDEQTNSQQNVVVVESHETEDLPNFEVDQQVKIVADTFFGEIARIAKIDQNKKVATVEFEFFGRINTLDLNFNDIQPYDEEAELEN*

>UUR10_RS00650 Ureaplasma_urealyticum_serovar_10_str_ATCC_33699_NC_011374 preprotein translocase subunit SecA

MNLISKISPQNRILNHARLIAEEVLKKEDEYTHFSDQELINKSDDIIEYLANNNPLDDRLVESLCIIREVIYRVHNKRAFKVQLIGAIIVYFGDFAEMMTGEGKTLTLVLVAYLNALYKKGVHMVTVNEYLVKVGAEFATPALNFLNMSVGQITANMNEYEKRNNYDCDITYTTNSELGFDYLRDNMVTNYNSKVQRGL*FAIVDEGDSVLIDEARTPLIISGEPQEEIGNYVKADRFVKTLYPQDFTLDPESQSVALTESGVEKAQKFFNTKNYYNFENSDIIHKVTNALRANFTFFNGREYIVKKDDEGEDVIALVDQSTGRIMEGRSYSAGLQQAIQAKEQIKIEPENLTVATITYQSLFRLYKKLAAVSGTAITEVEEFLNIYNMVVVTIPTNKPIRRIDHPDYVFDNKRTK*KYVIADVIRRHENGQPILIGTASVEDSEILHQLLERVNIPHEVLNAKNHAREAEIVARAGEYKAVTIATNMAGRGTDIKLSPESLEAGGLCVIGTERSDSRRIDNQLRGRAGRQGDIGESRFFISMEDTLFSRFATDNLAKADDKLSEDVISTKFFTRLLNNTQKKVESLNYDTRKNLIDYDHVLSNQRELIYKQRDKILVSSDNKDILYRMLDSVIDDIIYQSHNEPNEDIIDVKKLIDLATQNIFYDNYLNHDEYYGLDLDEIKTKLKNDCISFFEQKEQLMTPGIFNQILSEIMISNIDEE*TKHLDVTSKIREGVNLRAYEQKAPLNIYVEDSDKLFEKLKHDVA*KTVCSIGKINYVHQEYDKVNNEFIINDNEIIDNDNVIDFENTDHSLISEQEIEDSLVNIDELNDQNTKNENND*

>UUR10_RS00990 Ureaplasma_urealyticum_serovar_10_str_ATCC_33699_NC_011374 50S ribosomal protein L27

MNKLY*LTDLQLFASKKGVDSSKNGRDSNPKYLGAKLGDGQSTKAGQIIYRQRGNKIYPGLNVGQGKDHTLFAKTAGVVKYTKFMGDKTKVSVLPKEDNK*

>UUR10_RS02225 Ureaplasma_urealyticum_serovar_10_str_ATCC_33699_NC_011374 isoleucine--tRNA ligase

MKDYKSTLNMPSTAFEMRANLNIKEPKIQQF*IEHGIYEKLLAKNKDKKPFVLHDGPPYANGNIHIGHALNKILKDFVVSYHNMNNYYSPYIPG*DTHGLPIEVALSKKVKLSNLSVNERREQCKKYALEQVDNQIQQFLRLGMVSDFKQRYLTLDHSYEIDQLKLFANMLKKGFIYQDFKPVF*S*SSQTALAESEIEYGDRQSPAIYVKMQVVDSSELFNDKPTSFVI*TTTP*TLPANLAIAIHPELTYSLIEYKNENYVIAKSLVESFTKKVGFEDYKLIKDFKASALEKIKYISPITKKHAFIIMDEYVSANDGTGLVHNAPAFGLEDYYACKKYGIETEVIIDQFGKYNALVNDSELENMFYEDANQVILDRLICNQLLIHHELITHSVAHD*RTKKPVMYRATKQ*FVSIEKILPNILQTLKNDVKSTSFRGIERMHEMIVNRKE*CISRQRV*GVPIPMIFDENHEAIMDPDLVENIINVLNEKGVNA*FDLDVNAFLTPKYLSMKNKTFYKEKDIMDV*FDSGSSYNVLQHYNLPYPADVYLEGYDQYRG*FNSSLITGTILNNKAPYKYLVAHGMVLDGEGYKMSKSKGNVVDPLDVCKVYGADVLRL*IANSDYQNDTRISEEILKQNAEIYRRIRNTLFKYSLSILNDFEPSVDFSFDVRQEDQFVLNEFNELHLKVIKAYESFDYQTIVKLFNKFILDLSSWYFENIKDDMYCLAVDDPIRKQIQSTVY*ILKNSLIDLTPIIPHTTEEAYSFLNDANKKESIRLEDFYDQSQFQFKKGIAHVKAFFSIKDEIFNELENARKNNVLKKNNEALVTIAKNLILDDYLLNNPKLLAKWFGVAKIEFTNTTSVVNANFKKCLRC*NHFADDEMYDDELSMNCYKVINKIK*

>UUR10_RS03245 Ureaplasma_urealyticum_serovar_10_str_ATCC_33699_NC_011374 30S ribosomal protein S16

ILVKIRLTRVGTHKKPFFRIVVMDAKAKANGAYIENLGHYDPVLGKVVLKKEAILAQLQNGAQPSETVKNILSQEGI*KEFIALKDANKKRKAALAKAK*

>UUR10_RS00390 Ureaplasma_urealyticum_serovar_10_str_ATCC_33699_NC_011374 FAD-dependent oxidoreductase

MNQQIYDLVIIGAGPAGLAAAVYAKRSGLNVIIVEKQFPGGKVALTANVENYLGINSISGPELAYKMYEQVLNLDILVIYELADEITLKEKYKEVKLATQTLIAKTVIIATGTENRRLNIPGELTFENKGISYCAICDGPLYKNKVVSVIGSGNSAVEEAIYLATIAKEVHLIANKPEFKAERQMVEIVKNTSNIKIHYNKQTFEFFGEEFLQGLRFKDLVTNEITTLNVEANFTFIGLLPSRINASNLNIFNETNGFITTNKNMETNVHGIFAAGDIVDKSVRQIATAINDGVIAALYAKEYITRNN**

>UUR10_RS03380 Ureaplasma_urealyticum_serovar_10_str_ATCC_33699_NC_011374 thioredoxin

MLIKLENNQNLNQILKDNHSKPVLIDFYAD*CPPCRMLSPVLDSIEKKYGDEFTIIKVNVDHFPELSAQYQVKSIPSLFYVKNEEIKTNSLGFIDENSLVNKLRSI*

>UUR10_RS01210 Ureaplasma_urealyticum_serovar_10_str_ATCC_33699_NC_011374 translation initiation factor IF-1

MADTEKLKMLGKIVEILQGGNFRVQLENGITIMSHVSGKMRVNKINILPGDTVDVELSPYDLTRGRITYRHRDS*

>UUR10_RS03430 Ureaplasma_urealyticum_serovar_10_str_ATCC_33699_NC_011374 glutamate--tRNA ligase

MKIRTRYAPSPTGYLHIGGARTALFNYLLAKAYDGDFIIRIEDTDVERNVEGGIDSQFNFLE*MGIVADESIRNPKTFGPYIQSQKLKHYEALALDLVAQKKAYFCFCSKERLDADRELAEKLHETPKYKRHCLNLNEQTIQANLLANKEYTIRLKIDENNEYS*DDLIRGKISIPGSALTDPVILKSNKIAMYNFAVVIDDYEMQISHVIRGEEHISNTPYQLAIAQALNYDLSKIKYGHLSIIVDETGKKLSKRNLALKQFVSDYEKDGY*PHAITNFVALLG*SPKNNQEIMSLVEMVENFDVNNLSKSPAFFDINKMN*FSTQYFNNISQDEFIDFVKTHPLTKELVLKDTTFIDKALLFKSHIVNLKQLINLVDEQFNSNKQLLEEDVNHIKNNQLTNVVQVFYEQLIVSEKFDEQSIKEVIKQVQKTTNNKGANLYMPIRIATTFSSHGPELAKTIYYLGRENVLKNLQSILKVLG*

>UUR10_RS03445 Ureaplasma_urealyticum_serovar_10_str_ATCC_33699_NC_011374 membrane protein insertase YidC

MSSVDKQNLMNRMRISVSHFAGASNANSTKKERRKKILNILLKVFKVIVYTFFLGIGLYGCFQNMANH*TINSTVVGNGFELGFHVDPILGANDIRFDLIYSGTGP*YPMSDFSFDYGPFYALFVWPIAQILLHFMYATRD*PAGLNAILGLIIILLIIRVITMLISARATIQTERISEIQGKIAEINAKYKDAKDMQSRQKKQMETKELYQKHNVKPLAPFESMIITLPIFLIIYRVVTILRPLKFISIFYI*DLSATPISEIFSNFTTSG*PYIFFLLIIIPVQILSQKIPQLLAKKRNRSATTVGAKNKQQLKRVRMTQNIIAIVLAVVVAISASGIGLY*FFNAIFTILQSYIIHVIIMKRRSNSATRIESKLAKLGIS*

>UUR10_RS02740 Ureaplasma_urealyticum_serovar_10_str_ATCC_33699_NC_011374 glycine--tRNA ligase

MKNKFKTQEELVNHLKTVGFVFANSEIYNGLANA*DYGPLGVLLKNNLKNL**KEFVTKQKDVVGLDSAIILNPLV*KASGHLDNFSDPLIDCKNCKARYRADKLIESFDENIHIAENSSNEEFAKVLNDYEISCPTCKQFN*TEIRHFNLMFKTYQGVIEDAKNVVYLRPETAQGIFVNFKNVQRSMRLHLPFGIAQIGKSFRNEITPGNFIFRTREFEQMEIEFFLKEESAYDIFDKYLNQIEN*LVSACGLSLNNLRKHEHPKEELSHYSKKTIDFEYNFLHGFSELYGIAYRTNYDLSVHMNLSKKDLTYFDEQTKEKYVPHVIEPSVGVERLLYAILTEATFIEKLENDDERILMDLKYDLAPYKIAVMPLVNKLKDKAEEIYGKILDLNISATFDNSGSIGKRYRRQDAIGTIYCLTIDFDSLDDQQDPSFTIRERNSMAQKRIKLSELPLYLNQKAHEDFQRQCQK*

>UUR10_RS02850 Ureaplasma_urealyticum_serovar_10_str_ATCC_33699_NC_011374 acyl carrier protein

MVVNVKDIIIKVAKENKINLNMNNLDVELKSLGIDSLSAMSLIMKIEDKIGVQLVDEKLLKIKNLGDLIMAFEDALK*

>UUR10_RS00325 Ureaplasma_urealyticum_serovar_10_str_ATCC_33699_NC_011374 ABC transporter ATP-binding protein

MNQQSQTIKPVSKLHLIYYYFKDFKKSFIGLSIAIVL*LAATIAATFLLQETVDKYAVANGVVDTVVKMCGGLIGIYFLSFIFLLIMNEFAIRISFKIESHLSMMVINRIRYLPMKYFDVNKSGEIFTKTLSDPTSVQDGIVNFYIELNKTFFGALGFGIALLVTSPYIALIGIAIYTLVMLFNILIFKKSRQLMRKKRQDFGEMNGYIEEMIHGQNVVANFDEQAYFVKKLDNMIKNLYKN*VKAQYSSQIIFP*SIFSMRLMNAVLIVSYLLISIKGIHLPGIVSNIDPVTNMISFGGLVSISLFGNFFCDNFSQLSNAIPIFIIARTSLAKIDEIVKTPNEID*DEKLVIDDSQGIEVRFENVNFNYSKNKPTLKNINFVAKKNQKIAIIGPTGAGKTTITNLINKFYDINSGHIYFNDVDITNKSRASVREHISIVLQDPFLFSESIYENIKKGKMNATKEEIVEAAKKAQAHELILSFEKDYGTVISEKQSLSQGQKQLITIARAIVSDAKIIILDEATSSVDTQTEHKLQLAINNLLKGRTSFVVAHRLSTIINSDLILVVKDGEIIAQGNHDYLIKNSSFYQDLYYTNFAE*

>UUR10_RS00715 Ureaplasma_urealyticum_serovar_10_str_ATCC_33699_NC_011374 F0F1 ATP synthase subunit alpha

MTDNKNHSLISDIKSQIKKFSEKALTLEVGNVISLGDGIVLVDGLDNVMLNEIVRFENGVEGMALNLEEDAVGVVLLGDYSNIKEGDRVYRTKKIVEVPVGDIMLGRVVDALGKAVDNKGNIVANKFSVIEKIAPGVMDRKSVHQPLETGILSIDAMFPIGKGQRELIIGDRQTGKTTIAIDAIINQKGRNVNCVYVAIGQKNSTIANVVRDLEAHGAMEYTTVVTANASELPALQYIAPFTGVTIAEE*MHQGKDVLIVYDDLSKHAIAYRTLSLLLRRPPGREAYPGDVFYLHSRLLERACKLKDELGAGSITALPIIETQAGDISAYIPTNVISITDGQIFMMTSLFNAGQRPAIDAGQSVSRVGSAAQIKSVKQTGASLKLELANYRELEAFSQFGSDLDDETKRILKLGKAVMAVIKQEPNKPYNQTDEAIILFTVKEKLIPQVPVERIQDFKEYLLNYFKGTKLRADLEEKKAFDKENTPAFKCAIQKAINNFLNNSQDFKPCDELEQTAYDKFFNENEPIVVVNENEFFDEQINSLPTFESIHPVQIEEKVQELAEPREVFEVNKIEKEHLFEEVEPEKIICEHHQFEITEDQEEVDGQEVLEDENHEYAIYEVVEQNDTIENCKEANDEAEIQVPVAEVVQDEEILNERENRN*VFSDSAVSEVEKQTIMISISPNESEQLFDNGRSVVFFKVAPKYPVEKVLVYVTSPIQKVIGEFDLLKIDVNSVNSS*NKYRSSSVISSRKEYLEYFSSHKEAHALLASKVYKYRRPKDLASFNMKKGPSGFTYLK*

>UUR10_RS01215 Ureaplasma_urealyticum_serovar_10_str_ATCC_33699_NC_011374 50S ribosomal protein L36

MKVRASVKAICKDCKIVKRSGVVRVICANPKHKQRQG*

>UUR10_RS02080 Ureaplasma_urealyticum_serovar_10_str_ATCC_33699_NC_011374 NAD(P)-binding domain-containing protein

MSKILIIGSGAFGSALTQVLVSNHHAVDVYGINQNELNDLQQNQKNTTYFQDQKLSQPINNTYLDIHLALKNHYDFIVIVIPSFAIKNFVDSIKTLDLSQAIVVNAAKGLNLETKSS*CDYIQQNLKIKALIGLVGPSFAIDVFLKKPTVVNLVGTDLDALIKTKQAFEND*FKCVLSKQFEVANYISCFKNALAIGCGIIYGLEKSHNSLVAFLTKGINEMQLILETIYQKKVNPLEYFFIGDTILTCTDQKSRNFSFGLLVAQQGVQTALENKQKTVEGLNNIKVIYEIIKTKQIDAPLFESLYEVINENLTPKSLFNKSFC*

>UUR10_RS00005 Ureaplasma_urealyticum_serovar_10_str_ATCC_33699_NC_011374 ATP-binding protein

MSNNYQNLYDSAIKKIPYDLISDQAYEILEKAKVHKVYDGVLYIIVASAFEKTIINGNFINIISKYLSEEFKKENIVNFQFIVENEKVLINSNFLVKETVIKNRFNFSDEIMRYNFNNLVISDFNRKAVKAIESLLSTNYENSSMCNPLFLFGKVGIGKTHIVAAAGNQFANSNPNLKIYYYEGQDFFRKFCSASAKGTSHVEEFKKEIASANLLIFEDIQNIQSRDSAAELFFNIFNDIKLNGGKIILTSDRTPNELNGFHDRIISRLASGLQCKISQPDKNEAIKIINN*FEFKKKYQITDEAKEYIAEGFHTDIRQMIGNLKQICF*ADNDLNENLVITKDFIIECSVENDIPSNIIVKQQLKPEQVIEIIAKELNLKVDLIKSTTRKNSIVWARDIVCYVLKNKLNLTLTEIGKLLSGREHTTISHSVNKVEKILADKNSQEALQINLIIDKF*

>UUR10_RS02730 Ureaplasma_urealyticum_serovar_10_str_ATCC_33699_NC_011374 GTPase Era

MVKKYGIVAIVGKPNVGKSTLINAIMRKKVSIISNKPQTTRNAIKEIYEDDDSAIIFTDTPGFHEPSNKLDLFLNHEIEVSYKEANVILFVSSMDKELSEDDFEIINLIKESNKENVILVISKAEVAKNQDKIDERVHQLNKYIQFKDVIQISALHVINIDKLINTIKQYLHKDVVTDYFRQKVEKEDKFIIAETIREQCLLNLNHEVPHGVGVEIDESKYNQEANH*IIKASIIIEKNSHKPIVIGQNGAMIKKISMAARKQLHEIYDCHISLTIFVKVENN*RENNNVVKSLGYKIKK*

>UUR10_RS02100 Ureaplasma_urealyticum_serovar_10_str_ATCC_33699_NC_011374 16S rRNA (cytosine(1402)-N(4))-methyltransferase RsmH

MEFNQHTTVLLNETIELLHVKPDGIYVDCTFGRGGHSQLILKKLSKKGKLICIDQDQQAIDFANNLFKDNPNVIVIKTNFKNLKSVLYDHQIFHVDGFVFDLGLSSPQLDDPERGFSYHKDALLDMRMDQEQKLNAHYIVNHYSFAKLVNIFTKYGEIKYAKTIANGIVKERSTKAINTTLELVEIIKNYSPKKILFEKKHPARLFFQAIRIEVNDELNILKKAFNDAISMLNPLGVVAIISFHSLEDKIVKKVFNNYAKNKLPKEIPLNNYVNQYSLLNQKIMPSTQELNDNNRSRSSILRGLVKNY*

>UUR10_RS01015 Ureaplasma_urealyticum_serovar_10_str_ATCC_33699_NC_011374 serine/threonine-protein phosphatase

MNFGFISDIGSQRKHNDDCALVIQNEHQQTLLIVCDGLGGYKGGAAASHITLETIKDNFLATNFNEYDEQQIRK*YIKVIKLAQIEIDRAVLLDKDVYNMGTTVVASIIINDFVYTLNIGDSRAYLLSNNQSSQISRDHNLLQVLHERKVGPEVYEKHEKNLFSLTQFVGRTSNVVLSYDLFVTKLHHNEIIVLTSDGFHNYFELNDLYDKLIVTNQQTNNQILQQLINQAIDNGSNDNLSLAFLIF*

>UUR10_RS02725 Ureaplasma_urealyticum_serovar_10_str_ATCC_33699_NC_011374 rRNA maturation RNase YbeY

MRFLITNEVKSVFNDEIYLQRFEQIANLISIKLNIDKERFFECHFVDEKTIQEINRDYRNKDYITDVISFAFDDGEIITPLLGEMYICYQKVVNQAKEFGHSFERELCFLFTHGLLHLLGYDHIEVEEEKIMFGLQDEILNELNITRNVNGNKNG*

>UUR10_RS01085 Ureaplasma_urealyticum_serovar_10_str_ATCC_33699_NC_011374 50S ribosomal protein L35

MAKIRQKTKRAAAKRFSITKNGKLKRKHAYRSHLALGRSTKAKRHLRKDAIMSTSDTKRYTQCL*

>UUR10_RS03145 Ureaplasma_urealyticum_serovar_10_str_ATCC_33699_NC_011374 30S ribosomal protein S18

MAKVINNRNRKPRKKVCILSAKGIEHVDYKDVELLQRFINNNNKIASRRVTGASARMQRRIANAIKRARFVGLLPYVKE*

>UUR10_RS00705 Ureaplasma_urealyticum_serovar_10_str_ATCC_33699_NC_011374 F0F1 ATP synthase subunit gamma

MSLDAIKRKISSVQTTAKITNAMKLVATAKLKRQRDRLAAIKEYCHDYYDVIGLLLSVVNDIEFLKIPNAKNRTLYITINSTMGLAGSYNYNVNKLVSKIINEDDITFTIGKKGHDFMRLSNRLHQVNTYLNLNDNDLTFDMSLQIAREALELYSNGEVNKICIIYTKFINAITFEVNNIDVLPFDKTVLTKDNLAETIELAKDNIIFQPNKVELVKKILPTYIATVLYGSLIESKISENASRRNAMDAATKNAKALAEDYKLIYNTLRQGKITREITEIVAGSDD*

>UUR10_RS00805 Ureaplasma_urealyticum_serovar_10_str_ATCC_33699_NC_011374 tryptophan--tRNA ligase

MKRLISGIQPTNNLTLGNYLGAIKNFVDLQNDYEVFLFVADLHSLTPNIFDNTNFFATKRQIIATYLAAGIDPKKTCLFYQSDILAIPLLSHILLCSTSIGELTRMTQFKDKSAKATKMANNTEMIPSGLLTYPALMAADILAFNADVVPVGQDQKQHLELTRTLADRFNKRYGQTFKLPQVYIPKIGAKIMDLLDPSVKMSKSSKNPKGVIFLNDSREQIIKKIKGALTDNLNQVKYDVEQQPSVSNLITIYACLTNLTFAEIETKYNQQNYGVFKNDLANIVADFLENLQQKISY*LNSPELDIMIDNSCERANDVANQNVQLVLKQMQLK*

>UUR10_RS01600 Ureaplasma_urealyticum_serovar_10_str_ATCC_33699_NC_011374 30S ribosome-binding factor RbfA

MANEVRVARLESLIKDVINNALANEINDKIAKLARVTAVRLSNDLSVAKIFLDAHKRESMPKVLENVNKVSGLLRSKLAAE*TSYKVPELRFVIDETIDYANHIDELFKKIKQQEN*

>UUR10_RS03155 Ureaplasma_urealyticum_serovar_10_str_ATCC_33699_NC_011374 30S ribosomal protein S6

MAKYEIMLVVRGDLDQEQANKVANELKATLKNTEVKENNYEGVQQLAYEINKLKTAYRYVYNFETTDVSLINEFRRLAIINKNVLRHIIINLEKDYGYKATVNAKKVQRNEKRAEVYVRQKEEAERRAAERQAAYEAMKAEREAAGLPVKEFVKGANSKR*

>UUR10_RS01550 Ureaplasma_urealyticum_serovar_10_str_ATCC_33699_NC_011374 tRNA (adenosine(37)-N6)-threonylcarbamoyltransferase complex dimerization subunit type 1 TsaB

MNSLYQLFIDVTSKKCVLAIYKNFKILANIIVETNNNLTDIIVEHIIALLKAVHLKYQDLDAIYLDIGPGSFTGVRVGAIVAKTICTTHNQIKLFINDSLNIIANNKNNVFVHLDAKGNKSYTISIINNIQSDYRIITNEQLQIELKNTSLTIIDANQVDYHNLIYNLKFDNFKLTNILDFDLNYVKKPLS*

>UUR10_RS00055 Ureaplasma_urealyticum_serovar_10_str_ATCC_33699_NC_011374 50S ribosomal protein L7/L12

MSKLTIEQFIAAIKEMSMLELNDLVKAIETEFGVSAAAPVAVAAAPAAAEAPTEVTIKLVEAGANKVGVIKLIREITGLGLMEAKTAAETAGSVIKEDVKTEEANEIKKKFDELGAKVQLV*

>UUR10_RS00695 Ureaplasma_urealyticum_serovar_10_str_ATCC_33699_NC_011374 ATP synthase F1 subunit epsilon

MANLTKLKIVTPYAQNLEKDVYSVELKTSEGRIAVLPDHNPLMSIIENHVAYIRELPNAPRKPLLLLDGIVYVEEHQVRVFSDYFKFLDEIKIDEINSLLNKLKNDLANEEDDKKKLQLKSKIKLNESILIAYKDR*

>UUR10_RS01180 Ureaplasma_urealyticum_serovar_10_str_ATCC_33699_NC_011374 50S ribosomal protein L18

MKRINFSRAKQRALRAKRLHVKIRNLQLAANKPVLVITKTNAHI*AQLICYNKNITLASSSSVQLDLQNGNKDNARLVGADIAKKALAQGFKQVIFNKNGAKYHGRIKALADAAREAGLEF*

>UUR10_RS01220 Ureaplasma_urealyticum_serovar_10_str_ATCC_33699_NC_011374 30S ribosomal protein S13

MARILGVDIPNDKRVVISLTYIFGIGKSTSQKILKLANIDENIRVNDLADEQIAEIRRVALNFVKANGEKLQLEGDLRRTVAMDIKRLMEIGSYRGIRHRRGLPVRGQRTKTNARTRKGPRKTVANKKIETR*

>UUR10_RS01000 Ureaplasma_urealyticum_serovar_10_str_ATCC_33699_NC_011374 50S ribosomal protein L21

MFAIFQTGGKQYKVQQGEKIYVEKLDLEVGSKISFDQVIMVEGSVGTPFVKNAVVNATVLKQGKQKKINIIKFKSKKHHLKRQGHRQPYTQLVIDSISVK*

>UUR10_RS01160 Ureaplasma_urealyticum_serovar_10_str_ATCC_33699_NC_011374 50S ribosomal protein L5

MAFLKDLYKNKVAKDLQKEFAYSSVMQIPKIEKVVINAGIGNAVADKKHLEAAISELTLITGQRPVETKAKKSIATFKLRAGQSIGAKVTLRGDRM*AFIETLFNIALPRVRDFKGISNNSFDDQGNYTLGIKEQIIFPQVVYDDVKSVRGFDVTFVTTAKTAQEAKALLVGLGAPFQKVRGDK*

>UUR10_RS01710 Ureaplasma_urealyticum_serovar_10_str_ATCC_33699_NC_011374 (d)CMP kinase

MKKYINVAIDGPSGSGKSTAAKGLANKLGFLYINTGLMYRAYAYFLNENNLDINTNETACIEAIKNARFIFNGDDVKIDDQDVSDILRSNDVAMLASVVAANAKIRNLATNEQRKIASENNVVMDGRDIGSIVLVDADLKFYLNTSIQTRAKRRLAQNKDIEKLDYESIYNDIKERDYRDMTRDIAPLKKAIDAIEIFNDNMNLDQCVAHLYEIYLNKIKKS*

>UUR10_RS00815 Ureaplasma_urealyticum_serovar_10_str_ATCC_33699_NC_011374 NAD(+)/NADH kinase

MKDVKPVYFYDIYCFNPNKCTEDKGVLLLETKLKEYQKVTFLRSEQKPQIVFLLGGDGSFINFVNQQ*KQN*KIVGINYGQLGFYSSYDGINTINIDEIVDESMYANAFLIEVNINNENKFYCLNELSIFSNELASCDISINNTFYEKFRGSGLLFATPSGSTGKNKVAHGPIIFNNQPCFSMLEIFPVNHLKYSSLNAPVVFGKDYQISLTNIKFKRTLNLVVDGNNINFNNKIDFIEVKLIQASLQIHGLNNYKKYIERLRRSFIKEE*

>UUR10_RS01100 Ureaplasma_urealyticum_serovar_10_str_ATCC_33699_NC_011374 50S ribosomal protein L3

MKSLLGTKVGMTQVFTETGKAVAATVIYVEPNKVLAVKTNEKDGYNAIQIGYETVKEKALNKPLLGQFKKANSDPKRHIKEFRDVVAEVGAELTVSEFEPGQLVNAQAYTKGHGFTGSIKRHNFSMGPMGHGAGYPHRYVGSIAKGRGGSQAQRVFKGTKLPGHYGHELVTTKNLLVLDVKANENLILIKGAIPGPKGSIVLLKSAKKVGHIVSDPQVVNYLANKASSSEANK*

>UUR10_RS03075 Ureaplasma_urealyticum_serovar_10_str_ATCC_33699_NC_011374 energy-coupling factor transporter transmembrane protein EcfT

MSANAYVFRRSPIHRLNPAIKFISFILLIAMIFLPLGFFAQMIIGVFILIIFFVAKLPKKTL*NVFKSVIMLFVILLLIN*MTYKDPIAIYNITDQAKVILGDKD*INGPINKNLSFSLIYNDISSTHVQNLVSNIWGGEIKNYISPEIIKKLIDKPDYNVAKFLSENNITVKKLASTFNALNQDVRLNNYYPIYGDAVLRSGKVEVPLSHLSYYMSTNL*KIEGVKYQGLILSGVGDQLGKAETALFYTRSPFALSPVAIQLAIYISIKIFLMITLSSILTATTSSIELTNGLEDLLSPFKILRLPVAEASMMISIALRFIPSLLDESKRILNAQASRGVDFNNGGMLQKLKSLISLVVPLFSIAFKKAEDLANAMEARSYNPRYARTRYRAFPLNLTDYVLFGILCILVGFLISLAVIKFYFTPFGAFEASALFAK*

>UUR10_RS01185 Ureaplasma_urealyticum_serovar_10_str_ATCC_33699_NC_011374 30S ribosomal protein S5

MENNVKKETIVDSEKVEKQQPVTAPVVNKKENTQPKAKTFKRETTTSNFEERVVKIKRISKTTKGGRMMRFSALVVIGDKNGTVGFGMGKSIEVPDAIKKAIKNANNNLIKVKQTKKGSIYHDVNGRHGAAKVMLLPAPEGTGIIAGGPVRAVVELAGFTDIYTKSRGANAPMNVIRATINGLLQQLTPQEIARLRDKSLKEL*

>UUR10_RS01115 Ureaplasma_urealyticum_serovar_10_str_ATCC_33699_NC_011374 50S ribosomal protein L2

MAVKRIKNHSSGKRQTVVVDYKSILTTSKPEKSLLVTLPKKAGRNNQGKITIRHHGGGHKRKYRIIDFKRNKDNIYGTIKSIEYDPNRTSFISLVVYADGEKRYIIAPKGIKVGDKIISGNENIDILLGNSLPLEFIPEDTLVHNIELSPNAGGQITRSAGASAQILGFDETKKYILVKLNSGEVRKFRKECRATIGTVSNDEHILENLGKAGKSRHLGVRPTVRGSAMNPNDHPHGGGEGRSPVGMDAPRTP*GKRHMGVKTRNNKKSSTSMIVRRRK*

>UUR10_RS02180 Ureaplasma_urealyticum_serovar_10_str_ATCC_33699_NC_011374 tRNA 2-thiouridine(34) synthase MnmA

MEVNTKKRVVIGLSGGVDSSVSALLLKQQGYEVIGLFMAN*DTVANFENNRESDKKHQGCESELDYQDAQAVAQKIGIPLYRVEFIKEY*NNVFEYFLSEYQKNRTPNPDILCNQFIKFDSFLNYAKNELKADYIAMGHYAKVKHTNNLSYLLKATDVNKDQTYFLCNLKQTQLQNALFPIGDLTKQQVRTIAKEYGLVTANKKDSTGICFIGERNFKYFLENYIPNQPGEIVNIVNNQIVGHHMGTMYYTIGQRKGLNLGGMNERMFVCEKDINKKIIYVSPLSLEDQYLISNQALVENMNFIEPYNPQIPISVRFRHRQNLVVVNSFLCIENTNNVLINYEPAKAITPGQYAVFYQNDHCIGGGVIAQTNANHKKINF*

>UUR10_RS00025 Ureaplasma_urealyticum_serovar_10_str_ATCC_33699_NC_011374 L-threonylcarbamoyladenylate synthase

MKIYRITNLNAIYDALVANKCVLIPTDTIIGLLAKNQDVIYEIKRRDRNKKIVRFVADYKLLGDLTVEQEQFLDLF*PGSVTVIKNGVSYRMPNSPYILKLIQKLGPLYCSSANISGEEPVKNHNEAIFKFGANSKLIYVEAQQQIGVPSTIVDIDK*EYVRRGANIEMVDMFIKELKYNNTKEKE*

>UUR10_RS01125 Ureaplasma_urealyticum_serovar_10_str_ATCC_33699_NC_011374 50S ribosomal protein L22

MTNKVIQRNIHISHRKASLVIDLVRNKPVHEAIRILSNTPKKFAPIVLKLLNSAISNVQHNSKDMDPSKLYIYKIVANQGPTMKRTLPRAKGSADQLFKRTTHLEIVLSDDVNEREKELAAIKAKKSKKPLVVEPVAKVETKKVAKPSKVETKPVEKDENVDPELLKREQQVLKVVEKTASQKEEETTETIMISTSPKNAQVLFDDLEKNVIFYKTTPVNKVLRVLVYVTSPTKKVVGEFDLESVEIGAISSI*RKYNKQSVISKKEYDAYYEGKDKAHALVSKKAYKYRNPKDLSEYNMTKGPSGFQYLK*

>UUR10_RS03095 Ureaplasma_urealyticum_serovar_10_str_ATCC_33699_NC_011374 50S ribosomal protein L11

VAPKKKEVTRIAKLNLIGGQAKPGPALASVGINMAEFTKSFNDKTKDQNGKVIPVIITAYKDKSFDYVVKTTPVTYLLKDAAKIKSGAKDPKKQVVATISKEQALEIARYKLVDMTAYDEEAALRMIAGSAKQMGIAIEGVSAYKEKKGN*

>UUR10_RS00090 Ureaplasma_urealyticum_serovar_10_str_ATCC_33699_NC_011374 tRNA uridine-5-carboxymethylaminomethyl(34) synthesis GTPase MnmE

MSTIVALATAPMNCAIHIIRISGPQAFEMINKISTTKIKKETFKIWYTTLKDNDQVLDEVLVNTFVGPKTFTGEDLVEINCHGGVIVANLIIKILIKYGCQPAQRGEFSRRALLNKKMDLSKIEAINNLVNAKNELSVKGVIGALLGRVSQSISDFKHELFMIIGQIEVNIDYPEYDDVEQVDAINLKQRLLVLNEKIKKIIDQSKKFLPINKGIKVLIIGKPNVGKSTLLNALCNEQKAIVTDIPGTTRDVIESSINIDNITLNILDTAGIHSTNDFVENLGINKAKELINKVDLVLYLVPANNQQDLELYDLIKDQKHLLVYTKKDLIDQYSDDQIYINAKDNDIQALIDKIKELFYVQEFDNANIDVLQSQRQIGILENVNYLIDNAITNLEKGDTVDLVVADLEFCNLRLNELLGIGSEYDFLDDLFKNFCVGK*

>UUR10_RS01135 Ureaplasma_urealyticum_serovar_10_str_ATCC_33699_NC_011374 50S ribosomal protein L16

MLQPKRTKFRKPHKVSYEGKAKGNKQVDFGEFGLMALEGA*IDARQIESARIAISKRLLKTGKM*IRIFPHMSLTKKPLEVRMGSGKGSPEK*VAVVKAGTVMFEIANVSEELMREALRAAGNKLPIKVKIVKKGEAN*

>UUR10_RS02210 Ureaplasma_urealyticum_serovar_10_str_ATCC_33699_NC_011374 molecular chaperone DnaJ

MAKRDYYEVLGVSKSASPEEIKTAFRKLAKEHHPDRNKSADDTVFKEINEAYEVLSDPKKRAQYDQFGHDGPQGFAGAGGFSGFSDGFGGVDFDINDIFGSFFKNGASSRSSSSQYETYDIHLRLHLEFIEAIKGVSKNISYDRKITCNKCQGTGAKDPKDVKTCTKCHGRGTTIENVHSLFGTIQQEVECHECEGTGKVANSKCEQCYGKKVINERVNLTVEIPAGTQDNEKLVVSKKGNIINNQEFDLYLHISVKPSKYFAFDGLDIYSETYVDPIKAIVGGVIEVVTTSGIKTIEIPPNTPEGKKFRISGAGIVNKKPNIFSKKNGDFYTTIRYAKPLELTKEEIAYLKNISARTNQSVEYYKNKLLKEVNK*

>UUR10_RS03105 Ureaplasma_urealyticum_serovar_10_str_ATCC_33699_NC_011374 Asp-tRNA(Asn)/Glu-tRNA(Gln) amidotransferase subunit GatB

MQNFEVIIGIEVHTALNTKTKMFSNTPTSHKSMANTLINEIDLALPGTLPSVNQEVVHKGLFLANALHMHTNHQFIAFDRKHYYYLDLPKGYQITQNYFPIGQNGYIQITDENNNPKKIRIKQIHLEEDTAKQTSVNNQVYLDYNRAG*PLIEIVSEADLRSAQETVLFLEELRKILLFNDISDAKMEDGSLRVDVNISIRPRGAKSFGTKVEIKNINSISNVAKAINYEYNRQLNLILLNQSVEQQTRRFDDSTNTTVFMRSKNDAINYRYIRELNIAPIYLSDEYVSQLLSTKPYSINDLRQELLQKGLVSSAIEQLLGDGPLFKAFKYVNKIVNNPSSVYK*LCLEFIGLINKNTQIIEDISMELLQKIGAMIVLFDQTLINGKQTKTILEKIYLTNKDPQTLIKELGFEQITDENEITNL*NQILANNQEMLLQYEERPDRVEKFFMGEMMKLTKAQANPTISFNILKKILQK*

>UUR10_RS00555 Ureaplasma_urealyticum_serovar_10_str_ATCC_33699_NC_011374 serine--tRNA ligase

MFDINLIRKDIVVTKEKMLNKKVSSDLFDQIFGLDVLVRNLMQQEQNLNAKKNQLSKEIGILAKNKDPKLQQTLDLVNSIKSELQDISLTLSNKQDELNKLLLVIPNMPDDSVPIGNDENDNVEIKKVFEPRKFDFSPLAH*DLAAKNKLIDFDKSTKITGSRFIIYTNFGARLYRALQQFCLDMNVKAGFNEI*APVIVNQESLIGSGNLPKFVDDLFKLENSNYYLSPTAEVQLTNLHRNEILKASDLPLYYTALTPCFRSEAGSAGRDVRGVIRQHQFHKVELVKLCKPEDSFKELESMTRQAESILEALELPYRRIALCTGDLGFSSAKTYDLEVWLPSYNAYKEISSCSNCTNFQARRAKIRYKETVDAPTELVHTLNGSSLAIDRL*AAVVENYQQEDGSITIPKALEKYIY*

>UUR10_RS01745 Ureaplasma_urealyticum_serovar_10_str_ATCC_33699_NC_011374 RNA polymerase sigma factor

MSTKQNEPLFENLEDLKQKVKLSFTEEFSFSYALSEREAGIFESRNLKGADASNPEEILLNVVLDVSKRKRSRNEIKFNKLQNYFIHMNLRDEHFSEIVDVLENIGIRVPDYELVMQSKSKSTAKKKDEYGIDDTLEISTSKIGFSSTTTEKVDDGIKAYLGVLGESKMLRSDEETEYAKMVISNDPALIKIGKNQLYTSNMRLVTSIAKKYLNRGLDLEDLIQEGSSGLLKAIDKFDHEKGHKFSTYAT**IRQSITRAIADQARQIRIPVHMVETINKLTKAERSLIQELGRDPTAEEIAQAMNKASQAKNQKEQLITAQKVVEIKKLNVDPVSLDKQIGHDEESQFSDFISDDEIISPEKYTEKKALNDQINEMFEKVLNDNEQRVIKMRYGLLPFERPYTLEEVGEHLGVTRERARQIESKAIRKLKHPSKTAKLRSFIGESEN*

>UUR10_RS00400 Ureaplasma_urealyticum_serovar_10_str_ATCC_33699_NC_011374 HPr kinase/phosphorylase

MEIRGKLFVSQVVRKFNLNVVANSDYIDREISTTGITRVGFELAGEILFKEI*NIVYFGSKESNYFSKFSETIISKKLGKILDLNPPLIIFGKNFKHAGILLKLAERYKIPIVEVKYSFYELNFTINTYISQKLSHQSLVHGTLLSIYGIGVILMGESGVGKSELAIELVKKGHIFVGDDAILVNRIGGNLYGRAEDSTKDFIEIRGLGIMNFSRSFGIERMIESTKIEIVIELIKAAKHEKIKFERFGREIQHKEFLETKIAYYYIPVIEGRSISDIIETAITDYKLKTSGYNSAEEFILQIDKKGN*

>UUR10_RS00980 Ureaplasma_urealyticum_serovar_10_str_ATCC_33699_NC_011374 50S ribosomal protein L28

MARRDQLTGKGPLSGNTRSHAMNHSKRR*NVNLQKATIKTENGSQRVLVSAKTLKTLKKHNLLA*

>UUR10_RS00670 Ureaplasma_urealyticum_serovar_10_str_ATCC_33699_NC_011374 cysteine--tRNA ligase

MKLYDSYSNQLVEINDELISIYNCGPTVYNHIHIGNARPLITMDVLYRFLKKHNIKTKYVLNITDIDDKIINYALANNLKELEVSEYYFNEYLKIKKALNTLEMINPKVSTHMDKIIDYIQKLIDKQAGYFIGDDVYFDTKKALNYGQLSKRDLENDIVGMRIESAANKHNPNDFIL*KKTNKGIM*NTP*GIGRPGWHSECSCLINTYIGEQVSIHGGGIDLKFPHHENENAQNQVLYNKNLAKV*MHFGLVNINNEKMSKSLNNFILVKDLLAEYDYQVVRWFFYQADYKQPIKFSHEIMKQNEKEILKIKNAIYNAKNYLYFNHQLKSLTQIDHFGFFDERINDDLDFVGIVDLIHISVKKINILIKKNKDMNELKLNLTQLLYMLDILGINFVDLHNDENLALLNT*KNYVDKKDYVKADELRKQLINIGIL*

>UUR10_RS00015 Ureaplasma_urealyticum_serovar_10_str_ATCC_33699_NC_011374 peptide chain release factor 1

MEYNKKLYEAIERVAVKNKTLKQELETITSDFKKIKEINIQLKKTTKIAEAFAKYKQKIDAGIVAEELLNTEKDLELVELAQMDLDDAKASIPVIENELKIMLLPTDPNDDKNVIVEMRPAAGGDESSIFVGNLFDTYRAYTESNN*KMKIIEMTPNAVGFSFISFMISGEEVYSRMKFESGVHRVQRVPATESKGRVHTSTITVAVLPEQDEVDVVINPSDLRIDTYRASGAGGQHVNRTESAVRITHIPTGVVAACQEGKSQIENRETAMKMLRAKL*EAAQEQQNAEFANLRKNQVGTGDRSEKIRTYNYPQNRVTDHRISLTLNKLDQIMMGELDEIIDALITDEQTNLMANLGI*

>UUR10_RS02265 Ureaplasma_urealyticum_serovar_10_str_ATCC_33699_NC_011374 bifunctional oligoribonuclease/PAP phosphatase NrnA

MQKDLLNKLIEQTYGFSKISIFVHTNPDCDALGSAFALARILKLNTFGTRVKIVGINTLNPNDFKNFFTFDKNEVEDEFIEGSLAFIVDTANQERVLSQKHTLAKKTILVDHHVKTVSYTDLTYINDQSIATCEMLAYSLMHTNLNFDVKTLNYLLLGLTTDSNRLMYDKVSDITYEIMA*FFKNNVKHYQIYQQLYERNLDDILFDNELIKTIKTHKQIAYLNIDKS*NQKYNFTR*GDKVYLLSNIKNYPI*FVVYFDETTNTYKVSLRSNKYKVRLVANQFNGGGHDLAAGCSLANIDQLNDLLKALELLIKNQEVVD*

>UUR10_RS02390 Ureaplasma_urealyticum_serovar_10_str_ATCC_33699_NC_011374 proline--tRNA ligase

MAKKLEKIITRNENFAD*YTSIVNNAKLIQYTDIKGMMVFQPNA*AI*EAIKNQIDLEFKKHGVRNLAMPTLIPLSEFQKEKDHIEGFAPELFMVNQIGDKKLDNPYAIRPTSEILFCNYFKNIVNSYNDLPIKNNQ*CSVMRAEKTTRPFLRNAEFH*QELHAIFASEHEADEFAKTILDVYTDFVQNYLCIPVIKGLKTP*ERFAGAQKTYTIEAMMQDGQALQSATSHYLGQFFAKAYDIKFQGQDNQMHYVHQMSAGLSTRIIGALIMVHADDQGLILPPDIAFNQIAILSIFANKNPQLLTISEQIRNELSDYRLFEDHSDKGVGYKLAQQEIEGTPICILVGVKELANQQVVLVRRDTHEKINVNLIDLKSTIKKLLLDIKTNIYQKAKKQLDESIVFVNSIEELKQVIAQNKMAKAFFDGSKEDDEQIKLLTNASTRCIFDETQSGQCFYTNKKTNKLTLFARAY*

>UUR10_RS01350 Ureaplasma_urealyticum_serovar_10_str_ATCC_33699_NC_011374 arginine--tRNA ligase

MMITQKISEQLSKALEKMGIFETKVLVDKTKNIKFGDFYTNVAMTLSKRVNQSPLVVAKEIINNLDQDLFFKVNLQPPGFLNFTLKAKDHEDLLTQIYDQKDLFGQFAKKNITYNVEYVSANPTGYLHIAHAANAIYGDILANLLKIYGYDVKTEY*INDAGNQIDKLAMSVLVRYLQLQNINIELPTDAYHGQEIYLVAQALYEIYKDQFINVRLNEKEEIDDVIVNEQIKKFAVSYLLDEIKKDLASINTYIDTYTSEN*IRSSGRILEVLSKIKQHTYTLDGAL*LRTTAFGDDKDRVLIKSDGSYTYFTPDIAYHDYKFSKDNTTKLIDV*GTDHLGYIARLKAAMSALGYDPNNLEIVCAQVMKLVKNNEEFKLSKRSGQSLTIKDLVEIIGKDALR*FLGSSSMNSHVVIDVDIALSKNNNNPLYYVQYAHARANQVLNKQVYEFDFKTDLLIETRERELLNQLHFYKQTIANAANNREPHRISNYLYDLAQIFHNYYANIKINDENNKALSAQRYTLV*CVKQVLANGLAIMKITPYDQMY*

>UUR10_RS01540 Ureaplasma_urealyticum_serovar_10_str_ATCC_33699_NC_011374 30S ribosomal protein S20

MANIVSNEKTYRHTQKVRKENHAKMSKLRTIVKKTRSSNEQAQLNEAYKVIDTTASKGVIHKNKANRLKSRTAKAFKANLQVVA*

>UUR10_RS03235 Ureaplasma_urealyticum_serovar_10_str_ATCC_33699_NC_011374 50S ribosomal protein L19

MALFKINKGEIMNFVNSTQLKTDIPSFDSGDTIIVHNRIVEGKKTRIQKFEGVVLRRRGSGSSETVIVRKESNGVGVEQSFNIHSPLVEKIEVIKYGKVRRAYISYMRNRSGKSARIKELNKQ*

>UUR10_RS01860 Ureaplasma_urealyticum_serovar_10_str_ATCC_33699_NC_011374 Holliday junction resolvase RuvX

MRKLALDLGTKSCGFAISDLLGIIASGLDNFIYEENDFIAVLAKIDEIMINYHHEIDTIVLGYPTNVYDGSKNKRTYLIESFYTLLKQHFLNHEKIKIVYEDERFSTKIATQRLKNSCVKAAKIKKVKDKMSAVVILESYLSKNHFN*

>UUR10_RS02555 Ureaplasma_urealyticum_serovar_10_str_ATCC_33699_NC_011374 nicotinate-nucleotide adenylyltransferase

MKIILFCGAFDMVHNAHIAMAKYAIDLIKADKLIFLPSNFKFFKPINKDDNLEYEKTKLTHGHHRLAMLKIATKNLVNTEVSDYELNQVNKSYTINTIDHFKKLYGAEHEYYFIIGSDNLERFKQ*KD*ERILKEVKIICFKRSGVCLKKTCFQNQCNCENFNFFEHQIILVNDFNYNISSTEIKKQHNLASGIDPAVLDYINEHGLYAL*LLEKHLISYDNFNNLEKKIARINHCRRVAQMCVDLMNVYDKKLIDQAYCAGIYHDILKCLDEQESIAYFNEHKSELNIGDDFIS*RILHSYLGAHLLQTQYGFKNQLILNAIRRHTRPFDFIKDYSELTTLDKILYCADKLEPNRREEIDQINIDYYRKLVFEDLDKAFIEVYKYQQRQRK*

>UUR10_RS00395 Ureaplasma_urealyticum_serovar_10_str_ATCC_33699_NC_011374 prolipoprotein diacylglyceryl transferase

MQLEIINPESTLINDVVAHRIAFSIGSNFNIY*YGIIFVCGFLLAILTYSLRLKFHYKVPYDPGFYYIFLAIPMTIIGARL*SLAIGDAKDFFDFRNGGLAIQGGVIAGVLSAAIYFPLILRMPKYHVRDLDADGNVIIRQPSM*IYADAIIPTILIGQALGRWGNFINGEIFGAESTVNDLQ*LKKAMPAVFEGMKHYFIEGDKTLFTIYQPLFLYESFFNVIVFVFIYFGLSYIKQLKIGFVSMSYFFFYGVIRFSTESARAPQFSFAGTYVINSLLLIFGVLGALYVQFIAPILRKRFLLDAIIELFYKKKQQAHKFGQLRNPEEFLYYCHK*

>UUR10_RS00840 Ureaplasma_urealyticum_serovar_10_str_ATCC_33699_NC_011374 2,3-bisphosphoglycerate-independent phosphoglycerate mutase

MSLNKKLALIIIDGLGIGKKDDTNAVYLANPKTLNYLIKNYPTLEISAAQQPIGLLENQAGNSEIGHLTIGAGRIILNDNANINSYTKRLDYESLVLNDINNEIVHVVGMYSNGLVHSNYEHIH*IIKELVKNNNQVVLHLISDGRDDYPYGFAQFIEQINALKTQYNVIIKSLSGRYFAMDRDQR*ERTQKAFNTMFIKQDKICEQSLLEVAQSIANHYESDEFVEPIVFNNDEKYNLKPYQKVILTNYRSDRMRQLAHLLKPNRKFNYHNPFLIKDIHLITLVPFPDVDAITLFEKQNLNNTLGDVLNDHHIKQARVAETEKYGHISFFFDGGINKHYASKTQYLIPSQKVATYDLCPQMSASLITKTIIDHYFDHDVFIVNYANPDMVGHSGNMKQTIQAILSVDSEIQKLYDFFKKNNGVLMITGDHGNAETMIDANGQIITSHSINDV*FIITDNNIVFDQTQKFSLANIAPTILEYLNIKKPIEMAASSMIKKIHK*

>UUR10_RS01020 Ureaplasma_urealyticum_serovar_10_str_ATCC_33699_NC_011374 serine/threonine protein kinase

MREQIQTNSILNHKYKVVKHLADGGFSKVYLCCFLSDETKFIVVKVLDISDEKQQMVVYDELRISNLIKNSNSDKRSYIMEYYEYFESGSLETDDKRIYIVFEYIDGLTLREYLDEFKTVTYVKAVEIIRQVALGVSFFHSCNPQIIHRDLKPENCMINKTLSKIKIIDYGAASVFYNREDLTKDQEIKCTIIYASPKLLSLGQKVKEQASKGLNKNALSLINDALGVNYDIHSLGVMLYELITGTNPFSEHTIKDDRDYLEK*TTYDVEPLSSINKTIPKGIDNILIRCFA*KKEDNKLLYKDIYTLIDDLNNVMDPESSLNQDYIKPLNKLRIYRKNVAGLYEIKGKDRK*YLQK*FIILVGCLSIILIILLIIILSFKKSGAI*

>UUR10_RS01855 Ureaplasma_urealyticum_serovar_10_str_ATCC_33699_NC_011374 alanine--tRNA ligase

MLKLSTNEIRKK*IEFFESKDHLFIEPKSLIPKNDPTLL*INSGVSTLKDYFSGKVKPPHKRLVNSQKAIRTNDIFNVGLTSRHHTFFEMLGNFSIGDYFKKEAID*AYEFLIDVLKIDVKKLWVTVFEDDQFTYDE*IKLGIIKEQIIKCNRDRNF*DVGNGPCGPCTEIHYDRGEHFDPNKVGSKLILEDIENDRYVEI*NIVFSQFNNDGHNNYTELLQKNIDTGAGLERIACISQDVPTNFDSDVFMQITKSVEQFSEYKYDMNEYFHPNVAQNKINFAYKVIADHMRATVFAIADGAIPSNKERGYILRRLIRRTMVLVRRLNINNLL*VDAVVNAIASTMGDFYTYLKDEKTLAKIKMILNKEVQLFEKTLQLGLNIFESSIHNQELDKDITFKLVDTYGFPIELIKEICEQRNVKVDLEAFDAMFKHHQLVSKANKANLKVMESQNESLMQLDVDSTFHYEIFK*ENAKIITLFNEDFELVDGLDHEDGYVVFDNTCFYATSGGQQHDTGYIIKNDQQFFVDDVFKAPNRQHVHHVKNASLSMNEYVILQINEQDRKSITANHTAEHLLHYCLKQVLSPDIKQEGAAKYPHKVTFDFTYHAQPTKAQLDKLENVLNEMVQSNFDVQELHMDLDEAKAVGAAAYFEDVYKKLKGKLRVIKMGPSIELCGGTHAHHTSEIERIKIVECASKGAGS*RITMVTGHDNLAKYIHDLYVEYLNEINHLKANLDINDHKLNDLYNAFAN*KNLSIDDYDLLNEKFTELKQALINFKIEFDKQNAKQAIIDIKNTFNAQQTNKRIHVFKNTDNKNIFNALNELINENQNTLFISFNLDENKIQYLLAINEKFATTNQINLNKYIKELNTISNGKGGGKPYFVQGGTSEQEKLDELLTAIDK*VINA*

>UUR10_RS02230 Ureaplasma_urealyticum_serovar_10_str_ATCC_33699_NC_011374 tRNA (adenosine(37)-N6)-threonylcarbamoyltransferase complex transferase subunit TsaD

MEEKYLILSIESSCDETSLALFENNKLIAHKISSSASAQAFHGGVVPELASRYHEHNINRLFVDILNETKIDPLTITHVAYTAMPGLPGCLHVGKVFAKQLASLINAELVPINHLHAHVFSASIDQELVFPFLGLVVSGGESCLYLVSDYDQIKILNQTQDDAIGECYDKVARILG*NYPGGPIIDKNYQEDLATLEFIKSQPAAKNFSFSGLKTAVINYVHNSKQKKLDFDPIVIASSFQKFAINEVIKKVKYYLDLYQLKRLAIGGGVSANSLLRKKIRDLNVISYIPQMIYTGDNAAMIGAYAYALIKNHKKSILIK*

>UUR10_RS01110 Ureaplasma_urealyticum_serovar_10_str_ATCC_33699_NC_011374 50S ribosomal protein L23

MELTRVILHPYTTEKTYSIRNKSEHETLTFIVDKNANKYQIREAFIAIFGLKPLKIRTTNRGPAKIRTSTARPGYTKAKKIAYIVMPIGVKVAVSKEEVEAANAK*

>UUR10_RS02255 Ureaplasma_urealyticum_serovar_10_str_ATCC_33699_NC_011374 DNA polymerase III subunit alpha

MFINLNVHSYYSLLNSALSIDDLIQHALDNNQPYVCLTDLNNMYGCIEFYDKAKAHNLIPIIGLEFEYQNTTLVAYAKNYNGYLKLIK*SS*IMTNTTFIIQEDFDDLIIVCKKGGLVFENPNFYQAQNQNASNAIALQSVFYAQENDKTVFLAMLAIKNDLKLDDFIDCHEFDKNYFLNDHEAQSLFSTIALDNLNKVLNELQVEIHDLPINIPVYDKNNLTVSSEILKQLCISGLKQRLNAHDGQVKKVYAKRLKYELDVISEKQFDDYFLIVYDFINYAKSNGIIVGPGRGSAAGSLVAYCLYITDIDPIKHNLIFERFLNPTRKSMPDIDTDIMDEKRDQVIEYLFEKYGNDHVAYIVTFQRLKAKMALRDVGRILGIDLKVIDKICKNIKTDYDEDIDLAIKKSATLKEMYVLHKELFEISKKLIHAPRQIGTHAAGIILSNSSITNIIPIQLGINDRPLSQYSMEYLERFGLIKMDLLGLKNLTIIDNVLKMIYKTQNKKIDLFNIDYNDKFVFQDLAKAKTNGIFQLESPGMKKVLLKVKPQNIEDISIVSALFRPGPQQNIKTFVERRFKREEFSY*NEQTKKILEPTYGIIIYQEQVIELVKTIANFDIATSDNFRRAISKKDEKILMQLKDDFINGALANNYKQPLVNQIFEYIFSFAHYGFNHSHSLAYSYISY*LAYLKHYYPLEFLSVLLSHTSASKEKLLSYLDETKDFNISIKGPDIQHFSNDFVIDNHKQIIRFGFKTIKGFGDELLKKIKLALENAELSDYISYIDALKKGNISLKNIEILIRIGAFDSFEINRLFLLNNLEEIFEKTGLNGHFFDLNLVGLDYANDMSINERFQEDEIQYLGINLSSLNYTNYTNEIDYSNLKYEIESFNEINTNYEVNIVAQVLNIVQSKTKKGNDIFYLDVLVENKKEKLTIFQNSKHLVDEIDINGIYVFGVKLLNHFNFIVSVKQRV*

>UUR10_RS00415 Ureaplasma_urealyticum_serovar_10_str_ATCC_33699_NC_011374 DNA polymerase III subunit beta

MEVFVSIKKLIEAMKFSTTIANTNNANALLLGVLIEVNENKITFKTTNNQVSGYKEISDGFEYFSSGKILVTAKILLGLISKLKDKSVLLKQVDTNILLIKTENFETQINTMNIESFPSLNFSLEDYVKISLPHQIMQEINAKVLPNVLNSQGIEKIQPISGVLIDTETLDNQLIAIGTDKIKASCLTKPYLGEKFKFIISYSTMKLIMEVLRNVEYSNNQIVDFYVRNKSLVFKVNDAILQTRMIDGVYPNVYSIFNETNEEKNYVFDRRLLIEIIERGMNIVMQEQNPKISIKIENNEAEISLTTFEIGNMKEKMPIINLSNANVEFIVNPSLLAHVLKNFENNDVNFKVKDEILRPIIFIDAKDLGFKQILSRIKN*

>UUR10_RS00760 Ureaplasma_urealyticum_serovar_10_str_ATCC_33699_NC_011374 signal recognition particle-docking protein FtsY

MGFFKKIFNKILGKKDTGQVSEEISQKNEENRILKLEDNSINKFNEGLRKSSSALTNAINELATKYIDINEE*YEHLEEVLIGYDVGYVATNKIIESIRNEMIYQKVNDPELIKSIIIDKIFIYYIQDTEINTEINLKQNQTNVVLVVGVNGVGKTTSIAKITKKFINENKKVLLVAGDTFRAGAVEQLKV*AQRLNVDIELPIKEGQDPASVIYAGVKKGYEQKYDLVICDTSGRLQNKINLMNELKKIHDVIHKFDEHAPHETLLVLDATQGQSGINQAKAFNEVTKISGIILTKMDSTSRGGIVLAIKDAFNIPVKLIGLGEKLDDLSVFDLEMYVDSIVLGMKLDVK*

>UUR10_RS00550 Ureaplasma_urealyticum_serovar_10_str_ATCC_33699_NC_011374 ATP-dependent zinc metalloprotease FtsH

MHFFKKILNLFTSKIESEDNSVKKDDLTQPRKQSPEARKKRNRRIIF*LIVLLIIGTIIGVIIYFSVRKEYDSVIVKSAQTEIVNDKRVLYLNTVRPNSNQITRYTIDEDQLLAARVNILNNTNFQIISNASSLGLSRISFNIVREGVEKFKIGETSIYTPISGATNNQ*N*LTSIIAQNAGIPSSGFNAQVIISPLISIIFFAIFLYIILRVSKAQSDSLLGTNKGNAKLTKSSVRFSDVAGIAEVKEELIEIVDFLKEPKKYVAAGARIPKGVMLYGPPGTGKTLIAKAVAGEANVPFFQTTGSSFEDTFVGVGARRVRELFEKARKSAPAIIFIDEIDSVAKKRGNSLTAVQDQTINQLLSELDGFDTSSGVIVMAATNRLDTLDDAILRPGRFDRQISVNLPDILEREQILRIHSRNKNLSAKVSLEDIARRTAGFSGAQLENVLNEAALLSVRDKATSIHMNHLDEAIDRVIAGPSRPNKVISEREREQVSYHEAGHALIGLYSPGADVVQKITIVARGRAAGYTLQTPERNENILQNKTELISRVRTALGGRAAEELIYGPNEITTGAANDFYKITNIVRAMVASFGMTDVGLTQYIATEGVDNPYRNNYSEQTALAIDIEIEKIIQREYKIVKEMINEYREELELIVQTLLELETILKPQIDYIHQYKQLPPEVIANKNKREASQKQANSSVEEAKVVDDKEKDQKSN*

>UUR10_RS01145 Ureaplasma_urealyticum_serovar_10_str_ATCC_33699_NC_011374 30S ribosomal protein S17

MERSRRKVLEGLVVSDKMQKTVVVSVETKSKHPIYRKLVISHKKYHAHNDNDDAKVGDLVEITETRPLSATKN*RVSKILERAR*

>UUR10_RS01200 Ureaplasma_urealyticum_serovar_10_str_ATCC_33699_NC_011374 nucleoside monophosphate kinase

MKILLIGPPGSGKGSVSELLTKNNALKHVSTGNLFRAILKEDSELARKIKEINVSGGKLVPDEITNQVAKSAIDELIKNQQSFILDGYPRTINQALALEQYCDLDYIFYLDINHQELMKRLTGR*MCPKCAGIYNIHFKKPQVDGVCDNDQATLYQRADDHEDAVSIRLDEYDKLTLPLIKHYKTNPRFIKINANQPIKDVYEDINNYLKQNK*

>UUR10_RS01090 Ureaplasma_urealyticum_serovar_10_str_ATCC_33699_NC_011374 50S ribosomal protein L20

MRVKGGSVTRQRRKR*LEKAEGS*GTRNTSYRIARQTVIRAAEYAYRDRRNKKRDFRKL*ISRINAAVRELGYTYSQFMNALVKANVVTKDGQGLNRKMLSELAINNPEAFNQLVNKVMK*

>UUR10_RS02750 Ureaplasma_urealyticum_serovar_10_str_ATCC_33699_NC_011374 30S ribosomal protein S4

MSRYTGSIYKKSRRLGFSLLENNKEFNSGKKRTYGPGQHGNKKVKLSNYGQQLVEKQKLMFLYGLNDRQFRRLYRVALGRPGVLTLNLLQVLESRLDSLVYRAGFAPTRRAARQLVNHSHVLVNGKKVNIPSALVEVGSTIALKEKSLEMPLIKNTLNKPADFIELVDKDKKVAKLSRLPERSELPADVNEAYVVE*YNRLM*

>UUR10_RS00545 Ureaplasma_urealyticum_serovar_10_str_ATCC_33699_NC_011374 hypoxanthine phosphoribosyltransferase

MKDIDPRIKEVLITEEQIDQKITEAAN*INKEYEGKEPIMIGILKGCIPFIGKLLPKIKVDMKLDFLAISSFKGGTSAQTEPEIITDLKFEVKDQDLILVEDIVDTGRTIKKVYDLLKIRGARSIKLVTLVDKKDGRLVDLQADFACCDIPLVFIVGFGLDYKEIMRNLPYIGVLKEEVYQEDLNNKNEGDGE*

>UUR10_RS01175 Ureaplasma_urealyticum_serovar_10_str_ATCC_33699_NC_011374 50S ribosomal protein L6

MSRIGNRKLTIPANVNVSVESGKVHIVSQTAKLSVDFPVNLISVDVVDNTIKVSRANDEKQTKMFHGTVNANIANALVGVTTG*KKELEVKGVGFRAKVEGSKLNLGLGFSHPLLIQIPTGLKIETPSATEISISGSDKATVGAFAAVVRAYRKPEPYKGKGVMYKGERIVRKAGKTADKKK*

>UUR10_RS01865 Ureaplasma_urealyticum_serovar_10_str_ATCC_33699_NC_011374 leucine--tRNA ligase

MYNHNKIEKK*QKY*LDNKTFKFVDNPNNPKKFYVLDMFPYPSGKGLHVGHPKGYTATDVISRFKRLNGYDVLHPIG*DAFGLPAEQYALETNNHPHTFTQQNIKIFRKQLQMIGFDFDYDKEVDTTDPQFYQ*TQWIFVQLYKHNLAEIQDIDVN*CENLGTVLSNEEVVLNDKNERVSERGGHPVVRKPMKQ*VLKIVDYADKLLDGLNEVEFSESLKSLQRN*IGKSIGTNVQFKIKDSHLALDVFTTRIDTIYGAQYLVVAPEHPILKSIVSEQQASVVQAYVDQTKKISDLDRIADTNKTGVFSGTYAINPINQEIIPI*VSDYVLMNFATGAVMGVPAHDERDYAFAKKYDLPIKSVIDTKQSLPYTGDGLHINSPMINGLNIEQSQNILNDYLVKNHLAKRVVNYKLRN*IFSRQRY*GEPFPVLFDENNQIKIIEDLPVLLPNLDEFKPSKTGESPLANAQE*LYVEIDGKKYRRETNTMPQ*AGSS*YFLAYILKNEDGSYTPLNSEEAKKRFAK*LPVDVYIGGQEHAVLHLLYARF*HRFLYDIGVVPTKEPFYKVINQGMILGENNEKMSKSKGNVINPDDIIASHGADTLRIYEMFMGPLTASLP*SPDGLDAMRK*LDRVYRLYHNLSELEVVEDVNKLNEEIIITYHTLIKNYTKAINEQAFNIAISEMMVFVNVLYKNKVINYKLLDNFLILLSCFAPHLAEELYSLNHSESVCLQKMPIYDEQKIIAQNVTIPIQINGKLKHTINVLRDTNAEQLINLALACEQVKQAIGDQPIKKQIVVVNKIINFVI*

>UUR10_RS03360 Ureaplasma_urealyticum_serovar_10_str_ATCC_33699_NC_011374 transketolase

MNRYVNAMRSLALQAINKANQGHSGMSISAAPIIYTLYKGLMTISKSHPK*FNRDRLVLS

AGHGSMALYPVFYFSSLLTLDDIKNFRNDNHLTPGHPEVLSNNYIDASTGPLGQGVANAVGMAITESYLRAEFASLKGVVDHYTYCIVGDGDLQEGISYEAMSIAGKLKLSKLIILHDSNDYQLDSAVSDVNIEDLKMRVESMG*NYLKTDNNPENIFKAIAEAK*KKNVKPTFIEVKTIIGEGTSFENSNEAHAAAISKEELEKFGKRFHTKTNNFEFHQEIFDHFFFNVVARGESAYNQ*QQLVDQYMQTNPEQMQRLLNYINGNYEDLNKMLDENKIVNLSDSTRSYLKQYFAQLKDLKSALVLSADLAKSTFTKIGENAFNDDYKNPYIKFGIREFAMAGAMNGISLHQGAKAIGGTFLAFSDYMKPAIRLTAISNLANLFIFSHDSYAVGGDGPTHQPVDQLPMLRAIPNVEVIRPADHYEVKHALSYSFKQKQKPICLVTSRQAIKQINEQKPQDFTKGAYIINSPFSFSENPDYTIIASGSEVSLANDAAKEIFEKHQLKVKVISAFNLNLFLQQKPEVIKNLVSSKNGLLAIEASSEML**KLSVYTNKFMQIAANQFGRSADGNKLMHEFGFSVENIINQLLNKK*

>UUR10_RS00100 Ureaplasma_urealyticum_serovar_10_str_ATCC_33699_NC_011374 dTMP kinase

MMLTKNSNELKSPKKGLFIVFEGIDGAGKTSILKQLLDVLKEPKLVNKIFLTREPGGKNNTAAELIRDFFLKNLEAFDPLTLAYLYASSRAEHVKKTINPNLEKGHIVISDRFVHSSYIYQGIVQNQSLEVIHHVNQQAIGNLEIDYIFYFDVSVNNALNRMKNRFDNTNAFDSQNKQFYEKLLKQYPSVFNAYNQPKKIIFIDANKSENEVLCEVKERLLEIFKEHKYI*

>UUR10_RS01525 Ureaplasma_urealyticum_serovar_10_str_ATCC_33699_NC_011374 ATP-binding protein

MNNNEIEDELDLENFNYQKALEVPNLKAINLTEDEFNLHF*DIVGVYRSYLNNLKEPNDSGYIYELNRNEYNHLCLVVIKKESKVDKVKKNYILNTIKNMDYDISLTDDSQIFSKKSEILDNDLLVERNKLINFFLEEARKNKKQSANKEDNITTNDQQLKSAFIYGDFGVGKSIITQAYTNTISLKYNLKIAYITLNELFKNVIQFFNYKDISDSVVNELINELSNIDVLVIDDFSSGNLNY*SISTILMPIIENRLKSMKQTIFISNFSIEQLNNSTKNIANIEEQKAKLRLFNRIECLTYGNVFKIKGPSIFKVTNNL*

>UUR10_RS00720 Ureaplasma_urealyticum_serovar_10_str_ATCC_33699_NC_011374 hypothetical protein

MQSNTKLVKYSKIIANRLKAYSNQTRFIEIKTAFELNNEQKQRIKKTIINRFGDERPIKFIVDPSLIGGVSLKINLEIIDSSLKTKLNQIINIKEKEGA*

>UUR10_RS01835 Ureaplasma_urealyticum_serovar_10_str_ATCC_33699_NC_011374 asparagine--tRNA ligase

MQLKIKEIFDQDYTKLEGQKVQIKA*VRSNRDSKKIGFLVLNDGSSLTNLQAVYRVDKISNYEEITAARM*AAVAIEGVIKLTPTAKQPLELEVLNAQILKQSDEDFLLSNNDLSLETLRLNAHLRPRTNLFHAIMKVRATLAFAVHEFMNQNEYS*LAAPLFTGNDAEGAGETFSIQKFDNEEFFGKQTHLSVTGQLQAEAYAQAFGNVYTFGPTFRAEKSHTNRHLAEF*MIEPEMAFVDLKGMQDIVENLIKHVIKAVLEKNQQELEFLAQRNDENLIKKLQKVVDSKFERIEYKDAVKILANAVKSGHQFEDNEIFFGMDLGSEHERYMCETYHQGPVFLQNYPKDIKAFYMKLNDDQQTVASTDLLIPGVGELVGGSQREDNYEKLLKRCQELKMPIESLQ*YLDLRRFGYYMSSGFGIGFERLVMYVTGVNNIKDTIPFPRSHGQIEF*

>UUR10_RS00620 Ureaplasma_urealyticum_serovar_10_str_ATCC_33699_NC_011374 dihydrofolate reductase

MLKLI*CQTLNGGISKNNKLP*YVKEELEHFYKTTKNHKIVMGKSTFDSLEQKPLSNRTNIIFSSIMQTPEDQSYFVTNDFQQLLNDAKKEDIFIIGGKELFDIFLNHADALIVSVLNDYYDCNLYMKVDYNNFNLDKKDVYDNFVVNYYSSKKDK*

>UUR10_RS00050 Ureaplasma_urealyticum_serovar_10_str_ATCC_33699_NC_011374 50S ribosomal protein L10

MANVRPSVVFKQQEVDHMADILKNSKSFIVFEYHGLTAANILALRNVLHSSNSKLFVLKNNITARAFEKAGVTGFEDRLTGPNAIAVAMDDEIAAIKAVNDVAKEFDFVKIKGAYLENKFADTHKIDQLAAIPGREGLYSMLLSCFTAPLRNVLYGLKAVAEQKGE*

>UUR10_RS00700 Ureaplasma_urealyticum_serovar_10_str_ATCC_33699_NC_011374 F0F1 ATP synthase subunit beta

MTEVKKGKINQILGPVVDVRFPSE*LPEINTALELNNHGSKLVLEVSQLVGDNIARCIAM

DTTDGLVRGQEVINTEKPIMMPVGKQVLGRMFNVTGDPIDEQPAPTGKRMPIHRPAPSFAEQAEAIEILETGIKVVDLLVPFAKGGKIGLFGGAGVGKTVLMQELIHNIAKNHGGLSVFAGVGERTREGNDLYYEMAESDVLDKTALVFGQMNEPPGARMRVALSGLTMAEEFRDAFGQDVLLFIDNIFRFTQAGSEVSALLGRMPSAVGYQPTLAFEMGQLQERITSTKKGSITSVQAVYVPADDLTDPAPATTFSHLDAKVVLDRAIASLGLYPAISPLQSTSRLLDPLVVGVKHYSVARRVIEILQRFMELQDIIAILGMDELSEEDRQLVMRARKVRNYLSQPSHVAEKFSGQPGLSVKLEDTIEGFRKILDGECDDIHEQHFLYVGKIDDVFEKVAKSK*

>UUR10_RS00870 Ureaplasma_urealyticum_serovar_10_str_ATCC_33699_NC_011374 DNA-directed RNA polymerase subunit beta

MSQKGIKSLTISIASPEQILS*SKGEITKPETINYKSLKPEPNGLFDESIFGPSKDYECYCGKYRKVKHKGKVCERCHVEITESIVRRERMGHIELAAPVAHI*FTKELPSPSKISLLLDITYKEVDQVVYFVNYIVLDEGNNEYDGKSIFNKKEVLDLTSPKNSIRSRNKLRRTLRNIQERIEEELNHEREALIQDFDYRLAVTYDQMLKDSNIPFSVKDVMAFIEKHTGVRFGIGAEAIHELLEKLNLEEEHEKIKQAIQNSPNAYDQKTKRLLRRLECVR*IKDSGSKPE*MVMTRIPVTPSETRPIISLDGGRFTTSDTNNFYRKIIIRNERLKQMQATDAPEILLDNEKRLLQEAVDSLFDNNSRKKPVVGKDKRPLKSLSNHLKGKQGLFRQNLLGKRVDYSGRSVIVVGPELKMYEVGIPALMILKLFRPYIISELIRKRDEFGNEIQPICANIKLAEQKILAQDDEI*PVVEKVIKQRPVILNRAPTLHRLGIQAFEPKMVDGKAIRLHPLVTTAFNADFDGDQMAVHIPLSKEAVAEARSILLAS*HILGPKDGKPIITPTQDMILGIYYLTKEKFPQPIEEMILKDPVQARIEFINHFHIFATQDEAIRAYKLKTIRINDVIGITTKAFDNKSFSKEGILVTTVGKIIFNQAFPTNFPYINDVKNLYGDNQFEIIGMHESILDYLKAYNLKEPLTKKTLSTVIDYLYKVSEIEVVPQTMDKIKALGFKYSMISATSISAFDIPSYDQKYEYFKETDELVAKLREFYLDGKLTDDERYTKVVQA*SQTKDKVTHDIEKLINSDEYKDNPIVIMAKSGARGNTSNFTQLAGMRGLMSKSYNYDQKNNSGVIKDTIEIPIKHSFIEGLSVSEYFNSSFGARKGMTDTAMKTAKSGYMTRKLVDSTQAVVIKGNDCGTKEGIIVREIRNTKDNTSIESLKDRIVGRFSINPIYDTKNKLIIEGDKLITNEIANMIQNSGIREVEVRSPLHCSSLYGVCQKCFGLDLSTNKLIETGTAIGVIAAQSIGEPGTQLTMRTFHTGGVAGDTNITQGFERIKQLFDCIQPQENEKAIISQVKGTVDRIEKDSNTNGYNVVIKYNKDNFVSYPTRPNAVLRIKTGDNVVAGQKITEGSIDVNDLLKYAGIENVRHYIIKEVQKVYRMQGIEISDKYIEVIISQLTNKVTITNPGDSGLFVGETISINEFTEVAQSMLVNKKKPPSAINQVFGLDHAPSKSGSFLSAASFQDTKKILTDAAARSQKDMLIGLKENVILGNLIPAGTGLKDVEEVIAYGEEMYKKQY*

>UUR10_RS02455 Ureaplasma_urealyticum_serovar_10_str_ATCC_33699_NC_011374 phenylalanine--tRNA ligase subunit beta

MILSLNLLHKISPKLKKISLNELCTALMDLGCEVETINTIKPSTNLVFAKVLEKTKHPNANHLNLVKVKANQEVYEIVCGADNFNVNN*VVLAKINAELANGLKITPRELRGYVSNGMLCAYSEINPEVAHFLGQTDLDGILVLHDSYDHYKTPNQIFNLDDVILDLSIPSNRNDLNGYF*MAKELCAYFDLEYVIDATINHRSHKEIVNVRILSDDVNSYGMIEVKNIQNYTLK*NTKSVLVNNQIKIVNNFADNMNFLTLLTANPLHAFDAHKISGQIIVKNAEEDSILLGLDQKEYAIKKGDLIIVDDQKILALAGIIGSNDSKIDNNTTTAYIECANFNPLLIANTARRLKINTTAAMRFSKPLTNYVTKATLKKLLAHFKLDAKLICYFKHLVHNVIKNKIDQVSDFVGTKINLDTAHTFLKRLGYKINKTNLITPSHRYDVLNEFDVYEDIMKKFSIQQIKPQPINFDILSFKNNIAYDFEKKVSDFLVDQGLFECKTYNLKSQTQAYEIDFFNFQQAYEINNPISNIRSHLKLNNLNSLLEVLEYNQNQKNELENIFEISKINPINSNQQTILSIVLCKPLINAKLNDSIVVNNFVTTKALLHVLLTKLNISYVYDTNHIVNELYENNQLALVNENKQVFGFIGQLKNQIKKTYGLNNDIFVINLNLTSYLNQEQAITKVIKPSVYHDIVRDVSVKLASNVDLNDVMDNIEKIKNIRKVEISDLYVKDDEIIYTFKYYINDYSSNLSSEQIAVIEQEVNNYLKQF*

**Supplementary Sequence file 3 Human non-homologous essential protein sequences**

>UUR10_RS03090 Ureaplasma_urealyticum_serovar_10_str_ATCC_33699_NC_011374 50S ribosomal protein L1

MAKISKKLSAAYEGIDKQKAYPLFDAIKLAQEKSITKFDGSINIAVKLNLDTTKVEQQLRGSISLPNGNGKNVRVLVLSEDITKEQAAAVGADYFGGADYIQNIEKMLNQIDVIITNQKMMPLLAKLGKVLGPRGLMPNPKIGTVTNDVLKAVEEFKKGRIEYRTDTYGNIHMSIGRVSFETAKIEENANALLSLIRSKKPATVKGQYIQNIAISPTMGPGIKVIINNN*

>UUR10_RS00315 Ureaplasma_urealyticum_serovar_10_str_ATCC_33699_NC_011374 SsrA-binding protein SmpB

MIVSNKHARRNYELLEFFECGIVLKGTEVKSISRANCSINEAYVQIVKNEALILNMHVASFFEGNNFNQDPYRNRKLLLHKKEIIKLQHLVQTQRMTIVPTKIY*KNNKLKVEIALAKGKQLHDKREDLKKRDLARESRLF*

>UUR10_RS00735 Ureaplasma_urealyticum_serovar_10_str_ATCC_33699_NC_011374 ATP synthase subunit C

MSSFIDITNVISSHVEANLPAVSAENVQSLANGAGIAYLGKYIGTGITMLAAGAVGLMQGFSTANAVQAVARNPEAQPKILSTMIVGLALAEAVAIYALIVSILIIFVA*

>UUR10_RS01165 Ureaplasma_urealyticum_serovar_10_str_ATCC_33699_NC_011374 type Z 30S ribosomal protein S14

MAKKSLIAKQKKHQKFAVREYTRCVRCGRPHAVNRKFGVCRLCFRDLAYAGAIPGIKKAS**

>UUR10_RS01565 Ureaplasma_urealyticum_serovar_10_str_ATCC_33699_NC_011374 inorganic diphosphatase

MKLNVTIEIPKNSNIKYEYDRATKEITVDRILYGSMVYPHNYGFLKEALDYDGDELDVLVFADQAFQPGIKVPARILGAMKMIDGGETDTKLLAVIDVDPRYKHINTFKDIPLH*LAEVQDFFENYKNLQNKKVEILGFEDEV*AQKEYEECVALMQEHGHLKKDEFVSKMMKQRPEKYSQ*

>UUR10_RS01080 Ureaplasma_urealyticum_serovar_10_str_ATCC_33699_NC_011374 translation initiation factor IF-3

MNTPNNQRHMSSNNDARKNQPLINDQIRFRTMVVIDDHGNNLGEMNRIDALNLATSKNLDLVVIAKKGNIPVTKILDYGKYKYEQKRRQKESRKNQTIIKVKEIKIKPMIGEHDLKVRAENAKR*LEDKDNVKFVIEARGRMCTKDEFILQAYEKFIDLIKDYGTVVQANKKVSNYRYETIIEPIKK*

>UUR10_RS01340 Ureaplasma_urealyticum_serovar_10_str_ATCC_33699_NC_011374 nicotinate phosphoribosyltransferase

MGPVIPNTRLIDFKFDRDLLNKAYTSHYFIKTCKIIELHAPSHSVIMQFTHFSKTPIMVCGTSEVLALLEFCLSRKELKQLKIYYVPDGHVIKPKEALFAIEGPYEIFG*LENIIDSILARRSSVATNCYNVLNVINDEQKVIYMSDRSDDYSLQPYDGYAAAVGGMQYFVTQKQVEFLKDINYECKVMGSMPHALIQQNNGRVDLACEMFAQTFPNDPLIAVIDYNNNVLNDLEQLRYMFDRLYAVRIDTAKDLIDNSLLSTFDNVRNHDLHGCNPYLIDLVREYLDNNGGEHIKIIASSAIDLNSIKNFNKHNSAIDFYGIGTYLTHLSIHITADLVCLDNVYGAKVGRKIAKNFAEMTLY*

>UUR10_RS00010 Ureaplasma_urealyticum_serovar_10_str_ATCC_33699_NC_011374 50S ribosomal protein L31

MKDIHPVSKPCVYNCVTCKKEFVINSAAKNTEVAIEVCSNCHTFFIGKQNATTTLRGRAEKLNNRFEAGLNNINKKPEKKKVQGKSEPRKSLNEL*

>UUR10_RS01150 Ureaplasma_urealyticum_serovar_10_str_ATCC_33699_NC_011374 50S ribosomal protein L14

MIQHMTRLKVADNTGAKEVGVIKVLGGSKKRYASVGDIVVVSVKKATPAGLIAKGQMAKAVIVRTKKSIRRESGLLIRFDENACVLIKEDKTPRGSRIFGPVAREIRDRGYTKIASLAPEVL*

>UUR10_RS00950 Ureaplasma_urealyticum_serovar_10_str_ATCC_33699_NC_011374 30S ribosomal protein S15

MAVSKQQKHDLTVKFGGSASNTGKTEVQVAILSAEIDSLTTHMIENKKDKASKRGLYKKVAQRKKLLSYLQRVDIERYRALIKELNLRG*

>UUR10_RS01095 Ureaplasma_urealyticum_serovar_10_str_ATCC_33699_NC_011374 30S ribosomal protein S10

MNQELRIRLESYDHRLLDDTVKTIVDISNSTGSKLRGPIPLPTKKEIFTILRSPHVNKSSREQFERRTHKRLIILENPQPKTMEALKRLSVPFGVEVTFKI*

>UUR10_RS01025 Ureaplasma_urealyticum_serovar_10_str_ATCC_33699_NC_011374 ribosome small subunit-dependent GTPase A

MRAKITSVIVNNFYVYIYDLKIETKAIPKGIFKHDSHELKPMVGDDIEVELVDGVYLIVKIYDRYNQLIRPKVANVDIVLVVASIVQPDLNTLTLNKYLAFYEARNVKNVAIGLSKYDLASDSLKQKVDQLILDYQRNNYKVFVLTNEHDISLLKKFIKKHTLCLAGNSGVGKSTLINKLDPSIKQRTQEISQFLNRGKHTTTSTKLISFANGFLVDTPGFGNLEVNLTKNEMANAFSDFANYARFCKFSNCLHIDEPHCAIKKAVNDDQIVN*RYDDYLKIMKKLPNDVLEIKTRNQNKK*

>UUR10_RS02135 Ureaplasma_urealyticum_serovar_10_str_ATCC_33699_NC_011374 holo-ACP synthase

MKLVHGIDIIE*NREELNNPSFAKRILVDDELKYYLQLNSLKEKNRYLASIFASKEAVMKAFKLKYGYNDILILKTKNERQVYLNKILIKELVLSISYTENYVVASVVGLINTVESNS*

>UUR10_RS01195 Ureaplasma_urealyticum_serovar_10_str_ATCC_33699_NC_011374 preprotein translocase subunit SecY

MTNKQKKKNAFRQLLMIFKNKKVLVALIVTLSILILFRIGSVIPMPYIKLNGNFGNQGSFFSIINLLGGGGLSQFSLFAIGIGPYITAQIIMQLLSSELVPPLAKLSKSGERGRKKIEVITRIITLPLAVMQAVIIINLMTRANGFISIVPNAPFAIGSPLFYVTYIFLMVGGTYISLFLADLISKKGVGNGITLLILTGIVASLFNHFIAIFSNLGSLTSSKVSQIIGFILYILFYIMILIGVVFVNNSTRKIPIQQTGQALILDHEKLPFLPIKIMTAGVMPVIFASSVLAIPAQVAEFLDKQSMGYYVIHNYFIVDS*TGLAIYVVLILLFTFFFSYVQLNPPKMAEDIKKAGRFIPGVQVGMDTEKHITKVIYRVN*IGAPILAFLACLPHLVALVAKTINHGIPVIQPSTIFGGTSIIIMVTATLEL*NAIKSTSTSTSYAYQRKELETAITISVESDKSSKSQI**

>UUR10_RS01140 Ureaplasma_urealyticum_serovar_10_str_ATCC_33699_NC_011374 50S ribosomal protein L29

MSSIAQDLRKKDSLELEKIVIELKAKLLELRFAAANGEAEKLHTAKEIRKTIARALTILNERELAEKLNNKEANK*

>UUR10_RS00430 Ureaplasma_urealyticum_serovar_10_str_ATCC_33699_NC_011374 DNA topoisomerase (ATP-hydrolyzing) subunit A

MALKKPKKSRLTTEEIKQQLEGSTIKEQSITKEVETSFLDYSMSVIVARALPDVRDGFKPVHRRALFAAFENGMTHDKPYKKSAR*VGDVIGKYHPHGDQAVYQTIVRMAQEFSMRYLLVDGHGNFGSIDGDSAAAMRYTEARLSKISYELLKYIDKETVDFVPNYDASEQEPSVLPSGFPNLLTNGTTGIAVGMATNIPPHNLTEVCQAIKAYAKNHDISIPEIMEHLKGPDFPTGAEIYGDSGIINYFNTGRGSVTIRSKYEIEDIGQGRVAIVVTEIPYMVNKVNLIEKIVELVTNKQIEGISDLRDESSRDGIRIVIEVKRDVIPEVLLNKLFKTTALQTNFSVNNLALVNGVPMVLNIKEMIKYYFEHQIEVLVRRTKFDLRKAKERIHIVEGLVIAVNNIDEVIKIIKASGDDDIASKALIARFGLTELQTKAILEMRLRALTGLNIDKLKKEYEDLLLIIEDLEDILENYDRQVNIICENLDYLIEKFGDERRTEIMYGVSSHIDDEDLIPVEDIVVTMSKRGYFKRLPIDTYKNQRRGGVGVQGLKTYEDDDVEKILVANTHTDLLFFSDLGRVYRLRGHEVPLGSRQSKGIPAINFLPIEKSESILTILPIDNYEQGSLFFTTSKGIIKRANLSDFESIRANGKIAITLKEGDKLFSVMQTLGNDEVFIGASNGNVIRFNENDAREMGRIATGVKGINLEDDEYVVGTGLSSHGEYVLAVGSKGLGKLTDINDYRLTKRGAKGVNTLKVNDRTGNLVSIKVVNRDEEALIITTSGKVIRLSIQDISVIGRNTSGVKLISLENKEEVKSIAIFKKEEIDDNDDEQKTSHGNEHNLE*

>UUR10_RS01230 Ureaplasma_urealyticum_serovar_10_str_ATCC_33699_NC_011374 DNA-directed RNA polymerase subunit alpha

MRKFLKYQLDVPSINSEDKNRTVVKIAPLEIGFGDTLGNALRRICLSSIPGASMFAVKFGGYSHEFQPYEGVKEDITHIILNLKNLAIKIDELIYSEDYFNNLLIDK*PKMKINFKGPGVITAKDIVCPVGFEIVNQDLYIAEVTKPIDVEIEIFAKTGRGRVDFNTNKDFVSTLHIIATDSNYSPVLHYAYNVEMIKDSKSSMSEILTIDIATNGTISGSEAIAIAAKIMQAHLEPIMNIDKTINEMIIMREREEEEKRQNASISIDDLDLTVRAYNALKQSGINTTAELIELTKSQLEKIKNLGRKSVTEIIQKLTERSLELKKD*

>UUR10_RS03240 Ureaplasma_urealyticum_serovar_10_str_ATCC_33699_NC_011374 tRNA (guanosine(37)-N1)-methyltransferase TrmD

MKISILSLFPELYETWINHSIISNAIKNNQVTIEIINFRLYTNDKHKKVDDYQYGGGAGMVLMIEPIVSAIRAIRTPNSYVILTTPKGQVFNQELANEFVSKYDHIIIIAGHYEGFDERINYYVDAQYSIGDFVLTGGELPSMVISDAVIRLLDGVISSSSLESESFNNYLLDYPVYTRPVVFEGHQVPDVLLSGHHKNIADFRKQQQEMITKKNRPDLYQKYLNSKK*

>UUR10_RS01445 Ureaplasma_urealyticum_serovar_10_str_ATCC_33699_NC_011374 elongation factor P

MATIIQAKDLRAGHTFLYKGSIYQVIENSFNKTAMREGIVKCKVKNLRTGAITVEVLTGEKVEQAIIEKSKMTFSYDDGSGYVFMDNETYEQISIPYNQLS*EKNFIEEGTEVSVMRYDGELMGVSLPDQLVVTIVEAEEAVQGNSVQNATKRA*LASK*EFQVPQFIKSGEKVIINPSNGQYVGRAK*

>UUR10_RS01155 Ureaplasma_urealyticum_serovar_10_str_ATCC_33699_NC_011374 50S ribosomal protein L24

MNRIKKGDTVVVISGKNKNKSGVVIQVNPKEQTALVEGVNKIKRHQKKDQTHEQSGIIEKEAPIRLCKLALVDPKGKDKGKATKVKYLLKDNKKVRVARKSGSELDANKK*

>UUR10_RS02205 Ureaplasma_urealyticum_serovar_10_str_ATCC_33699_NC_011374 nucleotide exchange factor GrpE

MSKNNENIKHQNEGKLHDQVDKKETKNHAKQEFKYKELYEHELKKNKELQNVNELLINKNQQLEIQINQLNQDFVKQLETKTKQAQEILEQKVNELEARHETKVNDAVFKIFKFKMEPLLDAINHFTKIVNQNYDDPKIQAFIEGFKMFSQNMIDGLENLKITKISPQINDMLNDDTMEVFEVVQNTNKPSMHVTEVISDGFKYNDKVIKFAVVKVAK*

>UUR10_RS01170 Ureaplasma_urealyticum_serovar_10_str_ATCC_33699_NC_011374 30S ribosomal protein S8

MYLDPIAELITKINNGRKAHKAEVSFATSKLKTAILELLVKEGYIKSYDIRPTENNKSETVVKLKYKNQTTSSINGFKQISKPGLRIYSTHLNLPKVLNGLGIAIITTSKGVMSDKQARKENVGGEVIAYV**

>UUR10_RS03455 Ureaplasma_urealyticum_serovar_10_str_ATCC_33699_NC_011374 50S ribosomal protein L34

MKRTFQPNNRKRAKVHGFRARMKTKNGRNVLARRRLKGRHSLTVSGEK*

>UUR10_RS02910 Ureaplasma_urealyticum_serovar_10_str_ATCC_33699_NC_011374 UMP kinase

MSKQRIVIKISGACLRQDDNSIIDVNKINDLAKQIKEISKKYIVSIVLGGGNI*RGHIAKELGMNRNLADNMGMMATIINGLALENALNNYNVDAIVLSAIKCDKLVYESSANNIKKAIEKEQVMIFVGGTGFPYFTTDSCAAIKAAETESSIILMGKNGVDGVYDSDPKTNPNAQFYQHITFNMALTKNLKVMDATALALCQENDINLLVFNIDKPNAIVDVLEKKIKHTIVSK*

>UUR10_RS00095 Ureaplasma_urealyticum_serovar_10_str_ATCC_33699_NC_011374 DNA polymerase III subunit delta

MKYSFANLLIQSPKTSLTLGVEQIMLAFINEKNHEQQAYYINKVKNNQYFDLKIYDSLSMKKSDVIDLQNAFLYDGIEDINLKFYLIKNIDLASKYVLNALLKFIEEPPKNTIAIFSTKNLNQVLKTIKSRCQLFYLPANYDLYHQLIKQINQPISATECDLIFDDLDELKTLLENNEINEVLAYHAKLNDIKSFETLNDLKETFKNLSILQIHYLLKLIFIKINNINSKQAILDLMRANLKININKNSLFTIIYTIIIENRGD*

>UUR10_RS00740 Ureaplasma_urealyticum_serovar_10_str_ATCC_33699_NC_011374 F0F1 ATP synthase subunit A

MENYNPLDIMIALPHIAAIIIVTLIIATISLIYFSMIRKLTVHDVPNRFVIIIGMIVDYFRGLVVDTMGAKHVKLAPYVLFTFCYIFTANLVSLFGFKEATTASSVPLAMALATVVGGQIVALKYQKASFFLKFTFKIKGFPIMVNPLEIVSKLTPIISLTFRLWGNISAAAILLNITY*AFAGFTNVVP*VGVSLIAAVIILPILIGYFTCFAGTIQAFVFTLLTSIN*GLEIKEGEEHYAHLAHKKAEKLAAKKLAELDAQNQAQNNEVQVVL*

>UUR10_RS01130 Ureaplasma_urealyticum_serovar_10_str_ATCC_33699_NC_011374 30S ribosomal protein S3

MGQKVNPNGLRFGINKQ*LSR*VPTDQLQMAK*LVEDDKIRKYLSTKYKNAGIDHVEIERDQQRVNVYVYAVQSGLLIGTEASEKKLIELAINKIVGRKQLVSLKVVEVQIPELQASLMAREIADAIENRVSFRIAQKMVIKKVLKAGARGIKTHVSGRLGGVEMAREEGYTQGVMTLHTLRADIDYSMQEAHTTYGIIGVKV*INRGELFGNKLVNSVAHAANKEFSRSSKPKKGSFNRSSRSKNTKPAPKQAVSE*

>UUR10_RS00730 Ureaplasma_urealyticum_serovar_10_str_ATCC_33699_NC_011374 F0F1 ATP synthase subunit B

MLDKRREYIAKEITDAENAKQEALQYLENAKSEHLAAQAETAEIIAKAKSESLTLRELLEKEAREAADKIISSAKISIANERRENLERLQTEAREAAYIAAEALMKKELSREDNDKLVDQFIKELETNEK*

>UUR10_RS02250 Ureaplasma_urealyticum_serovar_10_str_ATCC_33699_NC_011374 5-3 exonuclease

MKKAIVIDGNSLIYRAFHATYKQAE*AVENQLMPTNAIKLVASMIFKILNEDQFSYALIALDASKKTFRAQEYAAYKATRKPMDEKLVVQLPYIKKLFTAMGFHIISQPGIEADDFVGSFSNLMSKSNIDTIIYSTDRDMLQLINPNTKLKLLKTGTSIVQEINLANFALLNNGLLPKQIIDYKGLVGDSSDNLVGVKGIGPKTAINLILKYTNLENIYANLEEITPSVKNKLIEHEKMAFLSKKIATIQTDLLLDETLENFILKPYNIQELDTLFESLKINNMHNYYK*

>UUR10_RS01585 Ureaplasma_urealyticum_serovar_10_str_ATCC_33699_NC_011374 transcription termination/antitermination protein NusA

MSNSFKSKEFIEYFKDTAKQNEIELEVLSSIIKEAFEKTYLRTHPGENFETNINLKEGTINCFRNLVVVENEKAHNEDLETCLDDAVEILLDDARKINANAQIGDTIKQYISIDDFKSIEVGQIGSLLRQKITEIHNKRVADF*KPSLMKMIRAKVAEINYNKQRNEITGVKVELDDQ*KTLGYLSRKDRIGDEKFKVGETYDFIIKEVKEQSRL*PVLLSRTEPELVEEILKREVVDIKNGNIEIKKIARIAGFKTKVAVSTNLLNIEPVAVVVGNKGLTITSISKQLNNERIDVIRYADDKRIFIANAIGLDKLKGLLVQENESDQRSAIAIVSKEDLPSVIGRGGANIRLIAKITE*NIDVKTIEQAFEENVVYEKFDEKIYRS*NIESINKKNVTNDEMLALIDNMQDEKVEKTEQVKDQLKQQEKQTIVSNNDDSENDDEQLEYLEGFEDFKF*

>UUR10_RS01665 Ureaplasma_urealyticum_serovar_10_str_ATCC_33699_NC_011374 50S ribosomal protein L32

MAVQQRRVSKSRKGMRRSHDHLTVSNTVACNECGKALLPHRACRDCKTYRSIKLSIK*

>UUR10_RS02195 Ureaplasma_urealyticum_serovar_10_str_ATCC_33699_NC_011374 glycerol-3-phosphate acyltransferase

MDQVYSVAMAYILTLIISPLYSYLIGSLNASIILSLLLKKQDIRHFASKNAGMTNMTRVYGKKLGILTLFLDIVKPIITISLTYIIYKYALNAPFVLSNGFNQAILVYFGGIFTIIGHCYPIFFKFQGGKGVASYGGFLITIDPIVAVIGIITLLIILLITKYMSLSAMITATITCFLVLIPGINYIPYYNEHFVEYLFDLNHVIKGTWYV*LFLLISASILIYRHKTNILSIATKQERKTFLFQPKPKNNI*

>UUR10_RS03135 Ureaplasma_urealyticum_serovar_10_str_ATCC_33699_NC_011374 AAA family ATPase

MSTKDLNDAILDLFCLVINNNDF*KDVILRLEAKDFPEKVQQNIFNTIANLNEQKYKISESNILNGLGNYVIVDEQDQNYLLHKNYLVQILERTDYLVDLKDCIEIIKNASIKNKLDLFANEILSTQISLTNAKDQFKEMHEKFLEILASRTEDTIENMELIANRYFEKLNKIGNSGIIPGVIKTKYDNIDKFTNGYKPGELVVIAARPGIGKTTFCLNVMVNNVNEIIEYNQNIQPNQKEKIIVMFSLEITKEQILQKFISIKTGISNREVIENKYRIAKGYDTRSFAMQAINEIKS*PIFVDDRPNISIVDIEAKLYDLKKRYDIALVVLDYLQLVSAGNANKNMTRTQEVGRVSSALKVIAKEINAPVIAIAQLSRKAEERDVSSNANMKNNPLVKTIDNSPKLSDLRESGSIEQDADVVAFLH*DRKQRNAMQNDNQETRMRDDLIEAKFIVEKNRNGSTGETDIIFSKLNSKFIRATTSKE*

>UUR10_RS02905 Ureaplasma_urealyticum_serovar_10_str_ATCC_33699_NC_011374 ribosome recycling factor

MNFKIYETKIREEFELVLK*MHNEFIKLRTGRATPAILDGILVDYYGSMTPINQLANISVPEPRVLAIKPYDRSSIKDVASAINASNLGVNPQVDVDIIRLTFAAPTEEVRKNLAKKAKQVGEEAKIRVRHIRQEAQDLFKKNSSTVEDDKKFFQTELDNLTKELNKEIEAVVSHKEKDIMTV*

>UUR10_RS01370 Ureaplasma_urealyticum_serovar_10_str_ATCC_33699_NC_011374 HU family DNA-binding protein

MSEKIKAKTRVQMIDELSKMLNIDKKQTKTFMDTYEAFLILELSRAKEVRFGNIGKFKVTVRAERKGINPKTGETVIIPEKTIPKFTFTKGIKEIINAGISVEDETVFLDDNDYEDDGDEFVEEYIAPESN*

>UUR10_RS03415 Ureaplasma_urealyticum_serovar_10_str_ATCC_33699_NC_011374 class II fructose-1,6-bisphosphate aldolase

MFSLVNAKKMVQNAYKNHYAIAAININNLE*IKAALLAAQETNSPLLLATSEGAVKYMGGYDNCYAMVVNLMKQMNIKTPVCLHLDHGTYEGCIKAIDAGYSSIMYDGSKISIQENIENTKKLLAIAKSKNVSVEVEVGSIGGTEDGITSEGELANVNDCYQMCLLDIDMLACGIGNIHGLYPEN*KGLNFDLLKEINIKVNKPIVLHGGSGISEEQILKAISLGVAKININTECQIAFSNALQDHLIKAGDLVAAKQYDPRKVLAYGVDAIKNTIIEKFTKFNSLNKA*

>UUR10_RS03055 Ureaplasma_urealyticum_serovar_10_str_ATCC_33699_NC_011374 50S ribosomal protein L33

MAIKRGVRLQCNESKSINYITTKNAKNNPDKLSLNKFCPKCRKVTTHVEIKKK*

>UUR10_RS03150 Ureaplasma_urealyticum_serovar_10_str_ATCC_33699_NC_011374 single-stranded DNA-binding protein

MNKVILIGNLVRDPEARQIPSGRLVTNFTVAVNDNIPNANANFIRCVA*NNQANFLTTYLKKGDAIAIEGRIVSRSYVDNNGKTNYVTEVYADQVQSLSRRNQNANDHNNDKVNVDTMMGAYASINTDAAFSSNQPQTNFQSTTSNSNKNDDEEDEITS*INLDDDLE*

>UUR10_RS03305 Ureaplasma_urealyticum_serovar_10_str_ATCC_33699_NC_011374 50S ribosomal protein L13

MQKSSMLKKEAAIARRQ*YLVDATDLVLGRLSVKVADILRGKNKVDYTPNVDAGDYVIIVNSDKVVLTGQKALREN*YNHSHYIGGLRTRSGEEMISKYSDELIRRSVKGMLPKNKLSKQILNKLFIYKNDKHSHEAQQPTILELKLK*

>UUR10_RS01190 Ureaplasma_urealyticum_serovar_10_str_ATCC_33699_NC_011374 50S ribosomal protein L15

MQLHNLEYKKGSRNHKEKRVGRGHGSGLGKTSGRGQDGQKARKSGMVRLAFEGGQTPLYRRVPKVGFNNDRFANKYNVVTLISLVKYETKELTAEFMYVNKIAKNEDLPIKVIGNAVLPSGTVVSAHKFSKGALESISNSKAKAQILE*

>UUR10_RS00405 Ureaplasma_urealyticum_serovar_10_str_ATCC_33699_NC_011374 tRNA lysidine(34) synthetase TilS

LTKL*TNLINKITNKKYLAAVSGGPDSMAMLNMYKRNISVVCHVNYHKRESADRDQEIVVDFCKKNNLPIEILDVDEKVYEKYAHIDNFQAKARLIRYDFFKEIGKKYNIQHLYIAHNFDDFLETAYMQRARQSKALFYGIKESNVVNGMIVKRPVLFIRKQTLQRYCDENKIKYGIDETNELDIYERNRVRKTISN*SLNEVYDFKKAVLKYNKEHSSFANFVELSYIEFKKNKYRYDYFVRQDDGVQYYLIYYFLIDQKISNPNENKIISLIKFFGKQINKEKAYRVQENLYMHVNEDDLISLISYDKNDVIDDPNIIEKQAGN*

>UUR10_RS01775 Ureaplasma_urealyticum_serovar_10_str_ATCC_33699_NC_011374 riboflavin biosynthesis protein RibF

MIIEITSTNIQQIRDQYFINELVIGFFDGIHLGHMNLLSDPNNQTILTFKNIPRKIKKLYDFNERIQQLEDLGFKRIFIYDIDQNNLSGEEFIDQILKPLTPKKIIVGANFTYGNNFCNASSLKQYFNVEIKIITNDVSTTKIKELIINKQVEIANKLLIKPYYRVGNVVRGDQIARNIGFNTANILCDNNLIDIAEGVYKAQVIFNNKKYDSVVYLGIPKTINTRSFSMIEAHILDFNQNIYDERIKIVFLKYLAPNLKFNNIDELITAIKNYIKLVLDKTN*

>UUR10_RS03335 Ureaplasma_urealyticum_serovar_10_str_ATCC_33699_NC_011374 transcription termination/antitermination protein NusG

MAYKIKDLDSKLLSDLKIDLNHTHQ*YIVTVVSGNEQKVIENIKDKLNGYGYGDKLSDLKIIKEKIKEVKIYEPSEAPRSMKNRANTK*ETIVVDGVTKYRCTKIKEGNKFNGYIFLKAEMTDQI*FLIRNTQMVTGLVGSSGKNVKPIPVPEDKILKLIADNDAKRALVSLDEQTNSQQNVVVVESHETEDLPNFEVDQQVKIVADTFFGEIARIAKIDQNKKVATVEFEFFGRINTLDLNFNDIQPYDEEAELEN*

>UUR10_RS00650 Ureaplasma_urealyticum_serovar_10_str_ATCC_33699_NC_011374 preprotein translocase subunit SecA

MNLISKISPQNRILNHARLIAEEVLKKEDEYTHFSDQELINKSDDIIEYLANNNPLDDRLVESLCIIREVIYRVHNKRAFKVQLIGAIIVYFGDFAEMMTGEGKTLTLVLVAYLNALYKKGVHMVTVNEYLVKVGAEFATPALNFLNMSVGQITANMNEYEKRNNYDCDITYTTNSELGFDYLRDNMVTNYNSKVQRGL*FAIVDEGDSVLIDEARTPLIISGEPQEEIGNYVKADRFVKTLYPQDFTLDPESQSVALTESGVEKAQKFFNTKNYYNFENSDIIHKVTNALRANFTFFNGREYIVKKDDEGEDVIALVDQSTGRIMEGRSYSAGLQQAIQAKEQIKIEPENLTVATITYQSLFRLYKKLAAVSGTAITEVEEFLNIYNMVVVTIPTNKPIRRIDHPDYVFDNKRTK*KYVIADVIRRHENGQPILIGTASVEDSEILHQLLERVNIPHEVLNAKNHAREAEIVARAGEYKAVTIATNMAGRGTDIKLSPESLEAGGLCVIGTERSDSRRIDNQLRGRAGRQGDIGESRFFISMEDTLFSRFATDNLAKADDKLSEDVISTKFFTRLLNNTQKKVESLNYDTRKNLIDYDHVLSNQRELIYKQRDKILVSSDNKDILYRMLDSVIDDIIYQSHNEPNEDIIDVKKLIDLATQNIFYDNYLNHDEYYGLDLDEIKTKLKNDCISFFEQKEQLMTPGIFNQILSEIMISNIDEE*TKHLDVTSKIREGVNLRAYEQKAPLNIYVEDSDKLFEKLKHDVA*KTVCSIGKINYVHQEYDKVNNEFIINDNEIIDNDNVIDFENTDHSLISEQEIEDSLVNIDELNDQNTKNENND*

>UUR10_RS00990 Ureaplasma_urealyticum_serovar_10_str_ATCC_33699_NC_011374 50S ribosomal protein L27

MNKLY*LTDLQLFASKKGVDSSKNGRDSNPKYLGAKLGDGQSTKAGQIIYRQRGNKIYPGLNVGQGKDHTLFAKTAGVVKYTKFMGDKTKVSVLPKEDNK*

>UUR10_RS01210 Ureaplasma_urealyticum_serovar_10_str_ATCC_33699_NC_011374 translation initiation factor IF-1

MADTEKLKMLGKIVEILQGGNFRVQLENGITIMSHVSGKMRVNKINILPGDTVDVELSPYDLTRGRITYRHRDS*

>UUR10_RS03445 Ureaplasma_urealyticum_serovar_10_str_ATCC_33699_NC_011374 membrane protein insertase YidC

MSSVDKQNLMNRMRISVSHFAGASNANSTKKERRKKILNILLKVFKVIVYTFFLGIGLYGCFQNMANH*TINSTVVGNGFELGFHVDPILGANDIRFDLIYSGTGP*YPMSDFSFDYGPFYALFVWPIAQILLHFMYATRD*PAGLNAILGLIIILLIIRVITMLISARATIQTERISEIQGKIAEINAKYKDAKDMQSRQKKQMETKELYQKHNVKPLAPFESMIITLPIFLIIYRVVTILRPLKFISIFYI*DLSATPISEIFSNFTTSG*PYIFFLLIIIPVQILSQKIPQLLAKKRNRSATTVGAKNKQQLKRVRMTQNIIAIVLAVVVAISASGIGLY*FFNAIFTILQSYIIHVIIMKRRSNSATRIESKLAKLGIS*

>UUR10_RS02850 Ureaplasma_urealyticum_serovar_10_str_ATCC_33699_NC_011374 acyl carrier protein

MVVNVKDIIIKVAKENKINLNMNNLDVELKSLGIDSLSAMSLIMKIEDKIGVQLVDEKLLKIKNLGDLIMAFEDALK*

>UUR10_RS01215 Ureaplasma_urealyticum_serovar_10_str_ATCC_33699_NC_011374 50S ribosomal protein L36

MKVRASVKAICKDCKIVKRSGVVRVICANPKHKQRQG*

>UUR10_RS02080 Ureaplasma_urealyticum_serovar_10_str_ATCC_33699_NC_011374 NAD(P)-binding domain-containing protein

MSKILIIGSGAFGSALTQVLVSNHHAVDVYGINQNELNDLQQNQKNTTYFQDQKLSQPINNTYLDIHLALKNHYDFIVIVIPSFAIKNFVDSIKTLDLSQAIVVNAAKGLNLETKSS*CDYIQQNLKIKALIGLVGPSFAIDVFLKKPTVVNLVGTDLDALIKTKQAFEND*FKCVLSKQFEVANYISCFKNALAIGCGIIYGLEKSHNSLVAFLTKGINEMQLILETIYQKKVNPLEYFFIGDTILTCTDQKSRNFSFGLLVAQQGVQTALENKQKTVEGLNNIKVIYEIIKTKQIDAPLFESLYEVINENLTPKSLFNKSFC*

>UUR10_RS00005 Ureaplasma_urealyticum_serovar_10_str_ATCC_33699_NC_011374 ATP-binding protein

MSNNYQNLYDSAIKKIPYDLISDQAYEILEKAKVHKVYDGVLYIIVASAFEKTIINGNFINIISKYLSEEFKKENIVNFQFIVENEKVLINSNFLVKETVIKNRFNFSDEIMRYNFNNLVISDFNRKAVKAIESLLSTNYENSSMCNPLFLFGKVGIGKTHIVAAAGNQFANSNPNLKIYYYEGQDFFRKFCSASAKGTSHVEEFKKEIASANLLIFEDIQNIQSRDSAAELFFNIFNDIKLNGGKIILTSDRTPNELNGFHDRIISRLASGLQCKISQPDKNEAIKIINN*FEFKKKYQITDEAKEYIAEGFHTDIRQMIGNLKQICF*ADNDLNENLVITKDFIIECSVENDIPSNIIVKQQLKPEQVIEIIAKELNLKVDLIKSTTRKNSIVWARDIVCYVLKNKLNLTLTEIGKLLSGREHTTISHSVNKVEKILADKNSQEALQINLIIDKF*

>UUR10_RS01015 Ureaplasma_urealyticum_serovar_10_str_ATCC_33699_NC_011374 serine/threonine-protein phosphatase

MNFGFISDIGSQRKHNDDCALVIQNEHQQTLLIVCDGLGGYKGGAAASHITLETIKDNFLATNFNEYDEQQIRK*YIKVIKLAQIEIDRAVLLDKDVYNMGTTVVASIIINDFVYTLNIGDSRAYLLSNNQSSQISRDHNLLQVLHERKVGPEVYEKHEKNLFSLTQFVGRTSNVVLSYDLFVTKLHHNEIIVLTSDGFHNYFELNDLYDKLIVTNQQTNNQILQQLINQAIDNGSNDNLSLAFLIF*

>UUR10_RS01085 Ureaplasma_urealyticum_serovar_10_str_ATCC_33699_NC_011374 50S ribosomal protein L35

MAKIRQKTKRAAAKRFSITKNGKLKRKHAYRSHLALGRSTKAKRHLRKDAIMSTSDTKRYTQCL*

>UUR10_RS03145 Ureaplasma_urealyticum_serovar_10_str_ATCC_33699_NC_011374 30S ribosomal protein S18

MAKVINNRNRKPRKKVCILSAKGIEHVDYKDVELLQRFINNNNKIASRRVTGASARMQRRIANAIKRARFVGLLPYVKE*

>UUR10_RS00705 Ureaplasma_urealyticum_serovar_10_str_ATCC_33699_NC_011374 F0F1 ATP synthase subunit gamma

MSLDAIKRKISSVQTTAKITNAMKLVATAKLKRQRDRLAAIKEYCHDYYDVIGLLLSVVNDIEFLKIPNAKNRTLYITINSTMGLAGSYNYNVNKLVSKIINEDDITFTIGKKGHDFMRLSNRLHQVNTYLNLNDNDLTFDMSLQIAREALELYSNGEVNKICIIYTKFINAITFEVNNIDVLPFDKTVLTKDNLAETIELAKDNIIFQPNKVELVKKILPTYIATVLYGSLIESKISENASRRNAMDAATKNAKALAEDYKLIYNTLRQGKITREITEIVAGSDD*

>UUR10_RS01600 Ureaplasma_urealyticum_serovar_10_str_ATCC_33699_NC_011374 30S ribosome-binding factor RbfA

MANEVRVARLESLIKDVINNALANEINDKIAKLARVTAVRLSNDLSVAKIFLDAHKRESMPKVLENVNKVSGLLRSKLAAE*TSYKVPELRFVIDETIDYANHIDELFKKIKQQEN*

>UUR10_RS03155 Ureaplasma_urealyticum_serovar_10_str_ATCC_33699_NC_011374 30S ribosomal protein S6

MAKYEIMLVVRGDLDQEQANKVANELKATLKNTEVKENNYEGVQQLAYEINKLKTAYRYVYNFETTDVSLINEFRRLAIINKNVLRHIIINLEKDYGYKATVNAKKVQRNEKRAEVYVRQKEEAERRAAERQAAYEAMKAEREAAGLPVKEFVKGANSKR*

>UUR10_RS01550 Ureaplasma_urealyticum_serovar_10_str_ATCC_33699_NC_011374 tRNA (adenosine(37)-N6)-threonylcarbamoyltransferase complex dimerization subunit type 1 TsaB

MNSLYQLFIDVTSKKCVLAIYKNFKILANIIVETNNNLTDIIVEHIIALLKAVHLKYQDLDAIYLDIGPGSFTGVRVGAIVAKTICTTHNQIKLFINDSLNIIANNKNNVFVHLDAKGNKSYTISIINNIQSDYRIITNEQLQIELKNTSLTIIDANQVDYHNLIYNLKFDNFKLTNILDFDLNYVKKPLS*

>UUR10_RS00055 Ureaplasma_urealyticum_serovar_10_str_ATCC_33699_NC_011374 50S ribosomal protein L7/L12

MSKLTIEQFIAAIKEMSMLELNDLVKAIETEFGVSAAAPVAVAAAPAAAEAPTEVTIKLVEAGANKVGVIKLIREITGLGLMEAKTAAETAGSVIKEDVKTEEANEIKKKFDELGAKVQLV*

>UUR10_RS00695 Ureaplasma_urealyticum_serovar_10_str_ATCC_33699_NC_011374 ATP synthase F1 subunit epsilon

MANLTKLKIVTPYAQNLEKDVYSVELKTSEGRIAVLPDHNPLMSIIENHVAYIRELPNAPRKPLLLLDGIVYVEEHQVRVFSDYFKFLDEIKIDEINSLLNKLKNDLANEEDDKKKLQLKSKIKLNESILIAYKDR*

>UUR10_RS01180 Ureaplasma_urealyticum_serovar_10_str_ATCC_33699_NC_011374 50S ribosomal protein L18

MKRINFSRAKQRALRAKRLHVKIRNLQLAANKPVLVITKTNAHI*AQLICYNKNITLASSSSVQLDLQNGNKDNARLVGADIAKKALAQGFKQVIFNKNGAKYHGRIKALADAAREAGLEF*

>UUR10_RS01220 Ureaplasma_urealyticum_serovar_10_str_ATCC_33699_NC_011374 30S ribosomal protein S13

MARILGVDIPNDKRVVISLTYIFGIGKSTSQKILKLANIDENIRVNDLADEQIAEIRRVALNFVKANGEKLQLEGDLRRTVAMDIKRLMEIGSYRGIRHRRGLPVRGQRTKTNARTRKGPRKTVANKKIETR*

>UUR10_RS01000 Ureaplasma_urealyticum_serovar_10_str_ATCC_33699_NC_011374 50S ribosomal protein L21

MFAIFQTGGKQYKVQQGEKIYVEKLDLEVGSKISFDQVIMVEGSVGTPFVKNAVVNATVLKQGKQKKINIIKFKSKKHHLKRQGHRQPYTQLVIDSISVK*

>UUR10_RS01160 Ureaplasma_urealyticum_serovar_10_str_ATCC_33699_NC_011374 50S ribosomal protein L5

MAFLKDLYKNKVAKDLQKEFAYSSVMQIPKIEKVVINAGIGNAVADKKHLEAAISELTLITGQRPVETKAKKSIATFKLRAGQSIGAKVTLRGDRM*AFIETLFNIALPRVRDFKGISNNSFDDQGNYTLGIKEQIIFPQVVYDDVKSVRGFDVTFVTTAKTAQEAKALLVGLGAPFQKVRGDK*

>UUR10_RS01710 Ureaplasma_urealyticum_serovar_10_str_ATCC_33699_NC_011374 (d)CMP kinase

MKKYINVAIDGPSGSGKSTAAKGLANKLGFLYINTGLMYRAYAYFLNENNLDINTNETACIEAIKNARFIFNGDDVKIDDQDVSDILRSNDVAMLASVVAANAKIRNLATNEQRKIASENNVVMDGRDIGSIVLVDADLKFYLNTSIQTRAKRRLAQNKDIEKLDYESIYNDIKERDYRDMTRDIAPLKKAIDAIEIFNDNMNLDQCVAHLYEIYLNKIKKS*

>UUR10_RS00815 Ureaplasma_urealyticum_serovar_10_str_ATCC_33699_NC_011374 NAD(+)/NADH kinase

MKDVKPVYFYDIYCFNPNKCTEDKGVLLLETKLKEYQKVTFLRSEQKPQIVFLLGGDGSFINFVNQQ*KQN*KIVGINYGQLGFYSSYDGINTINIDEIVDESMYANAFLIEVNINNENKFYCLNELSIFSNELASCDISINNTFYEKFRGSGLLFATPSGSTGKNKVAHGPIIFNNQPCFSMLEIFPVNHLKYSSLNAPVVFGKDYQISLTNIKFKRTLNLVVDGNNINFNNKIDFIEVKLIQASLQIHGLNNYKKYIERLRRSFIKEE*

>UUR10_RS03075 Ureaplasma_urealyticum_serovar_10_str_ATCC_33699_NC_011374 energy-coupling factor transporter transmembrane protein EcfT

MSANAYVFRRSPIHRLNPAIKFISFILLIAMIFLPLGFFAQMIIGVFILIIFFVAKLPKKTL*NVFKSVIMLFVILLLIN*MTYKDPIAIYNITDQAKVILGDKD*INGPINKNLSFSLIYNDISSTHVQNLVSNIWGGEIKNYISPEIIKKLIDKPDYNVAKFLSENNITVKKLASTFNALNQDVRLNNYYPIYGDAVLRSGKVEVPLSHLSYYMSTNL*KIEGVKYQGLILSGVGDQLGKAETALFYTRSPFALSPVAIQLAIYISIKIFLMITLSSILTATTSSIELTNGLEDLLSPFKILRLPVAEASMMISIALRFIPSLLDESKRILNAQASRGVDFNNGGMLQKLKSLISLVVPLFSIAFKKAEDLANAMEARSYNPRYARTRYRAFPLNLTDYVLFGILCILVGFLISLAVIKFYFTPFGAFEASALFAK*

>UUR10_RS00025 Ureaplasma_urealyticum_serovar_10_str_ATCC_33699_NC_011374 L-threonylcarbamoyladenylate synthase

MKIYRITNLNAIYDALVANKCVLIPTDTIIGLLAKNQDVIYEIKRRDRNKKIVRFVADYKLLGDLTVEQEQFLDLF*PGSVTVIKNGVSYRMPNSPYILKLIQKLGPLYCSSANISGEEPVKNHNEAIFKFGANSKLIYVEAQQQIGVPSTIVDIDK*EYVRRGANIEMVDMFIKELKYNNTKEKE*

>UUR10_RS01125 Ureaplasma_urealyticum_serovar_10_str_ATCC_33699_NC_011374 50S ribosomal protein L22

MTNKVIQRNIHISHRKASLVIDLVRNKPVHEAIRILSNTPKKFAPIVLKLLNSAISNVQHNSKDMDPSKLYIYKIVANQGPTMKRTLPRAKGSADQLFKRTTHLEIVLSDDVNEREKELAAIKAKKSKKPLVVEPVAKVETKKVAKPSKVETKPVEKDENVDPELLKREQQVLKVVEKTASQKEEETTETIMISTSPKNAQVLFDDLEKNVIFYKTTPVNKVLRVLVYVTSPTKKVVGEFDLESVEIGAISSI*RKYNKQSVISKKEYDAYYEGKDKAHALVSKKAYKYRNPKDLSEYNMTKGPSGFQYLK*

>UUR10_RS03095 Ureaplasma_urealyticum_serovar_10_str_ATCC_33699_NC_011374 50S ribosomal protein L11

VAPKKKEVTRIAKLNLIGGQAKPGPALASVGINMAEFTKSFNDKTKDQNGKVIPVIITAYKDKSFDYVVKTTPVTYLLKDAAKIKSGAKDPKKQVVATISKEQALEIARYKLVDMTAYDEEAALRMIAGSAKQMGIAIEGVSAYKEKKGN*

>UUR10_RS01135 Ureaplasma_urealyticum_serovar_10_str_ATCC_33699_NC_011374 50S ribosomal protein L16

MLQPKRTKFRKPHKVSYEGKAKGNKQVDFGEFGLMALEGA*IDARQIESARIAISKRLLKTGKM*IRIFPHMSLTKKPLEVRMGSGKGSPEK*VAVVKAGTVMFEIANVSEELMREALRAAGNKLPIKVKIVKKGEAN*

>UUR10_RS01745 Ureaplasma_urealyticum_serovar_10_str_ATCC_33699_NC_011374 RNA polymerase sigma factor

MSTKQNEPLFENLEDLKQKVKLSFTEEFSFSYALSEREAGIFESRNLKGADASNPEEILLNVVLDVSKRKRSRNEIKFNKLQNYFIHMNLRDEHFSEIVDVLENIGIRVPDYELVMQSKSKSTAKKKDEYGIDDTLEISTSKIGFSSTTTEKVDDGIKAYLGVLGESKMLRSDEETEYAKMVISNDPALIKIGKNQLYTSNMRLVTSIAKKYLNRGLDLEDLIQEGSSGLLKAIDKFDHEKGHKFSTYAT**IRQSITRAIADQARQIRIPVHMVETINKLTKAERSLIQELGRDPTAEEIAQAMNKASQAKNQKEQLITAQKVVEIKKLNVDPVSLDKQIGHDEESQFSDFISDDEIISPEKYTEKKALNDQINEMFEKVLNDNEQRVIKMRYGLLPFERPYTLEEVGEHLGVTRERARQIESKAIRKLKHPSKTAKLRSFIGESEN*

>UUR10_RS00400 Ureaplasma_urealyticum_serovar_10_str_ATCC_33699_NC_011374 HPr kinase/phosphorylase

MEIRGKLFVSQVVRKFNLNVVANSDYIDREISTTGITRVGFELAGEILFKEI*NIVYFGSKESNYFSKFSETIISKKLGKILDLNPPLIIFGKNFKHAGILLKLAERYKIPIVEVKYSFYELNFTINTYISQKLSHQSLVHGTLLSIYGIGVILMGESGVGKSELAIELVKKGHIFVGDDAILVNRIGGNLYGRAEDSTKDFIEIRGLGIMNFSRSFGIERMIESTKIEIVIELIKAAKHEKIKFERFGREIQHKEFLETKIAYYYIPVIEGRSISDIIETAITDYKLKTSGYNSAEEFILQIDKKGN*

>UUR10_RS00980 Ureaplasma_urealyticum_serovar_10_str_ATCC_33699_NC_011374 50S ribosomal protein L28

MARRDQLTGKGPLSGNTRSHAMNHSKRR*NVNLQKATIKTENGSQRVLVSAKTLKTLKKHNLLA*

>UUR10_RS02265 Ureaplasma_urealyticum_serovar_10_str_ATCC_33699_NC_011374 bifunctional oligoribonuclease/PAP phosphatase NrnA

MQKDLLNKLIEQTYGFSKISIFVHTNPDCDALGSAFALARILKLNTFGTRVKIVGINTLNPNDFKNFFTFDKNEVEDEFIEGSLAFIVDTANQERVLSQKHTLAKKTILVDHHVKTVSYTDLTYINDQSIATCEMLAYSLMHTNLNFDVKTLNYLLLGLTTDSNRLMYDKVSDITYEIMA*FFKNNVKHYQIYQQLYERNLDDILFDNELIKTIKTHKQIAYLNIDKS*NQKYNFTR*GDKVYLLSNIKNYPI*FVVYFDETTNTYKVSLRSNKYKVRLVANQFNGGGHDLAAGCSLANIDQLNDLLKALELLIKNQEVVD*

>UUR10_RS01540 Ureaplasma_urealyticum_serovar_10_str_ATCC_33699_NC_011374 30S ribosomal protein S20

MANIVSNEKTYRHTQKVRKENHAKMSKLRTIVKKTRSSNEQAQLNEAYKVIDTTASKGVIHKNKANRLKSRTAKAFKANLQVVA*

>UUR10_RS03235 Ureaplasma_urealyticum_serovar_10_str_ATCC_33699_NC_011374 50S ribosomal protein L19

MALFKINKGEIMNFVNSTQLKTDIPSFDSGDTIIVHNRIVEGKKTRIQKFEGVVLRRRGSGSSETVIVRKESNGVGVEQSFNIHSPLVEKIEVIKYGKVRRAYISYMRNRSGKSARIKELNKQ*

>UUR10_RS01860 Ureaplasma_urealyticum_serovar_10_str_ATCC_33699_NC_011374 Holliday junction resolvase RuvX

MRKLALDLGTKSCGFAISDLLGIIASGLDNFIYEENDFIAVLAKIDEIMINYHHEIDTIVLGYPTNVYDGSKNKRTYLIESFYTLLKQHFLNHEKIKIVYEDERFSTKIATQRLKNSCVKAAKIKKVKDKMSAVVILESYLSKNHFN*

>UUR10_RS02555 Ureaplasma_urealyticum_serovar_10_str_ATCC_33699_NC_011374 nicotinate-nucleotide adenylyltransferase

MKIILFCGAFDMVHNAHIAMAKYAIDLIKADKLIFLPSNFKFFKPINKDDNLEYEKTKLTHGHHRLAMLKIATKNLVNTEVSDYELNQVNKSYTINTIDHFKKLYGAEHEYYFIIGSDNLERFKQ*KD*ERILKEVKIICFKRSGVCLKKTCFQNQCNCENFNFFEHQIILVNDFNYNISSTEIKKQHNLASGIDPAVLDYINEHGLYAL*LLEKHLISYDNFNNLEKKIARINHCRRVAQMCVDLMNVYDKKLIDQAYCAGIYHDILKCLDEQESIAYFNEHKSELNIGDDFIS*RILHSYLGAHLLQTQYGFKNQLILNAIRRHTRPFDFIKDYSELTTLDKILYCADKLEPNRREEIDQINIDYYRKLVFEDLDKAFIEVYKYQQRQRK*

>UUR10_RS00395 Ureaplasma_urealyticum_serovar_10_str_ATCC_33699_NC_011374 prolipoprotein diacylglyceryl transferase

MQLEIINPESTLINDVVAHRIAFSIGSNFNIY*YGIIFVCGFLLAILTYSLRLKFHYKVPYDPGFYYIFLAIPMTIIGARL*SLAIGDAKDFFDFRNGGLAIQGGVIAGVLSAAIYFPLILRMPKYHVRDLDADGNVIIRQPSM*IYADAIIPTILIGQALGRWGNFINGEIFGAESTVNDLQ*LKKAMPAVFEGMKHYFIEGDKTLFTIYQPLFLYESFFNVIVFVFIYFGLSYIKQLKIGFVSMSYFFFYGVIRFSTESARAPQFSFAGTYVINSLLLIFGVLGALYVQFIAPILRKRFLLDAIIELFYKKKQQAHKFGQLRNPEEFLYYCHK*

>UUR10_RS00840 Ureaplasma_urealyticum_serovar_10_str_ATCC_33699_NC_011374 2,3-bisphosphoglycerate-independent phosphoglycerate mutase

MSLNKKLALIIIDGLGIGKKDDTNAVYLANPKTLNYLIKNYPTLEISAAQQPIGLLENQAGNSEIGHLTIGAGRIILNDNANINSYTKRLDYESLVLNDINNEIVHVVGMYSNGLVHSNYEHIH*IIKELVKNNNQVVLHLISDGRDDYPYGFAQFIEQINALKTQYNVIIKSLSGRYFAMDRDQR*ERTQKAFNTMFIKQDKICEQSLLEVAQSIANHYESDEFVEPIVFNNDEKYNLKPYQKVILTNYRSDRMRQLAHLLKPNRKFNYHNPFLIKDIHLITLVPFPDVDAITLFEKQNLNNTLGDVLNDHHIKQARVAETEKYGHISFFFDGGINKHYASKTQYLIPSQKVATYDLCPQMSASLITKTIIDHYFDHDVFIVNYANPDMVGHSGNMKQTIQAILSVDSEIQKLYDFFKKNNGVLMITGDHGNAETMIDANGQIITSHSINDV*FIITDNNIVFDQTQKFSLANIAPTILEYLNIKKPIEMAASSMIKKIHK*

>UUR10_RS01110 Ureaplasma_urealyticum_serovar_10_str_ATCC_33699_NC_011374 50S ribosomal protein L23

MELTRVILHPYTTEKTYSIRNKSEHETLTFIVDKNANKYQIREAFIAIFGLKPLKIRTTNRGPAKIRTSTARPGYTKAKKIAYIVMPIGVKVAVSKEEVEAANAK*

>UUR10_RS02255 Ureaplasma_urealyticum_serovar_10_str_ATCC_33699_NC_011374 DNA polymerase III subunit alpha

MFINLNVHSYYSLLNSALSIDDLIQHALDNNQPYVCLTDLNNMYGCIEFYDKAKAHNLIPIIGLEFEYQNTTLVAYAKNYNGYLKLIK*SS*IMTNTTFIIQEDFDDLIIVCKKGGLVFENPNFYQAQNQNASNAIALQSVFYAQENDKTVFLAMLAIKNDLKLDDFIDCHEFDKNYFLNDHEAQSLFSTIALDNLNKVLNELQVEIHDLPINIPVYDKNNLTVSSEILKQLCISGLKQRLNAHDGQVKKVYAKRLKYELDVISEKQFDDYFLIVYDFINYAKSNGIIVGPGRGSAAGSLVAYCLYITDIDPIKHNLIFERFLNPTRKSMPDIDTDIMDEKRDQVIEYLFEKYGNDHVAYIVTFQRLKAKMALRDVGRILGIDLKVIDKICKNIKTDYDEDIDLAIKKSATLKEMYVLHKELFEISKKLIHAPRQIGTHAAGIILSNSSITNIIPIQLGINDRPLSQYSMEYLERFGLIKMDLLGLKNLTIIDNVLKMIYKTQNKKIDLFNIDYNDKFVFQDLAKAKTNGIFQLESPGMKKVLLKVKPQNIEDISIVSALFRPGPQQNIKTFVERRFKREEFSY*NEQTKKILEPTYGIIIYQEQVIELVKTIANFDIATSDNFRRAISKKDEKILMQLKDDFINGALANNYKQPLVNQIFEYIFSFAHYGFNHSHSLAYSYISY*LAYLKHYYPLEFLSVLLSHTSASKEKLLSYLDETKDFNISIKGPDIQHFSNDFVIDNHKQIIRFGFKTIKGFGDELLKKIKLALENAELSDYISYIDALKKGNISLKNIEILIRIGAFDSFEINRLFLLNNLEEIFEKTGLNGHFFDLNLVGLDYANDMSINERFQEDEIQYLGINLSSLNYTNYTNEIDYSNLKYEIESFNEINTNYEVNIVAQVLNIVQSKTKKGNDIFYLDVLVENKKEKLTIFQNSKHLVDEIDINGIYVFGVKLLNHFNFIVSVKQRV*

>UUR10_RS00415 Ureaplasma_urealyticum_serovar_10_str_ATCC_33699_NC_011374 DNA polymerase III subunit beta

MEVFVSIKKLIEAMKFSTTIANTNNANALLLGVLIEVNENKITFKTTNNQVSGYKEISDGFEYFSSGKILVTAKILLGLISKLKDKSVLLKQVDTNILLIKTENFETQINTMNIESFPSLNFSLEDYVKISLPHQIMQEINAKVLPNVLNSQGIEKIQPISGVLIDTETLDNQLIAIGTDKIKASCLTKPYLGEKFKFIISYSTMKLIMEVLRNVEYSNNQIVDFYVRNKSLVFKVNDAILQTRMIDGVYPNVYSIFNETNEEKNYVFDRRLLIEIIERGMNIVMQEQNPKISIKIENNEAEISLTTFEIGNMKEKMPIINLSNANVEFIVNPSLLAHVLKNFENNDVNFKVKDEILRPIIFIDAKDLGFKQILSRIKN*

>UUR10_RS01145 Ureaplasma_urealyticum_serovar_10_str_ATCC_33699_NC_011374 30S ribosomal protein S17

MERSRRKVLEGLVVSDKMQKTVVVSVETKSKHPIYRKLVISHKKYHAHNDNDDAKVGDLVEITETRPLSATKN*RVSKILERAR*

>UUR10_RS01090 Ureaplasma_urealyticum_serovar_10_str_ATCC_33699_NC_011374 50S ribosomal protein L20

MRVKGGSVTRQRRKR*LEKAEGS*GTRNTSYRIARQTVIRAAEYAYRDRRNKKRDFRKL*ISRINAAVRELGYTYSQFMNALVKANVVTKDGQGLNRKMLSELAINNPEAFNQLVNKVMK*

>UUR10_RS02750 Ureaplasma_urealyticum_serovar_10_str_ATCC_33699_NC_011374 30S ribosomal protein S4

MSRYTGSIYKKSRRLGFSLLENNKEFNSGKKRTYGPGQHGNKKVKLSNYGQQLVEKQKLMFLYGLNDRQFRRLYRVALGRPGVLTLNLLQVLESRLDSLVYRAGFAPTRRAARQLVNHSHVLVNGKKVNIPSALVEVGSTIALKEKSLEMPLIKNTLNKPADFIELVDKDKKVAKLSRLPERSELPADVNEAYVVE*YNRLM*

>UUR10_RS01175 Ureaplasma_urealyticum_serovar_10_str_ATCC_33699_NC_011374 50S ribosomal protein L6

MSRIGNRKLTIPANVNVSVESGKVHIVSQTAKLSVDFPVNLISVDVVDNTIKVSRANDEKQTKMFHGTVNANIANALVGVTTG*KKELEVKGVGFRAKVEGSKLNLGLGFSHPLLIQIPTGLKIETPSATEISISGSDKATVGAFAAVVRAYRKPEPYKGKGVMYKGERIVRKAGKTADKKK*

>UUR10_RS01525 Ureaplasma_urealyticum_serovar_10_str_ATCC_33699_NC_011374 ATP-binding protein

MNNNEIEDELDLENFNYQKALEVPNLKAINLTEDEFNLHF*DIVGVYRSYLNNLKEPNDSGYIYELNRNEYNHLCLVVIKKESKVDKVKKNYILNTIKNMDYDISLTDDSQIFSKKSEILDNDLLVERNKLINFFLEEARKNKKQSANKEDNITTNDQQLKSAFIYGDFGVGKSIITQAYTNTISLKYNLKIAYITLNELFKNVIQFFNYKDISDSVVNELINELSNIDVLVIDDFSSGNLNY*SISTILMPIIENRLKSMKQTIFISNFSIEQLNNSTKNIANIEEQKAKLRLFNRIECLTYGNVFKIKGPSIFKVTNNL*

>UUR10_RS00720 Ureaplasma_urealyticum_serovar_10_str_ATCC_33699_NC_011374 hypothetical protein

MQSNTKLVKYSKIIANRLKAYSNQTRFIEIKTAFELNNEQKQRIKKTIINRFGDERPIKFIVDPSLIGGVSLKINLEIIDSSLKTKLNQIINIKEKEGA*

>UUR10_RS00620 Ureaplasma_urealyticum_serovar_10_str_ATCC_33699_NC_011374 dihydrofolate reductase

MLKLI*CQTLNGGISKNNKLP*YVKEELEHFYKTTKNHKIVMGKSTFDSLEQKPLSNRTNIIFSSIMQTPEDQSYFVTNDFQQLLNDAKKEDIFIIGGKELFDIFLNHADALIVSVLNDYYDCNLYMKVDYNNFNLDKKDVYDNFVVNYYSSKKDK*

>UUR10_RS00050 Ureaplasma_urealyticum_serovar_10_str_ATCC_33699_NC_011374 50S ribosomal protein L10

MANVRPSVVFKQQEVDHMADILKNSKSFIVFEYHGLTAANILALRNVLHSSNSKLFVLKNNITARAFEKAGVTGFEDRLTGPNAIAVAMDDEIAAIKAVNDVAKEFDFVKIKGAYLENKFADTHKIDQLAAIPGREGLYSMLLSCFTAPLRNVLYGLKAVAEQKGE*

>UUR10_RS02455 Ureaplasma_urealyticum_serovar_10_str_ATCC_33699_NC_011374 phenylalanine--tRNA ligase subunit beta

MILSLNLLHKISPKLKKISLNELCTALMDLGCEVETINTIKPSTNLVFAKVLEKTKHPNANHLNLVKVKANQEVYEIVCGADNFNVNN*VVLAKINAELANGLKITPRELRGYVSNGMLCAYSEINPEVAHFLGQTDLDGILVLHDSYDHYKTPNQIFNLDDVILDLSIPSNRNDLNGYF*MAKELCAYFDLEYVIDATINHRSHKEIVNVRILSDDVNSYGMIEVKNIQNYTLK*NTKSVLVNNQIKIVNNFADNMNFLTLLTANPLHAFDAHKISGQIIVKNAEEDSILLGLDQKEYAIKKGDLIIVDDQKILALAGIIGSNDSKIDNNTTTAYIECANFNPLLIANTARRLKINTTAAMRFSKPLTNYVTKATLKKLLAHFKLDAKLICYFKHLVHNVIKNKIDQVSDFVGTKINLDTAHTFLKRLGYKINKTNLITPSHRYDVLNEFDVYEDIMKKFSIQQIKPQPINFDILSFKNNIAYDFEKKVSDFLVDQGLFECKTYNLKSQTQAYEIDFFNFQQAYEINNPISNIRSHLKLNNLNSLLEVLEYNQNQKNELENIFEISKINPINSNQQTILSIVLCKPLINAKLNDSIVVNNFVTTKALLHVLLTKLNISYVYDTNHIVNELYENNQLALVNENKQVFGFIGQLKNQIKKTYGLNNDIFVINLNLTSYLNQEQAITKVIKPSVYHDIVRDVSVKLASNVDLNDVMDNIEKIKNIRKVEISDLYVKDDEIIYTFKYYINDYSSNLSSEQIAVIEQEVNNYLKQF*

>UUR10_RS03360 Ureaplasma_urealyticum_serovar_10_str_ATCC_33699_NC_011374 transketolase

MNRYVNAMRSLALQAINKANQGHSGMSISAAPIIYTLYKGLMTISKSHPK*FNRDRLVLSAGHGSMALYPVFYFSSLLTLDDIKNFRNDNHLTPGHPEVLSNNYIDASTGPLGQGVANAVGMAITESYLRAEFASLKGVVDHYTYCIVGDGDLQEGISYEAMSIAGKLKLSKLIILHDSNDYQLDSAVSDVNIEDLKMRVESMG*NYLKTDNNPENIFKAIAEAK*KKNVKPTFIEVKTIIGEGTSFENSNEAHAAAISKEELEKFGKRFHTKTNNFEFHQEIFDHFFFNVVARGESAYNQ*QQLVDQYMQTNPEQMQRLLNYINGNYEDLNKMLDENKIVNLSDSTRSYLKQYFAQLKDLKSALVLSADLAKSTFTKIGENAFNDDYKNPYIKFGIREFAMAGAMNGISLHQGAKAIGGTFLAFSDYMKPAIRLTAISNLANLFIFSHDSYAVGGDGPTHQPVDQLPMLRAIPNVEVIRPADHYEVKHALSYSFKQKQKPICLVTSRQAIKQINEQKPQDFTKGAYIINSPFSFSENPDYTIIASGSEVSLANDAAKEIFEKHQLKVKVISAFNLNLFLQQKPEVIKNLVSSKNGLLAIEASSEML**KLSVYTNKFMQIAANQFGRSADGNKLMHEFGFSVENIINQLLNKK*

**Supplementary Sequence file 4 Anti-target protein sequences in *Homo sapiens* (refer to Fatoba et al., 2021)**

>CAA41558.1 P-glycoprotein [Homo sapiens]

MDLEGDRNGGAKKKNFFKLNNKR

>CAA29547.1 P-glycoprotein (431 AA), partial [Homo sapiens]

LTLLLLAVVPIIAVSGIVEMKLLAGNAKRDKKELEAAGKIATEAIENIRTVVSLTQERKFESMYVEKLYGPYRNSVQKAHIYGITFSISQAFMYFSYAGCFRFGAYLIVNGHMRFRDVILVFSAIVFGAVALGHASSFAPDYAKAKLSAAHLFMLFERQPLIDSYSEEGLKPDKFEGNITFNEVVFNYPTRANVPVLQGLSLEVKKGQTLALVGSSGCGKSTVVQLLERFYDPLAGTVFVDFGFQLLDGQEAKKLNVQWLRAQLGIVSQEPILFDCSIAENIAYGDNSRVVSQDEIVSAAKAANIHPFIETLPHKYETRVGDKGTQLSGGQKQRIAIARALIRQPQILLLDEATSALDTESEKVVQEALDKAREGRTCIVIAHRLSTIQNADLIVVFQNGRVKEHGTHQQLLAQKGIYFSMVSVQAGTQNL

>CAA41416.1 70kDa peroxisomal membrane protein [Homo sapiens]

MAAFSKYLTARNSSLAGAAFLLLCLLHKRRRALGLHGKKSGKPPLQNNEKEGKKERAVVDKVFFSRLIQILKIMVPRTFCKETGYLVLIAVMLVSRTYCDVWMIQNGTLIESGIIGRSRKDFKRYLLNFIAAMPLISLVNNFLKYGLNELKLCFRVRLTKYLYEEYLQAFTYYKKGNLDNRIANPDQLLTQDVEKFCNSVVDLYSNLSKPFLDIVLYIFKLTSAIGAQGPASMMAYLVVSGLFLTRLRRPIGKMTITEQKYEGEYRYVNSRLITNSEEIAFYNGNKREKQTVHSVFRKLVEHLHNFILFRFSMGFIDSIIAKYLATVVGYLVVSRPFLDLSHPRHLKSTHSELLEDYYQSGRMLLRMSQALGRIVLAGREMTRLAGFTARITELMQVLKDLNHGKYERTMVSQQEKGIEGVQVIPLIPGAGEIIIADNIIKFDHVPLATPNGDVLIRDLNFEVRSGANVLICGPNGCGKSSLFRVLGELWPLFGGRLTKPERRKLFYVPQRPYMTLGTLRDQVIYPDGREDQKRKGISDLVQKEYLDNVQLGHILEREGGWDSVQDWMDVLSGGEKQRMAMARLFYHKPQFAILDECTSAVSVDVEGYIYSHCRKVGITLFTVSHRKSLWKHHEYYLHMDGRGNYEFKQITEDTVEFGS

>sp|P15382.1|KCNE1_HUMAN RecName: Full=Potassium voltage-gated channel subfamily E member 1; AltName: Full=Delayed rectifier potassium channel subunit IsK; AltName: Full=IKs producing slow voltage-gated potassium channel subunit beta Mink; AltName: Full=Minimal potassium channel

MILSNTTAVTPFLTKLWQETVQQGGNMSGLARRSPRSSDGKLEALYVLMVLGFFGFFTLGIMLSYIRSKKLEHSNDPFNVYIESDAWQEKDKAYVQARVLESYRSCYVVENHLAIEQPNTHLPETKPSP

>sp|P20813.1|CP2B6_HUMAN RecName: Full=Cytochrome P450 2B6; AltName: Full=1,4-cineole 2-exo-monooxygenase; AltName: Full=CYPIIB6; AltName: Full=Cytochrome P450 IIB1

MELSVLLFLALLTGLLLLLVQRHPNTHDRLPPGPRPLPLLGNLLQMDRRGLLKSFLRFREKYGDVFTVHLGPRPVVMLCGVEAIREALVDKAEAFSGRGKIAMVDPFFRGYGVIFANGNRWKVLRRFSVTTMRDFGMGKRSVEERIQEEAQCLIEELRKSKGALMDPTFLFQSITANIICSIVFGKRFHYQDQEFLKMLNLFYQTFSLISSVFGQLFELFSGFLKYFPGAHRQVYKNLQEINAYIGHSVEKHRETLDPSAPKDLIDTYLLHMEKEKSNAHSEFSHQNLNLNTLSLFFAGTETTSTTLRYGFLLMLKYPHVAERVYREIEQVIGPHRPPELHDRAKMPYTEAVIYEIQRFSDLLPMGVPHIVTQHTSFRGYIIPKDTEVFLILSTALHDPHYFEKPDAFNPDHFLDANGALKKTEAFIPFSLGKRICLGEGIARAELFLFFTTILQNFSMASPVAPEDIDLTPQECGVGKIPPTYQIRFLPR

>sp|P09211.2|GSTP1_HUMAN RecName: Full=Glutathione S-transferase P; AltName: Full=GST class-pi; AltName: Full=GSTP1-1

MPPYTVVYFPVRGRCAALRMLLADQGQSWKEEVVTVETWQEGSLKASCLYGQLPKFQDGDLTLYQSNTILRHLGRTLGLYGKDQQEAALVDMVNDGVEDLRCKYISLIYTNYEAGKDDYVKALPGQLKPFETLLSQNQGGKTFIVGDQISFADYNLLDLLLIHEVLAPGCLDAFPLLSAYVGRLSARPKLKAFLASPEYVNLPINGNGKQ

>AAA35496.1 alpha-1A-adrenergic receptor [Homo sapiens]

MAAALRSVMMAGYLSEWRTPTYRSTEMVQRLRMEAVQHSTSTAAVGGLVVSAQGVGVGVFLAAFILMAVAGNLLVILSVACNRHLQTVTNYFIVNLAVADLLLSATVLPFSATMEVLGFWAFGRAFCDVWAAVDVLCCTASILSLCTISVDRYVGVRHSLKYPAIMTERKAAAILALLWVVALVVSVGPLLGWKEPVPPDERFCGITEEAGYAVFSSVCSFYLPMAVIVVMYCRVYVVARSTTRSLEAGVKRERGKASEVVLRIHCRGAATGADGAHGMRSAKGHTFRSSLSVRLLKFSREKKAAKTLAIVVGVFVLCWFPFFFVLPLGSLFPQLKPSEGVFKVIFWLGYFNSCVNPLIYPCSSREFKRAFLRLLRCQCRRRRRRRPLWRVYGHHWRASTSGLRQDCAPSSGDAPPGAPLALTALPDPDPEPPGTPEMQAPVASRRSHPAPSASGGCWGRSGDPRPSCAPKSPACRTRSPPGARSAQRQRAPSAQRWRLCP

>AAA59575.1 P-glycoprotein [Homo sapiens]

MDLEGDRNGGAKKKNFFKLNNKSEKDKKEKKPTVSVFSMFRYSNWLDKLYMVVGTLAAIIHGAGLPLMMLVFGEMTDIFANAGNLEDLMSNITNRSDINDTGFFMNLEEDMTRYAYYYSGIGAGVLVAAYIQVSFWCLAAGRQIHKIRKQFFHAIMRQEIGWFDVHDVGELNTRLTDDVSKINEVIGDKIGMFFQSMATFFTGFIVGFTRGWKLTLVILAISPVLGLSAAVWAKILSSFTDKELLAYAKAGAVAEEVLAAIRTVIAFGGQKKELERYNKNLEEAKRIGIKKAITANISIGAAFLLIYASYALAFWYGTTLVLSGEYSIGQVLTVFFSVLIGAFSVGQASPSIEAFANARGAAYEIFKIIDNKPSIDSYSKSGHKPDNIKGNLEFRNVHFSYPSRKEVKILKGLNLKVQSGQTVALVGNSGCGKSTTVQLMQRLYDPTEGMVSVDGQDIRTINVRFLREIIGVVSQEPVLFATTIAENIRYGRENVTMDEIEKAVKEANAYDFIMKLPHKFDTLVGERGAQLSGGQKQRIAIARALVRNPKILLLDEATSALDTESEAVVQVALDKARKGRTTIVIAHRLSTVRNADVIAGFDDGVIVEKGNHDELMKEKGIYFKLVTMQTAGNEVELENAADESKSEIDALEMSSNDSRSSLIRKRSTRRSVRGSQAQDRKLSTKEALDESIPPVSFWRIMKLNLTEWPYFVVGVFCAIINGGLQPAFAIIFSKIIGVFTRIDDPETKRQNSNLFSLLFLALGIISFITFFLQGFTFGKAGEILTKRLRYMVFRSMLRQDVSWFDDPKNTTGALTTRLANDAAQVKGAIGSRLAVITQNIANLGTGIIISFIYGWQLTLLLLAIVPIIAIAGVVEMKMLSGQALKDKKELEGAGKIATEAIENFRTVVSLTQEQKFEHMYAQSLQVPYRNSLRKAHIFGITFSFTQAMMYFSYAGCFRFGAYLVAHKLMSFEDVLLVFSAVVFGAMAVGQVSSFAPDYAKAKISAAHIIMIIEKTPLIDSYSTEGLMPNTLEGNVTFGEVVFNYPTRPDIPVLQGLSLEVKKGQTLALVGSSGCGKSTVVQLLERFYDPLAGKVLLDGKEIKRLNVQWLRAHLGIVSQEPILFDCSIAENIAYGDNSRVVSQEEIVRAAKEANIHAFIESLPNKYSTKVGDKGTQLSGGQKQRIAIARALVRQPHILLLDEATSALDTESEKVVQEALDKAREGRTCIVIAHRLSTIQNADLIVVFQNGRVKEHGTHQQLLAQKGIYFSMVSVQAGTKRQ

>AAA36207.1 P-glycoprotein [Homo sapiens]

MDLEAAKNGTAWRPTSAEGDFELGISSKQKRKKTKTVKMIGVLTLFRYSDWQDKLFMSLGTIMAIAHGSGLPLMMIVFGEMTDKFVDTAGNFSFPVNFSLSLLNPGKILEEEMTRYAYYYSGLGAGVLVAAYIQVSFWTLAAGRQIRKIRQKFFHAILRQEIGWFDINDTTELNTRLTDDISKISEGIGDKVGMFFQAVATFFAGFIVGFIRGWKLTLVIMAISPILGLSAAVWAKILSAFSDKELAAYAKAGAVAEEALGAIRTVIAFGGQNKELERYQKHLENAKEIGIKKAISANISMGIAFLLIYASYALAFWYGSTLVISKEYTIGNAMTVFFSILIGAFSVGQAAPCIDAFANARGAAYVIFDIIDNNPKIDSFSERGHKPDSIKGNLEFNDVHFSYPSRANVKILKGLNLKVQSGQTVALVGSSGCGKSTTVQLIQRLYDPDEGTINIDGQDIRNFNVNYLREIIGVVSQEPVLFSTTIAENICYGRGNVTMDEIKKAVKEANAYEFIMKLPQKFDTLVGERGAQLSGGQKQRIAIARALVRNPKILLLDEATSALDTESEAEVQAALDKAREGRTTIVIAHRLSTVRNADVIAGFEDGVIVEQGSHSELMKKEGVYFKLVNMQTSGSQIQSEEFELNDEKAATRMAPNGWKSRLFRHSTQKNLKNSQMCQKSLDVETDGLEANVPPVSFLKVLKLNKTEWPYFVVGTVCAIANGGLQPAFSVIFSEIIAIFGPGDDAVKQQKCNIFSLIFLFLGIISFFTFFLQGFTFGKAGEILTRRLRSMAFKAMLRQDMSWFDDHKNSTGALSTRLATDAAQVQGATGTRLALIAQNIANLGTGIIISFIYGWQLTLLLLAVVPIIAVSGIVEMKLLAGNAKRDKKELEAAGKIATEAIENIRTVVSLTQERKFESMYVEKLYGPYRNSVQKAHIYGITFSISQAFMYFSYAGCFRFGAYLIVNGHMRFRDVILVFSAIVFGAVALGHASSFAPDYAKAKLSAAHLFMLFERQPLIDSYSEEGLKPDKFEGNITFNEVVFNYPTRANVPVLQGLSLEVKKGQTLALVGSSGCGKSTVVQLLERFYDPLAGTVLLDGQEAKKLNVQWLRAQLGIVSQEPILFDCSIAENIAYGDNSRVVSQDEIVSAAKAANIHPFIETLPHKYETRVGDKGTQLSGGQKQRIAIARALIRQPQILLLDEATSALDTESEKVVQEALDKAREGRTCIVIAHRLSTIQNADLIVVFQNGRVKEHGTHQQLLAQKGIYFSMVSVQAGTQNL

>AAA59576.1 P glycoprotein, partial [Homo sapiens]

MDLEGDRNGGAKKKNFFKLNNKSEKDKKEKKPTVSVFSMFRYSNWLDKLYMVVGTLAAIIHGAGLPLMMLVFGEMTDIFANAGNLEDLMSNITNRSDINDTGFFMNLEEDMTRYAYYYSGIGAGVLVAAYIQVSFWCLAAGRQIHKIRKQFFHAIMRQEIGWFDVHDVGELNTRLTDDVSKINEGIGDKIGMFFQSMATFFTGFIVGFTRGWKLTLVILAISPVLGLSAAVWAKILSSFTDKELLAYAKAGAVAEEVLAAIRTVIAFGGQKKELERYNKNLEEAKRIGIKKAITANISIGAAFLLIYASYALAFWYGTTLVLSGEYSIGQVLTVFFSVLIGAFSVGQASPSIEAFANARGAAYEIFKIIDNKPSIDSYSKSGHKPDNIKGNLEFRNVHFSYPSRKEVKILKGLNLKVQSGQTVALVGNSGCGKSTTVQLMQRLYDPTEGMVSVDGQDIRTINVRFLREIIGVVSQEPVLFATTIAENIRYGRENVTMDEIEKAVKEANAYDFIMKLPHKFDTLVGERGAQLSGGQKQRIAIARALVRNPKILLLDEATSALDTESEAVVQVALDKARKGRTTIVIAHRLSTVRNADVIAGFDDGVIVEKGNHDELMKEKGIYFKLVTMQTAGNEVELENAADESKSEIDALEMSSNDSRSSLIRKRSTRRSVRGSQAQDRKLSTKEALDESIPPVSFWRIMKLNLTEWPYFVVGVFCAIINGGLQPAFAIIFSKIIGVFTRIDDPETKRQNSNLFSLLFLALGIISFITFFLQGFTFGKAGEILTKRLRYMVFRSMLRQDVSWFDDPKNTTGALTTRLANDAAQVKGAIGSRLAVITQNIANLGTGIIISFIYGWQLTLLLLAIVPIIAIAGVVEMKMLSGQALKDKKELEGAGKIATEAIENFRTVVSLTQEQKFEHMYAQSLQVPYRNSLRKAHIFGITFSFTQAMMYFSYAGCFRFGAYLVAHKLMSFEDVLLVFSAVVFGAMAVGQVSSFAPDYAKAKISAAHIIMIIEKTPLIDSYSTEGLMPNTLEGNVTFGEVVFNYPTRPDIPVLQGLSLEVKKGQTLALVGSSGCGKSTVVQLLERFYDPLAGKVLLDGKEIKRLNVQWLRAHLGIVSQEPILFDCSIAENIAYGDNSRVVSQEEIVRAAKEANIHAFIESLPNKYSTKVGDKGTQLSGGQKQRIAIARALVRQPHILLLDEATSALDTESEKVVQEALDKAREGRTCIVIAHRLSTIQNADLIVVFQNGRVKEHGTHQQLLAQKGIYFSMVSVQAGTKRQ

>AAB26819.1 D2 dopamine receptor [Homo sapiens]

MDPLNLSWYDDDLERQNWSRPFNGSDGKADRPHYNYYATRLTLLIAVIVFGNVLVCMAVSREKALQTTTNYLIVSLAVADLLVATLVMPWVVYLEVVGEWKFSRIHCDIFVTLDVMMCTASILNLCAISIDRYTAVAMPMLYNTRYSSKRRVTVMISIVWVLSFTISCPLLFGLNNADQNECIIANPAFVVYSSIVSFYVPFIVTLLVYIKIYIVLRRRRKRVNTKRSSRAFRAHLRAPLKGNCTHPEDMKLCTVIMKSNGSFPVNRRRVEAARRAQELEMEMLSSTSPPERTRYSPIPPSHHQLTLPDPSHHGLHSTPDSPAKPEKNGHAKDHPKIAKIFEIQTMPNGKTRTSLKTMSRRKLSQQKEKKATQMLAIVLGVFIICWLPFFITHILNIHCDCNIPPVLYSAFTWLGYVNSAVNPIIYTTFNIEFRKAFLKILHC

>AAB31164.1 alpha adrenergic receptor subtype alpha 1b [human, heart, Peptide, 516 aa]

MNPDLDTGHNTSAPAHWGELKNANFTGPNQTSSNSTLPQLDITRAISVGLVLGAFILFAIVGNILVILSVACNRHLRTPTNYFIVNLAMADLLLSFTVLPFSAALEVLGYWVLGRIFCDIWAAVDVLCCTASILSLCAISIDRYIGVRYSLQYPTLVTRRKAILALLSVWVLSTVISIGPLLGWKEPAPNDDKECGVTEEPFYALFSSLGSFYIPLAVILVMYCRVYIVAKRTTKNLEAGVMKEMSNSKELTLRIHSKNFHEDTLSSTKAKGHNPRSSIAVKLFKFSREKKAAKTLGIVVGMFILCWLPFFIALPLGSLFSTLKPPDAVFKVVFWLGYFNSCLNPIIYPCSSKEFKRAFVRILGCQCRGRRRRRRRRRLGCAYTYRPWTRGGSLERSQSRKDSLDDSGSCLSGSQRTLPSASPSPGYLGRGAPPPVELCAFPEWKAPGALLSLPAPEPPGRRGRHDSGPLFTFKLLTEPESPGTDGGASNGGCEPRHVANGQPGFKSNMPLAPGQF

>AAB31165.1 alpha adrenergic receptor subtype alpha 1c [human, heart, Peptide, 466 aa]

MVFLSGNASDSSNCTQPPAPVNISKAILLGVILGGLILFGVLGNILVILSVACHRHLHSVTHYYIVNLAVADLLLTSTVLPFSAIFEVLGYWAFGRVFCNIWAAVDVLCCTASIMGLCIISIDRYIGVSYPLRYPTIVTQRRGLMALLCVWALSLVISIGPLFGWRQPAPEDETICQINEEPGYVLFSALGSFYLPLAIILVMYCRVYVVAKRESRGLKSGLKTDKSDSEQVTLRIHRKNAPAGGSGMASAKTKTHFSVRLLKFSREKKAAKTLGIVVGCFVLCWLPFFLVMPIGSFFPDFKPSETVFKIVFWLGYLNSCINPIIYPCSSQEFKKAFCNVLRIQCLRRKQSSKHALGYTLHPPSQAVEGQHKDMVRIPVGSRETFYRISKTDGVCEWKFFSSMPRGSARITVSKDQSSCTTARVRSKSFLQVCCCVGPSTPSLDKNHQVPTIKVHTISLSENGEEV

>AAA52328.1 dopamine D2 receptor, partial [Homo sapiens]

MDPLNLSWYDDDLERQNWSRPFNGSDGKADRPHYNYYATLLTLLIAVIVFGNVLVCMAVSREKALQTTTNYLIVSLAVADLLVATLVMPWVVYLEVVGEWKFSRIHCDIFVTLDVMMCTASILNLCAISIDRYTAVAMPMLYNTRYSSKRRVTVMISIVWVLSFTISCPLLFGLNNA

>AAA88023.1 unknown protein [Homo sapiens]

MRRELEASSSRRRLCPRGPMA

>CAA84542.1 MDR3 P-glycoprotein, partial [Homo sapiens]

MDLEAAKNGTAWRPTSAEGDFELGISSKQKRKKTKTVKMIGVLTLFRYSDWQDKLFMSLGTRGSSRVDLQAC

>prf||2111304A P glycoprotein

ENKKSIEKLKDVISMNASEFSEVQIALNEAKLSEEKVKSECHRVQEENARLKKKKEQLQQEIEDWSKLHAELSEQIKSFEKSQKDLEVALTHKDDNINALTNCITQLNLLECESESEGQNKGGNDSDELANGEVGG

>sp|P25100.2|ADA1D_HUMAN RecName: Full=Alpha-1D adrenergic receptor; AltName: Full=Alpha-1A adrenergic receptor; AltName: Full=Alpha-1D adrenoreceptor; Short=Alpha-1D adrenoceptor; AltName: Full=Alpha-adrenergic receptor 1a

MTFRDLLSVSFEGPRPDSSAGGSSAGGGGGSAGGAAPSEGPAVGGVPGGAGGGGGVVGAGSGEDNRSSAGEPGSAGAGGDVNGTAAVGGLVVSAQGVGVGVFLAAFILMAVAGNLLVILSVACNRHLQTVTNYFIVNLAVADLLLSATVLPFSATMEVLGFWAFGRAFCDVWAAVDVLCCTASILSLCTISVDRYVGVRHSLKYPAIMTERKAAAILALLWVVALVVSVGPLLGWKEPVPPDERFCGITEEAGYAVFSSVCSFYLPMAVIVVMYCRVYVVARSTTRSLEAGVKRERGKASEVVLRIHCRGAATGADGAHGMRSAKGHTFRSSLSVRLLKFSREKKAAKTLAIVVGVFVLCWFPFFFVLPLGSLFPQLKPSEGVFKVIFWLGYFNSCVNPLIYPCSSREFKRAFLRLLRCQCRRRRRRRPLWRVYGHHWRASTSGLRQDCAPSSGDAPPGAPLALTALPDPDPEPPGTPEMQAPVASRRKPPSAFREWRLLGPFRRPTTQLRAKVSSLSHKIRAGGAQRAEAACAQRSEVEAVSLGVPHEVAEGATCQAYELADYSNLRETDI

>sp|P35348.2|ADA1A_HUMAN RecName: Full=Alpha-1A adrenergic receptor; AltName: Full=Alpha-1A adrenoreceptor; Short=Alpha-1A adrenoceptor; AltName: Full=Alpha-1C adrenergic receptor; AltName: Full=Alpha-adrenergic receptor 1c

MVFLSGNASDSSNCTQPPAPVNISKAILLGVILGGLILFGVLGNILVILSVACHRHLHSVTHYYIVNLAVADLLLTSTVLPFSAIFEVLGYWAFGRVFCNIWAAVDVLCCTASIMGLCIISIDRYIGVSYPLRYPTIVTQRRGLMALLCVWALSLVISIGPLFGWRQPAPEDETICQINEEPGYVLFSALGSFYLPLAIILVMYCRVYVVAKRESRGLKSGLKTDKSDSEQVTLRIHRKNAPAGGSGMASAKTKTHFSVRLLKFSREKKAAKTLGIVVGCFVLCWLPFFLVMPIGSFFPDFKPSETVFKIVFWLGYLNSCINPIIYPCSSQEFKKAFQNVLRIQCLCRKQSSKHALGYTLHPPSQAVEGQHKDMVRIPVGSRETFYRISKTDGVCEWKFFSSMPRGSARITVSKDQSSCTTARVRSKSFLQVCCCVGPSTPSLDKNHQVPTIKVHTISLSENGEEV

>sp|P21917.2|DRD4_HUMAN RecName: Full=D(4) dopamine receptor; AltName: Full=D(2C) dopamine receptor; AltName: Full=Dopamine D4 receptor

MGNRSTADADGLLAGRGPAAGASAGASAGLAGQGAAALVGGVLLIGAVLAGNSLVCVSVATERALQTPTNSFIVSLAAADLLLALLVLPLFVYSEVQGGAWLLSPRLCDALMAMDVMLCTASIFNLCAISVDRFVAVAVPLRYNRQGGSRRQLLLIGATWLLSAAVAAPVLCGLNDVRGRDPAVCRLEDRDYVVYSSVCSFFLPCPLMLLLYWATFRGLQRWEVARRAKLHGRAPRRPSGPGPPSPTPPAPRLPQDPCGPDCAPPAPGLPRGPCGPDCAPAAPGLPPDPCGPDCAPPAPGLPQDPCGPDCAPPAPGLPRGPCGPDCAPPAPGLPQDPCGPDCAPPAPGLPPDPCGSNCAPPDAVRAAALPPQTPPQTRRRRRAKITGRERKAMRVLPVVVGAFLLCWTPFFVVHITQALCPACSVPPRLVSAVTWLGYVNSALNPVIYTVFNAEFRNVFRKALRACC

>AAB69423.1 P-glycoprotein [Homo sapiens]

MDLEGDRNGGAKKKNFFKLNNKSEKDKKEKKPTVSVFSMFRYSNWLDKLYMVVGTLAAIIHGAGLPLMMLVFGEMTDIFANAGNLEDLMSNITNRSDINDTGFFMNLEEDMTRYAYYYSGIGAGVLVAAYIQVSFWCLAAGRQIHKIRKQFFHAIMRQEIGWFDVHDVGELNTRLTDDVSKINEGIGDKIGMFFQSMATFFTGFIVGFTRGWKLTLVILAISPVLGLSAAVWAKILSSFTDKELLAYAKAGAVAEEVLAAIRTVIAFGGQKKELERYNKNLEEAKRIGIKKAITANISIGAAFLLIYASYALAFWYGTTLVLSGEYSIGQVLTVFSVLIGAFSVGQASPSIEAFANARGAAYEIFKIIDNKPSIDSYSKSGHKPDNIKGNLEFRNVHFSYPSRKEVKILKGLNLKVQSGQTVALVGNSGCGKSTTVQLMQRLYDPTEGMVSVDGQDIRTINVRFLREIIGVVSQEPVLFATTIAENIRYGRENVTMDEIEKAVKEANAYDFIMKLPHKFDTLVGERGAQLSGGQKQRIAIARALVRNPKILLLDEATSALDTESEAVVQVALDKARKGRTTIVIAHRLSTVRNADVIAGFDDGVIVEKGNHDELMKEKGIYFKLVTMQTAGNEVELENAADESKSEIDALEMSSNDSRSSLIRKRSTRRSVRGSQAQDRKLSTKEALDESIPPVSFWRIMKLNLTEWPYFVVGVFCAIINGGLQPAFAIIFSKIIGVFTRIDDPETKRQNSNLFSLLFLALGIISFITFFLQGFTFGKAGEILTKRLRYMVFRSMLRQDVSWFDDPKNTTGALTTRLANDAAQVKGAIGSRLAVITQNIANLGTGIIISFIYGWQLTLLLLAIVPIIAIAGVVEMKMLSGQALKDKKELEGAGKIATEAIENFRTVVSLTQEQKFEHMYAQSLQVPYRNSLRKAHIFGITFSFTQAMMYFSYAGCFRFGAYLVAHKLMSFEDVLLVFSAVVFGAMAVGQVSSFAPDYAKAKISAAHIIMIIEKTPLIDSYSTEGLMPNTLEGNVTFGEVVFNYPTRPDIPVLQGLSLEVKKGQTLALVGSSGCGKSTVVQLLERFYDPLAGKVLLDGKEIKRLNVQWLRAHLGIVSQEPILFDCSIAENIAYGDNSRVVSQEEIVRAAKEANIHAFIESLPNKYSTKVGDKGTQLSGGQKQRIAIARALVRQPHILLLDEATSALDTESEKVVQEALDKAREGRTCIVIAHRLSTIQNADLIVVFQNGRVKEHGTHQQLLAQKGIYFSMVSVQAGTKRQ

>AAB70218.1 P-glycoprotein, partial [Homo sapiens]

VFSVLIGAFSVGQASPSIEAFANARGAAYEIFKIIDN

>sp|P08183.2|MDR1_HUMAN RecName: Full=Multidrug resistance protein 1; AltName: Full=ATP-binding cassette sub-family B member 1; AltName: Full=P-glycoprotein 1; AltName: CD_antigen=CD243

MDLEGDRNGGAKKKNFFKLNNKSEKDKKEKKPTVSVFSMFRYSNWLDKLYMVVGTLAAIIHGAGLPLMMLVFGEMTDIFANAGNLEDLMSNITNRSDINDTGFFMNLEEDMTRYAYYYSGIGAGVLVAAYIQVSFWCLAAGRQIHKIRKQFFHAIMRQEIGWFDVHDVGELNTRLTDDVSKINEGIGDKIGMFFQSMATFFTGFIVGFTRGWKLTLVILAISPVLGLSAAVWAKILSSFTDKELLAYAKAGAVAEEVLAAIRTVIAFGGQKKELERYNKNLEEAKRIGIKKAITANISIGAAFLLIYASYALAFWYGTTLVLSGEYSIGQVLTVFFSVLIGAFSVGQASPSIEAFANARGAAYEIFKIIDNKPSIDSYSKSGHKPDNIKGNLEFRNVHFSYPSRKEVKILKGLNLKVQSGQTVALVGNSGCGKSTTVQLMQRLYDPTEGMVSVDGQDIRTINVRFLREIIGVVSQEPVLFATTIAENIRYGRENVTMDEIEKAVKEANAYDFIMKLPHKFDTLVGERGAQLSGGQKQRIAIARALVRNPKILLLDEATSALDTESEAVVQVALDKARKGRTTIVIAHRLSTVRNADVIAGFDDGVIVEKGNHDELMKEKGIYFKLVTMQTAGNEVELENAADESKSEIDALEMSSNDSRSSLIRKRSTRRSVRGSQAQDRKLSTKEALDESIPPVSFWRIMKLNLTEWPYFVVGVFCAIINGGLQPAFAIIFSKIIGVFTRIDDPETKRQNSNLFSLLFLALGIISFITFFLQGFTFGKAGEILTKRLRYMVFRSMLRQDVSWFDDPKNTTGALTTRLANDAAQVKGAIGSRLAVITQNIANLGTGIIISFIYGWQLTLLLLAIVPIIAIAGVVEMKMLSGQALKDKKELEGAGKIATEAIENFRTVVSLTQEQKFEHMYAQSLQVPYRNSLRKAHIFGITFSFTQAMMYFSYAGCFRFGAYLVAHKLMSFEDVLLVFSAVVFGAMAVGQVSSFAPDYAKAKISAAHIIMIIEKTPLIDSYSTEGLMPNTLEGNVTFGEVVFNYPTRPDIPVLQGLSLEVKKGQTLALVGSSGCGKSTVVQLLERFYDPLAGKVLLDGKEIKRLNVQWLRAHLGIVSQEPILFDCSIAENIAYGDNSRVVSQEEIVRAAKEANIHAFIESLPNKYSTKVGDKGTQLSGGQKQRIAIARALVRQPHILLLDEATSALDTESEKVVQEALDKAREGRTCIVIAHRLSTIQNADLIVVFQNGRVKEHGTHQQLLAQKGIYFSMVSVQAGTKRQ

>CAA84543.1 multidrug resistance protein 3, partial [Homo sapiens]

MDLEAAKNGTAWRPTSAEGDFELGISR

>AAC06138.1 alpha 1A adrenergic receptor isoform 4 [Homo sapiens]

MVFLSGNASDSSNCTQPPAPVNISKAILLGVILGGLILFGVLGNILVILSVACHRHLHSVTHYYIVNLAVADLLLTSTVLPFSAIFEVLGYWAFGRVFCNIWAAVDVLCCTASIMGLCIISIDRYIGVSYPLRYPTIVTQRRGLMALLCVWALSLVISIGPLFGWRQPAPEDETICQINEEPGYVLFSALGSFYLPLAIILVMYCRVYVVAKRESRGLKSGLKTDKSDSEQVTLRIHRKNAPAGGSGMASAKTKTHFSVRLLKFSREKKAAKTLGIVVGCFVLCWLPFFLVMPIGSFFPDFKPSETVFKIVFWLGYLNSCINPIIYPCSSQEFKKAFQNVLRIQCLCRKQSSKHALGYTLHPPSQAVEGQHKDMVRIPVGSRETFYRISKTDGVCEWKFFSSMPRGSARITVSKDQSSCTTARRGMDCRYFTKNCREHIKHVNFMMPPWRKGLEC

>CAA07081.1 m1 muscarinic acetylcholine receptor protein, partial [Homo sapiens]

MNTSAPPAVSP

>CAA09232.1 ether-a-go-go-related protein, partial [Homo sapiens]

RKFIIANARVENCAVIYCNDGFCELCGYSRAEVMQRPCTCDFLHGPRTQRRAAAQIAQALLGAEERKVEIAFYRKDGSCFLCLVDVVPVKNEDGAVIMFILNFEVVMEKDMVGSPAHDTNHRGPPTSWLAPGRAKTFRLKLPALLALTARESSVRSGGAGGAGAPGAVVVDVDLTPAAPSSESLALDEVTAMDNHVAGLGPAEERRALVGPGSPPRSAPGQLPSPRAHSLNPDASGSSCSLARTRSRESCASVRRASSADDIEAMRAGVLPPPPRHASTGAMHPLRSGLLNSTSDSDLVRYRTISKIPQITLNFVDLKGDPFLASPTSDREIIAPKIKERTHNVTEKVTQVLSLGADVLPEYKLQAPRIHRWTILHYSPFKAVWDWLILLLVIYTAVFTPYSAAFLLKETEEGPPATECGYACQPLAVVDLIVDIMFIVDILINFRTTYVNANEEVVSHPGRIAVHYFKGWFLIDMVAAIPFDLLIFGSGSEELIGLLKTARLLRLVRVARKLDRYSEYGAAVLFLLMCTFALIAHWLACIWYAIGNMEQPHMDSRIGWLHNLGDQIGKPYNSSGLGGPSIKDKYVTALYFTFSSLTSVGFGNVSPNTNSEKIFSICVMLIGSLMYASIFGNVSAIIQRLYSGTARYHTQMLRVREFIRFHQIPNPLRQRLEEYFQHAWSYTNGIDMNAVLKGFPECLQADICLHLNRSLLQHCKPFRGATKGCLRALAMKFKTTHAPPGDTLVHAGDLLTALYFISRGSIEILRGDVVVAILGKNDIFGEPLNLYARPGKSNGDVRALTYCDLHKIHRDDLLEVLDMYPEFSDHFWSSLEITFNLRDTNMIPGSPGSTELEGGFSRQRKRKLSFRRRTDKDTEQPGEVSALGPGRAGAGPSSRGRPGGPWGESPSSGPSSPESSEDEGPGRSSSPLRLVPFSSPRPPGEPPGGEPLMEDCEKSSDTCNPLSGAFSGVSNIFSFWGDSRGRQYQELPRCPAPTPSLLNIPLSSPGRRPRGDVESRLDALQRQLNRLETRLSADMATVLQLLQRQMTLVPPAYSAVTTPGPGPTSTSPLLPVSPLPTLTLDSLSQFPSSWRVRSCPRGPQSFPKKAPHDASPYRASWGPSPPSPCTDTARTRAV

>AAC69709.1 HERG-USO, partial [Homo sapiens]

VVAILGMGWGAGTGLEMPSAASRGASLLNMQSLGLWTWDCLQGHWAPLIHLNSGPPSGAMERSPTWGEAAELWGSHILLPFRIRHKQTLFASLK

>AAC78779.1 dopamine D2 receptor [Homo sapiens]

MDPLNLSWYDDDLERQNWSRPFNGSDGKADRPHYNYYATLLTLLIAVIVFGNVLVCMAVSREKALQTTTNYLIVSLAVADLLVATLVMPWVVYLEVVGEWKFSRIHCDIFVTLDVMMCTASILNLCAISIDRYTAVAMPMLYNTRYSSKRRVTVMISIVWVLSFTISCPLLFGLNNADQNECIIANPAFVVYSSIVSFYVPFIVTLLVYIKIYIVLRRRRKRVNTKRSSRAFRAHLRAPLKGNCTHPEDMKLCTVIMKSNGSFPVNRRRVEAARRAQELEMEMLSSTSPPERTRYSPIPPSHHQLTLPDPSHHGLHSTPDSPAKPEKNGHAKDHPKIAKIFEIQTMPNGKXRTSLKTMSRRKLSQQKEKKATQMLAIVLGVFIICWLPFFITHILNIHCDCNIPPVLYSAFTWLGYVNSAVNPIIYTTFNIEFRKAFLKILHC

>sp|Q14524.1|SCN5A_HUMAN RecName: Full=Sodium channel protein type 5 subunit alpha; AltName: Full=Sodium channel protein type V subunit alpha; AltName: Full=Voltage-gated sodium channel subunit alpha Nav1.5; AltName: Full=Sodium channel protein cardiac muscle subunit alpha; AltName: Full=HH1

MANFLLPRGTSSFRRFTRESLAAIEKRMAEKQARGSTTLQESREGLPEEEAPRPQLDLQASKKLPDLYGNPPQELIGEPLEDLDPFYSTQKTFIVLNKGKTIFRFSATNALYVLSPFHPVRRAAVKILVHSLFNMLIMCTILTNCVFMAQHDPPPWTKYVEYTFTAIYTFESLVKILARAFCLHAFTFLRDPWNWLDFSVIIMAYTTEFVDLGNVSALRTFRVLRALKTISVISGLKTIVGALIQSVKKLADVMVLTVFCLSVFALIGLQLFMGNLRHKCVRNFTALNGTNGSVEADGLVWESLDLYLSDPENYLLKNGTSDVLLCGNSSDAGTCPEGYRCLKAGENPDHGYTSFDSFAWAFLALFRLMTQDCWERLYQQTLRSAGKIYMIFFMLVIFLGSFYLVNLILAVVAMAYEEQNQATIAETEEKEKRFQEAMEMLKKEHEALTIRGVDTVSRSSLEMSPLAPVNSHERRSKRRKRMSSGTEECGEDRLPKSDSEDGPRAMNHLSLTRGLSRTSMKPRSSRGSIFTFRRRDLGSEADFADDENSTARESESHHTSLLVPWPLRRTSAQGQPSPGTSAPGHALHGKKNSTVDCNGVVSLLGAGDPEATSPGSHLLRPVMLEHPPDTTTPSEEPGGPQMLTSQAPCVDGFEEPGARQRALSAVSVLTSALEELEESRHKCPPCWNRLAQRYLIWECCPLWMSIKQGVKLVVMDPFTDLTITMCIVLNTLFMALEHYNMTSEFEEMLQVGNLVFTGIFTAEMTFKIIALDPYYYFQQGWNIFDSIIVILSLMELGLSRMSNLSVLRSFRLLRVFKLAKSWPTLNTLIKIIGNSVGALGNLTLVLAIIVFIFAVVGMQLFGKNYSELRDSDSGLLPRWHMMDFFHAFLIIFRILCGEWIETMWDCMEVSGQSLCLLVFLLVMVIGNLVVLNLFLALLLSSFSADNLTAPDEDREMNNLQLALARIQRGLRFVKRTTWDFCCGLLRHRPQKPAALAAQGQLPSCIATPYSPPPPETEKVPPTRKETQFEEGEQPGQGTPGDPEPVCVPIAVAESDTDDQEEDEENSLGTEEESSKQQESQPVSGWPRGPPDSRTWSQVSATASSEAEASASQADWRQQWKAEPQAPGCGETPEDSCSEGSTADMTNTAELLEQIPDLGQDVKDPEDCFTEGCVRRCPCCAVDTTQAPGKVWWRLRKTCYHIVEHSWFETFIIFMILLSSGALAFEDIYLEERKTIKVLLEYADKMFTYVFVLEMLLKWVAYGFKKYFTNAWCWLDFLIVDVSLVSLVANTLGFAEMGPIKSLRTLRALRPLRALSRFEGMRVVVNALVGAIPSIMNVLLVCLIFWLIFSIMGVNLFAGKFGRCINQTEGDLPLNYTIVNNKSQCESLNLTGELYWTKVKVNFDNVGAGYLALLQVATFKGWMDIMYAAVDSRGYEEQPQWEYNLYMYIYFVIFIIFGSFFTLNLFIGVIIDNFNQQKKKLGGQDIFMTEEQKKYYNAMKKLGSKKPQKPIPRPLNKYQGFIFDIVTKQAFDVTIMFLICLNMVTMMVETDDQSPEKINILAKINLLFVAIFTGECIVKLAALRHYYFTNSWNIFDFVVVILSIVGTVLSDIIQKYFFSPTLFRVIRLARIGRILRLIRGAKGIRTLLFALMMSLPALFNIGLLLFLVMFIYSIFGMANFAYVKWEAGIDDMFNFQTFANSMLCLFQITTSAGWDGLLSPILNTGPPYCDPTLPNSNGSRGDCGSPAVGILFFTTYIIISFLIVVNMYIAIILENFSVATEESTEPLSEDDFDMFYEIWEKFDPEATQFIEYSVLSDFADALSEPLRIAKPNQISLINMDLPMVSGDRIHCMDILFAFTKRVLGESGEMDALKIQMEEKFMAANPSKISYEPITTTLRRKHEEVSAMVIQRAFRRHLLQRSLKHASFLFRQQAGSGLSEEDAPEREGLIAYVMSENFSRPLGPPSSSSISSTSFPPSYDSVTRATSDNLQVRGSDYSHSEDLADFPPSPDRDRESIV

>BAA37096.1 HERG [Homo sapiens]

MPVRRGHVAPQNTFLDTIIRKFEGQSRKFIIANARVENCAVIYCNDGFCELCGYSRAEVMQRPCTCDFLHGPRTQRRAAAQIAQALLGAEERKVEIAFYRKDGSCFLCLVDVVPVKNEDGAVIMFILNFEVVMEKDMVGSPAHDTNHRGPPTSWLAPGRAKTFRLKLPALLALTARESSVRSGGAGGAGAPGAVVVDVDLTPAAPSSESLALDEVTAMDNHVAGLGPAEERRALVGPGSPPRSAPGQLPSPRAHSLNPDASGSSCSLARTRSRESCASVRRASSADDIEAMRAGVLPPPPRHASTGAMHPLRSGLLNSTSDSDLVRYRTISKIPQITLNFVDLKGDPFLASPTSDREIIAPKIKERTHNVTEKVTQVLSLGADVLPEYKLQAPRIHRWTILHYSPFKAVWDWLILLLVIYTAVFTPYSAAFLLKETEEGPPATECGYACQPLAVVDLIVDIMFIVDILINFRTTYVNANEEVVSHPGRIAVHYFKGWFLIDMVAAIPFDLLIFGSGSEELIGLLKTARLLRLVRVARKLDRYSEYGAAVLFLLMCTFALIAHWLACIWYAIGNMEQPHMDSRIGWLHNLGDQIGKPYNSSGLGGPSIKDKYVTALYFTFSSLTSVGFGNVSPNTNSEKIFSICVMLIGSLMYASIFGNVSAIIQRLYSGTARYHTQMLRVREFIRFHQIPNPLRQRLEEYFQHAWSYTNGIDMNAVLKGFPECLQADICLHLNRSLLQHCKPFRGATKGCLRALAMKFKTTHAPPGDTLVHAGDLLTALYFISRGSIEILRGDVVVAILGKNDIFGEPLNLYARPGKSNGDVRALTYCDLHKIHRDDLLEVLDMYPEFSDHFWSSLEITFNLRDTNMIPGSPGSTELEGGFSRQRKRKLSFRRRTDKDTEQPGEVSALGPGRAGAGPSSRGRPGGPWGESPSSGPSSPESSEDEGPGRSSSPLRLVPFSSPRPPGEPPGGEPLMEDCEKSSDTCNPLSGAFSGVSNIFSFWGDSRGRQYQELPRCPAPTPSLLNIPLSSPGRRPRGDVESRLDALQRQLNRLETRLSADMATVLQLLQRQMTLVPPAYSAVTTPGPGPTSTSPLLPVSPLPTLTLDSLSQVSQFMACEELPPGAPELPQEGPTRRLSLPGQLGALTSQPLHRHGSDPGS

>CAB37869.1 dopamine receptor D2, partial [Homo sapiens]

QNECIIANPAFVVYSSIVSFYVPFIVTLLVYIKIYIVLRRRRKRVNTKRSSRAFRAHLRAPLKGNCTHPEDMKLCTVIMKSNGSFPVNRRRVEAARRAQELEMEMLSSTSPPERTRYSPIPPSHHQLTLPDPSHHGLHSTPDSPAKPEKNGHAKDHPKIAKIFEIQTMPNGKTRTSLKTMSRRKLSQQKEKKATQMLAIVL

>NP_000670.1 alpha-1B adrenergic receptor [Homo sapiens]

MNPDLDTGHNTSAPAHWGELKNANFTGPNQTSSNSTLPQLDITRAISVGLVLGAFILFAIVGNILVILSVACNRHLRTPTNYFIVNLAMADLLLSFTVLPFSAALEVLGYWVLGRIFCDIWAAVDVLCCTASILSLCAISIDRYIGVRYSLQYPTLVTRRKAILALLSVWVLSTVISIGPLLGWKEPAPNDDKECGVTEEPFYALFSSLGSFYIPLAVILVMYCRVYIVAKRTTKNLEAGVMKEMSNSKELTLRIHSKNFHEDTLSSTKAKGHNPRSSIAVKLFKFSREKKAAKTLGIVVGMFILCWLPFFIALPLGSLFSTLKPPDAVFKVVFWLGYFNSCLNPIIYPCSSKEFKRAFVRILGCQCRGRGRRRRRRRRRLGGCAYTYRPWTRGGSLERSQSRKDSLDDSGSCLSGSQRTLPSASPSPGYLGRGAPPPVELCAFPEWKAPGALLSLPAPEPPGRRGRHDSGPLFTFKLLTEPESPGTDGGASNGGCEAAADVANGQPGFKSNMPLAPGQF

>NP_001901.1 cathepsin E isoform a preproprotein [Homo sapiens]

MKTLLLLLLVLLELGEAQGSLHRVPLRRHPSLKKKLRARSQLSEFWKSHNLDMIQFTESCSMDQSAKEPLINYLDMEYFGTISIGSPPQNFTVIFDTGSSNLWVPSVYCTSPACKTHSRFQPSQSSTYSQPGQSFSIQYGTGSLSGIIGADQVSVEGLTVVGQQFGESVTEPGQTFVDAEFDGILGLGYPSLAVGGVTPVFDNMMAQNLVDLPMFSVYMSSNPEGGAGSELIFGGYDHSHFSGSLNWVPVTKQAYWQIALDNIQVGGTVMFCSEGCQAIVDTGTSLITGPSDKIKQLQNAIGAAPVDGEYAVECANLNVMPDVTFTINGVPYTLSPTAYTLLDFVDGMQFCSSGFQGLDIHPPAGPLWILGDVFIRQFYSVFDRGNNRVGLAPAVP

>NP_000785.1 D(1A) dopamine receptor [Homo sapiens]

MRTLNTSAMDGTGLVVERDFSVRILTACFLSLLILSTLLGNTLVCAAVIRFRHLRSKVTNFFVISLAVSDLLVAVLVMPWKAVAEIAGFWPFGSFCNIWVAFDIMCSTASILNLCVISVDRYWAISSPFRYERKMTPKAAFILISVAWTLSVLISFIPVQLSWHKAKPTSPSDGNATSLAETIDNCDSSLSRTYAISSSVISFYIPVAIMIVTYTRIYRIAQKQIRRIAALERAAVHAKNCQTTTGNGKPVECSQPESSFKMSFKRETKVLKTLSVIMGVFVCCWLPFFILNCILPFCGSGETQPFCIDSNTFDVFVWFGWANSSLNPIIYAFNADFRKAFSTLLGCYRLCPATNNAIETVSINNNGAAMFSSHHEPRGSISKECNLVYLIPHAVGSSEDLKKEEAAGIARPLEKLSPALSVILDYDTDVSLEKIQPITQNGQHPT

>NP_000805.1 gamma-aminobutyric acid receptor subunit beta-3 isoform 1 precursor [Homo sapiens]

MWGLAGGRLFGIFSAPVLVAVVCCAQSVNDPGNMSFVKETVDKLLKGYDIRLRPDFGGPPVCVGMNIDIASIDMVSEVNMDYTLTMYFQQYWRDKRLAYSGIPLNLTLDNRVADQLWVPDTYFLNDKKSFVHGVTVKNRMIRLHPDGTVLYGLRITTTAACMMDLRRYPLDEQNCTLEIESYGYTTDDIEFYWRGGDKAVTGVERIELPQFSIVEHRLVSRNVVFATGAYPRLSLSFRLKRNIGYFILQTYMPSILITILSWVSFWINYDASAARVALGITTVLTMTTINTHLRETLPKIPYVKAIDMYLMGCFVFVFLALLEYAFVNYIFFGRGPQRQKKLAEKTAKAKNDRSKSESNRVDAHGNILLTSLEVHNEMNEVSGGIGDTRNSAISFDNSGIQYRKQSMPREGHGRFLGDRSLPHKKTHLRRRSSQLKIKIPDLTDVNAIDRWSRIVFPFTFSLFNLVYWLYYVN

>NP_002061.1 guanine nucleotide-binding protein G(i) subunit alpha-2 isoform 1 [Homo sapiens]

MGCTVSAEDKAAAERSKMIDKNLREDGEKAAREVKLLLLGAGESGKSTIVKQMKIIHEDGYSEEECRQYRAVVYSNTIQSIMAIVKAMGNLQIDFADPSRADDARQLFALSCTAEEQGVLPDDLSGVIRRLWADHGVQACFGRSREYQLNDSAAYYLNDLERIAQSDYIPTQQDVLRTRVKTTGIVETHFTFKDLHFKMFDVGGQRSERKKWIHCFEGVTAIIFCVALSAYDLVLAEDEEMNRMHESMKLFDSICNNKWFTDTSIILFLNKKDLFEEKITHSPLTICFPEYTGANKYDEAASYIQSKFEDLNKRKDTKEIYTHFTCATDTKNVQFVFDAVTDVIIKNNLKDCGLF

>NP_002788.1 proteasome subunit beta type-5 isoform 1 [Homo sapiens]

MALASVLERPLPVNQRGFFGLGGRADLLDLGPGSLSDGLSLAAPGWGVPEEPGIEMLHGTTTLAFKFRHGVIVAADSRATAGAYIASQTVKKVIEINPYLLGTMAGGAADCSFWERLLARQCRIYELRNKERISVAAASKLLANMVYQYKGMGLSMGTMICGWDKRGPGLYYVDSEGNRISGATFSVGSGSVYAYGVMDRGYSYDLEVEQAYDLARRAIYQATYRDAYSGGAVNLYHVREDGWIRVSSDNVADLHEKYSGSTP

>NP_003295.1 short transient receptor potential channel 1 isoform 2 [Homo sapiens]

MMAALYPSTDLSGASSSSLPSSPSSSSPNEVMALKDVREVKEENTLNEKLFLLACDKGDYYMVKKILEENSSGDLNINCVDVLGRNAVTITIENENLDILQLLLDYGCQKLMERIQNPEYSTTMDVAPVILAAHRNNYEILTMLLKQDVSLPKPHAVGCECTLCSAKNKKDSLRHSRFRLDIYRCLASPALIMLTEEDPILRAFELSADLKELSLVEVEFRNDYEELARQCKMFAKDLLAQARNSRELEVILNHTSSDEPLDKRGLLEERMNLSRLKLAIKYNQKEFVSQSNCQQFLNTVWFGQMSGYRRKPTCKKIMTVLTVGIFWPVLSLCYLIAPKSQFGRIIHTPFMKFIIHGASYFTFLLLLNLYSLVYNEDKKNTMGPALERIDYLLILWIIGMIWSDIKRLWYEGLEDFLEESRNQLSFVMNSLYLATFALKVVAHNKFHDFADRKDWDAFHPTLVAEGLFAFANVLSYLRLFFMYTTSSILGPLQISMGQMLQDFGKFLGMFLLVLFSFTIGLTQLYDKGYTSKEQKDCVGIFCEQQSNDTFHSFIGTCFALFWYIFSLAHVAIFVTRFSYGEELQSFVGAVIVGTYNVVVVIVLTKLLVAMLHKSFQLIANHEDKEWKFARAKLWLSYFDDKCTLPPPFNIIPSPKTICYMISSLSKWICSHTSKGKVKRQNSLKEWRNLKQKRDENYQKVMCCLVHRYLTSMRQKMQSTDQATVENLNELRQDLSKFRNEIRDLLGFRTSKYAMFYPRN

>NP_003349.1 ceramide glucosyltransferase [Homo sapiens]

MALLDLALEGMAVFGFVLFLVLWLMHFMAIIYTRLHLNKKATDKQPYSKLPGVSLLKPLKGVDPNLINNLETFFELDYPKYEVLLCVQDHDDPAIDVCKKLLGKYPNVDARLFIGGKKVGINPKINNLMPGYEVAKYDLIWICDSGIRVIPDTLTDMVNQMTEKVGLVHGLPYVADRQGFAATLEQVYFGTSHPRYYISANVTGFKCVTGMSCLMRKDVLDQAGGLIAFAQYIAEDYFMAKAIADRGWRFAMSTQVAMQNSGSYSISQFQSRMIRWTKLRINMLPATIICEPISECFVASLIIGWAAHHVFRWDIMVFFMCHCLAWFIFDYIQLRGVQGGTLCFSKLDYAVAWFIRESMTIYIFLSALWDPTISWRTGRYRLRCGGTAEEILDV

>NP_000605.1 ciliary neurotrophic factor [Homo sapiens]

MAFTEHSPLTPHRRDLCSRSIWLARKIRSDLTALTESYVKHQGLNKNINLDSADGMPVASTDQWSELTEAERLQENLQAYRTFHVLLARLLEDQQVHFTPTEGDFHQAIHTLLLQVAAFAYQIEELMILLEYKIPRNEADGMPINVGDGGLFEKKLWGLKVLQELSQWTVRSIHDLRFISSHQTGIPARGSHYIANNKKM

>NP_005113.1 nuclear receptor subfamily 1 group I member 3 isoform 3 [Homo sapiens]

MASREDELRNCVVCGDQATGYHFNALTCEGCKGFFRRTVSKSIGPTCPFAGSCEVSKTQRRHCPACRLQKCLDAGMRKDMILSAEALALRRAKQAQRRAQQTPVQLSKEQEELIRTLLGAHTRHMGTMFEQFVQFRPPAHLFIHHQPLPTLAPVLPLVTHFADINTFMVLQVIKFTKDLPVFRSLPIEDQISLLKGAAVEICHIVLNTTFCLQTQNFLCGPLRYTIEDGARVGFQVEFLELLFHFHGTLRKLQLQEPEYVLLAAMALFSPDRPGVTQRDEIDQLQEEMALTLQSYIKGQQRRPRDRFLYAKLLGLLAELRSINEAYGYQIQHIQGLSAMMPLLQEICS

>NP_005463.1 potassium voltage-gated channel subfamily E member 3 [Homo sapiens]

METTNGTETWYESLHAVLKALNATLHSNLLCRPGPGLGPDNQTEERRASLPGRDDNSYMYILFVMFLFAVTVGSLILGYTRSRKVDKRSDPYHVYIKNRVSMI

>NP_005391.1 protein kinase C epsilon type [Homo sapiens]

MVVFNGLLKIKICEAVSLKPTAWSLRHAVGPRPQTFLLDPYIALNVDDSRIGQTATKQKTNSPAWHDEFVTDVCNGRKIELAVFHDAPIGYDDFVANCTIQFEELLQNGSRHFEDWIDLEPEGRVYVIIDLSGSSGEAPKDNEERVFRERMRPRKRQGAVRRRVHQVNGHKFMATYLRQPTYCSHCRDFIWGVIGKQGYQCQVCTCVVHKRCHELIITKCAGLKKQETPDQVGSQRFSVNMPHKFGIHNYKVPTFCDHCGSLLWGLLRQGLQCKVCKMNVHRRCETNVAPNCGVDARGIAKVLADLGVTPDKITNSGQRRKKLIAGAESPQPASGSSPSEEDRSKSAPTSPCDQEIKELENNIRKALSFDNRGEEHRAASSPDGQLMSPGENGEVRQGQAKRLGLDEFNFIKVLGKGSFGKVMLAELKGKDEVYAVKVLKKDVILQDDDVDCTMTEKRILALARKHPYLTQLYCCFQTKDRLFFVMEYVNGGDLMFQIQRSRKFDEPRSRFYAAEVTSALMFLHQHGVIYRDLKLDNILLDAEGHCKLADFGMCKEGILNGVTTTTFCGTPDYIAPEILQELEYGPSVDWWALGVLMYEMMAGQPPFEADNEDDLFESILHDDVLYPVWLSKEAVSILKAFMTKNPHKRLGCVASQNGEDAIKQHPFFKEIDWVLLEQKKIKPPFKPRIKTKRDVNNFDQDFTREEPVLTLVDEAIVKQINQEEFKGFSYFGEDLMP

>NP_006570.1 3-beta-hydroxysteroid-Delta(8),Delta(7)-isomerase [Homo sapiens]

MTTNAGPLHPYWPQHLRLDNFVPNDRPTWHILAGLFSVTGVLVVTTWLLSGRAAVVPLGTWRRLSLCWFAVCGFIHLVIEGWFVLYYEDLLGDQAFLSQLWKEYAKGDSRYILGDNFTVCMETITACLWGPLSLWVVIAFLRQHPLRFILQLVVSVGQIYGDVLYFLTEHRDGFQHGELGHPLYFWFYFVFMNALWLVLPGVLVLDAVKHLTHAQSTLDAKATKAKSKKN

>NP_006752.1 14-3-3 protein epsilon [Homo sapiens]

MDDREDLVYQAKLAEQAERYDEMVESMKKVAGMDVELTVEERNLLSVAYKNVIGARRASWRIISSIEQKEENKGGEDKLKMIREYRQMVETELKLICCDILDVLDKHLIPAANTGESKVFYYKMKGDYHRYLAEFATGNDRKEAAENSLVAYKAASDIAMTELPPTHPIRLGLALNFSVFYYEILNSPDRACRLAKAAFDDAIAELDTLSEESYKDSTLIMQLLRDNLTLWTSDMQGDGEEQNKEALQDVEDENQ

>CAB56463.1 dopamine receptor D2 [Homo sapiens]

MDPLNLSWYDDDLERQNWSRPFNGSDGKADRPHYNYYATLLTLLIAVIVFGNVLVCMAVSREKALQTTTNYLIVSLAVADLLVATLVMPWVVYLEVVGEWKFSRIHCDIFVTLDVMMCTASILNLCAISIDRYTAVAMPMLYNTRYSSKRRVTVMISIVWVLSFTISCPLLFGLNNADQNECIIANPAFVVYSSIVSFYVPFIVTLLVYIKIYIVLRRRRKRVNTKRSSRAFRAHLRAPLKGNCTHPEDMKLCTVIMKSNGSFPVNRRRVEAARRAQELEMEMLSSTSPPERTRYSPIPPSHHQLTLPDPSHHGLHSTPDSPAKPEKNGHAKDHPKIAKIFEIQTMPNGKTRTSLKTMSRRKLSQQKEKKATQMLAIVLGVFIICWLPFFITHILNIHCDCNIPPVLYSAFTWLGYVNSAVNPIIYTTFNIEFRKAFLKILHC

>sp|O43525.2|KCNQ3_HUMAN RecName: Full=Potassium voltage-gated channel subfamily KQT member 3; AltName: Full=KQT-like 3; AltName: Full=Potassium channel subunit alpha KvLQT3; AltName: Full=Voltage-gated potassium channel subunit Kv7.3

MGLKARRAAGAAGGGGDGGGGGGGAANPAGGDAAAAGDEERKVGLAPGDVEQVTLALGAGADKDGTLLLEGGGRDEGQRRTPQGIGLLAKTPLSRPVKRNNAKYRRIQTLIYDALERPRGWALLYHALVFLIVLGCLILAVLTTFKEYETVSGDWLLLLETFAIFIFGAEFALRIWAAGCCCRYKGWRGRLKFARKPLCMLDIFVLIASVPVVAVGNQGNVLATSLRSLRFLQILRMLRMDRRGGTWKLLGSAICAHSKELITAWYIGFLTLILSSFLVYLVEKDVPEVDAQGEEMKEEFETYADALWWGLITLATIGYGDKTPKTWEGRLIAATFSLIGVSFFALPAGILGSGLALKVQEQHRQKHFEKRRKPAAELIQAAWRYYATNPNRIDLVATWRFYESVVSFPFFRKEQLEAASSQKLGLLDRVRLSNPRGSNTKGKLFTPLNVDAIEESPSKEPKPVGLNNKERFRTAFRMKAYAFWQSSEDAGTGDPMAEDRGYGNDFPIEDMIPTLKAAIRAVRILQFRLYKKKFKETLRPYDVKDVIEQYSAGHLDMLSRIKYLQTRIDMIFTPGPPSTPKHKKSQKGSAFTFPSQQSPRNEPYVARPSTSEIEDQSMMGKFVKVERQVQDMGKKLDFLVDMHMQHMERLQVQVTEYYPTKGTSSPAEAEKKEDNRYSDLKTIICNYSETGPPEPPYSFHQVTIDKVSPYGFFAHDPVNLPRGGPSSGKVQATPPSSATTYVERPTVLPILTLLDSRVSCHSQADLQGPYSDRISPRQRRSITRDSDTPLSLMSVNHEELERSPSGFSISQDRDDYVFGPNGGSSWMREKRYLAEGETDTDTDPFTPSGSMPLSSTGDGISDSVWTPSNKPI

>NP_000666.2 adenosine receptor A2a [Homo sapiens]

MPIMGSSVYITVELAIAVLAILGNVLVCWAVWLNSNLQNVTNYFVVSLAAADIAVGVLAIPFAITISTGFCAACHGCLFIACFVLVLTQSSIFSLLAIAIDRYIAIRIPLRYNGLVTGTRAKGIIAICWVLSFAIGLTPMLGWNNCGQPKEGKNHSQGCGEGQVACLFEDVVPMNYMVYFNFFACVLVPLLLMLGVYLRIFLAARRQLKQMESQPLPGERARSTLQKEVHAAKSLAIIVGLFALCWLPLHIINCFTFFCPDCSHAPLWLMYLAIVLSHTNSVVNPFIYAYRIREFRQTFRKIIRSHVLRQQEPFKAAGTSARVLAAHGSDGEQVSLRLNGHPPGVWANGSAPHPERRPNGYALGLVSGGSAQESQGNTGLPDVELLSHELKGVCPEPPGLDDPLAQDGAGVS

>NP_009143.1 E3 ubiquitin-protein ligase RING2 [Homo sapiens]

MSQAVQTNGTQPLSKTWELSLYELQRTPQEAITDGLEIVVSPRSLHSELMCPICLDMLKNTMTTKECLHRFCADCIITALRSGNKECPTCRKKLVSKRSLRPDPNFDALISKIYPSRDEYEAHQERVLARINKHNNQQALSHSIEEGLKIQAMNRLQRGKKQQIENGSGAEDNGDSSHCSNASTHSNQEAGPSNKRTKTSDDSGLELDNNNAAMAIDPVMDGASEIELVFRPHPTLMEKDDSAQTRYIKTSGNATVDHLSKYLAVRLALEELRSKGESNQMNLDTASEKQYTIYIATASGQFTVLNGSFSLELVSEKYWKVNKPMELYYAPTKEHK

>sp|O75469.1|NR1I2_HUMAN RecName: Full=Nuclear receptor subfamily 1 group I member 2; AltName: Full=Orphan nuclear receptor PAR1; AltName: Full=Orphan nuclear receptor PXR; AltName: Full=Pregnane X receptor; AltName: Full=Steroid and xenobiotic receptor; Short=SXR

MEVRPKESWNHADFVHCEDTESVPGKPSVNADEEVGGPQICRVCGDKATGYHFNVMTCEGCKGFFRRAMKRNARLRCPFRKGACEITRKTRRQCQACRLRKCLESGMKKEMIMSDEAVEERRALIKRKKSERTGTQPLGVQGLTEEQRMMIRELMDAQMKTFDTTFSHFKNFRLPGVLSSGCELPESLQAPSREEAAKWSQVRKDLCSLKVSLQLRGEDGSVWNYKPPADSGGKEIFSLLPHMADMSTYMFKGIISFAKVISYFRDLPIEDQISLLKGAAFELCQLRFNTVFNAETGTWECGRLSYCLEDTAGGFQQLLLEPMLKFHYMLKKLQLHEEEYVLMQAISLFSPDRPGVLQHRVVDQLQEQFAITLKSYIECNRPQPAHRFLFLKIMAMLTELRSINAQHTQRLLRIQDIHPFATPLMQELFGITGS

>sp|P51787.3|KCNQ1_HUMAN RecName: Full=Potassium voltage-gated channel subfamily KQT member 1; AltName: Full=IKs producing slow voltage-gated potassium channel subunit alpha KvLQT1; AltName: Full=KQT-like 1; AltName: Full=Voltage-gated potassium channel subunit Kv7.1

MAAASSPPRAERKRWGWGRLPGARRGSAGLAKKCPFSLELAEGGPAGGALYAPIAPGAPGPAPPASPAAPAAPPVASDLGPRPPVSLDPRVSIYSTRRPVLARTHVQGRVYNFLERPTGWKCFVYHFAVFLIVLVCLIFSVLSTIEQYAALATGTLFWMEIVLVVFFGTEYVVRLWSAGCRSKYVGLWGRLRFARKPISIIDLIVVVASMVVLCVGSKGQVFATSAIRGIRFLQILRMLHVDRQGGTWRLLGSVVFIHRQELITTLYIGFLGLIFSSYFVYLAEKDAVNESGRVEFGSYADALWWGVVTVTTIGYGDKVPQTWVGKTIASCFSVFAISFFALPAGILGSGFALKVQQKQRQKHFNRQIPAAASLIQTAWRCYAAENPDSSTWKIYIRKAPRSHTLLSPSPKPKKSVVVKKKKFKLDKDNGVTPGEKMLTVPHITCDPPEERRLDHFSVDGYDSSVRKSPTLLEVSMPHFMRTNSFAEDLDLEGETLLTPITHISQLREHHRATIKVIRRMQYFVAKKKFQQARKPYDVRDVIEQYSQGHLNLMVRIKELQRRLDQSIGKPSLFISVSEKSKDRGSNTIGARLNRVEDKVTQLDQRLALITDMLHQLLSLHGGSTPGSGGPPREGGAHITQPCGSGGSVDPELFLPSNTLPTYEQLTVPRRGPDEGS

>sp|P56696.1|KCNQ4_HUMAN RecName: Full=Potassium voltage-gated channel subfamily KQT member 4; AltName: Full=Voltage-gated potassium channel subunit Kv7.4; AltName: Full=Potassium channel subunit alpha KvLQT4; AltName: Full=KQT-like 4

MAEAPPRRLGLGPPPGDAPRAELVALTAVQSEQGEAGGGGSPRRLGLLGSPLPPGAPLPGPGSGSGSACGQRSSAAHKRYRRLQNWVYNVLERPRGWAFVYHVFIFLLVFSCLVLSVLSTIQEHQELANECLLILEFVMIVVFGLEYIVRVWSAGCCCRYRGWQGRFRFARKPFCVIDFIVFVASVAVIAAGTQGNIFATSALRSMRFLQILRMVRMDRRGGTWKLLGSVVYAHSKELITAWYIGFLVLIFASFLVYLAEKDANSDFSSYADSLWWGTITLTTIGYGDKTPHTWLGRVLAAGFALLGISFFALPAGILGSGFALKVQEQHRQKHFEKRRMPAANLIQAAWRLYSTDMSRAYLTATWYYYDSILPSFRELALLFEHVQRARNGGLRPLEVRRAPVPDGAPSRYPPVATCHRPGSTSFCPGESSRMGIKDRIRMGSSQRRTGPSKQQLAPPTMPTSPSSEQVGEATSPTKVQKSWSFNDRTRFRASLRLKPRTSAEDAPSEEVAEEKSYQCELTVDDIMPAVKTVIRSIRILKFLVAKRKFKETLRPYDVKDVIEQYSAGHLDMLGRIKSLQTRVDQIVGRGPGDRKAREKGDKGPSDAEVVDEISMMGRVVKVEKQVQSIEHKLDLLLGFYSRCLRSGTSASLGAVQVPLFDPDITSDYHSPVDHEDISVSAQTLSISRSVSTNMD

>BAA88711.1 sister p-glycoprotein, partial [Homo sapiens]

KQRIAIARAIVRDPKILLLDEATSALDTESEKTVQVALDKAREGRTCIVIAHRLSTIQNADII

>sp|Q9Y6J6.1|KCNE2_HUMAN RecName: Full=Potassium voltage-gated channel subfamily E member 2; AltName: Full=MinK-related peptide 1; AltName: Full=Minimum potassium ion channel-related peptide 1; AltName: Full=Potassium channel subunit beta MiRP1

MSTLSNFTQTLEDVFRRIFITYMDNWRQNTTAEQEALQAKVDAENFYYVILYLMVMIGMFSFIIVAILVSTVKSKRREHSNDPYHQYIVEDWQEKYKSQILNLEESKATIHENIGAAGFKMSP

>pdb|1BYW|A Chain A, PROTEIN (HUMAN ERG POTASSIUM CHANNEL)

SRKFIIANARVENCAVIYCNDGFCELCGYSRAEVMQRPCTCDFLHGPCTQRRAAAQIAQALLGAEERKVEIAFYRKDGSCFLCLVDVVPVKNEDGAVIMFILNFEVVMEK

>AAF61479.1 dopamine receptor D2longer [Homo sapiens]

MDPLNLSWYDDDLERQNWSRPFNGSDGKADRPHYNYYATLLTLLIAVIVFGNVLVCMAVSREKALQTTTNYLIVSLAVADLLVATLVMPWVVYLEVVGEWKFSRIHCDIFVTLDVMMCTASILNLCAISIDRYTAVAMPMLYNTRYSSKRRVTVMISIVWVLSFTISCPLLFGLNNADQNECIIANPAFVVYSSIVSFYVPFIVTLLVYIKIYIVLRRRRKRVNTKRSSRAFRAHLRAPLKGNCTHPEDMKLCTVIMKSNGSFPVNRRRVVQEAARRAQELEMEMLSSTSPPERTRYSPIPPSHHQLTLPDPSHHGLHSTPDSPAKPEKNGHAKDHPKIAKIFEIQTMPNGKTRTSLKTMSRRKLSQQKEKKATQMLAIVLGVFIICWLPFFITHILNIHCDCNIPPVLYSAFTWLGYVNSAVNPIIYTTFNIEFRKAFLKILHC

>AAB31163.2 alpha adrenergic receptor subtype alpha 1a [Homo sapiens]

MTFRDLLSVSFEGPRPDSSAGGSSAGGGGGGAGGAAPSEGPAVGGVPGGAGGGGGVVGAGSGEDNRSSAGEPGSAGAGGDVNGTAAVGGLVVSAQGVGVGVFLAAFILMAVAGNLLVILSVACNRHLQTVTNYFIVNLAVADLLLSATVLPFSATMEVLGFWAFGRAFCDVWAAVDVLCCTASILSLCTISVDRYVGVRHSLKYPAIMTERKAAAILALLWVVALVVSVGPLLGWKEPVPPDERFCGITEEAGYAVFSSVCSFYLPMAVIVVMYCRVYVVARSTTRSLEAGVKRERGKASEVVLRIHCRGAATGADGAHGMRSAKGHTFRSSLSVRLLKFSREKKAAKTLAIVVGVFVLCWFPFFFVLPLGSLFPQLKPSEGVFKVIFWLGYFNSCVNPLIYPCSSREFKRAFLRLLRCQCRRRRRRRPLWRVYGHHWRASTSGLRQDCAPSSGDAPPGAPLALTALPDPDPEPPGTPEMQAPVASRRKPPSAFREWRLLGPFRRPTTQLRAKVSSLSHKIPAGGAQRAEAACAQRSEVEAVSLGVPHEVAEGATCQAYELADYSNLRETDI

>NP_060182.1 pyroglutamyl-peptidase 1 isoform 1 [Homo sapiens]

MEQPRKAVVVTGFGPFGEHTVNASWIAVQELEKLGLGDSVDLHVYEIPVEYQTVQRLIPALWEKHSPQLVVHVGVSGMATTVTLEKCGHNKGYKGLDNCRFCPGSQCCVEDGPESIDSIIDMDAVCKRVTTLGLDVSVTISQDAGRYLCDFTYYTSLYQSHGRSAFVHVPPLGKPYNADQLGRALRAIIEEMLDLLEQSEGKINYCHKH

>NP_005680.1 ATP-binding cassette sub-family B member 6, mitochondrial isoform 1 [Homo sapiens]

MVTVGNYCEAEGPVGPAWMQDGLSPCFFFTLVPSTRMALGTLALVLALPCRRRERPAGADSLSWGAGPRISPYVLQLLLATLQAALPLAGLAGRVGTARGAPLPSYLLLASVLESLAGACGLWLLVVERSQARQRLAMGIWIKFRHSPGLLLLWTVAFAAENLALVSWNSPQWWWARADLGQQVQFSLWVLRYVVSGGLFVLGLWAPGLRPQSYTLQVHEEDQDVERSQVRSAAQQSTWRDFGRKLRLLSGYLWPRGSPALQLVVLICLGLMGLERALNVLVPIFYRNIVNLLTEKAPWNSLAWTVTSYVFLKFLQGGGTGSTGFVSNLRTFLWIRVQQFTSRRVELLIFSHLHELSLRWHLGRRTGEVLRIADRGTSSVTGLLSYLVFNVIPTLADIIIGIIYFSMFFNAWFGLIVFLCMSLYLTLTIVVTEWRTKFRRAMNTQENATRARAVDSLLNFETVKYYNAESYEVERYREAIIKYQGLEWKSSASLVLLNQTQNLVIGLGLLAGSLLCAYFVTEQKLQVGDYVLFGTYIIQLYMPLNWFGTYYRMIQTNFIDMENMFDLLKEETEVKDLPGAGPLRFQKGRIEFENVHFSYADGRETLQDVSFTVMPGQTLALVGPSGAGKSTILRLLFRFYDISSGCIRIDGQDISQVTQASLRSHIGVVPQDTVLFNDTIADNIRYGRVTAGNDEVEAAAQAAGIHDAIMAFPEGYRTQVGERGLKLSGGEKQRVAIARTILKAPGIILLDEATSALDTSNERAIQASLAKVCANRTTIVVAHRLSTVVNADQILVIKDGCIVERGRHEALLSRGGVYADMWQLQQGQEETSEDTKPQTMER

>NP_061338.1 phosphatidylcholine translocator ABCB4 isoform C [Homo sapiens]

MDLEAAKNGTAWRPTSAEGDFELGISSKQKRKKTKTVKMIGVLTLFRYSDWQDKLFMSLGTIMAIAHGSGLPLMMIVFGEMTDKFVDTAGNFSFPVNFSLSLLNPGKILEEEMTRYAYYYSGLGAGVLVAAYIQVSFWTLAAGRQIRKIRQKFFHAILRQEIGWFDINDTTELNTRLTDDISKISEGIGDKVGMFFQAVATFFAGFIVGFIRGWKLTLVIMAISPILGLSAAVWAKILSAFSDKELAAYAKAGAVAEEALGAIRTVIAFGGQNKELERYQKHLENAKEIGIKKAISANISMGIAFLLIYASYALAFWYGSTLVISKEYTIGNAMTVFFSILIGAFSVGQAAPCIDAFANARGAAYVIFDIIDNNPKIDSFSERGHKPDSIKGNLEFNDVHFSYPSRANVKILKGLNLKVQSGQTVALVGSSGCGKSTTVQLIQRLYDPDEGTINIDGQDIRNFNVNYLREIIGVVSQEPVLFSTTIAENICYGRGNVTMDEIKKAVKEANAYEFIMKLPQKFDTLVGERGAQLSGGQKQRIAIARALVRNPKILLLDEATSALDTESEAEVQAALDKAREGRTTIVIAHRLSTVRNADVIAGFEDGVIVEQGSHSELMKKEGVYFKLVNMQTSGSQIQSEEFELNDEKAATRMAPNGWKSRLFRHSTQKNLKNSQMCQKSLDVETDGLEANVPPVSFLKVLKLNKTEWPYFVVGTVCAIANGGLQPAFSVIFSEIIAIFGPGDDAVKQQKCNIFSLIFLFLGIISFFTFFLQGFTFGKAGEILTRRLRSMAFKAMLRQDMSWFDDHKNSTGALSTRLATDAAQVQGATGTRLALIAQNIANLGTGIIISFIYGWQLTLLLLAVVPIIAVSGIVEMKLLAGNAKRDKKELEAAGKIATEAIENIRTVVSLTQERKFESMYVEKLYGPYRVFSAIVFGAVALGHASSFAPDYAKAKLSAAHLFMLFERQPLIDSYSEEGLKPDKFEGNITFNEVVFNYPTRANVPVLQGLSLEVKKGQTLALVGSSGCGKSTVVQLLERFYDPLAGTVLLDGQEAKKLNVQWLRAQLGIVSQEPILFDCSIAENIAYGDNSRVVSQDEIVSAAKAANIHPFIETLPHKYETRVGDKGTQLSGGQKQRIAIARALIRQPQILLLDEATSALDTESEKVVQEALDKAREGRTCIVIAHRLSTIQNADLIVVFQNGRVKEHGTHQQLLAQKGIYFSMVSVQAGTQNL

>AAG23345.1 PAR2, partial [Homo sapiens]

MTVTRTHHF

>AAG33617.1 ATP-binding cassette half-transporter [Homo sapiens]

MAWSTCVPSRDPRSLAPSGYVSQGTRMSCAPLRVTSPGLRSRSARSLGRRPRIAMVTVGNYCEAEGPVGPAWMQDGLSPCFFFTLVPSTRMALGTLALVLALPCRRRERPAGADSLSWGAGPRISPYVLQLLLATLQAALPLAGLAGRVGTARGAPLPSYLLLASVLESLAGACGLWLLVVERSQARQRLAMGIWIKFRHSPGLLLLWTVAFAAENLALVSWNSPQWWWARADLGQQVQFSLWVLRYVVSGGLFVLGLWAPGLRPQSYTLQVHEEDQDVERSQVRSAAQQSTWRDFGRKLRLLSGYLWPRGSPALQLVVLICLGLMGLERALNVLVPIFYRNIVNLLTEKAPWNSLAWTVTSYVFLKFLQGGGTGSTGFVSNLRTFLWIRVQQFTSRRVELLIFSHLHELSLRWHLGRRTGEVLRIADRGTSSVTGLLSYLVFNVIPTLADIIIGIIYFSMFFNAWFGLIVFLCMSLYLTLTIVVTEWRTKFRRAMNTQENATRARAVDSLLNFETVKYYNAESYEVERYREAIIKYQGLEWKSSASLVLLNQTQNLVIGLGLLAGSLLCAYFVTEQKLQVGDYVLFGTYIIQLYMPLNWFGTYYRMIQTNFIDMENMFDLLKEETEVKDLPGAGPLRFQKGRIEFENVHFSYADGRETLQDVSFTVMPGQTLALVGPSGAGKSTILRLLFRFYDISSGCIRIDGQDISQVTQASLRSHIGVVPQDTVLFNDTIADNIRYGRVTAGNDEVEAAAQAAGIHDAIMAFPEGYRTQVGERGLKLSGGEKQRVAIARTILKAPGIILLDEATSALDTSNERAIQASLAKVCANRTTIVVAHRLSTVVNADQILVIKDGCIVERGRHEALLSRGGVYADMWQLQQGQEETSEDTKPQTMER

>AAG33618.1 ATP-binding cassette half-transporter, partial [Homo sapiens]

MAWSTCVPSRDPRSLAPSGYVSQGTRMSCAPLRVTSPGLRSRSARSLGRRPRIAMVTVGNYCEAEGPVGPAWMQDGLSPCFFFTLVPSTRMALGTLALVLALPCRRRERPAGADSLSWGAGPRISPYVLQLLLATLQAALPLAGLAGRVGTARGAPLPSYLLLASVLESLAGACGLWLLVVERSQARQRLAMGIWIKFRHSPGLLLLWTVAFAAENLALVSWNNPQWWWARADLGQQVQFSLWVLRYVVSGGLFVLGLWAPGLRPQSYTLQVHEEDQDVERSQ

>NP_071395.1 p53-regulated apoptosis-inducing protein 1 [Homo sapiens]

MGSSSEASFRSAQASCSGARRQGLGRGDQNLSVMPPNGRAQTHTPGWVSPCSENRDGLLPATAPGRLCSHRGADIPSFQTHQDPVTASGSSELHADCPQFRALDRAGN

>NP_071285.1 nuclear receptor subfamily 1 group I member 2 isoform 2 [Homo sapiens]

MTVTRTHHFKEGSLRAPAIPLHSAAAELASNHPRGPEANLEVRPKESWNHADFVHCEDTESVPGKPSVNADEEVGGPQICRVCGDKATGYHFNVMTCEGCKGFFRRAMKRNARLRCPFRKGACEITRKTRRQCQACRLRKCLESGMKKEMIMSDEAVEERRALIKRKKSERTGTQPLGVQGLTEEQRMMIRELMDAQMKTFDTTFSHFKNFRLPGVLSSGCELPESLQAPSREEAAKWSQVRKDLCSLKVSLQLRGEDGSVWNYKPPADSGGKEIFSLLPHMADMSTYMFKGIISFAKVISYFRDLPIEDQISLLKGAAFELCQLRFNTVFNAETGTWECGRLSYCLEDTAGGFQQLLLEPMLKFHYMLKKLQLHEEEYVLMQAISLFSPDRPGVLQHRVVDQLQEQFAITLKSYIECNRPQPAHRFLFLKIMAMLTELRSINAQHTQRLLRIQDIHPFATPLMQELFGITGS

>BAB19682.1 HERG-USO [Homo sapiens]

MPVRRGHVAPQNTFLDTIIRKFEGQSRKFIIANARVENCAVIYCNDGFCELCGYSRAEVMQRPCTCDFLHGPRTQRRAAAQIAQALLGAEERKVEIAFYRKDGSCFLCLVDVVPVKNEDGAVIMFILNFEVVMEKDMVGSPAHDTNHRGPPTSWLAPGRAKTFRLKLPALLALTARESSVRSGGAGGAGAPGAVVVDVDLTPAAPSSESLALDEVTAMDNHVAGLGPAEERRALVGPGSPPRSAPGQLPSPRAHSLNPDASGSSCSLARTRSRESCASVRRASSADDIEAMRAGVLPPPPRHASTGAMHPLRSGLLNSTSDSDLVRYRTISKIPQITLNFVDLKGDPFLASPTSDREIIAPKIKERTHNVTEKVTQVLSLGADVLPEYKLQAPRIHRWTILHYSPFKAVWDWLILLLVIYTAVFTPYSAAFLLKETEEGPPATECGYACQPLAVVDLIVDIMFIVDILINFRTTYVNANEEVVSHPGRIAVHYFKGWFLIDMVAAIPFDLLIFGSGSEELIGLLKTARLLRLVRVARKLDRYSXYGAAVLFLLMCTFALIAHWLACIWYAIGNMEQPHMDSRIGWLHNLGDQIGKPYNSSGLGGPSIKDKYVTALYFTFSSLTSVGFGNVSPNTNSEKIFSICVMLIGSLMYASIFGNVSAIIQRLYSGTARYHTQMLRVREFIRFHQIPNPLRQRLEEYFQHAWSYTNGIDMNAVLKGFPECLQADICLHLNRSLLQHCKPFRGATKGCLRALAMKFKTTHAPPGDTLVHAGDLLTALYFISRGSIEILRGDVVVAILGMGWGAGTGLEMPSAASRGASLLNMQSLGLWTWDCLQGHWAPLIHLNSGPPSGAMERSPTWGEAAELWGSHILLPFRIRHKQTLFASLK

>NP_068712.1 gamma-aminobutyric acid receptor subunit beta-3 isoform 2 precursor [Homo sapiens]

MCSGLLELLLPIWLSWTLGTRGSEPRSVNDPGNMSFVKETVDKLLKGYDIRLRPDFGGPPVCVGMNIDIASIDMVSEVNMDYTLTMYFQQYWRDKRLAYSGIPLNLTLDNRVADQLWVPDTYFLNDKKSFVHGVTVKNRMIRLHPDGTVLYGLRITTTAACMMDLRRYPLDEQNCTLEIESYGYTTDDIEFYWRGGDKAVTGVERIELPQFSIVEHRLVSRNVVFATGAYPRLSLSFRLKRNIGYFILQTYMPSILITILSWVSFWINYDASAARVALGITTVLTMTTINTHLRETLPKIPYVKAIDMYLMGCFVFVFLALLEYAFVNYIFFGRGPQRQKKLAEKTAKAKNDRSKSESNRVDAHGNILLTSLEVHNEMNEVSGGIGDTRNSAISFDNSGIQYRKQSMPREGHGRFLGDRSLPHKKTHLRRRSSQLKIKIPDLTDVNAIDRWSRIVFPFTFSLFNLVYWLYYVN

>NP_076917.1 5-hydroxytryptamine receptor 5A [Homo sapiens]

MDLPVNLTSFSLSTPSPLETNHSLGKDDLRPSSPLLSVFGVLILTLLGFLVAATFAWNLLVLATILRVRTFHRVPHNLVASMAVSDVLVAALVMPLSLVHELSGRRWQLGRRLCQLWIACDVLCCTASIWNVTAIALDRYWSITRHMEYTLRTRKCVSNVMIALTWALSAVISLAPLLFGWGETYSEGSEECQVSREPSYAVFSTVGAFYLPLCVVLFVYWKIYKAAKFRVGSRKTNSVSPISEAVEVKDSAKQPQMVFTVRHATVTFQPEGDTWREQKEQRAALMVGILIGVFVLCWIPFFLTELISPLCSCDIPAIWKSIFLWLGYSNSFFNPLIYTAFNKNYNSAFKNFFSRQH

>AAK38720.1 orphan nuclear receptor PXR.1 [Homo sapiens]

MEVRPKESWNHADFVHCEDTESVPGKPSVNADEEVGGPQICRVCGDKATGYHFNVMTCEGCKGFFRRAMKRNARLRCPFRKGACEITRKTRRQCQACRLRKCLESGMKKEMIMSDEAVEERRALIKRKKSERTGTQPLGVQGLTEEQRMMIRELMDAQMKTFDTTFSHFKNFRLPGVLSSGCELPESLQAPSREEAAKWSQVRKDLCSLKVSLQLRGEDGSVWNYKPPADSGGKEIFSLLPHMADMSTYMFKGIISFAKVISYFRDLPIEDQISLLKGAAFELCQLRFNTVFNAETGTWECGRLSYCLEDTAGGFQQLLLEPMLKFHYMLKKLQLHEEEYVLMQAISLFSPDRPGVLQHRVVDQLQEQFAITLKSYIECNRPQPAHRFLFLKIMAMLTELRSINAQHTQRLLRIQDIHPFATPLMQELFGITGS

>sp|O43526.2|KCNQ2_HUMAN RecName: Full=Potassium voltage-gated channel subfamily KQT member 2; AltName: Full=KQT-like 2; AltName: Full=Neuroblastoma-specific potassium channel subunit alpha KvLQT2; AltName: Full=Voltage-gated potassium channel subunit Kv7.2

MVQKSRNGGVYPGPSGEKKLKVGFVGLDPGAPDSTRDGALLIAGSEAPKRGSILSKPRAGGAGAGKPPKRNAFYRKLQNFLYNVLERPRGWAFIYHAYVFLLVFSCLVLSVFSTIKEYEKSSEGALYILEIVTIVVFGVEYFVRIWAAGCCCRYRGWRGRLKFARKPFCVIDIMVLIASIAVLAAGSQGNVFATSALRSLRFLQILRMIRMDRRGGTWKLLGSVVYAHSKELVTAWYIGFLCLILASFLVYLAEKGENDHFDTYADALWWGLITLTTIGYGDKYPQTWNGRLLAATFTLIGVSFFALPAGILGSGFALKVQEQHRQKHFEKRRNPAAGLIQSAWRFYATNLSRTDLHSTWQYYERTVTVPMYSSQTQTYGASRLIPPLNQLELLRNLKSKSGLAFRKDPPPEPSPSKGSPCRGPLCGCCPGRSSQKVSLKDRVFSSPRGVAAKGKGSPQAQTVRRSPSADQSLEDSPSKVPKSWSFGDRSRARQAFRIKGAASRQNSEEASLPGEDIVDDKSCPCEFVTEDLTPGLKVSIRAVCVMRFLVSKRKFKESLRPYDVMDVIEQYSAGHLDMLSRIKSLQSRVDQIVGRGPAITDKDRTKGPAEAELPEDPSMMGRLGKVEKQVLSMEKKLDFLVNIYMQRMGIPPTETEAYFGAKEPEPAPPYHSPEDSREHVDRHGCIVKIVRSSSSTGQKNFSAPPAAPPVQCPPSTSWQPQSHPRQGHGTSPVGDHGSLVRIPPPPAHERSLSAYGGGNRASMEFLRQEDTPGCRPPEGNLRDSDTSISIPSVDHEELERSFSGFSISQSKENLDALNSCYAAVAPCAKVRPYIAEGESDTDSDLCTPCGPPPRSATGEGPFGDVGWAGPRK

>AAK68112.1 m1 muscarinic cholinergic receptor [Homo sapiens]

MNTSAPPAVSPNITVLAPGKGPWQVAFIGITTGLLSLATVTGNLLVLISFKVNTELKTVNNYFLLSLACADLIIGTFSMNLYTTYLLMGHWALGTLACDLWLALDYVASNASVMNLLLISFDRYFSVTRPLSYRAKRTPRRAALMIGLAWLVSFVLWAPAILFWQYLVGERTVLAGQCYIQFLSQPIITFGTAMAAFYLPVTVMCTLYWRIYRETENRARELAALQGSETPGKGGGSSSSSERSQPGAEGSPETPPGRCCRCCRAPRLLQAYSWKEEEEEDEGSMESLTSSEGEEPGSEVVIKMPMVDPEAQAPTKQPPRSSPNTVKRPTKKGRDRAGKGQKPRGKEQLAKRKTFSLVKEKKAARTLSAILLAFILTWTPYNIMVLVSTFCKDCVPETLWELGYWLCYVNSTINPMCYALCNKAFRDTFRLLLLCRWDKRRWRKIPKRPGSVHR

>NP_148934.1 nuclear receptor subfamily 1 group I member 2 isoform 3 [Homo sapiens]

MEVRPKESWNHADFVHCEDTESVPGKPSVNADEEVGGPQICRVCGDKATGYHFNVMTCEGCKGFFRRAMKRNARLRCPFRKGACEITRKTRRQCQACRLRKCLESGMKKEMIMSDEAVEERRALIKRKKSERTGTQPLGVQGLTEEQRMMIRELMDAQMKTFDTTFSHFKNFRVSLQLRGEDGSVWNYKPPADSGGKEIFSLLPHMADMSTYMFKGIISFAKVISYFRDLPIEDQISLLKGAAFELCQLRFNTVFNAETGTWECGRLSYCLEDTAGGFQQLLLEPMLKFHYMLKKLQLHEEEYVLMQAISLFSPDRPGVLQHRVVDQLQEQFAITLKSYIECNRPQPAHRFLFLKIMAMLTELRSINAQHTQRLLRIQDIHPFATPLMQELFGITGS

>pdb|1ILH|A Chain A, Orphan Nuclear Receptor Pxr

MKKGHHHHHHGSERTGTQPLGVQGLTEEQRMMIRELMDAQMKTFDTTFSHFKNFRLPGVLSSGCELPESLQAPSREEAAKWSQVRKDLCSLKVSLQLRGEDGSVWNYKPPADSGGKEIFSLLPHMADMSTYMFKGIISFAKVISYFRDLPIEDQISLLKGAAFELCQLRFNTVFNAETGTWECGRLSYCLEDTAGGFQQLLLEPMLKFHYMLKKLQLHEEEYVLMQAISLFSPDRPGVLQHRVVDQLQEQFAITLKSYIECNRPQPAHRFLFLKIMAMLTELRSINAQHTQRLLRIQDIHPFATPLMQELFGITGS

>AAK77197.1 adrenergic receptor alpha-1a [Homo sapiens]

MVFLSGNASDSSNCTQPPAPVNISKAILLGVILGGLILFGVLGNILVILSVACHRHLHSVTHYYIVNLAVADLLLTSTVLPFSAIFEVLGYWAFGRVFCNIWAAVDVLCCTASIMGLCIISIDRYIGVSHPLRYPTIVTQRRGLMALLCVWALSLVISIGPLFGWRQPAPEDETICQINEEPGYVLFSALGSFYLPLAIILVMYCRVYVVAKRESRGLKSGLKTDKSDSEQVTLRIHRKNAPAGGSGMASAKTKTHFSVRLLKFSREKKAAKTLGIVVGCFVLCWLPFFLVMPIGSFFPDFKPSETVFKIVFWLGYLNSCINPIIYPCSSQEFKKAFQNVLRIQCLCRKQSSKHALGYTLHPPSQAVEGQHKDMVRIPVGSREAFYGISRTDGVCEWKFFSSMPRGSARITVSKDQSSCTTARVRSKSFLQVCCCVEPSTPSLDKNHQVPTIKVHTISLSENGEEV

>NP_055101.2 neuronal calcium sensor 1 isoform 1 [Homo sapiens]

MGKSNSKLKPEVVEELTRKTYFTEKEVQQWYKGFIKDCPSGQLDAAGFQKIYKQFFPFGDPTKFATFVFNVFDENKDGRIEFSEFIQALSVTSRGTLDEKLRWAFKLYDLDNDGYITRNEMLDIVDAIYQMVGNTVELPEEENTPEKRVDRIFAMMDKNADGKLTLQEFQEGSKADPSIVQALSLYDGLV

>NP_057658.2 D(2) dopamine receptor isoform short [Homo sapiens]

MDPLNLSWYDDDLERQNWSRPFNGSDGKADRPHYNYYATLLTLLIAVIVFGNVLVCMAVSREKALQTTTNYLIVSLAVADLLVATLVMPWVVYLEVVGEWKFSRIHCDIFVTLDVMMCTASILNLCAISIDRYTAVAMPMLYNTRYSSKRRVTVMISIVWVLSFTISCPLLFGLNNADQNECIIANPAFVVYSSIVSFYVPFIVTLLVYIKIYIVLRRRRKRVNTKRSSRAFRAHLRAPLKEAARRAQELEMEMLSSTSPPERTRYSPIPPSHHQLTLPDPSHHGLHSTPDSPAKPEKNGHAKDHPKIAKIFEIQTMPNGKTRTSLKTMSRRKLSQQKEKKATQMLAIVLGVFIICWLPFFITHILNIHCDCNIPPVLYSAFTWLGYVNSAVNPIIYTTFNIEFRKAFLKILHC

>CAD28599.1 unnamed protein product [Homo sapiens]

MDLEGDRNGGAKKKNFFKLNNKSEKDKKEKKPTVSVFSMFRYSNWLDKLYMVVGTLAAIIHGAGLPLMMLVFGEMTDIFANAGNLEDLMSNITNRSDINDTGFFMNLEEDMTRYAYYYSGIGAGVLVAAYIQVSFWCLAAGRQIHKIRKQFFHAIMRQEIGWFDVHDVGELNTRLTDDVSKINEGIGDKIGMFFQSMATFFTGFIVGFTRGWKLTLVILAISPVLGLSAAVWAKILSSFTDKELLAYAKAGAVAEEVLAAIRTVIAFGGQKKELERYNKNLEEAKRIGIKKAITANISIGAAFLLIYASYALAFWYGTTLVLSGEYSIGQVLTVFFSVLIGAFSVGQASPSIEAFANARGAAYEIFKIIDNKPSIDSYSKSGHKPDNIKGNLEFRNVHFSYPSRKEVKILKGLNLKVQSGQTVALVGNSGCGKSTTVQLMQRLYDPTEGMVSVDGQDIRTINVRFLREIIGVVSQEPVLFATTIAENIRYGRENVTMDEIEKAVKEANAYDFIMKLPHKFDTLVGERGAQLSGGQKQRIAIARALVRNPKILLLDEATSALDTESEAVVQVALDKARKGRTTIVIAHRLSTVRNADVIAGFDDGVIVEKGNHDELMKEKGIYFKLVTMQTAGNEVELENAADESKSEIDALEMSSNDSRSSLIRKRSTRRSVRGSQAQDRKLSTKEALDESIPPVSFWRIMKLNLTEWPYFVVGVFCAIINGGLQPAFAIIFSKIIGVFTRIDDPETKRQNSNLFSLLFLALGIISFITFFLQGFTFGKAGEILTKRLRYMVFRSMLRQDVSWFDDPKNTTGALTTRLANDAAQVKGAIGSRLAVITQNIANLGTGIIISFIYGWQLTLLLLAIVPIIAIAGVVEMKMLSGQALKDKKELEGAGKIATEAIENFRTVVSLTQEQKFEHMYAQSLQVPYRNSLRKAHIFGITFSFTQAMMYFSYAGCFRFGAYLVAHKLMSFEDVLLVFSAVVFGAMAVGQVSSFAPDYAKAKISAAHIIMIIEKTPLIDSYSTEGLMPNTLEGNVTFGEVVFNYPTRPDIPVLQGLSLEVKKGQTLALVGSSGCGKSTVVQLLERFYDPLAGKVLLDGKEIKRLNVQWLRAHLGIVSQEPILFDCSIAENIAYGDNSRVVSQEEIVRAAKEANIHAFIESLPNKYSTKVGDKGTQLSGGQKQRIAIARALVRQPHILLLDEATSALDTESEKVVQEALDKAREGRTCIVIAHRLSTIQNADLIVVFQNGRVKEHGTHQQLLAQKGIYFSMVSVQAGTKRQ

>NP_005066.2 organic anion transporting polypeptide A isoform b [Homo sapiens]

MFLTKNCKQEQERVESAKMFLLAITCAFVSKTLSGSYMNSMLTQIERQFNIPTSLVGFINGSFEIGNLLL

IIFVSYFGTKLHRPIMIGIGCVVMGLGCFLKSLPHFLMNQYEYESTVSVSGNLSSNSFLCMENGTQILRPTQDPSECTKEVKSLMWVYVLVGNIVRGMGETPILPLGISYIEDFAKFENSPLYIGLVETGAIIGPLIGLLLASFCANVYVDTGFVNTDDLIITPTDTRWVGAWWFGFLICAGVNVLTAIPFFFLPNTLPKEGLETNADIIKNENEDKQKEEVKKEKYGITKDFLPFMKSLSCNPIYMLFILVSVIQFNAFVNMISFMPKYLEQQYGISSSDAIFLMGIYNLPPICIGYIIGGLIMKKFKITVKQAAHIGCWLSLLEYLLYFLSFLMTCENSSVVGINTSYEGIPQDLYVENDIFADCNVDCNCPSKIWDPVCGNNGLSYLSACLAGCETSIGTGINMVFQNCSCIQTSGNSSAVLGLCDKGPDCSLMLQYFLILSAMSSFIYSLAAIPGYMVLLRCMKSEEKSLGVGLHTFCTRVFGKNSYFFPHPIRTILLLLFFITL

>NP_602307.1 solute carrier organic anion transporter family member 1A2 isoform 1 [Homo sapiens]

MGETEKRIETHRIRCLSKLKMFLLAITCAFVSKTLSGSYMNSMLTQIERQFNIPTSLVGFINGSFEIGNLLLIIFVSYFGTKLHRPIMIGIGCVVMGLGCFLKSLPHFLMNQYEYESTVSVSGNLSSNSFLCMENGTQILRPTQDPSECTKEVKSLMWVYVLVGNIVRGMGETPILPLGISYIEDFAKFENSPLYIGLVETGAIIGPLIGLLLASFCANVYVDTGFVNTDDLIITPTDTRWVGAWWFGFLICAGVNVLTAIPFFFLPNTLPKEGLETNADIIKNENEDKQKEEVKKEKYGITKDFLPFMKSLSCNPIYMLFILVSVIQFNAFVNMISFMPKYLEQQYGISSSDAIFLMGIYNLPPICIGYIIGGLIMKKFKITVKQAAHIGCWLSLLEYLLYFLSFLMTCENSSVVGINTSYEGIPQDLYVENDIFADCNVDCNCPSKIWDPVCGNNGLSYLSACLAGCETSIGTGINMVFQNCSCIQTSGNSSAVLGLCDKGPDCSLMLQYFLILSAMSSFIYSLAAIPGYMVLLRCMKSEEKSLGVGLHTFCTRVFAGIPAPIYFGALMDSTCLHWGTLKCGESGACRIYDSTTFRYIYLGLPAALRGSSFVPALIILILLRKCHLPGENASSGTELIETKVKGKENECKDIYQKSTVLKDDELKTKL

>NP_005106.2 major vault protein isoform 1 [Homo sapiens]

MATEEFIIRIPPYHYIHVLDQNSNVSRVEVGPKTYIRQDNERVLFAPMRMVTVPPRHYCTVANPVSRDAQGLVLFDVTGQVRLRHADLEIRLAQDPFPLYPGEVLEKDITPLQVVLPNTALHLKALLDFEDKDGDKVVAGDEWLFEGPGTYIPRKEVEVVEIIQATIIRQNQALRLRARKECWDRDGKERVTGEEWLVTTVGAYLPAVFEEVLDLVDAVILTEKTALHLRARRNFRDFRGVSRRTGEEWLVTVQDTEAHVPDVHEEVLGVVPITTLGPHNYCVILDPVGPDGKNQLGQKRVVKGEKSFFLQPGEQLEQGIQDVYVLSEQQGLLLRALQPLEEGEDEEKVSHQAGDHWLIRGPLEYVPSAKVEVVEERQAIPLDENEGIYVQDVKTGKVRAVIGSTYMLTQDEVLWEKELPPGVEELLNKGQDPLADRGEKDTAKSLQPLAPRNKTRVVSYRVPHNAAVQVYDYREKRARVVFGPELVSLGPEEQFTVLSLSAGRPKRPHARRALCLLLGPDFFTDVITIETADHARLQLQLAYNWHFEVNDRKDPQETAKLFSVPDFVGDACKAIASRVRGAVASVTFDDFHKNSARIIRTAVFGFETSEAKGPDGMALPRPRDQAVFPQNGLVVSSVDVQSVEPVDQRTRDALQRSVQLAIEITTNSQEAAAKHEAQRLEQEARGRLERQKILDQSEAEKARKELLELEALSMAVESTGTAKAEAESRAEAARIEGEGSVLQAKLKAQALAIETEAELQRVQKVRELELVYARAQLELEVSKAQQLAEVEVKKFKQMTEAIGPSTIRDLAVAGPEMQVKLLQSLGLKSTLITDGSTPINLFNTAFGLLGMGPEGQPLGRRVASGPSPGEGISPQSAQAPQAPGDNHVVPVLR

>NP_004153.2 ras-related protein Rab-5A isoform 1 [Homo sapiens]

MASRGATRPNGPNTGNKICQFKLVLLGESAVGKSSLVLRFVKGQFHEFQESTIGAAFLTQTVCLDDTTVKFEIWDTAGQERYHSLAPMYYRGAQAAIVVYDITNEESFARAKNWVKELQRQASPNIVIALSGNKADLANKRAVDFQEAQSYADDNSLLFMETSAKTSMNVNEIFMAIAKKLPKNEPQNPGANSARGRGVDLTEPTQPTRNQCCSN

>NP_006158.2 homeobox protein Nkx-3.1 isoform 1 [Homo sapiens]

MLRVPEPRPGEAKAEGAAPPTPSKPLTSFLIQDILRDGAQRQGGRTSSQRQRDPEPEPEPEPEGGRSRAGAQNDQLSTGPRAAPEEAETLAETEPERHLGSYLLDSENTSGALPRLPQTPKQPQKRSRAAFSHTQVIELERKFSHQKYLSAPERAHLAKNLKLTETQVKIWFQNRRYKTKRKQLSSELGDLEKHSSLPALKEEAFSRASLVSVYNSYPYYPYLYCVGSWSPAFW

>BAB91222.1 muscarinic acetylcholine receptor M5 [Homo sapiens]

MEGDSYHNATTVNGTPVNHQPLERHRLWEVITIAAVTAVVSLITIVGNVLVMISFKVNSQLKTVNNYYLLSLACADLIIGIFSMNLYTTYILMGRWALGSLACDLWLALDYVASNASVMNLLVISFDRYFSITRPLTYRAKRTPKRAGIMIGLAWLISFILWAPAILCWQYLVGKRTVPLDECQIQFLSEPTITFGTAIAAFYIPVSVMTILYCRIYRETEKRTKDLADLQGSDSVTKAEKRKPAHRALFRSCLRCPRPTLAQRERNQASWSSSRRSTSTTGKPSQATGPSANWAKAEQLTTCSSYPSSEDEDKPATDPVLQVVYKSQGKESPGEEFSAEETEETFVKAETEKSDYDTPNYLLSPAAAHRPKSQKCVAYKFRLVVKADGNQETNNGCHKVKIMPCPFPVAKEPSTKGLNPNPSHQMTKRKRVVLVKERKAAQTLSAILLAFIITWTPYNIMVLVSTFCDKCVPVTLWHLGYWLCYVNSTVNPICYALCNRTFRKTFKMLLLCRWKKKKVEEKLYWQGNSKLP

>NP_066919.2 voltage-dependent T-type calcium channel subunit alpha-1I isoform a [Homo sapiens]

MAESASPPSSSAAAPAAEPGVTTEQPGPRSPPSSPPGLEEPLDGADPHVPHPDLAPIAFFCLRQTTSPRNWCIKMVCNPWFECVSMLVILLNCVTLGMYQPCDDMDCLSDRCKILQVFDDFIFIFFAMEMVLKMVALGIFGKKCYLGDTWNRLDFFIVMAGMVEYSLDLQNINLSAIRTVRVLRPLKAINRVPSMRILVNLLLDTLPMLGNVLLLCFFVFFIFGIIGVQLWAGLLRNRCFLEENFTIQGDVALPPYYQPEEDDEMPFICSLSGDNGIMGCHEIPPLKEQGRECCLSKDDVYDFGAGRQDLNASGLCVNWNRYYNVCRTGSANPHKGAINFDNIGYAWIVIFQVITLEGWVEIMYYVMDAHSFYNFIYFILLIIVGSFFMINLCLVVIATQFSETKQREHRLMLEQRQRYLSSSTVASYAEPGDCYEEIFQYVCHILRKAKRRALGLYQALQSRRQALGPEAPAPAKPGPHAKEPRHYHGKTKGQGDEGRHLGSRHCQTLHGPASPGNDHSGRELCPQHSPLDATPHTLVQPIPATLASDPASCPCCQHEDGRRPSGLGSTDSGQEGSGSGSSAGGEDEADGDGARSSEDGASSELGKEEEEEEQADGAVWLCGDVWRETRAKLRGIVDSKYFNRGIMMAILVNTVSMGIEHHEQPEELTNILEICNVVFTSMFALEMILKLAAFGLFDYLRNPYNIFDSIIVIISIWEIVGQADGGLSVLRTFRLLRVLKLVRFMPALRRQLVVLMKTMDNVATFCMLLMLFIFIFSILGMHIFGCKFSLRTDTGDTVPDRKNFDSLLWAIVTVFQILTQEDWNVVLYNGMASTSPWASLYFVALMTFGNYVLFNLLVAILVEGFQAEGDANRSYSDEDQSSSNIEEFDKLQEGLDSSGDPKLCPIPMTPNGHLDPSLPLGGHLGPAGAAGPAPRLSLQPDPMLVALGSRKSSVMSLGRMSYDQRSLSSSRSSYYGPWGRSAAWASRRSSWNSLKHKPPSAEHESLLSAERGGGARVCEVAADEGPPRAAPLHTPHAHHIHHGPHLAHRHRHHRRTLSLDNRDSVDLAELVPAVGAHPRAAWRAAGPAPGHEDCNGRMPSIAKDVFTKMGDRGDRGEDEEEIDYTLCFRVRKMIDVYKPDWCEVREDWSVYLFSPENRFRVLCQTIIAHKLFDYVVLAFIFLNCITIALERPQIEAGSTERIFLTVSNYIFTAIFVGEMTLKVVSLGLYFGEQAYLRSSWNVLDGFLVFVSIIDIVVSLASAGGAKILGVLRVLRLLRTLRPLRVISRAPGLKLVVETLISSLKPIGNIVLICCAFFIIFGILGVQLFKGKFYHCLGVDTRNITNRSDCMAANYRWVHHKYNFDNLGQALMSLFVLASKDGWVNIMYNGLDAVAVDQQPVTNHNPWMLLYFISFLLIVSFFVLNMFVGVVVENFHKCRQHQEAEEARRREEKRLRRLEKKRRKAQRLPYYATYCHTRLLIHSMCTSHYLDIFITFIICLNVVTMSLEHYNQPTSLETALKYCNYMFTTVFVLEAVLKLVAFGLRRFFKDRWNQLDLAIVLLSVMGITLEEIEINAALPINPTIIRIMRVLRIARVLKLLKMATGMRALLDTVVQALPQVGNLGLLFMLLFFIYAALGVELFGKLVCNDENPCEGMSRHATFENFGMAFLTLFQVSTGDNWNGIMKDTLRDCTHDERSCLSSLQFVSPLYFVSFVLTAQFVLINVVVAVLMKHLDDSNKEAQEDAEMDAELELEMAHGLGPGPRLPTGSPGAPGRGPGGAGGGGDTEGGLCRRCYSPAQENLWLDSVSLIIKDSLEGELTIIDNLSGSIFHHYSSPAGCKKCHHDKQEVQLAETEAFSLNSDRSSSILLGDDLSLEDPTACPPGRKDSKGELDPPEPMRVGDLGECFFPLSSTAVSPDPENFLCEMEEIPFNPVRSWLKHDSSQAPPSPFSPDASSPLLPMPAEFFHPAVSASQKGPEKGTGTGTLPKIALQGSWASLRSPRVNCTLLRQATGSDTSLDASPSSSAGSLQTTLEDSLTLSDSPRRALGPPAPAPGPRAGLSPAARRRLSLRGRGLFSLRGLRAHQRSHSSGGSTSPGCTHHDSMDPSDEEGRGGAGGGGAGSEHSETLSSLSLTSLFCPPPPPPAPGLTPARKFSSTSSLAAPGRPHAAALAHGLARSPSWAADRSKDPPGRAPLPMGLGPLAPPPQPLPGELEPGDAASKRKR

>NP_003733.2 bile salt export pump [Homo sapiens]

MSDSVILRSIKKFGEENDGFESDKSYNNDKKSRLQDEKKGDGVRVGFFQLFRFSSSTDIWLMFVGSLCAFLHGIAQPGVLLIFGTMTDVFIDYDVELQELQIPGKACVNNTIVWTNSSLNQNMTNGTRCGLLNIESEMIKFASYYAGIAVAVLITGYIQICFWVIAAARQIQKMRKFYFRRIMRMEIGWFDCNSVGELNTRFSDDINKINDAIADQMALFIQRMTSTICGFLLGFFRGWKLTLVIISVSPLIGIGAATIGLSVSKFTDYELKAYAKAGVVADEVISSMRTVAAFGGEKREVERYEKNLVFAQRWGIRKGIVMGFFTGFVWCLIFLCYALAFWYGSTLVLDEGEYTPGTLVQIFLSVIVGALNLGNASPCLEAFATGRAAATSIFETIDRKPIIDCMSEDGYKLDRIKGEIEFHNVTFHYPSRPEVKILNDLNMVIKPGEMTALVGPSGAGKSTALQLIQRFYDPCEGMVTVDGHDIRSLNIQWLRDQIGIVEQEPVLFSTTIAENIRYGREDATMEDIVQAAKEANAYNFIMDLPQQFDTLVGEGGGQMSGGQKQRVAIARALIRNPKILLLDMATSALDNESEAMVQEVLSKIQHGHTIISVAHRLSTVRAADTIIGFEHGTAVERGTHEELLERKGVYFTLVTLQSQGNQALNEEDIKDATEDDMLARTFSRGSYQDSLRASIRQRSKSQLSYLVHEPPLAVVDHKSTYEEDRKDKDIPVQEEVEPAPVRRILKFSAPEWPYMLVGSVGAAVNGTVTPLYAFLFSQILGTFSIPDKEEQRSQINGVCLLFVAMGCVSLFTQFLQGYAFAKSGELLTKRLRKFGFRAMLGQDIAWFDDLRNSPGALTTRLATDASQVQGAAGSQIGMIVNSFTNVTVAMIIAFSFSWKLSLVILCFFPFLALSGATQTRMLTGFASRDKQALEMVGQITNEALSNIRTVAGIGKERRFIEALETELEKPFKTAIQKANIYGFCFAFAQCIMFIANSASYRYGGYLISNEGLHFSYVFRVISAVVLSATALGRAFSYTPSYAKAKISAARFFQLLDRQPPISVYNTAGEKWDNFQGKIDFVDCKFTYPSRPDSQVLNGLSVSISPGQTLAFVGSSGCGKSTSIQLLERFYDPDQGKVMIDGHDSKKVNVQFLRSNIGIVSQEPVLFACSIMDNIKYGDNTKEIPMERVIAAAKQAQLHDFVMSLPEKYETNVGSQGSQLSRGEKQRIAIARAIVRDPKILLLDEATSALDTESEKTVQVALDKAREGRTCIVIAHRLSTIQNADIIAVMAQGVVIEKGTHEELMAQKGAYYKLVTTGSPIS

>NP_003734.3 nuclear receptor coactivator 1 isoform 1 [Homo sapiens]

MSGLGDSSSDPANPDSHKRKGSPCDTLASSTEKRRREQENKYLEELAELLSANISDIDSLSVKPDKCKILKKTVDQIQLMKRMEQEKSTTDDDVQKSDISSSSQGVIEKESLGPLLLEALDGFFFVVNCEGRIVFVSENVTSYLGYNQEELMNTSVYSILHVGDHAEFVKNLLPKSLVNGVPWPQEATRRNSHTFNCRMLIHPPDEPGTENQEACQRYEVMQCFTVSQPKSIQEDGEDFQSCLICIARRLPRPPAITGVESFMTKQDTTGKIISIDTSSLRAAGRTGWEDLVRKCIYAFFQPQGREPSYARQLFQEVMTRGTASSPSYRFILNDGTMLSAHTKCKLCYPQSPDMQPFIMGIHIIDREHSGLSPQDDTNSGMSIPRVNPSVNPSISPAHGVARSSTLPPSNSNMVSTRINRQQSSDLHSSSHSNSSNSQGSFGCSPGSQIVANVALNQGQASSQSSNPSLNLNNSPMEGTGISLAQFMSPRRQVTSGLATRPRMPNNSFPPNISTLSSPVGMTSSACNNNNRSYSNIPVTSLQGMNEGPNNSVGFSASSPVLRQMSSQNSPSRLNIQPAKAESKDNKEIASILNEMIQSDNSSSDGKPLDSGLLHNNDRLSDGDSKYSQTSHKLVQLLTTTAEQQLRHADIDTSCKDVLSCTGTSNSASANSSGGSCPSSHSSLTERHKILHRLLQEGSPSDITTLSVEPDKKDSASTSVSVTGQVQGNSSIKLELDASKKKESKDHQLLRYLLDKDEKDLRSTPNLSLDDVKVKVEKKEQMDPCNTNPTPMTKPTPEEIKLEAQSQFTADLDQFDQLLPTLEKAAQLPGLCETDRMDGAVTSVTIKSEILPASLQSATARPTSRLNRLPELELEAIDNQFGQPGTGDQIPWTNNTVTAINQSKSEDQCISSQLDELLCPPTTVEGRNDEKALLEQLVSFLSGKDETELAELDRALGIDKLVQGGGLDVLSERFPPQQATPPLIMEERPNLYSQPYSSPSPTANLPSPFQGMVRQKPSLGTMPVQVTPPRGAFSPGMGMQPRQTLNRPPAAPNQLRLQLQQRLQGQQQLIHQNRQAILNQFAATAPVGINMRSGMQQQITPQPPLNAQMLAQRQRELYSQQHRQRQLIQQQRAMLMRQQSFGNNLPPSSGLPVQMGNPRLPQGAPQQFPYPPNYGTNPGTPPASTSPFSQLAANPEASLANRNSMVSRGMTGNIGGQFGTGINPQMQQNVFQYPGAGMVPQGEANFAPSLSPGSSMVPMPIPPPQSSLLQQTPPASGYQSPDMKAWQQGAIGNNNVFSQAVQNQPTPAQPGVYNNMSITVSMAGGNTNVQNMNPMMAQMQMSSLQMPGMNTVCPEQINDPALRHTGLYCNQLSSTDLLKTEADGTQQVQQVQVFADVQCTVNLVGGDPYLNQPGPLGTQKPTSGPQTPQAQQKSLLQQLLTE

>NP_671756.1 nuclear receptor coactivator 1 isoform 2 [Homo sapiens]

MSGLGDSSSDPANPDSHKRKGSPCDTLASSTEKRRREQENKYLEELAELLSANISDIDSLSVKPDKCKILKKTVDQIQLMKRMEQEKSTTDDDVQKSDISSSSQGVIEKESLGPLLLEALDGFFFVVNCEGRIVFVSENVTSYLGYNQEELMNTSVYSILHVGDHAEFVKNLLPKSLVNGVPWPQEATRRNSHTFNCRMLIHPPDEPGTENQEACQRYEVMQCFTVSQPKSIQEDGEDFQSCLICIARRLPRPPAITGVESFMTKQDTTGKIISIDTSSLRAAGRTGWEDLVRKCIYAFFQPQGREPSYARQLFQEVMTRGTASSPSYRFILNDGTMLSAHTKCKLCYPQSPDMQPFIMGIHIIDREHSGLSPQDDTNSGMSIPRVNPSVNPSISPAHGVARSSTLPPSNSNMVSTRINRQQSSDLHSSSHSNSSNSQGSFGCSPGSQIVANVALNQGQASSQSSNPSLNLNNSPMEGTGISLAQFMSPRRQVTSGLATRPRMPNNSFPPNISTLSSPVGMTSSACNNNNRSYSNIPVTSLQGMNEGPNNSVGFSASSPVLRQMSSQNSPSRLNIQPAKAESKDNKEIASILNEMIQSDNSSSDGKPLDSGLLHNNDRLSDGDSKYSQTSHKLVQLLTTTAEQQLRHADIDTSCKDVLSCTGTSNSASANSSGGSCPSSHSSLTERHKILHRLLQEGSPSDITTLSVEPDKKDSASTSVSVTGQVQGNSSIKLELDASKKKESKDHQLLRYLLDKDEKDLRSTPNLSLDDVKVKVEKKEQMDPCNTNPTPMTKPTPEEIKLEAQSQFTADLDQFDQLLPTLEKAAQLPGLCETDRMDGAVTSVTIKSEILPASLQSATARPTSRLNRLPELELEAIDNQFGQPGTGDQIPWTNNTVTAINQSKSEDQCISSQLDELLCPPTTVEGRNDEKALLEQLVSFLSGKDETELAELDRALGIDKLVQGGGLDVLSERFPPQQATPPLIMEERPNLYSQPYSSPSPTANLPSPFQGMVRQKPSLGTMPVQVTPPRGAFSPGMGMQPRQTLNRPPAAPNQLRLQLQQRLQGQQQLIHQNRQAILNQFAATAPVGINMRSGMQQQITPQPPLNAQMLAQRQRELYSQQHRQRQLIQQQRAMLMRQQSFGNNLPPSSGLPVQMGNPRLPQGAPQQFPYPPNYGTNPGTPPASTSPFSQLAANPEASLANRNSMVSRGMTGNIGGQFGTGINPQMQQNVFQYPGAGMVPQGEANFAPSLSPGSSMVPMPIPPPQSSLLQQTPPASGYQSPDMKAWQQGAIGNNNVFSQAVQNQPTPAQPGVYNNMSITVSMAGGNTNVQNMNPMMAQMQMSSLQMPGMNTVCPEQINDPALRHTGLYCNQLSSTDLLKTEADGTQDKKTEEFFSVVTTD

>NP_671766.1 nuclear receptor coactivator 1 isoform 3 [Homo sapiens]

MSGLGDSSSDPANPDSHKRKGSPCDTLASSTEKRRREQENKYLEELAELLSANISDIDSLSVKPDKCKILKKTVDQIQLMKRMEQEKSTTDDDVQKSDISSSSQGVIEKESLGPLLLEALDGFFFVVNCEGRIVFVSENVTSYLGYNQEELMNTSVYSILHVGDHAEFVKNLLPKSLVNGVPWPQEATRRNSHTFNCRMLIHPPDEPGTENQEACQRYEVMQCFTVSQPKSIQEDGEDFQSCLICIARRLPRPPAITGVESFMTKQDTTGKIISIDTSSLRAAGRTGWEDLVRKCIYAFFQPQGREPSYARQLFQEVMTRGTASSPSYRFILNDGTMLSAHTKCKLCYPQSPDMQPFIMGIHIIDREHSGLSPQDDTNSGMSIPRVNPSVNPSISPAHGVARSSTLPPSNSNMVSTRINRQQSSDLHSSSHSNSSNSQGSFGCSPGSQIVANVALNQGQASSQSSNPSLNLNNSPMEGTGISLAQFMSPRRQVTSGLATRPRMPNNSFPPNISTLSSPVGMTSSACNNNNRSYSNIPVTSLQGMNEGPNNSVGFSASSPVLRQMSSQNSPSRLNIQPAKAESKDNKEIASILNEMIQSDNSSSDGKPLDSGLLHNNDRLSDGDSKYSQTSHKLVQLLTTTAEQQLRHADIDTSCKDVLSCTGTSNSASANSSGGSCPSSHSSLTERHKILHRLLQEGSPSDITTLSVEPDKKDSASTSVSVTGQVQGNSSIKLELDASKKKESKDHQLLRYLLDKDEKDLRSTPNLSLDDVKVKVEKKEQMDPCNTNPTPMTKPTPEEIKLEAQSQFTADLDQFDQLLPTLEKAAQLPGLCETDRMDGAVTSVTIKSEILPASLQSATARPTSRLNRLPELELEAIDNQFGQPGTGDQIPWTNNTVTAINQSKSEDQCISSQLDELLCPPTTVEGRNDEKALLEQLVSFLSGKDETELAELDRALGIDKLVQGGGLDVLSERFPPQQATPPLIMEERPNLYSQPYSSPSPTANLPSPFQGMVRQKPSLGTMPVQVTPPRGAFSPGMGMQPRQTLNRPPAAPNQLRLQLQQRLQGQQQLIHQNRQAILNQFAATAPVGINMRSGMQQQITPQPPLNAQMLAQRQRELYSQQHRQRQLIQQQRAMLMRQQSFGNNLPPSSGLPVQMGNPRLPQGAPQQFPYPPNYGTNPGTPPASTSPFSQLAANPEASLANRNSMVSRGMTGNIGGQFGTGINPQMQQNVFQYPGAGMVPQGEANFAPSLSPGSSMVPMPIPPPQSSLLQQTPPASGYQSPDMKAWQQGAIGNNNVFSQAVQNQPTPAQPGVYNNMSITVSMAGGNTNVQNMNPMMAQMQMSSLQMPGMNTVCPEQINDPALRHTGLYCNQLSSTDLLKTEADGTQVQQVQVFADVQCTVNLVGGDPYLNQPGPLGTQKPTSGPQTPQAQQKSLLQQLLTE

>NP_004167.3 sterol regulatory element-binding protein 1 isoform 2 [Homo sapiens]

MDEPPFSEAALEQALGEPCDLDAALLTDIEDMLQLINNQDSDFPGLFDPPYAGSGAGGTDPASPDTSSPGSLSPPPATLSSSLEAFLSGPQAAPSPLSPPQPAPTPLKMYPSMPAFSPGPGIKEESVPLSILQTPTPQPLPGALLPQSFPAPAPPQFSSTPVLGYPSPPGGFSTGSPPGNTQQPLPGLPLASPPGVPPVSLHTQVQSVVPQQLLTVTAAPTAAPVTTTVTSQIQQVPVLLQPHFIKADSLLLTAMKTDGATVKAAGLSPLVSGTTVQTGPLPTLVSGGTILATVPLVVDAEKLPINRLAAGSKAPASAQSRGEKRTAHNAIEKRYRSSINDKIIELKDLVVGTEAKLNKSAVLRKAIDYIRFLQHSNQKLKQENLSLRTAVHKSKSLKDLVSACGSGGNTDVLMEGVKTEVEDTLTPPPSDAGSPFQSSPLSLGSRGSGSGGSGSDSEPDSPVFEDSKAKPEQRPSLHSRGMLDRSRLALCTLVFLCLSCNPLASLLGARGLPSPSDTTSVYHSPGRNVLGTESRDGPGWAQWLLPPVVWLLNGLLVLVSLVLLFVYGEPVTRPHSGPAVYFWRHRKQADLDLARGDFAQAAQQLWLALRALGRPLPTSHLDLACSLLWNLIRHLLQRLWVGRWLAGRAGGLQQDCALRVDASASARDAALVYHKLHQLHTMGKHTGGHLTATNLALSALNLAECAGDAVSVATLAEIYVAAALRVKTSLPRALHFLTRFFLSSARQACLAQSGSVPPAMQWLCHPVGHRFFVDGDWSVLSTPWESLYSLAGNPVDPLAQVTQLFREHLLERALNCVTQPNPSPGSADGDKEFSDALGYLQLLNSCSDAAGAPAYSFSISSSMATTTGVDPVAKWWASLTAVVIHWLRRDEEAAERLCPLVEHLPRVLQESERPLPRAALHSFKAARALLGCAKAESGPASLTICEKASGYLQDSLATTPASSSIDKAVQLFLCDLLLVVRTSLWRQQQPPAPAPAAQGTSSRPQASALELRGFQRDLSSLRRLAQSFRPAMRRVFLHEATARLMAGASPTRTHQLLDRSLRRRAGPGGKGGAVAELEPRPTRREHAEALLLASCYLPPGFLSAPGQRVGMLAEAARTLEKLGDRRLLHDCQQMLMRLGGGTTVTSS

>NP_683865.1 cathepsin E isoform b precursor [Homo sapiens]

MKTLLLLLLVLLELGEAQGSLHRVPLRRHPSLKKKLRARSQLSEFWKSHNLDMIQFTESCSMDQSAKEPLINYLDMEYFGTISIGSPPQNFTVIFDTGSSNLWVPSVYCTSPACKTHSRFQPSQSSTYSQPGQSFSIQYGTGSLSGIIGADQVSVEGLTVVGQQFGESVTEPGQTFVDAEFDGILGLGYPSLAVGGVTPVFDNMMAQNLVDLPMFSVYMSSNPEGGAGSELIFGGYDHSHFSGSLNWVPVTKQAYWQIALDNMLWSVPTLTSCRMSPSPLTESPIPSAQLPTPYWTSWMECSSAAVAFKDLTSTLQLGPSGSWGMSSFDSFTQSLTVGITVWDWPQQSPKEGPCVCACLSDRP

>NP_740753.1 DNA-directed RNA polymerase I subunit RPA12 [Homo sapiens]

MSVMDLANTCSSFQSDLDFCSDCGSVLPLPGAQDTVTCIRCGFNINVRDFEGKVVKTSVVFHQLGTAMPMSVEEGPECQGPVVDRRCPRCGHEGMAYHTRQMRSADEGQTVFYTCTNCKFQEKEDS

>AAN76500.1 P-glycoprotein [Homo sapiens]

MVVAHRLSTIRSADLIVTLKDGMLAEKGAHAELMAKRGLYYSLVMSQDIKKADEQMESMTYSTERKTNSLPLHSVKSIKSDFIDKAEESTQSKEISLPEVSLLKILKLNKPEWPFVVLGTLASVLNGTVHPVFSIIFAKIITMFGNNDKTTLKHDAEIYSMIFVILGVICFVSYFMQGLFYGRAGEILTMRLRHLAFKAMLYQDIAWFDEKENSTGGLTTILAIDIAQIQGATGSRIGVLTQNATNMGLSVIISFIYGWEMTFLILSIAPVLAVTGMIETAAMTGFANKDKQELKHAGKIATEALENIRTIVSLTREKAFEQMYEEMLQTQHRNTSKKAQIIGSCYAFSHAFIYFAYAAGFRFGAYLIQAGRMTPEGMFIVFTAIAYGAMAIGKTLVLAPEYSKAKSGAAHLFALLEKKPNIDSRSQEGKKPDTCEGNLEFREVSFFYPCRPDVFILRGLSLSIERGKTVAFVGSSGCGKSTSVQLLQRLYDPVQGQVLFDGVDAKELNVQWLRSQIAIVPQEPVLFNCSIAENIAYGDNSRVVPLDEIKEAANAANIHSFIEGLPEKYNTQVGLKGAQLSGGQKQRLAIARALLQKPKILLLDEATSALDNDSEKVVQHALDKARTGRTCLVVTHRLSAIQNADLIVVLHNGKIKEQGTHQELLRNRDIYFKLVNAQSVQ

>sp|Q9H252.1|KCNH6_HUMAN RecName: Full=Potassium voltage-gated channel subfamily H member 6; AltName: Full=Ether-a-go-go-related gene potassium channel 2; Short=ERG-2; Short=Eag-related protein 2; Short=Ether-a-go-go-related protein 2; Short=hERG-2; Short=hERG2; AltName: Full=Voltage-gated potassium channel subunit Kv11.2

MPVRRGHVAPQNTYLDTIIRKFEGQSRKFLIANAQMENCAIIYCNDGFCELFGYSRVEVMQQPCTCDFLTGPNTPSSAVSRLAQALLGAEECKVDILYYRKDASSFRCLVDVVPVKNEDGAVIMFILNFEDLAQLLAKCSSRSLSQRLLSQSFLGSEGSHGRPGGPGPGTGRGKYRTISQIPQFTLNFVEFNLEKHRSSSTTEIEIIAPHKVVERTQNVTEKVTQVLSLGADVLPEYKLQAPRIHRWTILHYSPFKAVWDWLILLLVIYTAVFTPYSAAFLLSDQDESRRGACSYTCSPLTVVDLIVDIMFVVDIVINFRTTYVNTNDEVVSHPRRIAVHYFKGWFLIDMVAAIPFDLLIFRTGSDETTTLIGLLKTARLLRLVRVARKLDRYSEYGAAVLFLLMCTFALIAHWLACIWYAIGNVERPYLEHKIGWLDSLGVQLGKRYNGSDPASGPSVQDKYVTALYFTFSSLTSVGFGNVSPNTNSEKVFSICVMLIGSLMYASIFGNVSAIIQRLYSGTARYHTQMLRVKEFIRFHQIPNPLRQRLEEYFQHAWSYTNGIDMNAVLKGFPECLQADICLHLHRALLQHCPAFSGAGKGCLRALAVKFKTTHAPPGDTLVHLGDVLSTLYFISRGSIEILRDDVVVAILGKNDIFGEPVSLHAQPGKSSADVRALTYCDLHKIQRADLLEVLDMYPAFAESFWSKLEVTFNLRDAAGGLHSSPRQAPGSQDHQGFFLSDNQSGSPHELGPQFPSKGYSLLGPGSQNSMGAGPCAPGHPDAAPPLSISDASGLWPELLQEMPPRHSPQSPQEDPDCWPLKLGSRLEQLQAQMNRLESRVSSDLSRILQLLQKPMPQGHASYILEAPASNDLALVPIASETTSPGPRLPQGFLPPAQTPSYGDLDDCSPKHRNSSPRMPHLAVATDKTLAPSSEQEQPEGLWPPLASPLHPLEVQGLICGPCFSSLPEHLGSVPKQLDFQRHGSDPGFAGSWGH

>sp|Q9NS40.1|KCNH7_HUMAN RecName: Full=Potassium voltage-gated channel subfamily H member 7; AltName: Full=Voltage-gated potassium channel subunit Kv11.3; AltName: Full=Ether-a-go-go-related gene potassium channel 3; Short=HERG-3; Short=Ether-a-go-go-related protein 3; Short=Eag-related protein 3

MPVRRGHVAPQNTFLGTIIRKFEGQNKKFIIANARVQNCAIIYCNDGFCEMTGFSRPDVMQKPCTCDFLHGPETKRHDIAQIAQALLGSEERKVEVTYYHKNGSTFICNTHIIPVKNQEGVAMMFIINFEYVTDNENAATPERVNPILPIKTVNRKFFGFKFPGLRVLTYRKQSLPQEDPDVVVIDSSKHSDDSVAMKHFKSPTKESCSPSEADDTKALIQPSKCSPLVNISGPLDHSSPKRQWDRLYPDMLQSSSQLSHSRSRESLCSIRRASSVHDIEGFGVHPKNIFRDRHASEDNGRNVKGPFNHIKSSLLGSTSDSNLNKYSTINKIPQLTLNFSEVKTEKKNSSPPSSDKTIIAPKVKDRTHNVTEKVTQVLSLGADVLPEYKLQTPRINKFTILHYSPFKAVWDWLILLLVIYTAIFTPYSAAFLLNDREEQKRRECGYSCSPLNVVDLIVDIMFIIDILINFRTTYVNQNEEVVSDPAKIAIHYFKGWFLIDMVAAIPFDLLIFGSGSDETTTLIGLLKTARLLRLVRVARKLDRYSEYGAAVLMLSMCIFALNAHWLACIWYAIGNVERPYLTDKIGWLDSLGQQIGKRYNDSDSSSGPSIKDKYVTALYFTFSSLTSVGFGNVSPNTNSEKIFSICVMLIGSLMYASIFGNVSAIIQRLYSGTARYHMQMLRVKEFIRFHQIPNPLRQRLEEYFQHAWTYTNGIDMNMVLKGFPECLQADICLHLNQTLLQNCKAFRGASKGCLRALAMKFKTTHALQGDTLVHCGDVLTALYFLSRGSIEISKNDMVVAILGKNDIFGEMVHLYAKPGKSNADVRALTYCDLHKIQREDLLEVLDMYPEFSDHFLTNLELTFNLRHESAKADLLRSQSMNDSEGDNCKLRRRKLSFESEGEKENSTNDPEDSADTIRHYQSSKRHFEEKKSRSSSFISSIDDEQKPLFSGIVDSSPGIGKASGLDFEETVPTSGRMHIDKRSHSCKDITDMRSWERENAHPQPEDSSPSALQRAAWGISETESDLTYGEVEQRLDLLQEQLNRLESQMTTDIQTILQLLQKQTTVVPPAYSMVTAGSEYQRPIIQLMRTSQPEASIKTDRSFSPSSQCPEFLDLEKSKLKSKESLSSGVHLNTASEDNLTSLLKQDSDLSLELHLRQRKTYVHPIRHPSLPDSSLSTVGIVGLHRHVSDPGLPGK

>NP_742053.1 potassium voltage-gated channel subfamily H member 2 isoform b [Homo sapiens]

MPVRRGHVAPQNTFLDTIIRKFEGQSRKFIIANARVENCAVIYCNDGFCELCGYSRAEVMQRPCTCDFLHGPRTQRRAAAQIAQALLGAEERKVEIAFYRKDGSCFLCLVDVVPVKNEDGAVIMFILNFEVVMEKDMVGSPAHDTNHRGPPTSWLAPGRAKTFRLKLPALLALTARESSVRSGGAGGAGAPGAVVVDVDLTPAAPSSESLALDEVTAMDNHVAGLGPAEERRALVGPGSPPRSAPGQLPSPRAHSLNPDASGSSCSLARTRSRESCASVRRASSADDIEAMRAGVLPPPPRHASTGAMHPLRSGLLNSTSDSDLVRYRTISKIPQITLNFVDLKGDPFLASPTSDREIIAPKIKERTHNVTEKVTQVLSLGADVLPEYKLQAPRIHRWTILHYSPFKAVWDWLILLLVIYTAVFTPYSAAFLLKETEEGPPATECGYACQPLAVVDLIVDIMFIVDILINFRTTYVNANEEVVSHPGRIAVHYFKGWFLIDMVAAIPFDLLIFGSGSEELIGLLKTARLLRLVRVARKLDRYSEYGAAVLFLLMCTFALIAHWLACIWYAIGNMEQPHMDSRIGWLHNLGDQIGKPYNSSGLGGPSIKDKYVTALYFTFSSLTSVGFGNVSPNTNSEKIFSICVMLIGSLMYASIFGNVSAIIQRLYSGTARYHTQMLRVREFIRFHQIPNPLRQRLEEYFQHAWSYTNGIDMNAVLKGFPECLQADICLHLNRSLLQHCKPFRGATKGCLRALAMKFKTTHAPPGDTLVHAGDLLTALYFISRGSIEILRGDVVVAILGMGWGAGTGLEMPSAASRGASLLNMQSLGLWTWDCLQGHWAPLIHLNSGPPSGAMERSPTWGEAAELWGSHILLPFRIRHKQTLFASLK

>NP_742054.1 potassium voltage-gated channel subfamily H member 2 isoform c [Homo sapiens]

MAAPAGKASRTGALRPRAQKGRVRRAVRISSLVAQEVLSLGADVLPEYKLQAPRIHRWTILHYSPFKAVWDWLILLLVIYTAVFTPYSAAFLLKETEEGPPATECGYACQPLAVVDLIVDIMFIVDILINFRTTYVNANEEVVSHPGRIAVHYFKGWFLIDMVAAIPFDLLIFGSGSEELIGLLKTARLLRLVRVARKLDRYSEYGAAVLFLLMCTFALIAHWLACIWYAIGNMEQPHMDSRIGWLHNLGDQIGKPYNSSGLGGPSIKDKYVTALYFTFSSLTSVGFGNVSPNTNSEKIFSICVMLIGSLMYASIFGNVSAIIQRLYSGTARYHTQMLRVREFIRFHQIPNPLRQRLEEYFQHAWSYTNGIDMNAVLKGFPECLQADICLHLNRSLLQHCKPFRGATKGCLRALAMKFKTTHAPPGDTLVHAGDLLTALYFISRGSIEILRGDVVVAILGKNDIFGEPLNLYARPGKSNGDVRALTYCDLHKIHRDDLLEVLDMYPEFSDHFWSSLEITFNLRDTNMIPGSPGSTELEGGFSRQRKRKLSFRRRTDKDTEQPGEVSALGPGRAGAGPSSRGRPGGPWGESPSSGPSSPESSEDEGPGRSSSPLRLVPFSSPRPPGEPPGGEPLMEDCEKSSDTCNPLSGAFSGVSNIFSFWGDSRGRQYQELPRCPAPTPSLLNIPLSSPGRRPRGDVESRLDALQRQLNRLETRLSADMATVLQLLQRQMTLVPPAYSAVTTPGPGPTSTSPLLPVSPLPTLTLDSLSQVSQFMACEELPPGAPELPQEGPTRRLSLPGQLGALTSQPLHRHGSDPGS

>NP_751895.1 potassium voltage-gated channel subfamily KQT member 4 isoform b [Homo sapiens]

MAEAPPRRLGLGPPPGDAPRAELVALTAVQSEQGEAGGGGSPRRLGLLGSPLPPGAPLPGPGSGSGSACGQRSSAAHKRYRRLQNWVYNVLERPRGWAFVYHVFIFLLVFSCLVLSVLSTIQEHQELANECLLILEFVMIVVFGLEYIVRVWSAGCCCRYRGWQGRFRFARKPFCVIDFIVFVASVAVIAAGTQGNIFATSALRSMRFLQILRMVRMDRRGGTWKLLGSVVYAHSKELITAWYIGFLVLIFASFLVYLAEKDANSDFSSYADSLWWGTITLTTIGYGDKTPHTWLGRVLAAGFALLGISFFALPAGILGSGFALKVQEQHRQKHFEKRRMPAANLIQAAWRLYSTDMSRAYLTATWYYYDSILPSFSSRMGIKDRIRMGSSQRRTGPSKQHLAPPTMPTSPSSEQVGEATSPTKVQKSWSFNDRTRFRASLRLKPRTSAEDAPSEEVAEEKSYQCELTVDDIMPAVKTVIRSIRILKFLVAKRKFKETLRPYDVKDVIEQYSAGHLDMLGRIKSLQTRVDQIVGRGPGDRKAREKGDKGPSDAEVVDEISMMGRVVKVEKQVQSIEHKLDLLLGFYSRCLRSGTSASLGAVQVPLFDPDITSDYHSPVDHEDISVSAQTLSISRSVSTNMD

>NP_751951.1 potassium voltage-gated channel subfamily E member 2 [Homo sapiens]

MSTLSNFTQTLEDVFRRIFITYMDNWRQNTTAEQEALQAKVDAENFYYVILYLMVMIGMFSFIIVAILVSTVKSKRREHSNDPYHQYIVEDWQEKYKSQILNLEESKATIHENIGAAGFKMSP

>NP_775267.1 protein-tyrosine kinase 2-beta isoform b [Homo sapiens]

MSGVSEPLSRVKLGTLRRPEGPAEPMVVVPVDVEKEDVRILKVCFYSNSFNPGKNFKLVKCTVQTEIREIITSILLSGRIGPNIRLAECYGLRLKHMKSDEIHWLHPQMTVGEVQDKYECLHVEAEWRYDLQIRYLPEDFMESLKEDRTTLLYFYQQLRNDYMQRYASKVSEGMALQLGCLELRRFFKDMPHNALDKKSNFELLEKEVGLDLFFPKQMQENLKPKQFRKMIQQTFQQYASLREEECVMKFFNTLAGFANIDQETYRCELIQGWNITVDLVIGPKGIRQLTSQDAKPTCLAEFKQIRSIRCLPLEEGQAVLQLGIEGAPQALSIKTSSLAEAENMADLIDGYCRLQGEHQGSLIIHPRKDGEKRNSLPQIPMLNLEARRSHLSESCSIESDIYAEIPDETLRRPGGPQYGIAREDVVLNRILGEGFFGEVYEGVYTNHKGEKINVAVKTCKKDCTLDNKEKFMSEAVIMKNLDHPHIVKLIGIIEEEPTWIIMELYPYGELGHYLERNKNSLKVLTLVLYSLQICKAMAYLESINCVHRDIAVRNILVASPECVKLGDFGLSRYIEDEDYYKASVTRLPIKWMSPESINFRRFTTASDVWMFAVCMWEILSFGKQPFFWLENKDVIGVLEKGDRLPKPDLCPPVLYTLMTRCWDYDPSDRPRFTELVCSLSDVYQMEKDIAMEQERNARYRTPKILEPTAFQEPPPKPSRPKYRPPPQTNLLAPKLQFQEEDFIQPSSREEAQQLWEAEKVKMRQILDKQQKQMVEDYQWLRQEEKSLDPMVYMNDKSPLTPEKEVGYLEFTGPPQKPPRLGAQSIQPTANLDRTDDLVYLNVMELVRAVLELKNELCQLPPEGYVVVVKNVGLTLRKLIGSVDDLLPSLPSSSRTEIEGTQKLLNKDLAELINKMRLAQQNAVTSLSEECKRQMLTASHTLAVDAKNLLDAVDQAKVLANLAHPPAE

>NP_775268.1 protein-tyrosine kinase 2-beta isoform a [Homo sapiens]

MSGVSEPLSRVKLGTLRRPEGPAEPMVVVPVDVEKEDVRILKVCFYSNSFNPGKNFKLVKCTVQTEIREIITSILLSGRIGPNIRLAECYGLRLKHMKSDEIHWLHPQMTVGEVQDKYECLHVEAEWRYDLQIRYLPEDFMESLKEDRTTLLYFYQQLRNDYMQRYASKVSEGMALQLGCLELRRFFKDMPHNALDKKSNFELLEKEVGLDLFFPKQMQENLKPKQFRKMIQQTFQQYASLREEECVMKFFNTLAGFANIDQETYRCELIQGWNITVDLVIGPKGIRQLTSQDAKPTCLAEFKQIRSIRCLPLEEGQAVLQLGIEGAPQALSIKTSSLAEAENMADLIDGYCRLQGEHQGSLIIHPRKDGEKRNSLPQIPMLNLEARRSHLSESCSIESDIYAEIPDETLRRPGGPQYGIAREDVVLNRILGEGFFGEVYEGVYTNHKGEKINVAVKTCKKDCTLDNKEKFMSEAVIMKNLDHPHIVKLIGIIEEEPTWIIMELYPYGELGHYLERNKNSLKVLTLVLYSLQICKAMAYLESINCVHRDIAVRNILVASPECVKLGDFGLSRYIEDEDYYKASVTRLPIKWMSPESINFRRFTTASDVWMFAVCMWEILSFGKQPFFWLENKDVIGVLEKGDRLPKPDLCPPVLYTLMTRCWDYDPSDRPRFTELVCSLSDVYQMEKDIAMEQERNARYRTPKILEPTAFQEPPPKPSRPKYRPPPQTNLLAPKLQFQVPEGLCASSPTLTSPMEYPSPVNSLHTPPLHRHNVFKRHSMREEDFIQPSSREEAQQLWEAEKVKMRQILDKQQKQMVEDYQWLRQEEKSLDPMVYMNDKSPLTPEKEVGYLEFTGPPQKPPRLGAQSIQPTANLDRTDDLVYLNVMELVRAVLELKNELCQLPPEGYVVVVKNVGLTLRKLIGSVDDLLPSLPSSSRTEIEGTQKLLNKDLAELINKMRLAQQNAVTSLSEECKRQMLTASHTLAVDAKNLLDAVDQAKVLANLAHPPAE

>NP_775115.1 potassium voltage-gated channel subfamily H member 6 isoform 2 [Homo sapiens]

MPVRRGHVAPQNTYLDTIIRKFEGQSRKFLIANAQMENCAIIYCNDGFCELFGYSRVEVMQQPCTCDFLTGPNTPSSAVSRLAQALLGAEECKVDILYYRKDASSFRCLVDVVPVKNEDGAVIMFILNFEDLAQLLAKCSSRSLSQRLLSQSFLGSEGSHGRPGGPGPGTGRGKYRTISQIPQFTLNFVEFNLEKHRSSSTTEIEIIAPHKVVERTQNVTEKVTQVLSLGADVLPEYKLQAPRIHRWTILHYSPFKAVWDWLILLLVIYTAVFTPYSAAFLLSDQDESRRGACSYTCSPLTVVDLIVDIMFVVDIVINFRTTYVNTNDEVVSHPRRIAVHYFKGWFLIDMVAAIPFDLLIFRTGSDETTTLIGLLKTARLLRLVRVARKLDRYSEYGAAVLFLLMCTFALIAHWLACICSLTSVGFGNVSPNTNSEKVFSICVMLIGSLMYASIFGNVSAIIQRLYSGTARYHTQMLRVKEFIRFHQIPNPLRQRLEEYFQHAWSYTNGIDMNAVLKGFPECLQADICLHLHRALLQHCPAFSGAGKGCLRALAVKFKTTHAPPGDTLVHLGDVLSTLYFISRGSIEILRDDVVVAILGKNDIFGEPVSLHAQPGKSSADVRALTYCDLHKIQRADLLEVLDMYPAFAESFWSKLEVTFNLRDAAGGLHSSPRQAPGSQDHQGFFLSDNQSDAAPPLSISDASGLWPELLQEMPPRHSPQSPQEDPDCWPLKLGSRLEQLQAQMNRLESRVSSDLSRILQLLQKPMPQGHASYILEAPASNDLALVPIASETTSPGPRLPQGFLPPAQTPSYGDLDDCSPKHRNSSPRMPHLAVATDKTLAPSSEQEQPEGLWPPLASPLHPLEVQGLICGPCFSSLPEHLGSVPKQLDFQRHGSDPGFAGSWGH

>NP_775185.1 potassium voltage-gated channel subfamily H member 7 isoform 2 [Homo sapiens]

MPVRRGHVAPQNTFLGTIIRKFEGQNKKFIIANARVQNCAIIYCNDGFCEMTGFSRPDVMQKPCTCDFLHGPETKRHDIAQIAQALLGSEERKVEVTYYHKNGSTFICNTHIIPVKNQEGVAMMFIINFEYVTDNENAATPERVNPILPIKTVNRKFFGFKFPGLRVLTYRKQSLPQEDPDVVVIDSSKHSDDSVAMKHFKSPTKESCSPSEADDTKALIQPSKCSPLVNISGPLDHSSPKRQWDRLYPDMLQSSSQLSHSRSRESLCSIRRASSVHDIEGFGVHPKNIFRDRHASEGPFNHIKSSLLGSTSDSNLNKYSTINKIPQLTLNFSEVKTEKKNSSPPSSDKTIIAPKVKDRTHNVTEKVTQVLSLGADVLPEYKLQTPRINKFTILHYSPFKAVWDWLILLLVIYTAIFTPYSAAFLLNDREEQKRRECGYSCSPLNVVDLIVDIMFIIDILINFRTTYVNQNEEVVSDPAKIAIHYFKGWFLIDMVAAIPFDLLIFGSGSDETTTLIGLLKTARLLRLVRVARKLDRYSEYGAAVLMLLMCIFALIAHWLACIWYAIGNVERPYLTDKIGWLDSLGQQIGKRYNDSDSSSGPSIKDKYVTALYFTFSSLTSVGFGNVSPNTNSEKIFSICVMLIGSLMYASIFGNVSAIIQRLYSGTARYHMQMLRVKEFIRFHQIPNPLRQRLEEYFQHAWTYTNGIDMNMVCMSVFQNESAAGIIVIAKME

>NP_005533.2 insulin-induced gene 1 protein isoform 1 [Homo sapiens]

MPRLHDHFWSCSCAHSARRRGPPRASAAGLAAKVGEMINVSVSGPSLLAAHGAPDADPAPRGRSAAMSGPEPGSPYPNTWHHRLLQRSLVLFSVGVVLALVLNLLQIQRNVTLFPEEVIATIFSSAWWVPPCCGTAAAVVGLLYPCIDSHLGEPHKFKREWASVMRCIAVFVGINHASAKLDFANNVQLSLTLAALSLGLWWTFDRSRSGLGLGITIAFLATLITQFLVYNGVYQYTSPDFLYIRSWLPCIFFSGGVTVGNIGRQLAMGVPEKPHSD

>NP_003158.2 sulfotransferase 2A1 [Homo sapiens]

MSDDFLWFEGIAFPTMGFRSETLRKVRDEFVIRDEDVIILTYPKSGTNWLAEILCLMHSKGDAKWIQSVPIWERSPWVESEIGYTALSETESPRLFSSHLPIQLFPKSFFSSKAKVIYLMRNPRDVLVSGYFFWKNMKFIKKPKSWEEYFEWFCQGTVLYGSWFDHIHGWMPMREEKNFLLLSYEELKQDTGRTIEKICQFLGKTLEPEELNLILKNSSFQSMKENKMSNYSLLSVDYVVDKAQLLRKGVSGDWKNHFTVAQAEDFDKLFQEKMADLPRELFPWE

>NP_848605.1 ankyrin repeat and protein kinase domain-containing protein 1 [Homo sapiens]

MAADPTELRLGSLPVFTRDDFEGDWRLVASGGFSQVFQARHRRWRTEYAIKCAPCLPPDAASSDVNYLIEEAAKMKKIKFQHIVSIYGVCKQPLGIVMEFMANGSLEKVLSTHSLCWKLRFRIIHETSLAMNFLHSIKPPLLHLDLKPGNILLDSNMHVKISDFGLSKWMEQSTRMQYIERSALRGMLSYIPPEMFLESNKAPGPKYDVYSFAIVIWELLTQKKPYSGFNMMMIIIRVAAGMRPSLQPVSDQWPSEAQQMVDLMKRCWDQDPKKRPCFLDITIETDILLSLLQSRVAVPESKALARKVSCKLSLRQPGEVNEDISQELMDSDSGNYLKRALQLSDRKNLVPRDEELCIYENKVTPLHFLVAQGSVEQVRLLLAHEVDVDCQTASGYTPLLIAAQDQQPDLCALLLAHGADANRVDEDGWAPLHFAAQNGDDGTARLLLDHGACVDAQEREGWTPLHLAAQNNFENVARLLVSRQADPNLHEAEGKTPLHVAAYFGHVSLVKLLTSQGAELDAQQRNLRTPLHLAVERGKVRAIQHLLKSGAVPDALDQSGYGPLHTAAARGKYLICKMLLRYGASLELPTHQGWTPLHLAAYKGHLEIIHLLAESHANMGALGAVNWTPLHLAARHGEEAVVSALLQCGADPNAAEQSGWTPLHLAVQRSTFLSVINLLEHHANVHARNKVGWTPAHLAALKGNTAILKVLVEAGAQLDVQDGVSCTPLQLALRSRKQGIMSFLEGKEPSVATLGGSKPGAEMEI

>NP_000448.3 hepatocyte nuclear factor 4-alpha isoform 2 [Homo sapiens]

MRLSKTLVDMDMADYSAALDPAYTTLEFENVQVLTMGNDTSPSEGTNLNAPNSLGVSALCAICGDRATGKHYGASSCDGCKGFFRRSVRKNHMYSCRFSRQCVVDKDKRNQCRYCRLKKCFRAGMKKEAVQNERDRISTRRSSYEDSSLPSINALLQAEVLSRQITSPVSGINGDIRAKKIASIADVCESMKEQLLVLVEWAKYIPAFCELPLDDQVALLRAHAGEHLLLGATKRSMVFKDVLLLGNDYIVPRHCPELAEMSRVSIRILDELVLPFQELQIDDNEYAYLKAIIFFDPDAKGLSDPGKIKRLRSQVQVSLEDYINDRQYDSRGRFGELLLLLPTLQSITWQMIEQIQFIKLFGMAKIDNLLQEMLLGGSPSDAPHAHHPLHPHLMQEHMGTNVIVANTMPTHLSNGQMCEWPRPRGQAATPETPQPSPPGGSGSEPYKLLPGAVATIVKPLSAIPQPTITKQEVI

>NP_849180.1 hepatocyte nuclear factor 4-alpha isoform 1 [Homo sapiens]

MRLSKTLVDMDMADYSAALDPAYTTLEFENVQVLTMGNDTSPSEGTNLNAPNSLGVSALCAICGDRATGKHYGASSCDGCKGFFRRSVRKNHMYSCRFSRQCVVDKDKRNQCRYCRLKKCFRAGMKKEAVQNERDRISTRRSSYEDSSLPSINALLQAEVLSRQITSPVSGINGDIRAKKIASIADVCESMKEQLLVLVEWAKYIPAFCELPLDDQVALLRAHAGEHLLLGATKRSMVFKDVLLLGNDYIVPRHCPELAEMSRVSIRILDELVLPFQELQIDDNEYAYLKAIIFFDPDAKGLSDPGKIKRLRSQVQVSLEDYINDRQYDSRGRFGELLLLLPTLQSITWQMIEQIQFIKLFGMAKIDNLLQEMLLGGSPSDAPHAHHPLHPHLMQEHMGTNVIVANTMPTHLSNGQMSTPETPQPSPPGGSGSEPYKLLPGAVATIVKPLSAIPQPTITKQEVI

>NP_849181.1 hepatocyte nuclear factor 4-alpha isoform 3 [Homo sapiens]

MRLSKTLVDMDMADYSAALDPAYTTLEFENVQVLTMGNDTSPSEGTNLNAPNSLGVSALCAICGDRATGKHYGASSCDGCKGFFRRSVRKNHMYSCRFSRQCVVDKDKRNQCRYCRLKKCFRAGMKKEAVQNERDRISTRRSSYEDSSLPSINALLQAEVLSRQITSPVSGINGDIRAKKIASIADVCESMKEQLLVLVEWAKYIPAFCELPLDDQVALLRAHAGEHLLLGATKRSMVFKDVLLLGNDYIVPRHCPELAEMSRVSIRILDELVLPFQELQIDDNEYAYLKAIIFFDPDAKGLSDPGKIKRLRSQVQVSLEDYINDRQYDSRGRFGELLLLLPTLQSITWQMIEQIQFIKLFGMAKIDNLLQEMLLGGPCQAQEGRGWSGDSPGDRPHTVSSPLSSLASPLCRFGQVA

>NP_733827.2 serine/threonine-protein kinase Sgk3 isoform 2 [Homo sapiens]

MQRDHTMDYKESCPSVSIPSSDEHREKKKRFTVYKVLVSVGRSEWFVFRRYAEFDKLYNTLKKQFPAMALKIPAKRIFGDNFDPDFIKQRRAGLNEFIQNLVRYPELYNHPDVRAFLQMDSPKHQSDPSEDEDERSSQKLHSTSQNINLGPSGNPHAKPTDFDFLKVIGKGSFGKVLLAKRKLDGKFYAVKVLQKKIVLNRKEQKHIMAERNVLLKNVKHPFLVGLHYSFQTTEKLYFVLDFVNGGELFFHLQRERSFPEHRARFYAAEIASALGYLHSIKIVYRDLKPENILLDSVGHVVLTDFGLCKEGIAISDTTTTFCGTPEPPFYCRDVAEMYDNILHKPLSLRPGVSLTAWSILEELLEKDRQNRLGAKEDFLEIQNHPFFESLSWADLVQKKIPPPFNPNVAGPDDIRNFDTAFTEETVPYSVCVSSDYSIVNASVLEADDAFVGFSYAPPSEDLFL

>NP_054790.2 nuclear receptor coactivator 6 isoform 1 [Homo sapiens]

MVLDDLPNLEDIYTSLCSSTMEDSEMDFDSGLEDDDTKSDSILEDSTIFVAFKGNIDDKDFKWKLDAILKNVPNLLHMESSKLKVQKVEPWNSVRVTFNIPREAAERLRILAQSNNQQLRDLGILSVQIEGEGAINLALAQNRSQDVRMNGPMGAGNSVRMEAGFPMASGPGIIRMNNPATVMIPPGGNVSSSMMAPGPNPELQPRTPRPASQSDAMDPLLSGLHIQQQSHPSGSLAPPHHPMQPVSVNRQMNPANFPQLQQQQQQQQQQQQQQQQQQQQQQQQQLQARPPQQHQQQQPQGIRPQFTAPTQVPVPPGWNQLPSGALQPPPAQGSLGTMTANQGWKKAPLPGPMQQQLQARPSLATVQTPSHPPPPYPFGSQQASQAHTNFPQMSNPGQFTAPQMKSLQGGPSRVPTPLQQPHLTNKSPASSPSSFQQGSPASSPTVNQTQQQMGPRPPQNNPLPQGFQQPVSSPGRNPMVQQGNVPPNFM

VMQQQPPNQGPQSLHPGLGGMPKRLPPGFSAGQANPNFMQGQVPSTTATTPGNSGAPQLQANQNVQHAGGQGAGPPQNQMQVSHGPPNMMQPSLMGIHGNMNNQQAGTSGVPQVNLSNMQGQPQQGPPSQLMGMHQQIVPSQGQMVQQQGTLNPQNPMILSRAQLMPQGQMMVNPPSQNLGPSPQRMTPPKQMLSQQGPQMMAPHNQMMGPQGQVLLQQNPMIEQIMTNQMQGNKQQFNTQNQSNVMPGPAQIMRGPTPNMQGNMVQFTGQMSGQMLPQQGPVNNSPSQVMGIQGQVLRPPGPSPHMAQQHGDPATTANNDVSLSQMMPDVSIQQTNMVPPHVQAMQGNSASGNHFSGHGMSFNAPFSGAPNGNQMSCGQNPGFPVNKDVTLTSPLLVNLLQSDISAGHFGVNNKQNNTNANKPKKKKPPRKKKNSQQDLNTPDTRPAGLEEADQPPLPGEQGINLDNSGPKLPEFSNRPPGYPSQPVEQRPLQQMPPQLMQHVAPPPQPPQQQPQPQLPQQQQPPPPSQPQSQQQQQQQQQMMMMLMMQQDPKSVRLPVSQNVHPPRGPLNPDSQRMPMQQSGSVPVMVSLQGPASVPPSPDKQRMPMPVNTPLGSNSRKMVYQESPQNPSSSPLAEMASLPEASGSEAPSVPGGPNNMPSHVVLPQNQLMMTGPKPGPSPLSATQGATPQQPPVNSLPSSHGHHFPNVAAPTQTSRPKTPNRASPRPYYPQTPNNRPPSTEPSEISLSPERLNASIAGLFPPQINIPLPPRPNLNRGFDQQGLNPTTLKAIGQAPSNLTMNPSNFATPQTHKLDSVVVNSGKQSNSGATKRASPSNSRRSSPGSSRKTTPSPGRQNSKAPKLTLASQTNAALLQNVELPRNVLVSPTPLANPPVPGSFPNNSGLNPQNSTVSVAAVGGVVEDNKESLNVPQDSDCQNSQSRKEQVNIELKAVPAQEVKMVVPEDQSKKDGQPSDPNKLPSVEENKNLVSPAMREAPTSLSQLLDNSGAPNVTIKPPGLTDLEVTPPVVSGEDLKKASVIPTLQDLSSSKEPSNSLNLPHSNELCSSLVHPELSEVSSNVAPSIPPVMSRPVSSSSISTPLPPNQITVFVTSNPITTSANTSAALPTHLQSALMSTVVTMPNAGSKVMVSEGQSAAQSNARPQFITPVFINSSSIIQVMKGSQPSTIPAAPLTTNSGLMPPSVAVVGPLHIPQNIKFSSAPVPPNALSSSPAPNIQTGRPLVLSSRATPVQLPSPPCTSSPVVPSHPPVQQVKELNPDEASPQVNTSADQNTLPSSQSTTMVSPLLTNSPGSSGNRRSPVSSSKGKGKVDKIGQILLTKACKKVTGSLEKGEEQYGADGETEGQGLDTTAPGLMGTEQLSTELDSKTPTPPAPTLLKMTSSPVGPGTASAGPSLPGGALPTSVRSIVTTLVPSELISAVPTTKSNHGGIASESLAGGLVEEKVGSHPELLPSIAPSQNLVSKETSTTALQASVARPELEVNAAIVSGQSSEPKEIVEKSKIPGRRNSRTEEPTVASESVENGHRKRSSRPASASSSTKDITSAVQSKRRKSK

>NP_000788.2 D(4) dopamine receptor [Homo sapiens]

MGNRSTADADGLLAGRGPAAGASAGASAGLAGQGAAALVGGVLLIGAVLAGNSLVCVSVATERALQTPTNSFIVSLAAADLLLALLVLPLFVYSEVQGGAWLLSPRLCDALMAMDVMLCTASIFNLCAISVDRFVAVAVPLRYNRQGGSRRQLLLIGATWLLSAAVAAPVLCGLNDVRGRDPAVCRLEDRDYVVYSSVCSFFLPCPLMLLLYWATFRGLQRWEVARRAKLHGRAPRRPSGPGPPSPTPPAPRLPQDPCGPDCAPPAPGLPRGPCGPDCAPAAPSLPQDPCGPDCAPPAPGLPPDPCGSNCAPPDAVRAAALPPQTPPQTRRRRRAKITGRERKAMRVLPVVVGAFLLCWTPFFVVHITQALCPACSVPPRLVSAVTWLGYVNSALNPVIYTVFNAEFRNVFRKALRACC

>AAQ03033.1 P-glycoprotein [Homo sapiens]

MGSTVVQLLQRLYDPDDGFIMVDENDIRALNVRHYRDHIGVVSQEPVLFGTTISNNIKYGRDDVTDEEMERAAREANAYDFIMEFPNKFNTLVGEKGAQMSGGQKQRIAIARALVRNPKILILDEATSALDSESKSAVQAALEKASKGRTTIVVAHRLSTIRSADLIVTLKDGMLAEKGAHAELMAKRGLYYSLVMSQDIKKADEQMESMTYSTERKTNSLPLHSVKSIKSDFIDKAEESTQSKEISLPEVSLLKILKLNKPEWPFVVLGTLASVLNGTVHPVFSIIFAKIITMFGNNDKTTLKHDAEIYSMIFVILGVICFVSYFMQGLFYGRAGEILTMRLRHLAFKAMLYQDIAWFDEKENSTGGLTTILAIDIAQIQGATGSRIGVLTQNATNMGLSVIISFIYGWEMTFLILSIAPVLAVTGMIETAAMTGFANKDKQELKHAGKIATEALENIRTIVSLTREKAFEQMYEEMLQTQHRNTSKKAQIIGSCYAFSHAFIYFAYAAGFRFGAYLIQAGRMTPEGMFIVFTAIAYGAMAIGKTLVLAPEYSKAKSGAAHLFALLEKKPNIDSRSQEGKKPDTCEGNLEFREVSFFYPCRPDVFILRGLSLSIERGKTVAFVGSSGCGKSTSVQLLQRLYDPVQGQVLFDGVDAKELNVQWLRSQIAIVPQEPVLFNCSIAENIAYGDNSRVVPLDEIKEAANAANIHSFIEGLPEKYNTQVGLKGAQLSGGQKQRLAIARALLQKPKILLLDEATSALDNDSEKVVQHALDKARTGRTCLVVTHRLSAIQNADLIVVLHNGKIKEQGTHQELLRNRDIYFKLVNAQSVQ

>NP_005393.2 ras-related protein Ral-A precursor [Homo sapiens]

MAANKPKGQNSLALHKVIMVGSGGVGKSALTLQFMYDEFVEDYEPTKADSYRKKVVLDGEEVQIDILDTAGQEDYAAIRDNYFRSGEGFLCVFSITEMESFAATADFREQILRVKEDENVPFLLVGNKSDLEDKRQVSVEEAKNRAEQWNVNYVETSAKTRANVDKVFFDLMREIRARKMEDSKEKNGKKKRKSLAKRIRERCCIL

>NP_899630.1 calcium-dependent secretion activator 1 isoform 3 [Homo sapiens]

MLDPSSSEEESDEIVEEESGKEVLGSAPSGARLSPSRTSEGSAGSAGLGGGGAGAGAGVGAGGGGGSGASSGGGAGGLQPSSRAGGGRPSSPSPSVVSEKEKEELERLQKEEEERKKRLQLYVFVMRCIAYPFNAKQPTDMARRQQKISKQQLQTVKDRFQAFLNGETQIMADEAFMNAVQSYYEVFLKSDRVARMVQSGGCSANDSREVFKKHIEKRVRSLPEIDGLSKETVLSSWMAKFDAIYRGEEDPRKQQARMTASAASELILSKEQLYEMFQNILGIKKFEHQLLYNACQLDNPDEQAAQIRRELDGRLQMADQIARERKFPKFVSKEMENMYIEELKSSVNLLMANLESMPVSKGGEFKLQKLKRSHNASIIDMGEESENQLSKSDVVLSFSLEVVIMEVQGLKSLAPNRIVYCTMEVEGGEKLQTDQAEASKPTWGTQGDFSTTHALPAVKVKLFTESTGVLALEDKELGRVILHPTPNSPKQSEWHKMTVSKNCPDQDLKIKLAVRMDKPQNMKHSGYLWAIGKNVWKRWKKRFFVLVQVSQYTFAMCSYREKKAEPQELLQLDGYTVDYTDPQPGLEGGRAFFNAVKEGDTVIFASDDEQDRILWVQAMYRATGQSHKPVPPTQVQKLNAKGGNVPQLDAPISQFYADRAQKHGMDEFISSNPCNFDHASLFEMGWFSPGQVFVLDEYCARNGVRGCHRHLCYLRDLLERAENGAMIDPTLLHYSFAFCASHVHGNRPDGIGTVTVEEKERFEEIKERLRVLLENQITHFRYCFPFGRPEGALKATLSLLERVLMKDIVTPVPQEEVKTVIRKCLEQAALVNYSRLSEYAKIEENVGRLITPAKKLEDTIRLAELVIEVLQQNEEHHAEAFAWWSDLMVEHAETFLSLFAVDMDAALEVQPPDTWDSFPLFQLLNDFLRTDYNLCNGKFHKHLQDLFAPLVVRYVDLMESSIAQSIHRGFERESWEPVNNGSGTSEDLFWKLDALQTFIRDLHWPEEEFGKHLEQRLKLMASDMIESCVKRTRIAFEVKLQKTSRSTDFRVPQSICTMFNVMVDAKAQSTKLCSMEMGQEHQYHSKIDELIEETVKEMITLLVAKFVTILEGVLAKLSRYDEGTLFSSFLSFTVKAASKYVDVPKPGMDVADAYVTFVRHSQDVLRDKVNEEMYIERLFDQWYNSSMNVICTWLTDRMDLQLHIYQLKTLIRMVKKTYRDFRLQGVLDSTLNSKTYETIRNRLTVEEATASVSEGGGLQGISMKDSDEEDEEDD

>NP_899631.1 calcium-dependent secretion activator 1 isoform 2 [Homo sapiens]

MLDPSSSEEESDEIVEEESGKEVLGSAPSGARLSPSRTSEGSAGSAGLGGGGAGAGAGVGAGGGGGSGASSGGGAGGLQPSSRAGGGRPSSPSPSVVSEKEKEELERLQKEEEERKKRLQLYVFVMRCIAYPFNAKQPTDMARRQQKISKQQLQTVKDRFQAFLNGETQIMADEAFMNAVQSYYEVFLKSDRVARMVQSGGCSANDSREVFKKHIEKRVRSLPEIDGLSKETVLSSWMAKFDAIYRGEEDPRKQQARMTASAASELILSKEQLYEMFQNILGIKKFEHQLLYNACQLDNPDEQAAQIRRELDGRLQMADQIARERKFPKFVSKEMENMYIEELKSSVNLLMANLESMPVSKGGEFKLQKLKRSHNASIIDMGEESENQLSKSDVVLSFSLEVVIMEVQGLKSLAPNRIVYCTMEVEGGEKLQTDQAEASKPTWGTQGDFSTTHALPAVKVKLFTESTGVLALEDKELGRVILHPTPNSPKQSEWHKMTVSKNCPDQDLKIKLAVRMDKPQNMKHSGYLWAIGKNVWKRWKKRFFVLVQVSQYTFAMCSYREKKAEPQELLQLDGYTVDYTDPQPGLEGGRAFFNAVKEGDTVIFASDDEQDRILWVQAMYRATGQSHKPVPPTQVQKLNAKGGNVPQLDAPISQFYADRAQKHGMDEFISSNPCNFDHASLFEMVQRLTLDHRLNDSYSCLGWFSPGQVFVLDEYCARNGVRGCHRHLCYLRDLLERAENGAMIDPTLLHYSFAFCASHVHGNRPDGIGTVTVEEKERFEEIKERLRVLLENQITHFRYCFPFGRPEGALKATLSLLERVLMKDIVTPVPQEEVKTVIRKCLEQAALVNYSRLSEYAKIEGKKREMYEHPVFCLASQVMDLTIQNVGRLITPAKKLEDTIRLAELVIEVLQQNEEHHAEAFAWWSDLMVEHAETFLSLFAVDMDAALEVQPPDTWDSFPLFQLLNDFLRTDYNLCNGKFHKHLQDLFAPLVVRYVDLMESSIAQSIHRGFERESWEPVNNGSGTSEDLFWKLDALQTFIRDLHWPEEEFGKHLEQRLKLMASDMIESCVKRTRIAFEVKLQKTSRSTDFRVPQSICTMFNVMVDAKAQSTKLCSMEMGQEHQYHSKIDELIEETVKEMITLLVAKFVTILEGVLAKLSRYDEGTLFSSFLSFTVKAASKYVDVPKPGMDVADAYVTFVRHSQDVLRDKVNEEMYIERLFDQWYNSSMNVICTWLTDRMDLQLHIYQLKTLIRMVKKTYRDFRLQGVLDSTLNSKTYETIRNRLTVEEATASVSEGGGLQGISMKDSDEEDEEDD

>NP_003707.2 calcium-dependent secretion activator 1 isoform 1 [Homo sapiens]

MLDPSSSEEESDEIVEEESGKEVLGSAPSGARLSPSRTSEGSAGSAGLGGGGAGAGAGVGAGGGGGSGASSGGGAGGLQPSSRAGGGRPSSPSPSVVSEKEKEELERLQKEEEERKKRLQLYVFVMRCIAYPFNAKQPTDMARRQQKISKQQLQTVKDRFQAFLNGETQIMADEAFMNAVQSYYEVFLKSDRVARMVQSGGCSANDSREVFKKHIEKRVRSLPEIDGLSKETVLSSWMAKFDAIYRGEEDPRKQQARMTASAASELILSKEQLYEMFQNILGIKKFEHQLLYNACQLDNPDEQAAQIRRELDGRLQMADQIARERKFPKFVSKEMENMYIEELKSSVNLLMANLESMPVSKGGEFKLQKLKRSHNASIIDMGEESENQLSKSDVVLSFSLEVVIMEVQGLKSLAPNRIVYCTMEVEGGEKLQTDQAEASKPTWGTQGDFSTTHALPAVKVKLFTESTGVLALEDKELGRVILHPTPNSPKQSEWHKMTVSKNCPDQDLKIKLAVRMDKPQNMKHSGYLWAIGKNVWKRWKKRFFVLVQVSQYTFAMCSYREKKAEPQELLQLDGYTVDYTDPQPGLEGGRAFFNAVKEGDTVIFASDDEQDRILWVQAMYRATGQSHKPVPPTQVQKLNAKGGNVPQLDAPISQFYADRAQKHGMDEFISSNPCNFDHASLFEMVQRLTLDHRLNDSYSCLGWFSPGQVFVLDEYCARNGVRGCHRHLCYLRDLLERAENGAMIDPTLLHYSFAFCASHVHGNRPDGIGTVTVEEKERFEEIKERLRVLLENQITHFRYCFPFGRPEGALKATLSLLERVLMKDIVTPVPQEEVKTVIRKCLEQAALVNYSRLSEYAKIEENQKDAENVGRLITPAKKLEDTIRLAELVIEVLQQNEEHHAEPHVDKGEAFAWWSDLMVEHAETFLSLFAVDMDAALEVQPPDTWDSFPLFQLLNDFLRTDYNLCNGKFHKHLQDLFAPLVVRYVDLMESSIAQSIHRGFERESWEPVKSLTSNLPNVNLPNVNLPKVPNLPVNIPLGIPQMPTFSAPSWMAAIYDADNGSGTSEDLFWKLDALQTFIRDLHWPEEEFGKHLEQRLKLMASDMIESCVKRTRIAFEVKLQKTSRSTDFRVPQSICTMFNVMVDAKAQSTKLCSMEMGQEHQYHSKIDELIEETVKEMITLLVAKFVTILEGVLAKLSRYDEGTLFSSFLSFTVKAASKYVDVPKPGMDVADAYVTFVRHSQDVLRDKVNEEMYIERLFDQWYNSSMNVICTWLTDRMDLQLHIYQLKTLIRMVKKTYRDFRLQGVLDSTLNSKTYETIRNRLTVEEATASVSEGGGLQGISMKDSDEEDEEDD

>AAH04311.2 KCNH2 protein, partial [Homo sapiens]

VLKGFPECLQADICLHLNRSLLQHCKPFRGATKGCLRALAMKFKTTHAPPGDTLVHAGDLLTALYFISRGSIEILRGDVVVAILGKNDIFGEPLNLYARPGKSNGDVRALTYCDLHKIHRDDLLEVLDMYPEFSDHFWSSLEITFNLRDTNMIPGSPGSTELEGGFSRQRKRKLSFRRRTDTDTEQPGEVSALGPGRAGAGPSSRGRPGGPWGESPSSGPSSPESSEDEGPGRSSSPLRLVPFSSPRPPGEPPGGEPLMEDCEKSSDTCNPLSGAFSGVSNIFSFWGDSRGRQYQELPRCPAPTPSLLNIPLSSPGRRPRGDVESRLDALQRQLNRLETRLSADMATVLQLLQRQMTLVPPAYSAVTTPGPGPTSTSPLLPVSPLPTLTLDSLSQVSQFMACEELPPGAPELPQEGPTRRLSLPGQLGALTSQPLHRHGSDPGS

>AAQ91594.1 potassium channel HERG, partial [Homo sapiens]

TEEGPPATECGYACQPLAVVDLIVDIMFIVDILINFRTTYVNANEEVVSHPGRIAVHYFKGWFLIDMVAAIPFDLLIFGSGSEE

>AAQ91599.1 potassium channel HERG, partial [Homo sapiens]

LIGLLKTARLLRLVRVARKLDRYSEYGAAVLFLLMCTFALIAHWLACIWYAIGNMEQPHMDSRIGWL

>AAQ91600.1 potassium channel HERG, partial [Homo sapiens]

LIGLLKTARLLRLVRVARKLDRYSEYGAAVLFLLMCTFALIAHWLACIWYAIGNMEQPHMDSRIGW

>AAM09027.1 P-glycoprotein [Homo sapiens]

MVDENDIRALNVRHYRDHIGVVSQEPVLFGTTISNNIKYGRDDVTDEEMERAAREANAYDFIMEFPNKFNTLVGEKGAQMSGGQKQRIAIARALVRNPKILILDEATSALDSESKSAVQAALEKASKGRTTIVVAHRLSTIRSADLIVTLKDGMLAEKGAHAELMAKRGLYYSLVMSQDIKKADEQMESMTYSTERKTNSLPLHSVKSIKSDFIDKAEESTQSKEISLPEVSLLKILKLNKPEWPFVVLGTLASVLNGTVHPVFSIIFAKIITMFGNNDKTTLKHDAEIYSMIFVILGVICFVSYFMQGLFYGRAGEILTMRLRHLAFKAMLYQDIAWFDEKENSTGGLTTILAIDIAQIQGATGSRIGVLTQNATNMGLSVIISFIYGWEMTFLILSIAPVLAVTGMIETAAMTGFANKDKQELKHAGKIATEALENIRTIVSLTREKAFEQMYEEMLQTQHRNTSKKAQIIGSCYAFSHAFIYFAYAAGFRFGAYLIQAGRMTPEGMFIVFTAIAYGAMAIGKTLVLAPEYSKAKSGAAHLFALLEKKPNIDSRSQEGKKPDTCEGNLEFREVSFFYPCRPDVFILRGLSLSIERGKTVAFVGSSGCGKSTSVQLLQRLYDPVQGQVLFDGVDAKELNVQWLRSQIAIVPQEPVLFNCSIAENIAYGDNSRVVPLDEIKEAANAANIHSFIEGLPEKYNTQVGLKGAQLSGGQKQRLAIARALLQKPKILLLDEATSALDNDSEKVVQHALDKARTGRTCLVVTHRLSAIQNADLIVVLHNGKIKEQGTHQELLRNRDIYFKLVNAQSVQ

>NP_000729.2 muscarinic acetylcholine receptor M1 [Homo sapiens]

MNTSAPPAVSPNITVLAPGKGPWQVAFIGITTGLLSLATVTGNLLVLISFKVNTELKTVNNYFLLSLACADLIIGTFSMNLYTTYLLMGHWALGTLACDLWLALDYVASNASVMNLLLISFDRYFSVTRPLSYRAKRTPRRAALMIGLAWLVSFVLWAPAILFWQYLVGERTVLAGQCYIQFLSQPIITFGTAMAAFYLPVTVMCTLYWRIYRETENRARELAALQGSETPGKGGGSSSSSERSQPGAEGSPETPPGRCCRCCRAPRLLQAYSWKEEEEEDEGSMESLTSSEGEEPGSEVVIKMPMVDPEAQAPTKQPPRSSPNTVKRPTKKGRDRAGKGQKPRGKEQLAKRKTFSLVKEKKAARTLSAILLAFILTWTPYNIMVLVSTFCKDCVPETLWELGYWLCYVNSTINPMCYALCNKAFRDTFRLLLLCRWDKRRWRKIPKRPGSVHRTPSRQC

>NP_938150.1 insulin induced gene 1 isoform 2 [Homo sapiens]

MPRLHDHFWSCSCAHSARRRGPPRASAAGLAAKVGEMINVSVSGPSLLAAHGAPDADPAPRGRSAAMSGPEPGSPYPNTWHHRLLQRSLVLFSVGVVLALVLNLLQIQRNVTLFPEEVIATIFSSAWWVPPCCGTAAAVVGLLYPCIDSHLGEPHKFKREWASVMRCIAVFVGINHASAKLDFANNVQLSLTLAALSLGLWWTFDRSRSGLGLGITIAFLATLITQFLVYNGVYQYTSPDFLYIRSWLPCIFFSGGVTVGNIGRQLAMLIPFCEELNLKTTWLFHKTRSNYRVFLKSPIVIESSKPPILRARKILEENLTVDYDKDYLFS

>NP_938151.1 insulin-induced gene 1 protein isoform 3 [Homo sapiens]

MPRLHDHFWSCSCAHSARRRGPPRASAAGLAAKVGEMINVSVSGPSLLAAHGAPDADPAPRGRSAAMSGPEPGSPYPNTWHHRLLQRSLVLFSVGVVLALVLNLLQIQRNVTLFPEEVIATIFSSAWWVPPCCGTAAGIHPQISSIFVLGSLVYFSQEASRWGT

>CAE82156.1 potassium voltage-gated channel, subfamily H (eag-related), member 2 [Homo sapiens]

MAAPAGKASRTGALRPRAQKGRVRRAVRISSLVAQEVLSLGADVLPEYKLQAPRIHRWTILHYSPFKAVWDWLILLLVIYTAVFTPYSAAFLLKETEEGPPATECGYACQPLAVVDLIVDIMFIVDILINFRTTYVNANEEVVSHPGRIAVHYFKGWFLIDMVAAIPFDLLIFGSGSEELIGLLKTARLLRLVRVARKLDRYSEYGAAVLFLLMCTFALIAHWLACIWYAIGNMEQPHMDSRIGWLHNLGDQIGKPYNSSGLGGPSIKDKYVTALYFTFSSLTSVGFGNVSPNTNSEKIFSICVMLIGSLMYASIFGNVSAIIQRLYSGTARYHTQMLRVREFIRFHQIPNPLRQRLEEYFQHAWSYTNGIDMNAVLKGFPECLQADICLHLNRSLLQHCKPFRGATKGCLRALAMKFKTTHAPPGDTLVHAGDLLTALYFISRGSIEILRGDVVVAILGMGWGAGTGLEMPSAASRGASLLNMQSLGLWTWDCLQGHWAPLIHLNSGPPSGAMERSPTWGEAAELWGSHILLPFRIRHKQTLFASLK

>NP_002063.2 guanine nucleotide-binding protein G(q) subunit alpha [Homo sapiens]

MTLESIMACCLSEEAKEARRINDEIERQLRRDKRDARRELKLLLLGTGESGKSTFIKQMRIIHGSGYSDEDKRGFTKLVYQNIFTAMQAMIRAMDTLKIPYKYEHNKAHAQLVREVDVEKVSAFENPYVDAIKSLWNDPGIQECYDRRREYQLSDSTKYYLNDLDRVADPAYLPTQQDVLRVRVPTTGIIEYPFDLQSVIFRMVDVGGQRSERRKWIHCFENVTSIMFLVALSEYDQVLVESDNENRMEESKALFRTIITYPWFQNSSVILFLNKKDLLEEKIMYSHLVDYFPEYDGPQRDAQAAREFILKMFVDLNPDSDKIIYSHFTCATDTENIRFVFAAVKDTILQLNLKEYNLV

>AAR83914.1 unknown [Homo sapiens]

MKKSTLFRYSPDS

>AAR91622.1 P-glycoprotein 1, partial [Homo sapiens]

MDLEGDRNGGAKKKNFFKLNNK

>AAR99172.1 P-glycoprotein, partial [Homo sapiens]

KPSIDSYSKSGHKPDNIKGNLEFRNVHFSYPSRKEVKILKGLNLKVQSGQTVALVGNSGCGKSTTVQLMQRLYDPTEGMLPFQLQMYESHTYFLFQVSVDGQDIRTINVRFLREIIGVVSQEPVLFATTIAENIRYGRENVTMDEIEKAVKEANAYDFIMKLPH

>NP_000738.2 acetylcholine receptor subunit beta precursor [Homo sapiens]

MTPGALLMLLGALGAPLAPGVRGSEAEGRLREKLFSGYDSSVRPAREVGDRVRVSVGLILAQLISLNEKDEEMSTKVYLDLEWTDYRLSWDPAEHDGIDSLRITAESVWLPDVVLLNNNDGNFDVALDISVVVSSDGSVRWQPPGIYRSSCSIQVTYFPFDWQNCTMVFSSYSYDSSEVSLQTGLGPDGQGHQEIHIHEGTFIENGQWEIIHKPSRLIQPPGDPRGGREGQRQEVIFYLIIRRKPLFYLVNVIAPCILITLLAIFVFYLPPDAGEKMGLSIFALLTLTVFLLLLADKVPETSLSVPIIIKYLMFTMVLVTFSVILSVVVLNLHHRSPHTHQMPLWVRQIFIHKLPLYLRLKRPKPERDLMPEPPHCSSPGSGWGRGTDEYFIRKPPSDFLFPKPNRFQPELSAPDLRRFIDGPNRAVALLPELREVVSSISYIARQLQEQEDHDALKEDWQFVAMVVDRLFLWTFIIFTSVGTLVIFLDATYHLPPPDPFP

>AAH01914.2 KCNH2 protein, partial [Homo sapiens]

VMQRPCTCDFLHGPRTQRRAAAQIAQALLGAEERKVEIAFYRKDGSCFLCLVDVVPVKNEDGAVIMFILNFEVVMEKDMVVDVDLTPAAPSSESLALDEVTAMDNHVAGLGPAEERRALVGPGSPPRSAPGQLPSPRAHSLNPDASGSSCSLARTRSRESCASVRRASSADDIEAMRAGVLPPPPRHASTGAMHPLRSGLLNSTSDSDLVRYRTISKIPQITLNFVDLKGDPFLASPTSDREIIAPKIKERTHNVTEKVTQVLSLGADVLPEYKLQAPRIHRWTILHYSPFKAVWDWLILLLVIYTAVFTPYSAAFLLKETEEGPPATECGYACQPLAVVDLIVDIMFIVDILINFRTTYVNANEEVVSHPGRIAVHYFKGWFLIDMVAAIPFDLLIFGSGSEELIGLLKTARLLRLVRVARKLDRYSEYGAAVLFLLMCTFALIAHWLACIWYAIGNMEQPHMDSRIGWLHNLGDQIGKPYNSSGLGGPSIKDKYVTALYFTFSSLTSVGFGNVSPNTNSEKIFSICVMLIGSLMYASIFGNVSAIIQRLYSGTARYHTQMLRVREFIRFHQIPNPLRQRLEEYFQHAWSYTNGIDMNAVLKGFPECLQADICLHLNRSLLQHCKPFRGATKGCLRALAMKFKTTHAPPGDTLVHAGDLLTALYFISRGSIEILRGDVVVAILGMGWGAGTGLEMPSAASRGASLLNMQSLGLWTWDCLQGHWAPLIHLNSGPPSGAMERSPTWGEAAELWGSHILLPFRIRHKQTLFASLK

>NP_996759.1 serine/threonine-protein phosphatase PP1-beta catalytic subunit isoform 1 [Homo sapiens]

MADGELNVDSLITRLLEVRGCRPGKIVQMTEAEVRGLCIKSREIFLSQPILLELEAPLKICGDIHGQYTDLLRLFEYGGFPPEANYLFLGDYVDRGKQSLETICLLLAYKIKYPENFFLLRGNHECASINRIYGFYDECKRRFNIKLWKTFTDCFNCLPIAAIVDEKIFCCHGGLSPDLQSMEQIRRIMRPTDVPDTGLLCDLLWSDPDKDVQGWGENDRGVSFTFGADVVSKFLNRHDLDLICRAHQVVEDGYEFFAKRQLVTLFSAPNYCGEFDNAGGMMSVDETLMCSFQILKPSEKKAKYQYGGLNSGRPVTPPRTANPPKKR

>sp|Q9NS86.1|LANC2_HUMAN RecName: Full=LanC-like protein 2; AltName: Full=Testis-specific adriamycin sensitivity protein

MGETMSKRLKLHLGGEAEMEERAFVNPFPDYEAAAGALLASGAAEETGCVRPPATTDEPGLPFHQDGKIIHNFIRRIQTKIKDLLQQMEEGLKTADPHDCSAYTGWTGIALLYLQLYRVTCDQTYLLRSLDYVKRTLRNLNGRRVTFLCGDAGPLAVGAVIYHKLRSDCESQECVTKLLQLQRSVVCQESDLPDELLYGRAGYLYALLYLNTEIGPGTVCESAIKEVVNAIIESGKTLSREERKTERCPLLYQWHRKQYVGAAHGMAGIYYMLMQPAAKVDQETLTEMVKPSIDYVRHKKFRSGNYPSSLSNETDRLVHWCHGAPGVIHMLMQAYKVFKEEKYLKEAMECSDVIWQRGLLRKGYGICHGTAGNGYSFLSLYRLTQDKKYLYRACKFAEWCLDYGAHGCRIPDRPYSLFEGMAGAIHFLSDVLGPETSRFPAFELDSSKRD

>AAT47159.1 constitutive androstane receptor SV1, partial [Homo sapiens]

MASREDELRNCVVCGDQATGYHFNALTCEGCKGFFRRTVSKSIGPTCPFAGSCEVSKTQRRHCPACRLQKCLDAGMRKDMILSAEALALRRAKQAQRRAQQTPVQLSKEQEELIRTLLGAHTRHMGTMFEQFVQFRPPAHLFIHHQPLPTLAPVLPLVTHFADINTFMVLQVIKFTKDLPVFRSLPIEDQISLLKGAAVEICHIVLNTTFCLQTQNFLCGPLRYTIEDGARVGFQVEFLELLFHFHGTLRKLQLQEPEYVLLAAMALFSPAPYLTDRPGVTQRDEIDQLQEEMALTLQSYIKGQQRRPRDRSPGTPWIHWSG

>AAT47160.1 constitutive androstane receptor SV2 [Homo sapiens]

MASREDELRNCVENSQQKHWSHLPLCWKL

>AAT47165.1 constitutive androstane receptor SV7, partial [Homo sapiens]

MLPKRSRRTVSKSIGPTCPFAGSCEVSKTQRRHCPACRLQKCLDAGMRKDMILSAEALALRRAKQAQRRAQQTPVQLSKEQEELIRTLLGAHTRHMGTMFEQFVQFRPPAHLFIHHQPLPTLAPVLPLVTHFADINTFMVLQVIKFTKDLPVFRSLPIEDQISLLKGAAVEICHIVLNTTFCLQTQNFLCGPLRYTIEDGARVSPTVGFQVEFLELLFHFHGTLRKLQLQEPEYVLLAAMALFSPAPYLTDRPGVTQRDEIDQLQEEMALTLQSYIKGQQRRPRDRSPGTPWIHWSG

>AAT47166.1 constitutive androstane receptor SV8, partial [Homo sapiens]

MASREDELRNCVVCGDQATGYHFNALTCEGCKGFFRRTVSKSIGPTCPFAGSCEVSKTQRRHCPACRLQKCLDAGMRKDMILSAEALALRRAKQAQRRAQQTPVQLSKEQEELIRTLLGAHTRHMGTMFEQFVQFRPPAHLFIHHQPLPTLAPVLPLVTHFADINTFMVLQVIKFTKDLPVFRSLPIEDQISLLKGAAVEICHIVLNTTFCLQTQNFLCGPLRYTIEDGARDRPGVTQRDEIDQLQEEMALTLQSYIKGQQRRPRDRSPGTPWIHWSG

>AAT47168.1 constitutive androstane receptor SV10 [Homo sapiens]

MLPKRSRRTVSKSIGPTCPFAGSCEVSKTQRRHCPACRLQKCLDAGMRKDTSSSSVHPSPALAHPGPCAASGHTLRRHQHFHGTASHQVY

>AAT47169.1 constitutive androstane receptor SV11, partial [Homo sapiens]

MLPKRSRRTVSKSIGPTCPFAGSCEVSKTQRRHCPACRLQKCLDAGMRKDMILSAEALALRRAKQAQRRAQQTPVQLSKEQEELIRTLLGAHTRHMGTMFEQFVQFRPPAHLFIHHQPLPTLAPVLPLVTHFADINTFMVLQVIKFTKDLPVFRSLPIEDQISLLKGAAVEICHIVLNTTFCLQTQNFLCGPLRYTIEDGARDRPGVTQRDEIDQLQEEMALTLQSYIKGQQRRPRDRSPGTPWIHWSG

>AAT47170.1 constitutive androstane receptor SV12, partial [Homo sapiens]

MLPKRSRRTVSKSIGPTCPFAGSCEVSKTQRRHCPACRLQKCLDAGMRKDMILSAEALALRRAKQAQRRAQQTPVQLSKEQEELIRTLLGAHTRHMGTMFEQFVQFRPPAHLFIHHQPLPTLAPVLPLVTHFADINTFMVLQVIKFTKDLPVFRSLPIEDQISLLKGAAVEICHIVLNTTFCLQTQNFLCGPLRYTIEDGARVGFQVEFLELLFHFHGTLRKLQLQEPEYVLLAAMALFSPAPYLTDRPGVTQRDEIDQLQEEMALTLQSYIKGQQRRPRDRSPGTPWIHWSG

>AAT47171.1 constitutive androstane receptor SV13, partial [Homo sapiens]

MLPKRSRRTVSKSIGPTCPFAGSCEVSKTQRRHCPACRLQKCLDAGMRKDMILSAEALALRRAKQAQRRAQQTPVQLSKEQEELIRTLLGAHTRHMGTMFEQFVQFRPPAHLFIHHQPLPTLAPVLPLVTHFADINTFMVLQVIKFTKDLPVFRSLPIEDQISLLKGAAVEICHIVLNTTFCLQTQNFLCGPLRYTIEDGARVGFQVEFLELLFHFHGTLRKLQLQEPEYVLLAAMALFSPDRPGVTQRDEIDQLQEEMALTLQSYIKGQQRRPRDRSPGTPWIHWSG

>AAT47172.1 constitutive androstane receptor SV14, partial [Homo sapiens]

MASREDELRNCVVCGDQATGYHFNALTCEGCKGFFRRTVSKSIGPTCPFAGSCEVSKTQRRHCPACRLQKCLDAGMRKDMILSAEALALRRAKQAQRRAQQTPVQLSKEQEELIRTLLGAHTRHMGTMFEQFVQFRPPAHLFIHHQPLPTLAPVLPLVTHFADINTFMVLQVIKFTKDLPVFRSLPIEDQISLLKGAAVEICHIVLNTTFCLQTQNFLCGPLRYTIEDGARVSPTVGFQVEFLELLFHFHGTLRKLQLQEPEYVLLAAMALFSPDRPGVTQRDEIDQLQEEMALTLQSYIKGQQRRPRDRSPGTPWIHWSG

>AAT47173.1 constitutive androstane receptor SV15, partial [Homo sapiens]

MLPKRSRRTVSKSIGPTCPFAGSCEVSKTQRRHCPACRLQKCLDAGMRKDMILSAEALALRRAKQAQRRAQQTPVQLPPAHLFIHHQPLPTLAPVLPLVTHFADINTFMVLQVIKFTKDLPVFRSLPIEDQISLLKGAAVEICHIVLNTTFCLQTQNFLCGPLRYTIEDGARVGFQVEFLELLFHFHGTLRKLQLQEPEYVLLAAMALFSPAPYLTDRPGVTQRDEIDQLQEEMALTLQSYIKGQQRRPRDRSPGTPWIHWSG

>AAT47174.1 constitutive androstane receptor SV16, partial [Homo sapiens]

MARRPCSLPSPAHLEHPGYTGVG

>AAT47176.1 constitutive androstane receptor SV18 [Homo sapiens]

MASREDELRNCVVCGDQATGYHFNALTCEGCKGFFRRTVSKSIGPTCPFAGSCEVSKTQRRHCPACRLQKCLDAGMRKDIPCPLKTRSPFSREQLWKSVTSYSIPLSVSKHKTSSAGLFATQLKMEPVTDLELPREMRLISCKRRWH

>AAT47178.1 constitutive androstane receptor SV20 [Homo sapiens]

MLPKRSRRTVSKSIGPTCPFAGSCEVSKTQRRHCPACRLQKCLDAGMRKDIPCPLKTRSPFSREQLWKSVTSYSIPLSVSKHKTSSAGLFATQLKMEPVTDLELPREMRLISCKRRWH

>AAT47179.1 constitutive androstane receptor SV21 [Homo sapiens]

MASREDELRNCVVCGDQATGYHFNALTCEGCKGFFRRTVSKSIGPTCPFAGSCEVSKTQRRHCPACRLQKCLDAGMRKDTSSSSVHPSPALAHPGPCAASGHTLRRHQHFHGTASHQVY

>AAT78421.1 Galphai2 protein [Homo sapiens]

MGCTVSAEDKAAAERSKMIDKNLREDGEKAAREVKLLLLGAGESGKSTIVKQMKIIHEDGYSEEECRQYRAVVYSNTIQSIMAIVKAMGNLQIDFADPSRADDARQLFALSCTAEEQGVLPDDLSGVIRRLWADHGVQACFGRSREYQLNDSAAYYLNDLERIAQSDYIPTQQDVLRTRVKTTGIVETHFTFKDLHFKMFDVGGQRSERKKWIHCFEGVTAIIFCVALSAYDLVLAEDEEMNRMHESMKLFDSICNNKWFTDTSIILFLNKKDLFEEKITHSPLTICFPEYTGANKYDEAASYIQSKFEDLNKRKDTKEIYTHFTCATDTKSRKLFRETYLKLSGPDQHPHPSPAPAPPLSSDSVP

>NP_001003406.1 voltage-dependent T-type calcium channel subunit alpha-1I isoform b [Homo sapiens]

MAESASPPSSSAAAPAAEPGVTTEQPGPRSPPSSPPGLEEPLDGADPHVPHPDLAPIAFFCLRQTTSPRNWCIKMVCNPWFECVSMLVILLNCVTLGMYQPCDDMDCLSDRCKILQVFDDFIFIFFAMEMVLKMVALGIFGKKCYLGDTWNRLDFFIVMAGMVEYSLDLQNINLSAIRTVRVLRPLKAINRVPSMRILVNLLLDTLPMLGNVLLLCFFVFFIFGIIGVQLWAGLLRNRCFLEENFTIQGDVALPPYYQPEEDDEMPFICSLSGDNGIMGCHEIPPLKEQGRECCLSKDDVYDFGAGRQDLNASGLCVNWNRYYNVCRTGSANPHKGAINFDNIGYAWIVIFQVITLEGWVEIMYYVMDAHSFYNFIYFILLIIVGSFFMINLCLVVIATQFSETKQREHRLMLEQRQRYLSSSTVASYAEPGDCYEEIFQYVCHILRKAKRRALGLYQALQSRRQALGPEAPAPAKPGPHAKEPRHYQLCPQHSPLDATPHTLVQPIPATLASDPASCPCCQHEDGRRPSGLGSTDSGQEGSGSGSSAGGEDEADGDGARSSEDGASSELGKEEEEEEQADGAVWLCGDVWRETRAKLRGIVDSKYFNRGIMMAILVNTVSMGIEHHEQPEELTNILEICNVVFTSMFALEMILKLAAFGLFDYLRNPYNIFDSIIVIISIWEIVGQADGGLSVLRTFRLLRVLKLVRFMPALRRQLVVLMKTMDNVATFCMLLMLFIFIFSILGMHIFGCKFSLRTDTGDTVPDRKNFDSLLWAIVTVFQILTQEDWNVVLYNGMASTSPWASLYFVALMTFGNYVLFNLLVAILVEGFQAEGDANRSYSDEDQSSSNIEEFDKLQEGLDSSGDPKLCPIPMTPNGHLDPSLPLGGHLGPAGAAGPAPRLSLQPDPMLVALGSRKSSVMSLGRMSYDQRSLSSSRSSYYGPWGRSAAWASRRSSWNSLKHKPPSAEHESLLSAERGGGARVCEVAADEGPPRAAPLHTPHAHHIHHGPHLAHRHRHHRRTLSLDNRDSVDLAELVPAVGAHPRAAWRAAGPAPGHEDCNGRMPSIAKDVFTKMGDRGDRGEDEEEIDYTLCFRVRKMIDVYKPDWCEVREDWSVYLFSPENRFRVLCQTIIAHKLFDYVVLAFIFLNCITIALERPQIEAGSTERIFLTVSNYIFTAIFVGEMTLKVVSLGLYFGEQAYLRSSWNVLDGFLVFVSIIDIVVSLASAGGAKILGVLRVLRLLRTLRPLRVISRAPGLKLVVETLISSLKPIGNIVLICCAFFIIFGILGVQLFKGKFYHCLGVDTRNITNRSDCMAANYRWVHHKYNFDNLGQALMSLFVLASKDGWVNIMYNGLDAVAVDQQPVTNHNPWMLLYFISFLLIVSFFVLNMFVGVVVENFHKCRQHQEAEEARRREEKRLRRLEKKRRKAQRLPYYATYCHTRLLIHSMCTSHYLDIFITFIICLNVVTMSLEHYNQPTSLETALKYCNYMFTTVFVLEAVLKLVAFGLRRFFKDRWNQLDLAIVLLSVMGITLEEIEINAALPINPTIIRIMRVLRIARVLKLLKMATGMRALLDTVVQALPQVGNLGLLFMLLFFIYAALGVELFGKLVCNDENPCEGMSRHATFENFGMAFLTLFQVSTGDNWNGIMKDTLRDCTHDERSCLSSLQFVSPLYFVSFVLTAQFVLINVVVAVLMKHLDDSNKEAQEDAEMDAELELEMAHGLGPGPRLPTGSPGAPGRGPGGAGGGGDTEGGLCRRCYSPAQENLWLDSVSLIIKDSLEGELTIIDNLSGSIFHHYSSPAGCKKCHHDKQEVQLAETEAFSLNSDRSSSILLGDDLSLEDPTACPPGRKDSKGELDPPEPMRVGDLGECFFPLSSTAVSPDPENFLCEMEEIPFNPVRSWLKHDSSQAPPSPFSPDASSPLLPMPAEFFHPAVSASQKGPEKGTGTGTLPKIALQGSWASLRSPRVNCTLLRQATGSDTSLDASPSSSAGSLQTTLEDSLTLSDSPRRALGPPAPAPGPRAGLSPAARRRLSLRGRGLFSLRGLRAHQRSHSSGGSTSPGCTHHDSMDPSDEEGRGGAGGGGAGSEHSETLSSLSLTSLFCPPPPPPAPGLTPARKFSSTSSLAAPGRPHAAALAHGLARSPSWAADRSKDPPGRAPLPMGLGPLAPPPQPLPGELEPGDAASKRKR

>sp|P63092.1|GNAS2_HUMAN RecName: Full=Guanine nucleotide-binding protein G(s) subunit alpha isoforms short; AltName: Full=Adenylate cyclase-stimulating G alpha protein

MGCLGNSKTEDQRNEEKAQREANKKIEKQLQKDKQVYRATHRLLLLGAGESGKSTIVKQMRILHVNGFNGEGGEEDPQAARSNSDGEKATKVQDIKNNLKEAIETIVAAMSNLVPPVELANPENQFRVDYILSVMNVPDFDFPPEFYEHAKALWEDEGVRACYERSNEYQLIDCAQYFLDKIDVIKQADYVPSDQDLLRCRVLTSGIFETKFQVDKVNFHMFDVGGQRDERRKWIQCFNDVTAIIFVVASSSYNMVIREDNQTNRLQEALNLFKSIWNNRWLRTISVILFLNKQDLLAEKVLAGKSKIEDYFPEFARYTTPEDATPEPGEDPRVTRAKYFIRDEFLRISTASGDGRHYCYPHFTCAVDTENIRRVFNDCRDIIQRMHLRQYELL

>NP_001005291.1 sterol regulatory element-binding protein 1 isoform 1 [Homo sapiens]

MDEPPFSEAALEQALGEPCDLDAALLTDIEGEVGAGRGRANGLDAPRAGADRGAMDCTFEDMLQLINNQDSDFPGLFDPPYAGSGAGGTDPASPDTSSPGSLSPPPATLSSSLEAFLSGPQAAPSPLSPPQPAPTPLKMYPSMPAFSPGPGIKEESVPLSILQTPTPQPLPGALLPQSFPAPAPPQFSSTPVLGYPSPPGGFSTGSPPGNTQQPLPGLPLASPPGVPPVSLHTQVQSVVPQQLLTVTAAPTAAPVTTTVTSQIQQVPVLLQPHFIKADSLLLTAMKTDGATVKAAGLSPLVSGTTVQTGPLPTLVSGGTILATVPLVVDAEKLPINRLAAGSKAPASAQSRGEKRTAHNAIEKRYRSSINDKIIELKDLVVGTEAKLNKSAVLRKAIDYIRFLQHSNQKLKQENLSLRTAVHKSKSLKDLVSACGSGGNTDVLMEGVKTEVEDTLTPPPSDAGSPFQSSPLSLGSRGSGSGGSGSDSEPDSPVFEDSKAKPEQRPSLHSRGMLDRSRLALCTLVFLCLSCNPLASLLGARGLPSPSDTTSVYHSPGRNVLGTESRDGPGWAQWLLPPVVWLLNGLLVLVSLVLLFVYGEPVTRPHSGPAVYFWRHRKQADLDLARGDFAQAAQQLWLALRALGRPLPTSHLDLACSLLWNLIRHLLQRLWVGRWLAGRAGGLQQDCALRVDASASARDAALVYHKLHQLHTMGKHTGGHLTATNLALSALNLAECAGDAVSVATLAEIYVAAALRVKTSLPRALHFLTRFFLSSARQACLAQSGSVPPAMQWLCHPVGHRFFVDGDWSVLSTPWESLYSLAGNPVDPLAQVTQLFREHLLERALNCVTQPNPSPGSADGDKEFSDALGYLQLLNSCSDAAGAPAYSFSISSSMATTTGVDPVAKWWASLTAVVIHWLRRDEEAAERLCPLVEHLPRVLQESERPLPRAALHSFKAARALLGCAKAESGPASLTICEKASGYLQDSLATTPASSSIDKAVQLFLCDLLLVVRTSLWRQQQPPAPAPAAQGTSSRPQASALELRGFQRDLSSLRRLAQSFRPAMRRVFLHEATARLMAGASPTRTHQLLDRSLRRRAGPGGKGGAVAELEPRPTRREHAEALLLASCYLPPGFLSAPGQRVGMLAEAARTLEKLGDRRLLHDCQQMLMRLGGGTTVTSS

>NP_036313.3 peptidyl-prolyl cis-trans isomerase FKBP8 isoform 1 [Homo sapiens]

MASCAEPSEPSAPLPAGVPPLEDFEVLDGVEDAEGEEEEEEEEEEEDDLSELPPLEDMGQPPAEEAEQPGALAREFLAAMEPEPAPAPAPEEWLDILGNGLLRKKTLVPGPPGSSRPVKGQVVTVHLQTSLENGTRVQEEPELVFTLGDCDVIQALDLSVPLMDVGETAMVTADSKYCYGPQGSRSPYIPPHAALCLEVTLKTAVDGPDLEMLTGQERVALANRKRECGNAHYQRADFVLAANSYDLAIKAITSSAKVDMTFEEEAQLLQLKVKCLNNLAASQLKLDHYRAALRSCSLVLEHQPDNIKALFRKGKVLAQQGEYSEAIPILRAALKLEPSNKTIHAELSKLVKKHAAQRSTETALYRKMLGNPSRLPAKCPGKGAWSIPWKWLFGATAVALGGVALSVVIAARN

>NP_000773.2 1,25-dihydroxyvitamin D(3) 24-hydroxylase, mitochondrial isoform 1 precursor [Homo sapiens]

MSSPISKSRSLAAFLQQLRSPRQPPRLVTSTAYTSPQPREVPVCPLTAGGETQNAAALPGPTSWPLLGSLLQILWKGGLKKQHDTLVEYHKKYGKIFRMKLGSFESVHLGSPCLLEALYRTESAYPQRLEIKPWKAYRDYRKEGYGLLILEGEDWQRVRSAFQKKLMKPGEVMKLDNKINEVLADFMGRIDELCDERGHVEDLYSELNKWSFESICLVLYEKRFGLLQKNAGDEAVNFIMAIKTMMSTFGRMMVTPVELHKSLNTKVWQDHTLAWDTIFKSVKACIDNRLEKYSQQPSADFLCDIYHQNRLSKKELYAAVTELQLAAVETTANSLMWILYNLSRNPQVQQKLLKEIQSVLPENQVPRAEDLRNMPYLKACLKESMRLTPSVPFTTRTLDKATVLGEYALPKGTVLMLNTQVLGSSEDNFEDSSQFRPERWLQEKEKINPFAHLPFGVGKRMCIGRRLAELQLHLALCWIVRKYDIQATDNEPVEMLHSGTLVPSRELPIAFCQR

>NP_061332.2 B-cell receptor-associated protein 29 isoform b [Homo sapiens]

MTLQWAAVATFLYAEIGLILIFCLPFIPPQRWQKIFSFNVWGKIATFWNKAFLTIIILLIVLFLDAVREVRKYSSVHTIEKSSTSRPDAYEHTQMKLFRSQRNLYISGFSLFFWLVLRRLVTLITQLAKELSNKGVLKTQAENTNKAAKKFMEENEKLKRILKSHGKDEECVLEAENKKLVEDQEKLKTELRKTSDALSKAQNDVMEMKMQSERLSKEYDQLLKEHSELQDRLERGNKKRL

>NP_001008405.1 B-cell receptor-associated protein 29 isoform a [Homo sapiens]

MTLQWAAVATFLYAEIGLILIFCLPFIPPQRWQKIFSFNVWGKIATFWNKAFLTIIILLIVLFLDAVREVRKYSSVHTIEKSSTSRPDAYEHTQMKLFRSQRNLYISGFSLFFWLVLRRLVTLITQLAKELSNKGVLKTQAENTNKAAKKFMEENEKLKRILKSHGKDEECVLEAENKKLVEDQEKLKTELRKTSDALSKAQNDVMEMKMQSERLSKEYDQLLKEHSELQHSSFGEFLSKRSHKNGSIGKQTGSRKGSFRKRQQEKTVNFIKDTCNILCQNDNFVMLASRKFKFRKMHYDRFVIFLMPHIGCIVMALSKYLMMFQIYCKVCIPALKKNISMLNTIFTY

>NP_001008406.1 B-cell receptor-associated protein BAP29 isoform c [Homo sapiens]

MTLQWAAVATFLYAEIGLILIFCLPFIPPQRWQKIFSFNVWGKIATFWNKAFLTIIILLIVLFLDAVREVRKYSSVHTIEKSSTSRPDAYEHTQMKLFRSQRNLYISGFSLFFWLVLRRLVTLITQLAKELSNKGVLKTQAENTNKAAKKFMEENEKLKRILKSHGKDEECVLEAENKKLVEDQEKLKTELRKTSDALSKAQNDVMEMKMQSERLSKEYDQLLKEHSELQKQREILPHRRGESTVTTEAEIGVMEPQQRNADSHQKLEEAKNRFFPRASSSRSMALQIPIKLILDFLASRTMGINLLFQAIKFVIICYCCHSKLIQEHAGQICNGLN

>pdb|1XV9|C Chain C, Retinoic acid receptor RXR-alpha

NEDMPVERILEAELAVEPKTETYVEANMGLNPSSPNDPVTNICQAADKQLFTLVEWAKRIPHFSELPLDDQVILLRAGWNELLIASFSHRSIAVKDGILLATGLHVHRNSAHSAGVGAIFDRVLTELVSKMRDMQMDKTELGCLRAIVLFNPDSKGLSNPAEVEALREKVYASLEAYCKHKYPEQPGRFAKLLLRLPALRSIGLKCLEHLFFFKLIGDTPIDTFLMEMLEAPHQMT

>pdb|1XV9|D Chain D, Orphan nuclear receptor NR1I3

PVQLSKEQEELIRTLLGAHTRHMGTMFEQFVQFRPPAHLFIHHQPLPTLAPVLPLVTHFADINTFMVLQVIKFTKDLPVFRSLPIEDQISLLKGAAVEICHIVLNTTFCLQTQNFLCGPLRYTIEDGARVGFQVEFLELLFHFHGTLRKLQLQEPEYVLLAAMALFSPDRPGVTQRDEIDQLQEEMALTLQSYIKGQQRRPRDRFLYAKLLGLLAELRSINEAYGYQIQHIQGLSAMMPLLQEICS

>BAD93069.1 dopamine receptor D2 isoform long variant, partial [Homo sapiens]

RAWPPSGSTALMDPLNLSWYDDDLERQNWSRPFNGSDGKADRPHYNYYATLLTLLIAVIVFGNVLVCMAVSREKALQTTTNYLIVSLAVADLLVATLVMPWVVYLEVGEWKFSRIHCDIFVTLDVMMCTASILNLCAISIDRYTAVAMPMLYNTRYSSKRRVTVMISIVWVLSFTISCPLLFGLNNADQNECIIANPAFVVYSSIVSFYVPFIVTLLVYIKIYIVLRRRRKRVNTKRSSRAFRAHLRAPLKGNCTHPEDMKLCTVIMKSNGSFPVNRRRVEAARRAQELEMEMLSSTSPPERTRYSPIPPSHHQLTLPDPSHHGLHSTPDSPAKPEKNGHAKDHPKIAKIFEIQTMPNGKTRTSLKTMSRRKLSQQKEKKATQMLAIVLGVFIICWLPFFITHILNIHCDCNIPPVLYSAFTWLGYVNSAVNPIIYTTFNIEFRKAFLKILHC

>pdb|1SKX|A Chain A, Orphan nuclear receptor PXR

MKKGHHHHHHGSERTGTQPLGVQGLTEEQRMMIRELMDAQMKTFDTTFSHFKNFRLPGVLSSGCELPESLQAPSREEAAKWSQVRKDLCSLKVSLQLRGEDGSVWNYKPPADSGGKEIFSLLPHMADMSTYMFKGIISFAKVISYFRDLPIEDQISLLKGAAFELCQLRFNTVFNAETGTWECGRLSYCLEDTAGGFQQLLLEPMLKFHYMLKKLQLHEEEYVLMQAISLFSPDRPGVLQHRVVDQLQEQFAITLKSYIECNRPQPAHRFLFLKIMAMLTELRSINAQHTQRLLRIQDIHPFATPLMQELFGI

>NP_005679.2 multidrug resistance-associated protein 5 isoform 1 [Homo sapiens]

MKDIDIGKEYIIPSPGYRSVRERTSTSGTHRDREDSKFRRTRPLECQDALETAARAEGLSLDASMHSQLRILDEEHPKGKYHHGLSALKPIRTTSKHQHPVDNAGLFSCMTFSWLSSLARVAHKKGELSMEDVWSLSKHESSDVNCRRLERLWQEELNEVGPDAASLRRVVWIFCRTRLILSIVCLMITQLAGFSGPAFMVKHLLEYTQATESNLQYSLLLVLGLLLTEIVRSWSLALTWALNYRTGVRLRGAILTMAFKKILKLKNIKEKSLGELINICSNDGQRMFEAAAVGSLLAGGPVVAILGMIYNVIILGPTGFLGSAVFILFYPAMMFASRLTAYFRRKCVAATDERVQKMNEVLTYIKFIKMYAWVKAFSQSVQKIREEERRILEKAGYFQSITVGVAPIVVVIASVVTFSVHMTLGFDLTAAQAFTVVTVFNSMTFALKVTPFSVKSLSEASVAVDRFKSLFLMEEVHMIKNKPASPHIKIEMKNATLAWDSSHSSIQNSPKLTPKMKKDKRASRGKKEKVRQLQRTEHQAVLAEQKGHLLLDSDERPSPEEEEGKHIHLGHLRLQRTLHSIDLEIQEGKLVGICGSVGSGKTSLISAILGQMTLLEGSIAISGTFAYVAQQAWILNATLRDNILFGKEYDEERYNSVLNSCCLRPDLAILPSSDLTEIGERGANLSGGQRQRISLARALYSDRSIYILDDPLSALDAHVGNHIFNSAIRKHLKSKTVLFVTHQLQYLVDCDEVIFMKEGCITERGTHEELMNLNGDYATIFNNLLLGETPPVEINSKKETSGSQKKSQDKGPKTGSVKKEKAVKPEEGQLVQLEEKGQGSVPWSVYGVYIQAAGGPLAFLVIMALFMLNVGSTAFSTWWLSYWIKQGSGNTTVTRGNETSVSDSMKDNPHMQYYASIYALSMAVMLILKAIRGVVFVKGTLRASSRLHDELFRRILRSPMKFFDTTPTGRILNRFSKDMDEVDVRLPFQAEMFIQNVILVFFCVGMIAGVFPWFLVAVGPLVILFSVLHIVSRVLIRELKRLDNITQSPFLSHITSSIQGLATIHAYNKGQEFLHRYQELLDDNQAPFFLFTCAMRWLAVRLDLISIALITTTGLMIVLM

HGQIPPAYAGLAISYAVQLTGLFQFTVRLASETEARFTSVERINHYIKTLSLEAPARIKNKAPSPDWPQEGEVTFENAEMRYRENLPLVLKKVSFTIKPKEKIGIVGRTGSGKSSLGMALFRLVELSGGCIKIDGVRISDIGLADLRSKLSIIPQEPVLFSGTVRSNLDPFNQYTEDQIWDALERTHMKECIAQLPLKLESEVMENGDNFSVGERQLLCIARALLRHCKILILDEATAAMDTETDLLIQETIREAFADCTMLTIAHRLHTVLGSDRIMVLAQGQVVEFDTPSVLLSNDSSRFYAMFAAAENKVAVKG

>NP_001018881.1 multidrug resistance-associated protein 5 isoform 2 [Homo sapiens]

MKDIDIGKEYIIPSPGYRSVRERTSTSGTHRDREDSKFRRTRPLECQDALETAARAEGLSLDASMHSQLRILDEEHPKGKYHHGLSALKPIRTTSKHQHPVDNAGLFSCMTFSWLSSLARVAHKKGELSMEDVWSLSKHESSDVNCRRLERLWQEELNEVGPDAASLRRVVWIFCRTRLILSIVCLMITQLAGFSGPNFQDGCILRSE

>NP_001019820.1 calnexin isoform d precursor [Homo sapiens]

MEGKWLLCMLLVLGTAIVEAHDGHDDDVIDIEDDLDDVIEEVEDSKPDTTAPPSSPKVTYKAPVPTGEVYFADSFDRGTLSGWILSKAKKDDTDDEIAKYDGKWEVEEMKESKLPGDKGLVLMSRAKHHAISAKLNKPFLFDTKPLIVQYEVNFQNGIECGGAYVKLLSKTPELNLDQFHDKTPYTIMFGPDKCGEDYKLHFIFRHKNPKTGIYEEKHAKRPDADLKTYFTDKKTHLYTLILNPDNSFEILVDQSVVNSGNLLNDMTPPVNPSREIEDPEDRKPEDWDERPKIPDPEAVKPDDWDEDAPAKIPDEEATKPEGWLDDEPEYVPDPDAEKPEDWDEDMDGEWEAPQIANPRCESAPGCGVWQRPVIDNPNYKGKWKPPMIDNPSYQGIWKPRKIPNPDFFEDLEPFRMTPFSAIGLELWSMTSDIFFDNFIICADRRIVDDWANDGWGLKKAADGAAEPGVVGQMIEAAEERPWLWVVYILTVALPVFLVILFCCSGKKQTSGMEYKKTDAPQPDVKEEEEEKEEEKDKGDEEEEGEEKLEEKQKSDAEEDGGTVSQEEEDRKPKAEEDEILNRSPRNRKPRRE

>NP_001025175.1 hepatocyte nuclear factor 4-alpha isoform 6 [Homo sapiens]

MVSVNAPLGAPVESSYDTSPSEGTNLNAPNSLGVSALCAICGDRATGKHYGASSCDGCKGFFRRSVRKNHMYSCRFSRQCVVDKDKRNQCRYCRLKKCFRAGMKKEAVQNERDRISTRRSSYEDSSLPSINALLQAEVLSRQITSPVSGINGDIRAKKIASIADVCESMKEQLLVLVEWAKYIPAFCELPLDDQVALLRAHAGEHLLLGATKRSMVFKDVLLLGNDYIVPRHCPELAEMSRVSIRILDELVLPFQELQIDDNEYAYLKAIIFFDPDAKGLSDPGKIKRLRSQVQVSLEDYINDRQYDSRGRFGELLLLLPTLQSITWQMIEQIQFIKLFGMAKIDNLLQEMLLGGPCQAQEGRGWSGDSPGDRPHTVSSPLSSLASPLCRFGQVA

>NP_787110.2 hepatocyte nuclear factor 4-alpha isoform 5 [Homo sapiens]

MVSVNAPLGAPVESSYDTSPSEGTNLNAPNSLGVSALCAICGDRATGKHYGASSCDGCKGFFRRSVRKNHMYSCRFSRQCVVDKDKRNQCRYCRLKKCFRAGMKKEAVQNERDRISTRRSSYEDSSLPSINALLQAEVLSRQITSPVSGINGDIRAKKIASIADVCESMKEQLLVLVEWAKYIPAFCELPLDDQVALLRAHAGEHLLLGATKRSMVFKDVLLLGNDYIVPRHCPELAEMSRVSIRILDELVLPFQELQIDDNEYAYLKAIIFFDPDAKGLSDPGKIKRLRSQVQVSLEDYINDRQYDSRGRFGELLLLLPTLQSITWQMIEQIQFIKLFGMAKIDNLLQEMLLGGSPSDAPHAHHPLHPHLMQEHMGTNVIVANTMPTHLSNGQMCEWPRPRGQAATPETPQPSPPGGSGSEPYKLLPGAVATIVKPLSAIPQPTITKQEVI

>NP_001025174.1 hepatocyte nuclear factor 4-alpha isoform 4 [Homo sapiens]

MVSVNAPLGAPVESSYDTSPSEGTNLNAPNSLGVSALCAICGDRATGKHYGASSCDGCKGFFRRSVRKNHMYSCRFSRQCVVDKDKRNQCRYCRLKKCFRAGMKKEAVQNERDRISTRRSSYEDSSLPSINALLQAEVLSRQITSPVSGINGDIRAKKIASIADVCESMKEQLLVLVEWAKYIPAFCELPLDDQVALLRAHAGEHLLLGATKRSMVFKDVLLLGNDYIVPRHCPELAEMSRVSIRILDELVLPFQELQIDDNEYAYLKAIIFFDPDAKGLSDPGKIKRLRSQVQVSLEDYINDRQYDSRGRFGELLLLLPTLQSITWQMIEQIQFIKLFGMAKIDNLLQEMLLGGSPSDAPHAHHPLHPHLMQEHMGTNVIVANTMPTHLSNGQMSTPETPQPSPPGGSGSEPYKLLPGAVATIVKPLSAIPQPTITKQEVI

>AAZ40507.1 potassium channel HERG1, partial [Homo sapiens]

MPVRRGHVAPQNTFLDTIIRKFEGQSRKFIIANAR

>NP_001028750.1 serine/threonine-protein kinase Sgk3 isoform 1 [Homo sapiens]

MQRDHTMDYKESCPSVSIPSSDEHREKKKRFTVYKVLVSVGRSEWFVFRRYAEFDKLYNTLKKQFPAMALKIPAKRIFGDNFDPDFIKQRRAGLNEFIQNLVRYPELYNHPDVRAFLQMDSPKHQSDPSEDEDERSSQKLHSTSQNINLGPSGNPHAKPTDFDFLKVIGKGSFGKVLLAKRKLDGKFYAVKVLQKKIVLNRKEQKHIMAERNVLLKNVKHPFLVGLHYSFQTTEKLYFVLDFVNGGELFFHLQRERSFPEHRARFYAAEIASALGYLHSIKIVYRDLKPENILLDSVGHVVLTDFGLCKEGIAISDTTTTFCGTPEYLAPEVIRKQPYDNTVDWWCLGAVLYEMLYGLPPFYCRDVAEMYDNILHKPLSLRPGVSLTAWSILEELLEKDRQNRLGAKEDFLEIQNHPFFESLSWADLVQKKIPPPFNPNVAGPDDIRNFDTAFTEETVPYSVCVSSDYSIVNASVLEADDAFVGFSYAPPSEDLFL

>sp|Q86UW7.2|CAPS2_HUMAN RecName: Full=Calcium-dependent secretion activator 2; AltName: Full=Calcium-dependent activator protein for secretion 2; Short=CAPS-2

MLDPSSSEEESDEGLEEESRDVLVAAGSSQRAPPAPTREGRRDAPGRAGGGGAARSVSPSPSVLSEGRDEPQRQLDDEQERRIRLQLYVFVVRCIAYPFNAKQPTDMARRQQKLNKQQLQLLKERFQAFLNGETQIVADEAFCNAVRSYYEVFLKSDRVARMVQSGGCSANDFREVFKKNIEKRVRSLPEIDGLSKETVLSSWIAKYDAIYRGEEDLCKQPNRMALSAVSELILSKEQLYEMFQQILGIKKLEHQLLYNACQLDNADEQAAQIRRELDGRLQLADKMAKERKFPKFIAKDMENMYIEELRSSVNLLMANLESLPVSKGGPEFKLQKLKRSQNSAFLDIGDENEIQLSKSDVVLSFTLEIVIMEVQGLKSVAPNRIVYCTMEVEGEKLQTDQAEASRPQWGTQGDFTTTHPRPVVKVKLFTESTGVLALEDKELGRVILYPTSNSSKSAELHRMVVPKNSQDSDLKIKLAVRMDKPAHMKHSGYLYALGQKVWKRWKKRYFVLVQVSQYTFAMCSYREKKSEPQELMQLEGYTVDYTDPHPGLQGGCMFFNAVKEGDTVIFASDDEQDRILWVQAMYRATGQSYKPVPAIQTQKLNPKGGTLHADAQLSGKDADRFQKHGMDEFISANPCKLDHAFLFRILQRQTLDHRLNDSYSCLGWFSPGQVFVLDEYCARYGVRGCHRHLCYLAELMEHSENGAVIDPTLLHYSFAFCASHVHGNRPDGIGTVSVEEKERFEEIKERLSSLLENQISHFRYCFPFGRPEGALKATLSLLERVLMKDIATPIPAEEVKKVVRKCLEKAALINYTRLTEYAKIEETMNQASPARKLEEILHLAELCIEVLQQNEEHHAEGREAFAWWPDLLAEHAEKFWALFTVDMDTALEAQPQDSWDSFPLFQLLNNFLRNDTLLCNGKFHKHLQEIFVPLVVRYVDLMESSIAQSIHRGFEQETWQPVKNIANSLPNVALPKVPSLPLNLPQIPNISTASWMPSLYESTNGSATSEDLFWKLDALQMFVFDLHWPEQEFAHHLEQRLKLMASDMLEACVKRTRTAFELKLQKASKTTDLRIPASVCTMFNVLVDAKKQSTKLCALDGGQEQQYHSKIDDLIDNSVKEIISLLVSKFVSVLEGVLSKLSRYDEGTFFSSILSFTVKAAAKYVDVPKPGMDLADTYIMFVRQNQDILREKVNEEMYIEKLFDQWYSSSMKVICVWLTDRLDLQLHIYQLKTLIKIVKKTYRDFRLQGVLEGTLNSKTYDTVHRRLTVEEATASVSEGGGLQGITMKDSDEEEEG

>NP_001034556.1 regulator of G-protein signaling 19 [Homo sapiens]

MPTPHEAEKQITGPEEADRPPSMSSHDTASPAAPSRNPCCLCWCCCCSCSWNQERRRAWQASRESKLQPLPSCEVCATPSPEEVQSWAQSFDKLMHSPAGRSVFRAFLRTEYSEENMLFWLACEELKAEANQHVVDEKARLIYEDYVSILSPKEVSLDSRVREGINKKMQEPSAHTFDDAQLQIYTLMHRDSYPRFLSSPTYRALLLQGPSQSSSEA

>NP_000787.2 D(3) dopamine receptor isoform a [Homo sapiens]

MASLSQLSGHLNYTCGAENSTGASQARPHAYYALSYCALILAIVFGNGLVCMAVLKERALQTTTNYLVVSLAVADLLVATLVMPWVVYLEVTGGVWNFSRICCDVFVTLDVMMCTASILNLCAISIDRYTAVVMPVHYQHGTGQSSCRRVALMITAVWVLAFAVSCPLLFGFNTTGDPTVCSISNPDFVIYSSVVSFYLPFGVTVLVYARIYVVLKQRRRKRILTRQNSQCNSVRPGFPQQTLSPDPAHLELKRYYSICQDTALGGPGFQERGGELKREEKTRNSLSPTIAPKLSLEVRKLSNGRLSTSLKLGPLQPRGVPLREKKATQMVAIVLGAFIVCWLPFFLTHVLNTHCQTCHVSPELYSATTWLGYVNSALNPVIYTTFNIEFRKAFLKILSC

>NP_387512.3 D(3) dopamine receptor isoform e [Homo sapiens]

MASLSQLSGHLNYTCGAENSTGASQARPHAYYALSYCALILAIVFGNGLVCMAVLKERALQTTTNYLVVSLAVADLLVATLVMPWVVYLEVTGGVWNFSRICCDVFVTLDVMMCTASILNLCAISIDRYTAVVMPVHYQHGTGQSSCRRVALMITAVWVLAFAVSCPLLFGFNTTGDPTVCSISNPDFVIYSSVVSFYLPFGVTVLVYARIYVVLKQRRRKRILTRQNSQCNSVRPGFPQQTLSPDPAHLELKRYYSICQDTALGGPGFQERGGELKREEKTRNSLMPLREKKATQMVAIVLGAFIVCWLPFFLTHVLNTHCQTCHVSPELYSATTWLGYVNSALNPVIYTTFNIEFRKAFLKILSC

>NP_060191.3 coiled-coil and C2 domain-containing protein 1A [Homo sapiens]

MHKRKGPPGPPGRGAAAARQLGLLVDLSPDGLMIPEDGANDEELEAEFLALVGGQPPALEKLKGKGPLPMEAIEKMASLCMRDPDEDEEEGTDEDDLEADDDLLAELNEVLGEEQKASETPPPVAQPKPEAPHPGLETTLQERLALYQTAIESARQAGDSAKMRRYDRGLKTLENLLASIRKGNAIDEADIPPPVAIGKGPASTPTYSPAPTQPAPRIASAPEPRVTLEGPSATAPASSPGLAKPQMPPGPCSPGPLAQLQSRQRDYKLAALHAKQQGDTTAAARHFRVAKSFDAVLEALSRGEPVDLSCLPPPPDQLPPDPPSPPSQPPTPATAPSTTEVPPPPRTLLEALEQRMERYQVAAAQAKSKGDQRKARMHERIVKQYQDAIRAHKAGRAVDVAELPVPPGFPPIQGLEATKPTQQSLVGVLETAMKLANQDEGPEDEEDEVPKKQNSPVAPTAQPKAPPSRTPQSGSAPTAKAPPKATSTRAQQQLAFLEGRKKQLLQAALRAKQKNDVEGAKMHLRQAKGLEPMLEASRNGLPVDITKVPPAPVNKDDFALVQRPGPGLSQEAARRYGELTKLIRQQHEMCLNHSNQFTQLGNITETTKFEKLAEDCKRSMDILKQAFVRGLPTPTARFEQRTFSVIKIFPDLSSNDMLLFIVKGINLPTPPGLSPGDLDVFVRFDFPYPNVEEAQKDKTSVIKNTDSPEFKEQFKLCINRSHRGFRRAIQTKGIKFEVVHKGGLFKTDRVLGTAQLKLDALEIACEVREILEVLDGRRPTGGRLEVMVRIREPLTAQQLETTTERWLVIDPVPAAVPTQVAGPKGKAPPVPAPARESGNRSARPLHSLSVLAFDQERLERKILALRQARRPVPPEVAQQYQDIMQRSQWQRAQLEQGGVGIRREYAAQLERQLQFYTEAARRLGNDGSRDAAKEALYRRNLVESELQRLRR

>ABF71886.1 voltage-gated potassium channel KV11.1 transcript variant 1 [Homo sapiens]

MPVRRGHVAPQSTFLDTIIRKFEGQSRKFIIANARVENCAVIYCNDGFCELCGYSRAEVMQRPCTCDFLHGPRTQRRAAAQIAQALLGAEERKVEIAFYRKDGSCFLCLVDVVPVKNEDGAVIMFILNFEVVMEKDMVGSPAHDTNHRGPPTSWLAPGRAKTFRLKLPALLALTARESSVRSGGAGGAGAPGAVVVDVDLTPAAPSSESLALDEVTAMDNHVAGLGPAEERRALVGPGSPPRSAPGQLPSPRAHSLNPDASGSSCSLARTRSRESCASVRRASSADDIEAMRAGVLPPPPRHASTGAMHPLRSGLLNSTSDSDLVRYRTISKIPQITLNFVDLKGDPFLASPTSDREIIAPKIKERTHNVTEKVTQVLSLGADVLPEYKLQAPRIHRWTILHYSPFKAVWDWLILLLVIYTAVFTPYSAAFLLKETEEGPPATECGYACQPLAVVDLIVDIMFIVDILINFRTTYVNANEEVVSHPGRIAVHYFKGWFLIDMVAAIPFDLLIFGSGSEELIGLLKTARLLRLVRVARKLDRYSEYGAAVLFLLMCTFALIAHWLACIWYAIGNMEQPHMDSRIGWLHNLGDQIGKPYNSSGLGGPSIKDKYVTALYFTFSSLTSVGFGNVSPNTNSEKIFSICVMLIGSLMYASIFGNVSAIIQRLYSGTARYHTQMLRVREFIRFHQIPNPLRQRLEEYFQHAWSYTNGIDMNAVLKGFPECLQADICLHLNRSLLQHCKPFRGATKGCLRALAMKFKTTHAPPGDTLVHAGDLLTALYFISRGSIEILRGDVVVAILGKNDIFGEPLNLYARPGKSNGDVRALTYCDLHKIHRDDLLEVLDMYPEFSDHFWSSLEITFNLRDTNMIPGSPGSTELEGGFSRQRKRKLSFRRRTDTDTEQPGEVSALGPGRAGAGPSSRGRPGGPWGESPSSGPSSPESSEDEGPGRSSSPLRLVPFSSPRPPGEPPGGEPLMEDCEKSSDTCNPLSGAFSGVSNIFSFWGDSRGRQYQELPRCPAPTPSLLNIPLSSPGRRPRGDVESRLDALQRQLNRLETRLSADMATVLQLLQRQMTLVPPAYSAVTTPGPGPTSTSPLLPVSPLPTLTLDSLSQVSQFMACEELPPGAPELPQEGPTRRLSLPGQLGALTSQPLHRHGSDPGS

>NP_150646.3 alpha-1A adrenergic receptor isoform 2 [Homo sapiens]

MVFLSGNASDSSNCTQPPAPVNISKAILLGVILGGLILFGVLGNILVILSVACHRHLHSVTHYYIVNLAVADLLLTSTVLPFSAIFEVLGYWAFGRVFCNIWAAVDVLCCTASIMGLCIISIDRYIGVSYPLRYPTIVTQRRGLMALLCVWALSLVISIGPLFGWRQPAPEDETICQINEEPGYVLFSALGSFYLPLAIILVMYCRVYVVAKRESRGLKSGLKTDKSDSEQVTLRIHRKNAPAGGSGMASAKTKTHFSVRLLKFSREKKAAKTLGIVVGCFVLCWLPFFLVMPIGSFFPDFKPSETVFKIVFWLGYLNSCINPIIYPCSSQEFKKAFQNVLRIQCLCRKQSSKHALGYTLHPPSQAVEGQHKDMVRIPVGSRETFYRISKTDGVCEWKFFSSMPRGSARITVSKDQSSCTTARTKSRSVTRLECSGMILAHCNLRLPGSRDSPASASQAAGTTGMCHQADATRPS

>NP_150645.2 alpha-1A adrenergic receptor isoform 3 [Homo sapiens]

MVFLSGNASDSSNCTQPPAPVNISKAILLGVILGGLILFGVLGNILVILSVACHRHLHSVTHYYIVNLAVADLLLTSTVLPFSAIFEVLGYWAFGRVFCNIWAAVDVLCCTASIMGLCIISIDRYIGVSYPLRYPTIVTQRRGLMALLCVWALSLVISIGPLFGWRQPAPEDETICQINEEPGYVLFSALGSFYLPLAIILVMYCRVYVVAKRESRGLKSGLKTDKSDSEQVTLRIHRKNAPAGGSGMASAKTKTHFSVRLLKFSREKKAAKTLGIVVGCFVLCWLPFFLVMPIGSFFPDFKPSETVFKIVFWLGYLNSCINPIIYPCSSQEFKKAFQNVLRIQCLCRKQSSKHALGYTLHPPSQAVEGQHKDMVRIPVGSRETFYRISKTDGVCEWKFFSSMPRGSARITVSKDQSSCTTARGHTPMT

>NP_150647.2 alpha-1A adrenergic receptor isoform 4 [Homo sapiens]

MVFLSGNASDSSNCTQPPAPVNISKAILLGVILGGLILFGVLGNILVILSVACHRHLHSVTHYYIVNLAVADLLLTSTVLPFSAIFEVLGYWAFGRVFCNIWAAVDVLCCTASIMGLCIISIDRYIGVSYPLRYPTIVTQRRGLMALLCVWALSLVISIGPLFGWRQPAPEDETICQINEEPGYVLFSALGSFYLPLAIILVMYCRVYVVAKRESRGLKSGLKTDKSDSEQVTLRIHRKNAPAGGSGMASAKTKTHFSVRLLKFSREKKAAKTLGIVVGCFVLCWLPFFLVMPIGSFFPDFKPSETVFKIVFWLGYLNSCINPIIYPCSSQEFKKAFQNVLRIQCLCRKQSSKHALGYTLHPPSQAVEGQHKDMVRIPVGSRETFYRISKTDGVCEWKFFSSMPRGSARITVSKDQSSCTTARRGMDCRYFTKNCREHIKHVNFMMPPWRKGSEC

>NP_002058.2 guanine nucleotide-binding protein subunit alpha-11 [Homo sapiens]

MTLESMMACCLSDEVKESKRINAEIEKQLRRDKRDARRELKLLLLGTGESGKSTFIKQMRIIHGAGYSEEDKRGFTKLVYQNIFTAMQAMIRAMETLKILYKYEQNKANALLIREVDVEKVTTFEHQYVSAIKTLWEDPGIQECYDRRREYQLSDSAKYYLTDVDRIATLGYLPTQQDVLRVRVPTTGIIEYPFDLENIIFRMVDVGGQRSERRKWIHCFENVTSIMFLVALSEYDQVLVESDNENRMEESKALFRTIITYPWFQNSSVILFLNKKDLLEDKILYSHLVDYFPEFDGPQRDAQAAREFILKMFVDLNPDSDKIIYSHFTCATDTENIRFVFAAVKDTILQLNLKEYNLV

>sp|Q2M3G0.2|ABCB5_HUMAN RecName: Full=ATP-binding cassette sub-family B member 5; AltName: Full=ABCB5 P-gp; AltName: Full=P-glycoprotein ABCB5

MVDENDIRALNVRHYRDHIGVVSQEPVLFGTTISNNIKYGRDDVTDEEMERAAREANAYDFIMEFPNKFNTLVGEKGAQMSGGQKQRIAIARALVRNPKILILDEATSALDSESKSAVQAALEKASKGRTTIVVAHRLSTIRSADLIVTLKDGMLAEKGAHAELMAKRGLYYSLVMSQDIKKADEQMESMTYSTERKTNSLPLHSVKSIKSDFIDKAEESTQSKEISLPEVSLLKILKLNKPEWPFVVLGTLASVLNGTVHPVFSIIFAKIITMFGNNDKTTLKHDAEIYSMIFVILGVICFVSYFMQGLFYGRAGEILTMRLRHLAFKAMLYQDIAWFDEKENSTGGLTTILAIDIAQIQGATGSRIGVLTQNATNMGLSVIISFIYGWEMTFLILSIAPVLAVTGMIETAAMTGFANKDKQELKHAGKIATEALENIRTIVSLTREKAFEQMYEEMLQTQHRNTSKKAQIIGSCYAFSHAFIYFAYAAGFRFGAYLIQAGRMTPEGMFIVFTAIAYGAMAIGKTLVLAPEYSKAKSGAAHLFALLEKKPNIDSRSQEGKKPDTCEGNLEFREVSFFYPCRPDVFILRGLSLSIERGKTVAFVGSSGCGKSTSVQLLQRLYDPVQGQVLFDGVDAKELNVQWLRSQIAIVPQEPVLFNCSIAENIAYGDNSRVVPLDEIKEAANAANIHSFIEGLPEKYNTQVGLKGAQLSGGQKQRLAIARALLQKPKILLLDEATSALDNDSEKVVQHALDKARTGRTCLVVTHRLSAIQNADLIVVLHNGKIKEQGTHQELLRNRDIYFKLVNAQSVQ

>NP_001070937.1 nuclear receptor subfamily 1 group I member 3 isoform 6 [Homo sapiens]

MASREDELRNCVVCGDQATGYHFNALTCEGCKGFFRRTVSKSIGPTCPFAGSCEVSKTQRRHCPACRLQKCLDAGMRKDMILSAEALALRRAKQAQRRAQQTPVQLSKEQEELIRTLLGAHTRHMGTMFEQFVQFRPPAHLFIHHQPLPTLAPVLPLVTHFADINTFMVLQVIKFTKDLPVFRSLPIEDQISLLKGAAVEICHIVLNTTFCLQTQNFLCGPLRYTIEDGARVGFQVEFLELLFHFHGTLRKLQLQEPEYVLLAAMALFSPAPYLTDRPGVTQRDEIDQLQEEMALTLQSYIKGQQRRPRDRSPGTPWIHWSGKMLGPKIGPGSKGAQWLQ

>NP_001070938.1 nuclear receptor subfamily 1 group I member 3 isoform 11 [Homo sapiens]

MLPKRSRRTVSKSIGPTCPFAGSCEVSKTQRRHCPACRLQKCLDAGMRKDMILSAEALALRRAKQAQRRAQQTPVQLSKEQEELIRTLLGAHTRHMGTMFEQFVQFRPPAHLFIHHQPLPTLAPVLPLVTHFADINTFMVLQVIKFTKDLPVFRSLPIEDQISLLKGAAVEICHIVLNTTFCLQTQNFLCGPLRYTIEDGARDRPGVTQRDEIDQLQEEMALTLQSYIKGQQRRPRDRFLYAKLLGLLAELRSINEAYGYQIQHIQGLSAMMPLLQEICS

>NP_001070939.1 nuclear receptor subfamily 1 group I member 3 isoform 5 [Homo sapiens]

MASREDELRNCVVCGDQATGYHFNALTCEGCKGFFRRTVSKSIGPTCPFAGSCEVSKTQRRHCPACRLQKCLDAGMRKDMILSAEALALRRAKQAQRRAQQTPVQLSKEQEELIRTLLGAHTRHMGTMFEQFVQFRPPAHLFIHHQPLPTLAPVLPLVTHFADINTFMVLQVIKFTKDLPVFRSLPIEDQISLLKGAAVEICHIVLNTTFCLQTQNFLCGPLRYTIEDGARDRPGVTQRDEIDQLQEEMALTLQSYIKGQQRRPRDRFLYAKLLGLLAELRSINEAYGYQIQHIQGLSAMMPLLQEICS

>NP_001070940.1 nuclear receptor subfamily 1 group I member 3 isoform 9 [Homo sapiens]

MLPKRSRRTVSKSIGPTCPFAGSCEVSKTQRRHCPACRLQKCLDAGMRKDMILSAEALALRRAKQAQRRAQQTPVQLSKEQEELIRTLLGAHTRHMGTMFEQFVQFRPPAHLFIHHQPLPTLAPVLPLVTHFADINTFMVLQVIKFTKDLPVFRSLPIEDQISLLKGAAVEICHIVLNTTFCLQTQNFLCGPLRYTIEDGARVGFQVEFLELLFHFHGTLRKLQLQEPEYVLLAAMALFSPAPYLTDRPGVTQRDEIDQLQEEMALTLQSYIKGQQRRPRDRFLYAKLLGLLAELRSINEAYGYQIQHIQGLSAMMPLLQEICS

>NP_001070941.1 nuclear receptor subfamily 1 group I member 3 isoform 12 [Homo sapiens]

MLPKRSRRTVSKSIGPTCPFAGSCEVSKTQRRHCPACRLQKCLDAGMRKDMILSAEALALRRAKQAQRRAQQTPVQLSKEQEELIRTLLGAHTRHMGTMFEQFVQFRPPAHLFIHHQPLPTLAPVLPLVTHFADINTFMVLQVIKFTKDLPVFRSLPIEDQISLLKGAAVEICHIVLNTTFCLQTQNFLCGPLRYTIEDGARVSPTVGFQVEFLELLFHFHGTLRKLQLQEPEYVLLAAMALFSPAPYLTDRPGVTQRDEIDQLQEEMALTLQSYIKGQQRRPRDRSPGTPWIHWSGKMLGPKIGPGSKGAQWLQ

>NP_001070942.1 nuclear receptor subfamily 1 group I member 3 isoform 8 [Homo sapiens]

MASREDELRNCVVCGDQATGYHFNALTCEGCKGFFRRTVSKSIGPTCPFAGSCEVSKTQRRHCPACRLQKCLDAGMRKDMILSAEALALRRAKQAQRRAQQTPVQLSKEQEELIRTLLGAHTRHMGTMFEQFVQFRPPAHLFIHHQPLPTLAPVLPLVTHFADINTFMVLQVIKFTKDLPVFRSLPIEDQISLLKGAAVEICHIVLNTTFCLQTQNFLCGPLRYTIEDGARDRPGVTQRDEIDQLQEEMALTLQSYIKGQQRRPRDRSPGTPWIHWSGKMLGPKIGPGSKGAQWLQ

>NP_001070943.1 nuclear receptor subfamily 1 group I member 3 isoform 15 [Homo sapiens]

MLPKRSRRTVSKSIGPTCPFAGSCEVSKTQRRHCPACRLQKCLDAGMRKDMILSAEALALRRAKQAQRRAQQTPVQLSKEQEELIRTLLGAHTRHMGTMFEQFVQFRPPAHLFIHHQPLPTLAPVLPLVTHFADINTFMVLQVIKFTKDLPVFRSLPIEDQISLLKGAAVEICHIVLNTTFCLQTQNFLCGPLRYTIEDGARDRPGVTQRDEIDQLQEEMALTLQSYIKGQQRRPRDRSPGTPWIHWSGKMLGPKIGPGSKGAQWLQ

>NP_001070944.1 nuclear receptor subfamily 1 group I member 3 isoform 13 [Homo sapiens]

MLPKRSRRTVSKSIGPTCPFAGSCEVSKTQRRHCPACRLQKCLDAGMRKDMILSAEALALRRAKQAQRRAQQTPVQLSKEQEELIRTLLGAHTRHMGTMFEQFVQFRPPAHLFIHHQPLPTLAPVLPLVTHFADINTFMVLQVIKFTKDLPVFRSLPIEDQISLLKGAAVEICHIVLNTTFCLQTQNFLCGPLRYTIEDGARVGFQVEFLELLFHFHGTLRKLQLQEPEYVLLAAMALFSPAPYLTDRPGVTQRDEIDQLQEEMALTLQSYIKGQQRRPRDRSPGTPWIHWSGKMLGPKIGPGSKGAQWLQ

>NP_001070946.1 nuclear receptor subfamily 1 group I member 3 isoform 7 [Homo sapiens]

MASREDELRNCVVCGDQATGYHFNALTCEGCKGFFRRTVSKSIGPTCPFAGSCEVSKTQRRHCPACRLQKCLDAGMRKDMILSAEALALRRAKQAQRRAQQTPVQLSKEQEELIRTLLGAHTRHMGTMFEQFVQFRPPAHLFIHHQPLPTLAPVLPLVTHFADINTFMVLQVIKFTKDLPVFRSLPIEDQISLLKGAAVEICHIVLNTTFCLQTQNFLCGPLRYTIEDGARVSPTVGFQVEFLELLFHFHGTLRKLQLQEPEYVLLAAMALFSPDRPGVTQRDEIDQLQEEMALTLQSYIKGQQRRPRDRSPGTPWIHWSGKMLGPKIGPGSKGAQWLQ

>NP_001070945.1 nuclear receptor subfamily 1 group I member 3 isoform 14 [Homo sapiens]

MLPKRSRRTVSKSIGPTCPFAGSCEVSKTQRRHCPACRLQKCLDAGMRKDMILSAEALALRRAKQAQRRAQQTPVQLSKEQEELIRTLLGAHTRHMGTMFEQFVQFRPPAHLFIHHQPLPTLAPVLPLVTHFADINTFMVLQVIKFTKDLPVFRSLPIEDQISLLKGAAVEICHIVLNTTFCLQTQNFLCGPLRYTIEDGARVGFQVEFLELLFHFHGTLRKLQLQEPEYVLLAAMALFSPDRPGVTQRDEIDQLQEEMALTLQSYIKGQQRRPRDRSPGTPWIHWSGKMLGPKIGPGSKGAQWLQ

>NP_001070947.1 nuclear receptor subfamily 1 group I member 3 isoform 10 [Homo sapiens]

MLPKRSRRTVSKSIGPTCPFAGSCEVSKTQRRHCPACRLQKCLDAGMRKDMILSAEALALRRAKQAQRRAQQTPVQLSKEQEELIRTLLGAHTRHMGTMFEQFVQFRPPAHLFIHHQPLPTLAPVLPLVTHFADINTFMVLQVIKFTKDLPVFRSLPIEDQISLLKGAAVEICHIVLNTTFCLQTQNFLCGPLRYTIEDGARVGFQVEFLELLFHFHGTLRKLQLQEPEYVLLAAMALFSPDRPGVTQRDEIDQLQEEMALTLQSYIKGQQRRPRDRFLYAKLLGLLAELRSINEAYGYQIQHIQGLSAMMPLLQEICS

>NP_001070948.1 nuclear receptor subfamily 1 group I member 3 isoform 2 [Homo sapiens]

MASREDELRNCVVCGDQATGYHFNALTCEGCKGFFRRTVSKSIGPTCPFAGSCEVSKTQRRHCPACRLQKCLDAGMRKDMILSAEALALRRAKQAQRRAQQTPVQLSKEQEELIRTLLGAHTRHMGTMFEQFVQFRPPAHLFIHHQPLPTLAPVLPLVTHFADINTFMVLQVIKFTKDLPVFRSLPIEDQISLLKGAAVEICHIVLNTTFCLQTQNFLCGPLRYTIEDGARVSPTVGFQVEFLELLFHFHGTLRKLQLQEPEYVLLAAMALFSPDRPGVTQRDEIDQLQEEMALTLQSYIKGQQRRPRDRFLYAKLLGLLAELRSINEAYGYQIQHIQGLSAMMPLLQEICS

>NP_001070949.1 nuclear receptor subfamily 1 group I member 3 isoform 4 [Homo sapiens]

MASREDELRNCVVCGDQATGYHFNALTCEGCKGFFRRTVSKSIGPTCPFAGSCEVSKTQRRHCPACRLQKCLDAGMRKDMILSAEALALRRAKQAQRRAQQTPVQLSKEQEELIRTLLGAHTRHMGTMFEQFVQFRPPAHLFIHHQPLPTLAPVLPLVTHFADINTFMVLQVIKFTKDLPVFRSLPIEDQISLLKGAAVEICHIVLNTTFCLQTQNFLCGPLRYTIEDGARAPYLTDRPGVTQRDEIDQLQEEMALTLQSYIKGQQRRPRDRFLYAKLLGLLAELRSINEAYGYQIQHIQGLSAMMPLLQEICS

>NP_001070950.1 nuclear receptor subfamily 1 group I member 3 isoform 1 [Homo sapiens]

MASREDELRNCVVCGDQATGYHFNALTCEGCKGFFRRTVSKSIGPTCPFAGSCEVSKTQRRHCPACRLQKCLDAGMRKDMILSAEALALRRAKQAQRRAQQTPVQLSKEQEELIRTLLGAHTRHMGTMFEQFVQFRPPAHLFIHHQPLPTLAPVLPLVTHFADINTFMVLQVIKFTKDLPVFRSLPIEDQISLLKGAAVEICHIVLNTTFCLQTQNFLCGPLRYTIEDGARVSPTVGFQVEFLELLFHFHGTLRKLQLQEPEYVLLAAMALFSPAPYLTDRPGVTQRDEIDQLQEEMALTLQSYIKGQQRRPRDRFLYAKLLGLLAELRSINEAYGYQIQHIQGLSAMMPLLQEICS

>AAI27674.1 KCNH2 protein [Homo sapiens]

MAAPAGKASRTGALRPRAQKGRVRRAVRISSLVAQEVLSLGADVLPEYKLQAPRIHRWTILHYSPFKAVWDWLILLLVIYTAVFTPYSAAFLLKETEEGPPATECGYACQPLAVVDLIVDIMFIVDILINFRTTYVNANEEVVSHPGRIAVHYFKGWFLIDMVAAIPFDLLIFGSGSEELIGLLKTARLLRLVRVARKLDRYSEYGAAVLFLLMCTFALIAHWLACIWYAIGNMEQPHMDSRIGWLHNLGDQIGKPYNSSGLGGPSIKDKYVTALYFTFSSLTSVGFGNVSPNTNSEKIFSICVMLIGSLMYASIFGNVSAIIQRLYSGTARYHTQMLRVREFIRFHQIPNPLRQCCDHPGAWPHFHIPAVARQPPPHPHLGLAFSGFPVHGV

>EAW57795.1 modifier of the HERG potassium channel [Homo sapiens]

MAQLEGYCFSAALSCTFLVSCLLFSAFSRALREPYMDEIFHLPQAQRYCEGHFSLSQWDPMITTLPGLYLVSVGVVKPAIWIFGWSEHVVCSIGMLRFVNLLFSVGNFYLLYLLFHKVQPRNKAASSIQRVLSTLTLAVFPTLYFFNFLYYTEAGSMFFTLFAYLMCLYGNHKTSAFLGFCGFMFRQTNIIWAVFCAGNVIAQKLTEAWKTELQKKEDRLPPIKGPFAEFRKILQFLLAYSMSFKNLSMLFCLTWPYILLGFLFCAFVVVNGGIVIGDRSSHEACLHFPQLFYFFSFTLFFSFPHLLSPSKIKTFLSLVWKHGILFLVVTLVSVFLVWKFTYAHKYLLADNRHYTFYVWKRVFQRYAILKYLLVPAYIFAGWSIADSLKSKPIFWNLMFFICLFIVIVPQKLLEFRYFILPYVIYRLNITLPPTSRLVCELSCYAIVNFITFYIFLNKTFQWPNSQDIQRFMW

>EAW67219.1 dopamine receptor D2, isoform CRA_a [Homo sapiens]

MRRELEASSSRRRLCPRAPYGLAWPPSGSTALMDPLNLSWYDDDLERQNWSRPFNGSDGKADRPHYNYYATLLTLLIAVIVFGNVLVCMAVSREKALQTTTNYLIVSLAVADLLVATLVMPWVVYLEVVGEWKFSRIHCDIFVTLDVMMCTASILNLCAISIDRYTAVAMPMLYNTRYSSKRRVTVMISIVWVLSFTISCPLLFGLNNADQNECIIANPAFVVYSSIVSFYVPFIVTLLVYIKIYIVLRRRRKRVNTKRSSRAFRAHLRAPLKGNCTHPEDMKLCTVIMKSNGSFPVNRRRVSPLPLVQEAARRAQELEMEMLSSTSPPERTRYSPIPPSHHQLTLPDPSHHGLHSTPDSPAKPEKNGHAKDHPKIAKIFEIQTMPNGKTRTSLKTMSRRKLSQQKEKKATQMLAIVLGVFIICWLPFFITHILNIHCDCNIPPVLYSAFTWLGYVNSAVNPIIYTTFNIEFRKAFLKILHC

>EAW67223.1 dopamine receptor D2, isoform CRA_d [Homo sapiens]

MRRELEASSSRRRLCPRAPYGLAWPPSGSTALMDPLNLSWYDDDLERQNWSRPFNGSDGKADRPHYNYYATLLTLLIAVIVFGNVLVCMAVSREKALQTTTNYLIVSLAVADLLVATLVMPWVVYLEVVGEWKFSRIHCDIFVTLDVMMCTASILNLCAISIDRYTAVAMPMLYNTRYSSKRRVTVMISIVWVLSFTISCPLLFGLNNADQNECIIANPAFVVYSSIVSFYVPFIVTLLVYIKIYIVLRRRRKRVNTKRSSRAFRAHLRAPLKEAARRAQELEMEMLSSTSPPERTRYSPIPPSHHQLTLPDPSHHGLHSTPDSPAKPEKNGHAKDHPKIAKIFEIQTMPNGKTRTSLKTMSRRKLSQQKEKKATQMLAIVLGVFIICWLPFFITHILNIHCDCNIPPVLYSAFTWLGYVNSAVNPIIYTTFNIEFRKAFLKILHC

>EAW67225.1 dopamine receptor D2, isoform CRA_e [Homo sapiens]

MRRELEASSSRRRLCPRAPYGLAWPPSGSTALMDPLNLSWYDDDLERQNWSRPFNGSDGKADRPHYNYYATLLTLLIAVIVFGNVLVCMAVSREKALQTTTNYLIVSLAVADLLVATLVMPWVVYLEVVGEWKFSRIHCDIFVTLDVMMCTASILNLCAISIDRYTAVAMPMLYNTRYSSKRRVTVMISIVWVLSFTISCPLLFGLNNADQNECIIANPAFVVYSSIVSFYVPFIVTLLVYIKIYIVLRRRRKRVNTKRSSRAFRAHLRAPLKGNCTHPEDMKLCTVIMKSNGSFPVNRRRVEAARRAQELEMEMLSSTSPPERTRYSPIPPSHHQLTLPDPSHHGLHSTPDSPAKPEKNGHAKDHPKIAKIFEIQTMPNGKTRTSLKTMSRRKLSQQKEKKATQMLAIVLGVFIICWLPFFITHILNIHCDCNIPPVLYSAFTWLGYVNSAVNPIIYTTFNIEFRKAFLKILHC

>pdb|2O9I|B Chain B, Orphan nuclear receptor PXR

GLTEEQRMMIRELMDAQMKTFDTTFSHFKNFRLPGVLSSGCELPESLQAPSREEAAKWSQVRKDLCSLKVSLQLRGEDGSVWNYKPPADSGGKEIFSLLPHMADMSTYMFKGIISFAKVISYFRDLPIEDQISLLKGAAFELSQLRFNTVFNAETGTWECGRLSYCLEDTAGGFQQLLLEPMLKFHYMLKKLQLHEEEYVLMQAISLFSPDRPGVLQHRVVDQLQEQFAITLKSYIECNRPQPAHRFLFLKIMAMLTELRSINAQHTQRLLRIQDIHPFATPLMQELFGITGS

>sp|P21439.2|MDR3_HUMAN RecName: Full=Phosphatidylcholine translocator ABCB4; AltName: Full=ATP-binding cassette sub-family B member 4; AltName: Full=Multidrug resistance protein 3; AltName: Full=P-glycoprotein 3

MDLEAAKNGTAWRPTSAEGDFELGISSKQKRKKTKTVKMIGVLTLFRYSDWQDKLFMSLGTIMAIAHGSGLPLMMIVFGEMTDKFVDTAGNFSFPVNFSLSLLNPGKILEEEMTRYAYYYSGLGAGVLVAAYIQVSFWTLAAGRQIRKIRQKFFHAILRQEIGWFDINDTTELNTRLTDDISKISEGIGDKVGMFFQAVATFFAGFIVGFIRGWKLTLVIMAISPILGLSAAVWAKILSAFSDKELAAYAKAGAVAEEALGAIRTVIAFGGQNKELERYQKHLENAKEIGIKKAISANISMGIAFLLIYASYALAFWYGSTLVISKEYTIGNAMTVFFSILIGAFSVGQAAPCIDAFANARGAAYVIFDIIDNNPKIDSFSERGHKPDSIKGNLEFNDVHFSYPSRANVKILKGLNLKVQSGQTVALVGSSGCGKSTTVQLIQRLYDPDEGTINIDGQDIRNFNVNYLREIIGVVSQEPVLFSTTIAENICYGRGNVTMDEIKKAVKEANAYEFIMKLPQKFDTLVGERGAQLSGGQKQRIAIARALVRNPKILLLDEATSALDTESEAEVQAALDKAREGRTTIVIAHRLSTVRNADVIAGFEDGVIVEQGSHSELMKKEGVYFKLVNMQTSGSQIQSEEFELNDEKAATRMAPNGWKSRLFRHSTQKNLKNSQMCQKSLDVETDGLEANVPPVSFLKVLKLNKTEWPYFVVGTVCAIANGGLQPAFSVIFSEIIAIFGPGDDAVKQQKCNIFSLIFLFLGIISFFTFFLQGFTFGKAGEILTRRLRSMAFKAMLRQDMSWFDDHKNSTGALSTRLATDAAQVQGATGTRLALIAQNIANLGTGIIISFIYGWQLTLLLLAVVPIIAVSGIVEMKLLAGNAKRDKKELEAAGKIATEAIENIRTVVSLTQERKFESMYVEKLYGPYRNSVQKAHIYGITFSISQAFMYFSYAGCFRFGAYLIVNGHMRFRDVILVFSAIVFGAVALGHASSFAPDYAKAKLSAAHLFMLFERQPLIDSYSEEGLKPDKFEGNITFNEVVFNYPTRANVPVLQGLSLEVKKGQTLALVGSSGCGKSTVVQLLERFYDPLAGTVFVDFGFQLLDGQEAKKLNVQWLRAQLGIVSQEPILFDCSIAENIAYGDNSRVVSQDEIVSAAKAANIHPFIETLPHKYETRVGDKGTQLSGGQKQRIAIARALIRQPQILLLDEATSALDTESEKVVQEALDKAREGRTCIVIAHRLSTIQNADLIVVFQNGRVKEHGTHQQLLAQKGIYFSMVSVQAGTQNL

>NP_115984.3 neurabin-2 [Homo sapiens]

MMKTEPRGPGGPLRSASPHRSAYEAGIQALKPPDAPGPDEAPKGAHHKKYGSNVHRIKSMFLQMGTTAGPSGEAGGGAGLAEAPRASERGVRLSLPRASSLNENVDHSALLKLGTSVSERVSRFDSKPAPSAQPAPPPHPPSRLQETRKLFERSAPAAAGGDKEAAARRLLRQERAGLQDRKLDVVVRFNGSTEALDKLDADAVSPTVSQLSAVFEKADSRTGLHRGPGLPRAAGVPQVNSKLVSKRSRVFQPPPPPPPAPSGDAPAEKERCPAGQQPPQHRVAPARPPPKPREVRKIKPVEVEESGESEAESAPGEVIQAEVTVHAALENGSTVATAASPAPEEPKAQAAPEKEAAAVAPPERGVGNGRAPDVAPEEVDESKKEDFSEADLVDVSAYSGLGEDSAGSALEEDDEDDEEDGEPPYEPESGCVEIPGLSEEEDPAPSRKIHFSTAPIQVFSTYSNEDYDRRNEDVDPMAASAEYELEKRVERLELFPVELEKDSEGLGISIIGMGAGADMGLEKLGIFVKTVTEGGAAHRDGRIQVNDLLVEVDGTSLVGVTQSFAASVLRNTKGRVRFMIGRERPGEQSEVAQLIQQTLEQERWQREMMEQRYAQYGEDDEETGEYATDEDEELSPTFPGGEMAIEVFELAENEDALSPVDMEPEKLVHKFKELQIKHAVTEAEIQQLKRKLQSLEQEKGRWRVEKAQLEQSVEENKERMEKLEGYWGEAQSLCQAVDEHLRETQAQYQALERKYSKAKRLIKDYQQKEIEFLKKETAQRRVLEESELARKEEMDKLLDKISELEGNLQTLRNSNST

>NP_005151.2 beta-adrenergic receptor kinase 2 [Homo sapiens]

MADLEAVLADVSYLMAMEKSKATPAARASKRIVLPEPSIRSVMQKYLAERNEITFDKIFNQKIGFLLFKDFCLNEINEAVPQVKFYEEIKEYEKLDNEEDRLCRSRQIYDAYIMKELLSCSHPFSKQAVEHVQSHLSKKQVTSTLFQPYIEEICESLRGDIFQKFMESDKFTRFCQWKNVELNIHLTMNEFSVHRIIGRGGFGEVYGCRKADTGKMYAMKCLDKKRIKMKQGETLALNERIMLSLVSTGDCPFIVCMTYAFHTPDKLCFILDLMNGGDLHYHLSQHGVFSEKEMRFYATEIILGLEHMHNRFVVYRDLKPANILLDEHGHARISDLGLACDFSKKKPHASVGTHGYMAPEVLQKGTAYDSSADWFSLGCMLFKLLRGHSPFRQHKTKDKHEIDRMTLTVNVELPDTFSPELKSLLEGLLQRDVSKRLGCHGGGSQEVKEHSFFKGVDWQHVYLQKYPPPLIPPRGEVNAADAFDIGSFDEEDTKGIKLLDCDQELYKNFPLVISERWQQEVTETVYEAVNADTDKIEARKRAKNKQLGHEEDYALGKDCIMHGYMLKLGNPFLTQWQRRYFYLFPNRLEWRGEGESRQNLLTMEQILSVEETQIKDKKCILFRIKGGKQFVLQCESDPEFVQWKKELNETFKEAQRLLRRAPKFLNKPRSGTVELPKPSLCHRNSNGL

>NP_001009571.2 calcium-dependent secretion activator 2 isoform b [Homo sapiens]
[truncated: 10,764 more chars]
